# Dispersal history of *Miniopterus fuliginosus* bats and their associated viruses in east Asia

**DOI:** 10.1371/journal.pone.0244006

**Published:** 2021-01-14

**Authors:** Thachawech Kimprasit, Mitsuo Nunome, Keisuke Iida, Yoshitaka Murakami, Min-Liang Wong, Chung-Hsin Wu, Ryosuke Kobayashi, Yupadee Hengjan, Hitoshi Takemae, Kenzo Yonemitsu, Ryusei Kuwata, Hiroshi Shimoda, Lifan Si, Joon-Hyuk Sohn, Susumu Asakawa, Kenji Ichiyanagi, Ken Maeda, Hong-Shik Oh, Tetsuya Mizutani, Junpei Kimura, Atsuo Iida, Eiichi Hondo

**Affiliations:** 1 Laboratory of Animal Morphology, Graduate School of Bioagricultural Sciences, Nagoya University, Nagoya, Japan; 2 Avian Bioscience Research Center, Graduate School of Bioagricultural Sciences, Nagoya University, Nagoya, Japan; 3 Department of Medical Statistics, Toho University, Tokyo, Japan; 4 Department of Veterinary Medicine, National Chung Hsing University, Taichung, Taiwan; 5 Department of Life Science, National Taiwan Normal University, Taipei, Taiwan; 6 Laboratory of Veterinary Microbiology, Joint Faculty of Veterinary Medicine, Yamaguchi University, Yamaguchi, Japan; 7 College of Animal Science and Technology, Henan University of Science and Technology, Luoyang, Henan, China; 8 Laboratory of Anatomy and Cell Biology and Research Institute for Veterinary Science, College of Veterinary Medicine, Seoul National University, Seoul, Korea; 9 Laboratory of Soil Biology and Chemistry, Graduate School of Bioagricultural Sciences, Nagoya University, Nagoya, Japan; 10 Laboratory of Genome and Epigenome Dynamics, Graduate School of Bioagricultural Sciences, Nagoya University, Nagoya, Japan; 11 Institute of Science Education, Jeju National University, Jeju, Korea; 12 Research and Education Center for Prevention of Global Infectious Diseases of Animals, Tokyo University of Agriculture and Technology, Tokyo, Japan; University of Reunion Island, RÉUNION

## Abstract

In this study, we examined the role of the eastern bent-winged bat *(Miniopterus fuliginosus)* in the dispersion of bat adenovirus and bat alphacoronavirus in east Asia, considering their gene flows and divergence times (based on deep-sequencing data), using bat fecal guano samples. Bats in China moved to Jeju Island and/or Taiwan in the last 20,000 years via the Korean Peninsula and/or Japan. The phylogenies of host mitochondrial D-loop DNA was not significantly congruent with those of bat adenovirus (m^2^_XY_ = 0.07, *p* = 0.08), and bat alphacoronavirus (m^2^_XY_ = 0.48, *p* = 0.20). We estimate that the first divergence time of bats carrying bat adenovirus in five caves studied (designated as K1, K2, JJ, N2, and F3) occurred approximately 3.17 million years ago. In contrast, the first divergence time of bat adenovirus among bats in the 5 caves was estimated to be approximately 224.32 years ago. The first divergence time of bats in caves CH, JJ, WY, N2, F1, F2, and F3 harboring bat alphacoronavirus was estimated to be 1.59 million years ago. The first divergence time of bat alphacoronavirus among the 7 caves was estimated to be approximately 2,596.92 years ago. The origin of bat adenovirus remains unclear, whereas our findings suggest that bat alphacoronavirus originated in Japan. Surprisingly, bat adenovirus and bat alphacoronavirus appeared to diverge substantially over the last 100 years, even though our gene-flow data indicate that the eastern bent-winged bat serves as an important natural reservoir of both viruses.

## Introduction

Bats have been considered to be natural reservoirs for many zoonotic viruses, including lyssaviruses [1], filoviruses [2, 3], henipaviruses [4], picornaviruses [5], coronaviruses [6, 7], and adenoviruses [8]. Infected bats exhibit few clinical symptoms for some viral infections, following natural and/or experimental infection [3, 9].

Bat adenovirus (BtAdV), which belong to the *Mastadenovirus* genus, was first isolated from *Pteropus dasymallus yayeyamae* in Okinawa, Japan [10]. Subsequently, the virus has been isolated from various bat species in several geographical regions [11–13]. Recently, BtAdV have been isolated from bats in *Miniopteridae* family inhabited in China [13]. At present, 7 species of BtAdV (namely bat *mastadenovirus* A–G) have been described by the International Committee on Taxonomy of Viruses and are classified into three groups, depending on the host; group 1 viruses include bat *mastadenovirus* A, B, and G from *Vespertilionidae* bats; group 2 include bat *mastadenovirus* C isolated from *Rhinolophidae* bats; and group 3 include bat *mastadenovirus* D, E, and F isolated from bats in the *Miniopteridae* and *Pteropodidae* families [14].

Alphacoronavirus (AlphaCoV) is one of the four genera of *Coronaviridae* family. This virus has been reported to infect both humans and other mammals including bats [15]. AlphaCoV is believed to has been evolving in bats over a long period of time [16]. There are at least four closely related alphaCoV detected in bent-winged bats (*Miniopterus spp*.) including bat alphaCoV 1A, 1B, HKU7, and HKU8 [16]. Recently, some strains of bat alphaCoV were detected in *M*. *fuliginosus* living in a cave located in Wakayama, Japan [17]. Furthermore, a novel alphaCoV, BtCoV/Rh/YN2012, was detected in a *Rhinolophidae* dwelling in China [18]. Thus, the *Miniopteridae* and *Rhinolophidae* families have been considered to be reservoirs of alphaCoV species.

The eastern bent-winged bat *(Miniopterus fuliginosus)* is an insectivorous bat that inhabits caves throughout east Asia [19]. The species often share roosting sites with *Rhinolophus ferrumequinum* [20, 21]. *M*. *fuliginosus* can fly long distances to change roosting sites between the summer and winter. Some of them can travel over 200 km [22–24]. This suggests that pathogens of *M*. *fuliginosus* can be shared with *R*. *ferrumequinum*, and be spread in wide area by *M*. *fuliginosus*. *M*. *fuliginosus* bats have been observed in China, Jeju Island, Taiwan, and Japan, and shared 100% identity between their mitochondrial D-loop DNA sequences [25, 26]. The mitochondrial D-loop is a non-coding DNA region with a high mutation rate and is used to examine genetic difference among individuals in the same species of animals [27]. This species might have undergone dynamic movement throughout east Asia [25].

Satellite tracking systems have been used to reveal the wide movement of megabats [28–30]. However, the systems cannot be applied to small insectivorous bats because of heavy weight of telemetry machines. Additionally, they are not suitable to investigate the movement of many bats because deploying devices on several bats can be difficult. Pathogens migrated along with their hosts [31], thus it is beneficial to know the gene flow and genetic compositions of *M*. *fuliginosus* among different populations, to enable tracking the movement of pathogens between different countries.

We hypothesized that if *M*. *fuliginosus* has undergone dynamic movement throughout east Asia and carried BtAdV and bat alphaCoV, genetic co-variation between host populations and the viruses should be detectable. In this study, we examined gene flows and genetic diversities of *M*. *fuliginosus* throughout east Asia together with their associated viruses, BtAdV and bat alphaCoV.

## Materials and methods

### Sample collection

Bat fecal guanos were carefully collected on the ground in the cave to limit disturbance of bats as possible as we could. This sampling procedure did not require ethical approval according to the policy of animal experiment in Nagoya University, Japan. Also, we did not need permission to access the caves in each country, because we did not catch any animal inside the caves.

**Fig 1:**
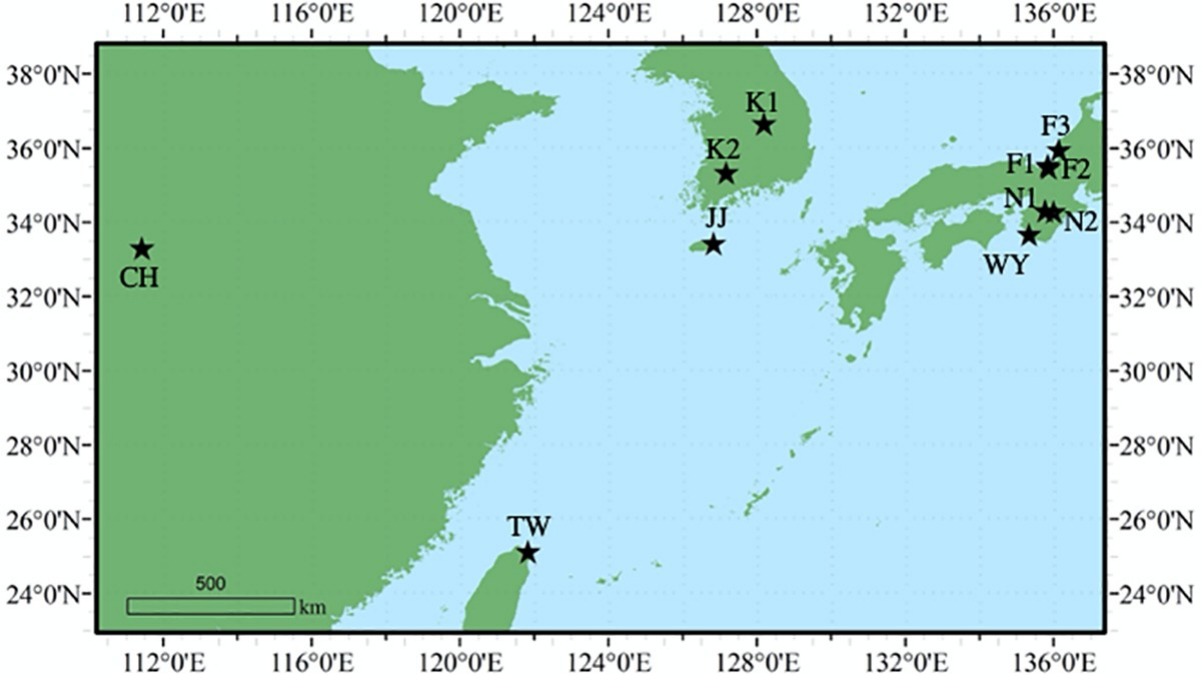
Sampling locations of 11 populations of *M*. *fuliginosus* in east Asia. The abbreviations of each sampling location are referred to Table 1.

In August 2017, bat fecal guano samples were collected from 11 caves, including six sites in Japan, two sites on the Korean Peninsula, and one site each in Jeju Island, China, and Taiwan (Fig 1 and Table 1). *M*. *fuliginosus* and *R*. *ferrumequinum* live together in these caves. To accurately evaluate the entire gene pooled, guano was collected randomly at several locations of the ground in each cave and/or around the entries of the caves, and then pooled for a total of 150 to 250 g guano per cave. The samples were kept on ice during transportation, and stored at -80°C until use.

**Table 1:** Global-Positioning System (GPS) coordinates, population size estimated using Migrate-n analysis, the number of D-loop haplotypes of *M*. *fuliginosus*, and GenBank accession numbers of D-loop sequences.

Population	GPS	Number of haplotypes	Population size	GenBank accession number
Henan, China (CH)	33.306N, 111.431E	1,519	8.71 ×10^−3^	MT684783-MT686301
North Gyeongsang, Korea (K1)	36.659N, 128.193E	1,493	8.92 ×10^−3^	MT686302-MT687794
North Jeolla, Korea (K2)	35.336N, 127.178E	1,468	8.85 ×10^−3^	MT687795-MT689262
Jeju Island, Korea (JJ)	33.436N, 126.838E	1,274	7.15 ×10^−3^	MT689263-MT690536
New Taipei, Taiwan (TW)	25.126N, 121.833E	1,265	7.09 ×10^−3^	MT690537-MT691801
Wakayama, Japan (WY)	33.672N, 135.331E	1,314	7.14 ×10^−3^	MT691802-MT693115
Nara, Japan (N1)	34.316N, 135.774E	1,388	7.05 ×10^−3^	MT693116-MT694503
Nara, Japan (N2)	34.284N, 136.002E	1,384	8.20 ×10^−3^	MT694504-MT695887
Fukui, Japan (F1)	35.473N, 135.865E	1,327	7.50 ×10^−3^	MT695888-MT697214
Fukui, Japan (F2)	35.531N, 135.839E	1,526	9.26 ×10^−3^	MT697215-MT698740
Fukui, Japan (F3)	35.948N, 136.135E	1,509	8.46 ×10^−3^	MT698741-MT700249

### Detection of mitochondrial D-loop DNA of *M*. *fuliginosus*

Mitochondrial DNA was extracted from the samples using the QIAamp DNA Stool Mini Kit (Qiagen, Hilden, Germany). The D-loop region was amplified from each sample by performing nested-polymerase chain reaction (PCR) runs, using two sets of specific primers for the eastern bent-winged bat. The sequences of the primers in the 1^st^ set were 5′-CCCATCTGATATAGATGCCA-3′ and 5′-TACAGCTTAGCCAAGGCTTA-3′, and those in the 2^nd^ set were 5′-TACACTGGTCTTGTAAACCA-3′ and 5′-TTCGGGGATACTTGCA TGTA-3′. PCR was performed using KOD Plus Polymerase (Toyobo, Japan). The PCR amplification protocol was as follows: 94°C for 2 min, followed by 35 cycles of 10 s at 98°C, 1 min 30 s at 55°C and 2 min at 68°C. The PCR products were subjected to agarose gel electrophoresis. Each target band (1,500 base pairs long) was excised from the gel, and the DNA was cleaned up using QIAquick^®^ Gel Extraction Kit (Qiagen).

### Detection of BtAdV and bat alphaCoV

Total viral nucleic acids were extracted from the bat fecal samples using the High Pure Viral Nucleic Acid Extraction Kit (Roche, Germany).

For BtAdV, the viral DNA was amplified by nested PCR using the following degenerate primers designed by our team targeting the *hexon* gene (nucleotide position, 17,800–18,091): 5′-GCTTCHACYYTRGAAGCTATG-3′ and 5′-CAAMARYCTRTCATTWCCYG GCCA-3′ (first round) and 5′-GCTTCHACYYTRGAAGCTATG-3′ and 5′-GAATKGMAAC TCTYCTRAARGTATG-3′ (second round). Thermocycling was performed using KOD Plus Polymerase (Toyobo, Japan). The PCR amplification protocol was as follows: 2 min at 94°C, followed by 35 cycles of 10 s at 98°C, 30 s at 50°C and 30 sec at 68°C.

Bat alphaCoV RNA was reverse transcribed using SuperScript IV Reverse Transcriptase (Invitrogen^TM^, USA) with random hexamers. Then, the resulting complementary DNA was amplified by nested-PCR using the following primer sets specific for the *RdRp* gene (nucleotide position, 12,670–12,943): 5′-AAYCARGATWSTTATGGTGGTGC-3′ and 5′-TC HGGTTCAGTRCCATTACAGG-3′ (first round) and 5′-AAYCARGATWSTTATGGTGGT GC-3′ and 5′-TCTAGTCGAGMTGCACTAGAG-3′ (second round). Thermocycling was performed using GoTaq^®^ Green Master Mix (Promega, USA). The PCR amplification protocol was as follows: 2 min at 95°C, followed by 35 cycles of 30 s at 95°C, 30 s at 50°C and 30 sec at 72°C, and terminal incubation at 72°C for 5 min.

The PCR products were subjected to agarose gel electrophoresis. The target bands (292 base pairs for BtAdV, and 274 base pairs for bat alphaCoV) were excised from the gel and cleaned up using the QIAquick^®^ Gel Extraction Kit (Qiagen).

### Next-Generation Sequencing (NGS) of bat mitochondrial D-loop DNA and viral fragment

Multiple haplotypes of the viruses and their host were expected to be present within the same guano samples. Therefore, the NGS was used to sequence the host and viral PCR products. The PCR products mentioned above were purified with the Agencourt AMPure XP reagent (Beckman Coulter, Brea, Calif., USA). An NGS library was constructed using the NEBNext® Ultra™ RNA Library Prep Kit for Illumina® (New England BioLabs, Massachusetts, USA). Sequencing of paired ends was performed using the MiSeq Reagent Kit v2 (300 cycles) (Illumina®, San Diego, CA, USA), wherein each read was exported from MiSeq Reporter software in FASTQ format, and the NGS data were analyzed using CLC Genomics Workbench software, version 10.1.1 (Filgen, Nagoya, Japan). The overlapped sequence from each end was connected (Mismatch cost = 2, Gap cost = 3, max unaligned mismatches = 0, Minimum score = 6). Then, the sequences were mapped onto reference sequences obtained from the NCBI database. The mapped sequences with 96.44%-100%, similarity to each other were defined as one haplotype of *M fuliginosus* D-loop DNA [32]. On the other hand, the viral sequences with 100% similarity to each other were defined as one haplotype of either BtAdV or bat alphaCoV. GenBank accession numbers of all sequencing data of the mitochondrial D-loop DNA, and both of the viruses were presented in Tables 1 and 2, respectively.

**Table 2:** Population size estimated using migrate-n analysis, number of haplotypes and GenBank accession numbers of bat alphacoronavirus and bat adenovirus.

Sampling location	number of haplotypes	population size	GenBank accession number
Bat Alphacoronavirus			
Henan, China (CH)	10	1.87×10^−2^	MT708742-MT708714
Jeju Island, Korea (JJ)	3	1.70×10^−2^	MT708752-MT708754
Wakayama, Japan (WY)	10	1.72×10^−2^	MT708755-MT708764
Nara, Japan (N2)	3	1.57×10^−2^	MT708765-MT708767
Fukui, Japan (F1)	2	1.72×10^−2^	MT708768-MT708769
Fukui, Japan (F2)	8	1.98×10^−2^	MT708770-MT708777
Fukui, Japan (F3)	8	1.35×10^−2^	MT708778-MT708785
Bat Adenovirus			
Jeju Island, Korea (JJ)	10	2.60×10^−2^	MT708724-MT708733
North Gyeongsang, Korea (K1)	8	2.63×10^−2^	MT708707-MT708714
North Jeolla, Korea (K2)	9	2.42×10^−2^	MT708715-MT708723
Nara, Japan (N2)	4	2.57×10^−2^	MT708734-MT708737
Fukui, Japan (F3)	4	2.52×10^−2^	MT708738-MT708741

### Analysis of molecular variance (AMOVA)

Analysis of molecular variance (AMOVA) was performed using Arlequin software, version 3.5.2.2 [33] for five groups of *M*. *fuliginosus* populations, which were China, South Korea, Jeju island, Japan, and Taiwan to verify how large the genetic variations in these species were attributed to among groups and within groups.

### Mantel test

Geographic distances among 11 caves were calculated using the geodist package in program R (http://cran.r-project.org). A Mantel test [34] was performed using Arlequin software, version 3.5.2.2 [33] to examine correlations between genetic (*Φ*_*ST*_) and geographic distance among populations. Significance of the correlation value was evaluated using 1,000 permutation tests.

### Determination of host genetic structure and gene flow

Bat mitochondrial D-loop DNA and viral sequences were investigated in terms of gene flow, using Migrate-n software [35]. A CX400 Super Computer System (Fujitsu, Tokyo, Japan) was used for calculations requiring the Migrate-n software. The parameters used for the Migrate-n simulations were modified and optimized three times before the desired data were obtained (S1 Table). The mutation rate/generation between each population was calculated. Based on the calculated mutation rate, the population size in each cave and the gene flow among the caves were estimated. Genetic relationships among populations were inferred based on the pairwise *Φ*_*ST*_ genetic distance [36], which was calculated using Arlequin software, version 3.5.2.2 [33]. The results of the gene flow and the pairwise *Φ*_*ST*_ genetic distance derived from CH, K1, K2, JJ, TW, and WY were then subjected to determine migration pattern of *M*. *fuliginosus* among east Asian countries. To determine the migration pattern of *M*. *fuliginosus* in Japan, one cave in the Wakayama prefecture was selected as the central point of Japan. This strategy was chosen because the cave in Wakayama is known as a large-scale birthing and nursery roost for *M*. *fuliginosus* bats [37].

### Genetic correlations between bats and viruses

Correlation between *M*. *fuliginosus* phylogeny with each of their associated virus phylogeny was assessed using Procrustes Approach to Cophylogeny, PACo [38] in the R program [39]. PACo firstly constructs principal coordinate plots of a host (bats) and a parasite (viruses) based on the genetic distances between populations, and applies a Procrustes approach to examine a similarity between the plotting patterns of the two species. Goodness-of-fit test was performed to test the cophylogenetic patterns of *M*. *fuliginosus* and their associated virus with 100,000 permutations. The threshold for statistical significance was set at *p* < 0.05.

### Divergence-time estimation

When studying *M*. *fuliginosus* populations, the divergence times of bat alphaCoV and BtAdV were calculated using a Bayesian Markov Chain Monte Carlo (MCMC) approach, which was implemented using BEAST software, version 2.6.0 [40]. HKY+G and Coalescent constant size models were used to analyze the sequences of bat mitochondrial D-loop DNA, the *RdRp* fragment of bat alphaCoV, and the *hexon* fragment of BtAdV. The mean evolutionary rate of bat alphaCoV had previously been estimated to be 1.3 × 10^−4^ nucleotide substitutions/site/year based on the partial *RdRp* gene of coronaviruses [41]. Since no substitution rate was available for the *hexon* region of BtAdV, the substitution rate in the *hexon* gene of Human adenovirus-7, HAdV-7 (1.107 × 10^−3^ nucleotide substitutions/site/ year), which belongs to the same genus as BtAdV, was applied to calculate the divergence time of BtAdV found in this study [42]. The divergence time of the bats was estimated, based on an evolutionary rate for mitochondrial D-loop of 20% nucleotide substitution/site/million years. The MCMC run was 3× 10^7^ steps long, with sampling every 300 steps. The convergence of the MCMC run was checked using Tracer version 1.7.1. Higher ESS values for most of phylogenetic parameters were shown after discarding 95% of the sampled data as burn-in, but they are still very low. Thus, we carried out a MCMC run once again and found that the second MCMC run reached to the same values for each phylogenetic parameter as the first run. Because the MCMC run took a long time at once (more than one week) due to a huge number of bat D-loop sequences, we copied the last 5% of tree data ten times and combined them as a single tree file using the software, LogCombiner version 2.6.0. The Bayesian phylogenetic trees were constructed with Tree Annotator software, version 2.6.0 based on the combined tree data.

## Results

### Detection of mitochondrial D-loop sequences of *M*. *fuliginosus*

All of eleven bat populations exhibited the targeted band of the D-loop region of *M*. *fuliginosus* based on PCR detection. After purification, the PCR products were sequenced by NGS method. Number of D-loop haplotypes of each bat populations were presented in Table 1. In total, 15,467 of D-loop haplotypes obtained from the eleven populations were then subjected for genetic quantification and gene flow analyses.

### Genetic differences among bat populations

AMOVA indicated that more than half of the total genetic variations among eleven *M*. *fuliginosus* populations (approximately 53.0%) were attributed to the genetic variations within each population. Genetic differences between 11 populations and between five groups contributed to 27.5% and 19.5% of the total genetic variations, respectively (S2 Table). The result of Mantel tests (S3 Table) indicated that there was no significant correlation (*P>0*.*05*) between genetic and geographic distances for all of *M*. *fuliginosus* populations.

### Genetic structure of bats and gene flow in east Asia

**Fig 2:**
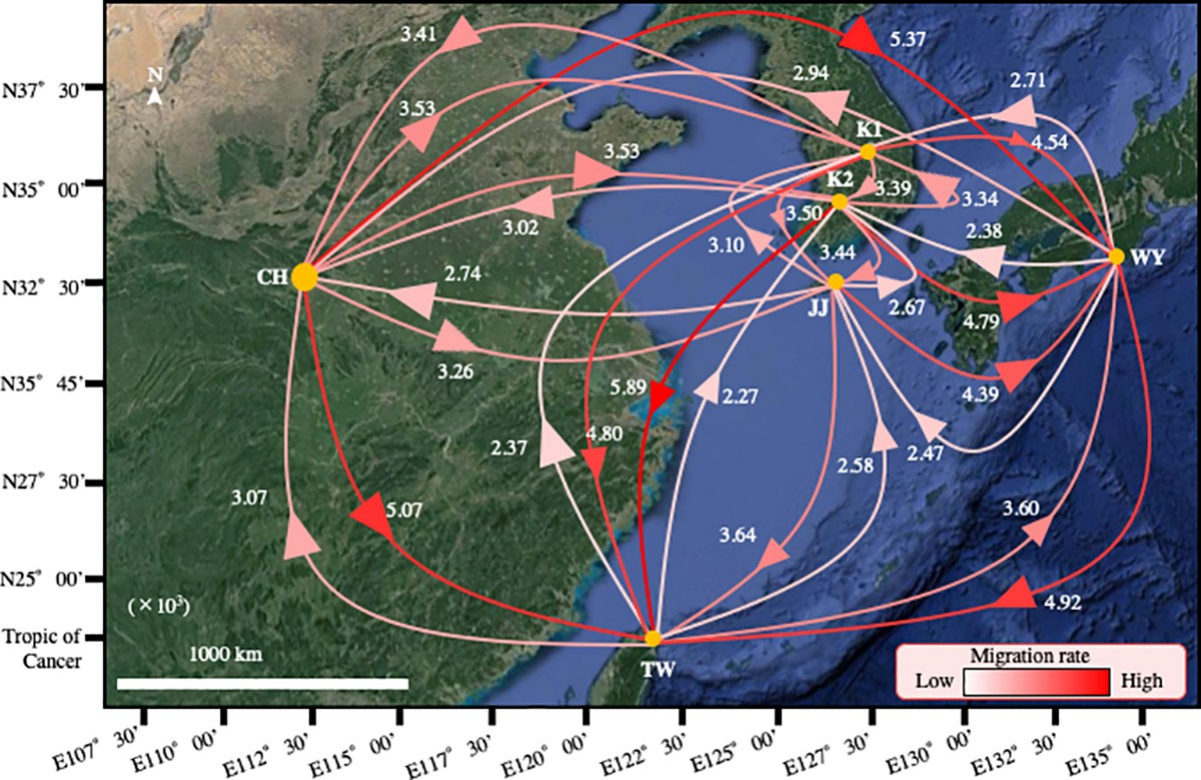
Gene flows among the populations of *M*. *fuliginosus* in east Asia. The gene flows were estimated, based on the D-loop amplicon-sequencing results, determined by analysis with Migrate-n software. The arrowheads on the lines show the directions of gene flows and the numbers near the arrowheads are the relative values of the gene flows (×10^3^).

Less gene flow of *M*. *fuliginosus* was observed from China to Korea than from China to Japan, and from China to Taiwan. In addition, gene flow from Korea to Japan, and from Korea to Taiwan exceeded that from Korea to China (Fig 2). The number of haplotypes and the population sizes in China and Korea were similar (Table 1). In addition, the *Φ*_*ST*_ genetic distance between China and Korea was small (Table 3).

**Table 3:** The *Φ*_*ST*_ genetic distance among the *M*. *fuliginosus* populations in east Asia.

	CH	K1	K2	JJ	TW	WY
CH						
K1	0.03					
K2	0.02	0.02				
JJ	0.29	0.30	0.27			
TW	0.41	0.42	0.39	0.18		
WY	0.74	0.75	0.73	0.47	0.48	

All values show significance at *P* ≤ 0.05.

Gene flow between the Korean and Jeju Island populations was smaller or comparable when compared with that between other countries (Fig 2). The number of haplotypes and the size of the population on Jeju Island were relatively small (Table 1).

Using Migrate-n analysis to compare gene flows from China and Korea to Japan revealed that the gene flow from China to Japan was larger (Fig 2). Additionally, the *Φ*_*ST*_ genetic distance between Japan and China was large (Table 3). The gene flows from Japan, Korea, and China to Taiwan were larger than the gene flows to Japan, Korea, and China (Fig 2). The *Φ*_*ST*_ genetic distances between Taiwan and Japan (TW-WY: 0.48), Taiwan and Korea (TW-K1: 0.42, TW-K2: 0.39), and Taiwan and China (TW-CH: 0.41) were almost equivalent (Table 3).

### Genetic structure of bats and gene flow in Japan

**Fig 3:**
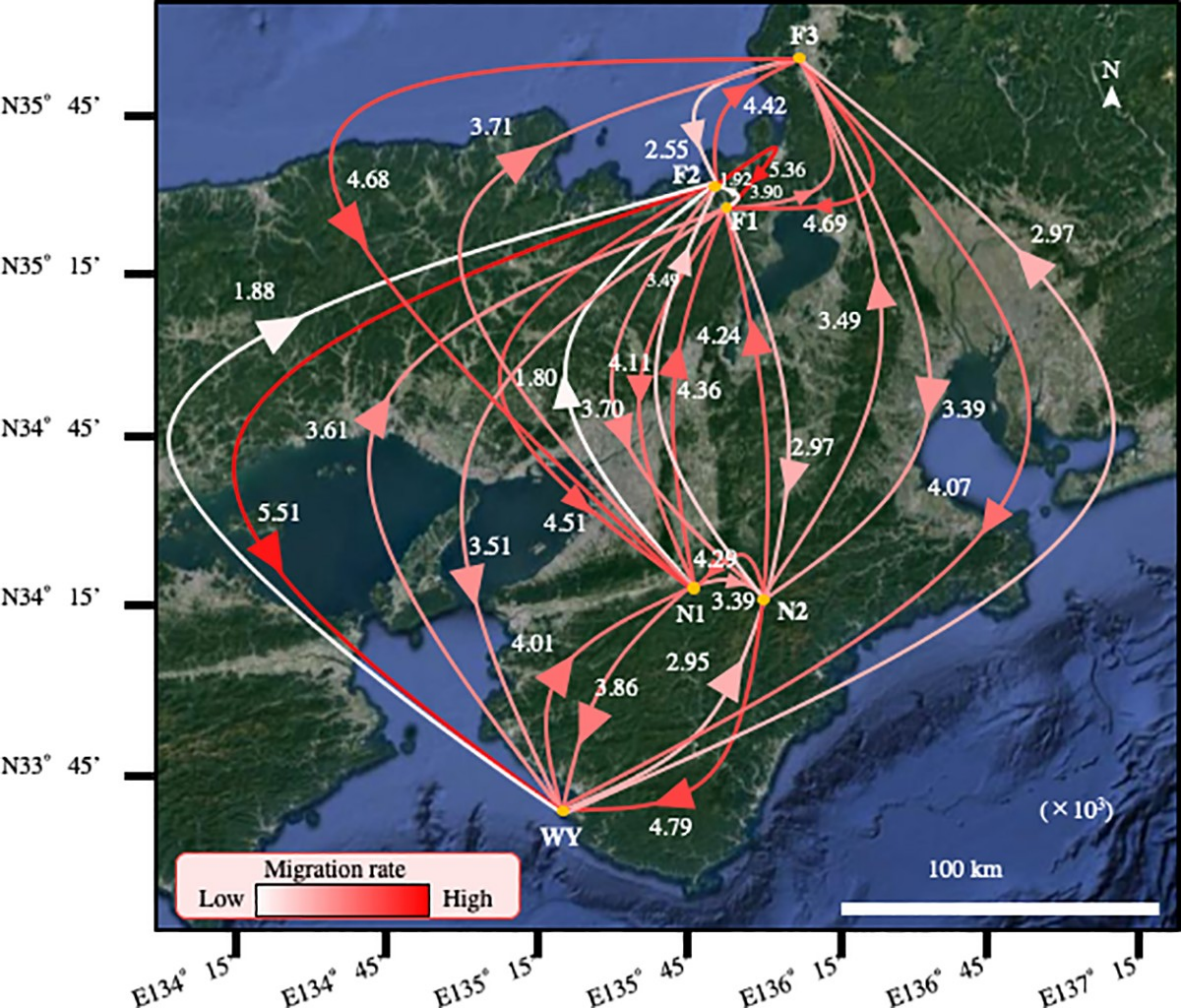
Gene flows among populations of *M*. *fuliginosus* bats in Japan (Wakayama, Nara, Fukui prefecture). The gene flows were estimated based on the D-loop amplicon sequencing results and subsequent analysis with Migrate-n software. The arrowheads on the lines show the directions of gene flow and the numbers near the arrowheads are the relative values of the gene flows (×10^3^).

The gene flows among six bat populations in Wakayama, Nara, and Fukui are shown in Fig 3. The gene flow to the bat population of Wakayama was mostly the largest of any population we sampled. In addition, gene flow to the population in Nara (N1) from other populations was relatively large.

The population of Fukui (F2) was larger than other populations (Table 1). Furthermore, the gene flow to the F2 population from other populations was remarkably small, especially from the N1 and Wakayama populations.

### Diversity of bat viruses

The fecal samples of eleven bat populations, which had been determined for the gene flow as described above, were then subjected to further analysis in terms of the viruses harbored. The results showed that seven bat populations (CH, JJ, WY, N2, F1, F2, and F3) contained bat alphaCoV, and five bat populations (K1, K2, JJ, N2, and F3) contained BtAdV. In total, 44 haplotypes of bat alphaCoV, and 35 haplotypes of BtAdV (Table 2). All viral sequences were then subjected to PACo analysis to observe genetic correlations between *M*. *fuliginosus* and the viruses.

### Genetic correlations between bats and viruses

**Fig 4:**
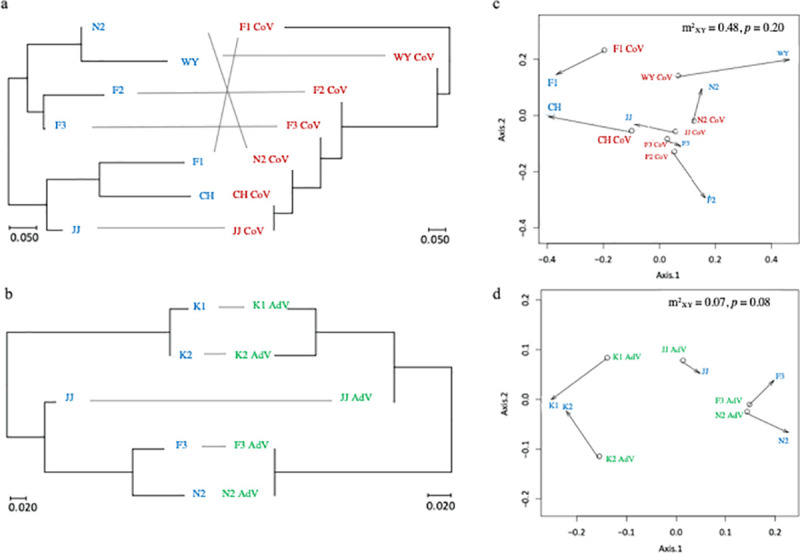
Congruence of phylogenetic patterns between *M*. *fuliginosus* and the related viruses. Comparison of population phylogenetic trees of A) *M*. *fuliginosus* and bat alphaCoV, and B) *M*. *fuliginosus* and BtAdV. Procrustean superimposition plots of C) *M*. *fuliginosus* and bat alphaCoV, and D) *M*. *fuliginosus* and BtAdV produced using PACo. D-loop of *M*. *fuliginosus* in each population are represented in blue color. Bat alphaCoV and BtAdV populations are represented in red color and green color, respectively.

Results of the PACo analysis (Fig 4) showed lack of the significant congruence in phylogenetic patterns between *M*. *fuliginosus* and BtAdV (m^2^_XY_ = 0.07, *p* = 0.08), and between *M*. *fuliginosus* and bat alphaCoV (m^2^_XY_ = 0.48, *p* = 0.20).

### Divergence times of bats and their viruses

**Fig 5:**
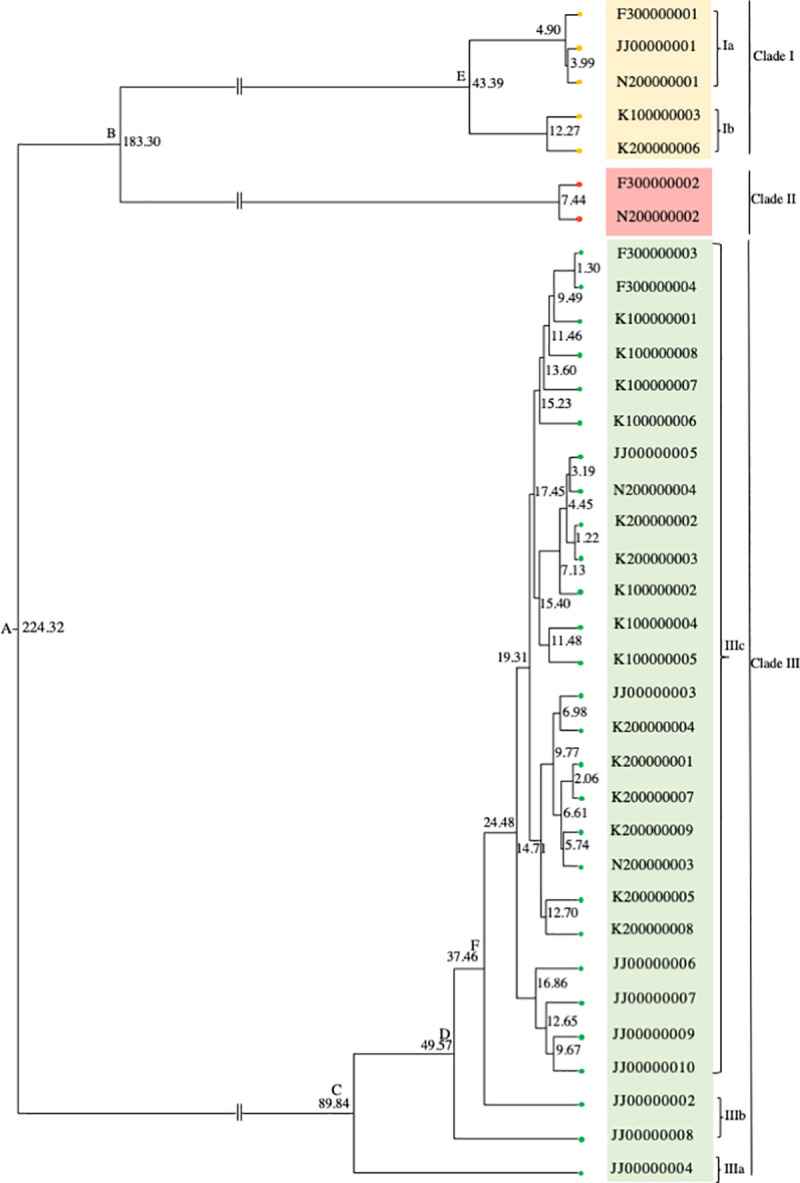
Divergence-time estimation of BtAdV based on the nucleotide-substitution rate per site per year of the *hexon* gene of HAdV-7. The time-scaled phylogenetic tree summarizes all MCMC phylogenies of the *hexon* gene data set, analyzed under HKY+G and Coalescent constant size models in BEAST software, version 2.6.0. The number at each node indicates the divergence time (years ago).

A Bayesian phylogenetic tree (Fig 5, S1 Fig) showed that the BtAdV sequences from fecal samples in Korea, Japan, and Jeju Island diverged into three major clades (clades I, II, and III). Clades I and III were derived from BtAdV sequences present in all positive caves. The clade I sequences diverged into two sub-clades; clade Ia consisted of sequences from Japan and Jeju island and clade Ib consisted of sequences from Korea. Clade III diverged into sub-clades IIIa, IIIb, and IIIc. Sub-clades IIIa and IIIb were both comprised of rare sequences found only in Jeju Island, whereas the sub-clade IIIc included sequences from all three regions (Korea, Japan, and Jeju Island). In contrast, clade II consisted of only the sequences found in Japan. The divergence times were estimated for the nodes between each of the clades and sub-clades (A, B, C, D, E, and F). Node A, which was the first divergence of the viruses among the five bat populations showed a divergence time of approximately 224.32 years ago. The time of the second divergence at node B was estimated to be 183.30 years ago. Nodes C, D, and F represented splits of rare BtAdV sequences from Jeju Island in the major sub-clade, IIIc, at which the divergence times were estimated to be approximately 89.84, 49.57, and 37.46 years ago, respectively.

**Fig 6:**
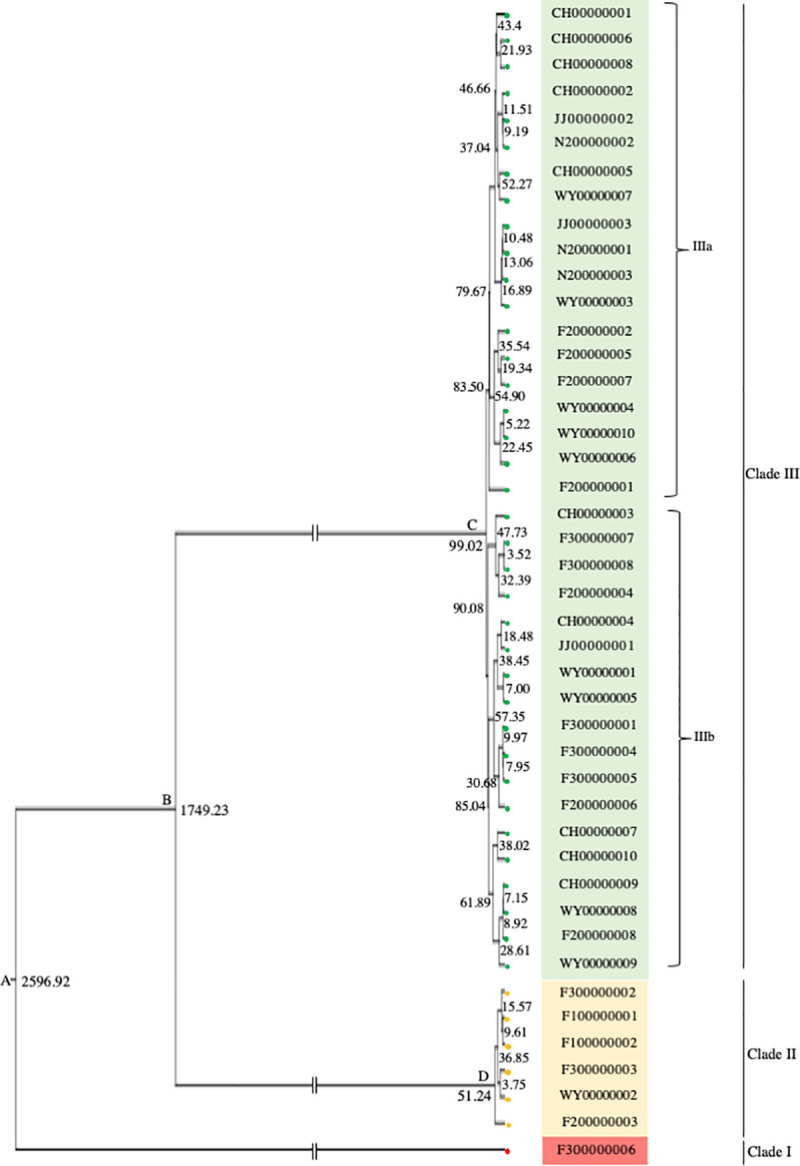
Divergence-time estimation of bat alphaCoV based on the nucleotide-substitution rate per site per year of the *RdRp* gene of coronavirus. The time-scaled phylogenetic tree summarizes all MCMC phylogenies of the *RdRp* gene data set, analyzed under HKY+G and Coalescent constant size models in BEAST software, version 2.6.0. The number at each node indicates the divergence time (years ago).

The Bayesian phylogenetic tree (Fig 6, S2 Fig) revealed that all bat alphaCoV sequences found in this study diverged into three major clades (I, II, and III). Only clade III was assigned into two sub-clades (IIIa and IIIb). Clades I and II consisted of sequences found in all positive caves in Japan, except for N2, whereas clade III was composed of sequences detected in all positive caves in Japan, Jeju Island, and China. The split times were estimated for all nodes among these clades, which were labeled as A, B, C, and D. Node A represented the first divergence time of bat alphaCoV, which was estimated to occur at 2,596.62 years ago. The second divergence of the virus was estimated to occur around 1,749.23 years ago at node B, which was between clades II and III, whereas node C resided between clades IIIa and IIIb, which represented a divergence time of around 99.02 years ago.

The divergence times based on the mitochondrial D-loop sequences of bats from 5 caves and 7 caves, which corresponded to positive dwellings for BtAdV and bat alphaCoV, respectively, are exhibited in S3 and S4 Figs. The Bayesian phylogenetic tree showed that the bats migrated among different geographic regions several times over time. The first divergence time of BtAdV-infected bats occur approximately 3.16 million years ago, whereas the first divergence time of alphaCoV-infected bats was estimated to occur 1.59 million years ago.

## Discussion

Satellite tracking systems have been used to determine migration of large frugivorous bats [28–30]. The systems cannot be applied to small bats because of heavy weight of telemetry machines [43]. A telemetry equipment cannot exceed more than 5% of bat body weight [44]. The weight of *M*. *fuliginosus* and *R*. *ferrumequinum* ranges from 10–14 g [45, 46] and 17–34 g [47], respectively. So, the telemetry equipment has to be less than 0.7 g for *M*. *fuliginosus*, and 1.7 g for *R*. *ferrumequinum*. To overcome these limitations, indirect population genetic methods including gene flow determination and genetic diversity analysis among populations have been used to evaluate movement patterns of animals [48–50].

### Bat fecal samples and mitochondrial D-loop DNA

In this study, the mitochondrial D-loop DNA sequences of *M*. *fuliginosus* in China, Korea, Taiwan, and Japan were examined, and the dynamic gene flows among these populations were revealed, using fecal guano samples taken from different caves. It has been reported that mitochondrial DNA degrades more slowly than nuclear DNA and that fecal mitochondrial DNA can still be sequenced, even at 60 days post-excretion [51, 52]. Generally, DNA in guano adheres to soil particles, and is more resistant to degradation than DNA alone [53, 54]. The study of Cai et al., 2006 [55] revealed that the existence of soil colloids and minerals was able to protect DNA to resist degradation by DNase I. Therefore, the presence of soil inside caves lengthens the shelf-life of mitochondrial DNA in feces; thus, the D-loop sequences obtained in this study could represent a large number of bats that lived in the caves for many years. Although coronavirus has shown stability in soil [56], and adenoviral DNA could be long preserved with soil [57], both of the viral genomic fragments have not been detected in TW and N1 cave by PCR. The reason remains unclear.

### The movement pattern of *M*. *fuliginosus* in east Asia

Previously, it was revealed that an ancestral population of the *M*. *fuliginosus* bat (before the Quaternary Glacier period) existed in southwest China, near the borders of Vietnam and Laos [19]. In this study, it was assumed that the bat population in the Henan Province (China) was most similar to the ancestral population, because this location was closest to the place where *M*. *fuliginosus* is thought to have originated.

Gene flow could be essential to determine movement patterns of animals. More gene flow means mixing of the population leading to increasing homogeneity of the population, and less gene flow means that populations are less mixed and genetically more differentiated. But in the case of bats, philopatry seems to play a very important role in the preservation of geographic genetic structure [58, 59]. If bats return to their natural colonies, rather than disperse to new or foreign colonies, then this reduces gene flow among populations and increases genetic differences especially among continental and island populations. Genetic similarity among all *M*. *fuliginosus* populations in our study were suggested by the results of Mantel test and AMOVA, which means frequent bat migrations among east Asian countries. Low *Φ*_*ST*_ values between the bat populations indicate a recent diversification of the three populations, CH, K1, and K2, which caused the low migration rates among the populations because Migrate-n estimated historical gene flows between populations.

The results shown in Fig 2 and Table 3 suggest that the genetic compositions of the Chinese and Korean bat populations were the most similar among all countries surveyed. Furthermore, our findings indicated that the bats in China, which were most similar to the ancestral population, might have moved to Korea and that such movement has been active between these populations.

After the genetic structure of these populations reached near equilibrium, the bats might have moved to Japan and Taiwan relatively recently. For example, previous results revealed that the bats in Japan, Korea, and China shared the same D-loop sequences [25, 26]. Assuming that the mutation rate of the D-loop DNA sequence is approximately one base every 20,000 years [60], we inferred that the bats in Japan arrived from Korea or from China during the last 20,000 years.

The geographical distance between the Korean Peninsula and Jeju Island (K1-JJ: 379 km, K2-JJ: 213 km) is smaller than that between China and Jeju Island (CH-JJ: 1,429 km), between Taiwan and Jeju Island (TW-JJ: 1,043 km), and between Japan and Jeju Island (WY-JJ: 788 km). However, it is possible that when *M*. *fuliginosus* bats migrated to Jeju Island from China and the Korean Peninsula, limited food resources reduced the population size, resulting in a bottleneck effect on Jeju Island. This possibility could explain the different genetic compositions of the population in Jeju Island versus those in China and on the Korean Peninsula.

The migration rate estimated using Migrate-n software does not reflect the current gene flow, but rather, the gene flow that occurred during the evolutionary history of the local bat populations [61]. Regarding the gene flow between China, Korea, and Japan, and the *Φ*_*ST*_ genetic distance between China and Japan, our findings suggest that the Japanese bat population might have been maintained as an independent population for a very long time after the bats migrated to Japan from China and Korea. We assumed that after the *M*. *fuliginosus* bats migrated from China to the Korean Peninsula and Japan, they later migrated to Jeju Island and/or Taiwan. Such migrations could have been caused by climate change, particularly by declining sea levels during the glacial period and the subsequent warming period that occurred 20,000 years ago [62].

### The movement pattern of *M*. *fuliginosus* in Japan

It was previously reported that approximately 15,000 pregnant female bats migrated to the nursery roost in Wakayama from mid-June to mid-August to give birth and raise their offspring collaboratively. When the offspring reached two months of age, the colony of female bats and offspring flew away from the nursery roost. These bats were then found in winter roosts, which are located 10–200 km away from the nursery roost [24]. Additionally, it is possible that N1 serves as a relay point connecting the nursery roost in Wakayama to winter roosts in various other regions. The winter roosts in Fukui and Shiga are located more than 200 km away from Wakayama. Thus, the N1 roost may serve as a stopover site, since the bats might have difficulty completing such a journey in a single night.

Based on the population size of the bats in the F2 cave (Table 1), we assumed that the F2 population might be formed earlier and was comprised of sedentary bats that did not use the Wakayama cave as a nursery one. It is also possible that the bats of the F2 population fly to the nursery roost in Wakayama without stopping at the site of the N1 population, or that they go to another nursery roost to give birth and raise their offspring. The largest *Φ*_*ST*_ genetic distance of bat WY population to the other populations (Table 4) and large gene flows from WY to the other populations (Fig 3) supported that WY cave has been used for a long time as a nursery roost for bats. The low gene flows of bats into F2 from other populations (Fig 3), and low *Φ*_*ST*_ between F2 and F3 of bat populations (Table 4) also supported the recent establishment of bat F2 populations by migrants from F3 population.

**Table 4:** *Φ*_*ST*_ genetic distance between *M*. *fuliginosus* populations in Japan.

	N1	N2	WY	F1	F2	F3
N1						
N2	0.16					
WY	0.01	0.19				
F1	0.71	0.41	0.73			
F2	0.43	0.23	0.44	0.56		
F3	0.36	0.11	0.37	0.40	0.12	

All values show significance at *P* ≤ 0.05.

### Genetic correlations between viruses and their hosts

Originally, we expected that if bats carry viruses, significant genetic co-variation between the viruses and *M*. *fuliginosus* populations could be detected. However, no significant co-variation was observed between the phylogenies of bats and viruses. Although the population genetic structure of bats revealed in this study was historically constructed by bat individuals which produced descendants successfully, there must be some bats which moved between the caves, but did not succeed in mating. We supposed that the individual contact between the *M*. *fuliginosus* bats, which did not result in successful mating, might also increase transmission opportunities of viruses that were not reflected in the host genetic pattern.

### The divergence time of bats and viruses

The Bayesian phylogenetic tree of BtAdV (Fig 5) did not indicate the geographical origin of BtAdV observed in our study because all clades contained Japanese and Korean viral strains. Lineage diversification of BtAdV was inferred to recently occur within the last 50 years in all five bat populations. In contrast, the Bayesian phylogenetic tree of bat alphaCoV (Fig 6) revealed three major clades, each of which shared the virus sequences detected in Fukui, Japan (F3). We propose that the virus of the F3 population may have been the common ancestor of all bat alphaCoV lineages that presented approximately 2,596.92 years ago. According to this theory, the virus was then distributed to seven bat populations around 1,749.23 years ago. The third and fourth large divergences of bat alphaCoV occurred in the last 100 years. Our result was consistent with the previous report, in which all alphaCoV lineage was estimated to occur approximately 4,381 years ago based on the *RdRp* gene [41]. Whilst, the recent divergence time of BtAdV (50 years ago) corresponds to the report of Lin et al., 2015 [42], in which the most recent common ancestor of HAdV-7 was dated approximately 71 years ago based on the *hexon* gene. Even though a short fragment of viral sequences (less than 300 bps in length) were analyzed in this study, the regions possessed a large number of variable sites. Thus, we decided the sequences were informative enough to resolve phylogenetic relationships and to estimate divergence times among viral populations examined in this study.

Based on the findings of this study, it seems that other animals and/or other bat species may be involved in the divergences of BtAdV and bat alphaCoV in recent years. Viral transmission could be happened within and among bat colonies by direct and/or indirect contact. Not only does viral transmission observed among bats, but occasionally transmission to other animals inhabited in the same foraging area can be exposed to their infectious bodily fluids [63]. Hematophagous arthropods which are in close contact with bats could serve as pathogen transmitters among bats and humans [64]. It is suggested that houseflies *(Musca domestica Linnaceaus)* are able to fly up to 20 km, and could be the vectors of porcine alphaCoV [65]. Also, mammalian adenoviral DNA has been detected in blow flies *(Calliphoridae)* and flesh flies *(Sarcophagidae)* [66]. Even though there is no scientific evidence that bat alphaCoV and BtAdV are transmitted by the flies, it is possible that the flies could be act as the vector in viral dispersion.

Additionally, the pathogen transmission among different bat species which is co-roosting in the same cave should be considered. Host-switching event of alphaCoV has been found in the cave shared by *Miniopterus* bats and *Rhinolophus* bats [67]. There is a report that *R*. *ferrumequinum* bats have been found in China, Korea, and Japan, and exhibited close phylogenetic relationship in their mitochondrial *cytochrome b* DNA [68]. Therefore, the species have moved over the three countries [68]. Moreover, *R*. *ferrumequinum* inhabit in China have been reported to be the host of alphaCoV [18]. The species often share their roosting sites with *M*. *fuliginosus* [20, 21]. Thus, it is considered that the viruses found in our study might be shared between *M*. *fuliginosus* and *R*. *ferrumequinum*. It provides opportunities for cross-species transmission of viruses among different bat species.

## Supporting information

S1 Fig Divergence time estimation of BtAdV based on the nucleotide-substitution rate per site per year of the *hexon* gene of HAdV-7. The time-scaled phylogenetic tree summarizes all MCMC phylogenic analyses of the *hexon* gene data set, analyzed under HKY+G and Coalescent constant size models in BEAST software, version 2.6.0. The number at each node indicates the posterior probability and confidence interval.
(TIF)

S2 Fig Divergence time estimation of bat alphaCoV based on the nucleotide-substitution rate per site per year of the *RdRp* gene of coronavirus. The time-scaled phylogenetic tree summarizes all MCMC phylogenies of the *RdRp* gene data set, analyzed under HKY+G and Coalescent constant size models in BEAST software, version 2.6.0. The number at each node indicates the posterior probability and confidence interval.
(TIF)

S3 Fig Divergence time estimation of *M*. *fuliginosus*, which harbored BtAdV, based on mitochondrial D-loop region. The nucleotide substitution rate was 20% per site per million years. The time-scaled phylogeny was summarized from all MCMC phylogenies of the D-loop DNA data set analyzed under HKY+G and Coalescent constant size models in BEAST version 2.6.0. The number indicates the divergence time (million years ago).
(PDF)

S4 Fig Divergence time estimation of *M*. *fuliginosus*, which harbored bat alphaCoV, based on mitochondrial D-loop region. The nucleotide substitution rate was 20% per site per million years. The time-scaled phylogeny was summarized from all MCMC phylogenies of the D-loop DNA data set analyzed under HKY+G and Coalescent constant size models in BEAST version 2.6.0. The number indicates the earliest divergence time (million years ago).
(PDF)

S1 Table Parameters used for migrate-n analysis. (XLSX)

S2 Table Analysis of molecular variance (AMOVA) among eleven populations of *M*. *fuliginosus*. (DOCX)

S3 Table Mantel test analysis among eleven population of *M*. *fuliginosus*. (DOCX)
