# Supplementary figures and images for: Dispersal history of Miniopterus fuliginosus bats and their associated viruses in east Asia
Source: PLoS One. 2021 Jan 14;16(1):e0244006. doi: 10.1371/journal.pone.0244006 (PMC7808576; doi:10.1371/journal.pone.0244006)

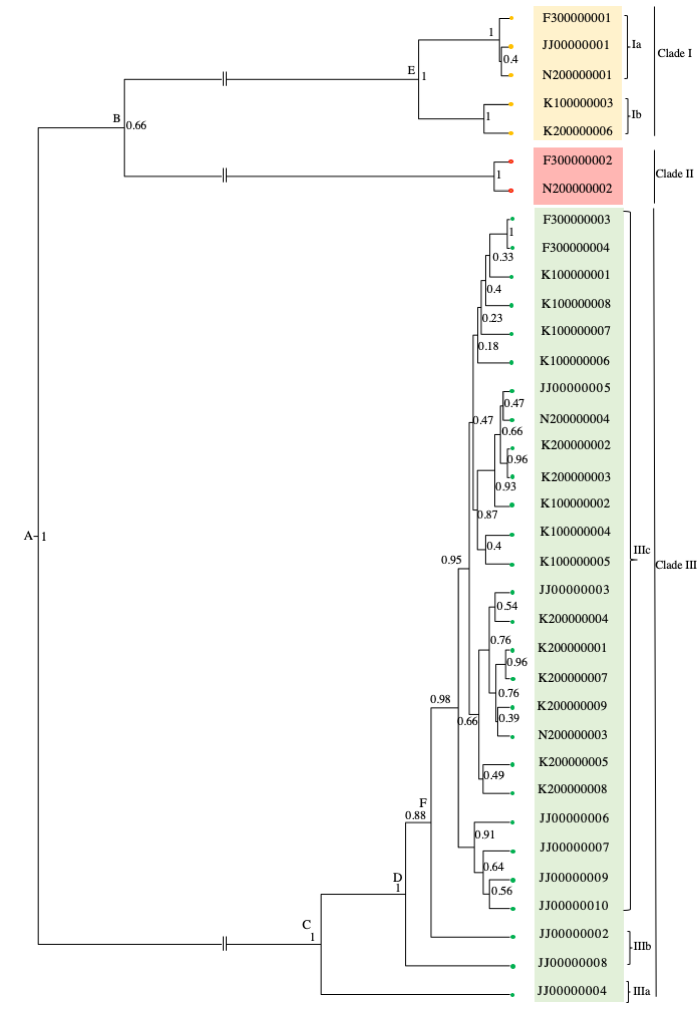

Supplement: S1 Fig — The time-scaled phylogenetic tree summarizes all MCMC phylogenic analyses of the hexon gene data set, analyzed under HKY+G and Coalescent constant size models in BEAST software, version 2.6.0. The number at each node indicates the posterior probability and confidence interval. (TIF) [file pone.0244006.s001.tif]

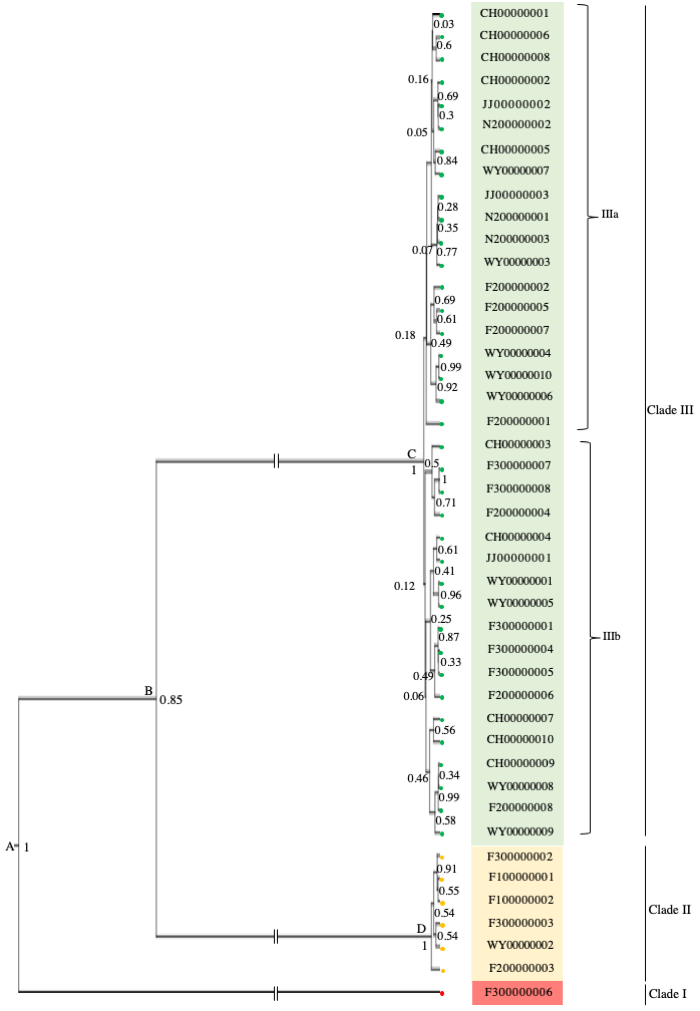

Supplement: S2 Fig — The time-scaled phylogenetic tree summarizes all MCMC phylogenies of the RdRp gene data set, analyzed under HKY+G and Coalescent constant size models in BEAST software, version 2.6.0. The number at each node indicates the posterior probability and confidence interval. (TIF) [file pone.0244006.s002.tif]
