# Supplementary material for: Dispersal history of Miniopterus fuliginosus bats and their associated viruses in east Asia
Source: PLoS One. 2021 Jan 14;16(1):e0244006. doi: 10.1371/journal.pone.0244006 (PMC7808576; doi:10.1371/journal.pone.0244006)
Supplement: S2 Table — (DOCX) [file pone.0244006.s006.docx]

**S2 Table.** Analysis of molecular variance (AMOVA) among the eleven populations of *M. fuliginosus*

|  |  | *M. fuliginosus* population |
| --- | --- | --- |
| Groups and populations | Group 1 | CH |
|  | Group 2 | K1, K2 |
|  | Group 3 | JJ |
|  | Group 4 | TW |
|  | Group 5 | WY, N1, N2, F1, F2, F3 |
| Variance components (Proportion of each variation accounting for total genetic variation) | Among groups | 1.478 (19.50%) |
|  | Among population within groups | 2.086 (27.52%) |
|  | Within populations | 4.015 (52.97%) |
