# Supplementary material for: Dispersal history of Miniopterus fuliginosus bats and their associated viruses in east Asia
Source: PLoS One. 2021 Jan 14;16(1):e0244006. doi: 10.1371/journal.pone.0244006 (PMC7808576; doi:10.1371/journal.pone.0244006)
Supplement: S3 Table — (DOCX) [file pone.0244006.s007.docx]

**S3 Table.** Mantel test analysis among eleven populations of *M. fuliginosus*

|  | Coeffecient value of *M. fuliginosus* populations |
| --- | --- |
| Correlation coefficient | 0.161 |
| Coefficient of determination (R^2^) | 0.026 |
| *P-value* | >0.05 |
