# Supplementary material for: Dispersal history of Miniopterus fuliginosus bats and their associated viruses in east Asia
Source: PLoS One. 2021 Jan 14;16(1):e0244006. doi: 10.1371/journal.pone.0244006 (PMC7808576; doi:10.1371/journal.pone.0244006)
Supplement: S3 Fig — The nucleotide substitution rate was 20% per site per million years. The time-scaled phylogeny was summarized from all MCMC phylogenies of the D-loop DNA data set analyzed under HKY+G and Coalescent constant size models in BEAST version 2.6.0. The number indicates the divergence time (million years ago). (PDF) [file pone.0244006.s003.pdf]

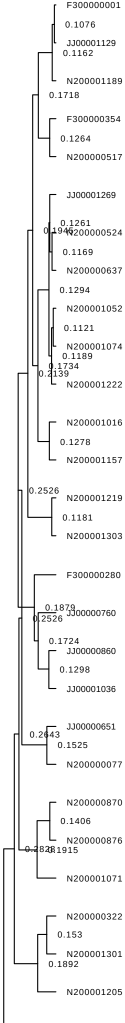
F300000001
0.1076
JJ00001129
0.1162
N200001189
0.1718
F300000354
0.1264
N200000517
JJ00001269
0.1261
0.1945
N200000524
0.1169
N200000637
0.1294
N200001052
0.1121
N200001074
0.1189
0.1734
0.2139
N200001222
N200001016
0.1278
N200001157
0.2526
N200001219
0.1181
N200001303
F300000280
0.1879
JJ00000760
0.2526
0.1724
JJ00000860
0.1298
JJ00001036
JJ00000651
0.2643
0.1525
N200000077
N200000870
0.1406
N200000876
0.2828
0.1915
N200001071
N200000322
0.153
N200001301
0.1892
N200001205

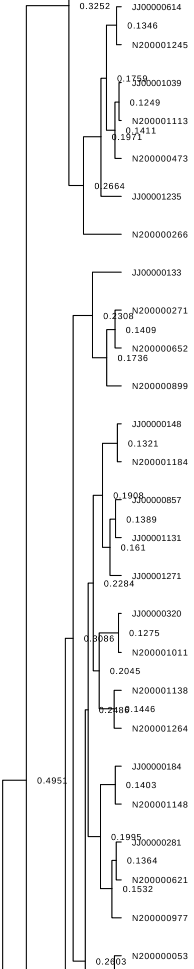

0.3252
JJ00000614
0.1346
N200001245
0.1759
JJ00001039
0.1249
N200001113
0.1411
0.1971
N200000473
0.2664
JJ00001235
N200000266
JJ00000133
N200000271
0.2308
0.1409
N200000652
0.1736
N200000899
JJ00000148
0.1321
N200001184
0.1908
JJ00000857
0.1389
JJ00001131
0.161
JJ00001271
0.2284
JJ00000320
0.1275
0.3086
N200001011
0.2045
N200001138
0.2486
0.1446
N200001264
JJ00000184
0.4951
0.1403
N200001148
0.1995
JJ00000281
0.1364
N200000621
0.1532
N200000977
N200000053
0.2603

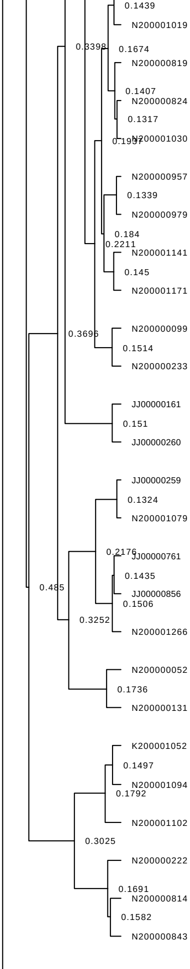
0.1439
N200001019
0.3398
0.1674
N200000819
0.1407
N200000824
0.1317
N200001030
0.1937
N200000957
0.1339
N200000979
0.184
0.2211
N200001141
0.145
N200001171
N200000099
0.3696
0.1514
N200000233
JJ00000161
0.151
JJ00000260
JJ00000259
0.1324
N200001079
0.2176
JJ00000761
0.1435
JJ00000856
0.485
0.1506
0.3252
N200001266
N200000052
0.1736
N200000131
K200001052
0.1497
N200001094
0.1792
N200001102
0.3025
N200000222
0.1691
N200000814
0.1582
N200000843

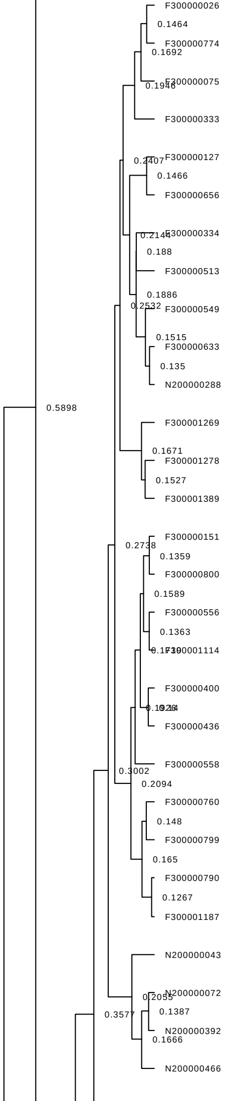
F300000026
0.1464
F300000774
0.1692
F300000075
0.1946
F300000333
F300000127
0.2407
0.1466
F300000656
F300000334
0.2144
0.188
F300000513
0.1886
0.2532
F300000549
0.1515
F300000633
0.135
N200000288
0.5898
F300001269
0.1671
F300001278
0.1527
F300001389
F300000151
0.2738
0.1359
F300000800
0.1589
F300000556
0.1363
F300001114
F300000400
F300000436
F300000558
0.3002
0.2094
F300000760
0.148
F300000799
0.165
F300000790
0.1267
F300001187
N200000043
N200000072
0.2055
0.1387
N200000392
0.3577
0.1666
N200000466

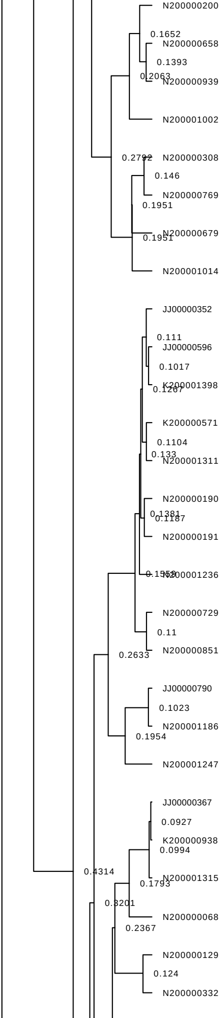

N200000200
0.1652
N200000658
0.1393
0.2063
N200000939
N200001002
0.2792
N200000308
0.146
N200000769
0.1951
N200000679
0.1951
N200001014
JJ00000352
0.111
JJ00000596
0.1017
K200001398
0.1267
K200000571
0.1104
0.133
N200001311
N200000190
0.1381
0.1187
N200000191
0.1559
N200001236
N200000729
0.11
N200000851
0.2633
JJ00000790
0.1023
N200001186
0.1954
N200001247
JJ00000367
0.0927
K200000938
0.0994
0.4314
N200001315
0.1793
0.3201
N200000068
0.2367
N200000129
0.124
N200000332

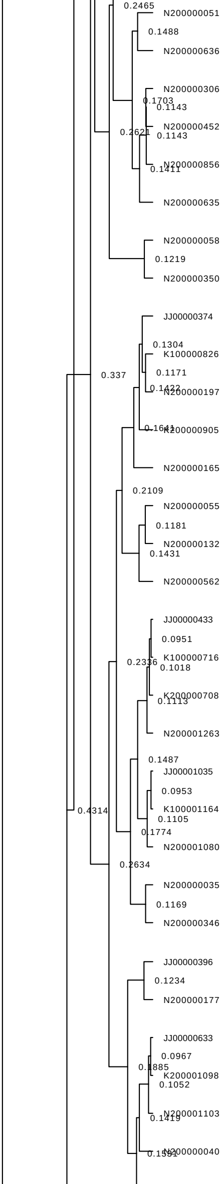
0.2465
N200000051
0.1488
N200000636
N200000306
0.1703
0.1143
N200000452
0.2621
0.1143
N200000856
0.1411
N200000635
N200000058
0.1219
N200000350
JJ00000374
0.1304
K100000826
0.337
0.1171
0.1422
N200000197
0.1641
K200000905
N200000165
0.2109
N200000055
0.1181
N200000132
0.1431
N200000562
JJ00000433
0.0951
K100000716
0.2336
0.1018
K200000708
0.1113
N200001263
0.1487
JJ00001035
0.0953
K100001164
0.4314
0.1105
0.1774
N200001080
0.2634
N200000035
0.1169
N200000346
JJ00000396
0.1234
N200000177
JJ00000633
0.0967
0.1885
K200001098
0.1052
N200001103
0.1419
N200000040
0.1531

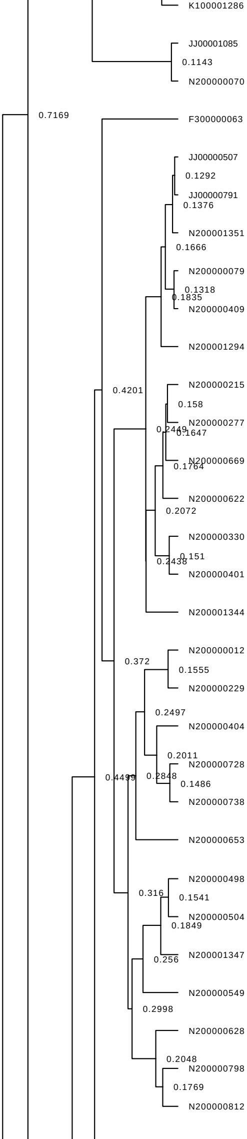
K100001286
JJ00001085
0.1143
N200000070
0.7169
F300000063
JJ00000507
0.1292
JJ00000791
0.1376
N200001351
0.1666
N200000079
0.1318
0.1835
N200000409
N200001294
N200000215
0.4201
0.158
N200000277
0.2449
0.1647
N200000669
0.1764
N200000622
0.2072
N200000330
0.151
0.2438
N200000401
N200001344
N200000012
0.372
0.1555
N200000229
0.2497
N200000404
0.2011
N200000728
0.2848
0.4499
0.1486
N200000738
N200000653
N200000498
0.316
0.1541
N200000504
0.1849
N200001347
0.256
N200000549
0.2998
N200000628
0.2048
N200000798
0.1769
N200000812

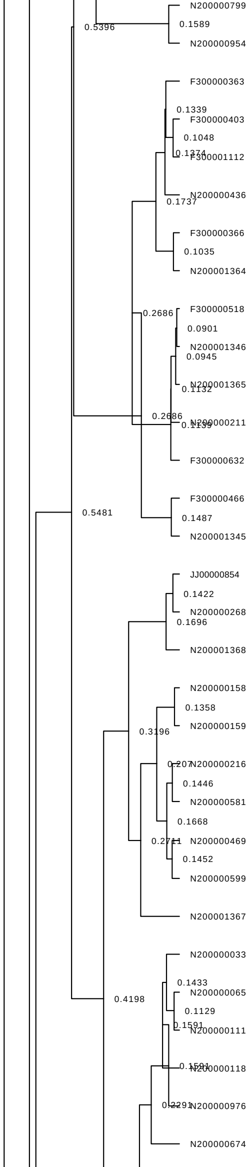

N200000799
0.1589
0.5396
N200000954
F300000363
0.1339
F300000403
0.1048
0.1374
F300001112
N200000436
0.1737
F300000366
0.1035
N200001364
F300000518
0.2686
0.0901
N200001346
0.0945
N200001365
0.1132
0.2686
N200000211
0.1139
F300000632
F300000466
0.5481
0.1487
N200001345
JJ00000854
0.1422
N200000268
0.1696
N200001368
N200000158
0.1358
N200000159
0.3196
0.207
N200000216
0.1446
N200000581
0.1668
0.2711
N200000469
0.1452
N200000599
N200001367
N200000033
0.1433
N200000065
0.4198
0.1129
0.1591
N200000111
0.1591
N200000118
0.2291
N200000976
N200000674

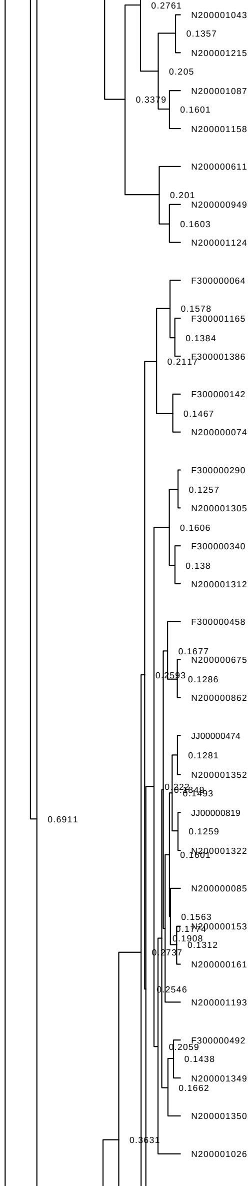
0.2761
N200001043
0.1357
N200001215
0.205
N200001087
0.3379
0.1601
N200001158
N200000611
0.201
N200000949
0.1603
N200001124
F300000064
0.1578
F300001165
0.1384
F300001386
0.2117
F300000142
0.1467
N200000074
F300000290
0.1257
N200001305
0.1606
F300000340
0.138
N200001312
F300000458
0.1677
N200000675
0.2593
0.1286
N200000862
JJ00000474
0.1281
N200001352
0.222
0.1493
0.6911
JJ00000819
0.1259
N200001322
0.1601
N200000085
0.1563
N200000153
0.1774
0.1908
0.1312
0.2737
N200000161
0.2546
N200001193
F300000492
0.2059
0.1438
N200001349
0.1662
N200001350
0.3631
N200001026

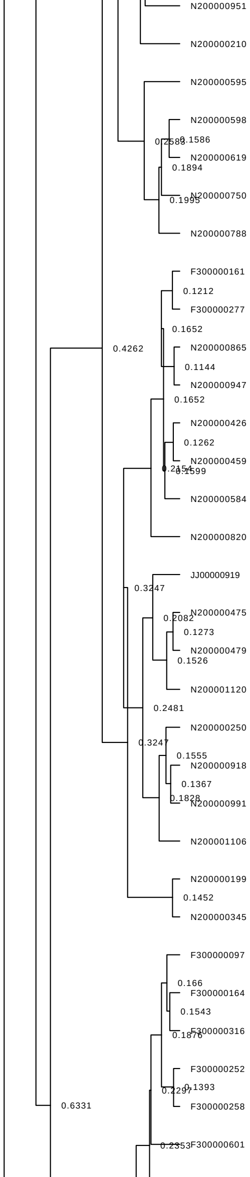
N200000951
N200000210
N200000595
N200000598
0.2583
0.1586
N200000619
0.1894
N200000750
0.1995
N200000788
F300000161
0.1212
F300000277
0.1652
N200000865
0.4262
0.1144
N200000947
0.1652
N200000426
0.1262
N200000459
0.2154
0.1599
N200000584
N200000820
JJ00000919
0.3247
N200000475
0.2082
0.1273
N200000479
0.1526
N200001120
0.2481
N200000250
0.3247
0.1555
N200000918
0.1367
0.1828
N200000991
N200001106
N200000199
0.1452
N200000345
F300000097
0.166
F300000164
0.1543
F300000316
0.1876
F300000252
0.1393
0.2297
F300000258
0.6331
0.2353
F300000601

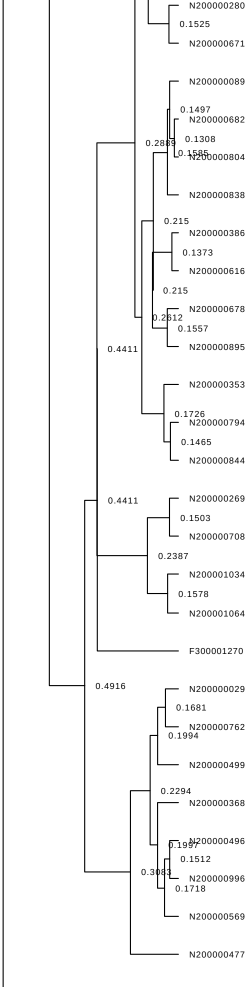
N200000280
0.1525
N200000671
N200000089
0.1497
N200000682
0.1308
0.2889
0.1585
N200000804
N200000838
0.215
N200000386
0.1373
N200000616
0.215
N200000678
0.2612
0.1557
N200000895
0.4411
N200000353
0.1726
N200000794
0.1465
N200000844
0.4411
N200000269
0.1503
N200000708
0.2387
N200001034
0.1578
N200001064
F300001270
0.4916
N200000029
0.1681
N200000762
0.1994
N200000499
0.2294
N200000368
N200000496
0.1997
0.1512
0.3083
N200000996
0.1718
N200000569
N200000477

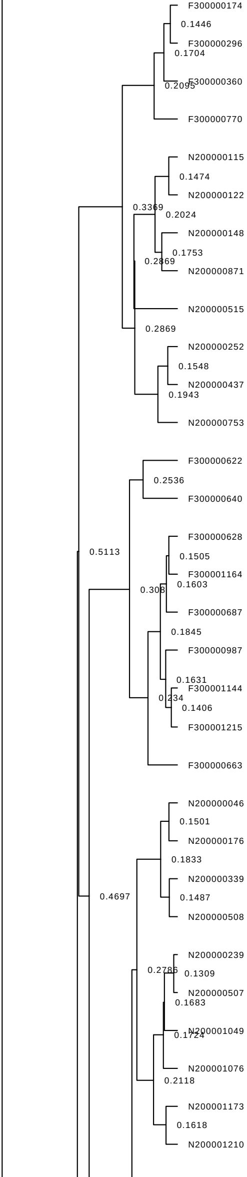
F300000174
0.1446
F300000296
0.1704
F300000360
0.2095
F300000770
N200000115
0.1474
N200000122
0.3369
0.2024
N200000148
0.1753
0.2869
N200000871
N200000515
0.2869
N200000252
0.1548
N200000437
0.1943
N200000753
F300000622
0.2536
F300000640
F300000628
0.5113
0.1505
F300001164
0.1603
0.308
F300000687
0.1845
F300000987
0.1631
F300001144
0.234
0.1406
F300001215
F300000663
N200000046
0.1501
N200000176
0.1833
N200000339
0.4697
0.1487
N200000508
N200000239
0.2786
0.1309
N200000507
0.1683
N200001049
0.1724
N200001076
0.2118
N200001173
0.1618
N200001210

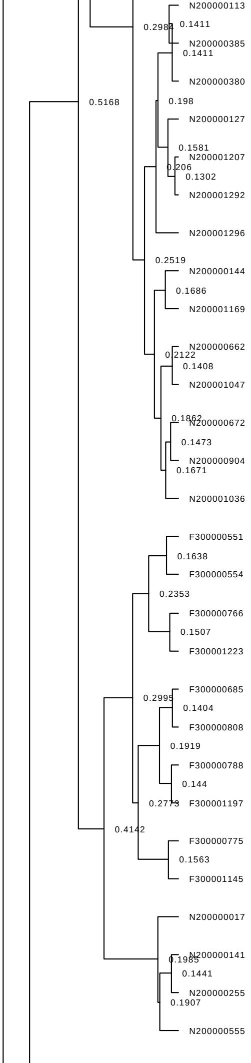

N200000113
0.2984
0.1411
N200000385
0.1411
N200000380
0.5168
0.198
N200000127
0.1581
N200001207
0.206
0.1302
N200001292
N200001296
0.2519
N200000144
0.1686
N200001169
N200000662
0.2122
0.1408
N200001047
0.1862
N200000672
0.1473
N200000904
0.1671
N200001036
F300000551
0.1638
F300000554
0.2353
F300000766
0.1507
F300001223
F300000685
0.2995
0.1404
F300000808
0.1919
F300000788
0.144
0.2773
F300001197
0.4142
F300000775
0.1563
F300001145
N200000017
N200000141
0.1985
0.1441
N200000255
0.1907
N200000555

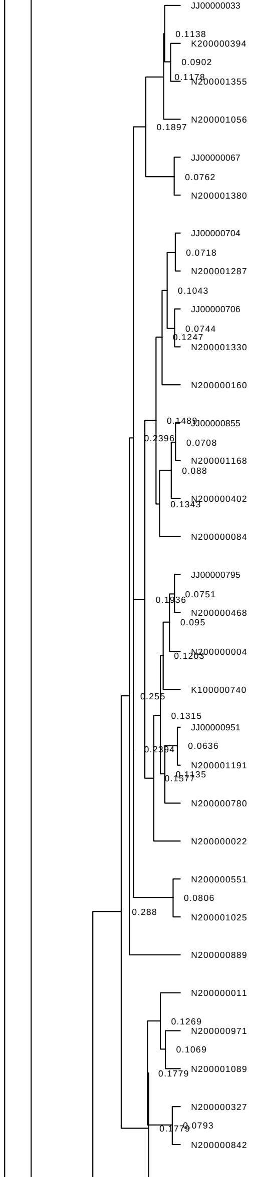
JJ00000033
0.1138
K200000394
0.0902
0.1178
N200001355
N200001056
0.1897
JJ00000067
0.0762
N200001380
JJ00000704
0.0718
N200001287
0.1043
JJ00000706
0.0744
0.1247
N200001330
N200000160
0.1489
JJ00000855
0.2396
0.0708
N200001168
0.088
N200000402
0.1343
N200000084
JJ00000795
0.0751
0.1936
N200000468
0.095
N200000004
0.1203
K100000740
0.255
0.1315
JJ00000951
0.0636
0.2394
N200001191
0.1135
0.1577
N200000780
N200000022
N200000551
0.0806
0.288
N200001025
N200000889
N200000011
0.1269
N200000971
0.1069
N200001089
0.1779
N200000327
0.1779
0.0793
N200000842

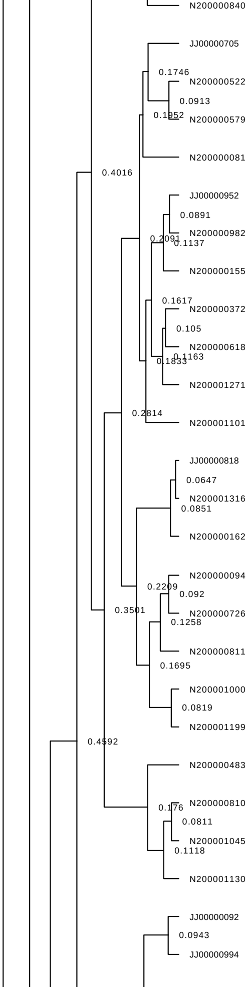
N200000840
JJ00000705
0.1746
N200000522
0.0913
0.1952
N200000579
N200000081
0.4016
JJ00000952
0.0891
N200000982
0.2091
0.1137
N200000155
0.1617
N200000372
0.105
N200000618
0.1163
0.1833
N200001271
0.2814
N200001101
JJ00000818
0.0647
N200001316
0.0851
N200000162
N200000094
0.2209
0.092
N200000726
0.1258
0.3501
N200000811
0.1695
N200001000
0.0819
N200001199
0.4592
N200000483
0.176
N200000810
0.0811
N200001045
0.1118
N200001130
JJ00000092
0.0943
JJ00000994

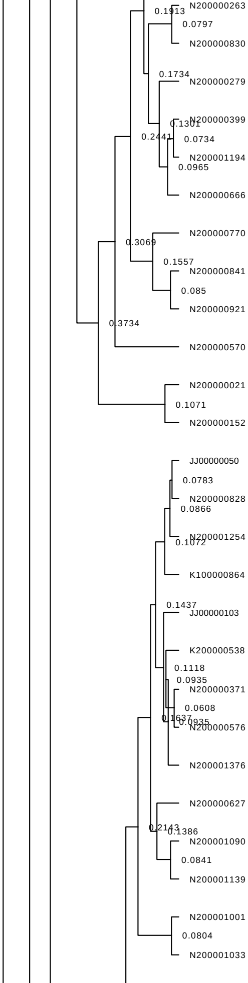

N200000263
0.1913
0.0797
N200000830
0.1734
N200000279
N200000399
0.1301
0.2441
0.0734
N200001194
0.0965
N200000666
N200000770
0.3069
0.1557
N200000841
0.085
N200000921
0.3734
N200000570
N200000021
0.1071
N200000152
JJ00000050
0.0783
N200000828
0.0866
N200001254
0.1072
K100000864
0.1437
JJ00000103
K200000538
0.1118
0.0935
N200000371
0.0608
0.1637
0.0935
N200000576
N200001376
N200000627
0.2143
0.1386
N200001090
0.0841
N200001139
N200001001
0.0804
N200001033

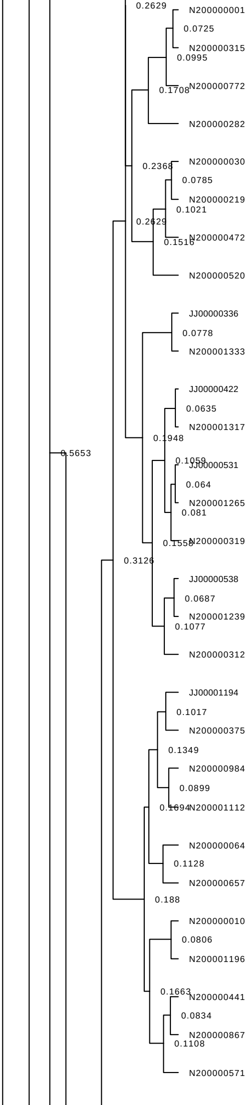
0.2629
N200000001
0.0725
N200000315
0.0995
N200000772
0.1708
N200000282
0.2368
N200000030
0.0785
N200000219
0.1021
0.2629
N200000472
0.1516
N200000520
JJ00000336
0.0778
N200001333
JJ00000422
0.0635
N200001317
0.1948
0.5653
0.1059
JJ00000531
0.064
N200001265
0.081
N200000319
0.1558
0.3126
JJ00000538
0.0687
N200001239
0.1077
N200000312
JJ00001194
0.1017
N200000375
0.1349
N200000984
0.0899
0.1694
N200001112
N200000064
0.1128
N200000657
0.188
N200000010
0.0806
N200001196
0.1663
N200000441
0.0834
N200000867
0.1108
N200000571

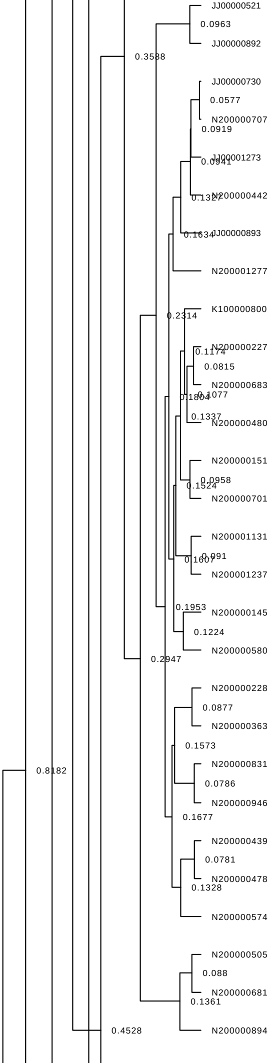
JJ00000521
0.0963
JJ00000892
0.3588
JJ00000730
0.0577
N200000707
0.0919
JJ00001273
0.0941
N200000442
0.1327
JJ00000893
0.1634
N200001277
K100000800
0.2314
N200000227
0.1174
0.0815
N200000683
0.1077
0.1804
0.1337
N200000480
N200000151
0.0958
0.1524
N200000701
N200001131
0.091
0.1607
N200001237
0.1953
N200000145
0.1224
N200000580
0.2947
N200000228
0.0877
N200000363
0.1573
N200000831
0.0786
N200000946
0.1677
N200000439
0.0781
N200000478
0.1328
N200000574
N200000505
0.088
N200000681
0.1361
N200000894
0.4528
0.8182

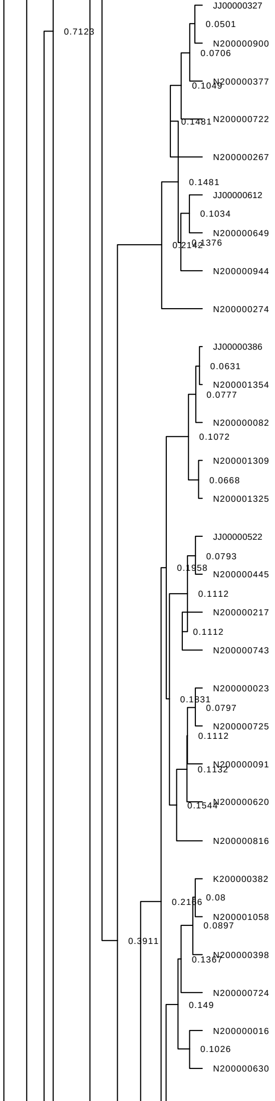

JJ00000327
0.0501
N200000900
0.0706
N200000377
0.1049
N200000722
0.1481
N200000267
0.1481
JJ00000612
0.1034
N200000649
0.2142
0.1376
N200000944
N200000274
0.7123
JJ00000386
0.0631
N200001354
0.0777
N200000082
0.1072
N200001309
0.0668
N200001325
JJ00000522
0.0793
0.1958
N200000445
0.1112
N200000217
0.1112
N200000743
N200000023
0.1331
0.0797
N200000725
0.1112
N200000091
0.1132
N200000620
0.1544
N200000816
K200000382
0.08
0.2156
N200001058
0.0897
0.3911
N200000398
0.1367
N200000724
0.149
N200000016
0.1026
N200000630

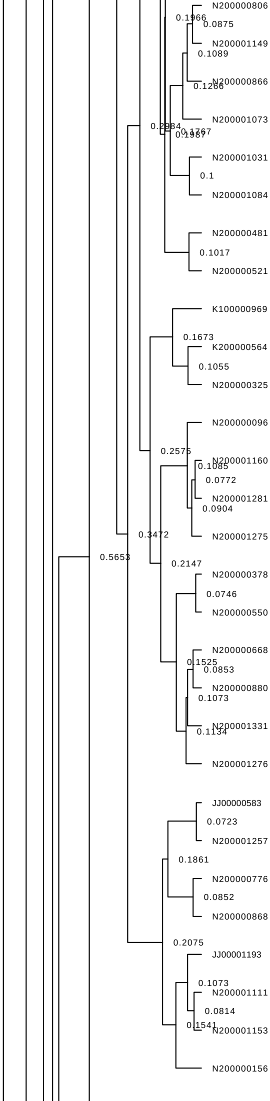
N200000806
0.1966
0.0875
N200001149
0.1089
N200000866
0.1266
N200001073
0.2984
0.1767
0.1987
N200001031
0.1
N200001084
N200000481
0.1017
N200000521
K100000969
0.1673
K200000564
0.1055
N200000325
N200000096
0.2575
N200001160
0.1085
0.0772
N200001281
0.0904
0.3472
N200001275
0.5653
0.2147
N200000378
0.0746
N200000550
N200000668
0.1525
0.0853
N200000880
0.1073
N200001331
0.1134
N200001276
JJ00000583
0.0723
N200001257
0.1861
N200000776
0.0852
N200000868
0.2075
JJ00001193
0.1073
N200001111
0.0814
0.1541
N200001153
N200000156

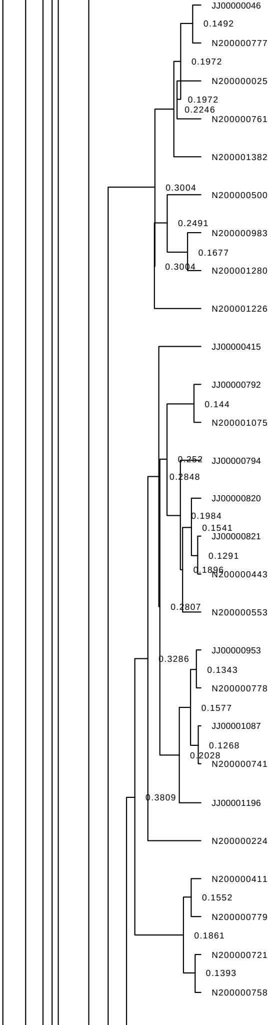
JJ00000046
0.1492
N200000777
0.1972
N200000025
0.1972
0.2246
N200000761
N200001382
0.3004
N200000500
0.2491
N200000983
0.1677
0.3004
N200001280
N200001226
JJ00000415
JJ00000792
0.144
N200001075
0.252
JJ00000794
0.2848
JJ00000820
0.1984
0.1541
JJ00000821
0.1291
0.1896
N200000443
0.2807
N200000553
JJ00000953
0.3286
0.1343
N200000778
0.1577
JJ00001087
0.1268
0.2028
N200000741
0.3809
JJ00001196
N200000224
N200000411
0.1552
N200000779
0.1861
N200000721
0.1393
N200000758

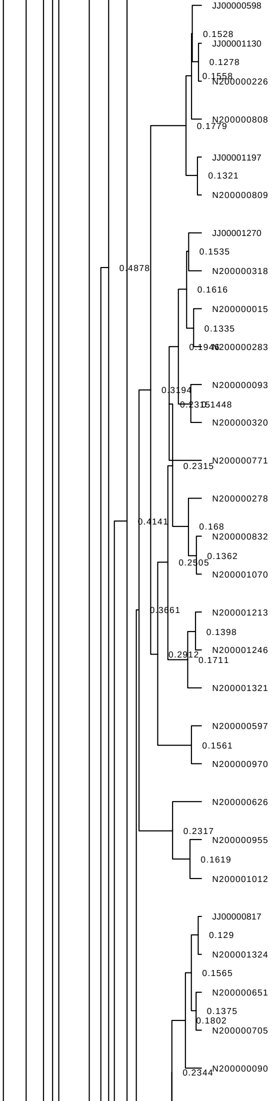
JJ00000598
0.1528
JJ00001130
0.1278
0.1558
N200000226
N200000808
0.1779
JJ00001197
0.1321
N200000809
JJ00001270
0.1535
0.4878
N200000318
0.1616
N200000015
0.1335
0.1946
N200000283
N200000093
0.3194
0.2315
0.1448
N200000320
N200000771
0.2315
N200000278
0.168
0.4141
N200000832
0.1362
0.2505
N200001070
0.3661
N200001213
0.1398
N200001246
0.2912
0.1711
N200001321
N200000597
0.1561
N200000970
N200000626
0.2317
N200000955
0.1619
N200001012
JJ00000817
0.129
N200001324
0.1565
N200000651
0.1375
0.1802
N200000705
N200000090
0.2344

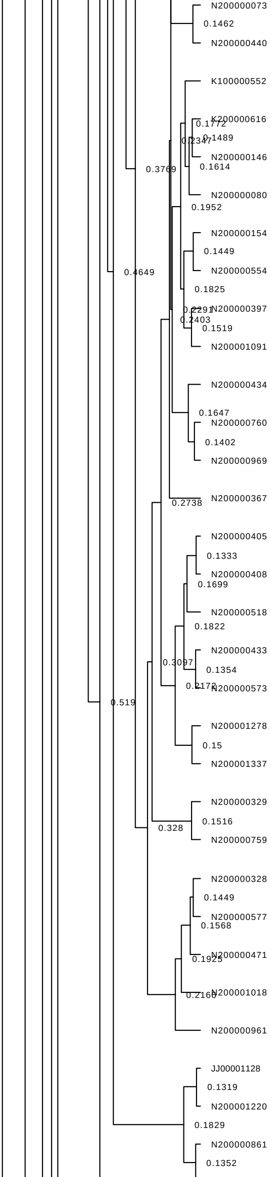
N200000073
0.1462
N200000440
K100000552
K200000616
0.1772
0.1489
0.2347
N200000146
0.3769
0.1614
N200000080
0.1952
N200000154
0.1449
N200000554
0.4649
0.1825
N200000397
0.2291
0.2403
0.1519
N200001091
N200000434
0.1647
N200000760
0.1402
N200000969
N200000367
0.2738
N200000405
0.1333
N200000408
0.1699
N200000518
0.1822
N200000433
0.3097
0.1354
0.2172
N200000573
0.519
N200001278
0.15
N200001337
N200000329
0.1516
0.328
N200000759
N200000328
0.1449
N200000577
0.1568
N200000471
0.1925
N200001018
0.2160
N200000961
JJ00001128
0.1319
N200001220
0.1829
N200000861
0.1352

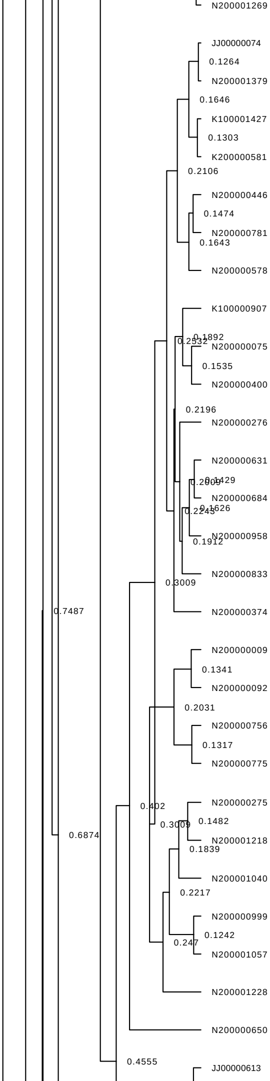
N200001269
JJ00000074
0.1264
N200001379
0.1646
K100001427
0.1303
K200000581
0.2106
N200000446
0.1474
N200000781
0.1643
N200000578
K100000907
0.1892
0.2532
N200000075
0.1535
N200000400
0.2196
N200000276
N200000631
0.1429
0.2005
N200000684
0.1626
0.2243
N200000958
0.1912
N200000833
0.3009
N200000374
0.7487
N200000009
0.1341
N200000092
0.2031
N200000756
0.1317
N200000775
N200000275
0.402
0.1482
0.3009
N200001218
0.6874
0.1839
N200001040
0.2217
N200000999
0.1242
0.247
N200001057
N200001228
N200000650
0.4555
JJ00000613

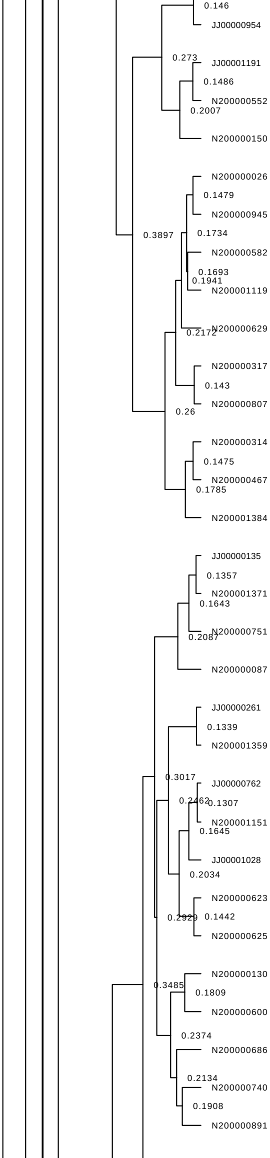
0.146
JJ00000954
0.273
JJ00001191
0.1486
N200000552
0.2007
N200000150
N200000026
0.1479
N200000945
0.3897
0.1734
N200000582
0.1693
0.1941
N200001119
N200000629
0.2172
N200000317
0.143
N200000807
0.26
N200000314
0.1475
N200000467
0.1785
N200001384
JJ00000135
0.1357
N200001371
0.1643
N200000751
0.2087
N200000087
JJ00000261
0.1339
N200001359
0.3017
JJ00000762
0.2462
0.1307
N200001151
0.1645
JJ00001028
0.2034
N200000623
0.1442
0.2929
N200000625
N200000130
0.3485
0.1809
N200000600
0.2374
N200000686
0.2134
N200000740
0.1908
N200000891

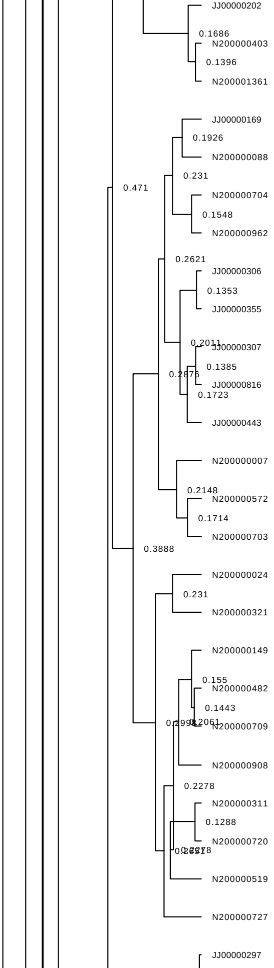
JJ00000202
0.1686
N200000403
0.1396
N200001361
JJ00000169
0.1926
N200000088
0.231
0.471
N200000704
0.1548
N200000962
0.2621
JJ00000306
0.1353
JJ00000355
0.2011
JJ00000307
0.1385
0.2876
JJ00000816
0.1723
JJ00000443
N200000007
0.2148
N200000572
0.1714
N200000703
0.3888
N200000024
0.231
N200000321
N200000149
0.155
N200000482
0.1443
N200000709
N200000908
0.2278
N200000311
0.1288
N200000720
N200000519
N200000727
JJ00000297

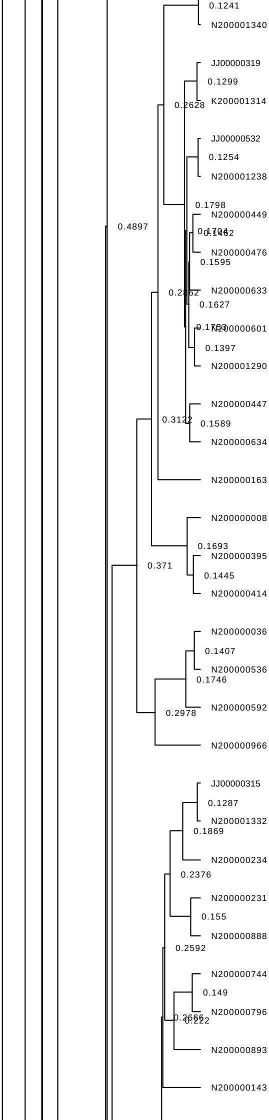
0.1241
N200001340
JJ00000319
0.1299
K200001314
0.2628
JJ00000532
0.1254
N200001238
0.1798
N200000449
0.4897
0.1704
0.1462
N200000476
0.1595
N200000633
0.2862
0.1627
0.1753
N200000601
0.1397
N200001290
N200000447
0.3122
0.1589
N200000634
N200000163
N200000008
0.1693
N200000395
0.371
0.1445
N200000414
N200000036
0.1407
N200000536
0.1746
N200000592
0.2978
N200000966
JJ00000315
0.1287
N200001332
0.1869
N200000234
0.2376
N200000231
0.155
N200000888
0.2592
N200000744
0.149
N200000796
0.2666
0.222
N200000893
N200000143

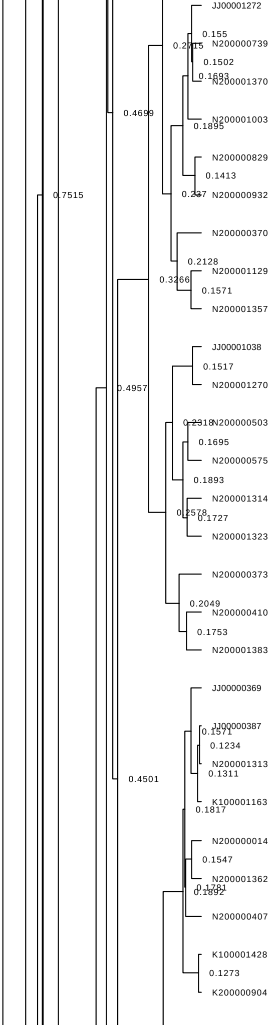
JJ00001272
0.155
0.2715
N200000739
0.1502
0.1693
N200001370
0.4699
N200001003
0.1895
N200000829
0.1413
0.7515
0.237
N200000932
N200000370
0.2128
N200001129
0.3266
0.1571
N200001357
JJ00001038
0.1517
N200001270
0.4957
0.2318
N200000503
0.1695
N200000575
0.1893
N200001314
0.2578
0.1727
N200001323
N200000373
0.2049
N200000410
0.1753
N200001383
JJ00000369
JJ00000387
0.1571
0.1234
N200001313
0.1311
0.4501
K100001163
0.1817
N200000014
0.1547
N200001362
0.1781
0.1892
N200000407
K100001428
0.1273
K200000904

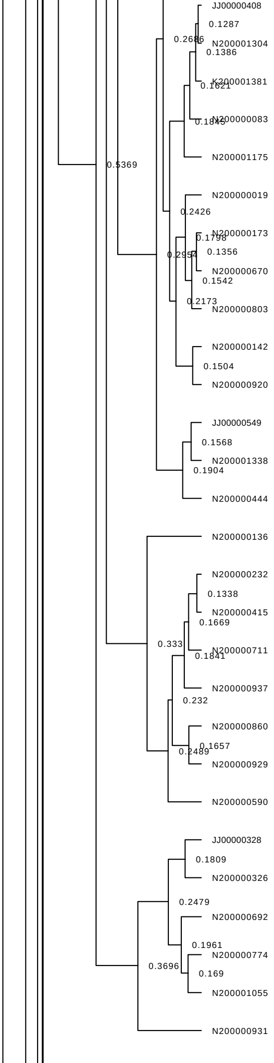
JJ00000408
0.1287
0.2686
N200001304
0.1386
K200001381
0.1621
N200000083
0.1845
N200001175
0.5369
N200000019
0.2426
N200000173
0.1798
0.1356
0.2954
N200000670
0.1542
0.2173
N200000803
N200000142
0.1504
N200000920
JJ00000549
0.1568
N200001338
0.1904
N200000444
N200000136
N200000232
0.1338
N200000415
0.1669
0.333
N200000711
0.1841
N200000937
0.232
N200000860
0.1657
0.2489
N200000929
N200000590
JJ00000328
0.1809
N200000326
0.2479
N200000692
0.1961
N200000774
0.3696
0.169
N200001055
N200000931

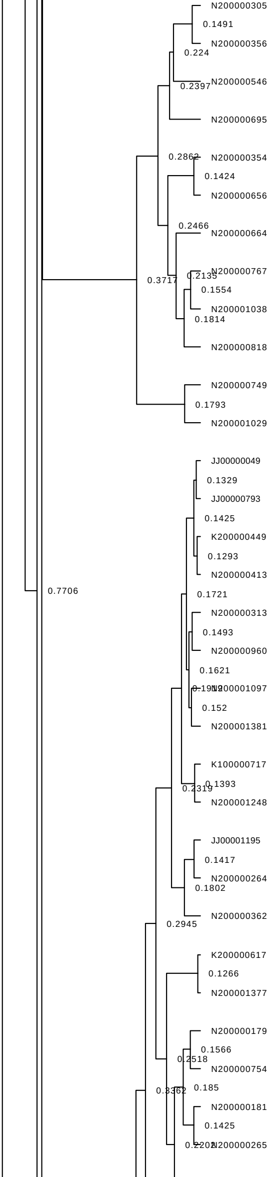
N200000305
0.1491
N200000356
0.224
0.2397
N200000546
N200000695
0.2862
N200000354
0.1424
N200000656
0.2466
N200000664
N200000767
0.2135
0.3717
0.1554
N200001038
0.1814
N200000818
N200000749
0.1793
N200001029
JJ00000049
0.1329
JJ00000793
0.1425
K200000449
0.1293
N200000413
0.7706
0.1721
N200000313
0.1493
N200000960
0.1621
N200001097
0.152
N200001381
K100000717
0.1393
0.2319
N200001248
JJ00001195
0.1417
N200000264
0.1802
N200000362
0.2945
K200000617
0.1266
N200001377
N200000179
0.1566
0.2518
N200000754
0.185
0.3362
N200000181
0.1425
N200000265

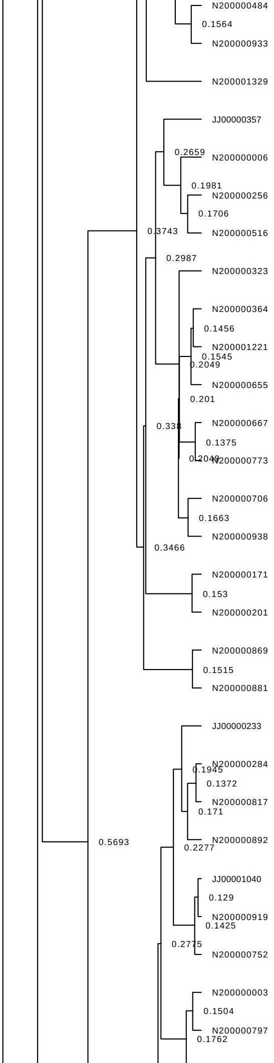

N200000484
0.1564
N200000933
N200001329
JJ00000357
0.2659
N200000006
0.1981
N200000256
0.1706
N200000516
0.3743
0.2987
N200000323
N200000364
0.1456
N200001221
0.1545
0.2049
N200000655
0.201
0.338
N200000667
0.1375
0.2049
N200000773
N200000706
0.1663
N200000938
0.3466
N200000171
0.153
N200000201
N200000869
0.1515
N200000881
JJ00000233
N200000284
0.1945
0.1372
N200000817
0.171
N200000892
0.5693
0.2277
JJ00001040
0.129
N200000919
0.1425
0.2775
N200000752
N200000003
0.1504
N200000797
0.1762

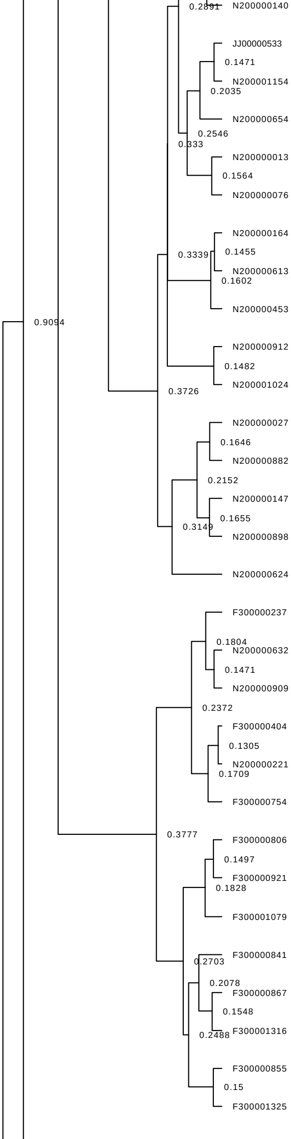
0.2891
N200000140
JJ00000533
0.1471
N200001154
0.2035
N200000654
0.2546
0.333
N200000013
0.1564
N200000076
N200000164
0.1455
0.3339
N200000613
0.1602
N200000453
0.9094
N200000912
0.1482
N200001024
0.3726
N200000027
0.1646
N200000882
0.2152
N200000147
0.1655
0.3149
N200000898
N200000624
F300000237
0.1804
N200000632
0.1471
N200000909
0.2372
F300000404
0.1305
N200000221
0.1709
F300000754
0.3777
F300000806
0.1497
F300000921
0.1828
F300001079
F300000841
0.2703
0.2078
F300000867
0.1548
F300001316
0.2488
F300000855
0.15
F300001325

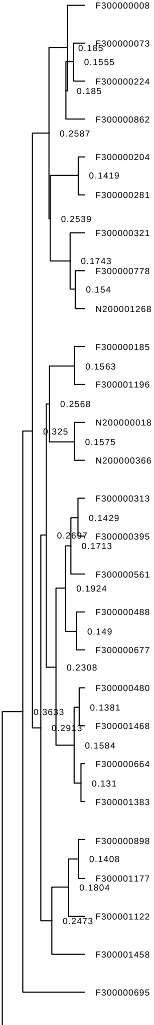
F300000008
0.185
F300000073
0.1555
F300000224
0.185
F300000862
0.2587
F300000204
0.1419
F300000281
0.2539
F300000321
0.1743
F300000778
0.154
N200001268
F300000185
0.1563
F300001196
0.2568
N200000018
0.325
0.1575
N200000366
F300000313
0.1429
0.2697
F300000395
0.1713
F300000561
0.1924
F300000488
0.149
F300000677
0.2308
F300000480
0.1381
0.3633
F300001468
0.2913
0.1584
F300000664
0.131
F300001383
F300000898
0.1408
F300001177
0.1804
F300001122
0.2473
F300001458
F300000695

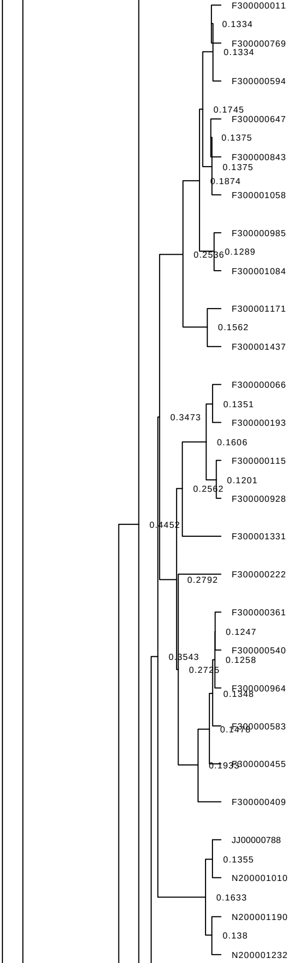
F300000011
0.1334
F300000769
0.1334
F300000594
0.1745
F300000647
0.1375
F300000843
0.1375
0.1874
F300001058
F300000985
0.2536
0.1289
F300001084
F300001171
0.1562
F300001437
F300000066
0.1351
0.3473
F300000193
0.1606
F300000115
0.1201
0.2562
F300000928
0.4452
F300001331
F300000222
0.2792
F300000361
0.1247
F300000540
0.3543
0.1258
0.2725
F300000964
0.1348
F300000583
0.1478
F300000455
0.1933
F300000409
JJ00000788
0.1355
N200001010
0.1633
N200001190
0.138
N200001232

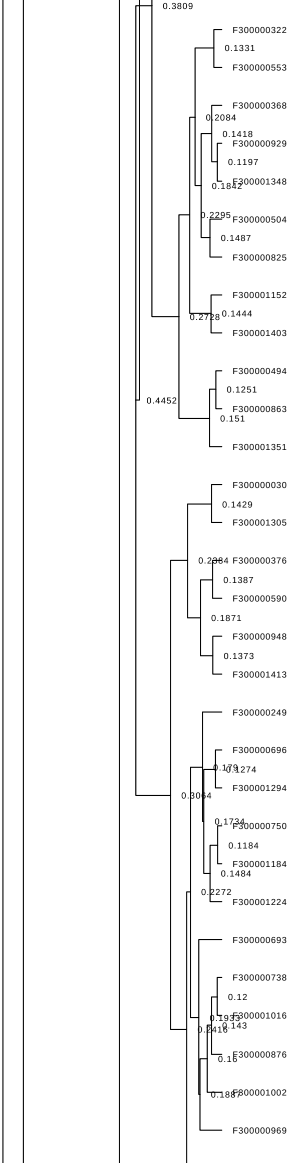

0.3809
F300000322
0.1331
F300000553
F300000368
0.2084
0.1418
F300000929
0.1197
F300001348
0.1842
0.2295
F300000504
0.1487
F300000825
F300001152
0.1444
0.2728
F300001403
F300000494
0.1251
0.4452
F300000863
0.151
F300001351
F300000030
0.1429
F300001305
0.2384
F300000376
0.1387
F300000590
0.1871
F300000948
0.1373
F300001413
F300000249
F300000696
0.179
0.1274
F300001294
0.3064
0.1734
F300000750
0.1184
F300001184
0.1484
0.2272
F300001224
F300000693
F300000738
0.12
F300001016
0.1933
0.143
0.2416
F300000876
0.16
F300001002
0.1887
F300000969

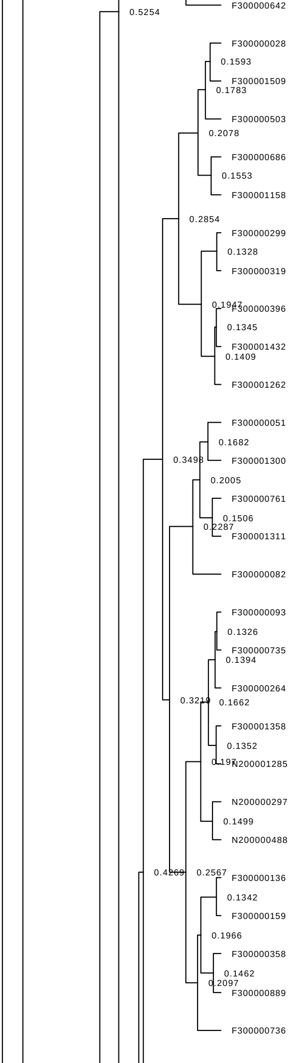

F300000642
0.5254
F300000028
0.1593
F300001509
0.1783
F300000503
0.2078
F300000686
0.1553
F300001158
0.2854
F300000299
0.1328
F300000319
0.1947
F300000396
0.1345
F300001432
0.1409
F300001262
F300000051
0.1682
0.3498
F300001300
0.2005
F300000761
0.1506
0.2287
F300001311
F300000082
F300000093
0.1326
F300000735
0.1394
F300000264
0.3219
0.1662
F300001358
0.1352
0.197
N200001285
N200000297
0.1499
N200000488
0.4269
0.2567
F300000136
0.1342
F300000159
0.1966
F300000358
0.1462
0.2097
F300000889
F300000736

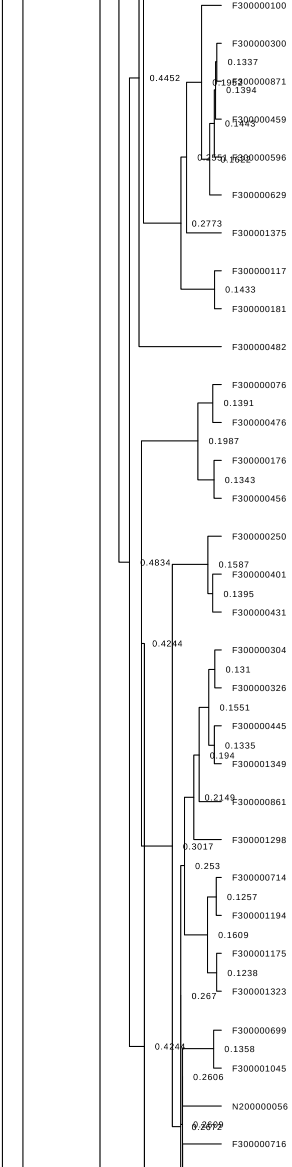
F300000100
F300000300
0.1337
0.4452
0.1952
F300000871
0.1394
F300000459
0.1443
0.2551
F300000596
0.1622
F300000629
0.2773
F300001375
F300000117
0.1433
F300000181
F300000482
F300000076
0.1391
F300000476
0.1987
F300000176
0.1343
F300000456
F300000250
0.4834
0.1587
F300000401
0.1395
F300000431
0.4244
F300000304
0.131
F300000326
0.1551
F300000445
0.1335
0.194
F300001349
0.2149
F300000861
F300001298
0.3017
0.253
F300000714
0.1257
F300001194
0.1609
F300001175
0.1238
F300001323
0.267
F300000699
0.4244
0.1358
F300001045
0.2606
N200000056
0.2609
0.2672
F300000716

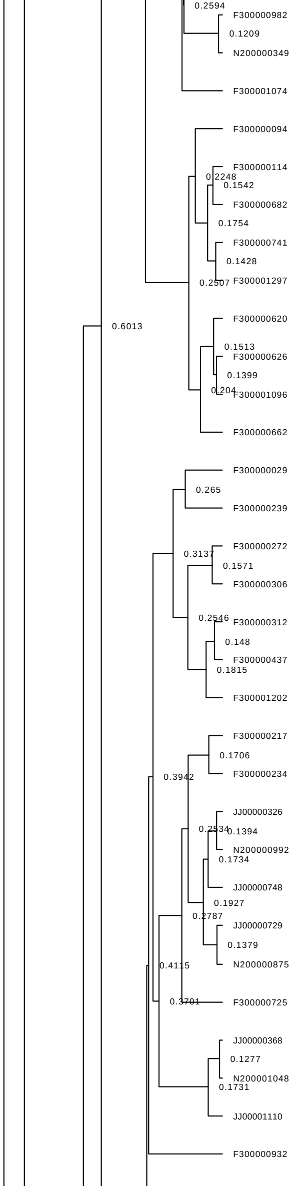
0.2594
F300000982
0.1209
N200000349
F300001074
F300000094
F300000114
0.2248
0.1542
F300000682
0.1754
F300000741
0.1428
F300001297
0.2507
0.6013
F300000620
0.1513
F300000626
0.1399
0.204
F300001096
F300000662
F300000029
0.265
F300000239
F300000272
0.3137
0.1571
F300000306
0.2546
F300000312
0.148
F300000437
0.1815
F300001202
F300000217
0.1706
F300000234
0.3942
JJ00000326
0.2534
0.1394
N200000992
0.1734
JJ00000748
0.1927
0.2787
JJ00000729
0.1379
N200000875
0.4115
0.3701
F300000725
JJ00000368
0.1277
N200001048
0.1731
JJ00001110
F300000932

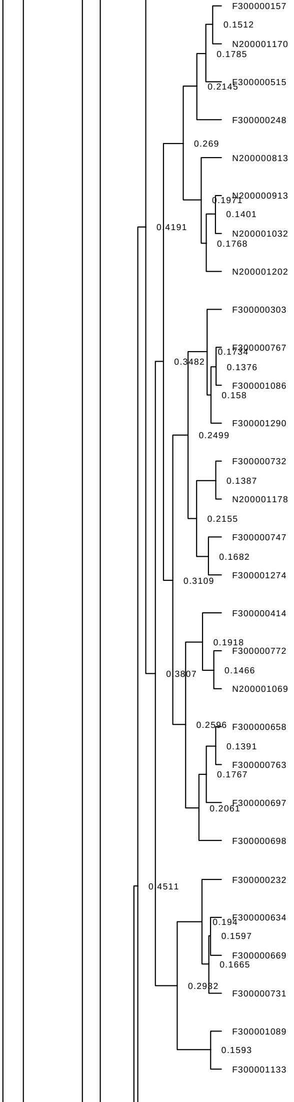
F300000157
0.1512
N200001170
0.1785
F300000515
0.2145
F300000248
0.269
N200000813
N200000913
0.1971
0.1401
N200001032
0.1768
0.4191
N200001202
F300000303
F300000767
0.1734
0.3482
0.1376
F300001086
0.158
F300001290
0.2499
F300000732
0.1387
N200001178
0.2155
F300000747
0.1682
F300001274
0.3109
F300000414
0.1918
F300000772
0.1466
0.3807
N200001069
0.2596
F300000658
0.1391
F300000763
0.1767
F300000697
0.2061
F300000698
F300000232
0.4511
F300000634
0.194
0.1597
F300000669
0.1665
0.2982
F300000731
F300001089
0.1593
F300001133

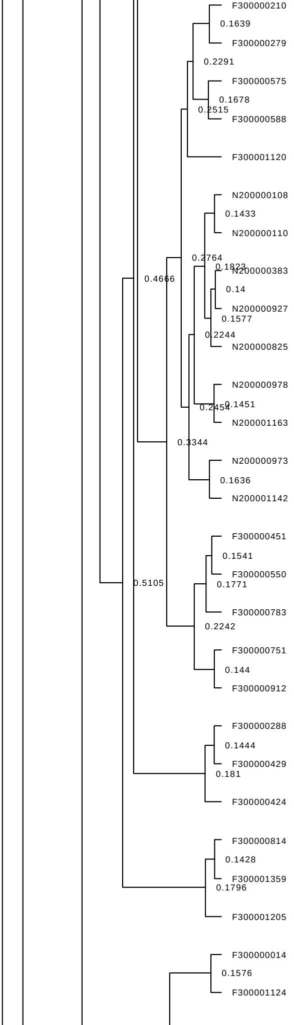
F300000210
0.1639
F300000279
0.2291
F300000575
0.1678
0.2515
F300000588
F300001120
N200000108
0.1433
N200000110
0.2764
0.1823
N200000383
0.4666
0.14
N200000927
0.1577
0.2244
N200000825
N200000978
0.1451
0.2454
N200001163
0.3344
N200000973
0.1636
N200001142
F300000451
0.1541
F300000550
0.5105
0.1771
F300000783
0.2242
F300000751
0.144
F300000912
F300000288
0.1444
F300000429
0.181
F300000424
F300000814
0.1428
F300001359
0.1796
F300001205
F300000014
0.1576
F300001124

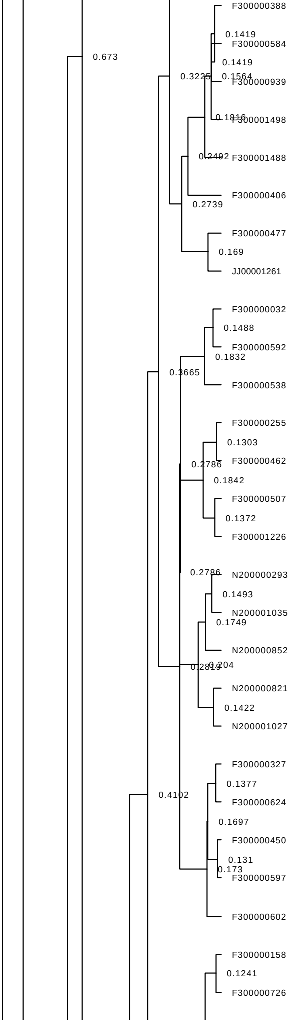

F300000388
0.1419
F300000584
0.673
0.1419
0.3225
0.1564
F300000939
0.1816
F300001498
0.2492
F300001488
F300000406
0.2739
F300000477
0.169
JJ00001261
F300000032
0.1488
F300000592
0.1832
0.3665
F300000538
F300000255
0.1303
F300000462
0.2786
0.1842
F300000507
0.1372
F300001226
0.2786
N200000293
0.1493
N200001035
0.1749
N200000852
0.2819
0.204
N200000821
0.1422
N200001027
F300000327
0.1377
0.4102
F300000624
0.1697
F300000450
0.131
0.173
F300000597
F300000602
F300000158
0.1241
F300000726

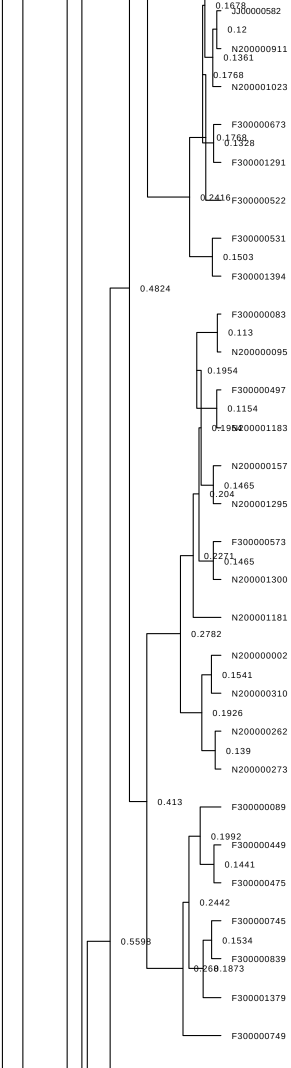
0.1678
JJ00000582
0.12
N200000911
0.1361
0.1768
N200001023
F300000673
0.1768
0.1328
F300001291
0.2416
F300000522
F300000531
0.1503
F300001394
0.4824
F300000083
0.113
N200000095
0.1954
F300000497
0.1154
0.1954
N200001183
N200000157
0.1465
0.204
N200001295
F300000573
0.2271
0.1465
N200001300
N200001181
0.2782
N200000002
0.1541
N200000310
0.1926
N200000262
0.139
N200000273
0.413
F300000089
0.1992
F300000449
0.1441
F300000475
0.2442
F300000745
0.1534
F300000839
0.5598
0.268
0.1873
F300001379
F300000749

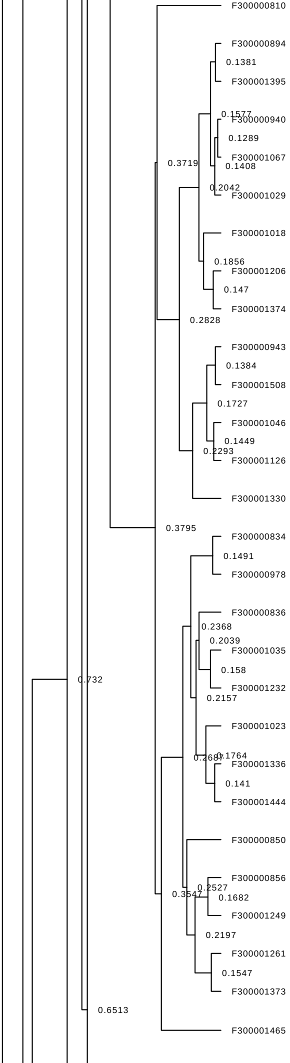
F300000810
F300000894
0.1381
F300001395
0.1577
F300000940
0.1289
F300001067
0.3719
0.1408
0.2042
F300001029
F300001018
0.1856
F300001206
0.147
F300001374
0.2828
F300000943
0.1384
F300001508
0.1727
F300001046
0.1449
0.2293
F300001126
F300001330
0.3795
F300000834
0.1491
F300000978
F300000836
0.2368
0.2039
F300001035
0.158
0.732
F300001232
0.2157
F300001023
0.2687
0.1764
F300001336
0.141
F300001444
F300000850
F300000856
0.2527
0.3547
0.1682
F300001249
0.2197
F300001261
0.1547
F300001373
0.6513
F300001465

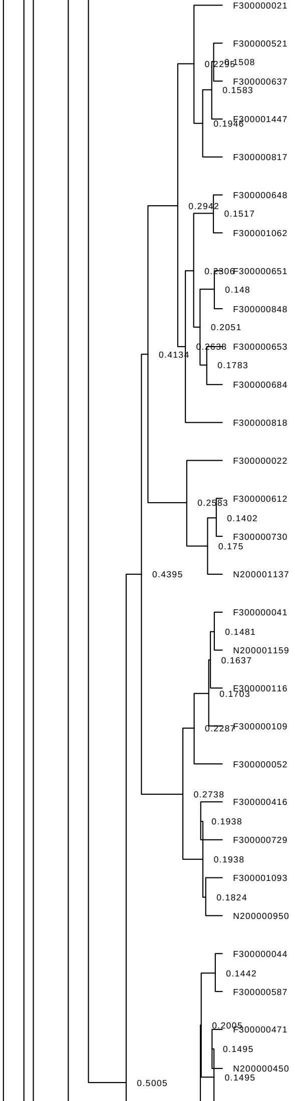
F300000021
F300000521
0.2295
0.1508
F300000637
0.1583
F300001447
0.1946
F300000817
F300000648
0.2942
0.1517
F300001062
0.2306
F300000651
0.148
F300000848
0.2051
0.2638
F300000653
0.4134
0.1783
F300000684
F300000818
F300000022
F300000612
0.2583
0.1402
F300000730
0.175
0.4395
N200001137
F300000041
0.1481
N200001159
0.1637
F300000116
0.1703
F300000109
0.2287
F300000052
0.2738
F300000416
0.1938
F300000729
0.1938
F300001093
0.1824
N200000950
F300000044
0.1442
F300000587
0.2005
F300000471
0.1495
N200000450
0.1495
0.5005

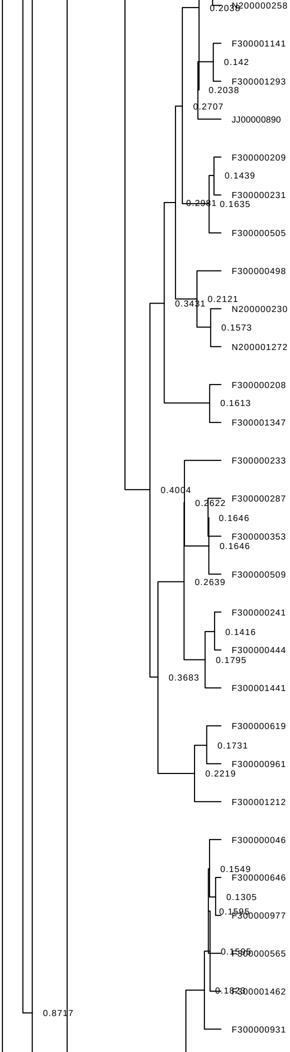

0.2038
N200000258
F300001141
0.142
F300001293
0.2038
0.2707
JJ00000890
F300000209
0.1439
F300000231
0.2981
0.1635
F300000505
F300000498
0.3431
0.2121
N200000230
0.1573
N200001272
F300000208
0.1613
F300001347
F300000233
0.4004
0.2622
F300000287
0.1646
F300000353
0.1646
F300000509
0.2639
F300000241
0.1416
F300000444
0.1795
0.3683
F300001441
F300000619
0.1731
F300000961
0.2219
F300001212
F300000046
0.1549
F300000646
0.1305
0.1595
F300000977
0.1595
F300000565
0.1828
F300001462
0.8717
F300000931

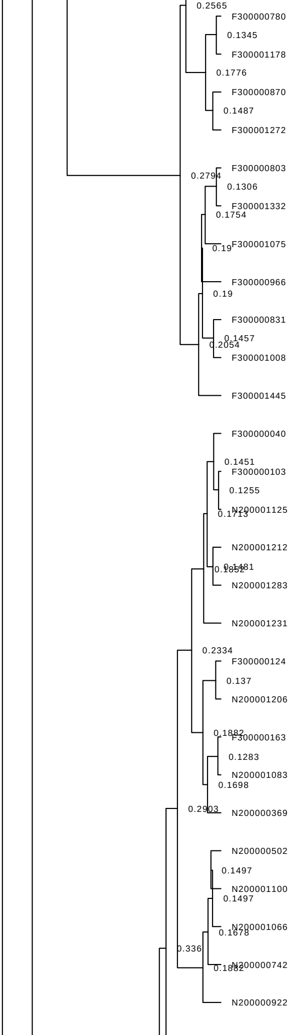

0.2565
F300000780
0.1345
F300001178
0.1776
F300000870
0.1487
F300001272
F300000803
0.2794
0.1306
F300001332
0.1754
0.19
F300001075
F300000966
0.19
F300000831
0.1457
0.2054
F300001008
F300001445
F300000040
0.1451
F300000103
0.1255
N200001125
0.1713
N200001212
0.1481
0.1852
N200001283
N200001231
0.2334
F300000124
0.137
N200001206
0.1882
F300000163
0.1283
N200001083
0.1698
0.2903
N200000369
N200000502
0.1497
N200001100
0.1497
N200001066
0.1678
0.336
N200000742
0.1882
N200000922

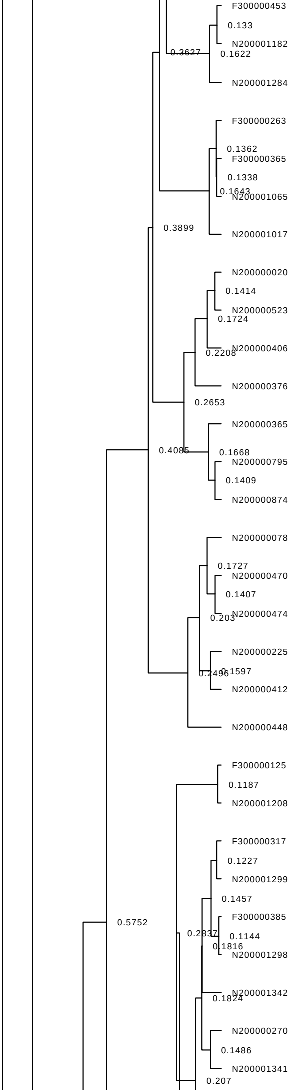
F300000453
0.133
N200001182
0.3627
0.1622
N200001284
F300000263
0.1362
F300000365
0.1338
0.1643
N200001065
0.3899
N200001017
N200000020
0.1414
N200000523
0.1724
N200000406
0.2208
N200000376
0.2653
N200000365
0.4085
0.1668
N200000795
0.1409
N200000874
N200000078
0.1727
N200000470
0.1407
N200000474
0.203
N200000225
0.2496
0.1597
N200000412
N200000448
F300000125
0.1187
N200001208
F300000317
0.1227
N200001299
0.1457
F300000385
0.5752
0.2837
0.1144
0.1816
N200001298
N200001342
0.1824
N200000270
0.1486
N200001341
0.207

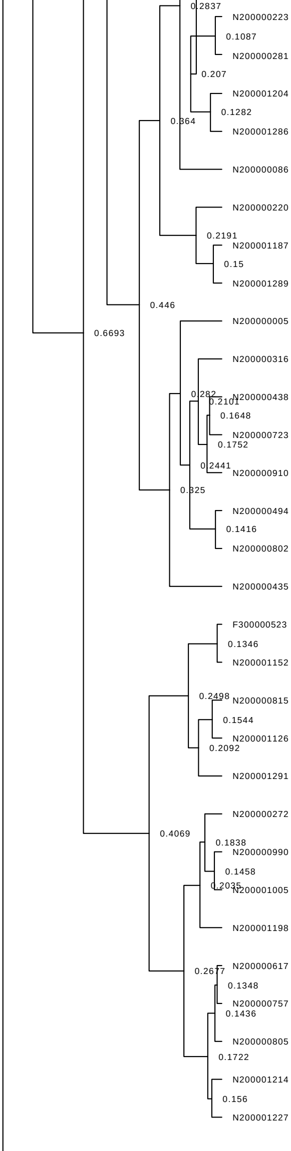
0.2837
N200000223
0.1087
N200000281
0.207
N200001204
0.1282
N200001286
0.364
N200000086
N200000220
0.2191
N200001187
0.15
N200001289
0.446
0.6693
N200000005
N200000316
0.282
N200000438
0.2101
0.1648
N200000723
0.1752
0.2441
N200000910
0.325
N200000494
0.1416
N200000802
N200000435
F300000523
0.1346
N200001152
0.2498
N200000815
0.1544
N200001126
0.2092
N200001291
N200000272
0.4069
0.1838
N200000990
0.1458
0.2035
N200001005
N200001198
N200000617
0.2677
0.1348
N200000757
0.1436
N200000805
0.1722
N200001214
0.156
N200001227

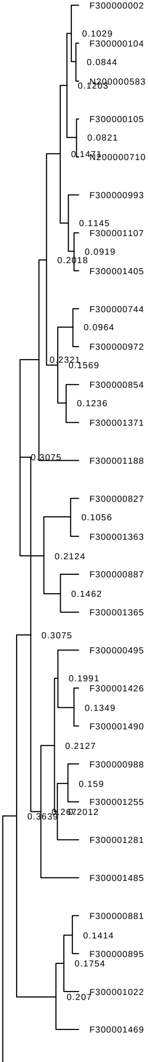

F300000002
0.1029
F300000104
0.0844
N200000583
0.1203
F300000105
0.0821
0.1471
N200000710
F300000993
0.1145
F300001107
0.0919
0.2018
F300001405
F300000744
0.0964
F300000972
0.2321
0.1569
F300000854
0.1236
F300001371
0.3075
F300001188
F300000827
0.1056
F300001363
0.2124
F300000887
0.1462
F300001365
0.3075
F300000495
0.1991
F300001426
0.1349
F300001490
0.2127
F300000988
0.159
F300001255
0.3639
F300001281
F300001485
F300000881
0.1414
F300000895
0.1754
F300001022
0.207
F300001469

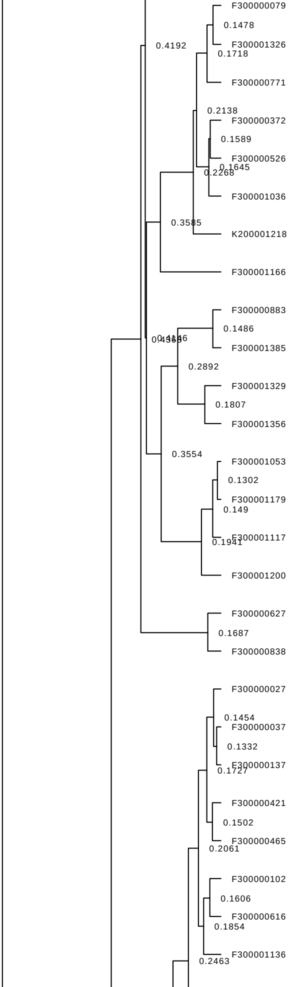
F300000079
0.1478
F300001326
0.4192
0.1718
F300000771
0.2138
F300000372
0.1589
F300000526
0.1645
0.2268
F300001036
0.3585
K200001218
F300001166
F300000883
0.1486
F300001385
0.2892
F300001329
0.1807
F300001356
0.3554
F300001053
0.1302
F300001179
0.149
F300001117
0.1941
F300001200
F300000627
0.1687
F300000838
F300000027
0.1454
F300000037
0.1332
F300000137
0.1727
F300000421
0.1502
F300000465
0.2061
F300000102
0.1606
F300000616
0.1854
F300001136
0.2463

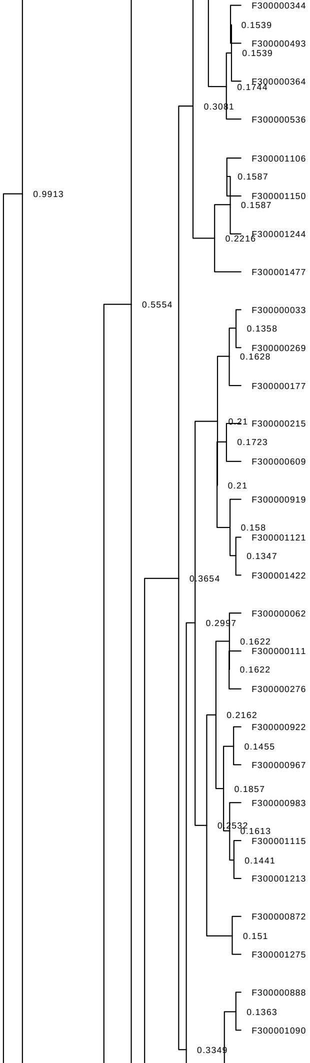
F300000344
0.1539
F300000493
0.1539
F300000364
0.1744
0.3081
F300000536
F300001106
0.1587
F300001150
0.1587
F300001244
0.2216
F300001477
0.9913
0.5554
F300000033
0.1358
F300000269
0.1628
F300000177
0.21
F300000215
0.1723
F300000609
0.21
F300000919
0.158
F300001121
0.1347
F300001422
0.3654
F300000062
0.2997
0.1622
F300000111
0.1622
F300000276
0.2162
F300000922
0.1455
F300000967
0.1857
F300000983
0.2532
0.1613
F300001115
0.1441
F300001213
F300000872
0.151
F300001275
F300000888
0.1363
F300001090
0.3349

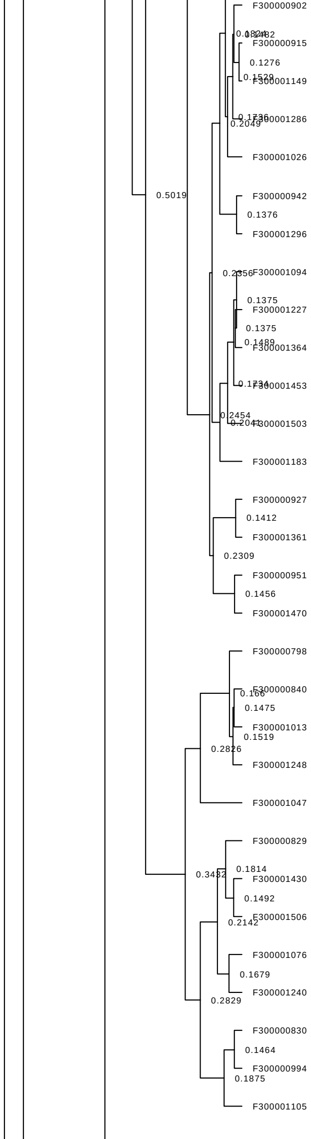
F300000902
0.1824
0.1482
F300000915
0.1276
0.1529
F300001149
0.1736
F300001286
0.2049
F300001026
0.5019
F300000942
0.1376
F300001296
0.2356
F300001094
0.1375
F300001227
0.1375
0.1489
F300001364
0.1734
F300001453
0.2454
0.2041
F300001503
F300001183
F300000927
0.1412
F300001361
0.2309
F300000951
0.1456
F300001470
F300000798
F300000840
0.166
0.1475
F300001013
0.1519
0.2826
F300001248
F300001047
F300000829
0.1814
0.3432
F300001430
0.1492
F300001506
0.2142
F300001076
0.1679
F300001240
0.2829
F300000830
0.1464
F300000994
0.1875
F300001105

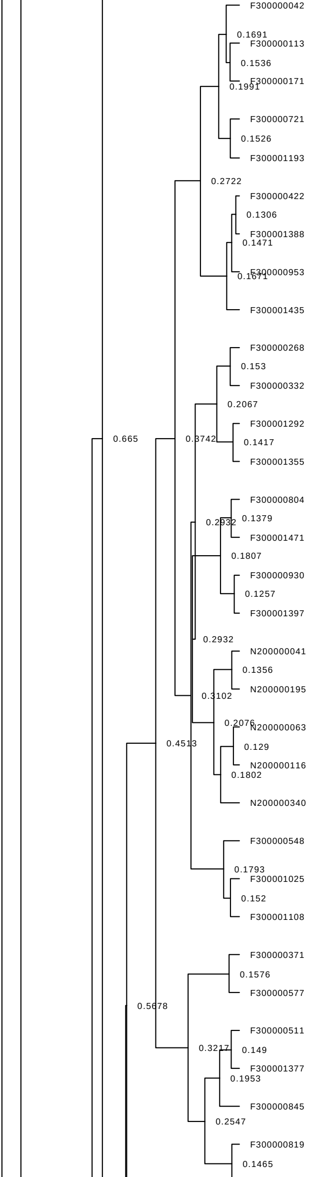
F300000042
0.1691
F300000113
0.1536
F300000171
0.1991
F300000721
0.1526
F300001193
0.2722
F300000422
0.1306
F300001388
0.1471
F300000953
0.1671
F300001435
F300000268
0.153
F300000332
0.2067
F300001292
0.665
0.3742
0.1417
F300001355
F300000804
0.1379
0.2932
F300001471
0.1807
F300000930
0.1257
F300001397
0.2932
N200000041
0.1356
N200000195
0.3102
0.2076
N200000063
0.4513
0.129
N200000116
0.1802
N200000340
F300000548
0.1793
F300001025
0.152
F300001108
F300000371
0.1576
F300000577
0.5678
F300000511
0.3217
0.149
F300001377
0.1953
F300000845
0.2547
F300000819
0.1465

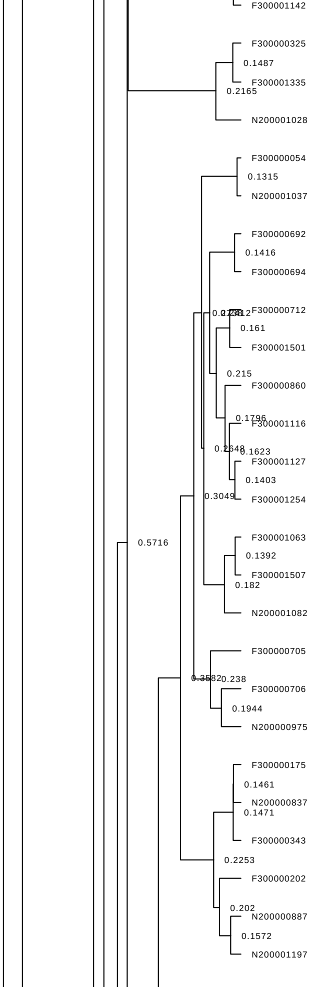
F300001142
F300000325
0.1487
F300001335
0.2165
N200001028
F300000054
0.1315
N200001037
F300000692
0.1416
F300000694
F300000712
0.161
F300001501
0.215
F300000860
0.1796
F300001116
0.2648
0.1623
F300001127
0.1403
F300001254
0.3049
F300001063
0.1392
F300001507
0.182
N200001082
0.5716
F300000705
0.3582
0.238
F300000706
0.1944
N200000975
F300000175
0.1461
N200000837
0.1471
F300000343
0.2253
F300000202
0.202
N200000887
0.1572
N200001197

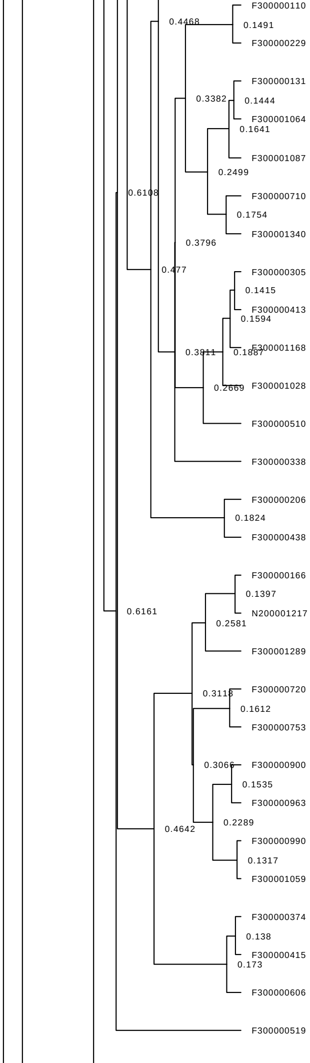
F300000110
0.4468
0.1491
F300000229
F300000131
0.3382
0.1444
F300001064
0.1641
F300001087
0.2499
0.6108
F300000710
0.1754
F300001340
0.3796
0.477
F300000305
0.1415
F300000413
0.1594
0.3811
0.1887
F300001168
0.2669
F300001028
F300000510
F300000338
F300000206
0.1824
F300000438
F300000166
0.1397
0.6161
N200001217
0.2581
F300001289
0.3118
F300000720
0.1612
F300000753
0.3066
F300000900
0.1535
F300000963
0.2289
0.4642
F300000990
0.1317
F300001059
F300000374
0.138
F300000415
0.173
F300000606
F300000519

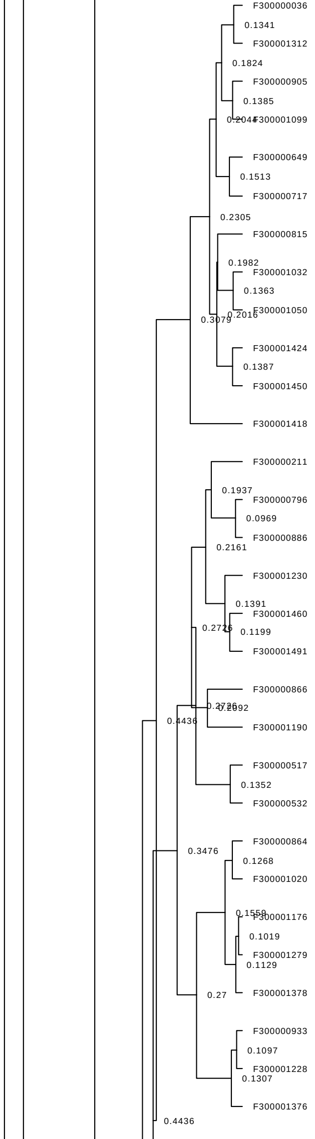
F300000036
0.1341
F300001312
0.1824
F300000905
0.1385
0.2044
F300001099
F300000649
0.1513
F300000717
0.2305
F300000815
0.1982
F300001032
0.1363
F300001050
0.2016
0.3079
F300001424
0.1387
F300001450
F300001418
F300000211
0.1937
F300000796
0.0969
F300000886
0.2161
F300001230
0.1391
F300001460
0.2726
0.1199
F300001491
F300000866
0.2726
0.2092
0.4436
F300001190
F300000517
0.1352
F300000532
F300000864
0.3476
0.1268
F300001020
0.1559
F300001176
0.1019
F300001279
0.1129
F300001378
0.27
F300000933
0.1097
F300001228
0.1307
F300001376
0.4436

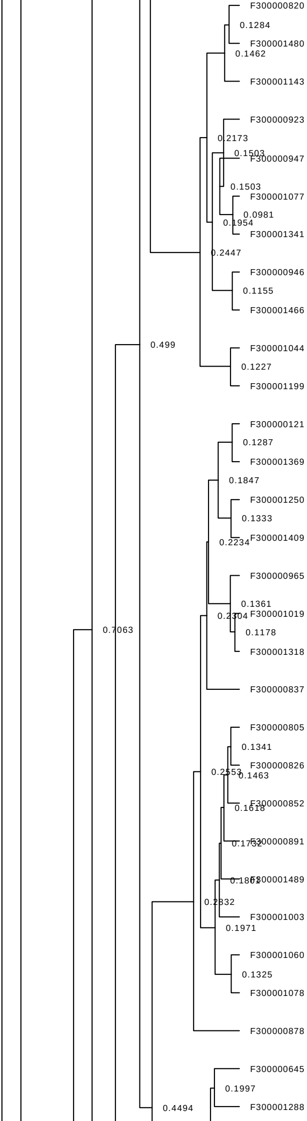
F300000820
0.1284
F300001480
0.1462
F300001143
F300000923
0.2173
0.1503
F300000947
0.1503
F300001077
0.0981
0.1954
F300001341
0.2447
F300000946
0.1155
F300001466
0.499
F300001044
0.1227
F300001199
F300000121
0.1287
F300001369
0.1847
F300001250
0.1333
F300001409
0.2234
F300000965
0.1361
0.2304
F300001019
0.1178
0.7063
F300001318
F300000837
F300000805
0.1341
F300000826
0.2553
0.1463
F300000852
0.1618
F300000891
0.1732
F300001489
0.1861
0.2332
F300001003
0.1971
F300001060
0.1325
F300001078
F300000878
F300000645
0.1997
0.4494
F300001288

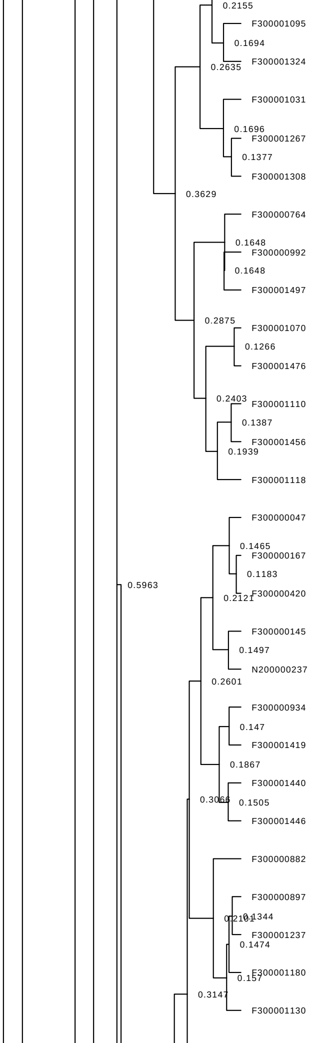
0.2155
F300001095
0.1694
F300001324
0.2635
F300001031
0.1696
F300001267
0.1377
F300001308
0.3629
F300000764
0.1648
F300000992
0.1648
F300001497
0.2875
F300001070
0.1266
F300001476
0.2403
F300001110
0.1387
F300001456
0.1939
F300001118
F300000047
0.1465
F300000167
0.1183
F300000420
0.2121
0.5963
F300000145
0.1497
N200000237
0.2601
F300000934
0.147
F300001419
0.1867
F300001440
0.3066
0.1505
F300001446
F300000882
F300000897
0.2101
0.1344
F300001237
0.1474
F300001180
0.157
0.3147
F300001130

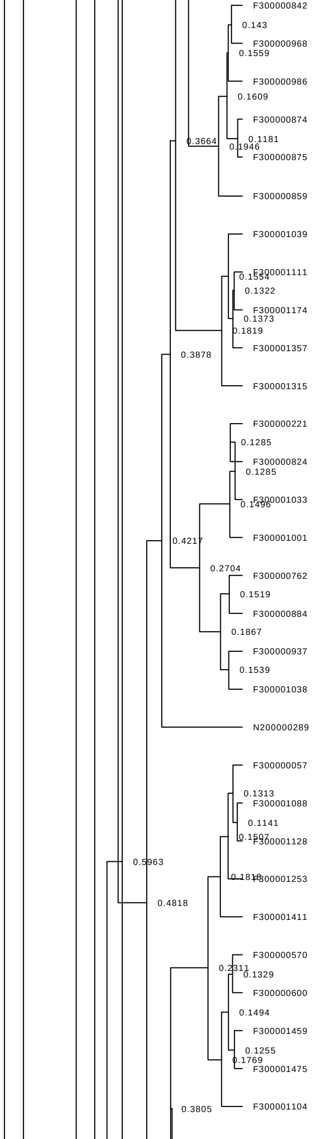
F300000842
0.143
F300000968
0.1559
F300000986
0.1609
F300000874
0.1181
0.3664
0.1946
F300000875
F300000859
F300001039
F300001111
0.1554
0.1322
F300001174
0.1373
0.1819
F300001357
0.3878
F300001315
F300000221
0.1285
F300000824
0.1285
F300001033
0.1496
F300001001
0.4217
0.2704
F300000762
0.1519
F300000884
0.1867
F300000937
0.1539
F300001038
N200000289
F300000057
0.1313
F300001088
0.1141
0.1507
F300001128
0.5963
0.1818
F300001253
0.4818
F300001411
F300000570
0.2311
0.1329
F300000600
0.1494
F300001459
0.1255
0.1769
F300001475
F300001104
0.3805

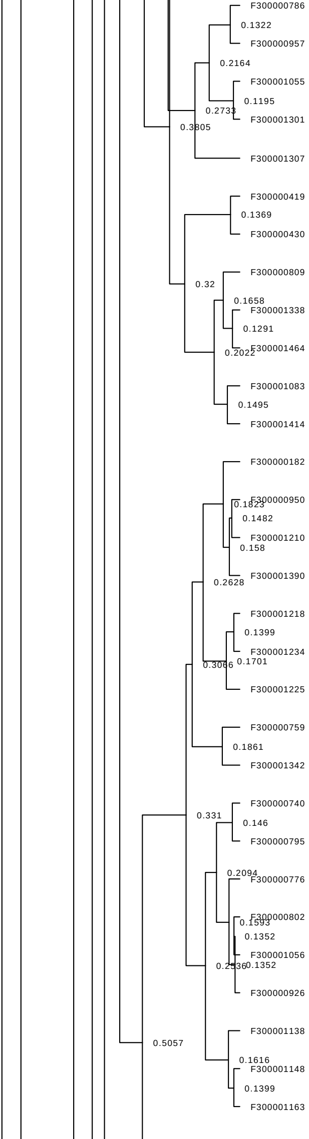

F300000786
0.1322
F300000957
0.2164
F300001055
0.1195
0.2733
F300001301
0.3805
F300001307
F300000419
0.1369
F300000430
F300000809
0.32
0.1658
F300001338
0.1291
F300001464
0.2022
F300001083
0.1495
F300001414
F300000182
F300000950
0.1823
0.1482
F300001210
0.158
F300001390
0.2628
F300001218
0.1399
F300001234
0.3066
0.1701
F300001225
F300000759
0.1861
F300001342
F300000740
0.331
0.146
F300000795
0.2094
F300000776
F300000802
0.1593
0.1352
F300001056
0.2536
0.1352
F300000926
F300001138
0.5057
0.1616
F300001148
0.1399
F300001163

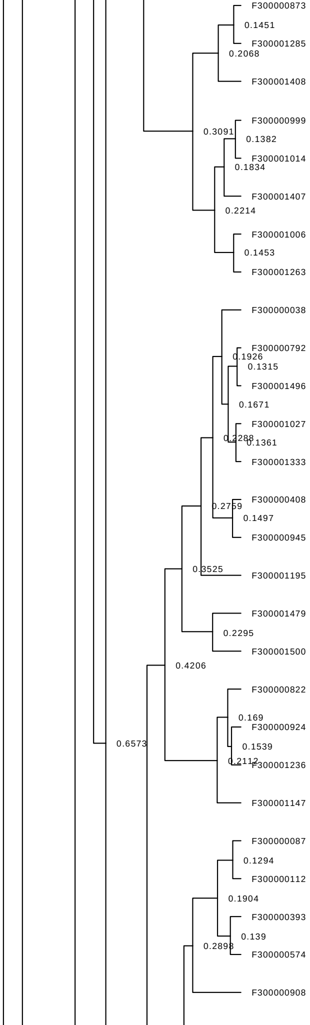
F300000873
0.1451
F300001285
0.2068
F300001408
F300000999
0.3091
0.1382
F300001014
0.1834
F300001407
0.2214
F300001006
0.1453
F300001263
F300000038
F300000792
0.1926
0.1315
F300001496
0.1671
F300001027
0.2288
0.1361
F300001333
F300000408
0.2759
0.1497
F300000945
0.3525
F300001195
F300001479
0.2295
F300001500
0.4206
F300000822
0.169
F300000924
0.6573
0.1539
0.2112
F300001236
F300001147
F300000087
0.1294
F300000112
0.1904
F300000393
0.139
0.2898
F300000574
F300000908

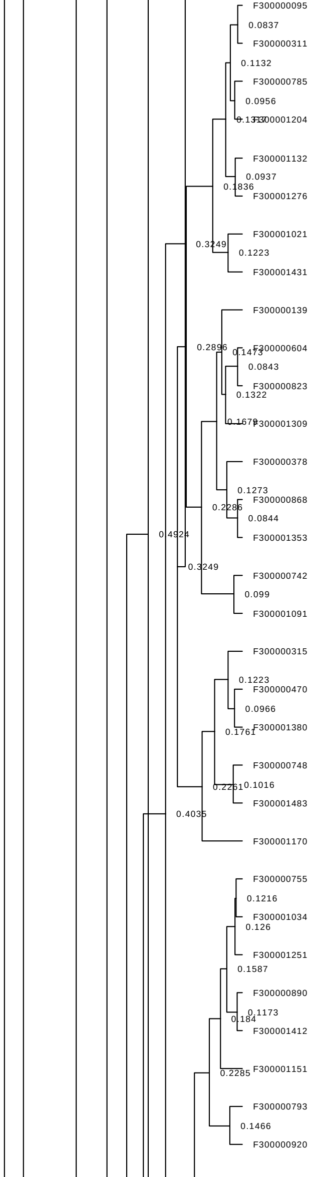

F300000095
0.0837
F300000311
0.1132
F300000785
0.0956
F300001204
F300001132
0.0937
0.1836
F300001276
F300001021
0.3249
0.1223
F300001431
F300000139
0.2896
F300000604
0.1473
0.0843
F300000823
0.1322
0.1679
F300001309
F300000378
0.1273
F300000868
0.2286
0.0844
F300001353
0.4924
0.3249
F300000742
0.099
F300001091
F300000315
0.1223
F300000470
0.0966
F300001380
0.1761
F300000748
0.2261
0.1016
F300001483
0.4035
F300001170
F300000755
0.1216
F300001034
0.126
F300001251
0.1587
F300000890
0.1173
0.184
F300001412
F300001151
0.2285
F300000793
0.1466
F300000920

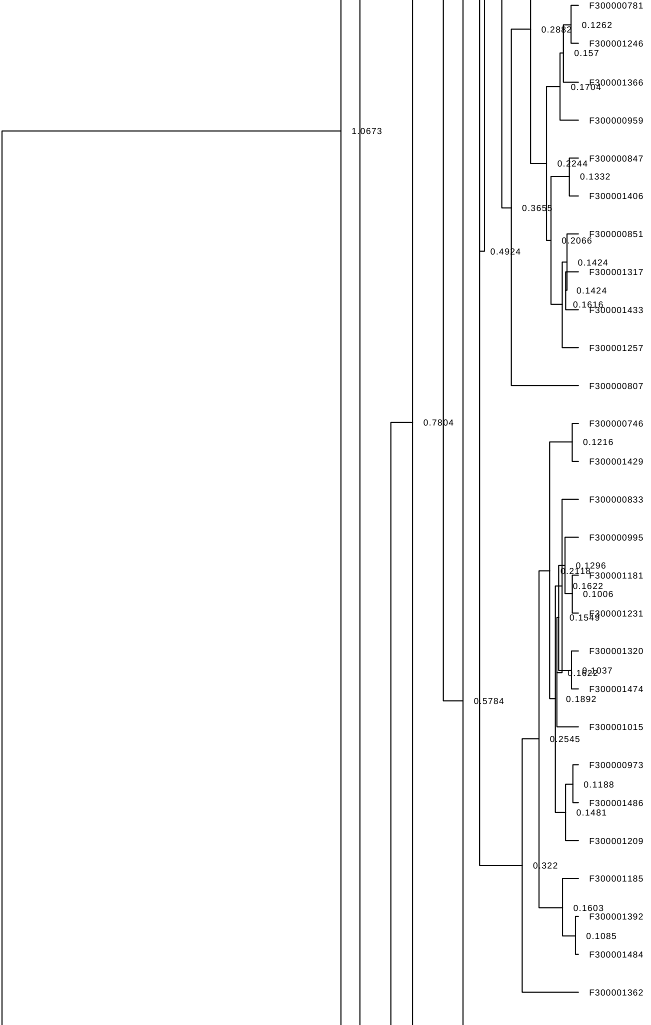
F300000781
0.1262
0.2882
F300001246
0.157
F300001366
0.1704
F300000959
1.0673
F300000847
0.2244
0.1332
F300001406
0.3655
F300000851
0.2066
0.4924
0.1424
F300001317
0.1424
0.1616
F300001433
F300001257
F300000807
0.7804
F300000746
0.1216
F300001429
F300000833
F300000995
0.1296
0.2118
F300001181
0.1622
0.1006
F300001231
0.1549
F300001320
0.1037
0.1622
F300001474
0.1892
0.5784
F300001015
0.2545
F300000973
0.1188
F300001486
0.1481
F300001209
0.322
F300001185
0.1603
F300001392
0.1085
F300001484
F300001362

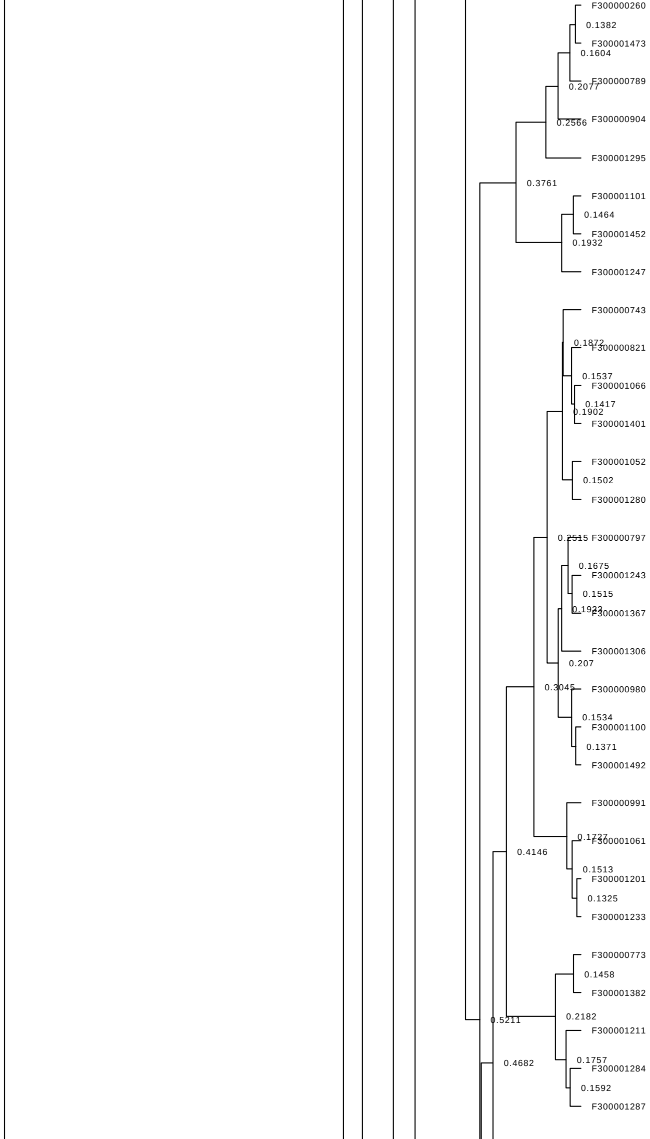
F300000260
0.1382
F300001473
0.1604
F300000789
0.2077
F300000904
0.2566
F300001295
0.3761
F300001101
0.1464
F300001452
0.1932
F300001247
F300000743
0.1872
F300000821
0.1537
F300001066
0.1417
0.1902
F300001401
F300001052
0.1502
F300001280
0.2515
F300000797
0.1675
F300001243
0.1515
0.1933
F300001367
F300001306
0.207
0.3045
F300000980
0.1534
F300001100
0.1371
F300001492
F300000991
0.1727
F300001061
0.1513
F300001201
0.1325
F300001233
0.4146
F300000773
0.1458
F300001382
0.2182
0.5211
F300001211
0.1757
0.4682
F300001284
0.1592
F300001287

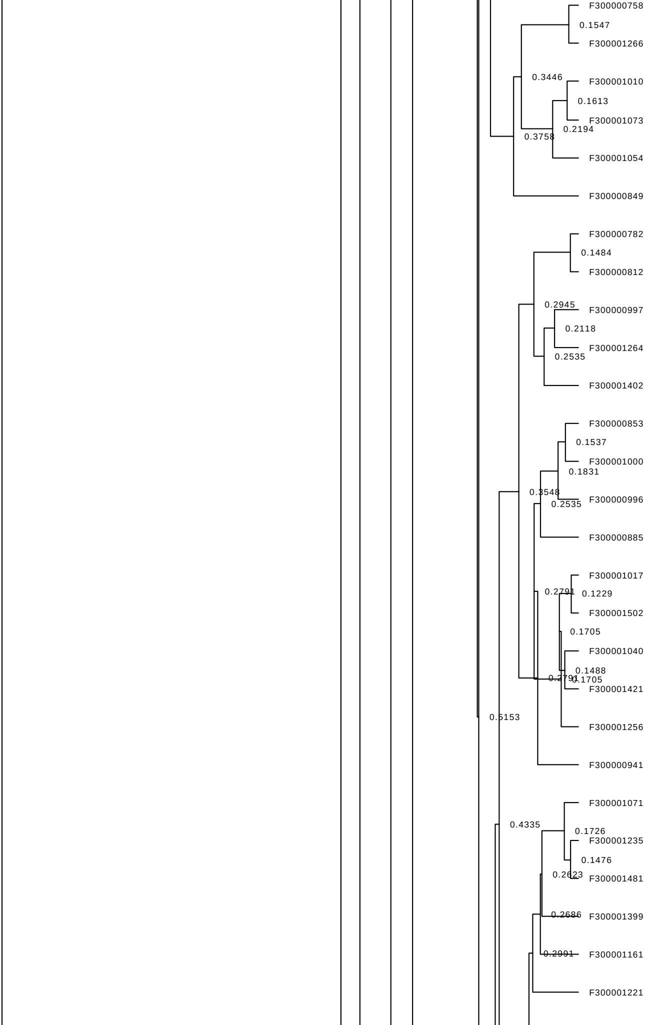
F300000758
0.1547
F300001266
0.3446
F300001010
0.1613
F300001073
0.2194
0.3758
F300001054
F300000849
F300000782
0.1484
F300000812
0.2945
F300000997
0.2118
F300001264
0.2535
F300001402
F300000853
0.1537
F300001000
0.1831
0.3548
F300000996
0.2535
F300000885
F300001017
0.2791
0.1229
F300001502
0.1705
F300001040
0.1488
0.2791
0.1705
F300001421
0.5153
F300001256
F300000941
F300001071
0.4335
0.1726
F300001235
0.1476
0.2623
F300001481
0.2686
F300001399
0.2991
F300001161
F300001221

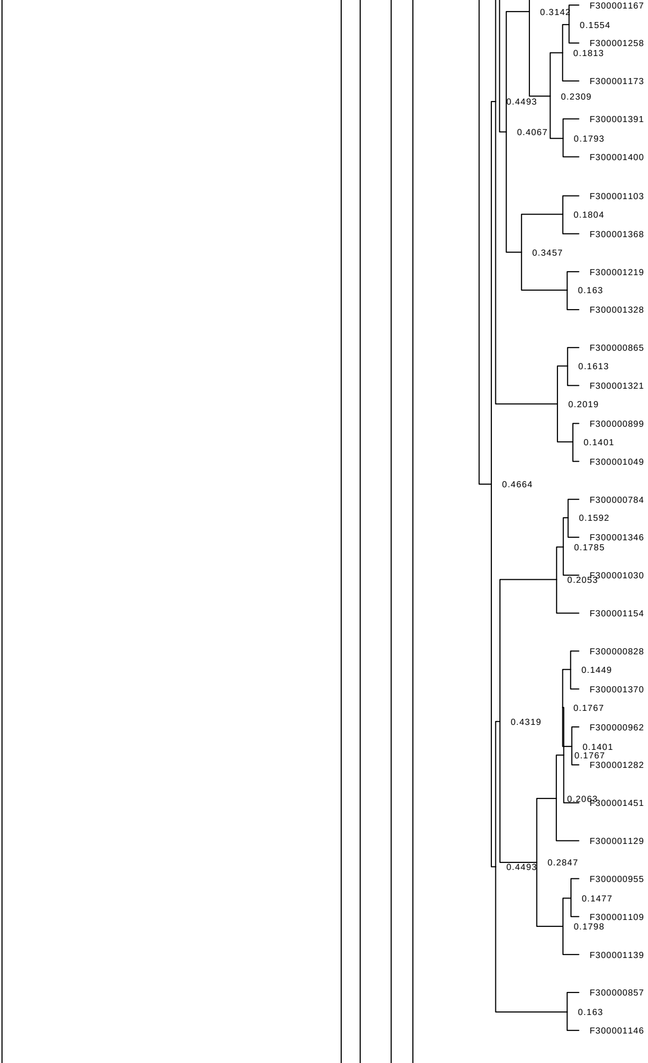

F300001167
0.3142
0.1554
F300001258
0.1813
F300001173
0.2309
0.4493
F300001391
0.4067
0.1793
F300001400
F300001103
0.1804
F300001368
0.3457
F300001219
0.163
F300001328
F300000865
0.1613
F300001321
0.2019
F300000899
0.1401
F300001049
0.4664
F300000784
0.1592
F300001346
0.1785
F300001030
0.2053
F300001154
F300000828
0.1449
F300001370
0.1767
0.4319
F300000962
0.1401
0.1767
F300001282
0.2063
F300001451
F300001129
0.2847
0.4493
F300000955
0.1477
F300001109
0.1798
F300001139
F300000857
0.163
F300001146

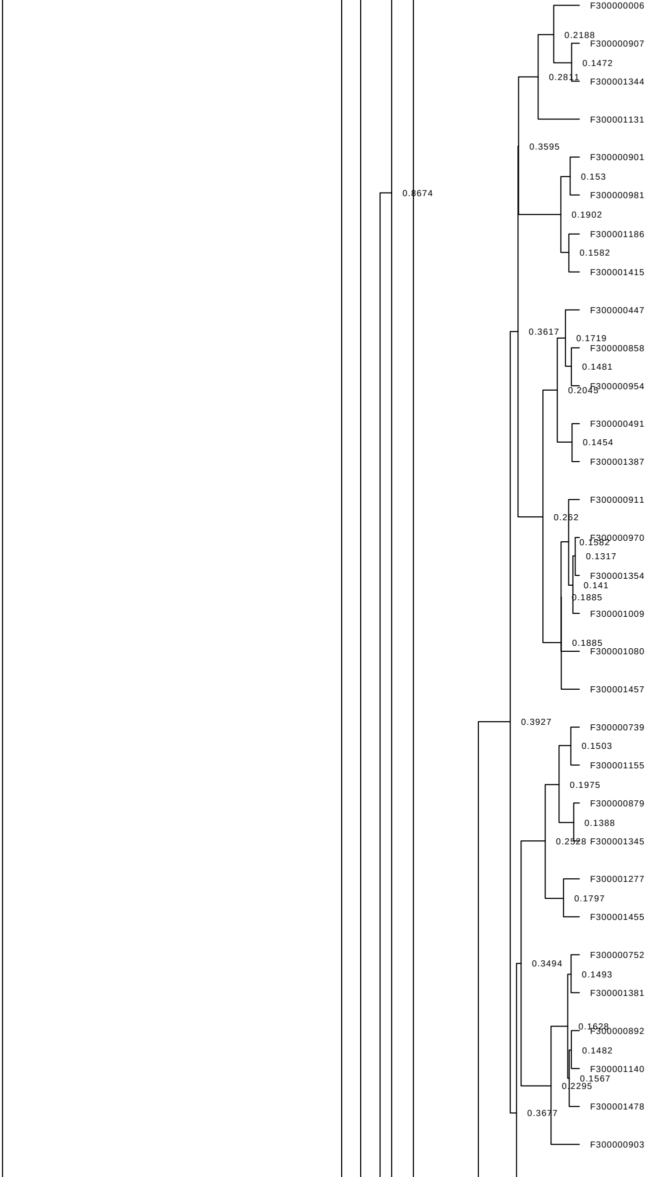

F300000006
0.2188
F300000907
0.1472
0.2811
F300001344
F300001131
0.3595
F300000901
0.153
0.8674
F300000981
0.1902
F300001186
0.1582
F300001415
F300000447
0.3617
0.1719
F300000858
0.1481
F300000954
0.2045
F300000491
0.1454
F300001387
F300000911
0.262
F300000970
0.1582
0.1317
F300001354
0.141
0.1885
F300001009
0.1885
F300001080
F300001457
0.3927
F300000739
0.1503
F300001155
0.1975
F300000879
0.1388
0.2528
F300001345
F300001277
0.1797
F300001455
F300000752
0.3494
0.1493
F300001381
0.1628
F300000892
0.1482
F300001140
0.1567
0.2295
F300001478
0.3677
F300000903

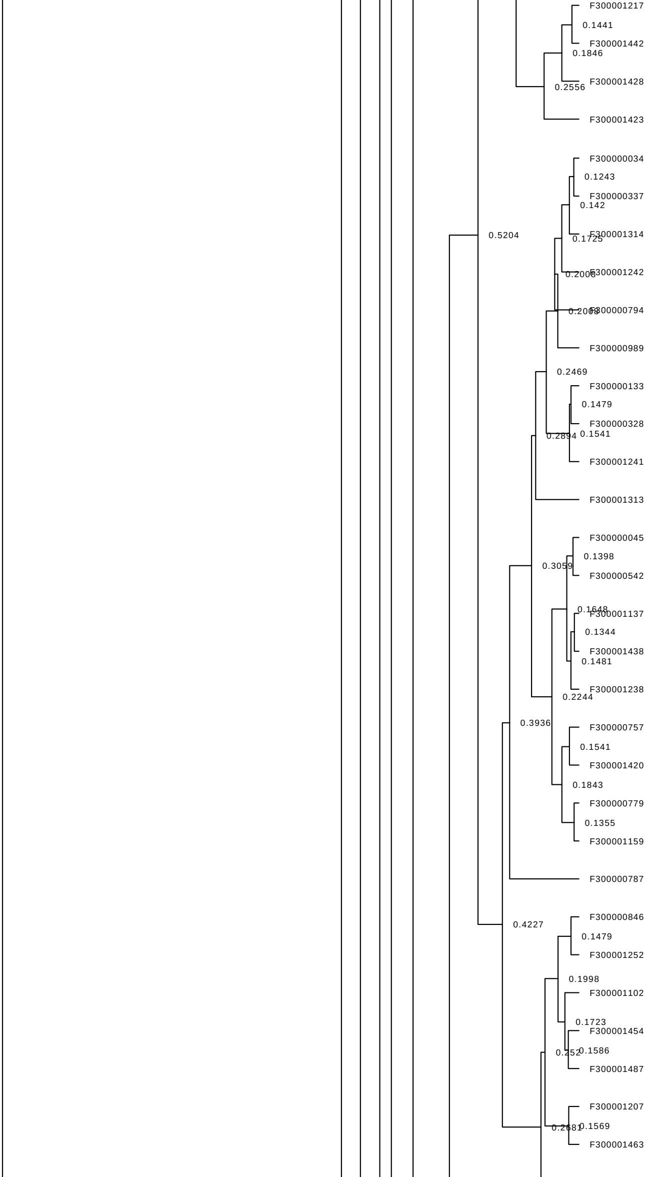
F300001217
0.1441
F300001442
0.1846
F300001428
0.2556
F300001423
F300000034
0.1243
F300000337
0.142
0.5204
F300001314
0.1725
F300001242
0.2008
F300000794
0.2008
F300000989
0.2469
F300000133
0.1479
F300000328
0.2894
0.1541
F300001241
F300001313
F300000045
0.1398
0.3059
F300000542
0.1648
F300001137
0.1344
F300001438
0.1481
F300001238
0.2244
0.3936
F300000757
0.1541
F300001420
0.1843
F300000779
0.1355
F300001159
F300000787
0.4227
F300000846
0.1479
F300001252
0.1998
F300001102
0.1723
F300001454
0.252
0.1586
F300001487
F300001207
0.2681
0.1569
F300001463

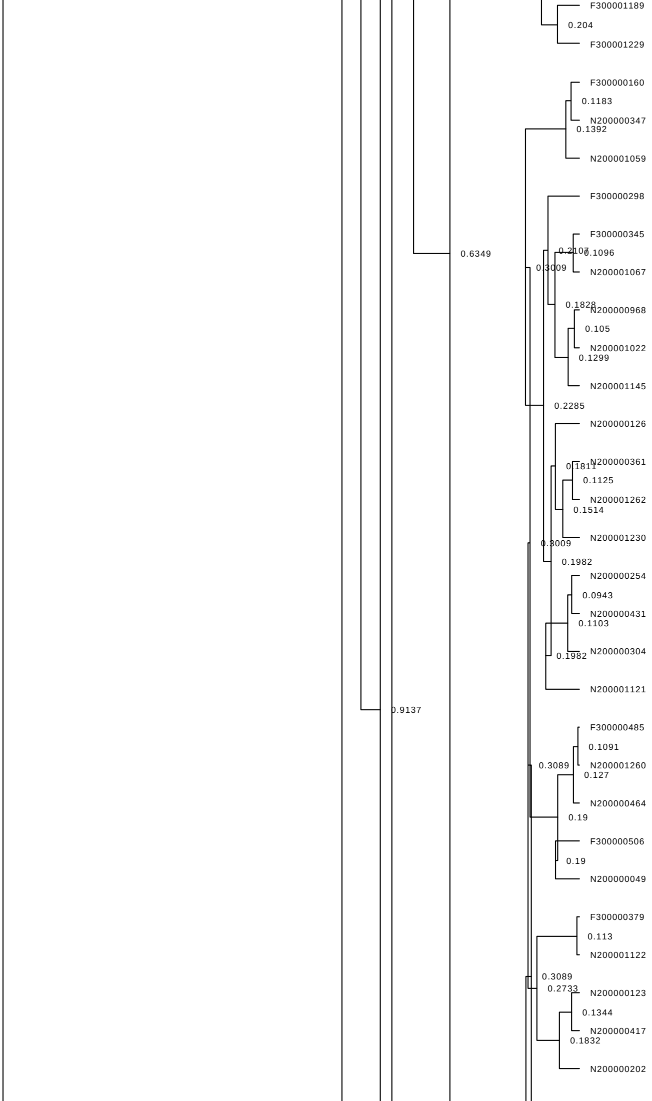
F300001189
0.204
F300001229
F300000160
0.1183
N200000347
0.1392
N200001059
F300000298
F300000345
0.2107
0.1096
0.6349
0.3009
N200001067
0.1828
N200000968
0.105
N200001022
0.1299
N200001145
0.2285
N200000126
N200000361
0.1811
0.1125
N200001262
0.1514
N200001230
0.3009
0.1982
N200000254
0.0943
N200000431
0.1103
N200000304
0.1982
N200001121
0.9137
F300000485
0.1091
0.3089
N200001260
0.127
N200000464
0.19
F300000506
0.19
N200000049
F300000379
0.113
N200001122
0.3089
0.2733
N200000123
0.1344
N200000417
0.1832
N200000202

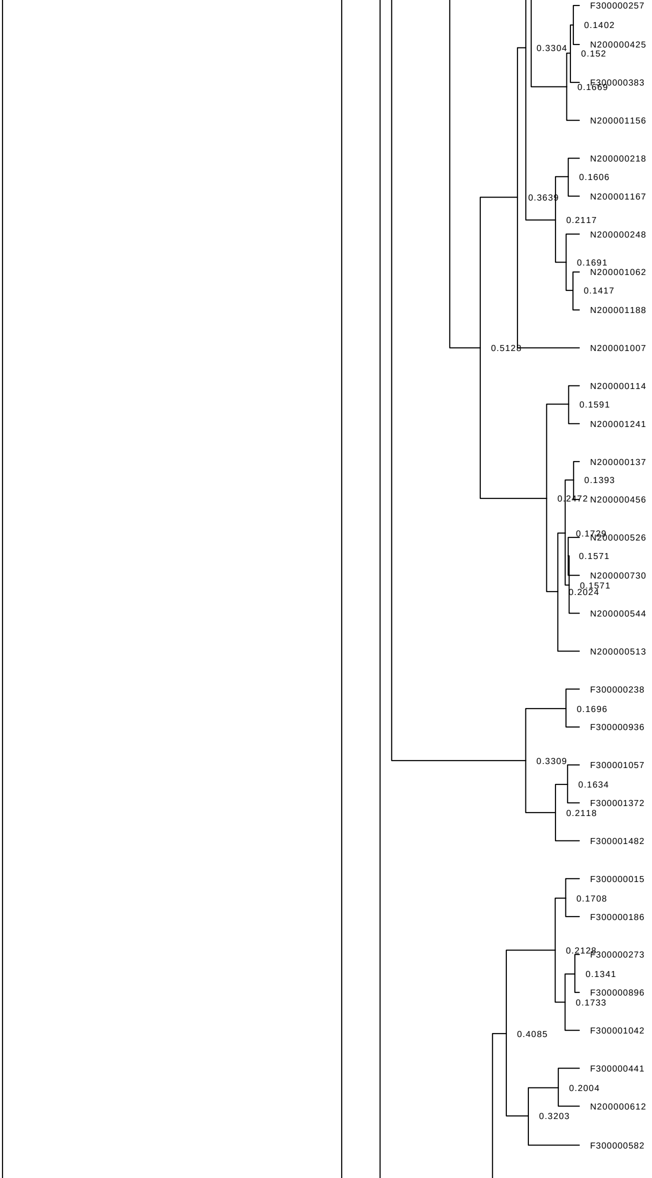
F300000257
0.1402
N200000425
0.3304
0.152
F300000383
0.1669
N200001156
N200000218
0.1606
N200001167
0.3639
0.2117
N200000248
0.1691
N200001062
0.1417
N200001188
0.5128
N200001007
N200000114
0.1591
N200001241
N200000137
0.1393
0.2472
N200000456
0.1729
N200000526
0.1571
N200000730
0.1571
0.2024
N200000544
N200000513
F300000238
0.1696
F300000936
0.3309
F300001057
0.1634
F300001372
0.2118
F300001482
F300000015
0.1708
F300000186
0.2128
F300000273
0.1341
F300000896
0.1733
F300001042
0.4085
F300000441
0.2004
N200000612
0.3203
F300000582

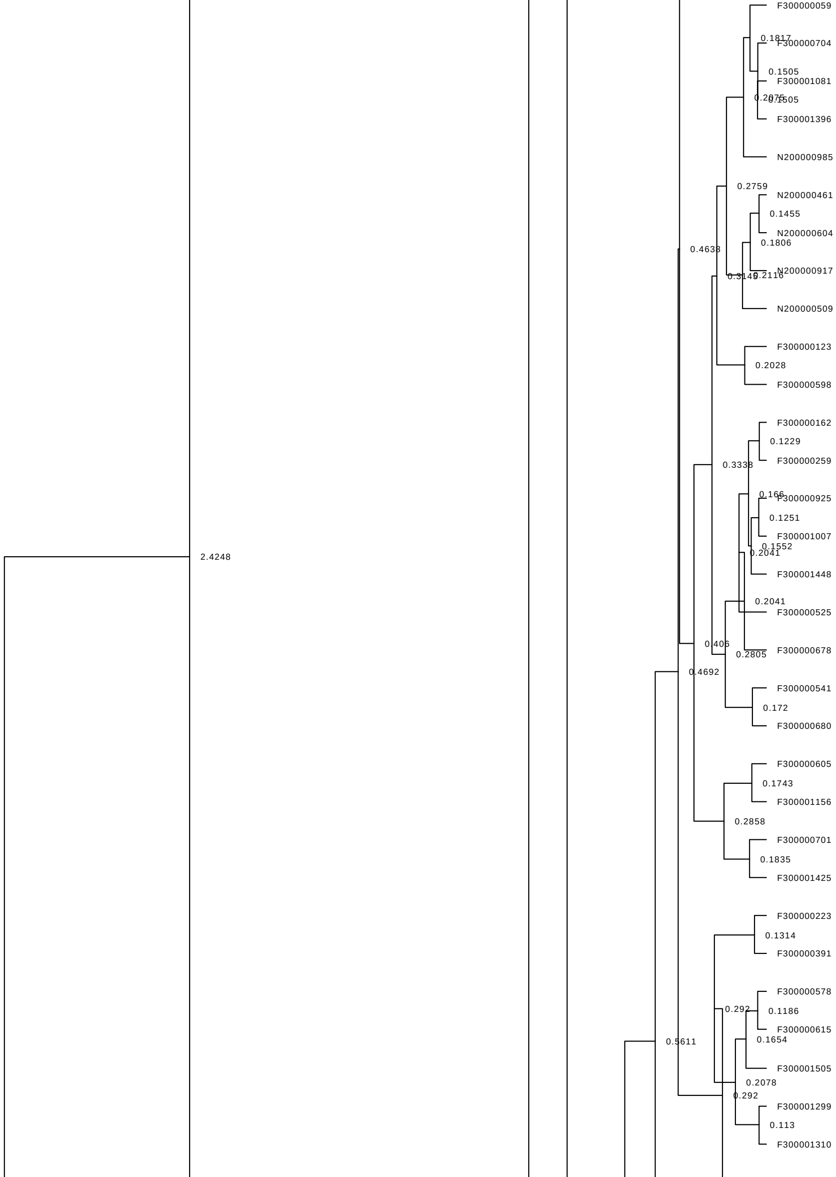
F300000059
0.1817
F300000704
0.1505
F300001081
0.2075
0.1505
F300001396
N200000985
0.2759
N200000461
0.1455
N200000604
0.1806
0.4638
N200000917
0.3149
0.2116
N200000509
F300000123
0.2028
F300000598
F300000162
0.1229
F300000259
0.3338
0.166
F300000925
0.1251
F300001007
0.1552
0.2041
F300001448
0.2041
F300000525
0.406
F300000678
0.2805
0.4692
F300000541
0.172
F300000680
F300000605
0.1743
F300001156
0.2858
F300000701
0.1835
F300001425
F300000223
0.1314
F300000391
F300000578
0.292
0.1186
F300000615
0.1654
0.5611
F300001505
0.2078
0.292
F300001299
0.113
F300001310
2.4248

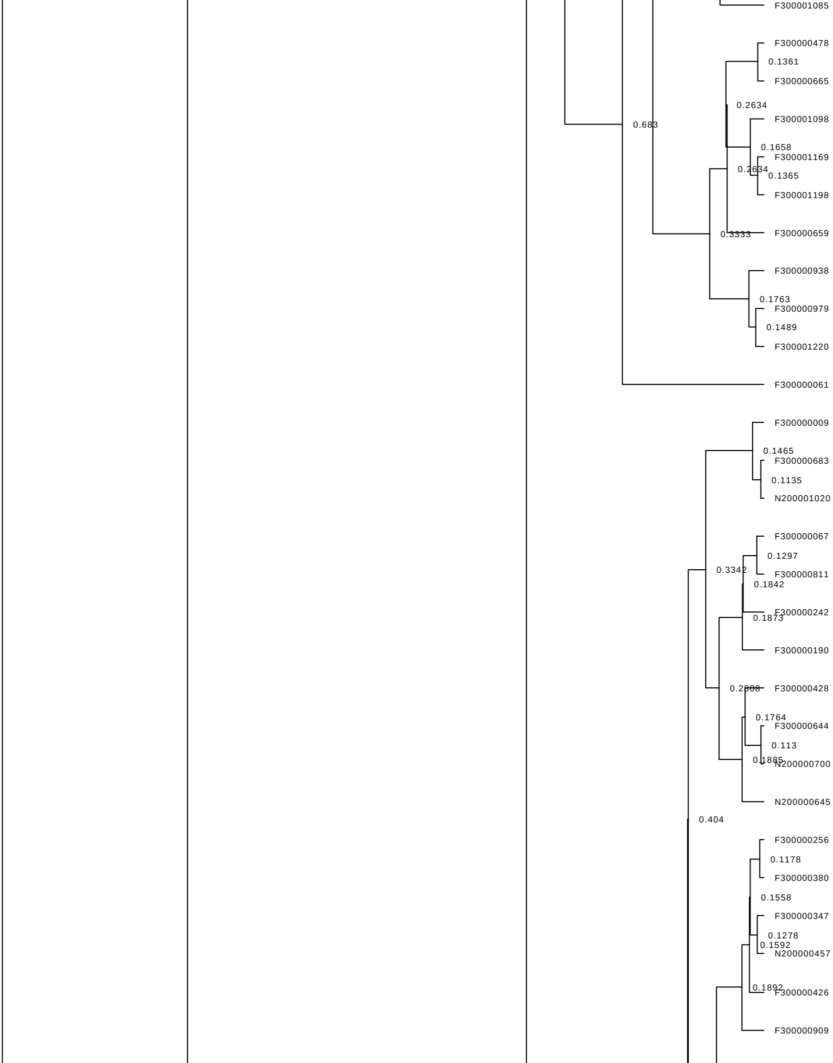
F300001085
F300000478
0.1361
F300000665
0.2634
F300001098
0.683
0.1658
F300001169
0.2634
0.1365
F300001198
0.3333
F300000659
F300000938
0.1763
F300000979
0.1489
F300001220
F300000061
F300000009
0.1465
F300000683
0.1135
N200001020
F300000067
0.1297
0.3342
F300000811
0.1842
F300000242
0.1873
F300000190
0.2808
F300000428
0.1764
F300000644
0.113
0.1885
N200000700
N200000645
0.404
F300000256
0.1178
F300000380
0.1558
F300000347
0.1278
0.1592
N200000457
0.1892
F300000426
F300000909

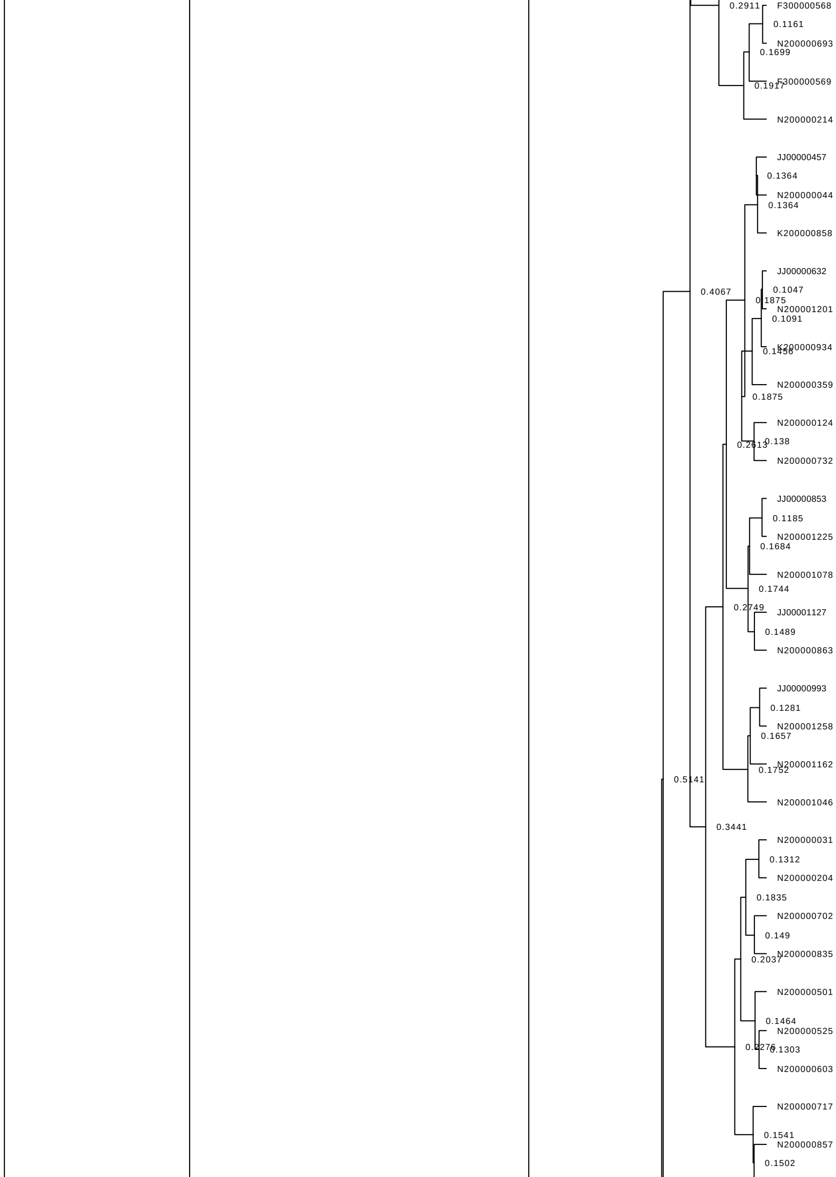
F300000568
0.2911
0.1161
N200000693
0.1699
F300000569
0.1917
N200000214
JJ00000457
0.1364
N200000044
0.1364
K200000858
JJ00000632
0.1047
0.4067
0.1875
N200001201
0.1091
K200000934
0.1456
N200000359
0.1875
N200000124
0.138
0.2613
N200000732
JJ00000853
0.1185
N200001225
0.1684
N200001078
0.1744
0.2749
JJ00001127
0.1489
N200000863
JJ00000993
0.1281
N200001258
0.1657
N200001162
0.1752
0.5141
N200001046
0.3441
N200000031
0.1312
N200000204
0.1835
N200000702
0.149
N200000835
0.2037
N200000501
0.1464
N200000525
0.2276
0.1303
N200000603
N200000717
0.1541
N200000857
0.1502

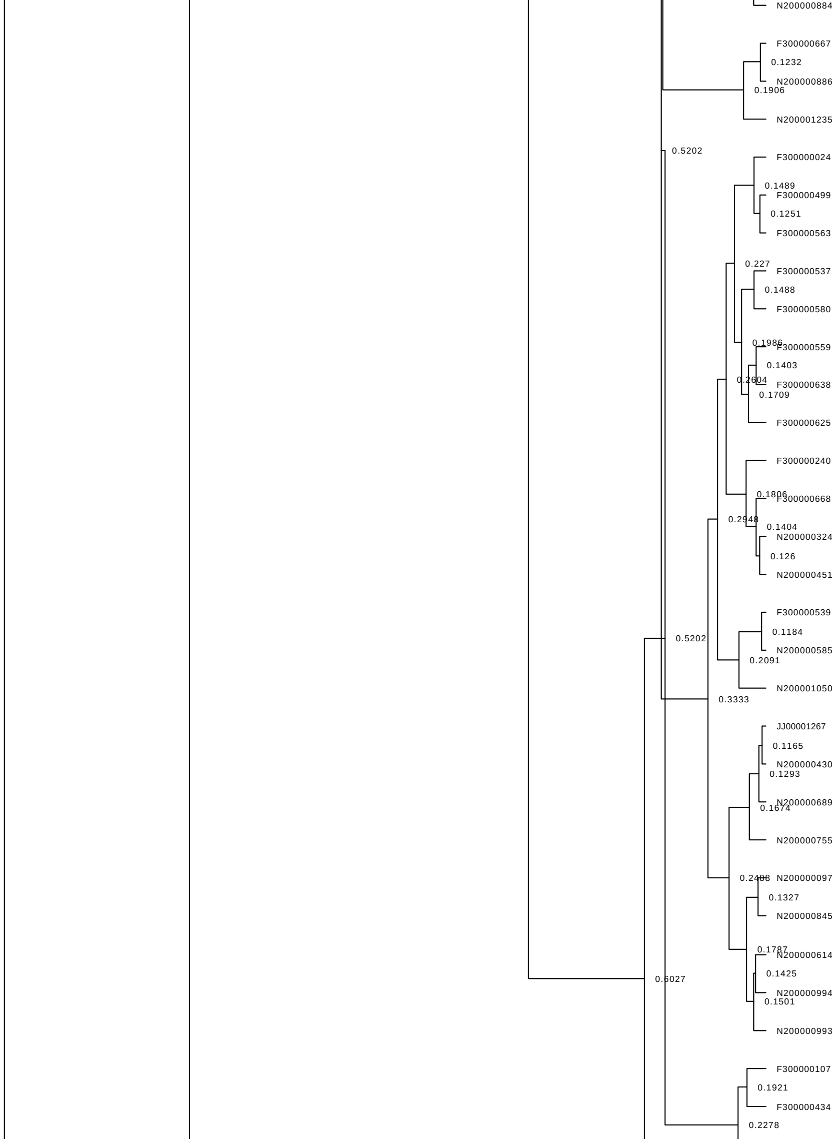
N200000884
F300000667
0.1232
N200000886
0.1906
N200001235
0.5202
F300000024
0.1489
F300000499
0.1251
F300000563
0.227
F300000537
0.1488
F300000580
0.1986
F300000559
0.1403
0.2604
F300000638
0.1709
F300000625
F300000240
0.1806
F300000668
0.2948
0.1404
N200000324
0.126
N200000451
F300000539
0.1184
0.5202
N200000585
0.2091
N200001050
0.3333
JJ00001267
0.1165
N200000430
0.1293
N200000689
0.1674
N200000755
0.2488
N200000097
0.1327
N200000845
0.1787
N200000614
0.1425
0.6027
N200000994
0.1501
N200000993
F300000107
0.1921
F300000434
0.2278

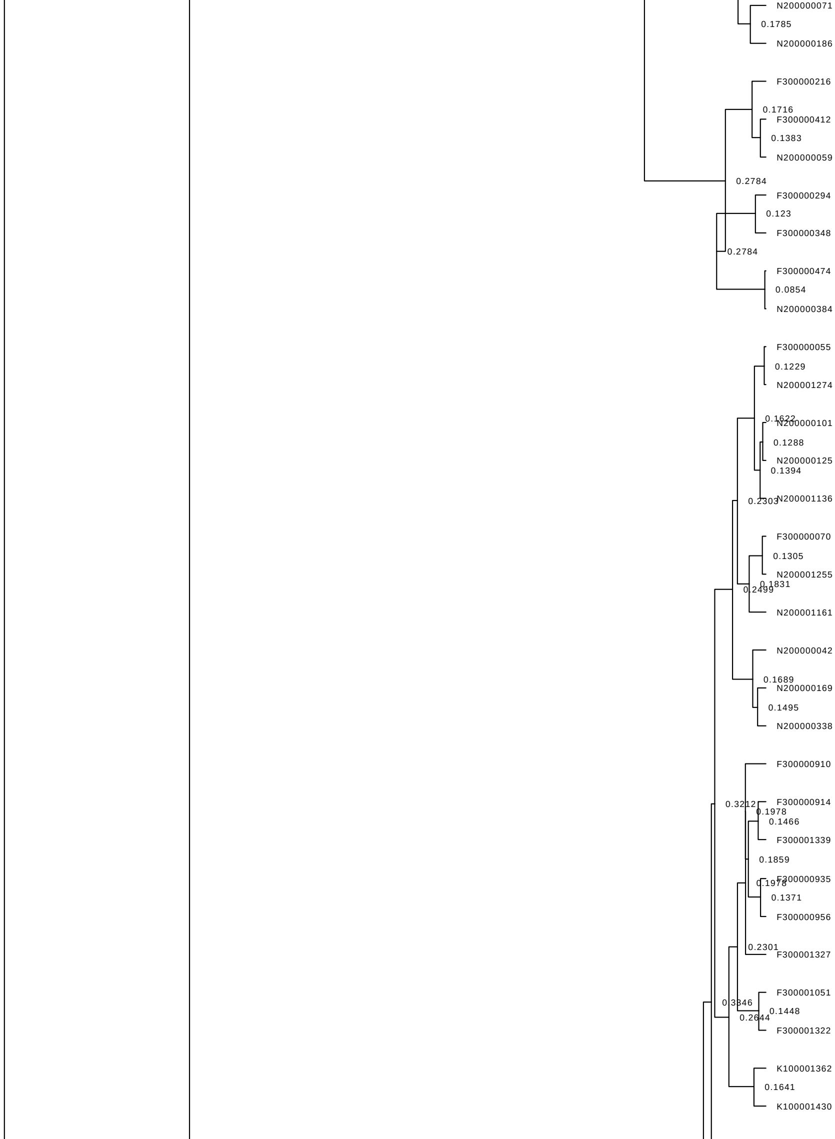

N200000071
0.1785
N200000186
F300000216
0.1716
F300000412
0.1383
N200000059
0.2784
F300000294
0.123
F300000348
0.2784
F300000474
0.0854
N200000384
F300000055
0.1229
N200001274
0.1622
N200000101
0.1288
N200000125
0.1394
0.2303
N200001136
F300000070
0.1305
N200001255
0.1831
0.2499
N200001161
N200000042
0.1689
N200000169
0.1495
N200000338
F300000910
0.3212
F300000914
0.1978
0.1466
F300001339
0.1859
F300000935
0.1978
0.1371
F300000956
0.2301
F300001327
F300001051
0.3346
0.1448
0.2644
F300001322
K100001362
0.1641
K100001430

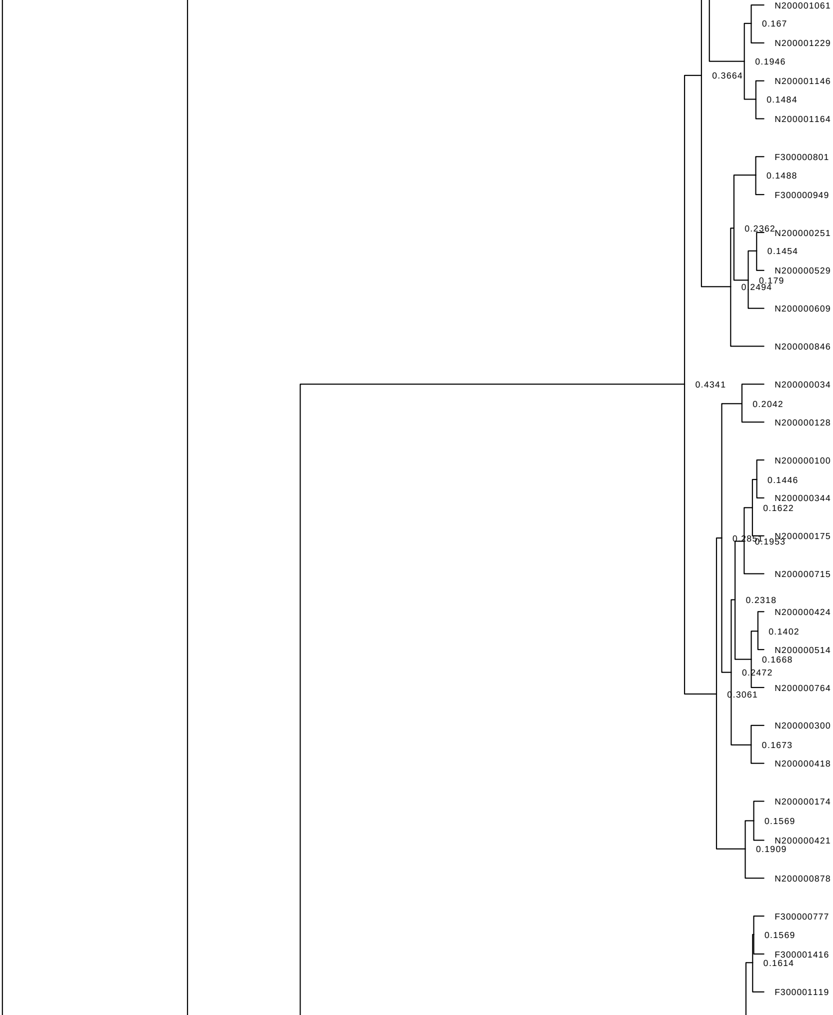
N200001061
0.167
N200001229
0.1946
0.3664
N200001146
0.1484
N200001164
F300000801
0.1488
F300000949
0.2362
N200000251
0.1454
N200000529
0.179
0.2494
N200000609
N200000846
0.4341
N200000034
0.2042
N200000128
N200000100
0.1446
N200000344
0.1622
0.2851
N200000175
0.1953
N200000715
0.2318
N200000424
0.1402
N200000514
0.1668
0.2472
N200000764
0.3061
N200000300
0.1673
N200000418
N200000174
0.1569
N200000421
0.1909
N200000878
F300000777
0.1569
F300001416
0.1614
F300001119

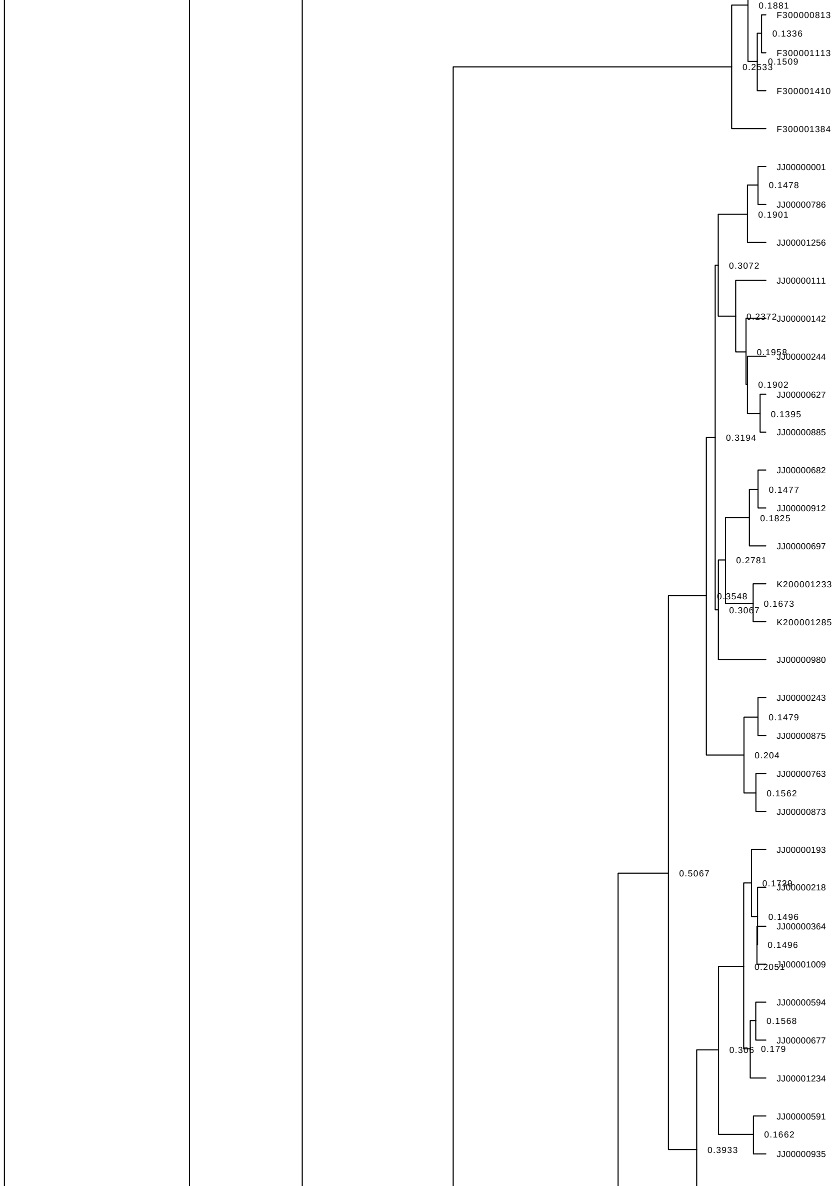
0.1881
F300000813
0.1336
F300001113
0.1509
0.2533
F300001410
F300001384
JJ00000001
0.1478
JJ00000786
0.1901
JJ00001256
0.3072
JJ00000111
0.2372
JJ00000142
0.1958
JJ00000244
0.1902
JJ00000627
0.1395
JJ00000885
0.3194
JJ00000682
0.1477
JJ00000912
0.1825
JJ00000697
0.2781
K200001233
0.3548
0.1673
0.3067
K200001285
JJ00000980
JJ00000243
0.1479
JJ00000875
0.204
JJ00000763
0.1562
JJ00000873
JJ00000193
0.5067
0.1739
JJ00000218
0.1496
JJ00000364
0.1496
JJ00001009
0.2051
JJ00000594
0.1568
JJ00000677
0.306
0.179
JJ00001234
JJ00000591
0.1662
0.3933
JJ00000935

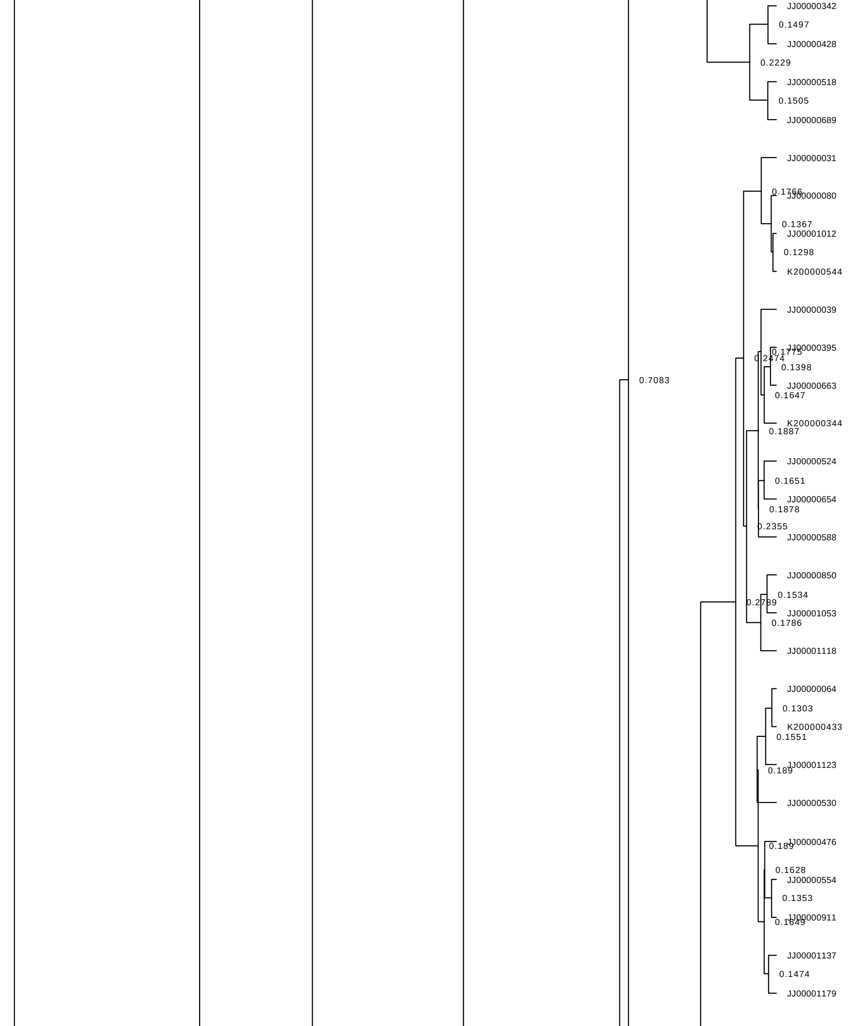
JJ00000342
0.1497
JJ00000428
0.2229
JJ00000518
0.1505
JJ00000689
JJ00000031
0.1766
JJ00000080
0.1367
JJ00001012
0.1298
K200000544
JJ00000039
JJ00000395
0.1775
0.2474
0.1398
0.7083
JJ00000663
0.1647
K200000344
0.1887
JJ00000524
0.1651
JJ00000654
0.1878
0.2355
JJ00000588
JJ00000850
0.1534
0.2789
JJ00001053
0.1786
JJ00001118
JJ00000064
0.1303
K200000433
0.1551
JJ00001123
0.189
JJ00000530
JJ00000476
0.189
0.1628
JJ00000554
0.1353
JJ00000911
0.1649
JJ00001137
0.1474
JJ00001179

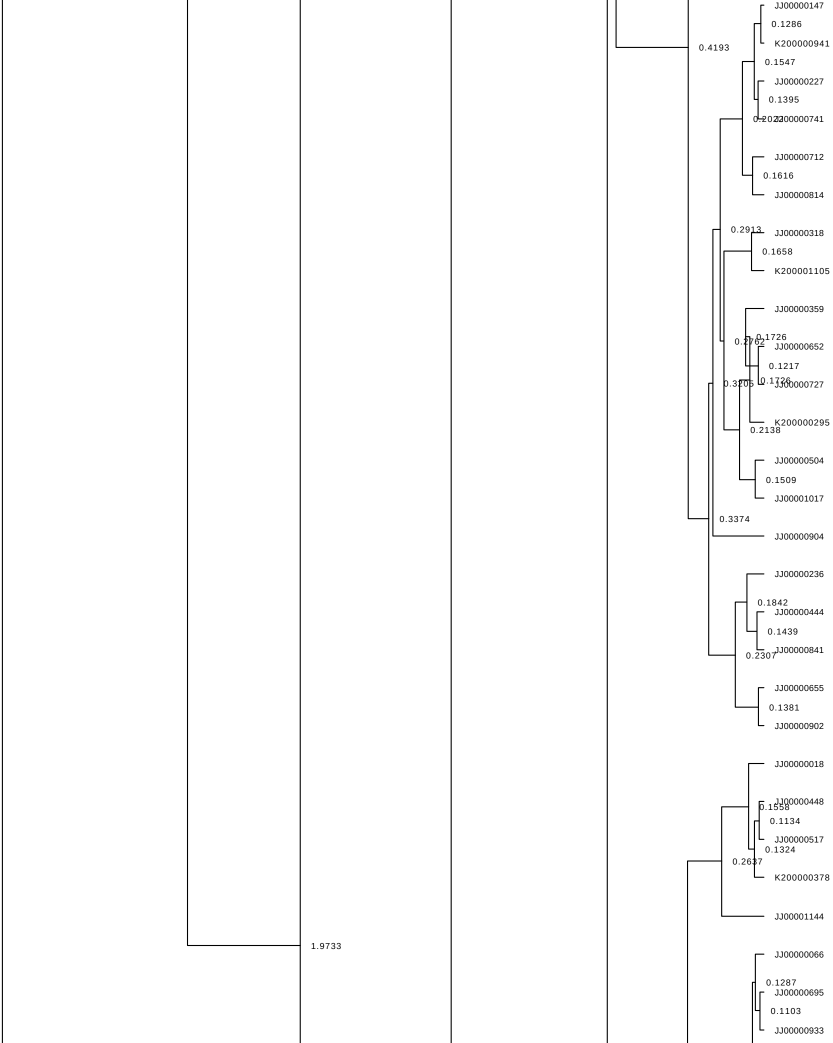

JJ00000147
0.1286
K200000941
0.4193
0.1547
JJ00000227
0.1395
0.2022
JJ00000712
0.1616
JJ00000814
0.2913
JJ00000318
0.1658
K200001105
JJ00000359
0.1726
0.2762
JJ00000652
0.1217
0.3205
0.1726
JJ00000727
K200000295
0.2138
JJ00000504
0.1509
JJ00001017
0.3374
JJ00000904
JJ00000236
0.1842
JJ00000444
0.1439
JJ00000841
0.2307
JJ00000655
0.1381
JJ00000902
JJ00000018
JJ00000448
0.1558
0.1134
JJ00000517
0.1324
0.2637
K200000378
JJ00001144
1.9733
JJ00000066
0.1287
JJ00000695
0.1103
JJ00000933

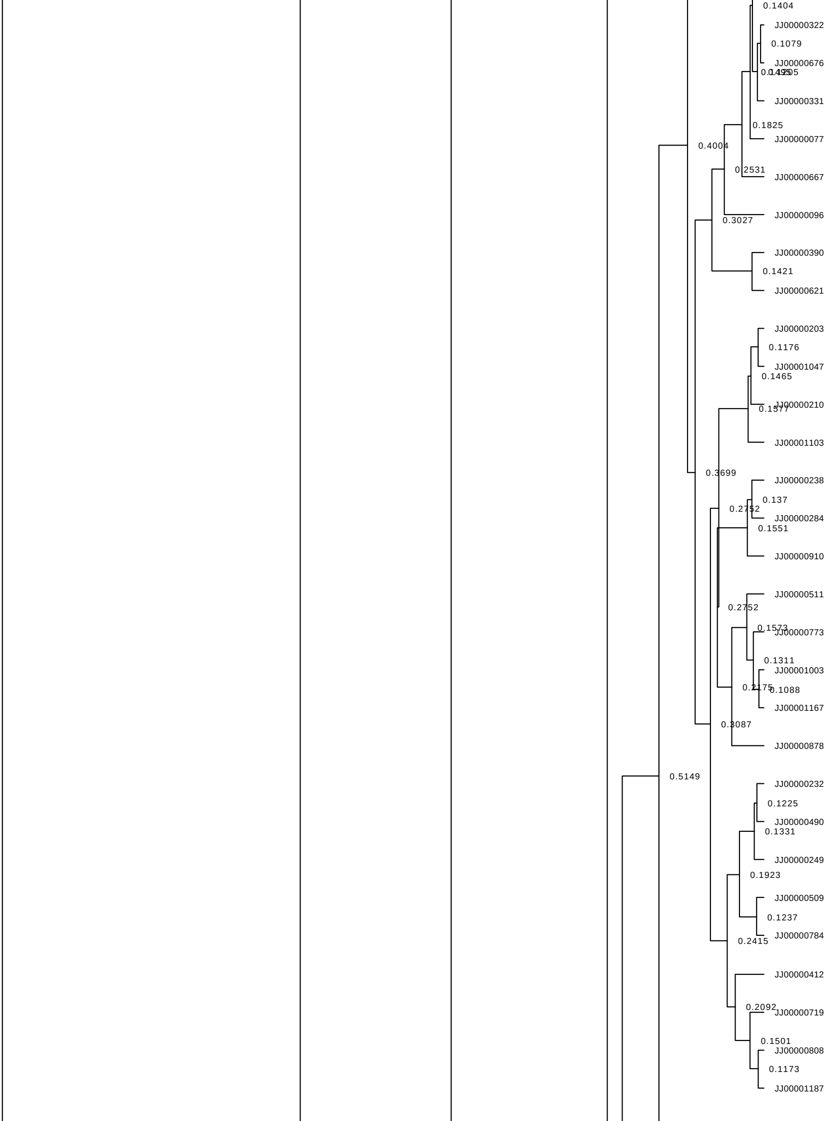
0.1404
JJ00000322
0.1079
JJ00000676
JJ00000331
0.1825
JJ00000077
0.4004
0.2531
JJ00000667
JJ00000096
0.3027
JJ00000390
0.1421
JJ00000621
JJ00000203
0.1176
JJ00001047
0.1465
JJ00000210
0.1577
JJ00001103
0.3699
JJ00000238
0.137
0.2752
JJ00000284
0.1551
JJ00000910
JJ00000511
0.2752
0.1573
JJ00000773
0.1311
JJ00001003
0.2175
0.1088
JJ00001167
0.3087
JJ00000878
0.5149
JJ00000232
0.1225
JJ00000490
0.1331
JJ00000249
0.1923
JJ00000509
0.1237
JJ00000784
0.2415
JJ00000412
0.2092
JJ00000719
0.1501
JJ00000808
0.1173
JJ00001187

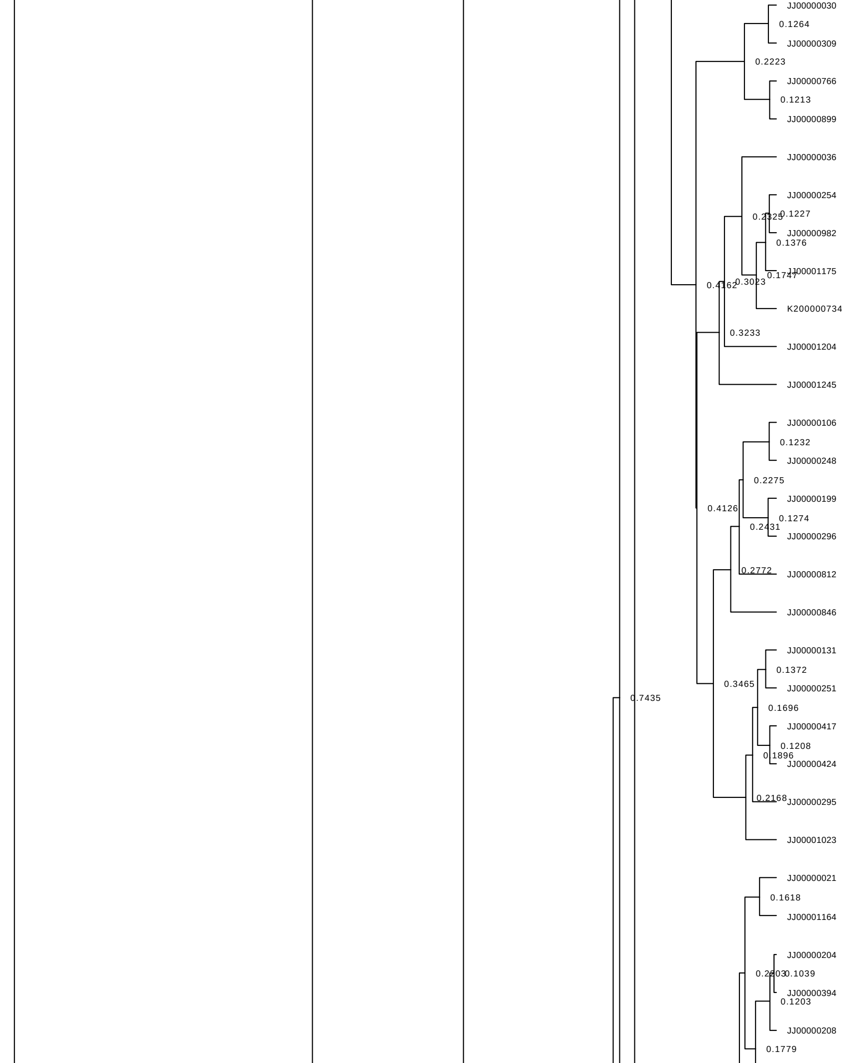

JJ00000030
0.1264
JJ00000309
0.2223
JJ00000766
0.1213
JJ00000899
JJ00000036
JJ00000254
0.2325
0.1227
JJ00000982
0.1376
JJ00001175
0.1747
0.4162
0.3023
K200000734
0.3233
JJ00001204
JJ00001245
JJ00000106
0.1232
JJ00000248
0.2275
JJ00000199
0.4126
0.1274
0.2431
JJ00000296
0.2772
JJ00000812
JJ00000846
JJ00000131
0.1372
0.3465
JJ00000251
0.7435
0.1696
JJ00000417
0.1208
0.1896
JJ00000424
0.2168
JJ00000295
JJ00001023
JJ00000021
0.1618
JJ00001164
JJ00000204
0.2203
0.1039
JJ00000394
0.1203
JJ00000208
0.1779

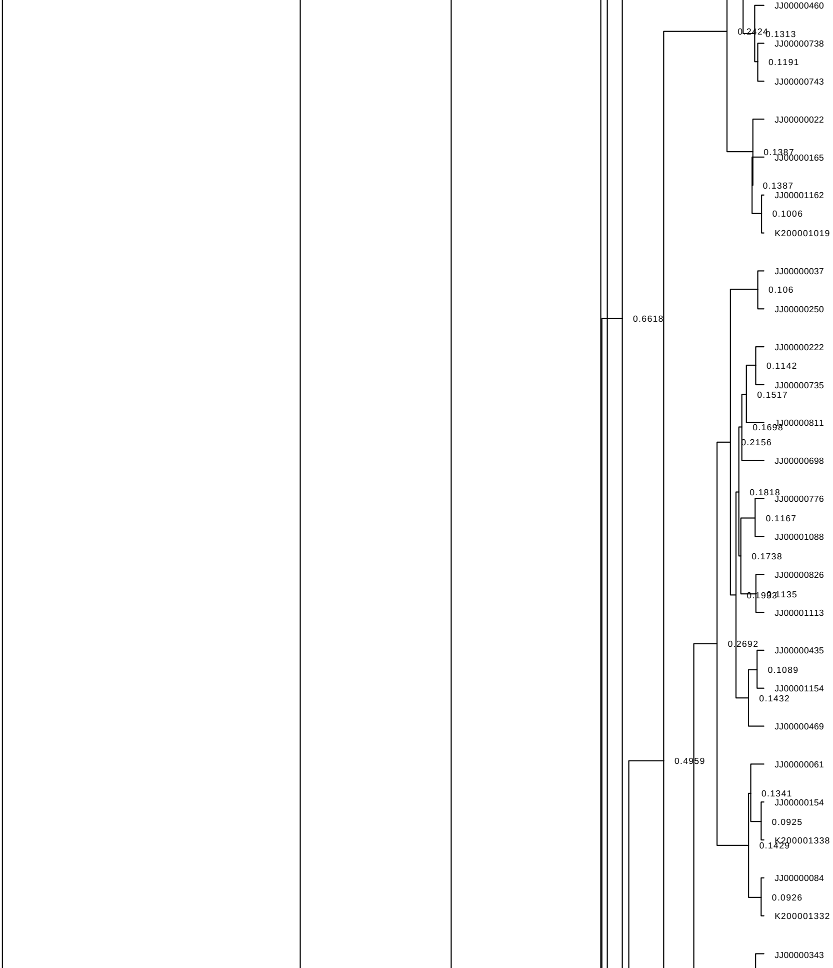
JJ00000460
0.2424
0.1313
JJ00000738
0.1191
JJ00000743
JJ00000022
0.1387
JJ00000165
0.1387
JJ00001162
0.1006
K200001019
JJ00000037
0.106
JJ00000250
0.6618
JJ00000222
0.1142
JJ00000735
0.1517
JJ00000811
0.1698
0.2156
JJ00000698
0.1818
JJ00000776
0.1167
JJ00001088
0.1738
JJ00000826
0.1993
0.1135
JJ00001113
0.2692
JJ00000435
0.1089
JJ00001154
0.1432
JJ00000469
0.4959
JJ00000061
0.1341
JJ00000154
0.0925
K200001338
0.1429
JJ00000084
0.0926
K200001332
JJ00000343

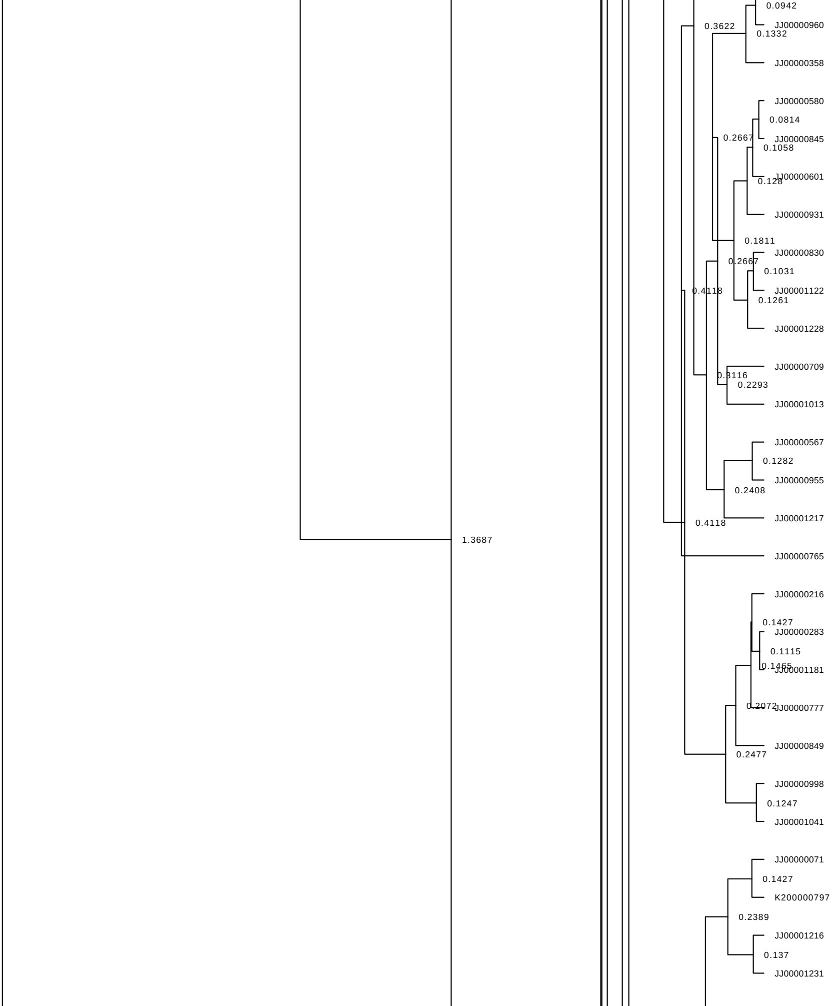
0.0942
JJ00000960
0.3622
0.1332
JJ00000358
JJ00000580
0.0814
0.2667
JJ00000845
0.1058
JJ00000601
0.128
JJ00000931
0.1811
JJ00000830
0.2667
0.1031
0.4118
JJ00001122
0.1261
JJ00001228
JJ00000709
0.3116
0.2293
JJ00001013
JJ00000567
0.1282
JJ00000955
0.2408
JJ00001217
0.4118
1.3687
JJ00000765
JJ00000216
0.1427
JJ00000283
0.1115
0.1465
JJ00001181
0.2072
JJ00000777
JJ00000849
0.2477
JJ00000998
0.1247
JJ00001041
JJ00000071
0.1427
K200000797
0.2389
JJ00001216
0.137
JJ00001231

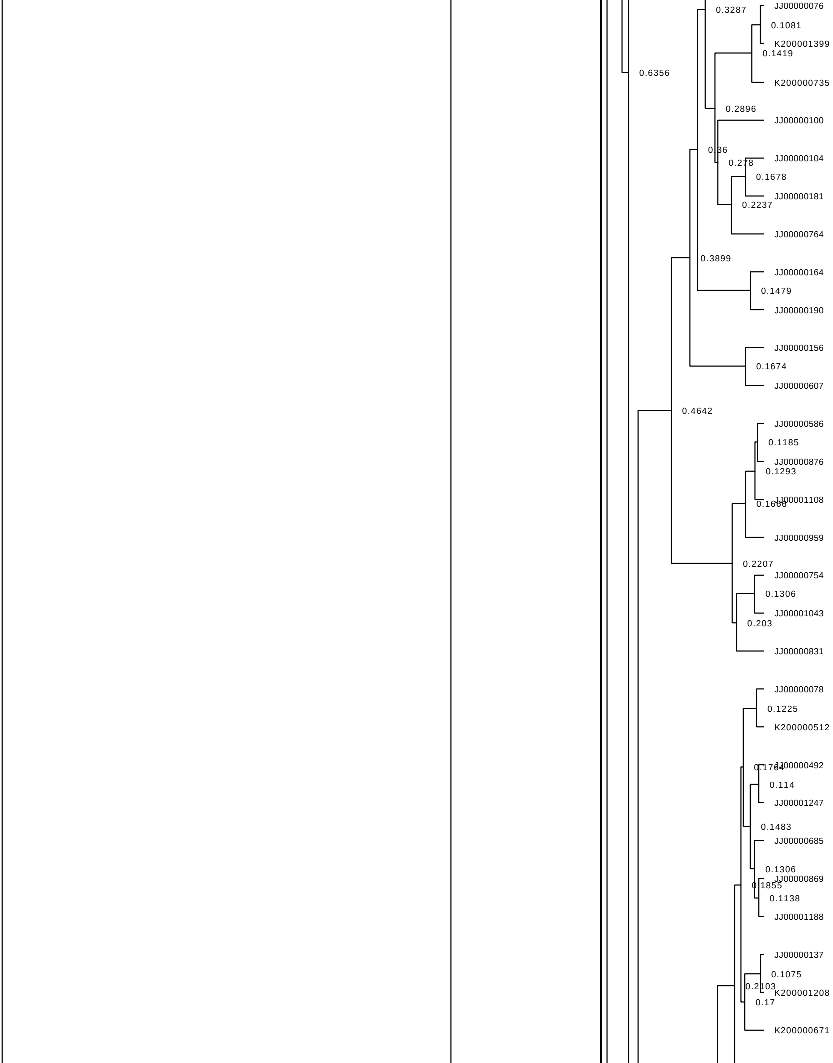
0.3287
JJ00000076
0.1081
K200001399
0.1419
0.6356
K200000735
0.2896
JJ00000100
0.36
JJ00000104
0.278
0.1678
JJ00000181
0.2237
JJ00000764
0.3899
JJ00000164
0.1479
JJ00000190
JJ00000156
0.1674
JJ00000607
0.4642
JJ00000586
0.1185
JJ00000876
0.1293
JJ00001108
0.1666
JJ00000959
0.2207
JJ00000754
0.1306
JJ00001043
0.203
JJ00000831
JJ00000078
0.1225
K200000512
JJ00000492
0.1764
0.114
JJ00001247
0.1483
JJ00000685
0.1306
JJ00000869
0.1855
0.1138
JJ00001188
JJ00000137
0.1075
0.2103
K200001208
0.17
K200000671

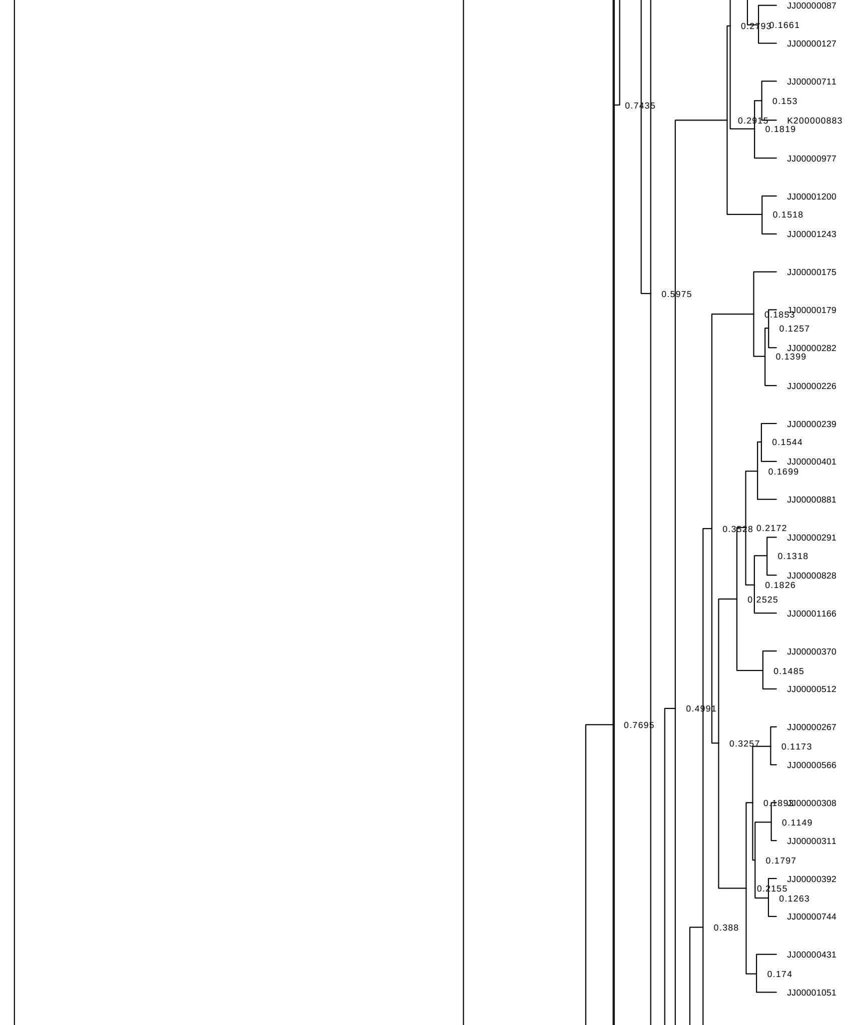
JJ00000087
0.2793
0.1661
JJ00000127
JJ00000711
0.153
0.7435
0.2915
K200000883
0.1819
JJ00000977
JJ00001200
0.1518
JJ00001243
JJ00000175
0.5975
JJ00000179
0.1853
0.1257
JJ00000282
0.1399
JJ00000226
JJ00000239
0.1544
JJ00000401
0.1699
JJ00000881
0.3528
0.2172
JJ00000291
0.1318
JJ00000828
0.1826
0.2525
JJ00001166
JJ00000370
0.1485
JJ00000512
0.4991
0.7695
JJ00000267
0.3257
0.1173
JJ00000566
0.1898
JJ00000308
0.1149
JJ00000311
0.1797
JJ00000392
0.2155
0.1263
JJ00000744
0.388
JJ00000431
0.174
JJ00001051

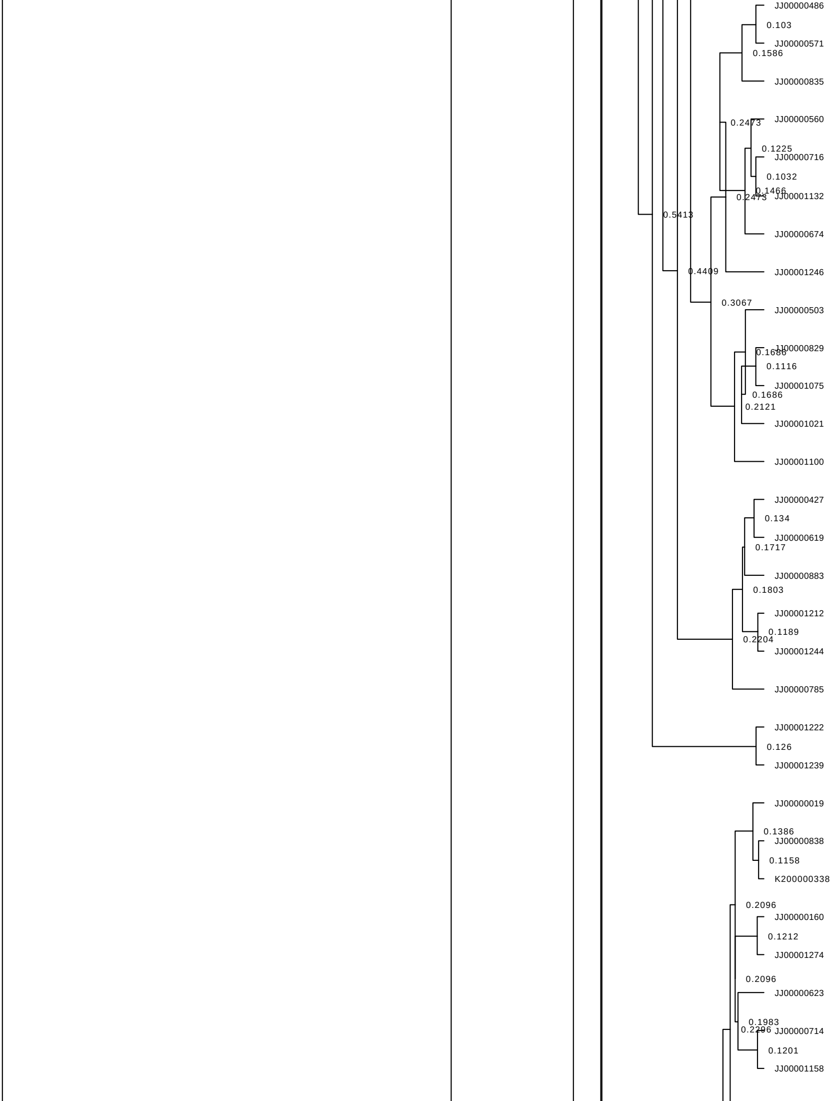
JJ00000486
0.103
JJ00000571
0.1586
JJ00000835
JJ00000560
0.2473
0.1225
JJ00000716
0.1032
0.1466
0.2473
JJ00001132
0.5413
JJ00000674
JJ00001246
0.4409
0.3067
JJ00000503
JJ00000829
0.1686
0.1116
JJ00001075
0.1686
0.2121
JJ00001021
JJ00001100
JJ00000427
0.134
JJ00000619
0.1717
JJ00000883
0.1803
JJ00001212
0.1189
0.2204
JJ00001244
JJ00000785
JJ00001222
0.126
JJ00001239
JJ00000019
0.1386
JJ00000838
0.1158
K200000338
0.2096
JJ00000160
0.1212
JJ00001274
0.2096
JJ00000623
0.1983
0.2296
JJ00000714
0.1201
JJ00001158

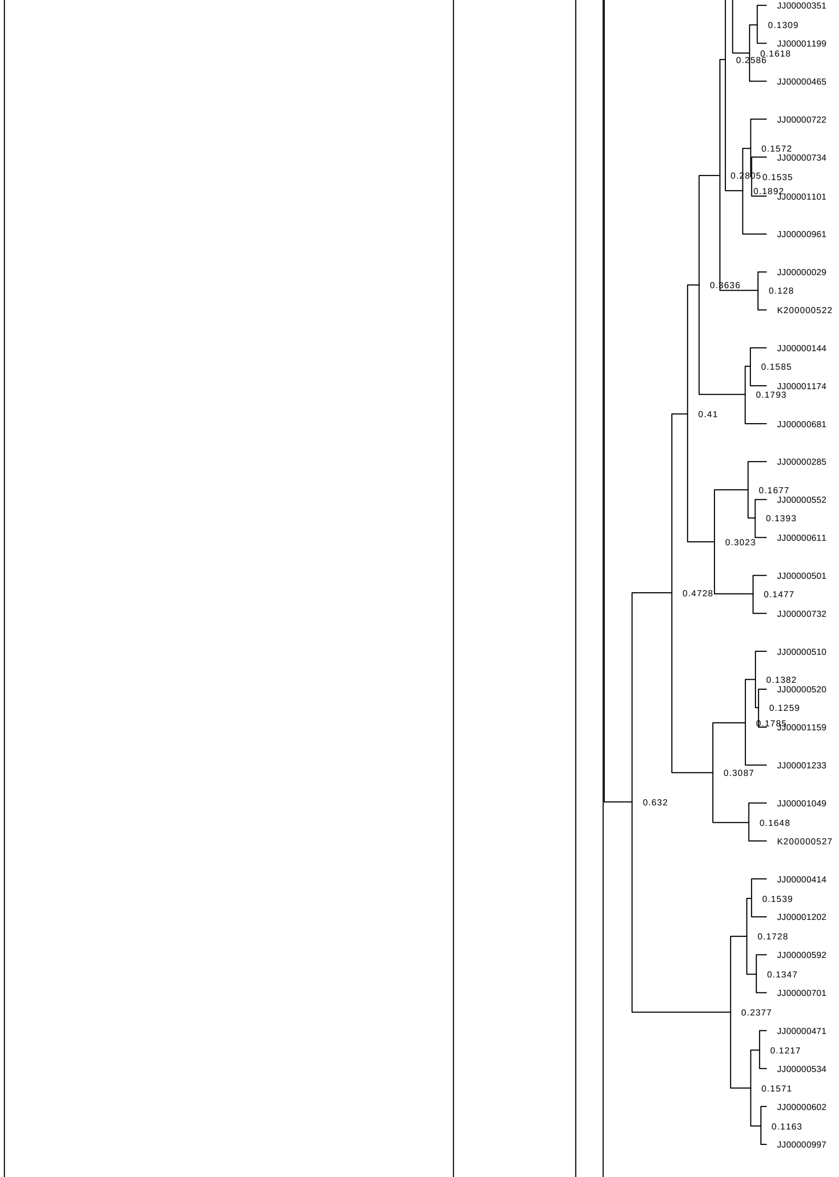
JJ00000351
0.1309
JJ00001199
0.1618
0.2586
JJ00000465
JJ00000722
0.1572
JJ00000734
0.2805
0.1535
0.1892
JJ00001101
JJ00000961
JJ00000029
0.3636
0.128
K200000522
JJ00000144
0.1585
JJ00001174
0.1793
0.41
JJ00000681
JJ00000285
0.1677
JJ00000552
0.1393
JJ00000611
0.3023
JJ00000501
0.4728
0.1477
JJ00000732
JJ00000510
0.1382
JJ00000520
0.1259
0.1785
JJ00001159
JJ00001233
0.3087
JJ00001049
0.632
0.1648
K200000527
JJ00000414
0.1539
JJ00001202
0.1728
JJ00000592
0.1347
JJ00000701
0.2377
JJ00000471
0.1217
JJ00000534
0.1571
JJ00000602
0.1163
JJ00000997

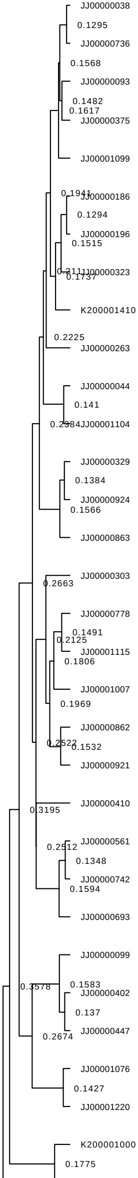

JJ00000038
0.1295
JJ00000736
0.1568
JJ00000093
0.1482
0.1617
JJ00000375
JJ00001099
0.1941
JJ00000186
0.1294
JJ00000196
0.1515
0.2111
JJ00000323
0.1737
K200001410
0.2225
JJ00000263
JJ00000044
0.141
0.2384
JJ00001104
JJ00000329
0.1384
JJ00000924
0.1566
JJ00000863
JJ00000303
0.2663
JJ00000778
0.1491
0.2125
JJ00001115
0.1806
JJ00001007
0.1969
JJ00000862
0.2522
0.1532
JJ00000921
JJ00000410
0.3195
JJ00000561
0.2512
0.1348
JJ00000742
0.1594
JJ00000693
JJ00000099
0.1583
0.3578
JJ00000402
0.137
JJ00000447
0.2674
JJ00001076
0.1427
JJ00001220
K200001000
0.1775

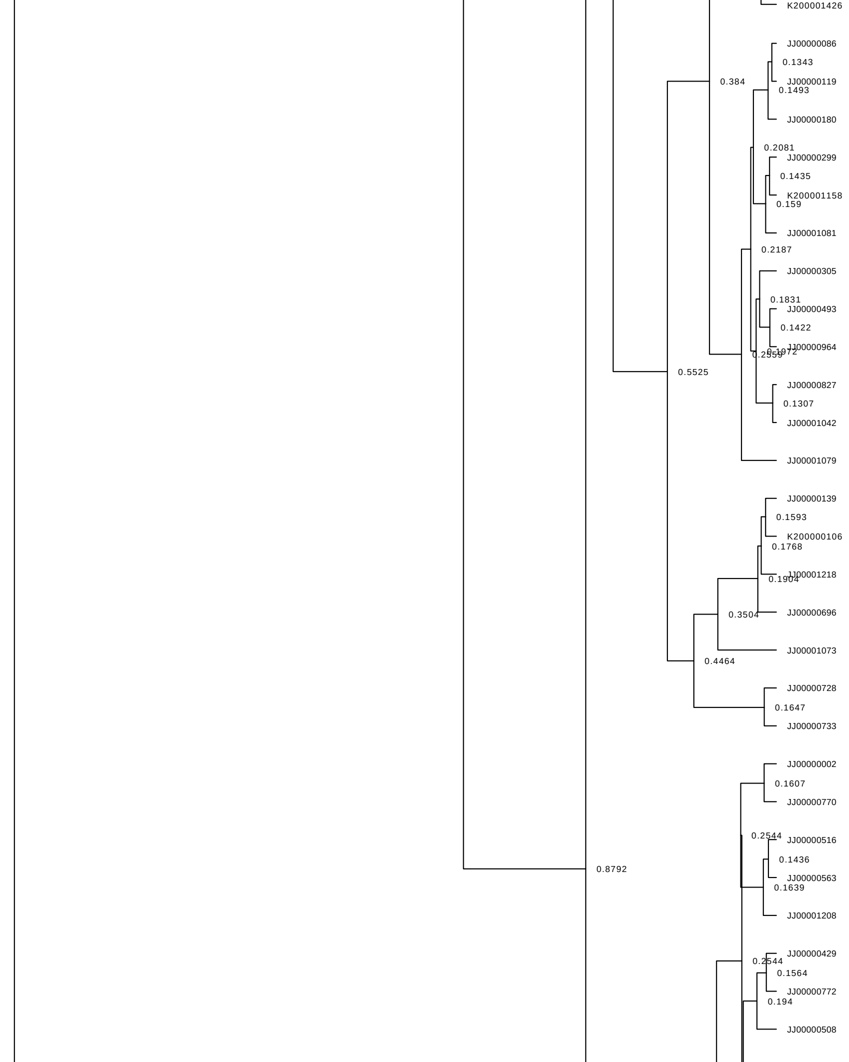
K200001426
JJ00000086
0.1343
JJ00000119
0.1493
0.384
JJ00000180
0.2081
JJ00000299
0.1435
K200001158
0.159
JJ00001081
0.2187
JJ00000305
0.1831
JJ00000493
0.1422
JJ00000964
0.1972
0.2559
0.5525
JJ00000827
0.1307
JJ00001042
JJ00001079
JJ00000139
0.1593
K200000106
0.1768
JJ00001218
0.1904
JJ00000696
0.3504
JJ00001073
0.4464
JJ00000728
0.1647
JJ00000733
JJ00000002
0.1607
JJ00000770
0.2544
JJ00000516
0.1436
JJ00000563
0.1639
0.8792
JJ00001208
JJ00000429
0.2544
0.1564
JJ00000772
0.194
JJ00000508

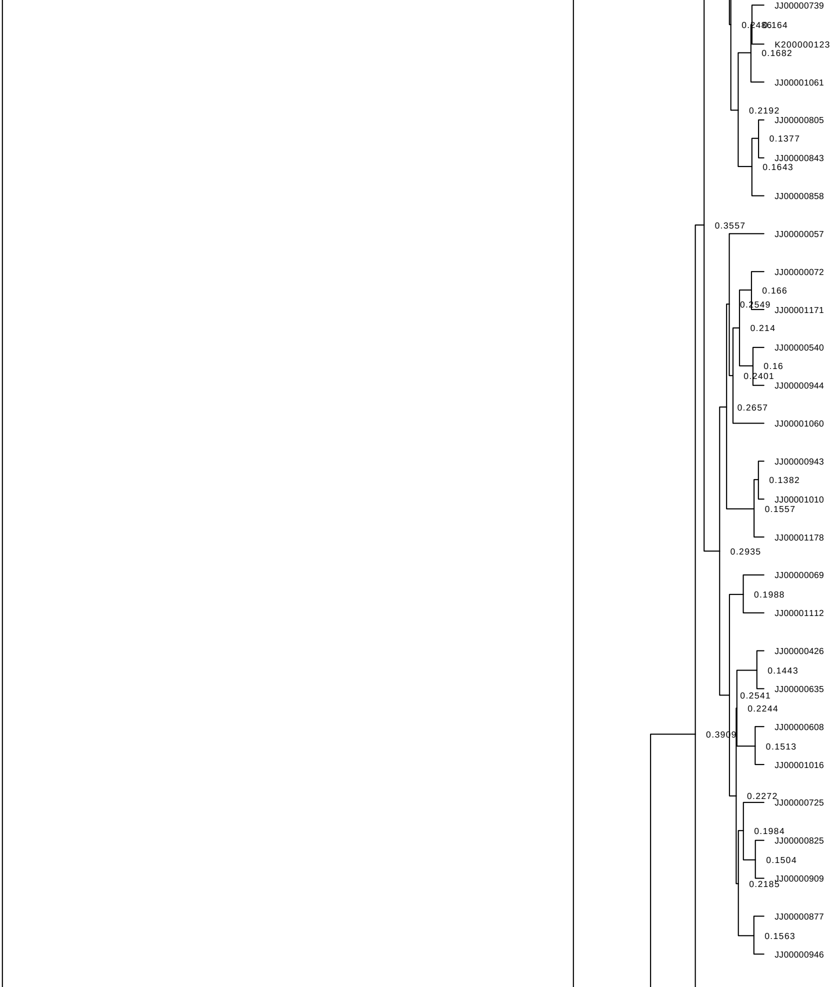
JJ00000739
K200000123
0.1682
JJ00001061
0.2192
JJ00000805
0.1377
JJ00000843
0.1643
JJ00000858
0.3557
JJ00000057
JJ00000072
0.166
0.2549
JJ00001171
0.214
JJ00000540
0.16
0.2401
JJ00000944
0.2657
JJ00001060
JJ00000943
0.1382
JJ00001010
0.1557
JJ00001178
0.2935
JJ00000069
0.1988
JJ00001112
JJ00000426
0.1443
JJ00000635
0.2541
0.2244
JJ00000608
0.3909
0.1513
JJ00001016
0.2272
JJ00000725
0.1984
JJ00000825
0.1504
JJ00000909
0.2185
JJ00000877
0.1563
JJ00000946

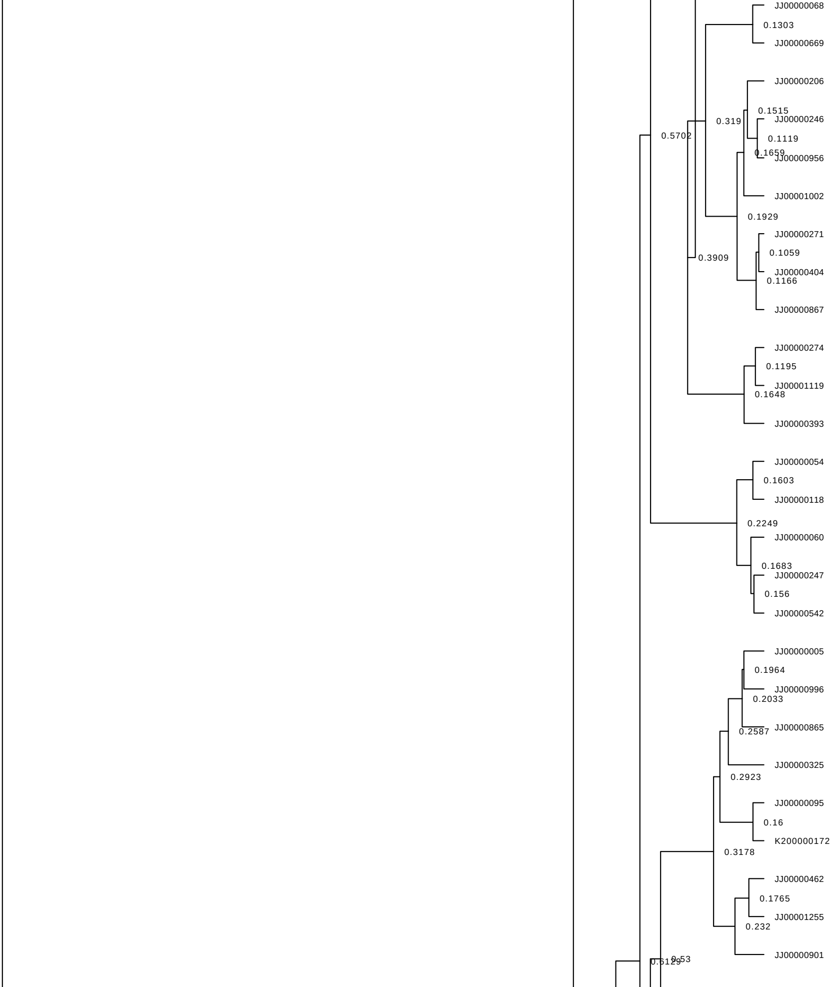
JJ00000068
0.1303
JJ00000669
JJ00000206
0.1515
JJ00000246
0.319
0.1119
0.5702
0.1659
JJ00000956
JJ00001002
0.1929
JJ00000271
0.1059
0.3909
JJ00000404
0.1166
JJ00000867
JJ00000274
0.1195
JJ00001119
0.1648
JJ00000393
JJ00000054
0.1603
JJ00000118
0.2249
JJ00000060
0.1683
JJ00000247
0.156
JJ00000542
JJ00000005
0.1964
JJ00000996
0.2033
JJ00000865
0.2587
JJ00000325
0.2923
JJ00000095
0.16
K200000172
0.3178
JJ00000462
0.1765
JJ00001255
0.232
JJ00000901
0.6129
0.53

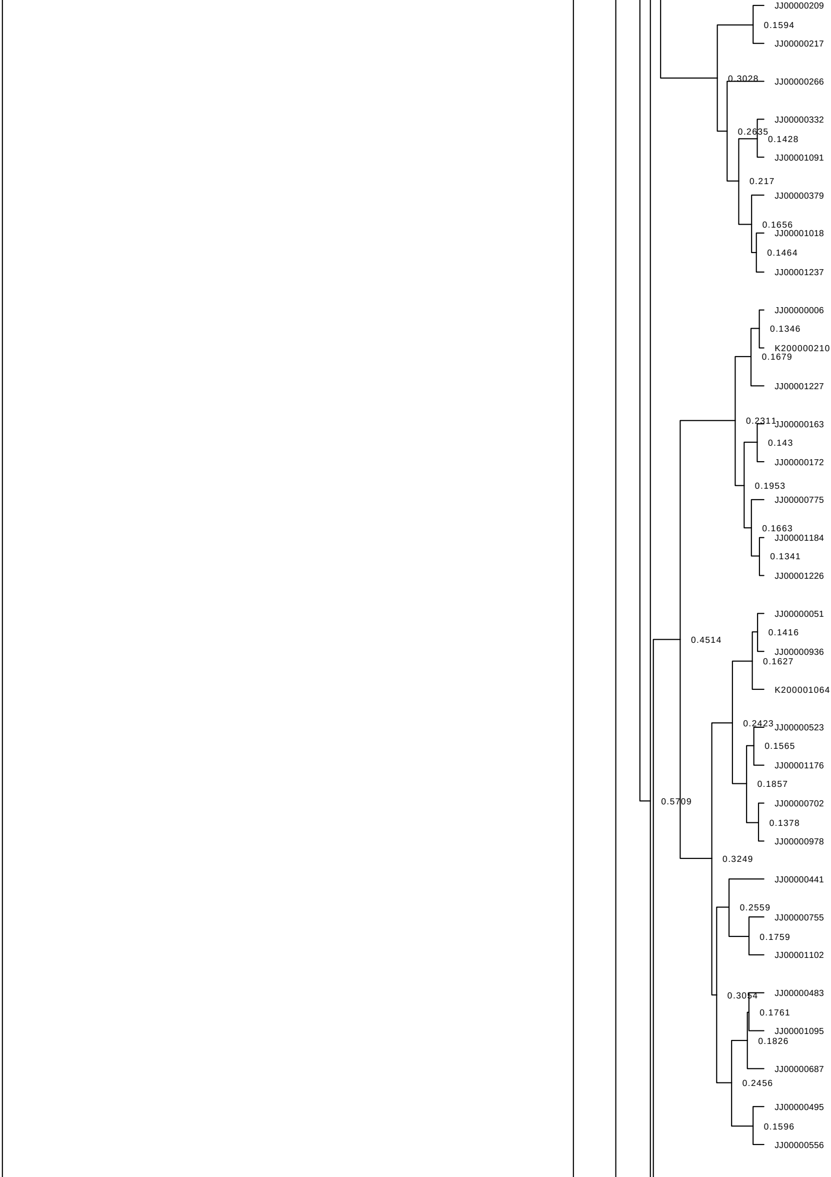
JJ00000209
0.1594
JJ00000217
0.3028
JJ00000266
JJ00000332
0.2635
0.1428
JJ00001091
0.217
JJ00000379
0.1656
JJ00001018
0.1464
JJ00001237
JJ00000006
0.1346
K200000210
0.1679
JJ00001227
0.2311
JJ00000163
0.143
JJ00000172
0.1953
JJ00000775
0.1663
JJ00001184
0.1341
JJ00001226
JJ00000051
0.1416
0.4514
JJ00000936
0.1627
K200001064
0.2423
JJ00000523
0.1565
JJ00001176
0.1857
JJ00000702
0.5709
0.1378
JJ00000978
0.3249
JJ00000441
0.2559
JJ00000755
0.1759
JJ00001102
0.3054
JJ00000483
0.1761
JJ00001095
0.1826
JJ00000687
0.2456
JJ00000495
0.1596
JJ00000556

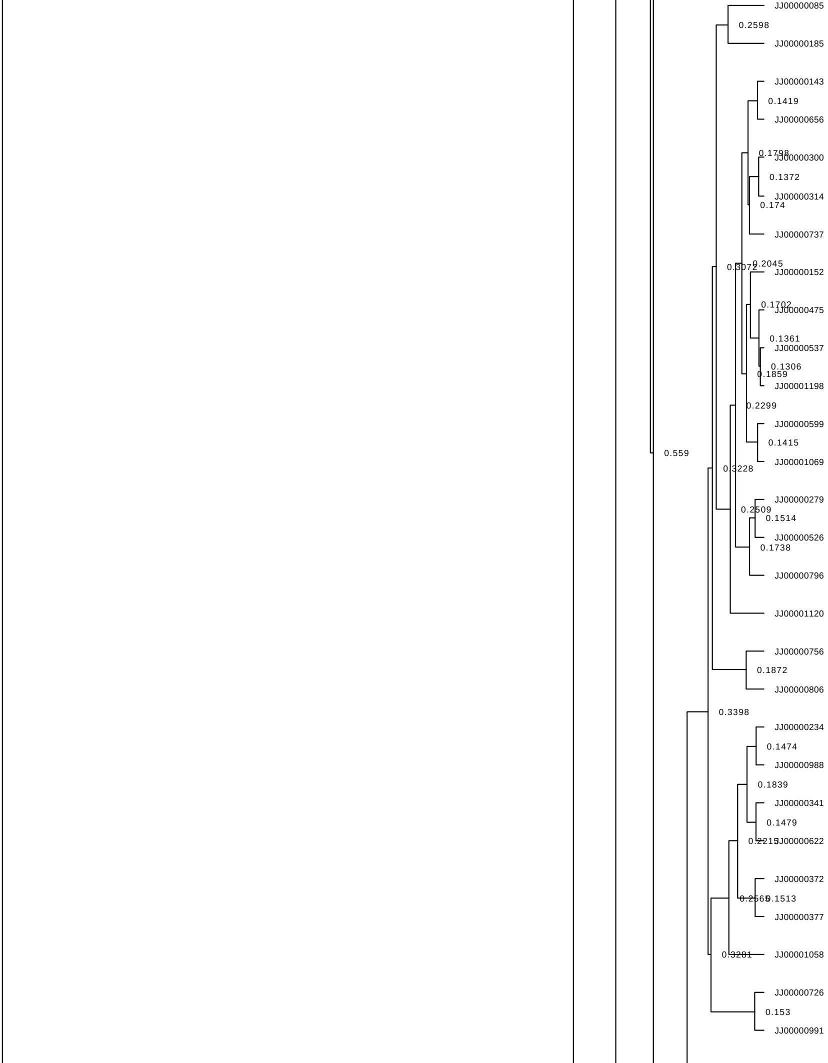
JJ00000085
0.2598
JJ00000185
JJ00000143
0.1419
JJ00000656
0.1798
JJ00000300
0.1372
JJ00000314
0.174
JJ00000737
0.2045
0.3072
JJ00000152
0.1702
JJ00000475
0.1361
JJ00000537
0.1306
0.1859
JJ00001198
0.2299
JJ00000599
0.1415
JJ00001069
0.559
0.3228
JJ00000279
0.2509
0.1514
JJ00000526
0.1738
JJ00000796
JJ00001120
JJ00000756
0.1872
JJ00000806
0.3398
JJ00000234
0.1474
JJ00000988
0.1839
JJ00000341
0.1479
0.2215
JJ00000622
JJ00000372
0.2565
0.1513
JJ00000377
0.3281
JJ00001058
JJ00000726
0.153
JJ00000991

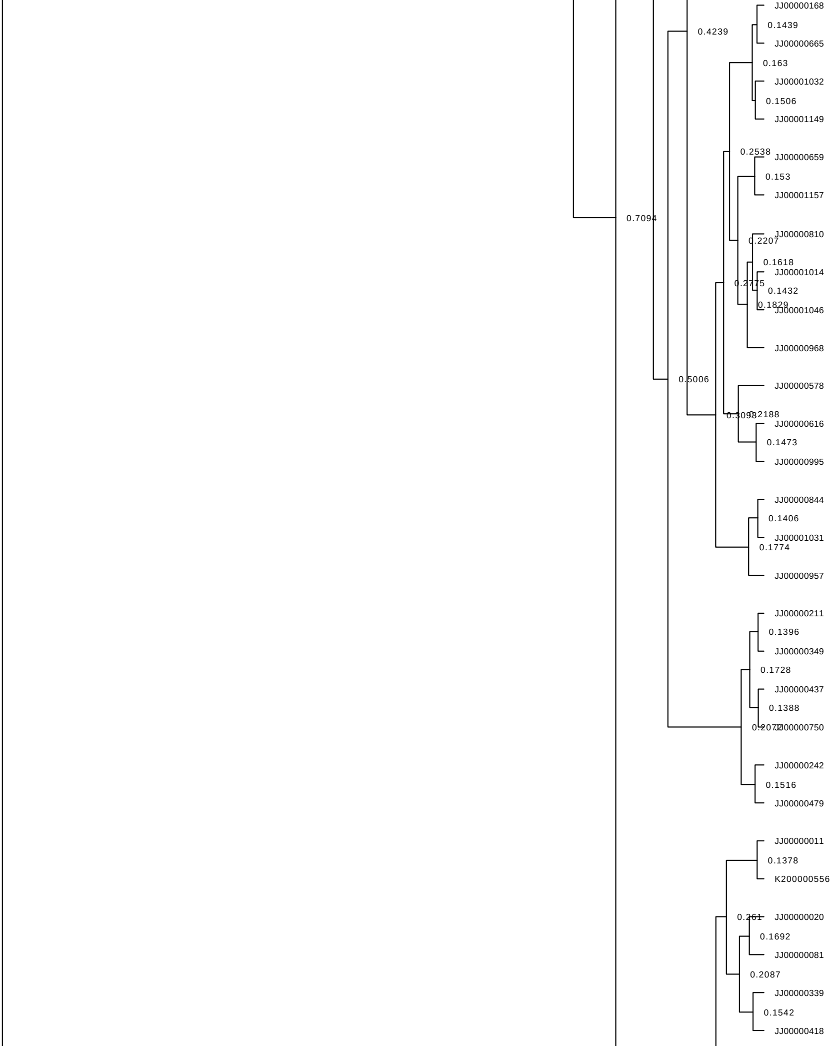
JJ00000168
0.1439
0.4239
JJ00000665
0.163
JJ00001032
0.1506
JJ00001149
0.2538
JJ00000659
0.153
JJ00001157
0.7094
JJ00000810
0.2207
0.1618
JJ00001014
0.2775
0.1432
0.1829
JJ00001046
JJ00000968
0.5006
JJ00000578
0.3098
0.2188
JJ00000616
0.1473
JJ00000995
JJ00000844
0.1406
JJ00001031
0.1774
JJ00000957
JJ00000211
0.1396
JJ00000349
0.1728
JJ00000437
0.1388
0.2072
JJ00000750
JJ00000242
0.1516
JJ00000479
JJ00000011
0.1378
K200000556
0.261
JJ00000020
0.1692
JJ00000081
0.2087
JJ00000339
0.1542
JJ00000418

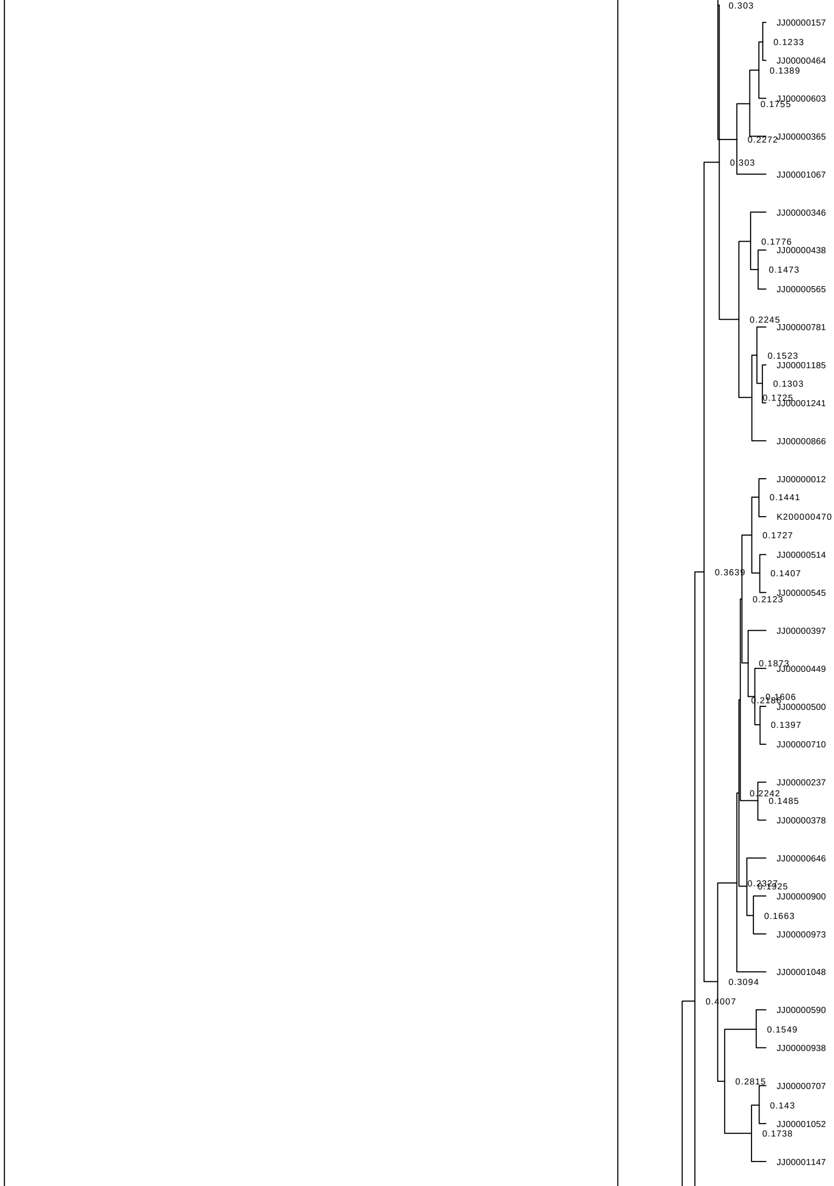
0.303
JJ00000157
0.1233
JJ00000464
0.1389
JJ00000603
0.1755
JJ00000365
0.2272
0.303
JJ00001067
JJ00000346
0.1776
JJ00000438
0.1473
JJ00000565
0.2245
JJ00000781
0.1523
JJ00001185
0.1303
0.1725
JJ00001241
JJ00000866
JJ00000012
0.1441
K200000470
0.1727
JJ00000514
0.3639
0.1407
JJ00000545
0.2123
JJ00000397
0.1873
JJ00000449
0.1606
0.2186
JJ00000500
0.1397
JJ00000710
JJ00000237
0.2242
0.1485
JJ00000378
JJ00000646
0.2327
0.1925
JJ00000900
0.1663
JJ00000973
JJ00001048
0.3094
0.4007
JJ00000590
0.1549
JJ00000938
0.2815
JJ00000707
0.143
JJ00001052
0.1738
JJ00001147

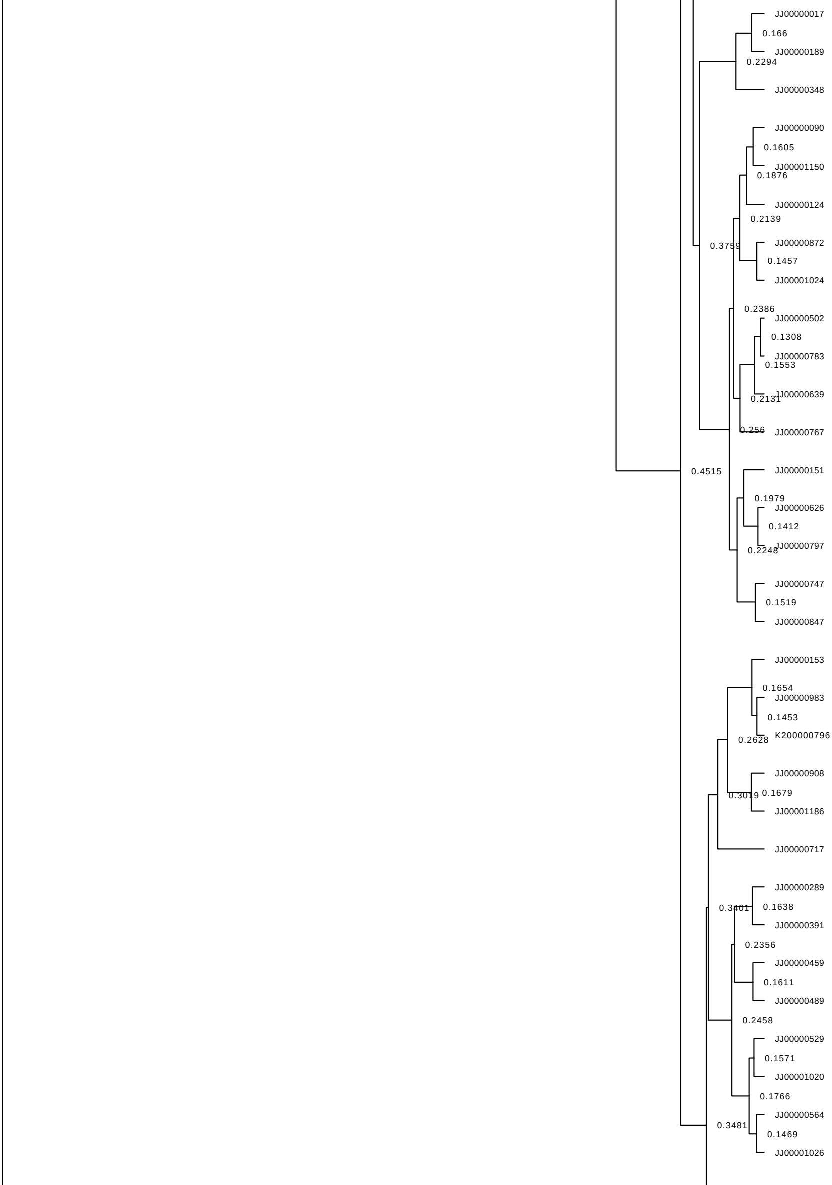
JJ00000017
0.166
JJ00000189
0.2294
JJ00000348
JJ00000090
0.1605
JJ00001150
0.1876
JJ00000124
0.2139
JJ00000872
0.3759
0.1457
JJ00001024
0.2386
JJ00000502
0.1308
JJ00000783
0.1553
JJ00000639
0.2131
0.256
JJ00000767
JJ00000151
0.4515
0.1979
JJ00000626
0.1412
JJ00000797
0.2248
JJ00000747
0.1519
JJ00000847
JJ00000153
0.1654
JJ00000983
0.1453
K200000796
0.2628
JJ00000908
0.1679
0.3019
JJ00001186
JJ00000717
JJ00000289
0.3401
0.1638
JJ00000391
0.2356
JJ00000459
0.1611
JJ00000489
0.2458
JJ00000529
0.1571
JJ00001020
0.1766
JJ00000564
0.3481
0.1469
JJ00001026

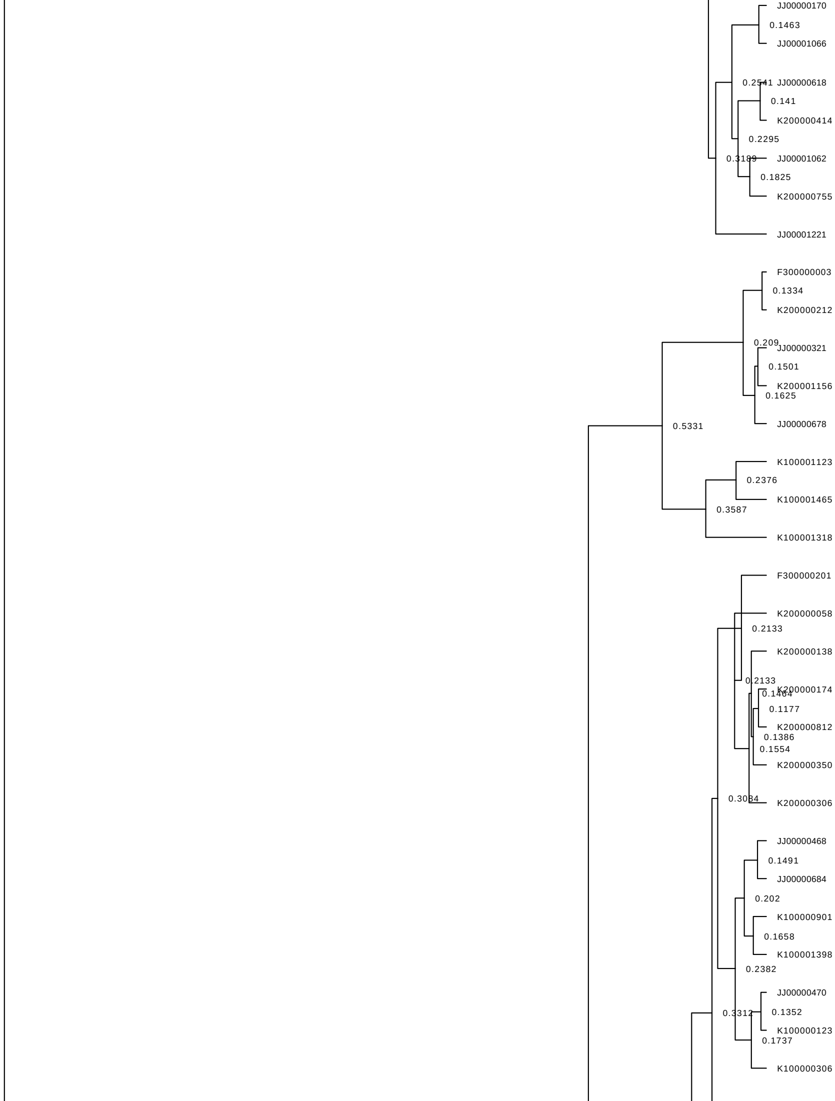
JJ00000170
0.1463
JJ00001066
0.2541
JJ00000618
0.141
K200000414
0.2295
0.3189
JJ00001062
0.1825
K200000755
JJ00001221
F300000003
0.1334
K200000212
0.209
JJ00000321
0.1501
K200001156
0.1625
JJ00000678
0.5331
K100001123
0.2376
K100001465
0.3587
K100001318
F300000201
K200000058
0.2133
K200000138
0.2133
K200000174
0.1464
0.1177
K200000812
0.1386
0.1554
K200000350
0.3084
K200000306
JJ00000468
0.1491
JJ00000684
0.202
K100000901
0.1658
K100001398
0.2382
JJ00000470
0.3312
0.1352
K100000123
0.1737
K100000306

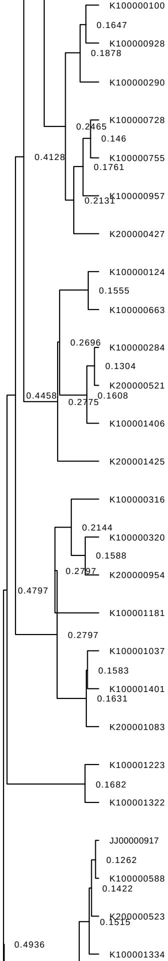
K100000100
0.1647
K100000928
0.1878
K100000290
K100000728
0.2465
0.146
K100000755
0.4128
0.1761
K100000957
0.2131
K200000427
K100000124
0.1555
K100000663
0.2696
K100000284
0.1304
K200000521
0.4458
0.1608
0.2775
K100001406
K200001425
K100000316
0.2144
K100000320
0.1588
0.2797
K200000954
0.4797
K100001181
0.2797
K100001037
0.1583
K100001401
0.1631
K200001083
K100001223
0.1682
K100001322
JJ00000917
0.1262
K100000588
0.1422
K200000523
0.1515
0.4936
K100001334

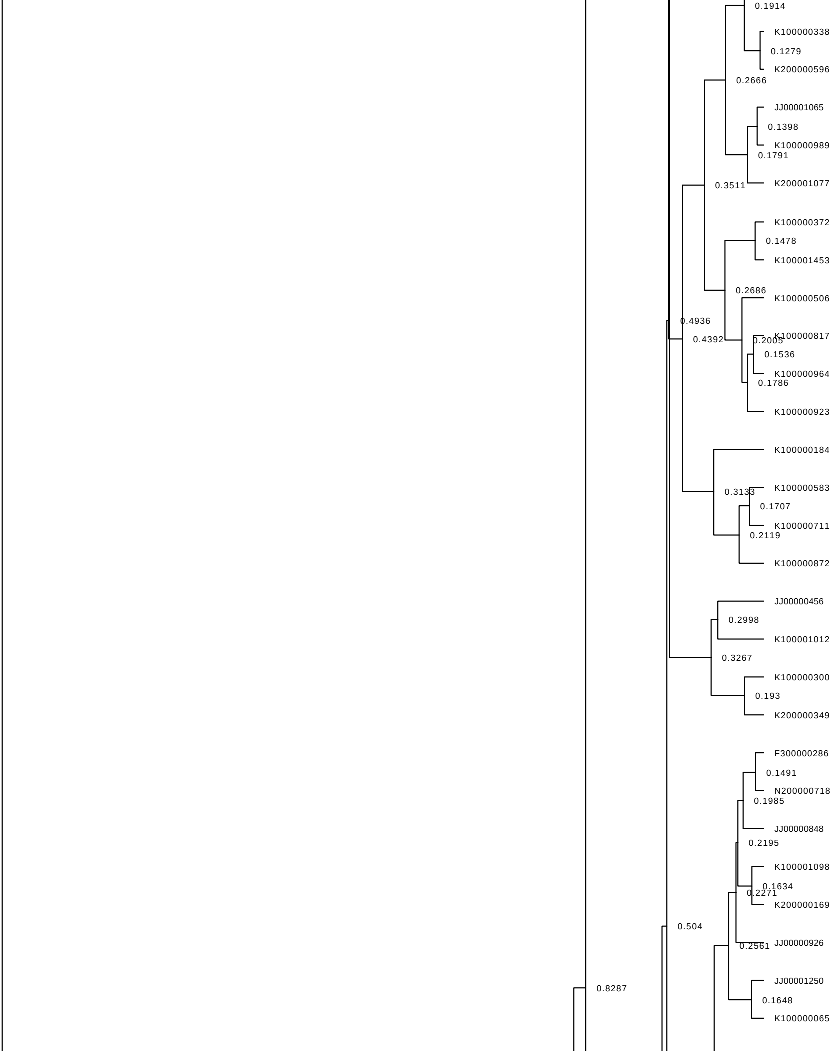

0.1914
K100000338
0.1279
K200000596
0.2666
JJ00001065
0.1398
K100000989
0.1791
K200001077
0.3511
K100000372
0.1478
K100001453
0.2686
K100000506
0.4936
K100000817
0.4392
0.2005
0.1536
K100000964
0.1786
K100000923
K100000184
K100000583
0.3133
0.1707
K100000711
0.2119
K100000872
JJ00000456
0.2998
K100001012
0.3267
K100000300
0.193
K200000349
F300000286
0.1491
N200000718
0.1985
JJ00000848
0.2195
K100001098
0.1634
0.2271
K200000169
0.504
JJ00000926
0.2561
0.8287
JJ00001250
0.1648
K100000065

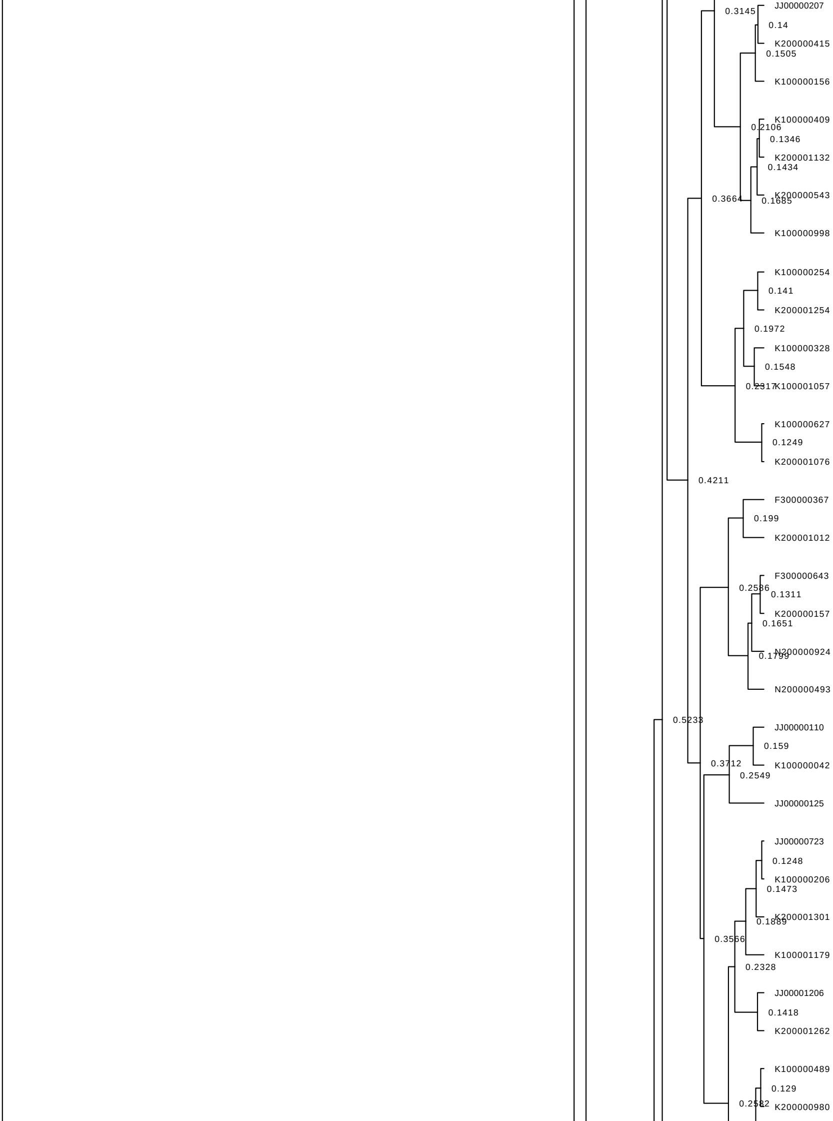

JJ00000207
0.3145
0.14
K200000415
0.1505
K100000156
K100000409
0.2106
0.1346
K200001132
0.1434
K200000543
0.3664
0.1685
K100000998
K100000254
0.141
K200001254
0.1972
K100000328
0.1548
0.2317
K100001057
K100000627
0.1249
K200001076
0.4211
F300000367
0.199
K200001012
F300000643
0.1311
K200000157
0.1651
N200000924
0.1799
N200000493
0.5233
JJ00000110
0.159
0.3712
K100000042
0.2549
JJ00000125
JJ00000723
0.1248
K100000206
0.1473
K200001301
0.1889
0.3566
K100001179
0.2328
JJ00001206
0.1418
K200001262
K100000489
0.129
K200000980

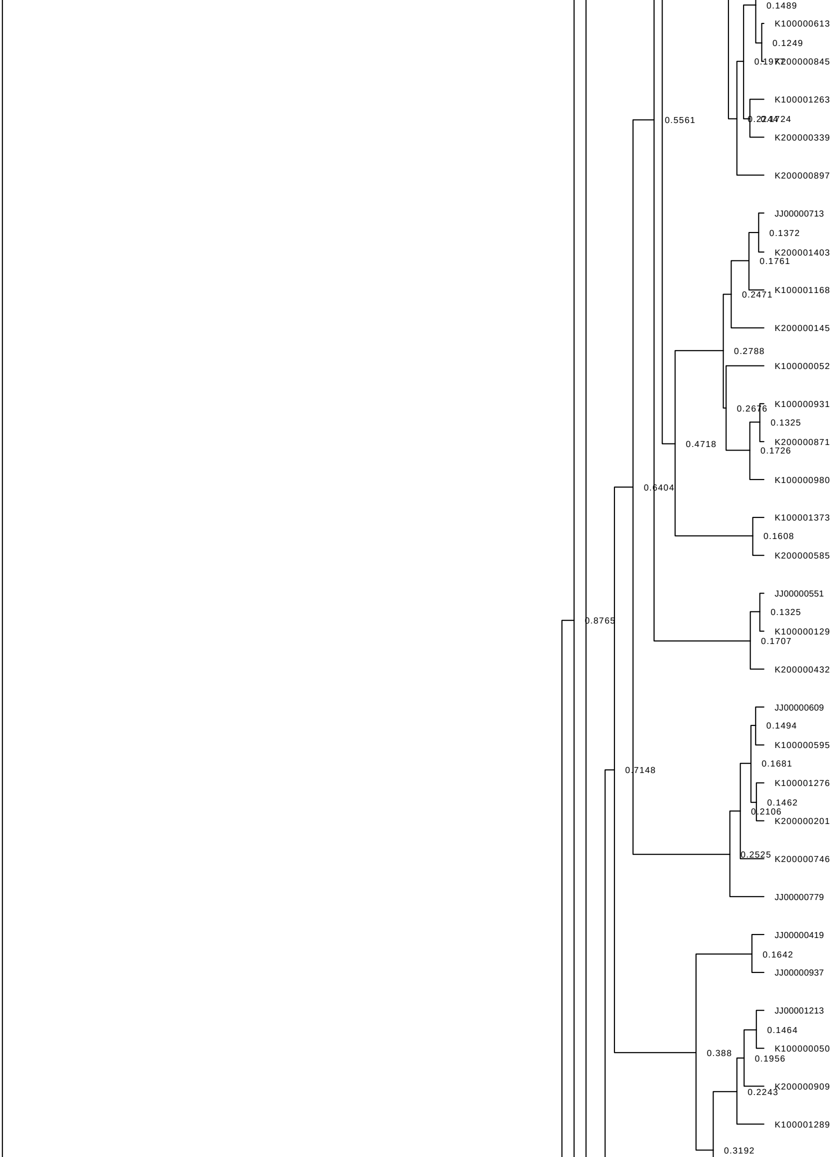
0.1489
K100000613
0.1249
0.1972
K200000845
K100001263
0.2044
0.1724
0.5561
K200000339
K200000897
JJ00000713
0.1372
K200001403
0.1761
K100001168
0.2471
K200000145
0.2788
K100000052
K100000931
0.2676
0.1325
K200000871
0.1726
0.4718
K100000980
0.6404
K100001373
0.1608
K200000585
JJ00000551
0.1325
0.8765
K100000129
0.1707
K200000432
JJ00000609
0.1494
K100000595
0.1681
0.7148
K100001276
0.1462
0.2106
K200000201
0.2525
K200000746
JJ00000779
JJ00000419
0.1642
JJ00000937
JJ00001213
0.1464
K100000050
0.388
0.1956
K200000909
0.2243
K100001289
0.3192

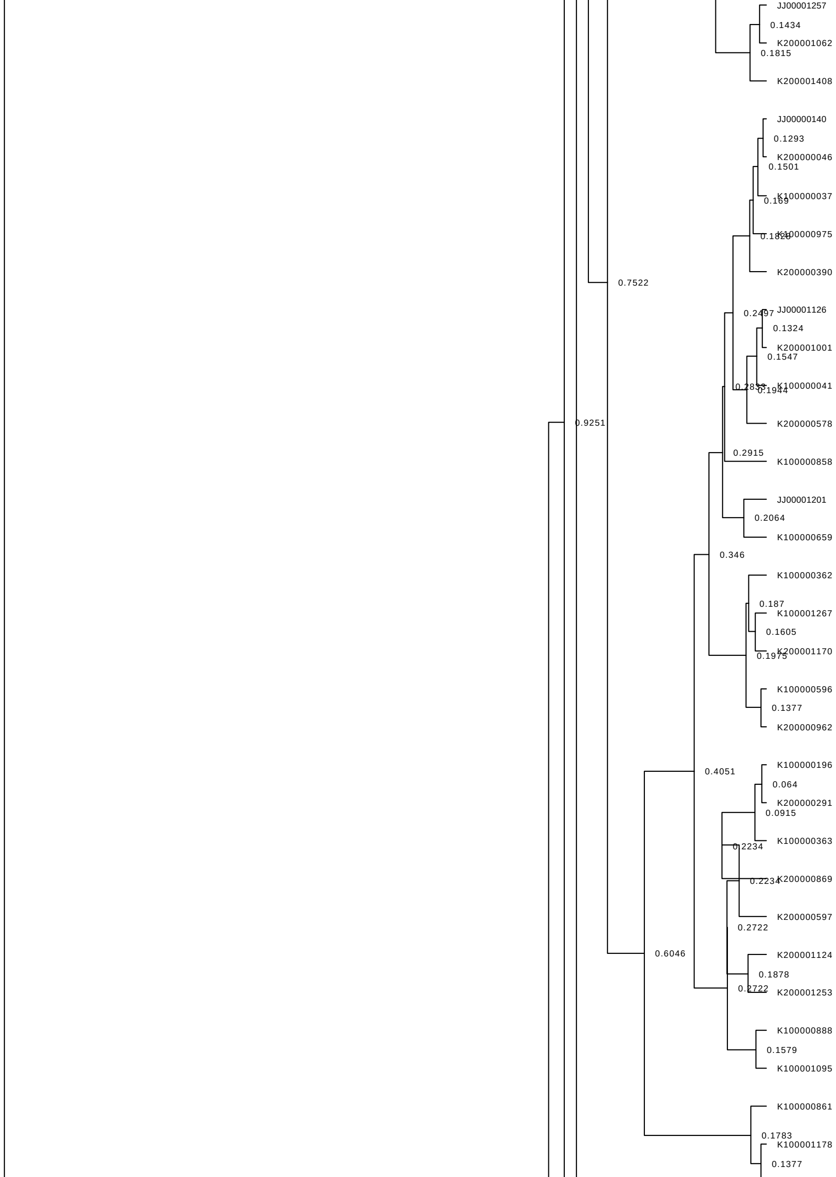
JJ00001257
0.1434
K200001062
0.1815
K200001408
JJ00000140
0.1293
K200000046
0.1501
K100000037
0.169
K100000975
0.1828
K200000390
0.7522
0.2497
JJ00001126
0.1324
K200001001
0.1547
0.2833
K100000041
0.1944
K200000578
0.9251
0.2915
K100000858
JJ00001201
0.2064
K100000659
0.346
K100000362
0.187
K100001267
0.1605
K200001170
0.1975
K100000596
0.1377
K200000962
K100000196
0.4051
0.064
K200000291
0.0915
K100000363
0.2234
K200000869
0.2234
K200000597
0.2722
K200001124
0.6046
0.1878
K200001253
0.2722
K100000888
0.1579
K100001095
K100000861
0.1783
K100001178
0.1377

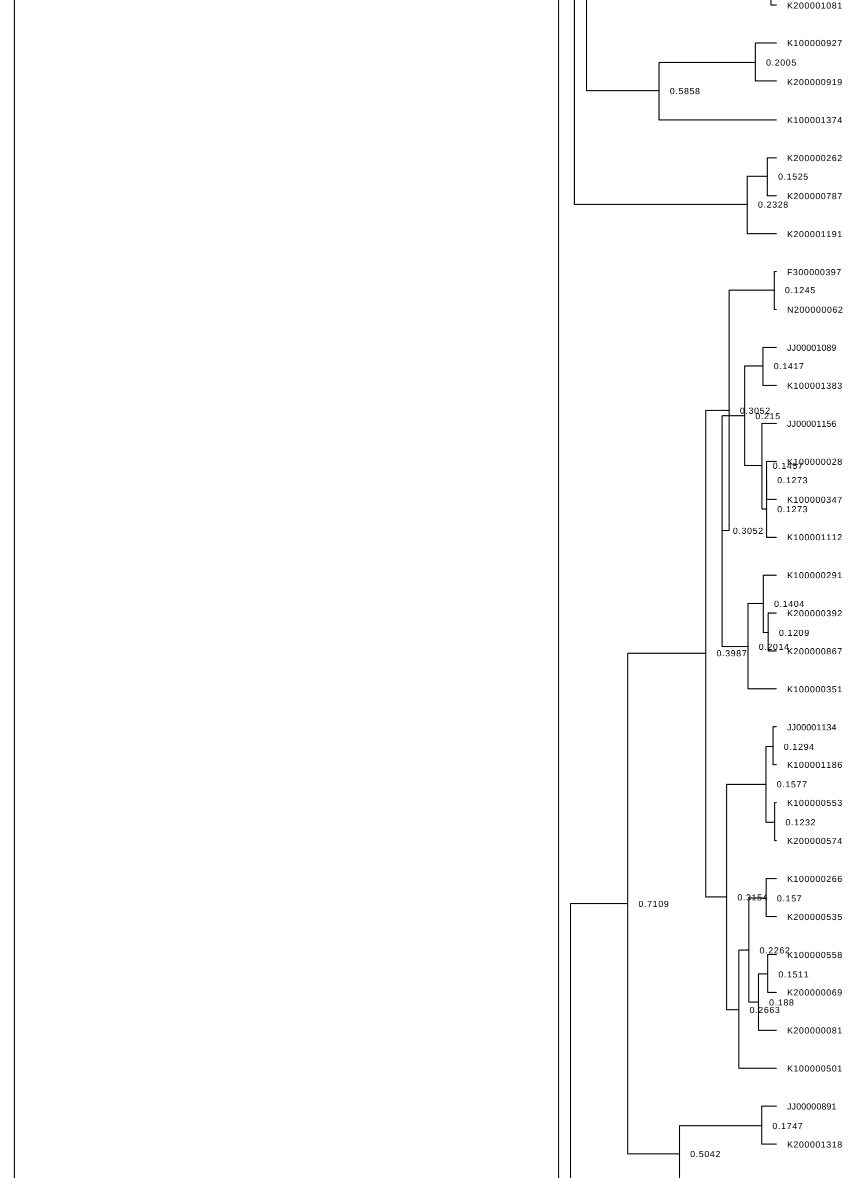
K200001081
K100000927
0.2005
K200000919
0.5858
K100001374
K200000262
0.1525
K200000787
0.2328
K200001191
F300000397
0.1245
N200000062
JJ00001089
0.1417
K100001383
0.3052
0.215
JJ00001156
K100000028
0.1457
0.1273
K100000347
0.1273
0.3052
K100001112
K100000291
0.1404
K200000392
0.1209
0.2014
K200000867
0.3987
K100000351
JJ00001134
0.1294
K100001186
0.1577
K100000553
0.1232
K200000574
K100000266
0.3154
0.157
0.7109
K200000535
0.2262
K100000558
0.1511
K200000069
0.188
0.2663
K200000081
K100000501
JJ00000891
0.1747
K200001318
0.5042

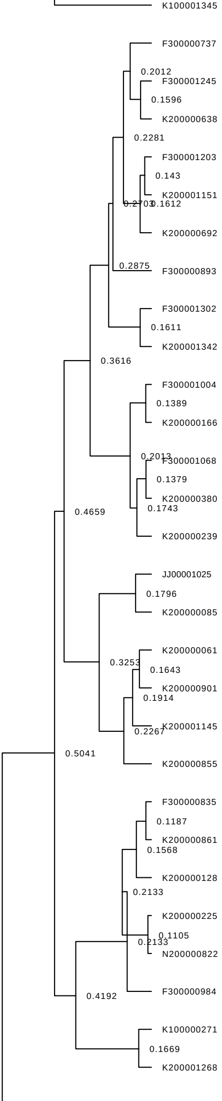

K100001345
F300000737
0.2012
F300001245
0.1596
K200000638
0.2281
F300001203
0.143
K200001151
0.2703
0.1612
K200000692
0.2875
F300000893
F300001302
0.1611
K200001342
0.3616
F300001004
0.1389
K200000166
0.2013
F300001068
0.1379
K200000380
0.1743
0.4659
K200000239
JJ00001025
0.1796
K200000085
K200000061
0.3253
0.1643
K200000901
0.1914
K200001145
0.2267
0.5041
K200000855
F300000835
0.1187
K200000861
0.1568
K200000128
0.2133
K200000225
0.1105
0.2133
N200000822
F300000984
0.4192
K100000271
0.1669
K200001268

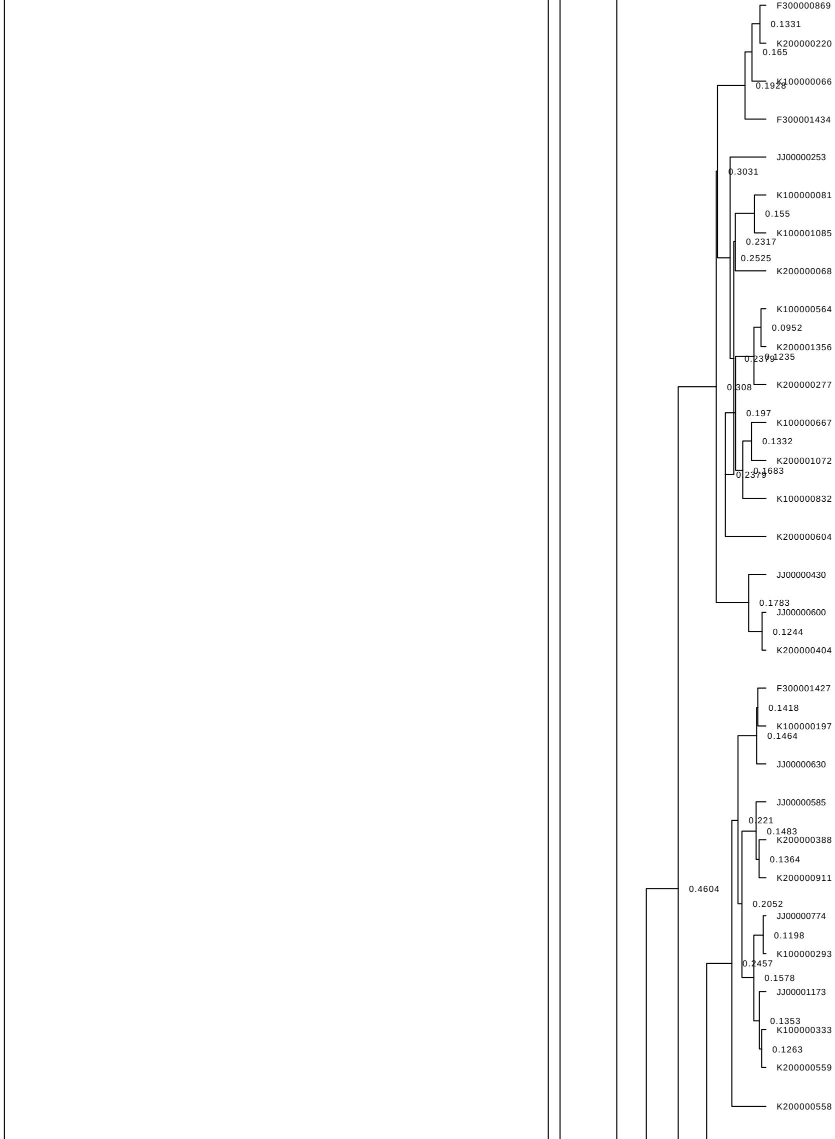
F300000869
0.1331
K200000220
0.165
K100000066
0.1928
F300001434
JJ00000253
0.3031
K100000081
0.155
K100001085
0.2317
0.2525
K200000068
K100000564
0.0952
K200001356
0.2379
0.1235
K200000277
0.308
0.197
K100000667
0.1332
K200001072
0.1683
0.2379
K100000832
K200000604
JJ00000430
0.1783
JJ00000600
0.1244
K200000404
F300001427
0.1418
K100000197
0.1464
JJ00000630
JJ00000585
0.221
0.1483
K200000388
0.1364
K200000911
0.4604
0.2052
JJ00000774
0.1198
K100000293
0.2457
0.1578
JJ00001173
0.1353
K100000333
0.1263
K200000559
K200000558

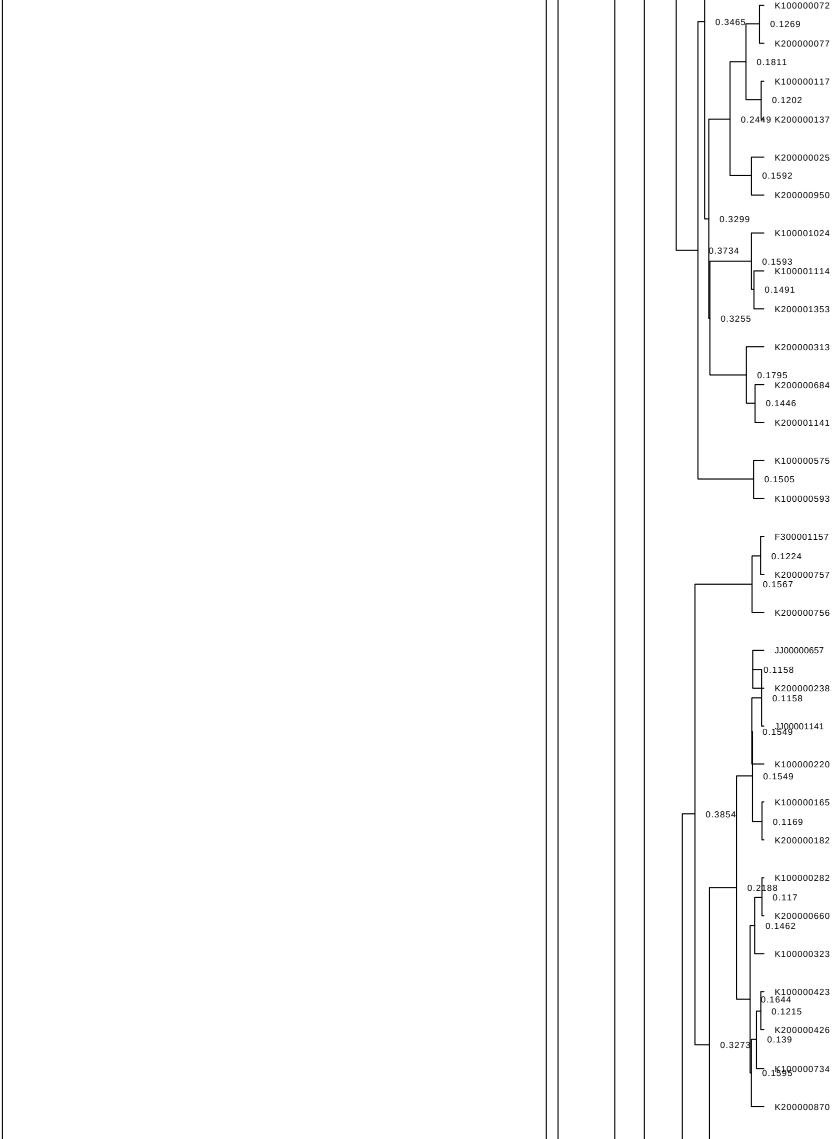
K100000072
0.3465
0.1269
K200000077
0.1811
K100000117
0.1202
0.2449
K200000137
K200000025
0.1592
K200000950
0.3299
K100001024
0.3734
0.1593
K100001114
0.1491
K200001353
0.3255
K200000313
0.1795
K200000684
0.1446
K200001141
K100000575
0.1505
K100000593
F300001157
0.1224
K200000757
0.1567
K200000756
JJ00000657
0.1158
K200000238
0.1158
JJ00001141
0.1549
K100000220
0.1549
K100000165
0.3854
0.1169
K200000182
K100000282
0.2188
0.117
K200000660
0.1462
K100000323
K100000423
0.1644
0.1215
K200000426
0.139
0.3273
K100000734
0.1595
K200000870

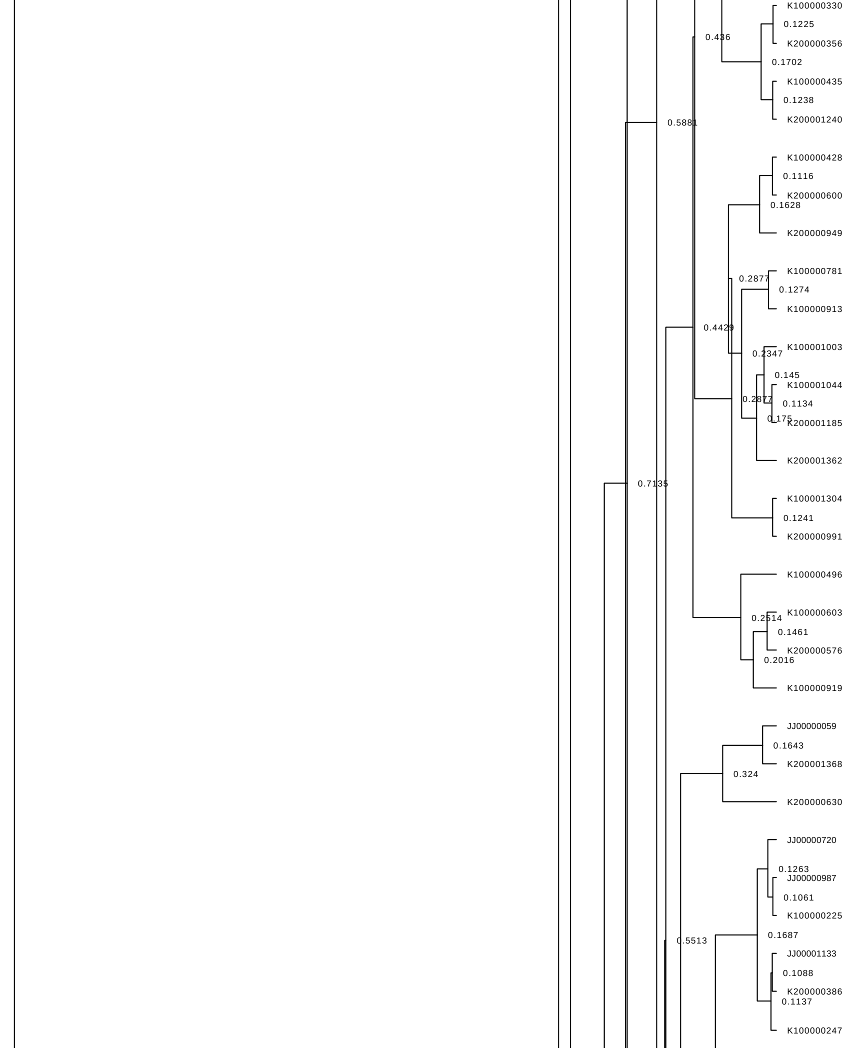
K100000330
0.1225
K200000356
0.436
0.1702
K100000435
0.1238
K200001240
0.5881
K100000428
0.1116
K200000600
0.1628
K200000949
K100000781
0.2877
0.1274
K100000913
0.4429
K100001003
0.2347
0.145
K100001044
0.2877
0.1134
0.175
K200001185
K200001362
0.7135
K100001304
0.1241
K200000991
K100000496
K100000603
0.2514
0.1461
K200000576
0.2016
K100000919
JJ00000059
0.1643
K200001368
0.324
K200000630
JJ00000720
0.1263
JJ00000987
0.1061
K100000225
0.1687
0.5513
JJ00001133
0.1088
K200000386
0.1137
K100000247

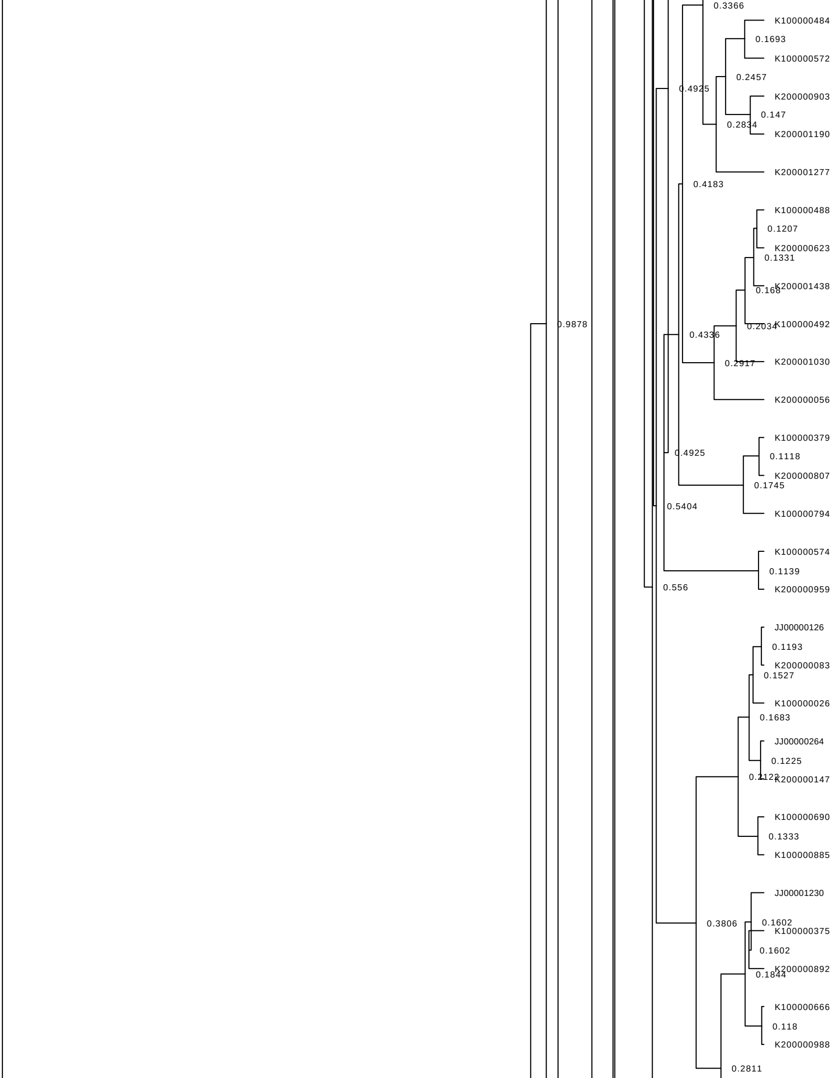

0.3366
K100000484
0.1693
K100000572
0.2457
0.4925
K200000903
0.147
0.2834
K200001190
K200001277
0.4183
K100000488
0.1207
K200000623
0.1331
K200001438
0.168
0.9878
K100000492
0.2034
0.4336
K200001030
0.2917
K200000056
K100000379
0.4925
0.1118
K200000807
0.1745
0.5404
K100000794
K100000574
0.1139
0.556
K200000959
JJ00000126
0.1193
K200000083
0.1527
K100000026
0.1683
JJ00000264
0.1225
0.2122
K200000147
K100000690
0.1333
K100000885
JJ00001230
0.1602
0.3806
K100000375
0.1602
K200000892
0.1844
K100000666
0.118
K200000988
0.2811

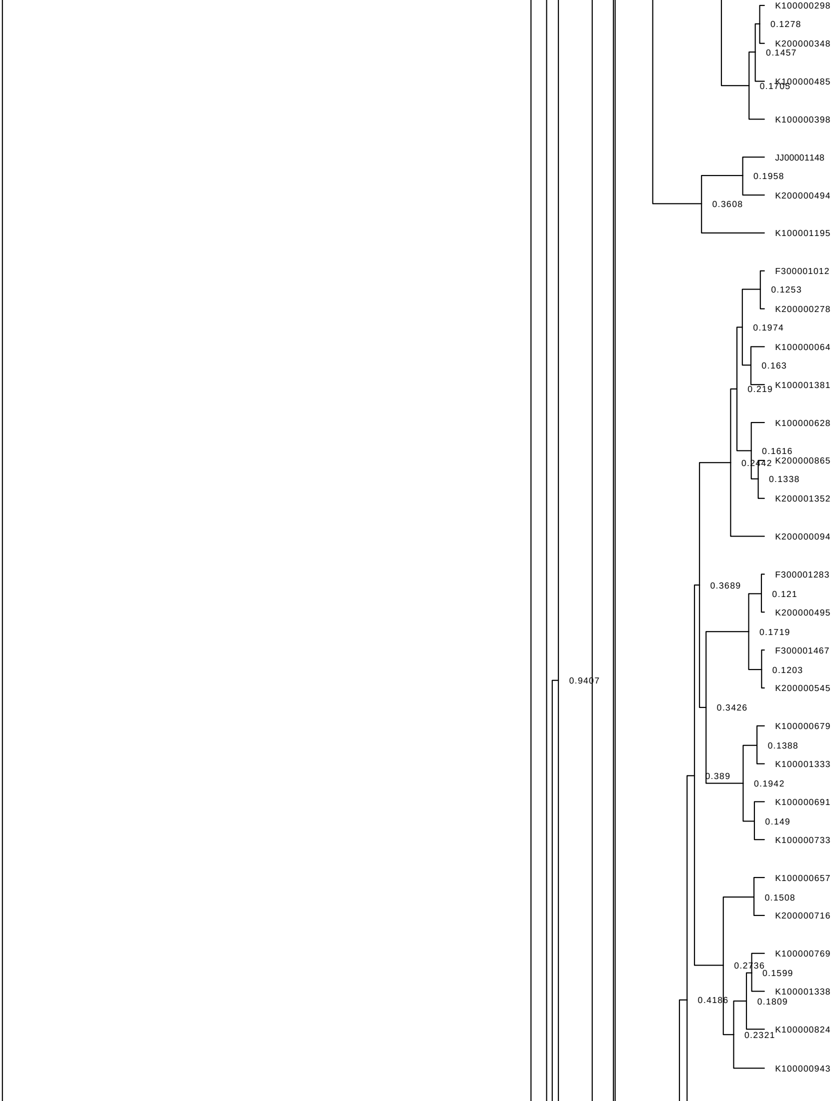

K100000298
0.1278
K200000348
0.1457
K100000485
0.1705
K100000398
JJ00001148
0.1958
K200000494
0.3608
K100001195
F300001012
0.1253
K200000278
0.1974
K100000064
0.163
K100001381
0.219
K100000628
0.1616
K200000865
0.2442
0.1338
K200001352
K200000094
F300001283
0.3689
0.121
K200000495
0.1719
F300001467
0.1203
0.9407
K200000545
0.3426
K100000679
0.1388
K100001333
0.389
0.1942
K100000691
0.149
K100000733
K100000657
0.1508
K200000716
K100000769
0.2736
0.1599
K100001338
0.4186
0.1809
K100000824
0.2321
K100000943

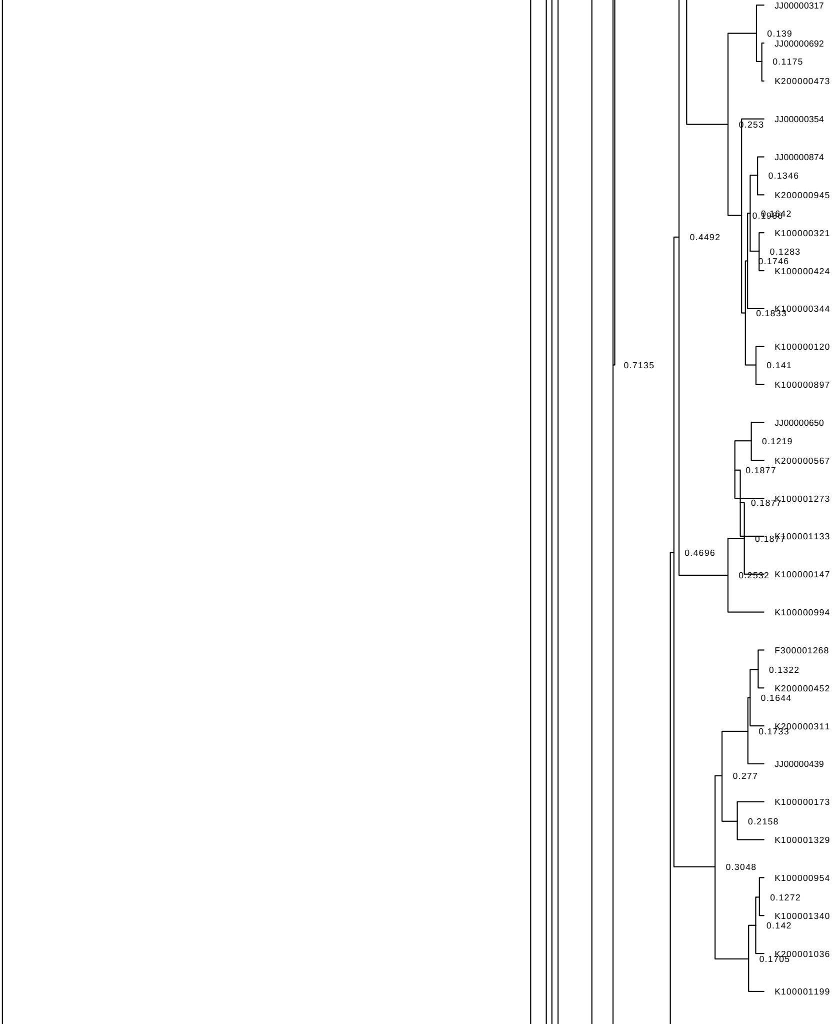
JJ00000317
0.139
JJ00000692
0.1175
K200000473
JJ00000354
0.253
JJ00000874
0.1346
K200000945
K100000321
0.1283
0.1746
K100000424
K100000344
0.1833
0.4492
K100000120
0.141
K100000897
0.7135
JJ00000650
0.1219
K200000567
0.1877
K100001273
0.1877
K100001133
0.1877
0.4696
K100000147
0.2532
K100000994
F300001268
0.1322
K200000452
0.1644
K200000311
0.1733
JJ00000439
0.277
K100000173
0.2158
K100001329
0.3048
K100000954
0.1272
K100001340
0.142
K200001036
0.1705
K100001199

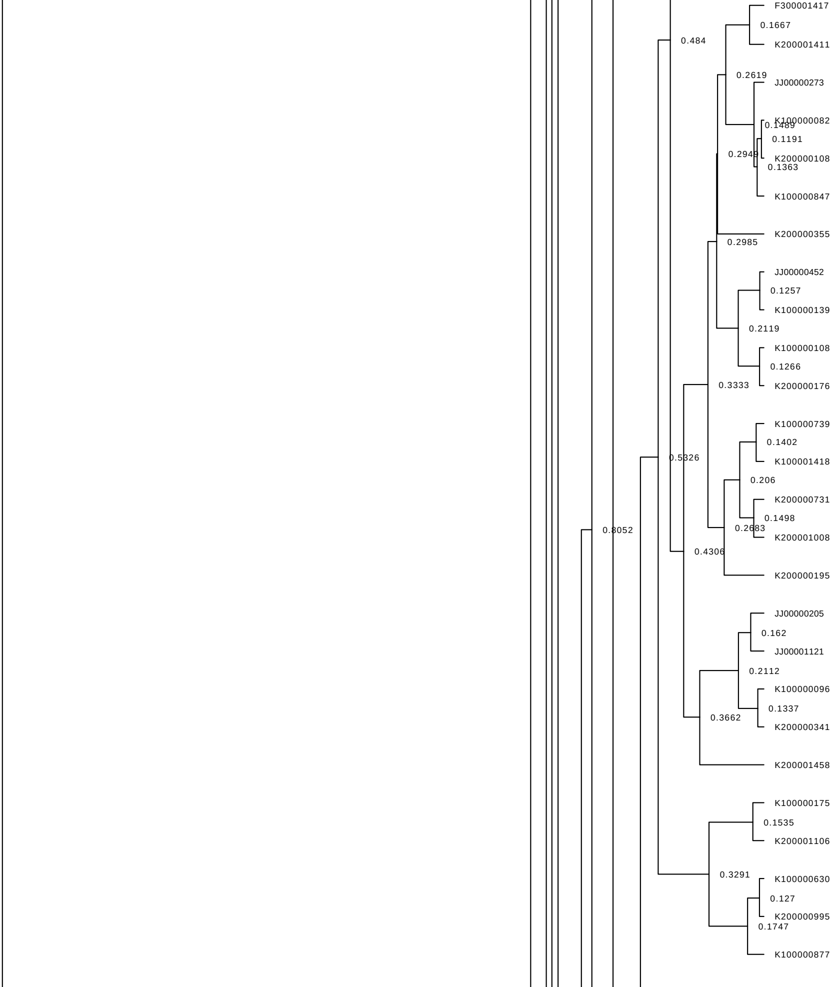
F300001417
0.1667
K200001411
0.484
0.2619
JJ00000273
K100000082
0.1489
0.1191
0.2949
K200000108
0.1363
K100000847
K200000355
0.2985
JJ00000452
0.1257
K100000139
0.2119
K100000108
0.1266
K200000176
0.3333
K100000739
0.1402
K100001418
0.5326
0.206
K200000731
0.1498
0.2683
K200001008
0.8052
0.4306
K200000195
JJ00000205
0.162
JJ00001121
0.2112
K100000096
0.1337
0.3662
K200000341
K200001458
K100000175
0.1535
K200001106
0.3291
K100000630
0.127
K200000995
0.1747
K100000877

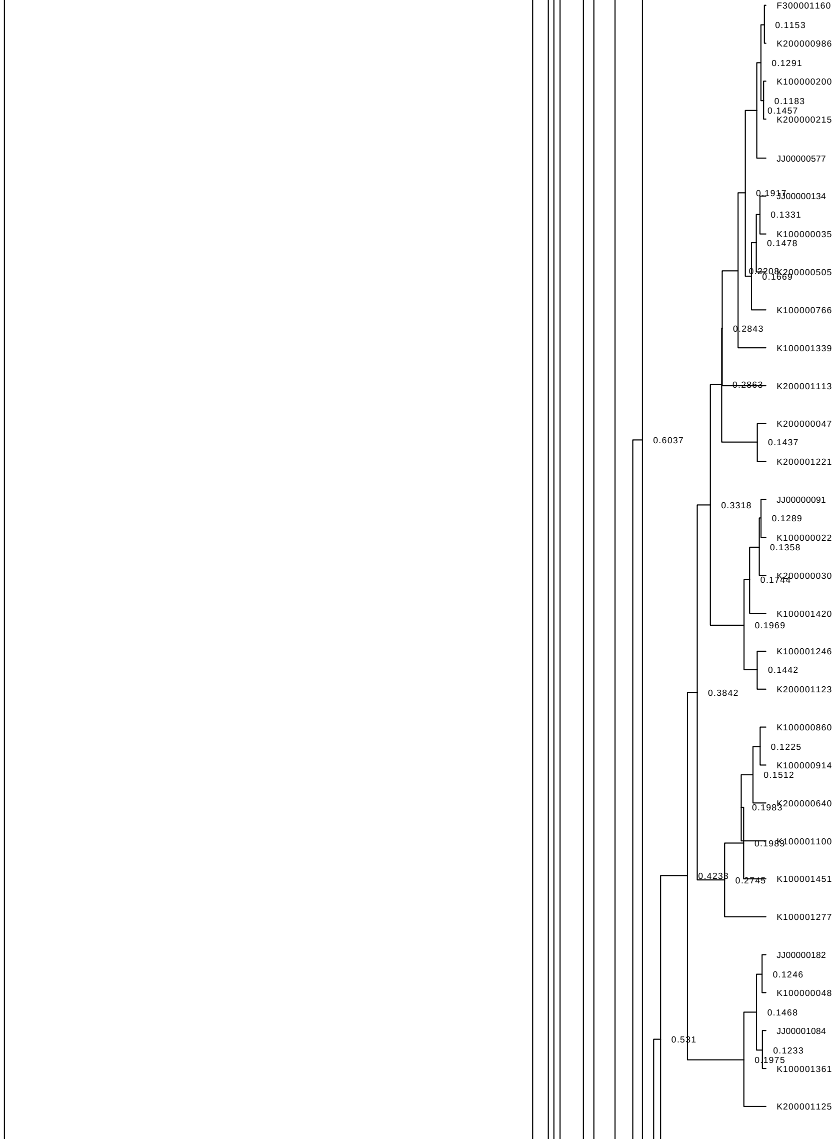
F300001160
0.1153
K200000986
0.1291
K100000200
0.1183
0.1457
K200000215
JJ00000577
0.1917
JJ00000134
0.1331
K100000035
0.1478
0.2208
K200000505
0.1669
K100000766
0.2843
K100001339
0.2863
K200001113
K200000047
0.1437
K200001221
0.6037
JJ00000091
0.3318
0.1289
K100000022
0.1358
K200000030
0.1744
K100001420
0.1969
K100001246
0.1442
K200001123
0.3842
K100000860
0.1225
K100000914
0.1512
K200000640
0.1983
K100001100
0.1983
0.4233
K100001451
0.2745
K100001277
JJ00000182
0.1246
K100000048
0.1468
JJ00001084
0.531
0.1233
0.1975
K100001361
K200001125

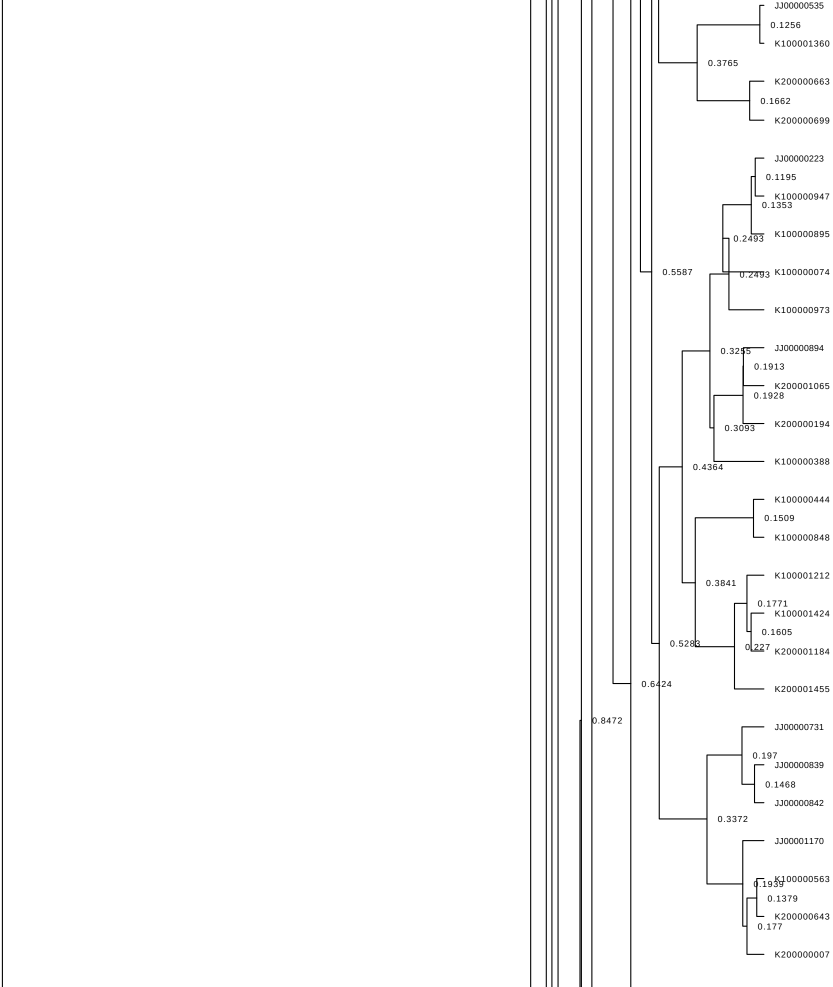
JJ00000535
0.1256
K100001360
0.3765
K200000663
0.1662
K200000699
JJ00000223
0.1195
K100000947
0.1353
K100000895
0.2493
K100000074
0.2493
0.5587
K100000973
JJ00000894
0.3255
0.1913
K200001065
0.1928
K200000194
0.3093
K100000388
0.4364
K100000444
0.1509
K100000848
K100001212
0.3841
0.1771
K100001424
0.1605
0.5283
0.227
K200001184
K200001455
0.6424
0.8472
JJ00000731
0.197
JJ00000839
0.1468
JJ00000842
0.3372
JJ00001170
K100000563
0.1939
0.1379
K200000643
0.177
K200000007

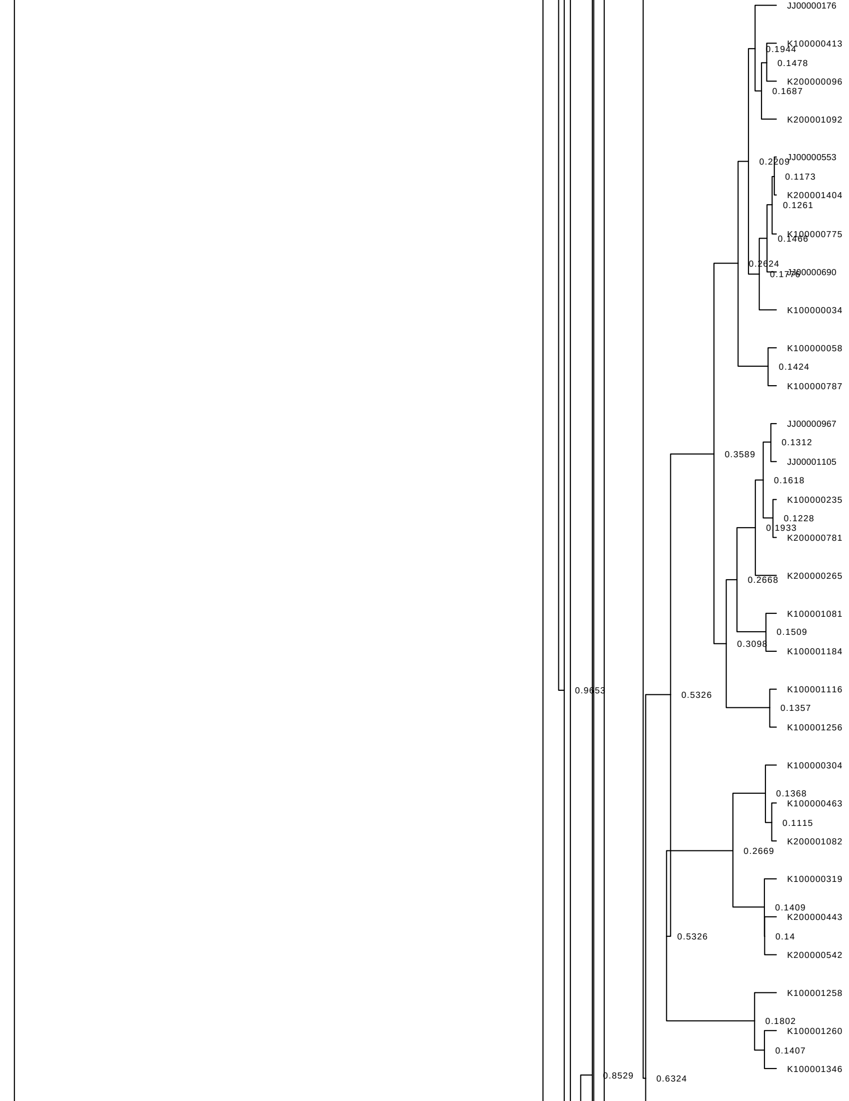

JJ00000176
K100000413
0.1944
0.1478
K200000096
0.1687
K200001092
JJ00000553
0.2209
0.1173
K200001404
0.1261
K100000775
0.1466
0.2624
JJ00000690
0.1776
K100000034
K100000058
0.1424
K100000787
JJ00000967
0.1312
0.3589
JJ00001105
0.1618
K100000235
0.1228
0.1933
K200000781
K200000265
0.2668
K100001081
0.1509
0.3098
K100001184
0.9653
0.5326
K100001116
0.1357
K100001256
K100000304
0.1368
K100000463
0.1115
K200001082
0.2669
K100000319
0.1409
K200000443
0.5326
0.14
K200000542
K100001258
0.1802
K100001260
0.1407
0.8529
0.6324
K100001346

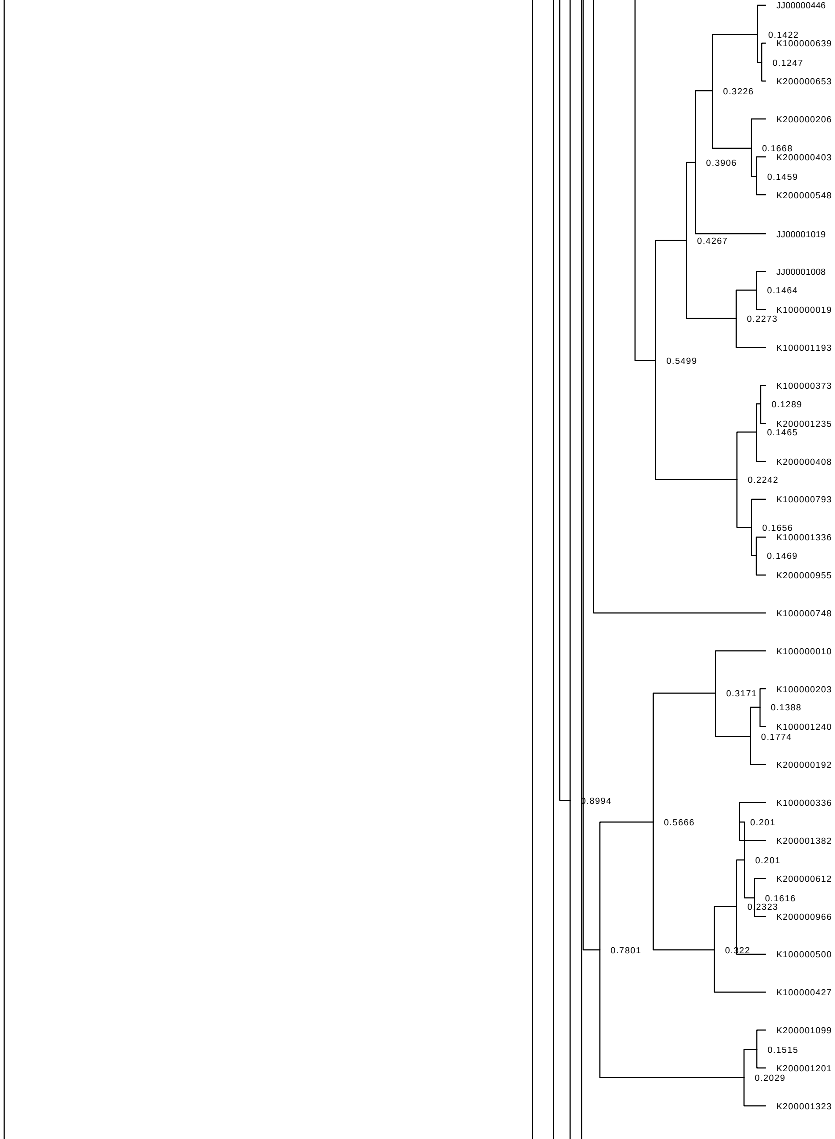
JJ00000446
0.1422
K100000639
0.1247
K200000653
0.3226
K200000206
0.1668
K200000403
0.3906
0.1459
K200000548
JJ00001019
0.4267
JJ00001008
0.1464
K100000019
0.2273
K100001193
0.5499
K100000373
0.1289
K200001235
0.1465
K200000408
0.2242
K100000793
0.1656
K100001336
0.1469
K200000955
K100000748
K100000010
K100000203
0.3171
0.1388
K100001240
0.1774
K200000192
0.8994
K100000336
0.5666
0.201
K200001382
0.201
K200000612
0.1616
0.2323
K200000966
0.7801
0.322
K100000500
K100000427
K200001099
0.1515
K200001201
0.2029
K200001323

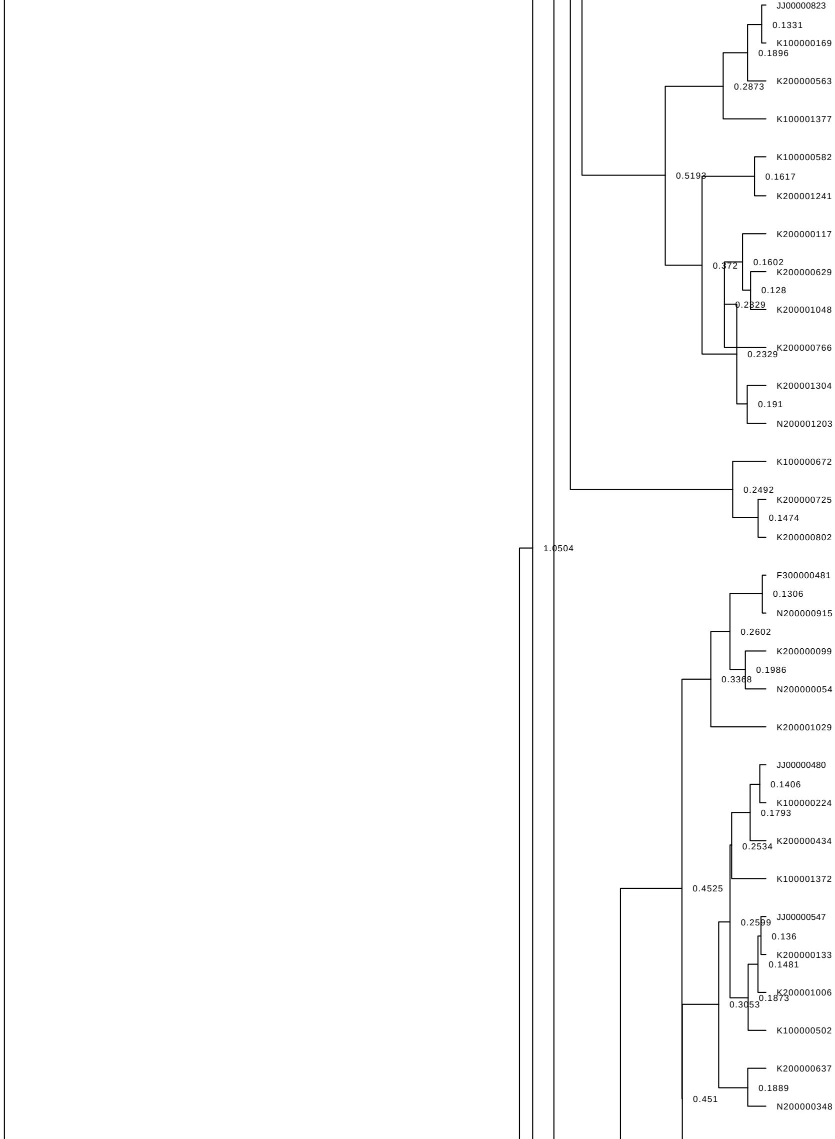

JJ00000823
0.1331
K100000169
0.1896
K200000563
0.2873
K100001377
K100000582
0.5193
0.1617
K200001241
K200000117
0.1602
0.372
K200000629
0.128
0.2329
K200001048
K200000766
0.2329
K200001304
0.191
N200001203
K100000672
0.2492
K200000725
0.1474
K200000802
1.0504
F300000481
0.1306
N200000915
0.2602
K200000099
0.1986
0.3368
N200000054
K200001029
JJ00000480
0.1406
K100000224
0.1793
K200000434
0.2534
K100001372
0.4525
JJ00000547
0.2599
0.136
K200000133
0.1481
K200001006
0.1873
0.3053
K100000502
K200000637
0.1889
0.451
N200000348

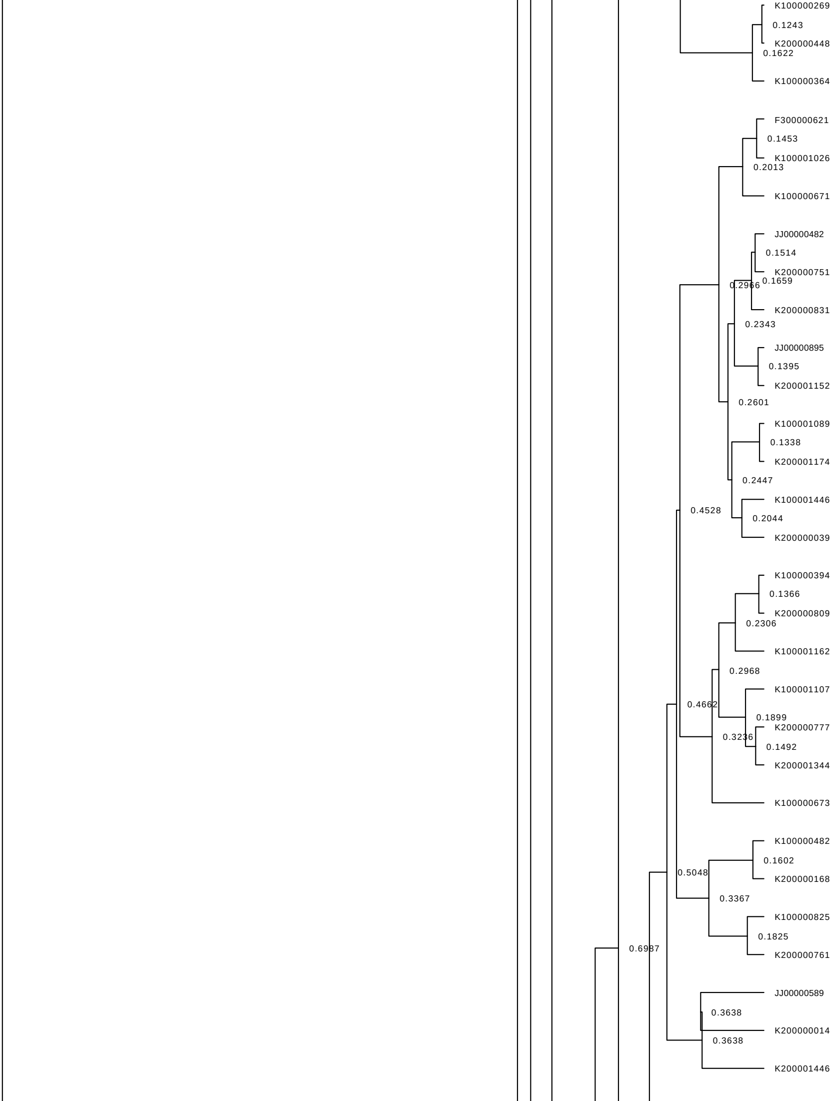
K100000269
0.1243
K200000448
0.1622
K100000364
F300000621
0.1453
K100001026
0.2013
K100000671
JJ00000482
0.1514
K200000751
0.1659
0.2966
K200000831
0.2343
JJ00000895
0.1395
K200001152
0.2601
K100001089
0.1338
K200001174
0.2447
K100001446
0.4528
0.2044
K200000039
K100000394
0.1366
K200000809
0.2306
K100001162
0.2968
K100001107
0.4662
0.1899
K200000777
0.3236
0.1492
K200001344
K100000673
K100000482
0.1602
0.5048
K200000168
0.3367
K100000825
0.1825
0.6987
K200000761
JJ00000589
0.3638
K200000014
0.3638
K200001446

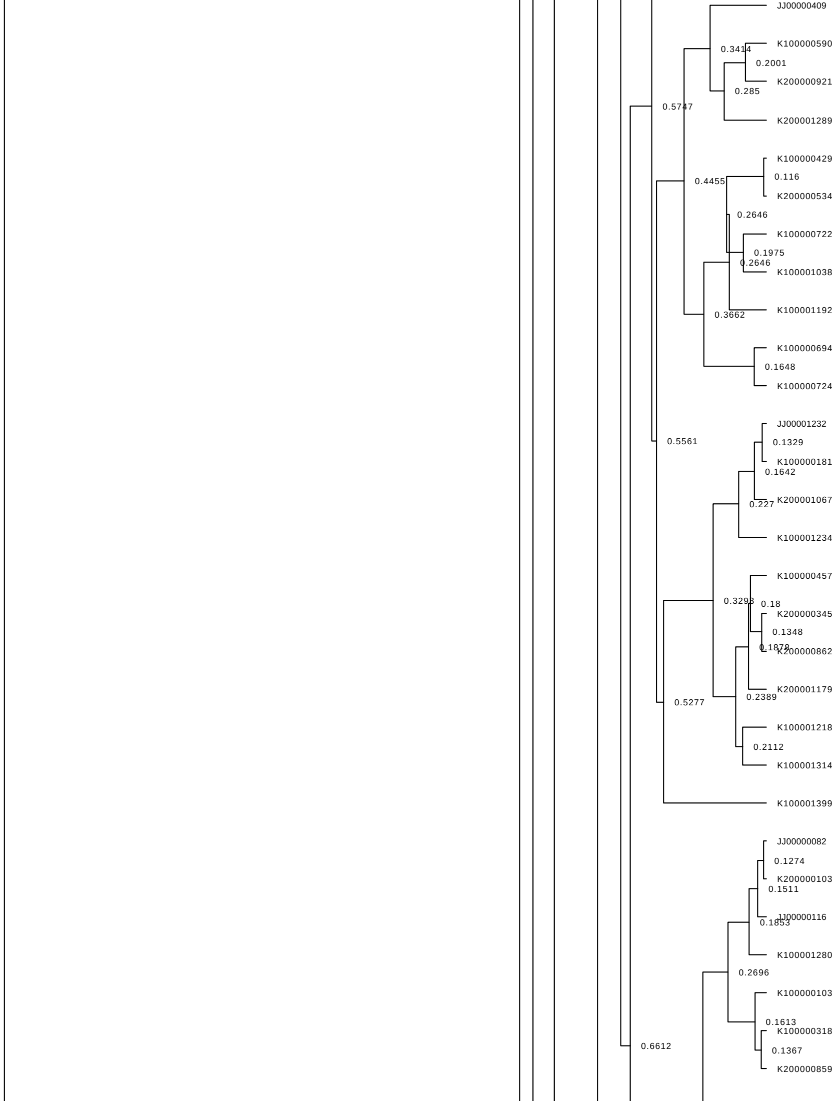

JJ00000409
0.3414
K100000590
0.2001
K200000921
0.285
0.5747
K200001289
K100000429
0.116
0.4455
K200000534
0.2646
K100000722
0.1975
0.2646
K100001038
K100001192
0.3662
K100000694
0.1648
K100000724
JJ00001232
0.1329
0.5561
K100000181
0.1642
K200001067
0.227
K100001234
K100000457
0.3293
0.18
K200000345
0.1348
0.1878
K200000862
K200001179
0.2389
0.5277
K100001218
0.2112
K100001314
K100001399
JJ00000082
0.1274
K200000103
0.1511
JJ00000116
0.1853
K100001280
0.2696
K100000103
0.1613
K100000318
0.6612
0.1367
K200000859

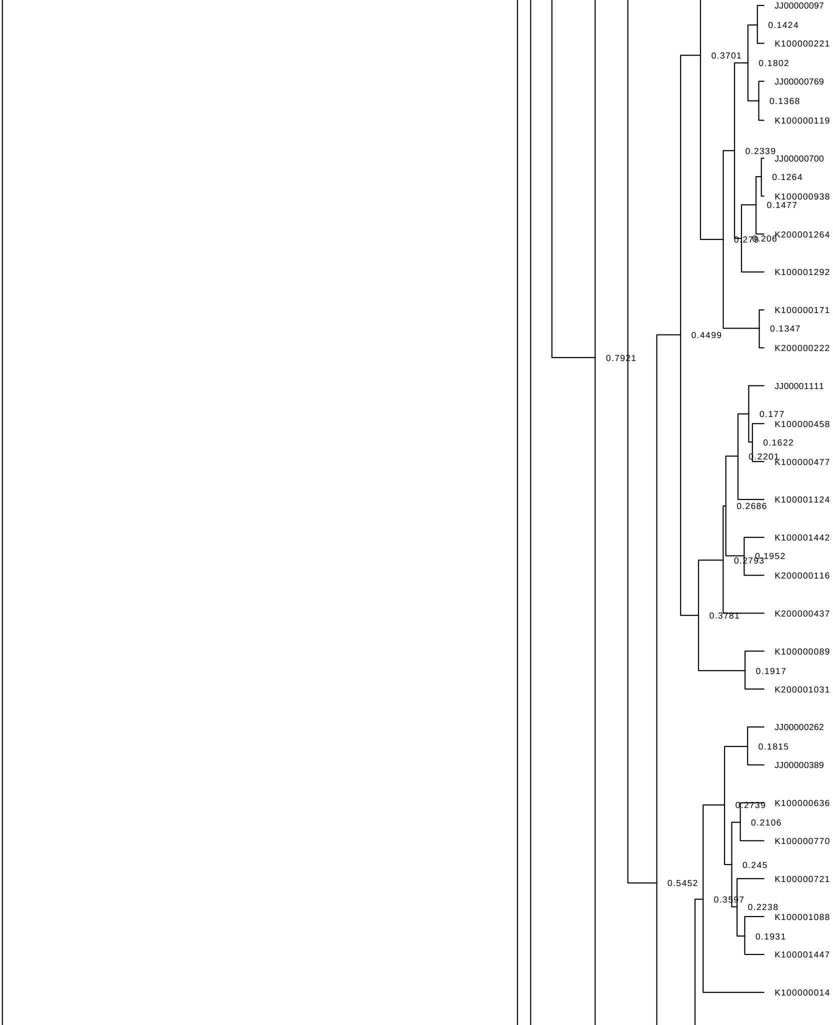
JJ00000097
0.1424
K100000221
0.3701
0.1802
JJ00000769
0.1368
K100000119
0.2339
JJ00000700
0.1264
K100000938
0.1477
K200001264
0.206
K100001292
K100000171
0.1347
0.4499
K200000222
0.7921
JJ00001111
0.177
K100000458
0.1622
0.2201
K100000477
K100001124
0.2686
K100001442
0.1952
0.2793
K200000116
K200000437
0.3781
K100000089
0.1917
K200001031
JJ00000262
0.1815
JJ00000389
0.2739
K100000636
0.2106
K100000770
0.245
K100000721
0.5452
0.3597
0.2238
K100001088
0.1931
K100001447
K100000014

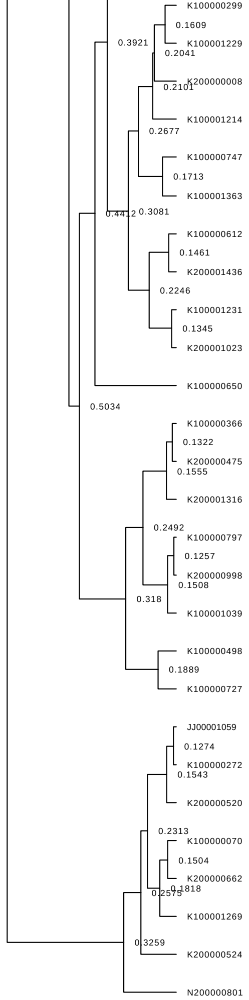

K100000299
0.1609
K100001229
0.3921
0.2041
K200000008
0.2101
K100001214
0.2677
K100000747
0.1713
K100001363
0.4412
0.3081
K100000612
0.1461
K200001436
0.2246
K100001231
0.1345
K200001023
K100000650
0.5034
K100000366
0.1322
K200000475
0.1555
K200001316
0.2492
K100000797
0.1257
K200000998
0.1508
0.318
K100001039
K100000498
0.1889
K100000727
JJ00001059
0.1274
K100000272
0.1543
K200000520
0.2313
K100000070
0.1504
K200000662
0.1818
0.2575
K100001269
0.3259
K200000524
N200000801

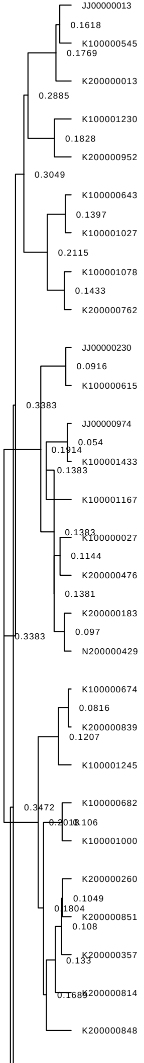

JJ00000013
0.1618
K100000545
0.1769
K200000013
0.2885
K100001230
0.1828
K200000952
0.3049
K100000643
0.1397
K100001027
0.2115
K100001078
0.1433
K200000762
JJ00000230
0.0916
K100000615
0.3383
JJ00000974
0.054
0.1914
K100001433
0.1383
K100001167
0.1383
K100000027
0.1144
K200000476
0.1381
K200000183
0.097
0.3383
N200000429
K100000674
0.0816
K200000839
0.1207
K100001245
0.3472
K100000682
0.106
K100001000
K200000260
0.1049
K200000851
0.108
K200000357
0.133
K200000814
K200000848

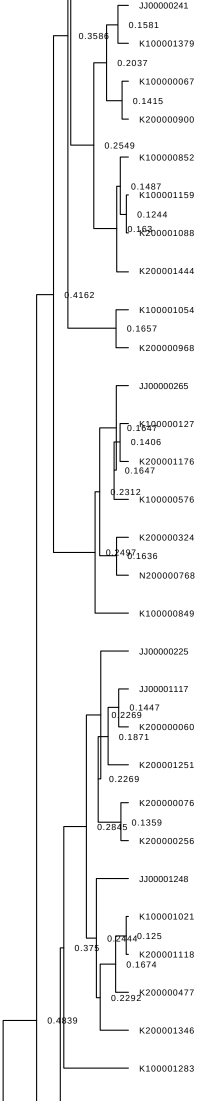
JJ00000241
0.1581
0.3586
K100001379
0.2037
K100000067
0.1415
K200000900
0.2549
K100000852
0.1487
K100001159
0.1244
0.163
K200001088
K200001444
0.4162
K100001054
0.1657
K200000968
JJ00000265
K100000127
0.1647
0.1406
K200001176
0.1647
0.2312
K100000576
K200000324
0.2497
0.1636
N200000768
K100000849
JJ00000225
JJ00001117
0.1447
0.2269
K200000060
0.1871
K200001251
0.2269
K200000076
0.1359
0.2845
K200000256
JJ00001248
K100001021
0.2444
0.125
K200001118
0.375
0.1674
K200000477
0.2292
0.4839
K200001346
K100001283

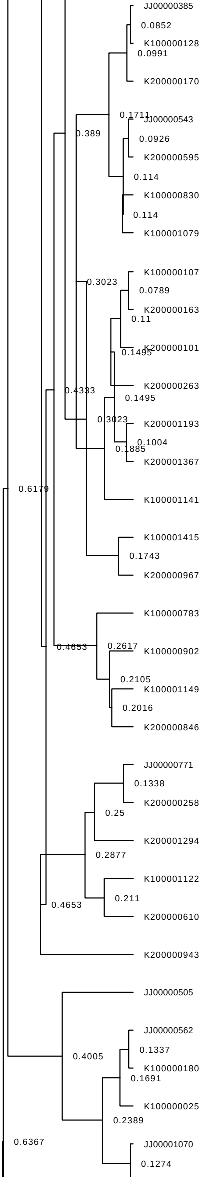
JJ00000385
0.0852
K100000128
0.0991
K200000170
0.1711
JJ00000543
0.389
0.0926
K200000595
0.114
K100000830
0.114
K100001079
K100000107
0.3023
0.0789
K200000163
0.11
K200000101
0.1495
K200000263
0.4333
0.1495
0.3023
K200001193
0.1004
0.1885
K200001367
0.6179
K100001141
K100001415
0.1743
K200000967
K100000783
0.2617
0.4653
K100000902
0.2105
K100001149
0.2016
K200000846
JJ00000771
0.1338
K200000258
0.25
K200001294
0.2877
K100001122
0.211
0.4653
K200000610
K200000943
JJ00000505
JJ00000562
0.1337
0.4005
K100000180
0.1691
K100000025
0.2389
0.6367
JJ00001070
0.1274

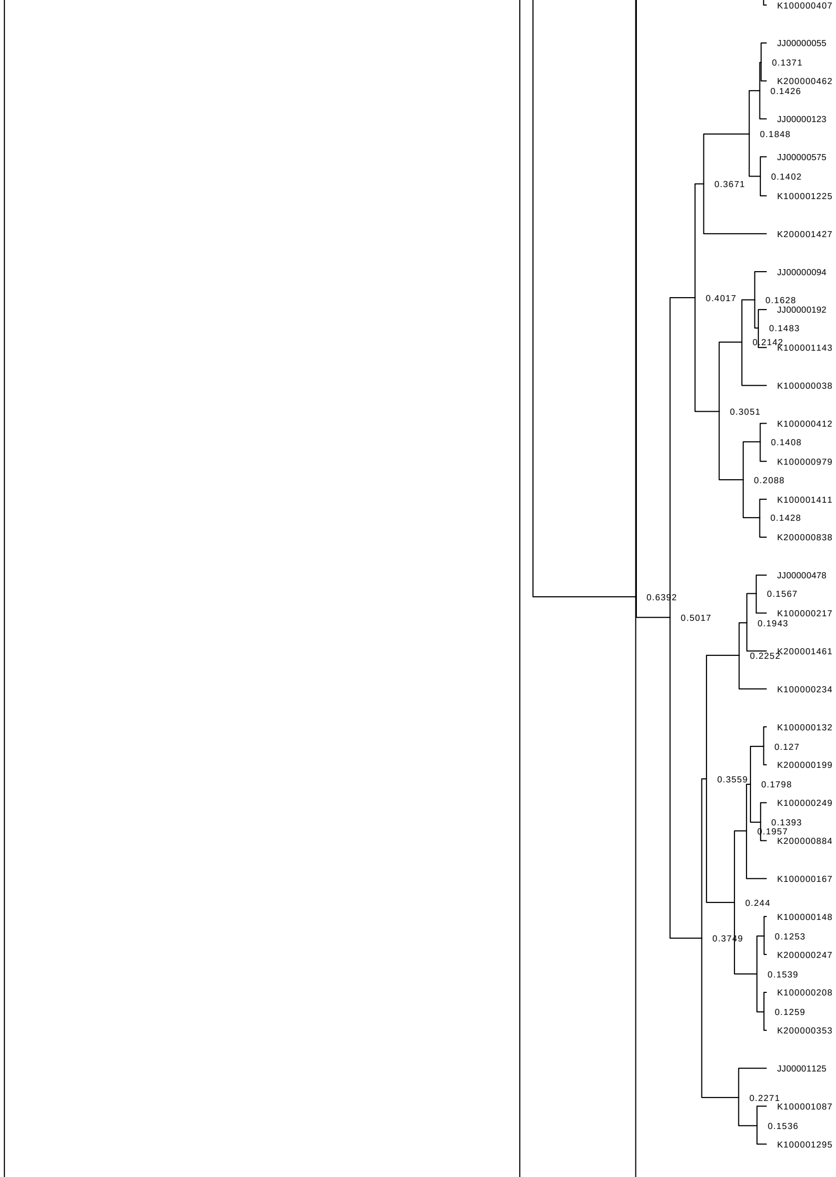

K100000407
JJ00000055
0.1371
K200000462
0.1426
JJ00000123
0.1848
JJ00000575
0.1402
K100001225
0.3671
K200001427
JJ00000094
0.4017
0.1628
JJ00000192
0.1483
0.2142
K100001143
K100000038
0.3051
K100000412
0.1408
K100000979
0.2088
K100001411
0.1428
K200000838
JJ00000478
0.1567
0.6392
K100000217
0.5017
0.1943
K200001461
0.2252
K100000234
K100000132
0.127
K200000199
0.3559
0.1798
K100000249
0.1393
0.1957
K200000884
K100000167
0.244
K100000148
0.1253
0.3749
K200000247
0.1539
K100000208
0.1259
K200000353
JJ00001125
0.2271
K100001087
0.1536
K100001295

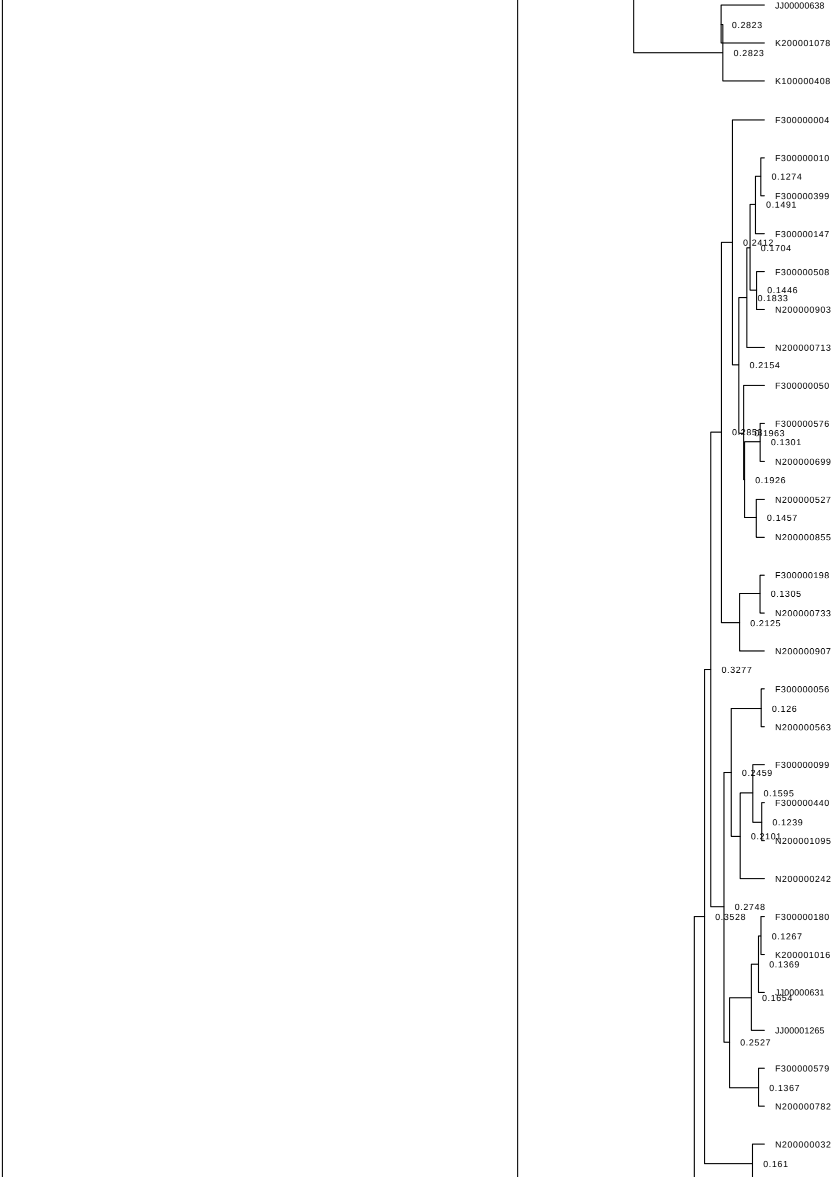
JJ00000638
0.2823
K200001078
0.2823
K100000408
F300000004
F300000010
0.1274
F300000399
0.1491
F300000147
0.2412
0.1704
F300000508
0.1446
0.1833
N200000903
N200000713
0.2154
F300000050
F300000576
0.285
0.1963
0.1301
N200000699
0.1926
N200000527
0.1457
N200000855
F300000198
0.1305
N200000733
0.2125
N200000907
0.3277
F300000056
0.126
N200000563
F300000099
0.2459
0.1595
F300000440
0.1239
0.2101
N200001095
N200000242
0.2748
0.3528
F300000180
0.1267
K200001016
0.1369
JJ00000631
0.1654
JJ00001265
0.2527
F300000579
0.1367
N200000782
N200000032
0.161

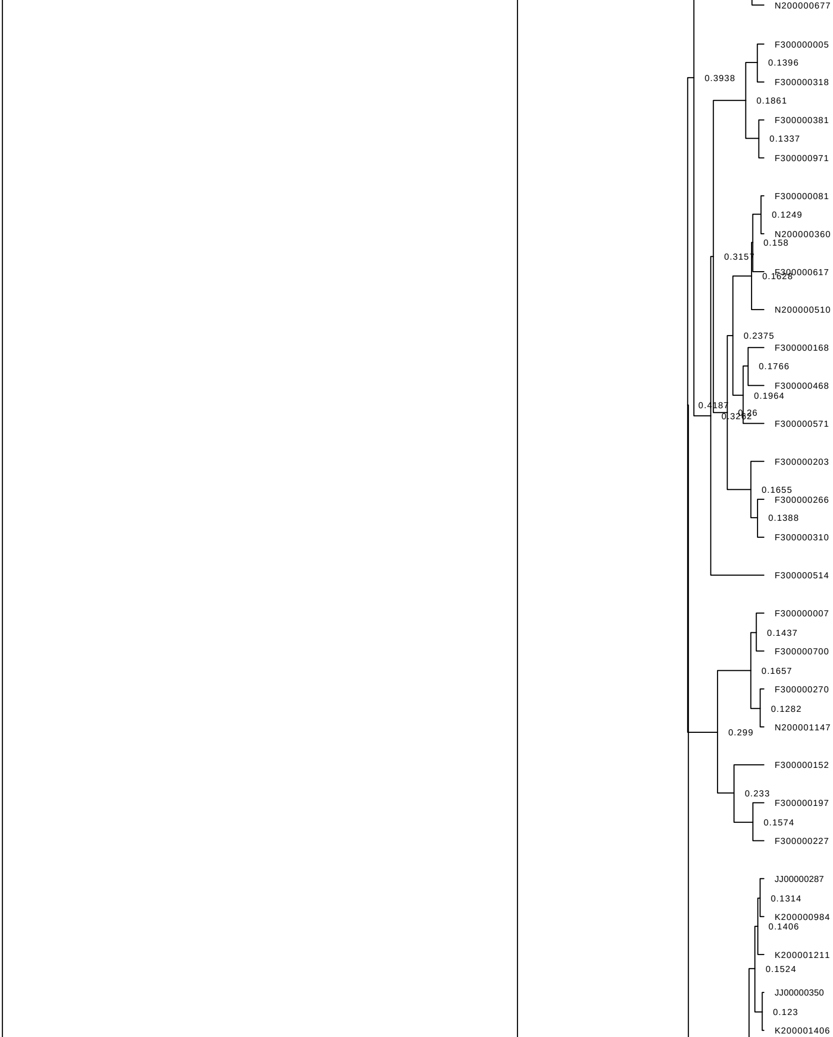
N200000677
F300000005
0.1396
0.3938
F300000318
0.1861
F300000381
0.1337
F300000971
F300000081
0.1249
N200000360
0.158
0.3157
F300000617
0.1628
N200000510
0.2375
F300000168
0.1766
F300000468
0.1964
0.4187
0.3262
0.26
F300000571
F300000203
0.1655
F300000266
0.1388
F300000310
F300000514
F300000007
0.1437
F300000700
0.1657
F300000270
0.1282
N200001147
0.299
F300000152
0.233
F300000197
0.1574
F300000227
JJ00000287
0.1314
K200000984
0.1406
K200001211
0.1524
JJ00000350
0.123
K200001406

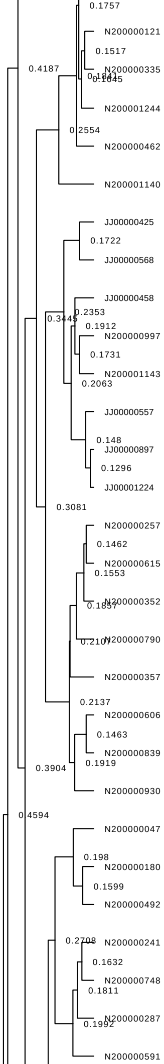
0.1757
N200000121
0.1517
0.4187
N200000335
0.1841
0.1645
N200001244
0.2554
N200000462
N200001140
JJ00000425
0.1722
JJ00000568
JJ00000458
0.2353
0.3445
0.1912
N200000997
0.1731
N200001143
0.2063
JJ00000557
0.148
JJ00000897
0.1296
JJ00001224
0.3081
N200000257
0.1462
N200000615
0.1553
N200000352
0.1857
N200000790
0.2107
N200000357
0.2137
N200000606
0.1463
N200000839
0.1919
0.3904
N200000930
0.4594
N200000047
0.198
N200000180
0.1599
N200000492
0.2708
N200000241
0.1632
N200000748
0.1811
N200000287
0.1992
N200000591

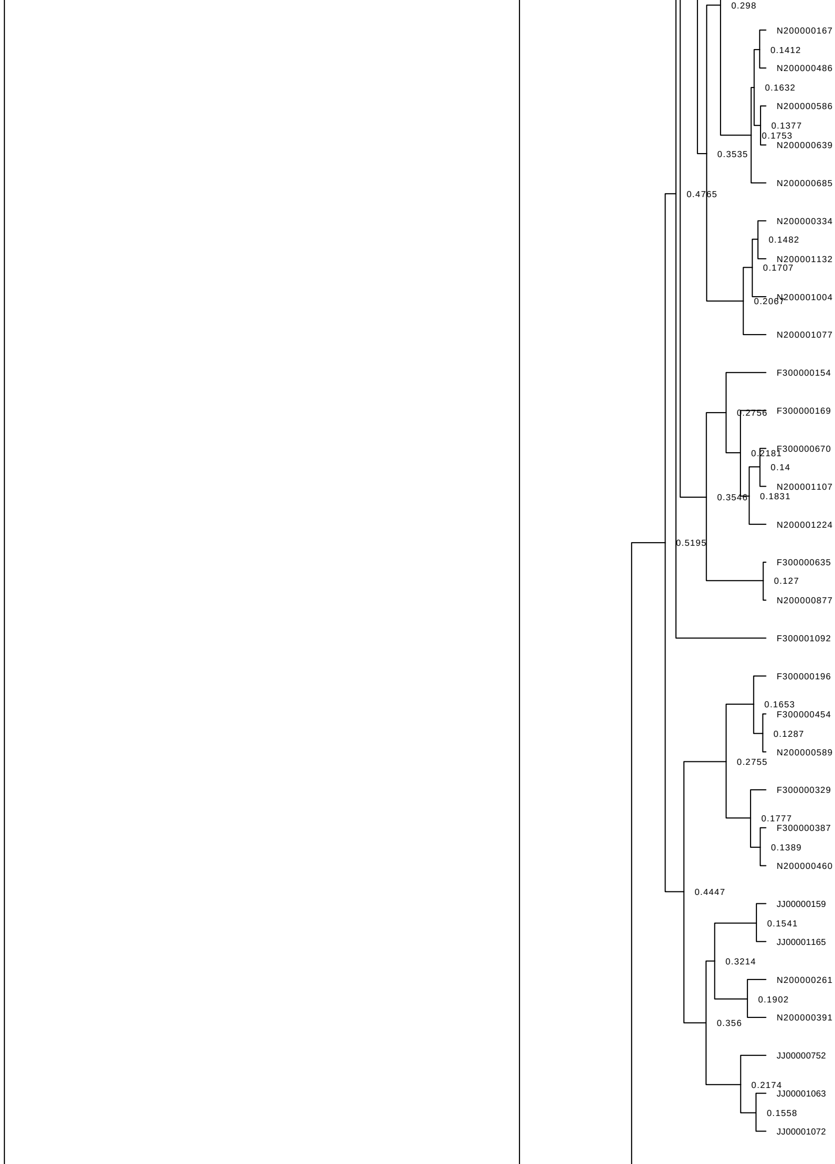
0.298
N200000167
0.1412
N200000486
0.1632
N200000586
0.1377
0.1753
N200000639
0.3535
N200000685
0.4765
N200000334
0.1482
N200001132
0.1707
N200001004
0.2067
N200001077
F300000154
0.2756
F300000169
0.2181
F300000670
0.14
N200001107
0.3546
0.1831
N200001224
0.5195
F300000635
0.127
N200000877
F300001092
F300000196
0.1653
F300000454
0.1287
N200000589
0.2755
F300000329
0.1777
F300000387
0.1389
N200000460
0.4447
JJ00000159
0.1541
JJ00001165
0.3214
N200000261
0.1902
N200000391
0.356
JJ00000752
0.2174
JJ00001063
0.1558
JJ00001072

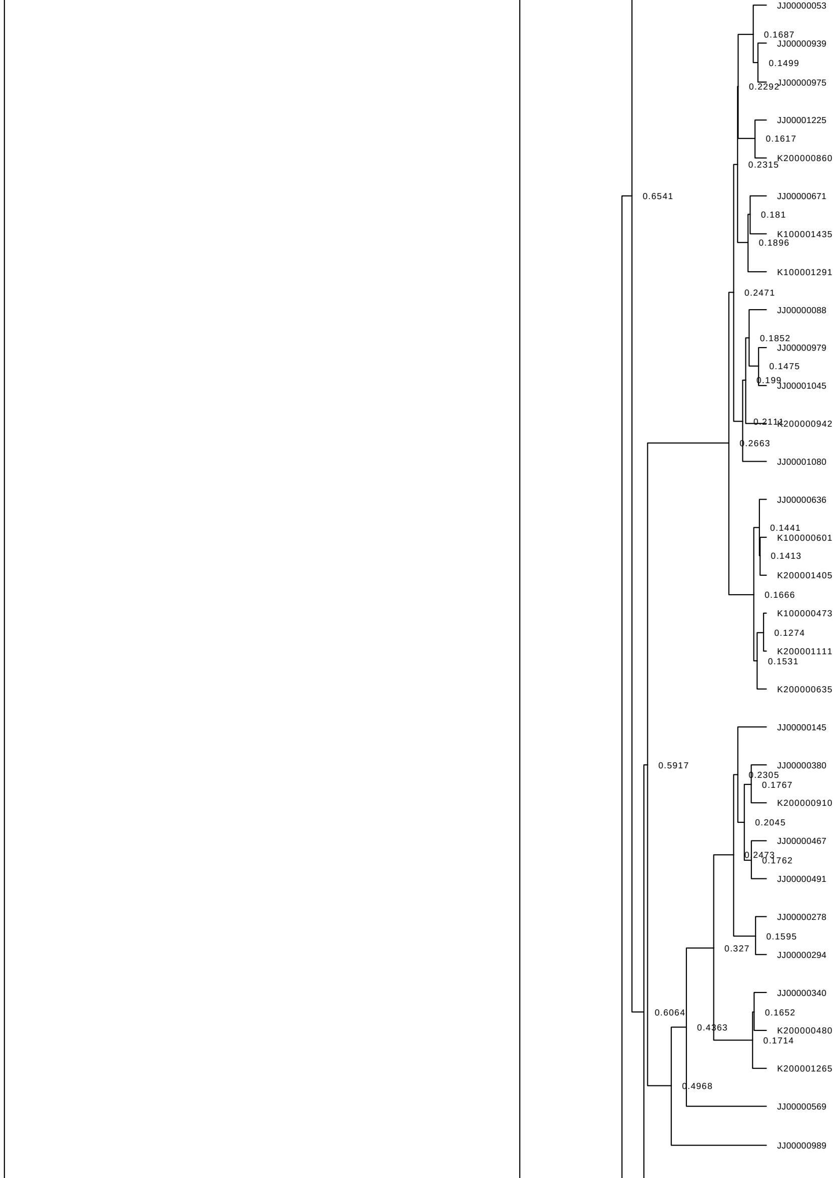
JJ00000053
0.1687
JJ00000939
0.1499
JJ00000975
0.2292
JJ00001225
0.1617
K200000860
0.2315
0.6541
JJ00000671
0.181
K100001435
0.1896
K100001291
0.2471
JJ00000088
0.1852
JJ00000979
0.1475
0.199
JJ00001045
0.2111
K200000942
0.2663
JJ00001080
JJ00000636
0.1441
K100000601
0.1413
K200001405
0.1666
K100000473
0.1274
K200001111
0.1531
K200000635
JJ00000145
0.5917
JJ00000380
0.2305
0.1767
K200000910
0.2045
JJ00000467
0.2473
0.1762
JJ00000491
JJ00000278
0.1595
0.327
JJ00000294
JJ00000340
0.1652
0.6064
0.4363
K200000480
0.1714
K200001265
0.4968
JJ00000569
JJ00000989

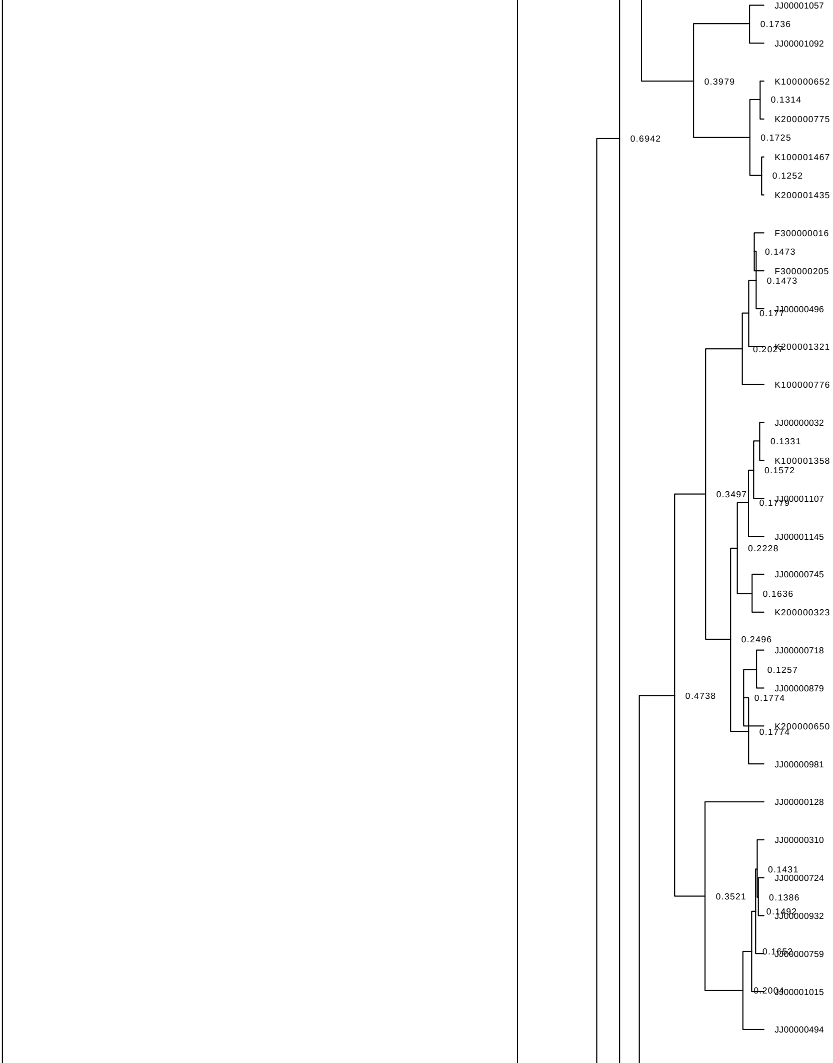

JJ00001057
0.1736
JJ00001092
0.3979
K100000652
0.1314
K200000775
0.6942
0.1725
K100001467
0.1252
K200001435
F300000016
0.1473
F300000205
0.1473
JJ00000496
0.177
K200001321
0.2027
K100000776
JJ00000032
0.1331
K100001358
0.1572
0.3497
JJ00001107
0.1779
JJ00001145
0.2228
JJ00000745
0.1636
K200000323
0.2496
JJ00000718
0.1257
JJ00000879
0.4738
0.1774
K200000650
0.1774
JJ00000981
JJ00000128
JJ00000310
0.1431
JJ00000724
0.3521
0.1386
0.1492
JJ00000932
0.1652
JJ00000759
0.2004
JJ00001015
JJ00000494

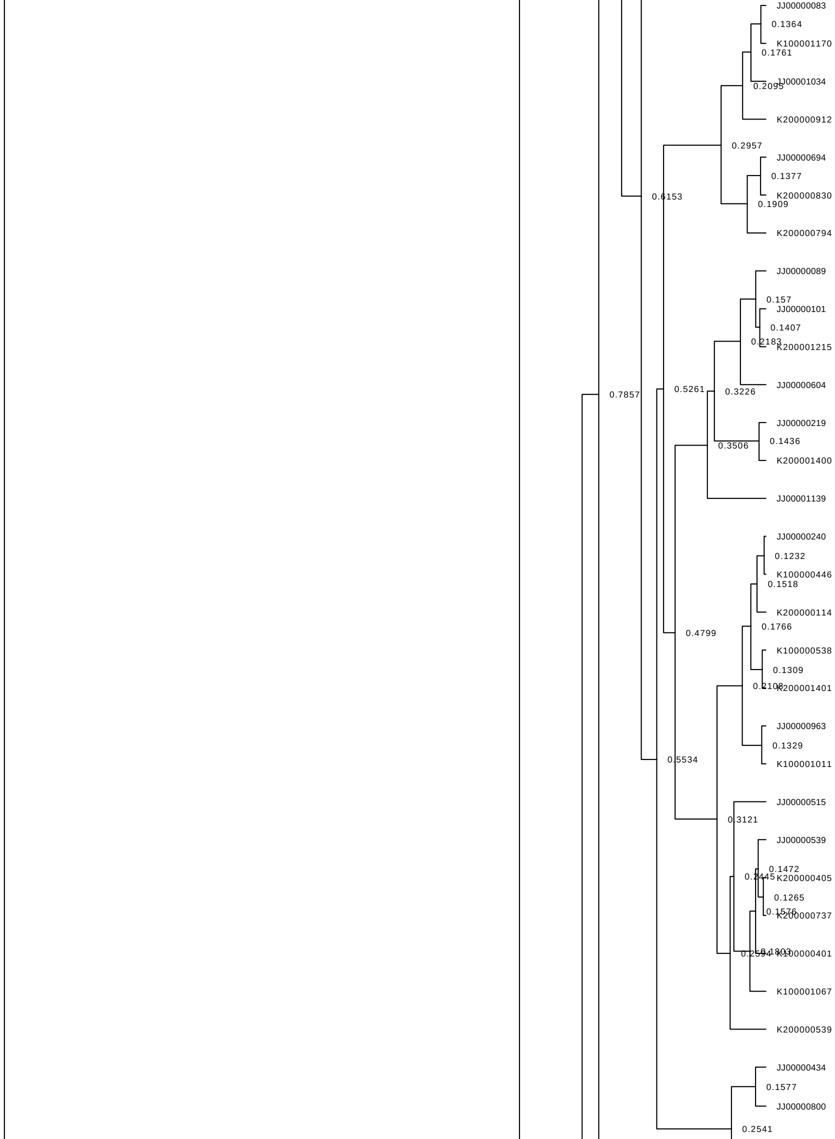
JJ00000083
0.1364
K100001170
0.1761
JJ00001034
0.2095
K200000912
0.2957
JJ00000694
0.1377
K200000830
0.6153
0.1909
K200000794
JJ00000089
0.157
JJ00000101
0.1407
0.2183
K200001215
JJ00000604
0.7857
0.5261
0.3226
JJ00000219
0.1436
0.3506
K200001400
JJ00001139
JJ00000240
0.1232
K100000446
0.1518
K200000114
0.1766
0.4799
K100000538
0.1309
0.2108
K200001401
JJ00000963
0.1329
0.5534
K100001011
JJ00000515
0.3121
JJ00000539
0.1472
0.2445
K200000405
0.1265
0.1576
K200000737
0.1803
0.2594
K100000401
K100001067
K200000539
JJ00000434
0.1577
JJ00000800
0.2541

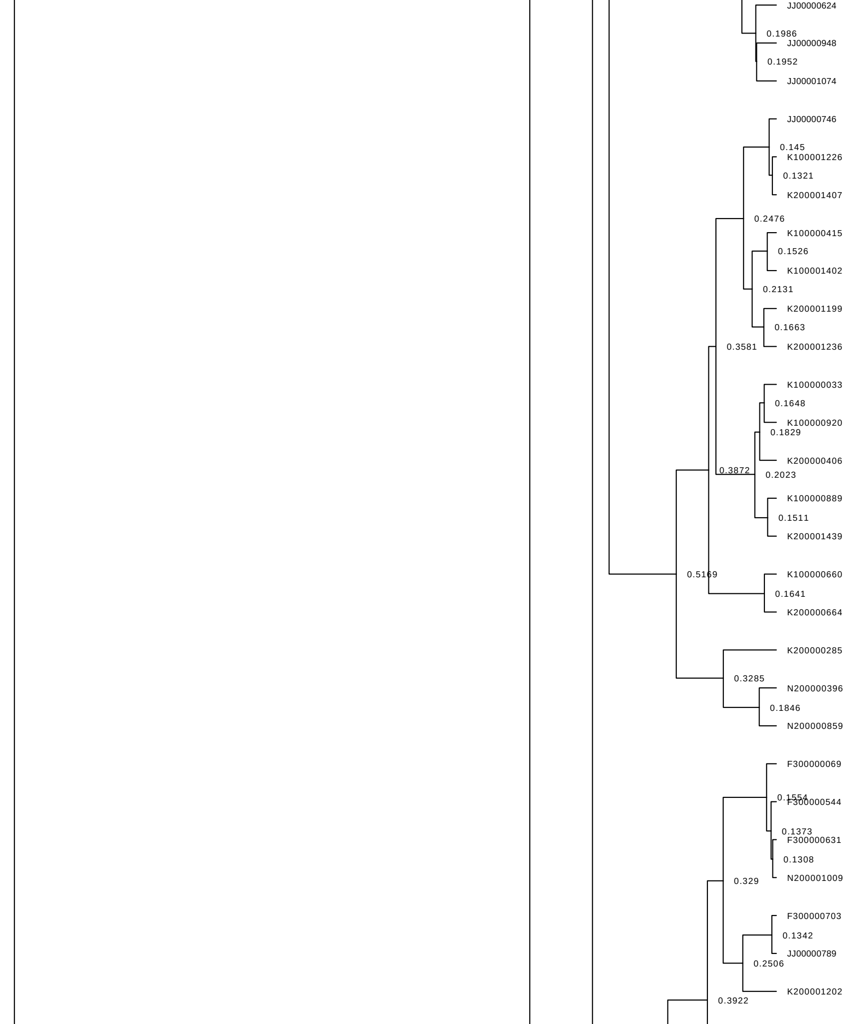
JJ00000624
0.1986
JJ00000948
0.1952
JJ00001074
JJ00000746
0.145
K100001226
0.1321
K200001407
0.2476
K100000415
0.1526
K100001402
0.2131
K200001199
0.1663
0.3581
K200001236
K100000033
0.1648
K100000920
0.1829
K200000406
0.3872
0.2023
K100000889
0.1511
K200001439
K100000660
0.5169
0.1641
K200000664
K200000285
0.3285
N200000396
0.1846
N200000859
F300000069
0.1554
F300000544
0.1373
F300000631
0.1308
N200001009
0.329
F300000703
0.1342
JJ00000789
0.2506
K200001202
0.3922

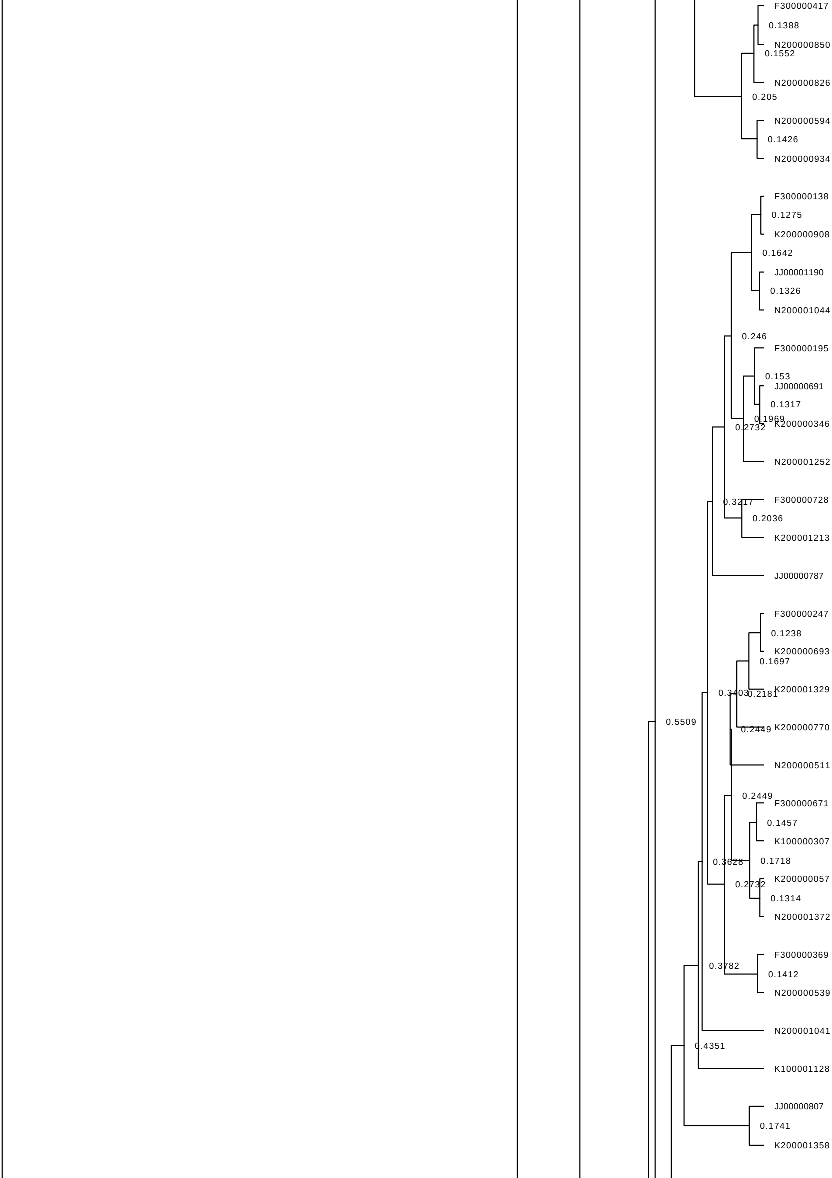
F300000417
0.1388
N200000850
0.1552
N200000826
0.205
N200000594
0.1426
N200000934
F300000138
0.1275
K200000908
0.1642
JJ00001190
0.1326
N200001044
0.246
F300000195
0.153
JJ00000691
0.1317
0.1969
0.2732
K200000346
N200001252
0.3217
F300000728
0.2036
K200001213
JJ00000787
F300000247
0.1238
K200000693
0.1697
0.3403
0.2181
K200001329
0.5509
0.2449
K200000770
N200000511
0.2449
F300000671
0.1457
K100000307
0.1718
0.3628
0.2732
K200000057
0.1314
N200001372
F300000369
0.3782
0.1412
N200000539
N200001041
0.4351
K100001128
JJ00000807
0.1741
K200001358

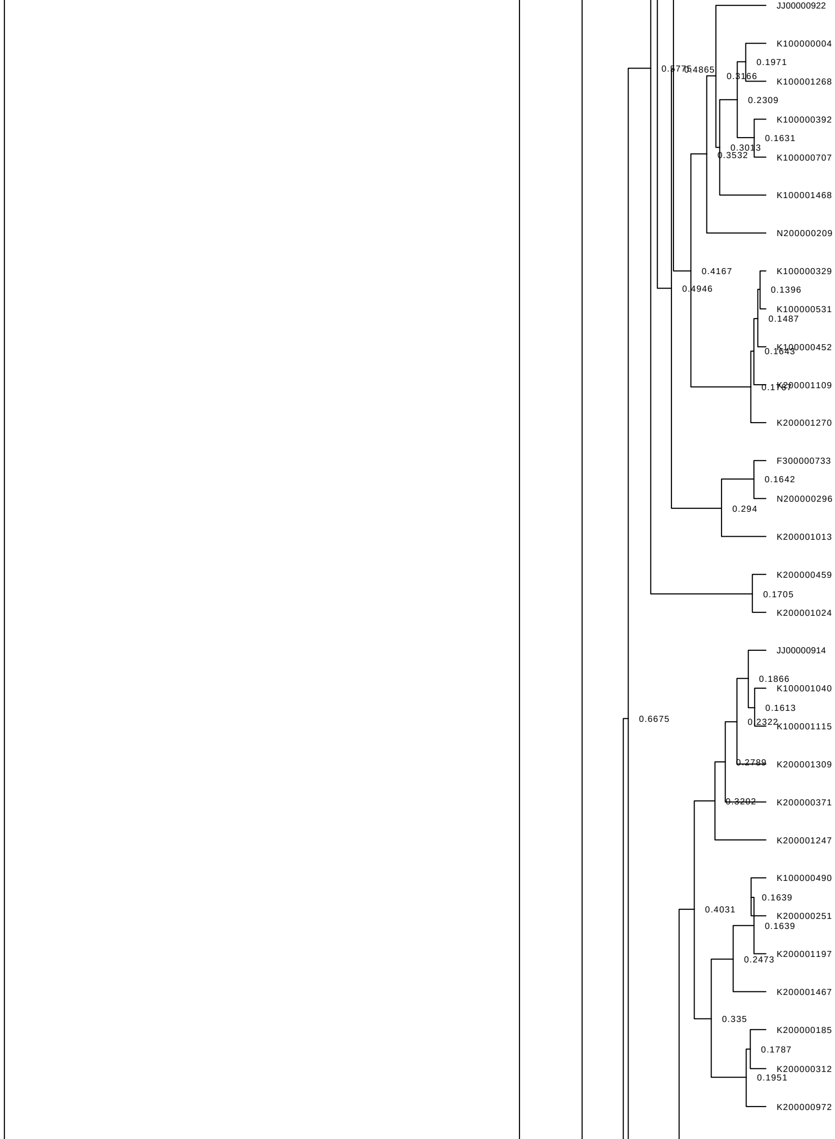

JJ00000922
K100000004
0.1971
0.5775
0.4865
0.3166
K100001268
0.2309
K100000392
0.1631
0.3013
0.3532
K100000707
K100001468
N200000209
0.4167
K100000329
0.4946
0.1396
K100000531
0.1487
K100000452
0.1643
K200001109
0.1767
K200001270
F300000733
0.1642
N200000296
0.294
K200001013
K200000459
0.1705
K200001024
JJ00000914
0.1866
K100001040
0.1613
0.6675
0.2322
K100001115
0.2789
K200001309
0.3202
K200000371
K200001247
K100000490
0.1639
0.4031
K200000251
0.1639
K200001197
0.2473
K200001467
0.335
K200000185
0.1787
K200000312
0.1951
K200000972

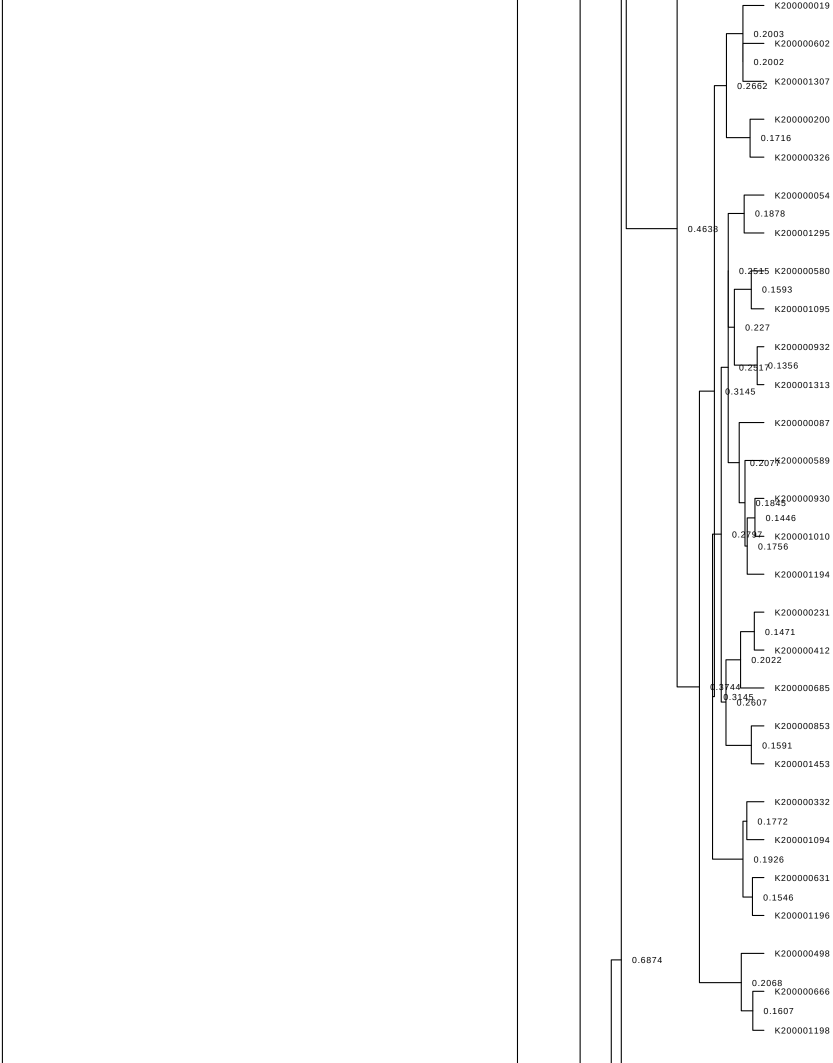

K200000019
0.2003
K200000602
0.2002
K200001307
0.2662
K200000200
0.1716
K200000326
K200000054
0.1878
0.4638
K200001295
0.2515
K200000580
0.1593
K200001095
0.227
K200000932
0.2517
0.1356
K200001313
0.3145
K200000087
0.2077
K200000589
K200000930
0.1845
0.1446
0.2797
K200001010
0.1756
K200001194
K200000231
0.1471
K200000412
0.2022
0.3744
K200000685
0.3145
0.2607
K200000853
0.1591
K200001453
K200000332
0.1772
K200001094
0.1926
K200000631
0.1546
K200001196
K200000498
0.6874
0.2068
K200000666
0.1607
K200001198

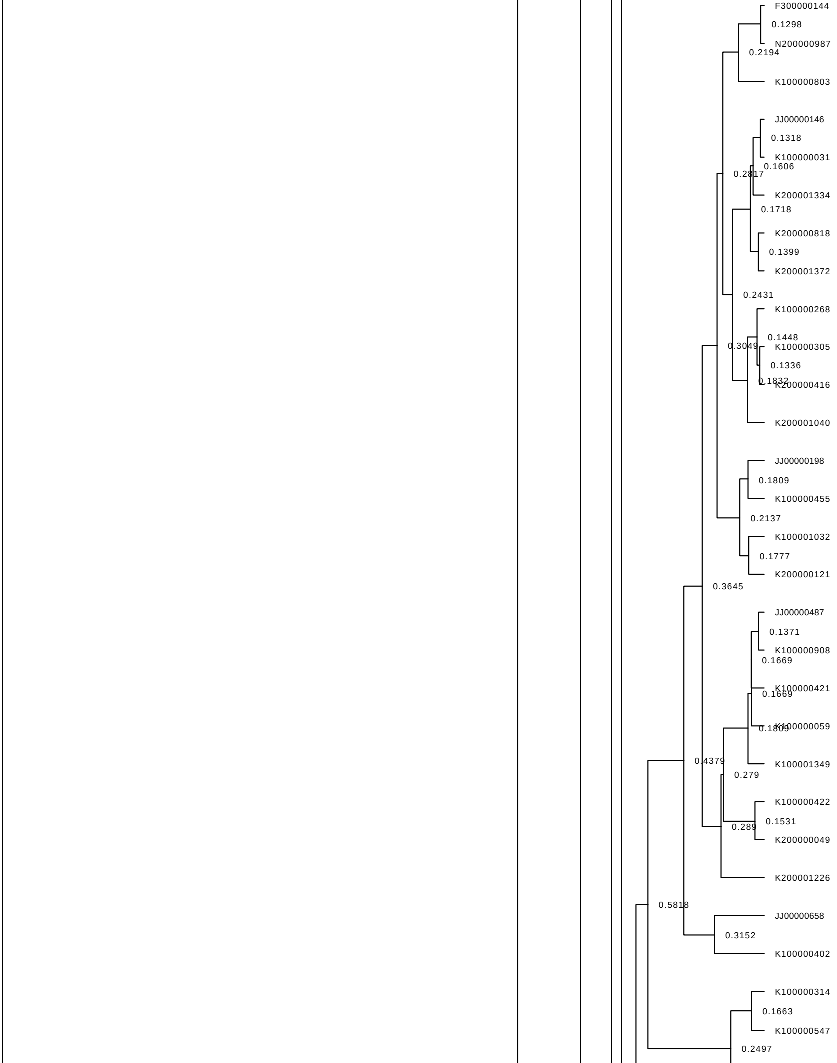
F300000144
0.1298
N200000987
0.2194
K100000803
JJ00000146
0.1318
K100000031
0.1606
0.2817
K200001334
0.1718
K200000818
0.1399
K200001372
0.2431
K100000268
0.1448
0.3049
K100000305
0.1336
0.1832
K200000416
K200001040
JJ00000198
0.1809
K100000455
0.2137
K100001032
0.1777
K200000121
0.3645
JJ00000487
0.1371
K100000908
0.1669
K100000421
0.1669
K100000059
0.1809
0.4379
K100001349
0.279
K100000422
0.1531
0.289
K200000049
K200001226
0.5818
JJ00000658
0.3152
K100000402
K100000314
0.1663
K100000547
0.2497

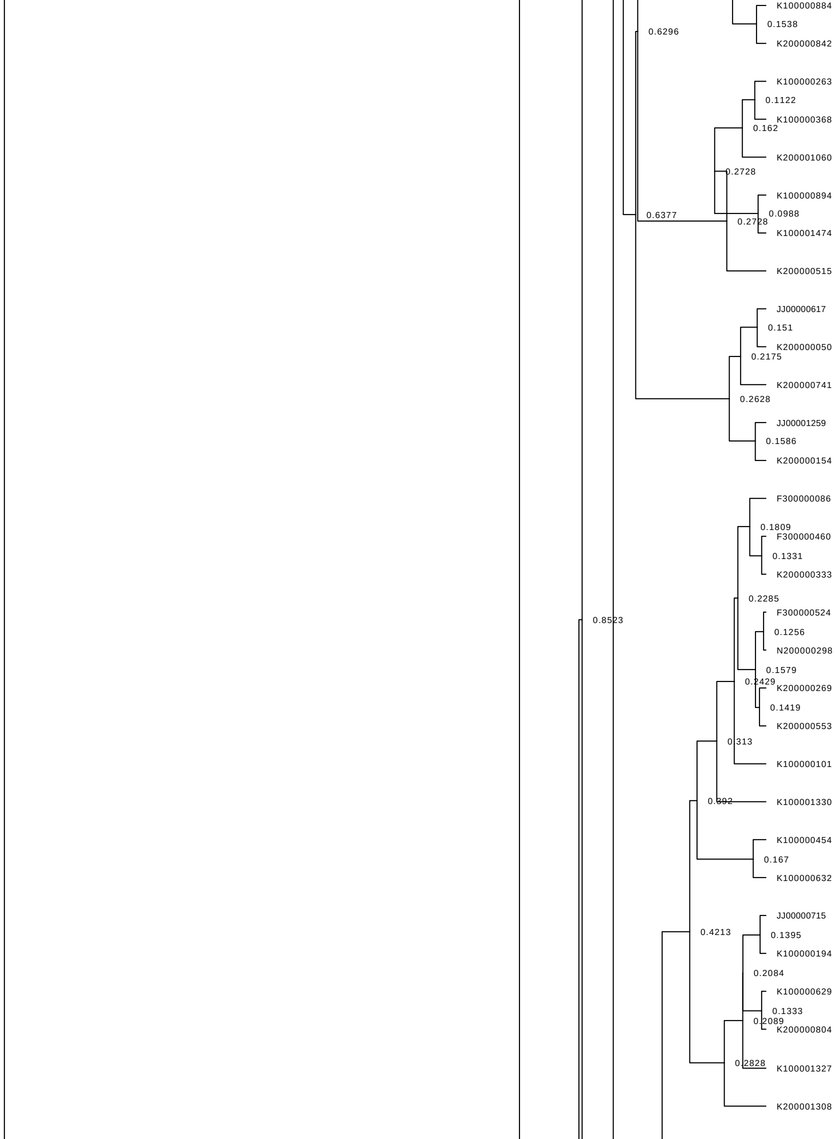
K100000884
0.1538
K200000842
0.6296
K100000263
0.1122
K100000368
0.162
K200001060
0.2728
K100000894
0.0988
0.6377
0.2728
K100001474
K200000515
JJ00000617
0.151
K200000050
0.2175
K200000741
0.2628
JJ00001259
0.1586
K200000154
F300000086
0.1809
F300000460
0.1331
K200000333
0.2285
F300000524
0.8523
0.1256
N200000298
0.1579
0.2429
K200000269
0.1419
K200000553
0.313
K100000101
0.392
K100001330
K100000454
0.167
K100000632
JJ00000715
0.4213
0.1395
K100000194
0.2084
K100000629
0.1333
0.2089
K200000804
0.2828
K100001327
K200001308

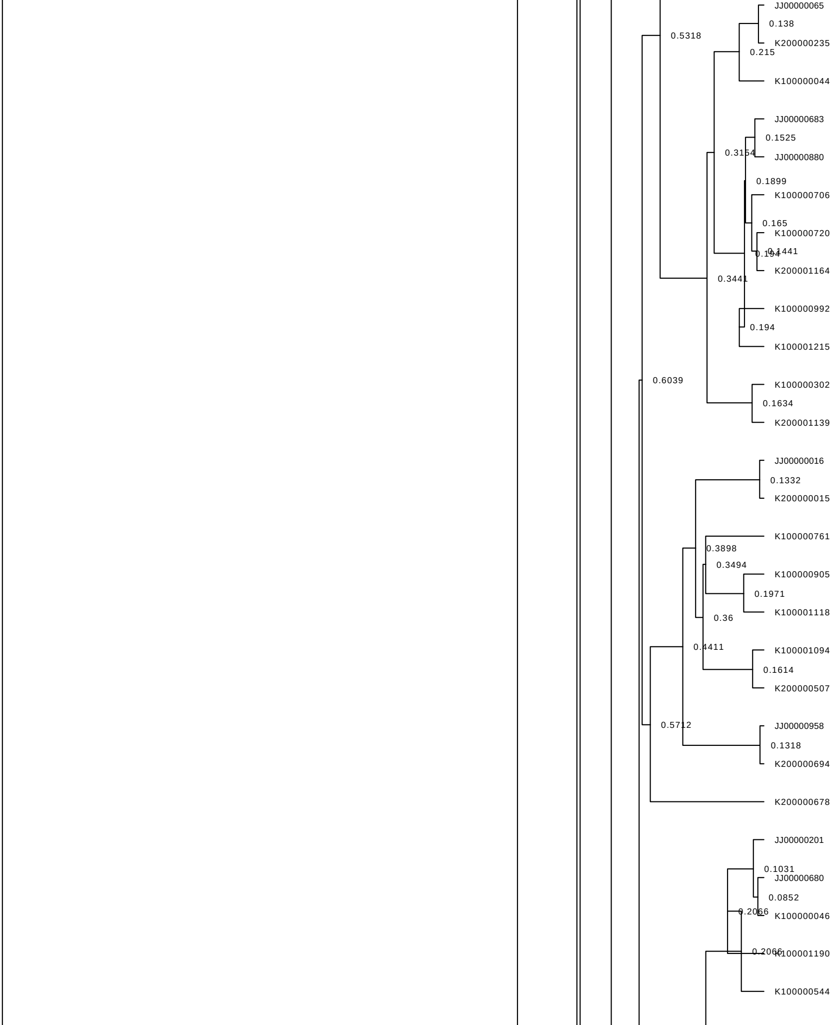

JJ00000065
0.138
K200000235
0.5318
0.215
K100000044
JJ00000683
0.1525
0.3154
JJ00000880
0.1899
K100000706
0.165
K100000720
0.1441
0.194
K200001164
0.3441
K100000992
0.194
K100001215
0.6039
K100000302
0.1634
K200001139
JJ00000016
0.1332
K200000015
K100000761
0.3898
0.3494
K100000905
0.1971
K100001118
0.36
0.4411
K100001094
0.1614
K200000507
0.5712
JJ00000958
0.1318
K200000694
K200000678
JJ00000201
0.1031
JJ00000680
0.0852
0.2066
K100000046
0.2066
K100001190
K100000544

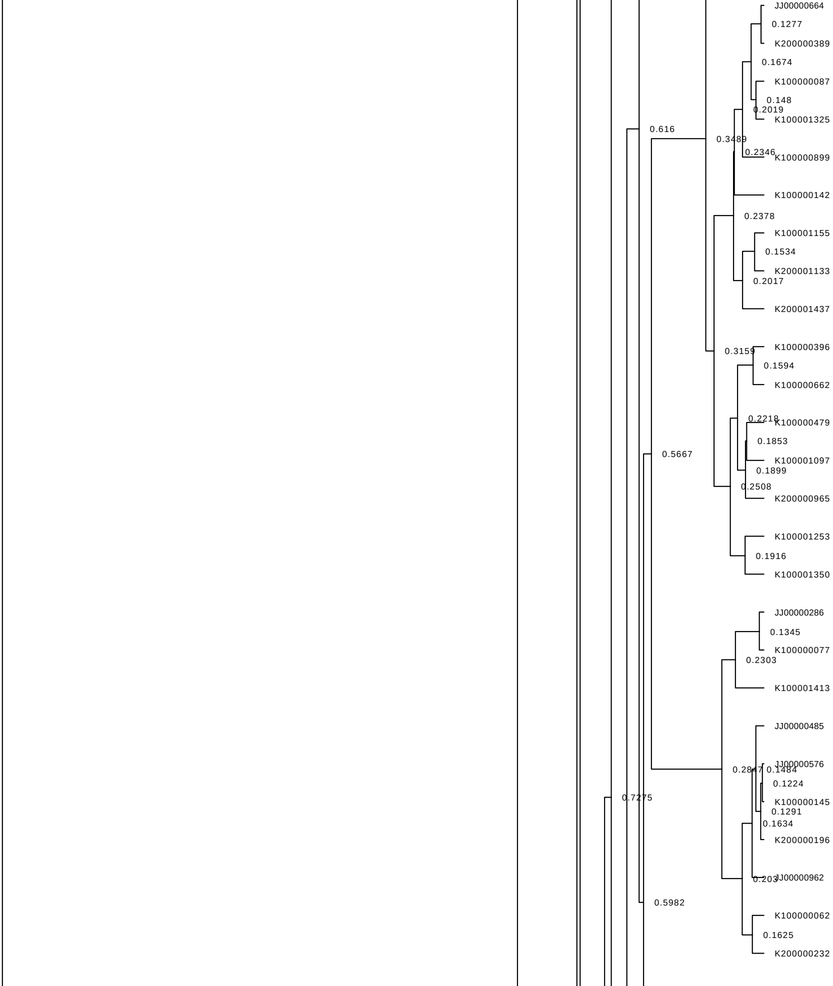

JJ00000664
0.1277
K200000389
0.1674
K100000087
0.148
0.2019
K100001325
0.616
0.3489
0.2346
K100000899
K100000142
0.2378
K100001155
0.1534
K200001133
0.2017
K200001437
K100000396
0.3159
0.1594
K100000662
0.2218
K100000479
0.1853
K100001097
0.5667
0.1899
0.2508
K200000965
K100001253
0.1916
K100001350
JJ00000286
0.1345
K100000077
0.2303
K100001413
JJ00000485
JJ00000576
0.2847
0.1484
0.1224
0.7275
K100000145
0.1291
0.1634
K200000196
0.203
JJ00000962
0.5982
K100000062
0.1625
K200000232

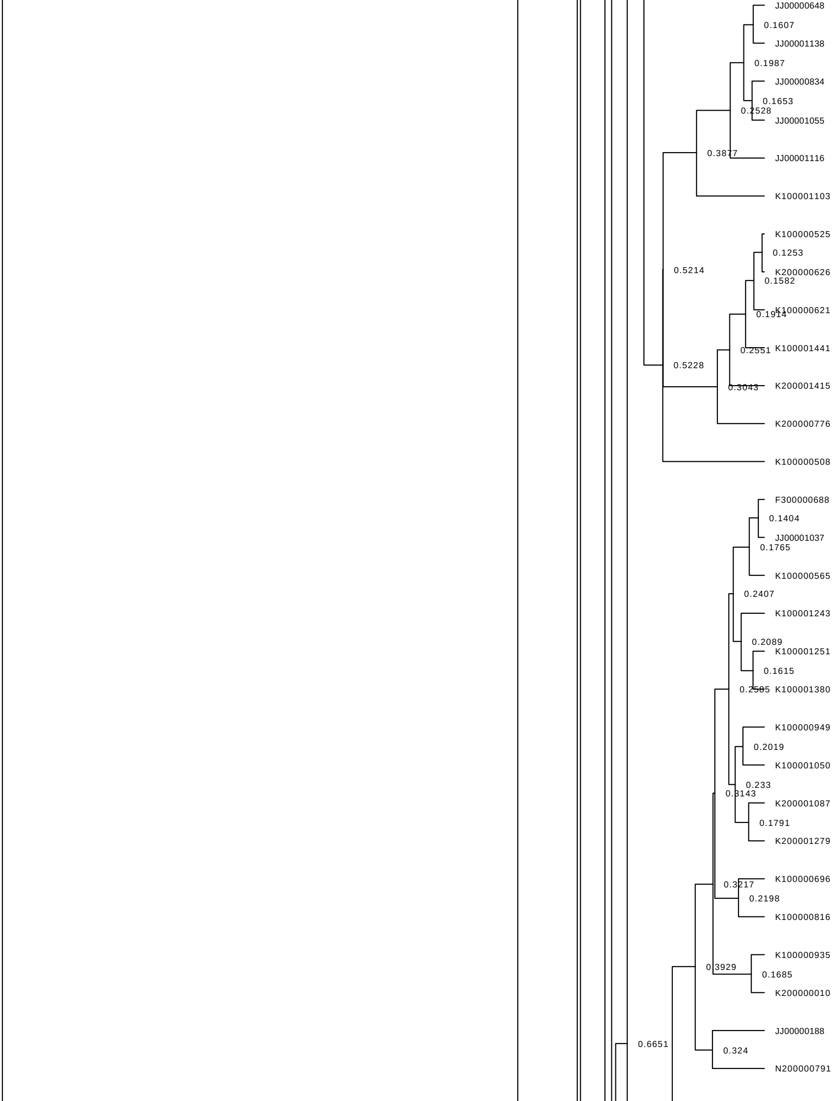
JJ00000648
0.1607
JJ00001138
0.1987
JJ00000834
0.1653
0.2528
JJ00001055
0.3877
JJ00001116
K100001103
K100000525
0.1253
K200000626
0.1582
0.5214
K100000621
0.1914
K100001441
0.2551
0.5228
K200001415
0.3043
K200000776
K100000508
F300000688
0.1404
JJ00001037
0.1765
K100000565
0.2407
K100001243
0.2089
K100001251
0.1615
0.2585
K100001380
K100000949
0.2019
K100001050
0.233
0.3143
K200001087
0.1791
K200001279
K100000696
0.3217
0.2198
K100000816
K100000935
0.3929
0.1685
K200000010
JJ00000188
0.6651
0.324
N200000791

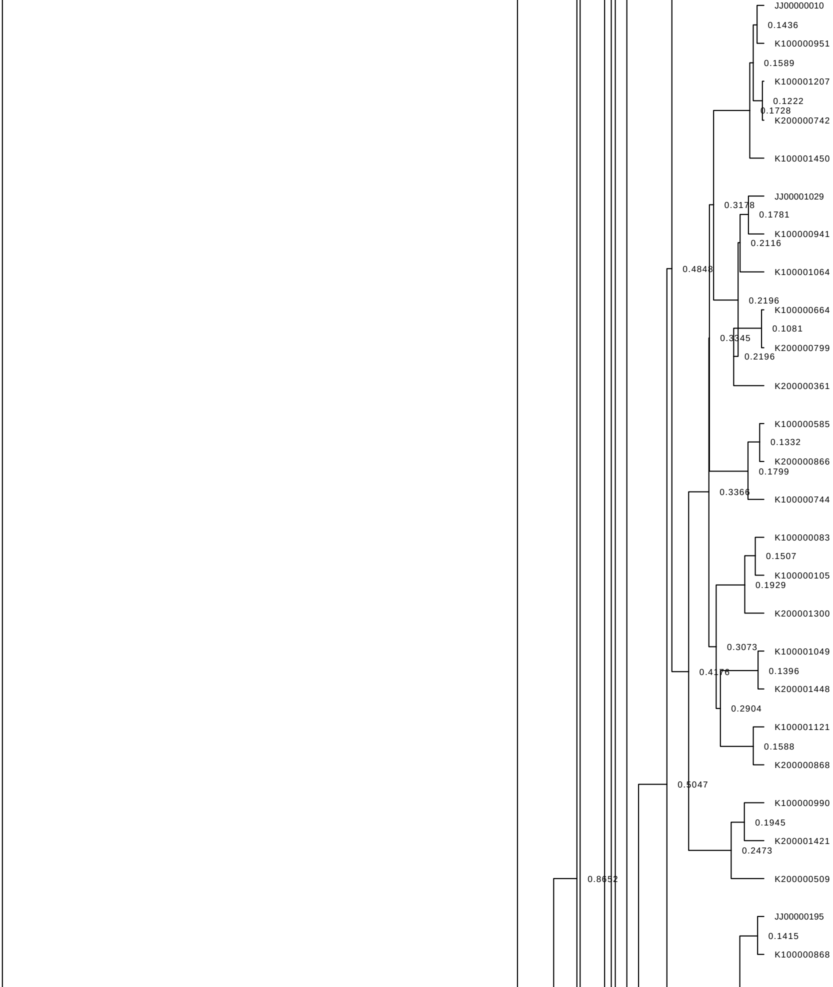
JJ00000010
0.1436
K100000951
0.1589
K100001207
0.1222
0.1728
K200000742
K100001450
JJ00001029
0.3178
0.1781
K100000941
0.2116
0.4848
K100001064
0.2196
K100000664
0.1081
0.3345
K200000799
0.2196
K200000361
K100000585
0.1332
K200000866
0.1799
0.3366
K100000744
K100000083
0.1507
K100000105
0.1929
K200001300
0.3073
K100001049
0.1396
0.4176
K200001448
0.2904
K100001121
0.1588
K200000868
0.5047
K100000990
0.1945
K200001421
0.2473
K200000509
0.8652
JJ00000195
0.1415
K100000868

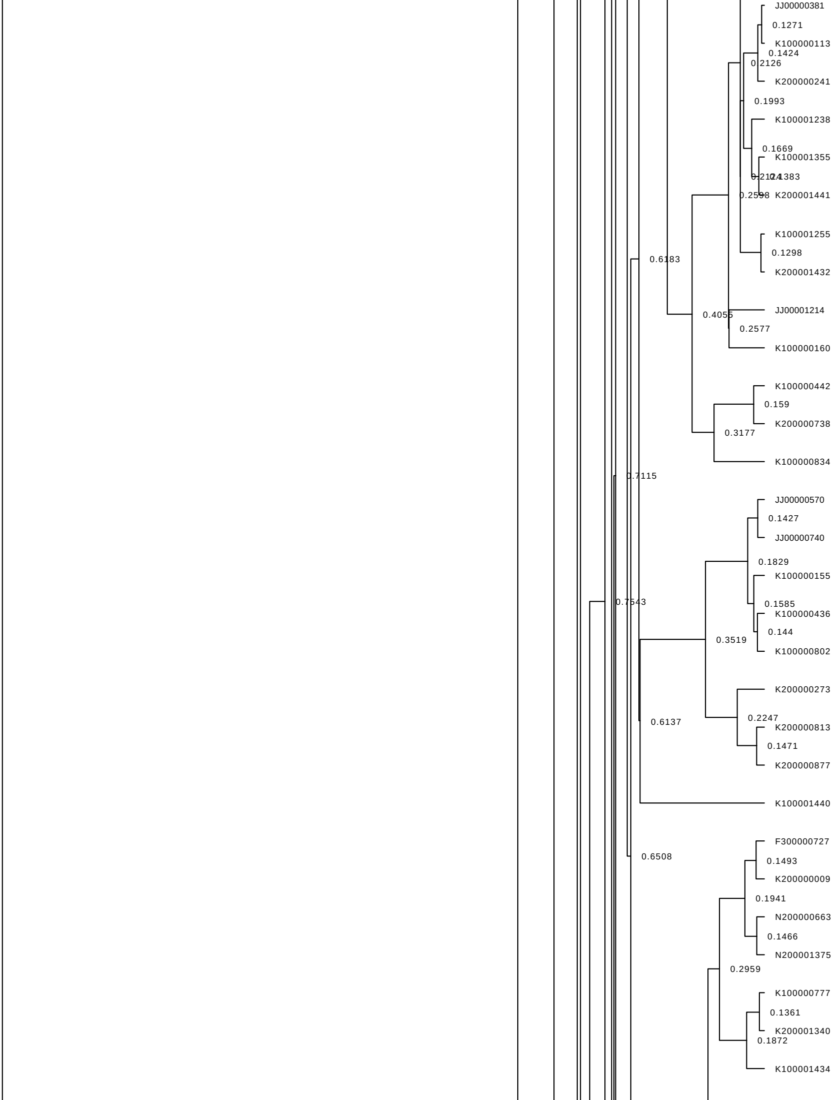
JJ00000381
0.1271
K100000113
0.1424
0.2126
K200000241
0.1993
K100001238
0.1669
K100001355
0.1383
0.2598
K200001441
K100001255
0.1298
K200001432
0.6183
JJ00001214
0.4056
0.2577
K100000160
K100000442
0.159
K200000738
0.3177
K100000834
0.7115
JJ00000570
0.1427
JJ00000740
0.1829
K100000155
0.7543
0.1585
K100000436
0.144
0.3519
K100000802
K200000273
0.2247
0.6137
K200000813
0.1471
K200000877
K100001440
F300000727
0.6508
0.1493
K200000009
0.1941
N200000663
0.1466
N200001375
0.2959
K100000777
0.1361
K200001340
0.1872
K100001434

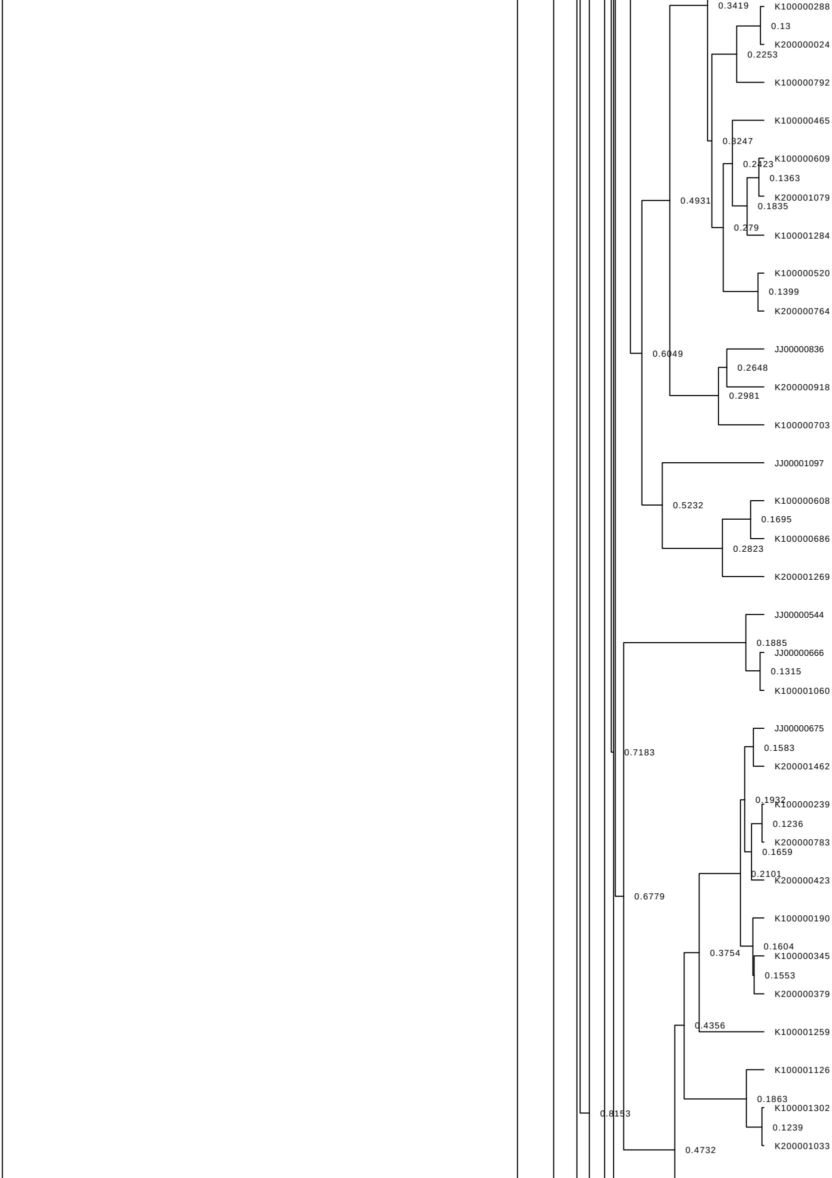
0.3419
K100000288
0.13
K200000024
0.2253
K100000792
K100000465
0.3247
K100000609
0.2423
0.1363
K200001079
0.4931
0.1835
0.279
K100001284
K100000520
0.1399
K200000764
JJ00000836
0.6049
0.2648
K200000918
0.2981
K100000703
JJ00001097
K100000608
0.5232
0.1695
K100000686
0.2823
K200001269
JJ00000544
0.1885
JJ00000666
0.1315
K100001060
JJ00000675
0.1583
0.7183
K200001462
0.1932
K100000239
0.1236
K200000783
0.1659
0.2101
K200000423
0.6779
K100000190
0.1604
0.3754
K100000345
0.1553
K200000379
0.4356
K100001259
K100001126
0.1863
K100001302
0.8153
0.1239
K200001033
0.4732

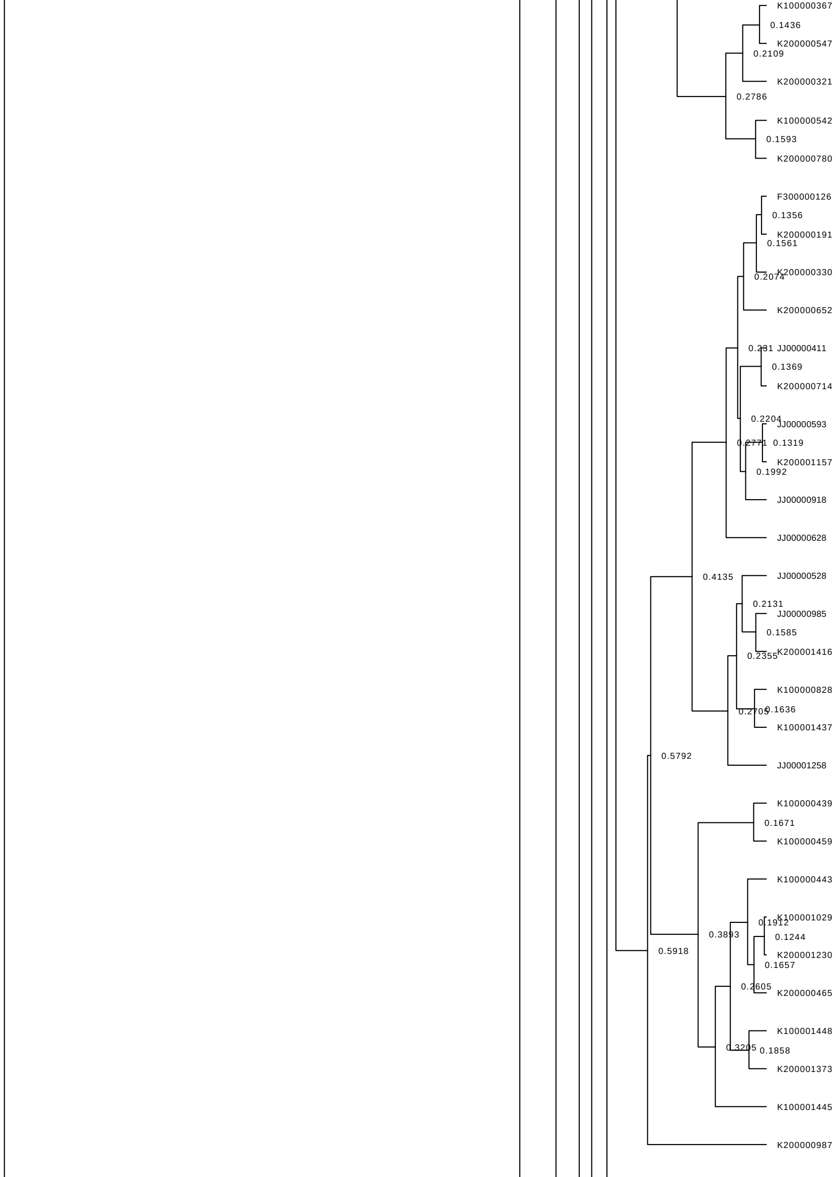

K100000367
0.1436
K200000547
0.2109
K200000321
0.2786
K100000542
0.1593
K200000780
F300000126
0.1356
K200000191
0.1561
K200000330
0.2074
K200000652
0.231
JJ00000411
0.1369
K200000714
0.2204
JJ00000593
0.2771
0.1319
K200001157
0.1992
JJ00000918
JJ00000628
0.4135
JJ00000528
0.2131
JJ00000985
0.1585
K200001416
0.2355
K100000828
0.2705
0.1636
K100001437
0.5792
JJ00001258
K100000439
0.1671
K100000459
K100000443
K100001029
0.1912
0.3893
0.1244
0.5918
K200001230
0.1657
0.2605
K200000465
K100001448
0.3205
0.1858
K200001373
K100001445
K200000987

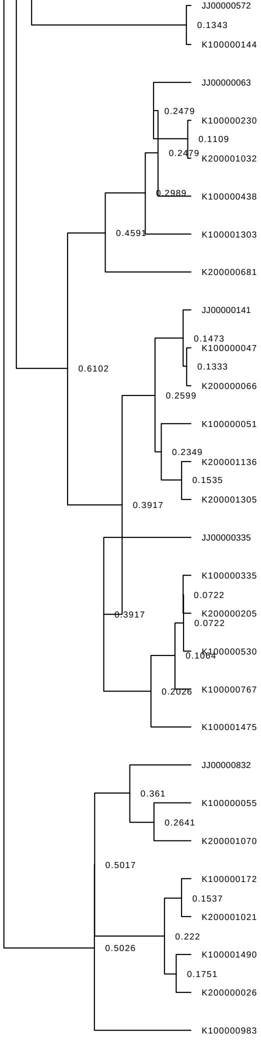

JJ00000572
0.1343
K100000144
JJ00000063
0.2479
K100000230
0.1109
0.2479
K200001032
0.2989
K100000438
0.4591
K100001303
K200000681
JJ00000141
0.1473
K100000047
0.1333
K200000066
0.2599
0.6102
K100000051
0.2349
K200001136
0.1535
K200001305
0.3917
JJ00000335
K100000335
0.0722
0.3917
K200000205
0.0722
K100000530
0.1064
K100000767
0.2026
K100001475
JJ00000832
0.361
K100000055
0.2641
K200001070
0.5017
K100000172
0.1537
K200001021
0.222
0.5026
K100001490
0.1751
K200000026
K100000983

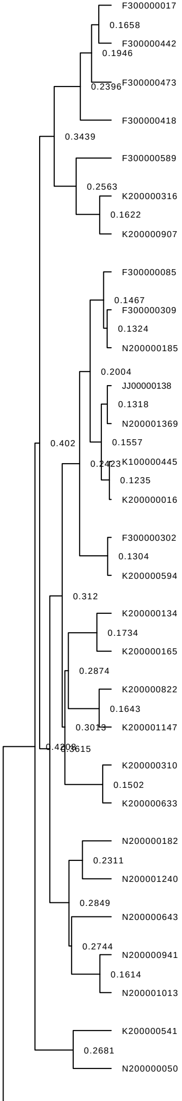

F300000017
0.1658
F300000442
0.1946
F300000473
0.2396
F300000418
0.3439
F300000589
0.2563
K200000316
0.1622
K200000907
F300000085
0.1467
F300000309
0.1324
N200000185
0.2004
JJ00000138
0.1318
N200001369
0.1557
0.402
K100000445
0.2423
0.1235
K200000016
F300000302
0.1304
K200000594
0.312
K200000134
0.1734
K200000165
0.2874
K200000822
0.1643
0.3013
K200001147
0.4208
0.3615
K200000310
0.1502
K200000633
N200000182
0.2311
N200001240
0.2849
N200000643
0.2744
N200000941
0.1614
N200001013
K200000541
0.2681
N200000050

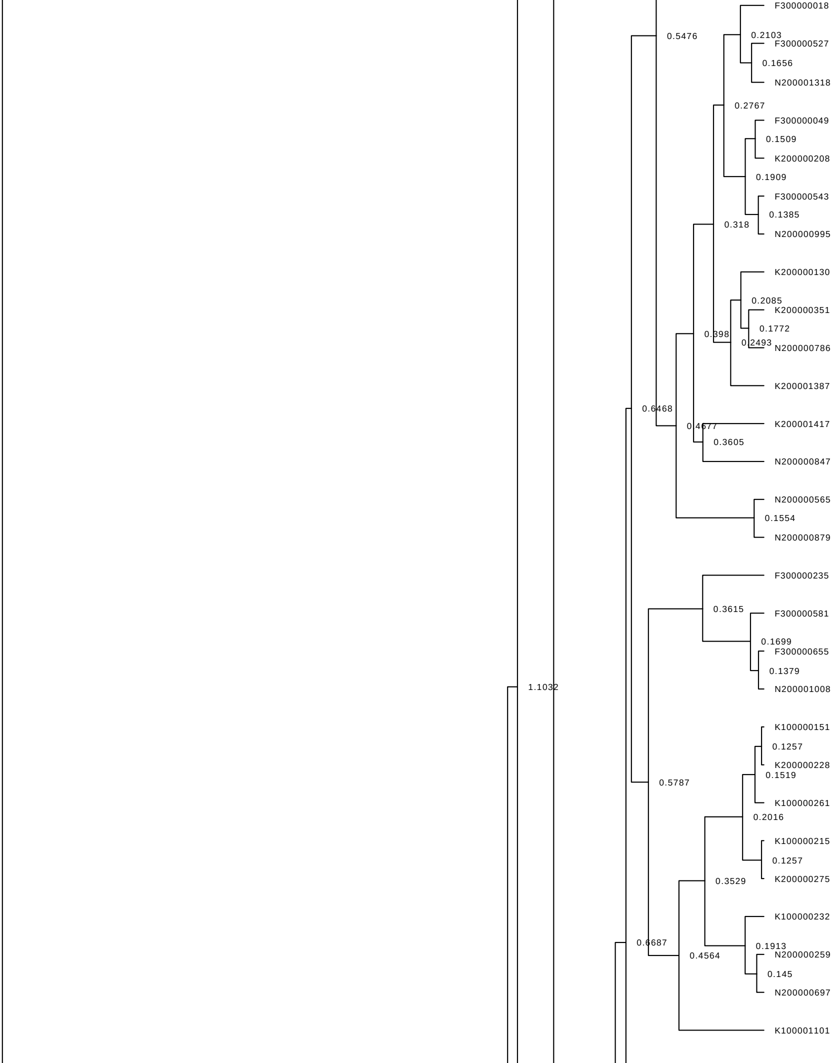
F300000018
0.5476
0.2103
F300000527
0.1656
N200001318
0.2767
F300000049
0.1509
K200000208
0.1909
F300000543
0.1385
0.318
N200000995
K200000130
0.2085
K200000351
0.1772
0.398
0.2493
N200000786
K200001387
0.6468
K200001417
0.4677
0.3605
N200000847
N200000565
0.1554
N200000879
F300000235
0.3615
F300000581
0.1699
F300000655
0.1379
1.1032
N200001008
K100000151
0.1257
K200000228
0.1519
0.5787
K100000261
0.2016
K100000215
0.1257
K200000275
0.3529
K100000232
0.1913
0.6687
N200000259
0.4564
0.145
N200000697
K100001101

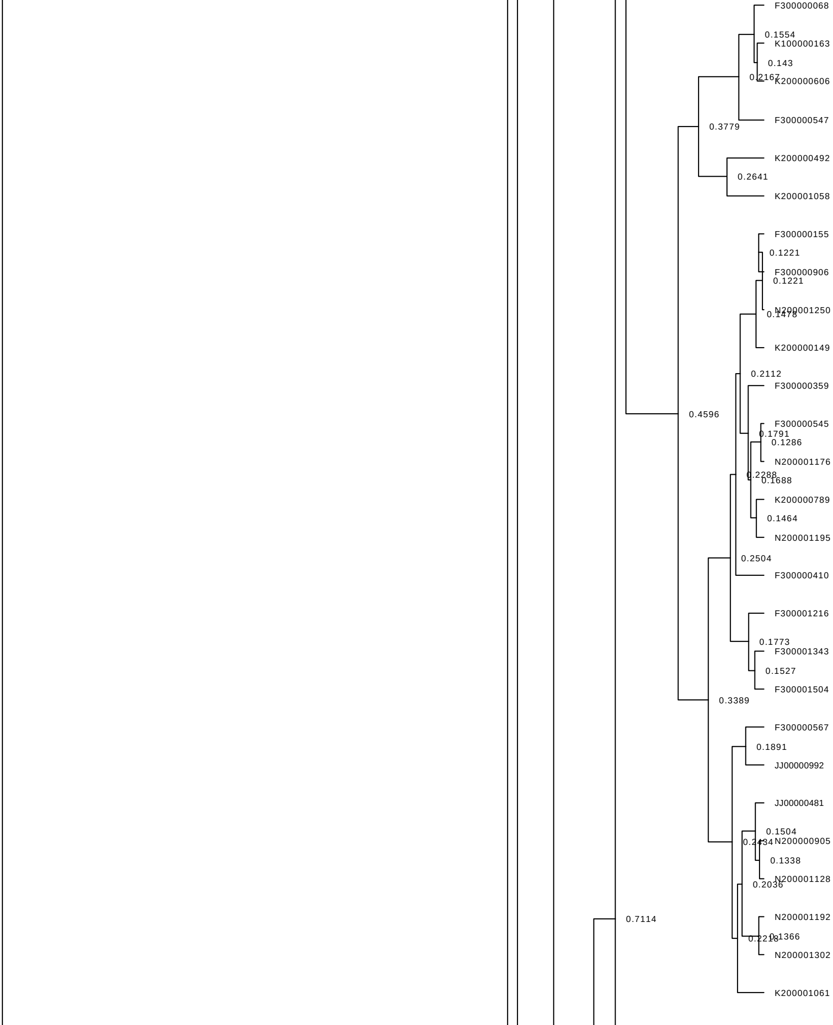
F300000068
0.1554
K100000163
0.143
0.2167
K200000606
F300000547
0.3779
K200000492
0.2641
K200001058
F300000155
0.1221
F300000906
0.1221
N200001250
0.1478
K200000149
0.2112
F300000359
0.4596
F300000545
0.1791
0.1286
N200001176
0.2288
0.1688
K200000789
0.1464
N200001195
0.2504
F300000410
F300001216
0.1773
F300001343
0.1527
F300001504
0.3389
F300000567
0.1891
JJ00000992
JJ00000481
0.1504
0.2434
N200000905
0.1338
N200001128
0.2036
0.7114
N200001192
0.1366
0.2218
N200001302
K200001061

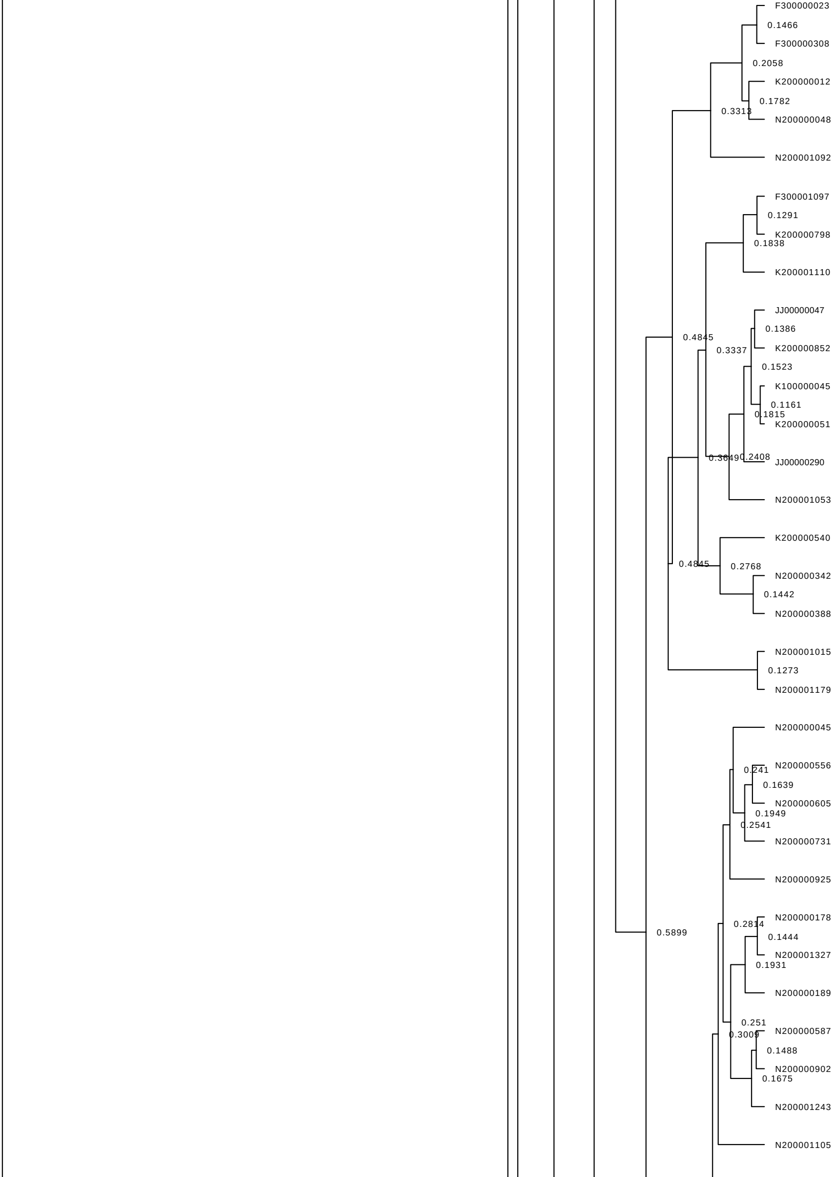
F300000023
0.1466
F300000308
0.2058
K200000012
0.1782
0.3313
N200000048
N200001092
F300001097
0.1291
K200000798
0.1838
K200001110
JJ00000047
0.1386
0.4845
K200000852
0.3337
0.1523
K100000045
0.1161
0.1815
K200000051
0.3649
0.2408
JJ00000290
N200001053
K200000540
0.4845
0.2768
N200000342
0.1442
N200000388
N200001015
0.1273
N200001179
N200000045
N200000556
0.241
0.1639
N200000605
0.1949
0.2541
N200000731
N200000925
N200000178
0.2814
0.5899
0.1444
N200001327
0.1931
N200000189
0.251
N200000587
0.3009
0.1488
N200000902
0.1675
N200001243
N200001105

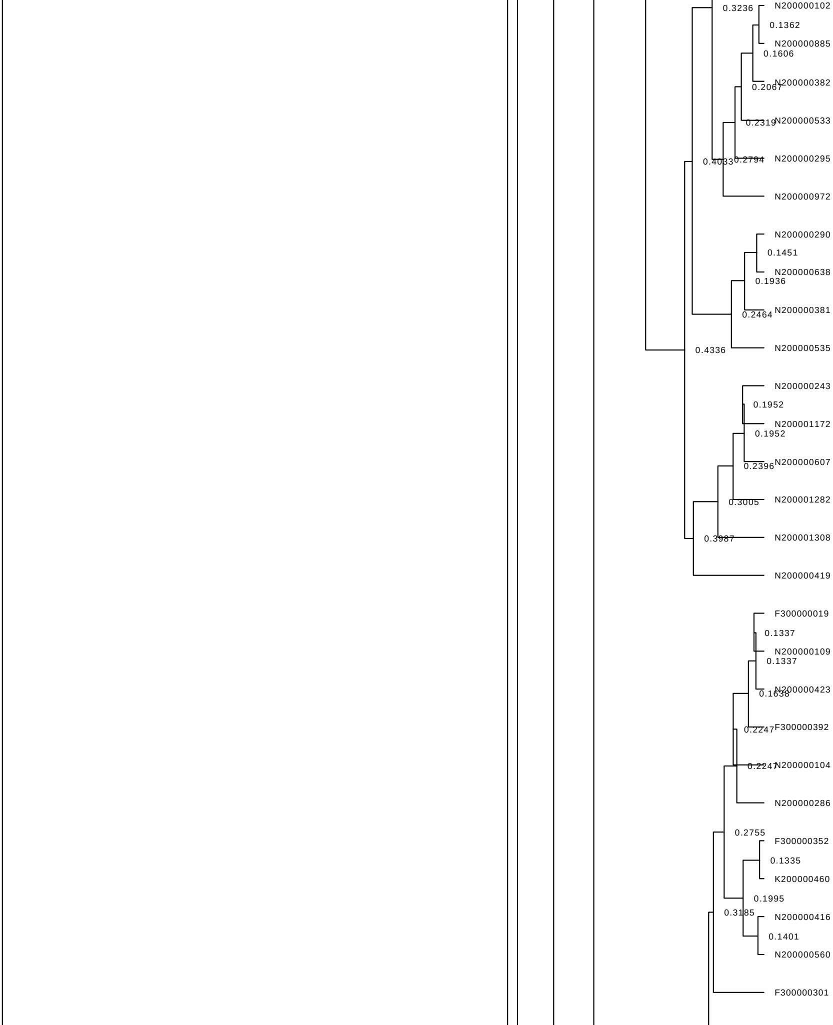

0.3236
N200000102
0.1362
N200000885
0.1606
N200000382
0.2067
N200000533
0.2319
0.4033
0.2794
N200000295
N200000972
N200000290
0.1451
N200000638
0.1936
N200000381
0.2464
0.4336
N200000535
N200000243
0.1952
N200001172
0.1952
N200000607
0.2396
N200001282
0.3005
N200001308
0.3987
N200000419
F300000019
0.1337
N200000109
0.1337
N200000423
0.1638
F300000392
0.2247
N200000104
0.2247
N200000286
0.2755
F300000352
0.1335
K200000460
0.1995
0.3185
N200000416
0.1401
N200000560
F300000301

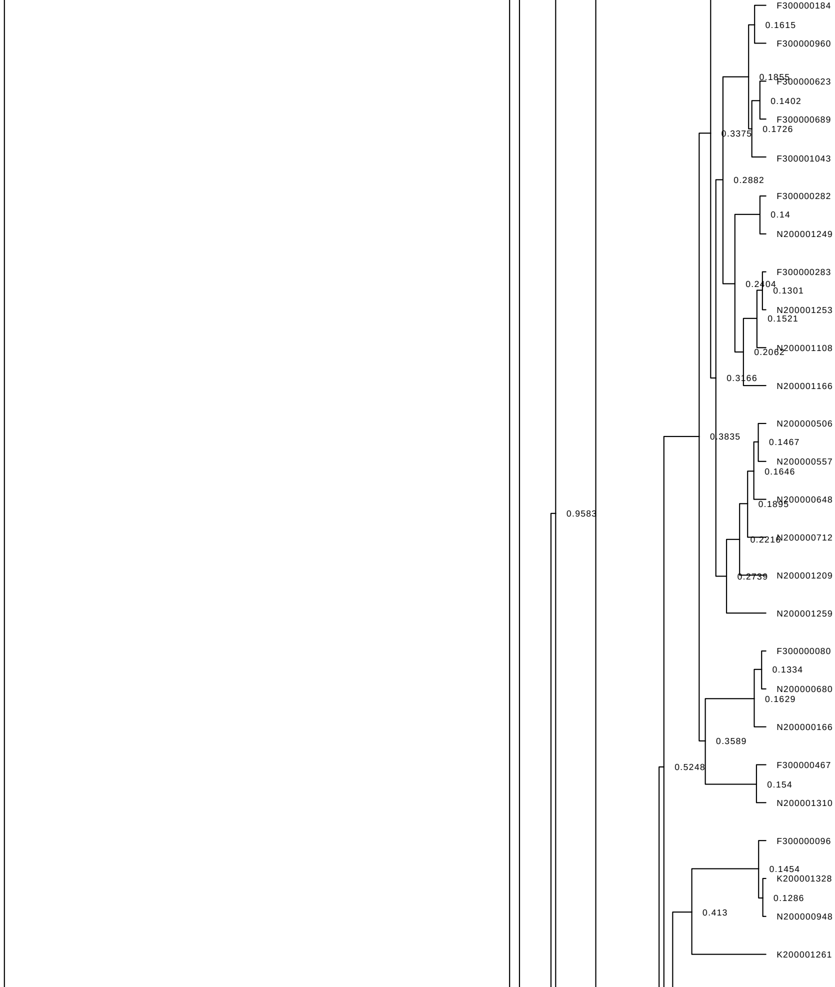
F300000184
0.1615
F300000960
0.1855
F300000623
0.1402
F300000689
0.3375
0.1726
F300001043
0.2882
F300000282
0.14
N200001249
F300000283
0.2404
0.1301
N200001253
0.1521
N200001108
0.2062
0.3166
N200001166
N200000506
0.3835
0.1467
N200000557
0.1646
N200000648
0.1895
0.9583
N200000712
0.2216
N200001209
0.2739
N200001259
F300000080
0.1334
N200000680
0.1629
N200000166
0.3589
F300000467
0.5248
0.154
N200001310
F300000096
0.1454
K200001328
0.1286
0.413
N200000948
K200001261

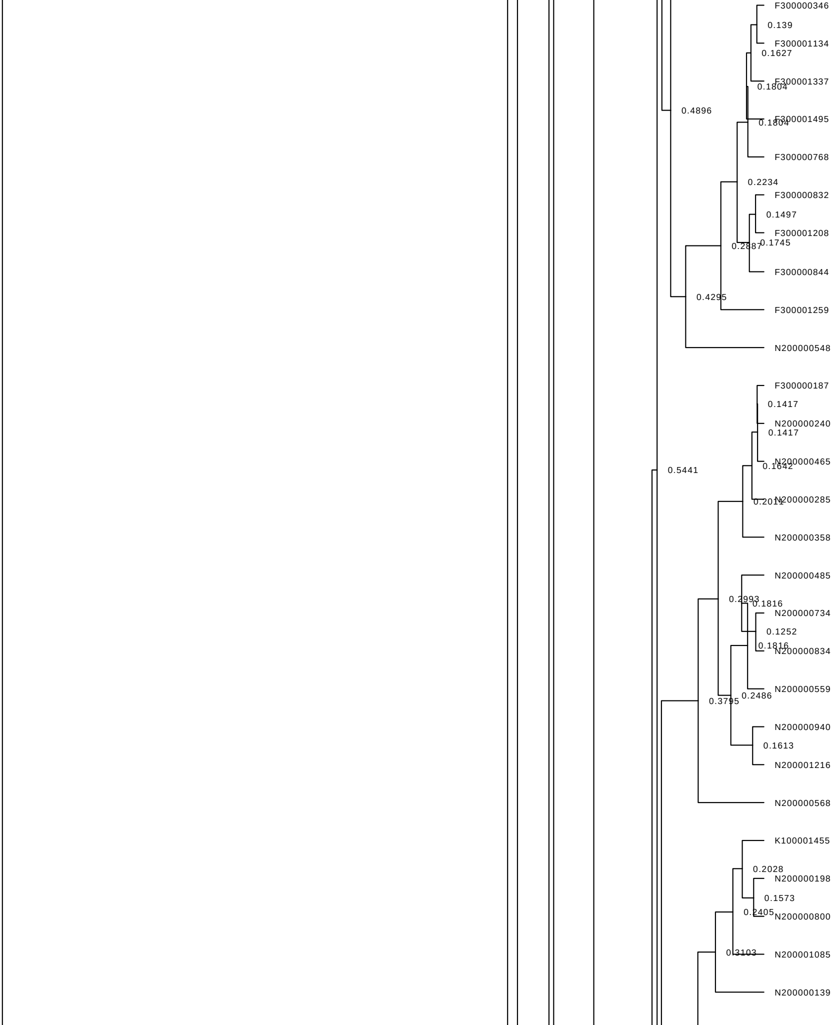
F300000346
0.139
F300001134
0.1627
F300001337
0.1804
0.4896
F300001495
0.1804
F300000768
0.2234
F300000832
0.1497
F300001208
0.2887
0.1745
F300000844
0.4295
F300001259
N200000548
F300000187
0.1417
N200000240
0.1417
0.5441
N200000465
0.1642
N200000285
0.2011
N200000358
N200000485
0.2993
0.1816
N200000734
0.1252
0.1816
N200000834
N200000559
0.3795
0.2486
N200000940
0.1613
N200001216
N200000568
K100001455
0.2028
N200000198
0.1573
0.2405
N200000800
0.3103
N200001085
N200000139

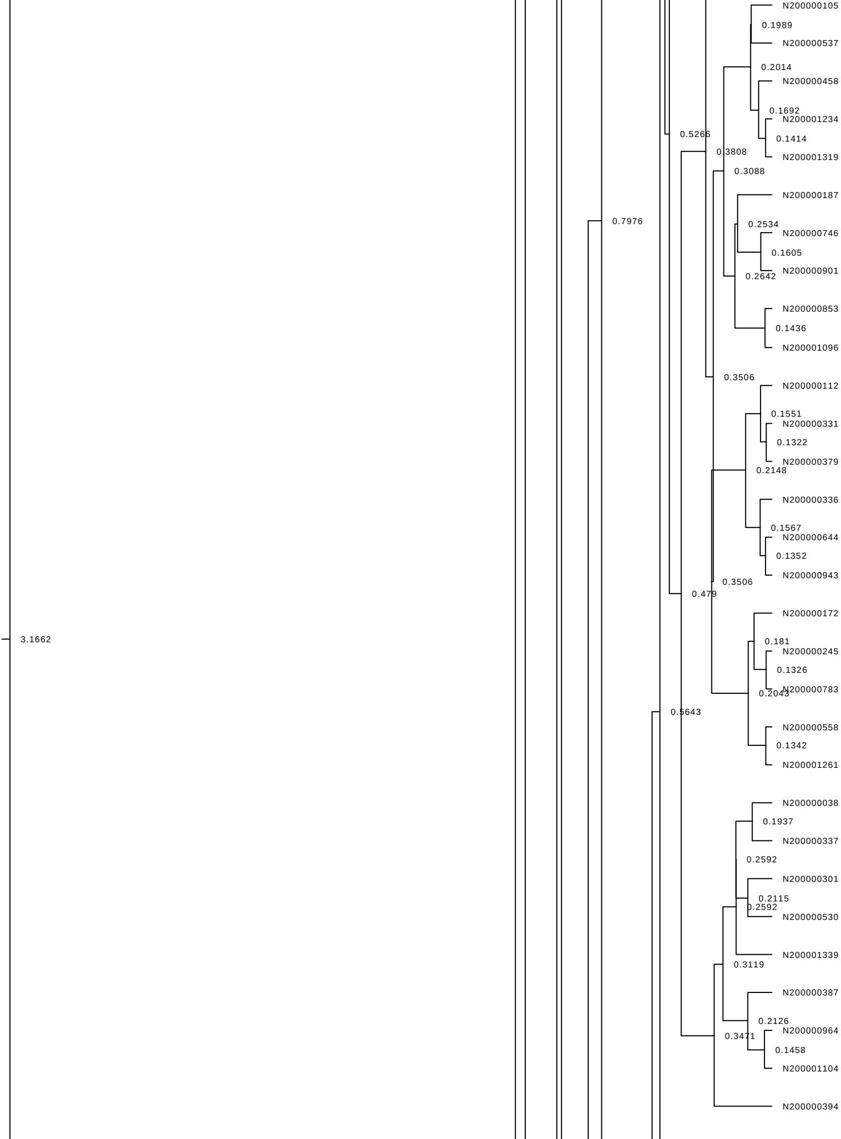
N200000105
0.1989
N200000537
0.2014
N200000458
0.1692
N200001234
0.1414
0.5266
N200001319
0.3808
0.3088
N200000187
0.7976
0.2534
N200000746
0.1605
N200000901
0.2642
N200000853
0.1436
N200001096
0.3506
N200000112
0.1551
N200000331
0.1322
N200000379
0.2148
N200000336
0.1567
N200000644
0.1352
N200000943
0.3506
0.479
N200000172
3.1662
0.181
N200000245
0.1326
N200000783
0.2043
0.5643
N200000558
0.1342
N200001261
N200000038
0.1937
N200000337
0.2592
N200000301
0.2115
0.2592
N200000530
N200001339
0.3119
N200000387
0.2126
N200000964
0.3471
0.1458
N200001104
N200000394

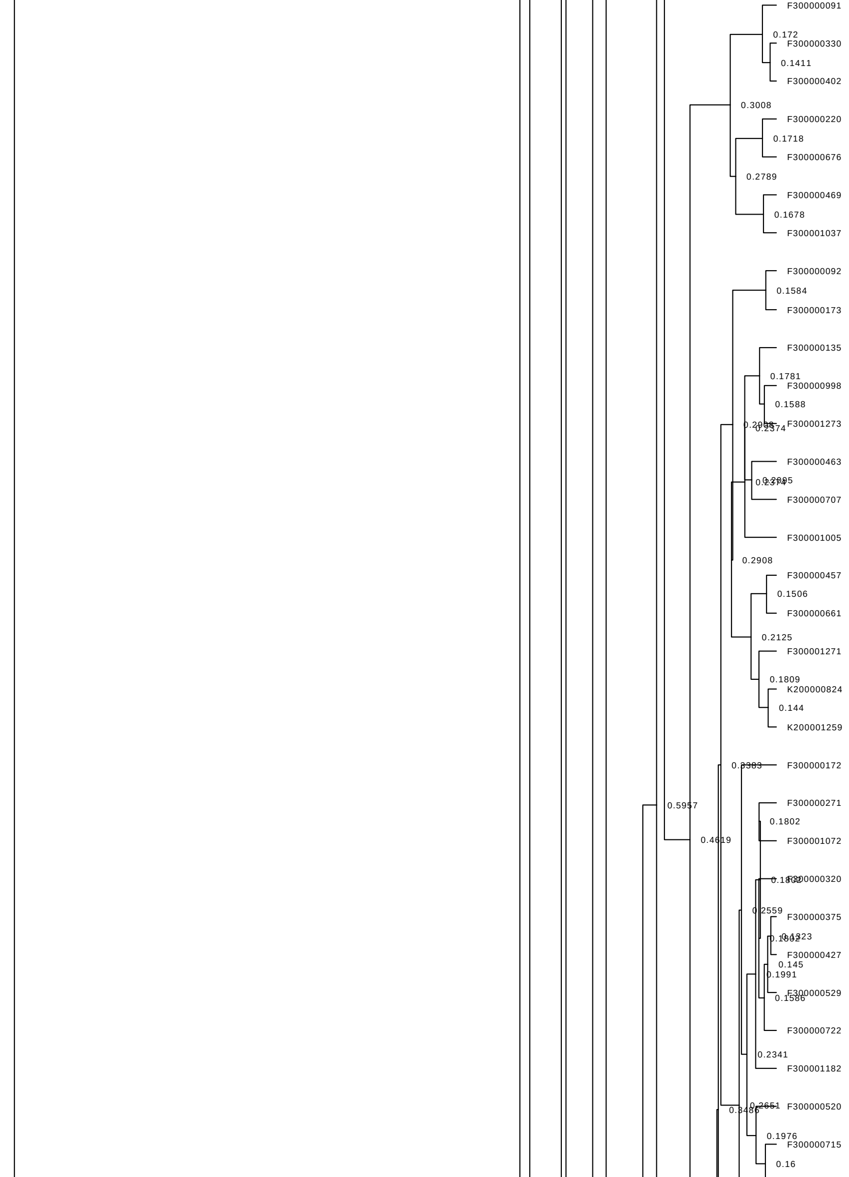

F300000091
0.172
F300000330
0.1411
F300000402
0.3008
F300000220
0.1718
F300000676
0.2789
F300000469
0.1678
F300001037
F300000092
0.1584
F300000173
F300000135
0.1781
F300000998
0.1588
0.2374
F300001273
F300000463
0.2374
F300000707
F300001005
0.2908
F300000457
0.1506
F300000661
0.2125
F300001271
0.1809
K200000824
0.144
K200001259
F300000172
F300000271
0.5957
0.1802
0.4619
F300001072
F300000320
0.2559
F300000375
0.1323
0.1802
F300000427
0.145
0.1991
F300000529
0.1586
F300000722
0.2341
F300001182
0.2651
F300000520
0.3486
0.1976
F300000715
0.16

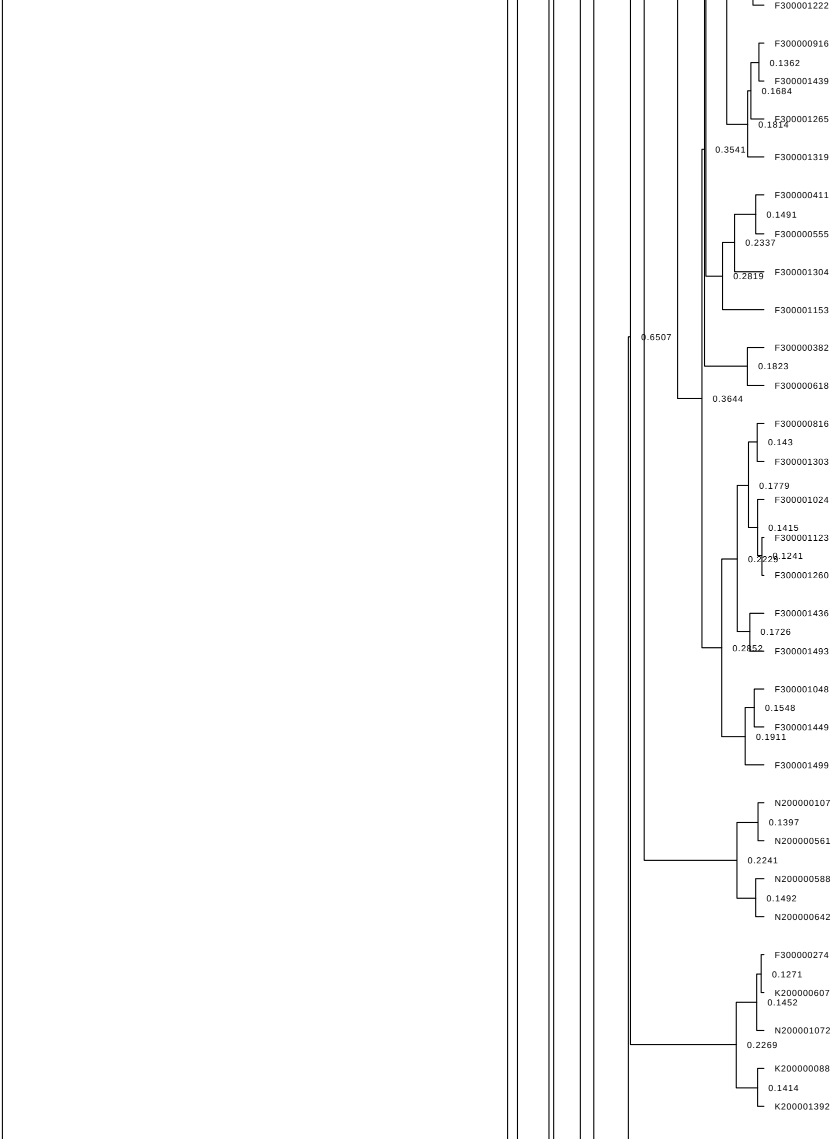

F300001222
F300000916
0.1362
F300001439
0.1684
F300001265
0.1814
0.3541
F300001319
F300000411
0.1491
F300000555
0.2337
F300001304
0.2819
F300001153
0.6507
F300000382
0.1823
F300000618
0.3644
F300000816
0.143
F300001303
0.1779
F300001024
0.1415
F300001123
0.1241
0.2229
F300001260
F300001436
0.1726
0.2852
F300001493
F300001048
0.1548
F300001449
0.1911
F300001499
N200000107
0.1397
N200000561
0.2241
N200000588
0.1492
N200000642
F300000274
0.1271
K200000607
0.1452
N200001072
0.2269
K200000088
0.1414
K200001392

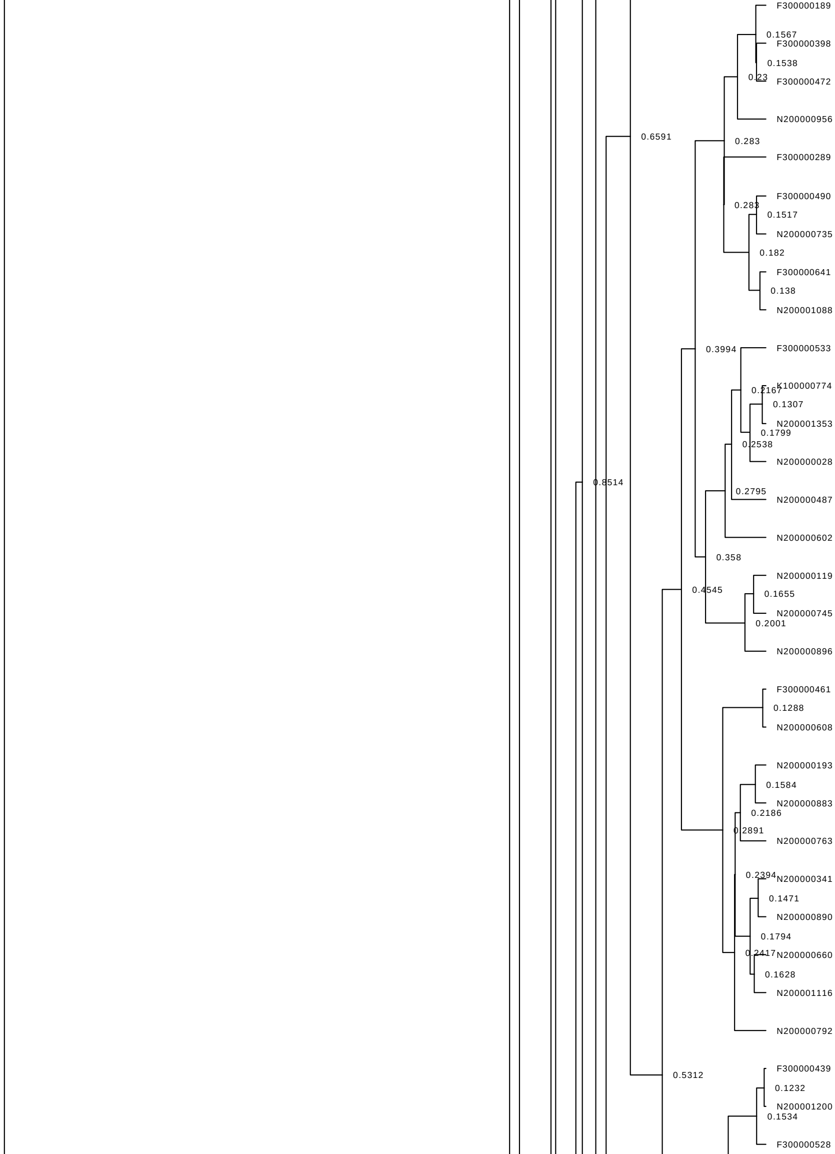

F300000189
0.1567
F300000398
0.1538
0.23
F300000472
N200000956
0.6591
0.283
F300000289
F300000490
0.283
0.1517
N200000735
0.182
F300000641
0.138
N200001088
0.3994
F300000533
K100000774
0.2167
0.1307
N200001353
0.1799
0.2538
N200000028
0.8514
0.2795
N200000487
N200000602
0.358
N200000119
0.4545
0.1655
N200000745
0.2001
N200000896
F300000461
0.1288
N200000608
N200000193
0.1584
N200000883
0.2186
0.2891
N200000763
0.2394
N200000341
0.1471
N200000890
0.1794
0.2417
N200000660
0.1628
N200001116
N200000792
F300000439
0.5312
0.1232
N200001200
0.1534
F300000528

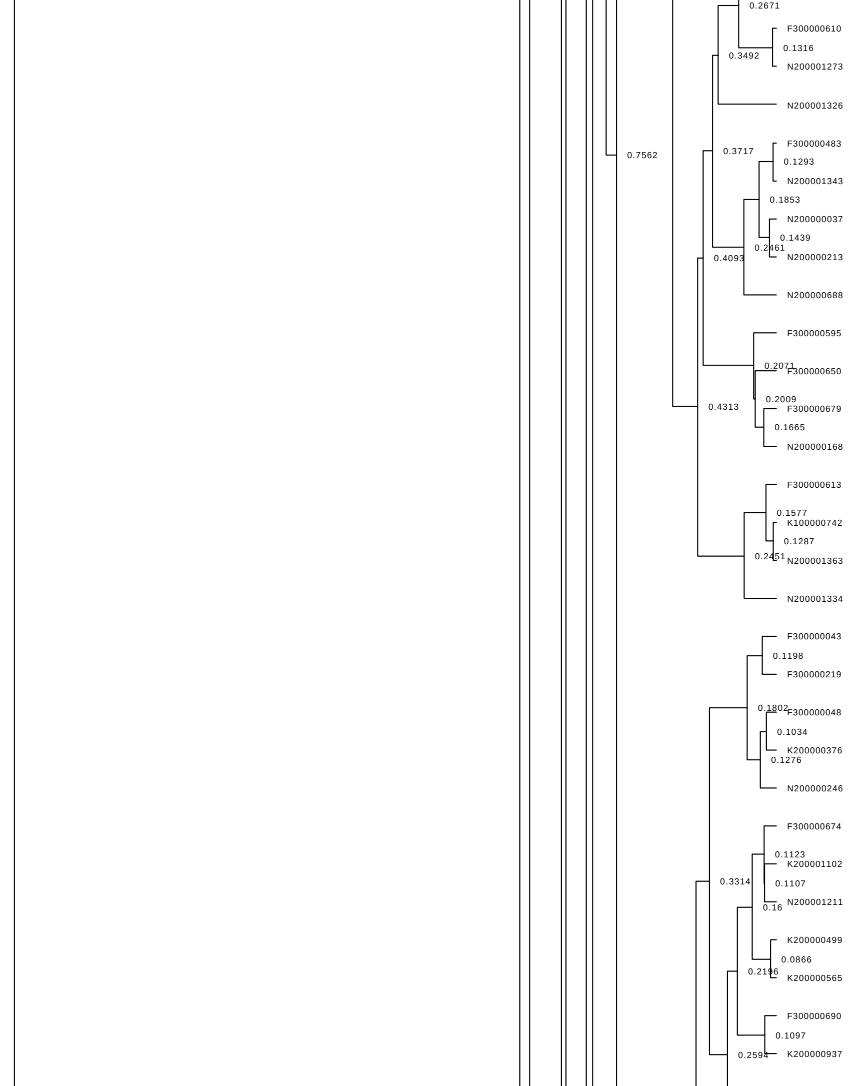

0.2671
F300000610
0.1316
0.3492
N200001273
N200001326
0.7562
F300000483
0.3717
0.1293
N200001343
0.1853
N200000037
0.1439
0.2461
0.4093
N200000213
N200000688
F300000595
0.2071
F300000650
0.2009
0.4313
F300000679
0.1665
N200000168
F300000613
0.1577
K100000742
0.1287
0.2451
N200001363
N200001334
F300000043
0.1198
F300000219
0.1802
F300000048
0.1034
K200000376
0.1276
N200000246
F300000674
0.1123
K200001102
0.3314
0.1107
N200001211
0.16
K200000499
0.0866
0.2196
K200000565
F300000690
0.1097
0.2594
K200000937

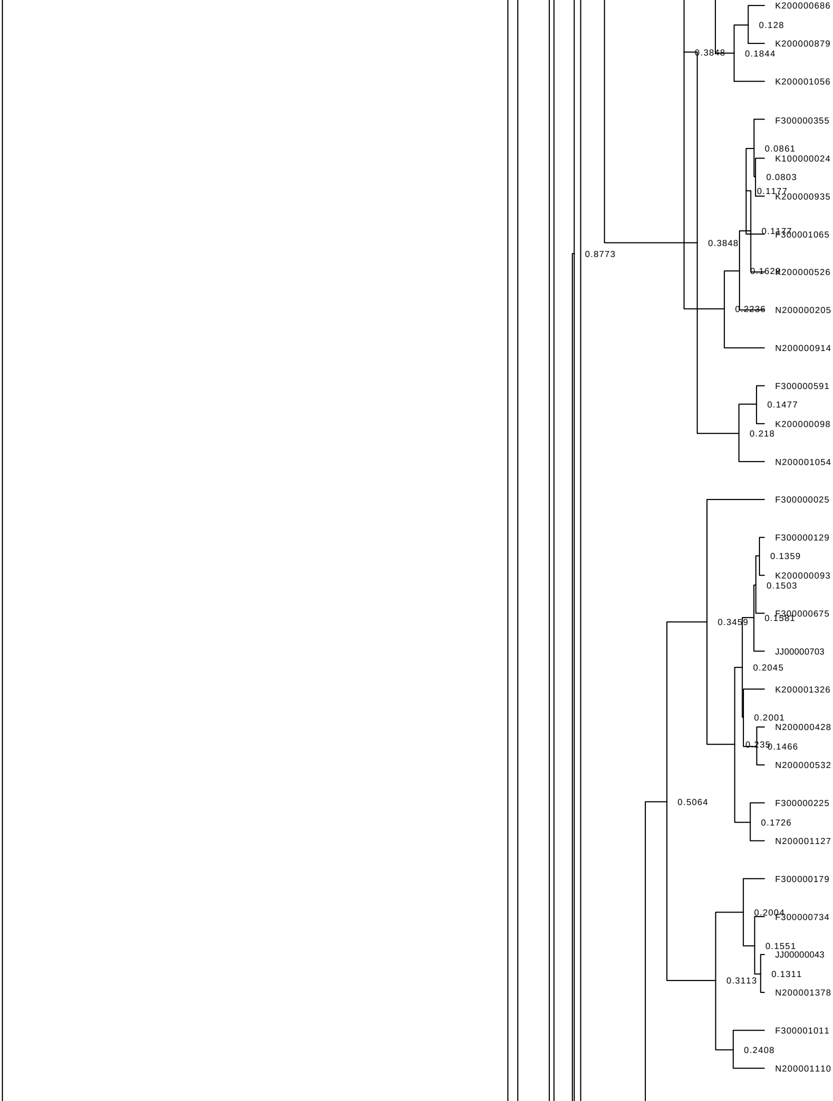

K200000686
0.128
K200000879
0.3848
0.1844
K200001056
F300000355
0.0861
K100000024
0.0803
0.1177
K200000935
0.1177
F300001065
0.3848
0.8773
0.1629
K200000526
0.2236
N200000205
N200000914
F300000591
0.1477
K200000098
0.218
N200001054
F300000025
F300000129
0.1359
K200000093
0.1503
F300000675
0.1581
0.3459
JJ00000703
0.2045
K200001326
0.2001
N200000428
0.235
0.1466
N200000532
0.5064
F300000225
0.1726
N200001127
F300000179
0.2004
F300000734
0.1551
JJ00000043
0.1311
0.3113
N200001378
F300001011
0.2408
N200001110

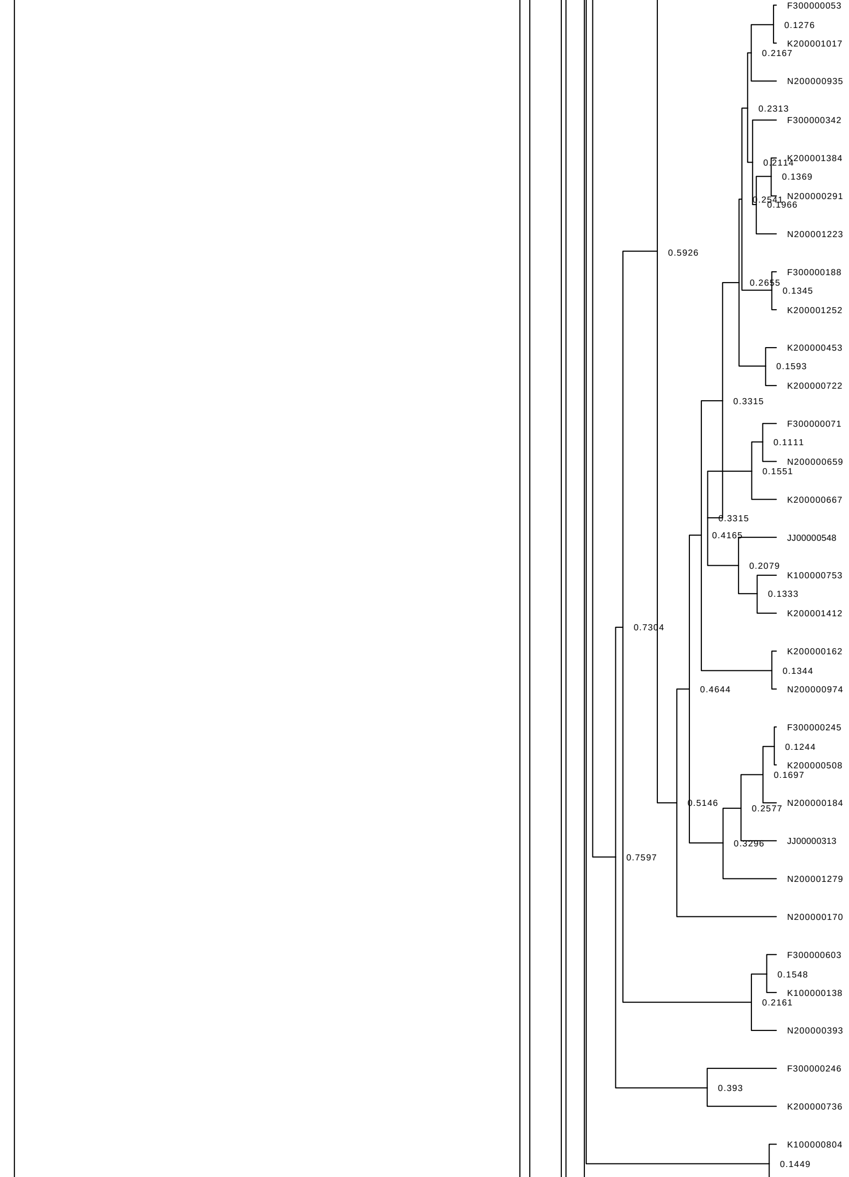

F300000053
0.1276
K200001017
0.2167
N200000935
0.2313
F300000342
K200001384
0.2114
0.1369
N200000291
0.2541
0.1966
N200001223
0.5926
F300000188
0.2655
0.1345
K200001252
K200000453
0.1593
K200000722
0.3315
F300000071
0.1111
N200000659
0.1551
K200000667
0.3315
0.4165
JJ00000548
0.2079
K100000753
0.1333
K200001412
0.7304
K200000162
0.1344
N200000974
0.4644
F300000245
0.1244
K200000508
0.1697
N200000184
0.5146
0.2577
JJ00000313
0.3296
0.7597
N200001279
N200000170
F300000603
0.1548
K100000138
0.2161
N200000393
F300000246
0.393
K200000736
K100000804
0.1449

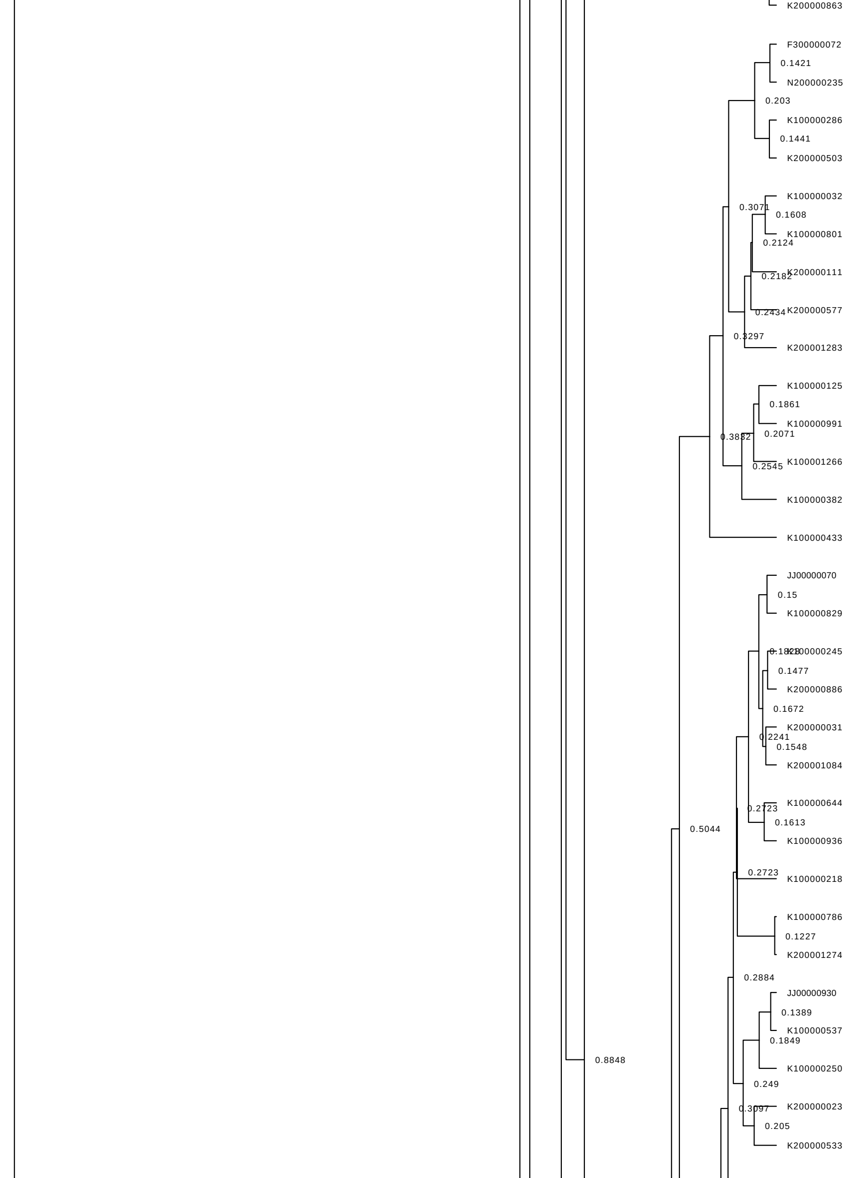
K200000863
F300000072
0.1421
N200000235
0.203
K100000286
0.1441
K200000503
K100000032
0.3071
0.1608
K100000801
0.2124
K200000111
0.2182
K200000577
0.2434
0.3297
K200001283
K100000125
0.1861
K100000991
0.2071
0.3832
K100001266
0.2545
K100000382
K100000433
JJ00000070
0.15
K100000829
K200000245
0.1477
K200000886
0.1672
K200000031
0.2241
0.1548
K200001084
K100000644
0.2723
0.1613
K100000936
0.5044
0.2723
K100000218
K100000786
0.1227
K200001274
0.2884
JJ00000930
0.1389
K100000537
0.1849
0.8848
K100000250
0.249
K200000023
0.3097
0.205
K200000533

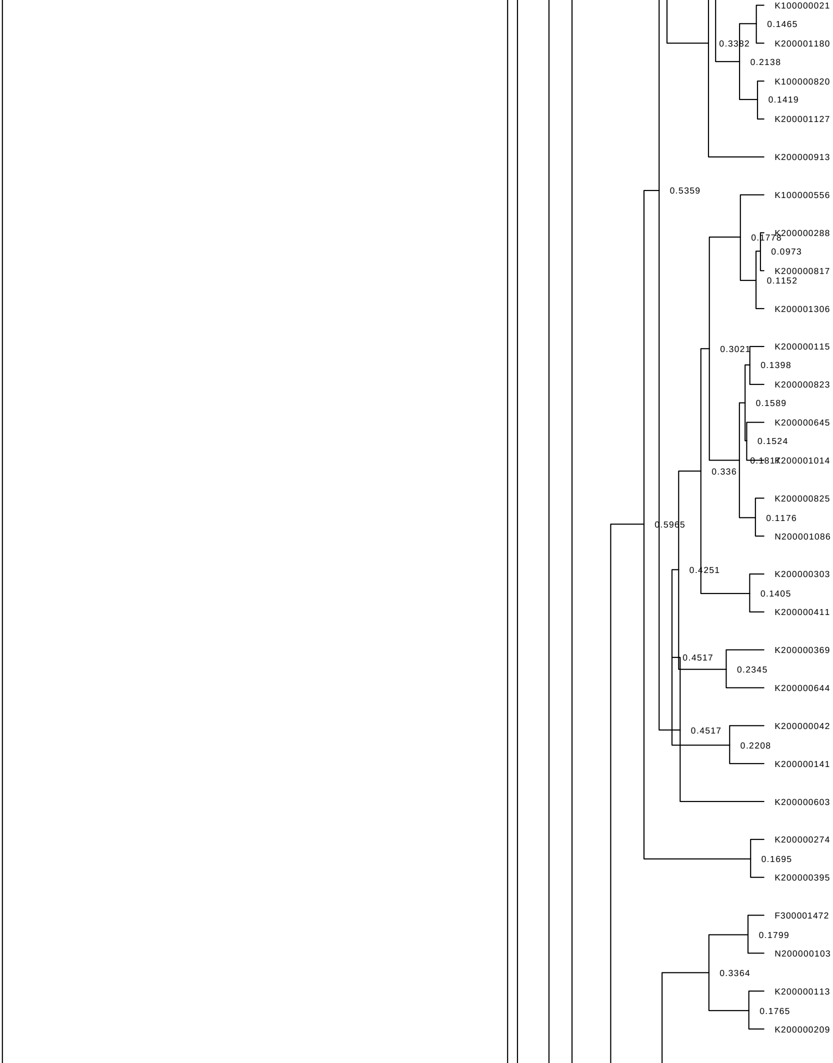
K100000021
0.1465
K200001180
0.3382
0.2138
K100000820
0.1419
K200001127
K200000913
0.5359
K100000556
K200000288
0.1778
0.0973
K200000817
0.1152
K200001306
K200000115
0.3021
0.1398
K200000823
0.1589
K200000645
0.1524
K200001014
0.1817
0.336
K200000825
0.1176
N200001086
0.5965
0.4251
K200000303
0.1405
K200000411
K200000369
0.4517
0.2345
K200000644
K200000042
0.4517
0.2208
K200000141
K200000603
K200000274
0.1695
K200000395
F300001472
0.1799
N200000103
0.3364
K200000113
0.1765
K200000209

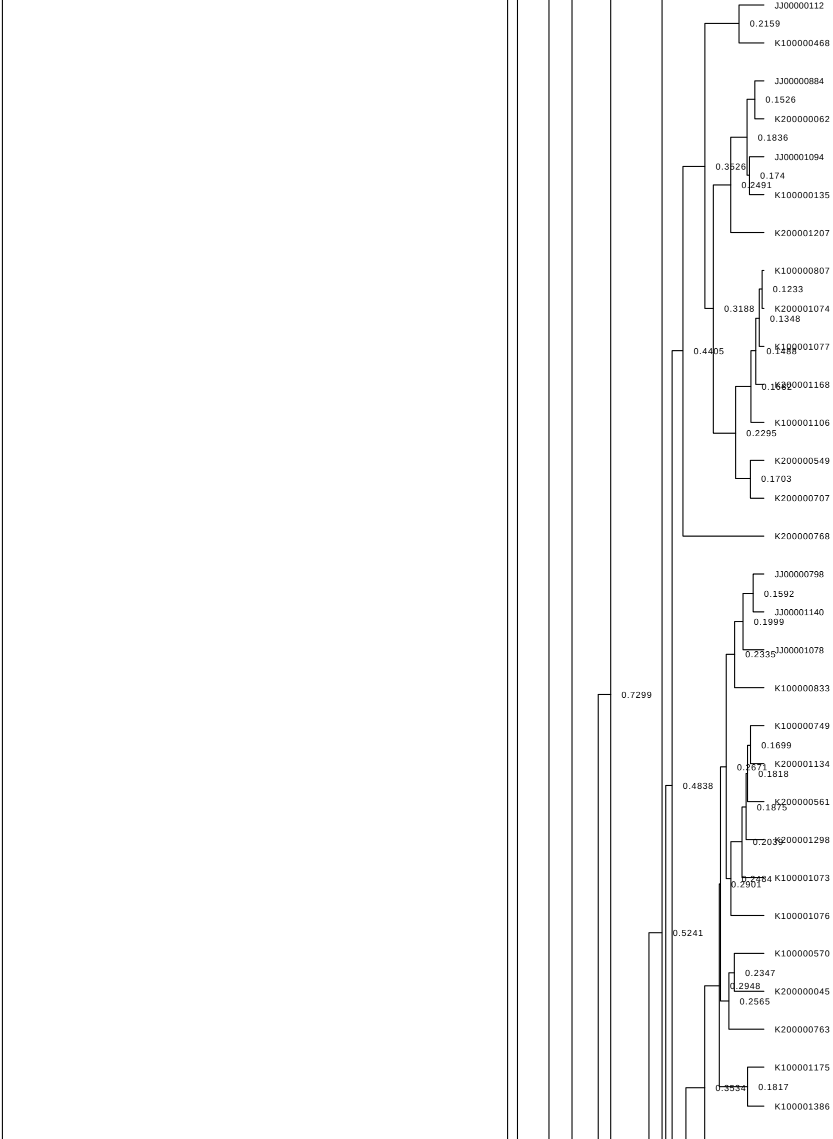
JJ00000112
0.2159
K100000468
JJ00000884
0.1526
K200000062
0.1836
JJ00001094
0.3526
0.174
0.2491
K100000135
K200001207
K100000807
0.1233
0.3188
K200001074
0.1348
K100001077
0.4405
0.1488
K200001168
0.1682
K100001106
0.2295
K200000549
0.1703
K200000707
K200000768
JJ00000798
0.1592
JJ00001140
0.1999
JJ00001078
0.2335
K100000833
0.7299
K100000749
0.1699
K200001134
0.2671
0.1818
0.4838
K200000561
0.1875
K200001298
0.2039
K100001073
0.2484
0.2901
K100001076
0.5241
K100000570
0.2347
0.2948
K200000045
0.2565
K200000763
K100001175
0.3534
0.1817
K100001386

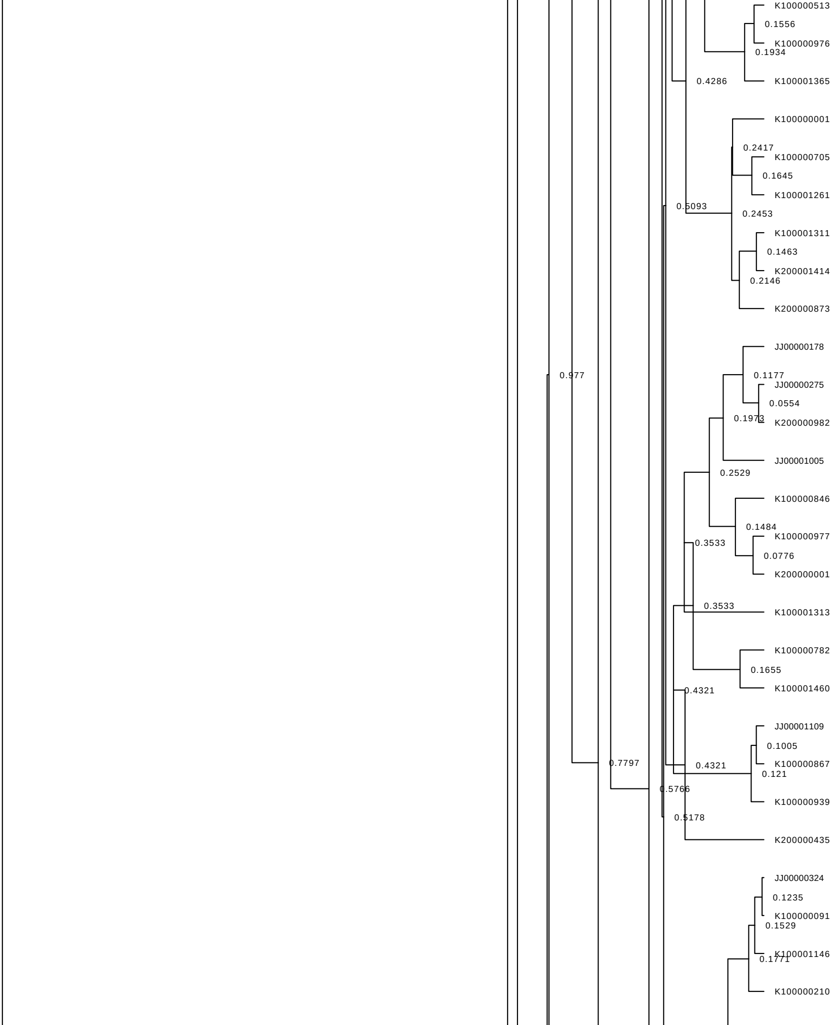
K100000513
0.1556
K100000976
0.1934
0.4286
K100001365
K100000001
0.2417
K100000705
0.1645
K100001261
0.5093
0.2453
K100001311
0.1463
K200001414
0.2146
K200000873
JJ00000178
0.977
0.1177
JJ00000275
0.0554
0.1973
K200000982
JJ00001005
0.2529
K100000846
0.1484
K100000977
0.3533
0.0776
K200000001
0.3533
K100001313
K100000782
0.1655
K100001460
0.4321
JJ00001109
0.1005
0.7797
0.4321
K100000867
0.121
0.5766
K100000939
0.5178
K200000435
JJ00000324
0.1235
K100000091
0.1529
K100001146
0.1771
K100000210

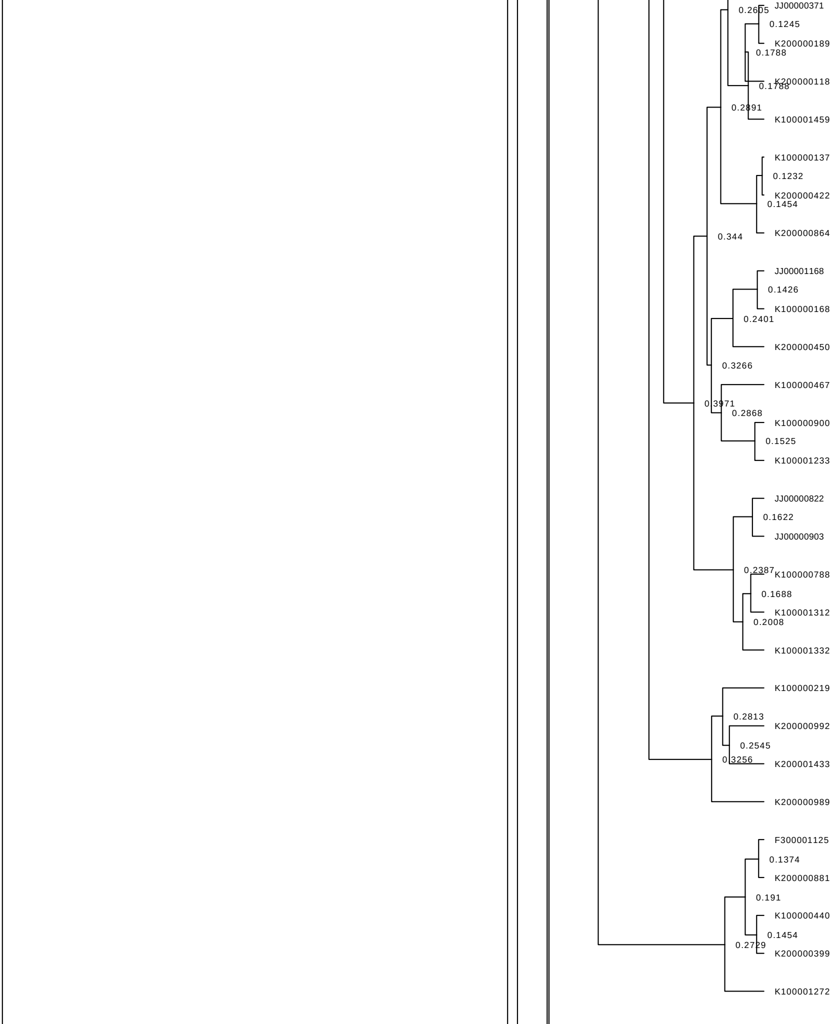
JJ00000371
0.2605
0.1245
K200000189
0.1788
K200000118
0.1788
0.2891
K100001459
K100000137
0.1232
K200000422
0.1454
K200000864
0.344
JJ00001168
0.1426
K100000168
0.2401
K200000450
0.3266
K100000467
0.3971
0.2868
K100000900
0.1525
K100001233
JJ00000822
0.1622
JJ00000903
0.2387
K100000788
0.1688
K100001312
0.2008
K100001332
K100000219
0.2813
K200000992
0.2545
0.3256
K200001433
K200000989
F300001125
0.1374
K200000881
0.191
K100000440
0.1454
0.2729
K200000399
K100001272

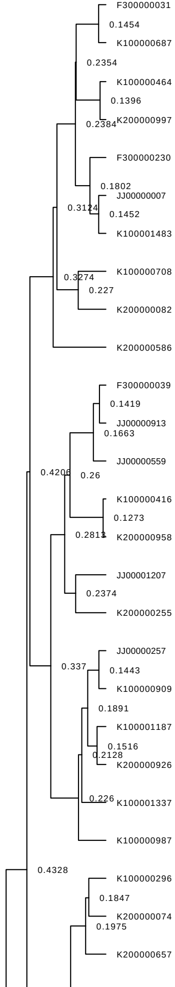
F300000031
0.1454
K100000687
0.2354
K100000464
0.1396
0.2384
K200000997
F300000230
0.1802
JJ00000007
0.3124
0.1452
K100001483
K100000708
0.3274
0.227
K200000082
K200000586
F300000039
0.1419
JJ00000913
0.1663
JJ00000559
0.4206
0.26
K100000416
0.1273
0.2813
K200000958
JJ00001207
0.2374
K200000255
JJ00000257
0.337
0.1443
K100000909
0.1891
K100001187
0.1516
0.2128
K200000926
0.226
K100001337
K100000987
0.4328
K100000296
0.1847
K200000074
0.1975
K200000657

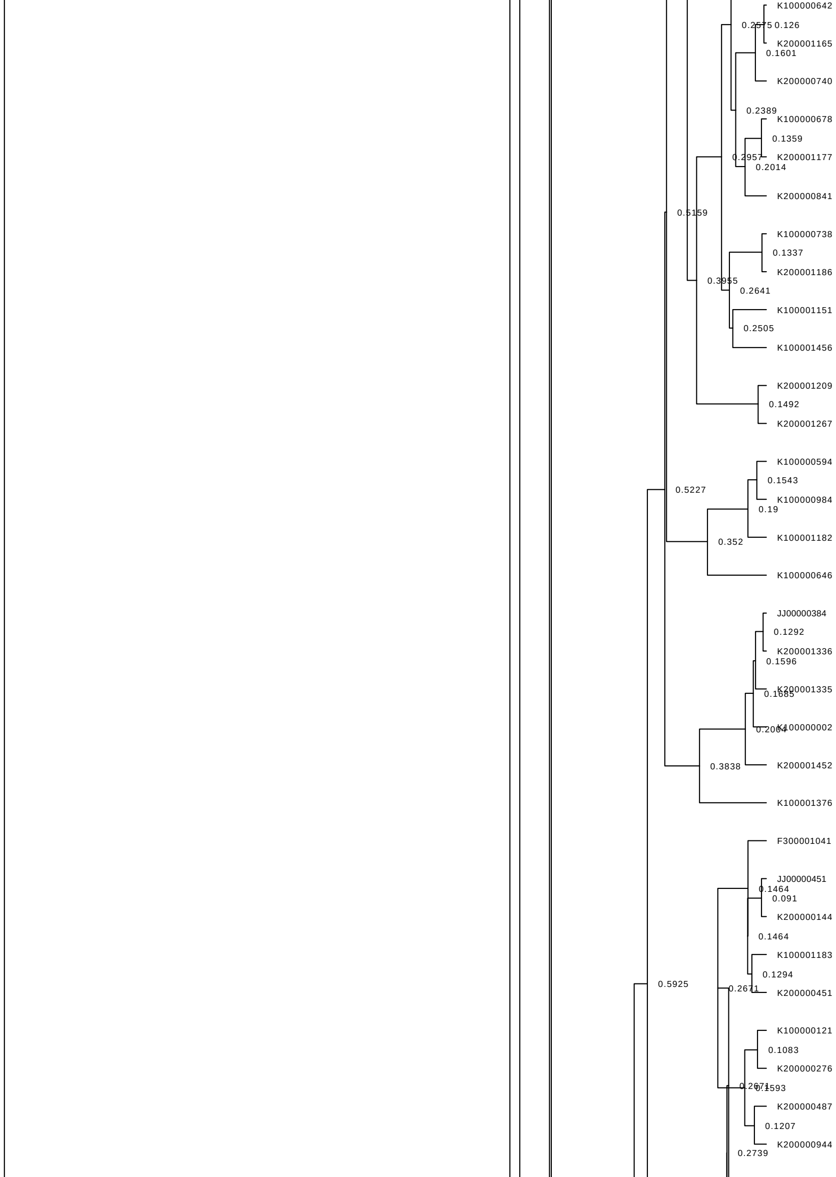

K100000642
0.2575
0.126
K200001165
0.1601
K200000740
0.2389
K100000678
0.1359
0.2957
K200001177
0.2014
K200000841
0.5159
K100000738
0.1337
K200001186
0.3955
0.2641
K100001151
0.2505
K100001456
K200001209
0.1492
K200001267
K100000594
0.1543
0.5227
K100000984
0.19
K100001182
0.352
K100000646
JJ00000384
0.1292
K200001336
0.1596
K200001335
0.1685
K100000002
0.2004
K200001452
0.3838
K100001376
F300001041
JJ00000451
0.1464
0.091
K200000144
0.1464
K100001183
0.1294
0.5925
0.2671
K200000451
K100000121
0.1083
K200000276
0.2671
0.1593
K200000487
0.1207
K200000944
0.2739

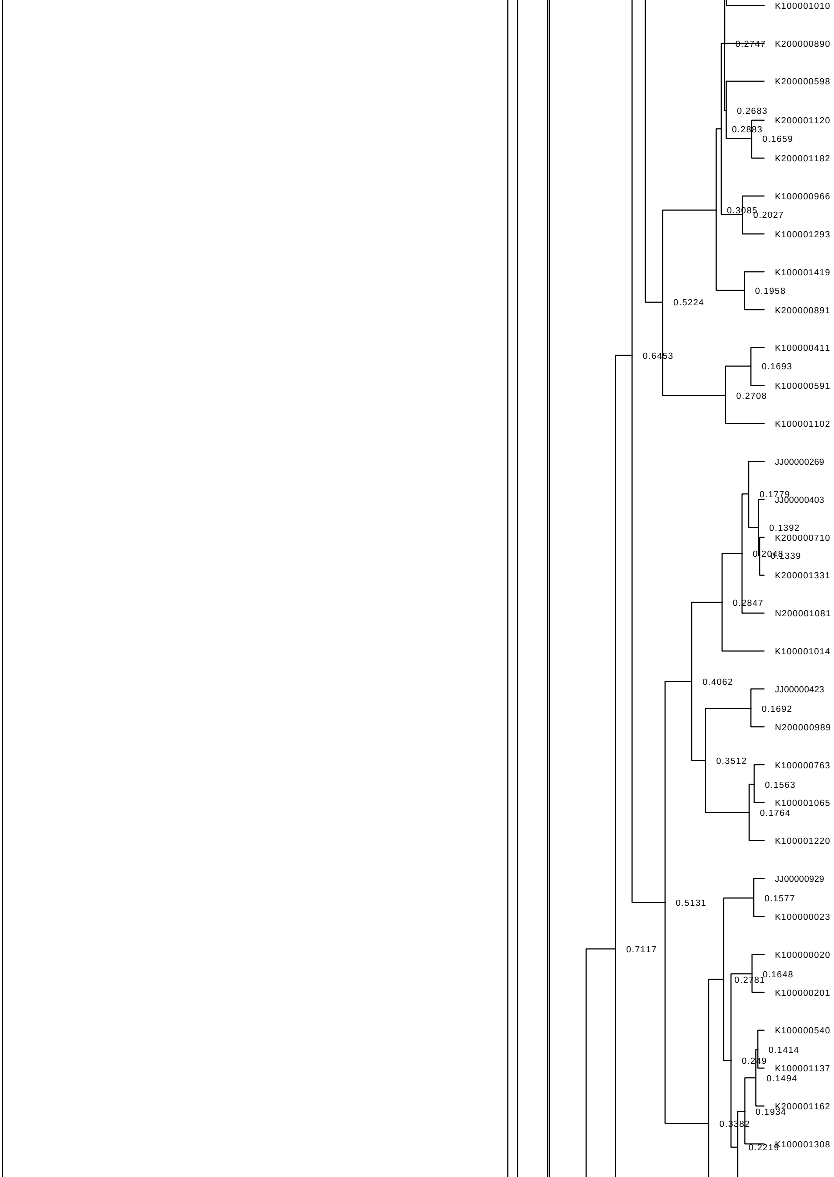

K100001010
0.2747
K200000890
K200000598
0.2683
K200001120
0.2883
0.1659
K200001182
K100000966
0.3085
0.2027
K100001293
K100001419
0.1958
0.5224
K200000891
K100000411
0.6453
0.1693
K100000591
0.2708
K100001102
JJ00000269
0.1779
JJ00000403
0.1392
K200000710
0.2048
0.1339
K200001331
0.2847
N200001081
K100001014
0.4062
JJ00000423
0.1692
N200000989
0.3512
K100000763
0.1563
K100001065
0.1764
K100001220
JJ00000929
0.1577
0.5131
K100000023
0.7117
K100000020
0.1648
0.2781
K100000201
K100000540
0.1414
0.249
K100001137
0.1494
K200001162
0.1934
0.3382
K100001308
0.2219

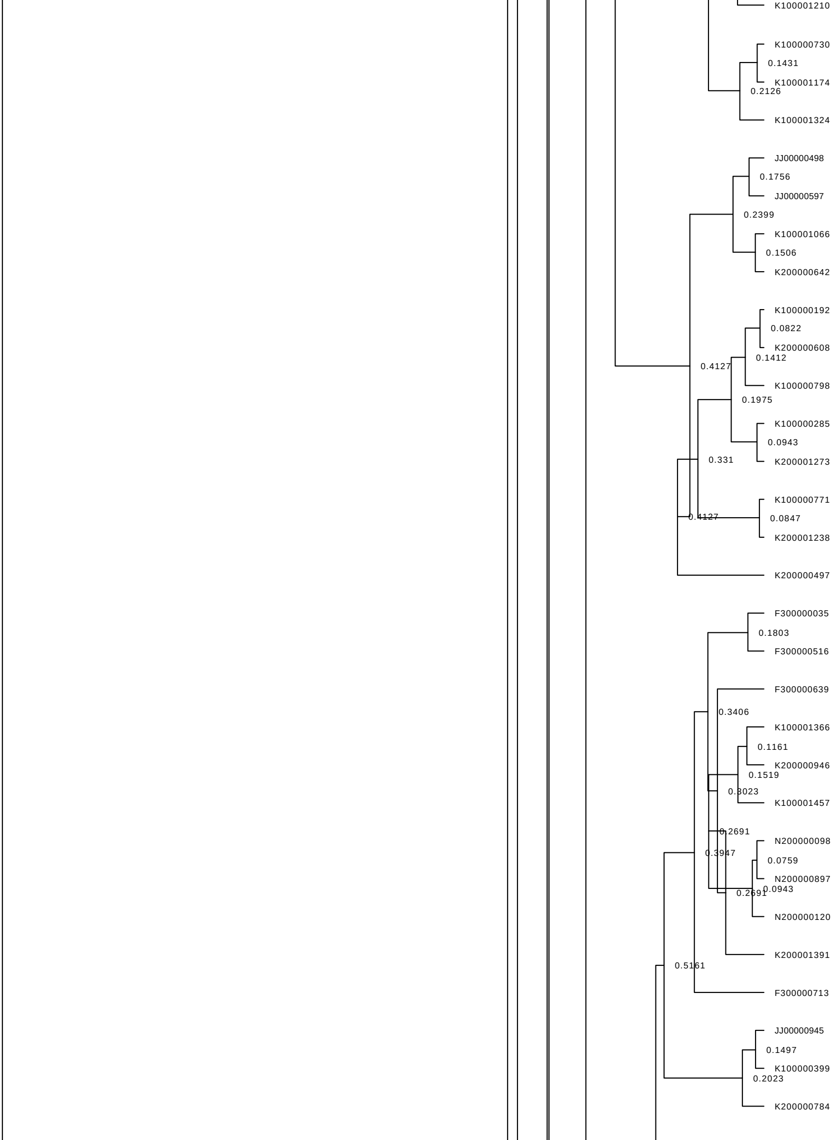
K100001210
K100000730
0.1431
K100001174
0.2126
K100001324
JJ00000498
0.1756
JJ00000597
0.2399
K100001066
0.1506
K200000642
K100000192
0.0822
K200000608
0.1412
0.4127
K100000798
0.1975
K100000285
0.0943
K200001273
0.331
K100000771
0.4127
0.0847
K200001238
K200000497
F300000035
0.1803
F300000516
F300000639
0.3406
K100001366
0.1161
K200000946
0.1519
0.3023
K100001457
0.2691
N200000098
0.3947
0.0759
N200000897
0.0943
0.2691
N200000120
K200001391
0.5161
F300000713
JJ00000945
0.1497
K100000399
0.2023
K200000784

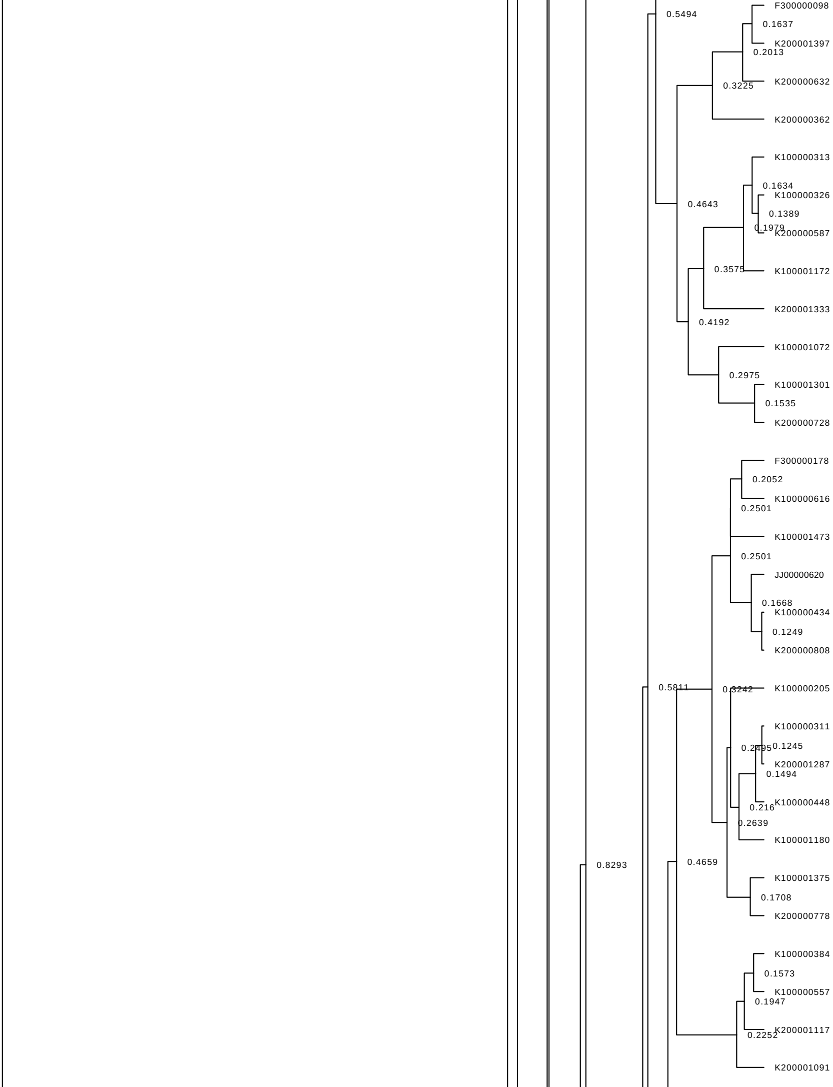

F300000098
0.5494
0.1637
K200001397
0.2013
K200000632
0.3225
K200000362
K100000313
0.1634
K100000326
0.4643
0.1389
0.1979
K200000587
0.3575
K100001172
K200001333
0.4192
K100001072
0.2975
K100001301
0.1535
K200000728
F300000178
0.2052
K100000616
0.2501
K100001473
0.2501
JJ00000620
0.1668
K100000434
0.1249
K200000808
0.5811
0.3242
K100000205
K100000311
0.2495
0.1245
K200001287
0.1494
K100000448
0.216
0.2639
K100001180
0.8293
0.4659
K100001375
0.1708
K200000778
K100000384
0.1573
K100000557
0.1947
K200001117
0.2252
K200001091

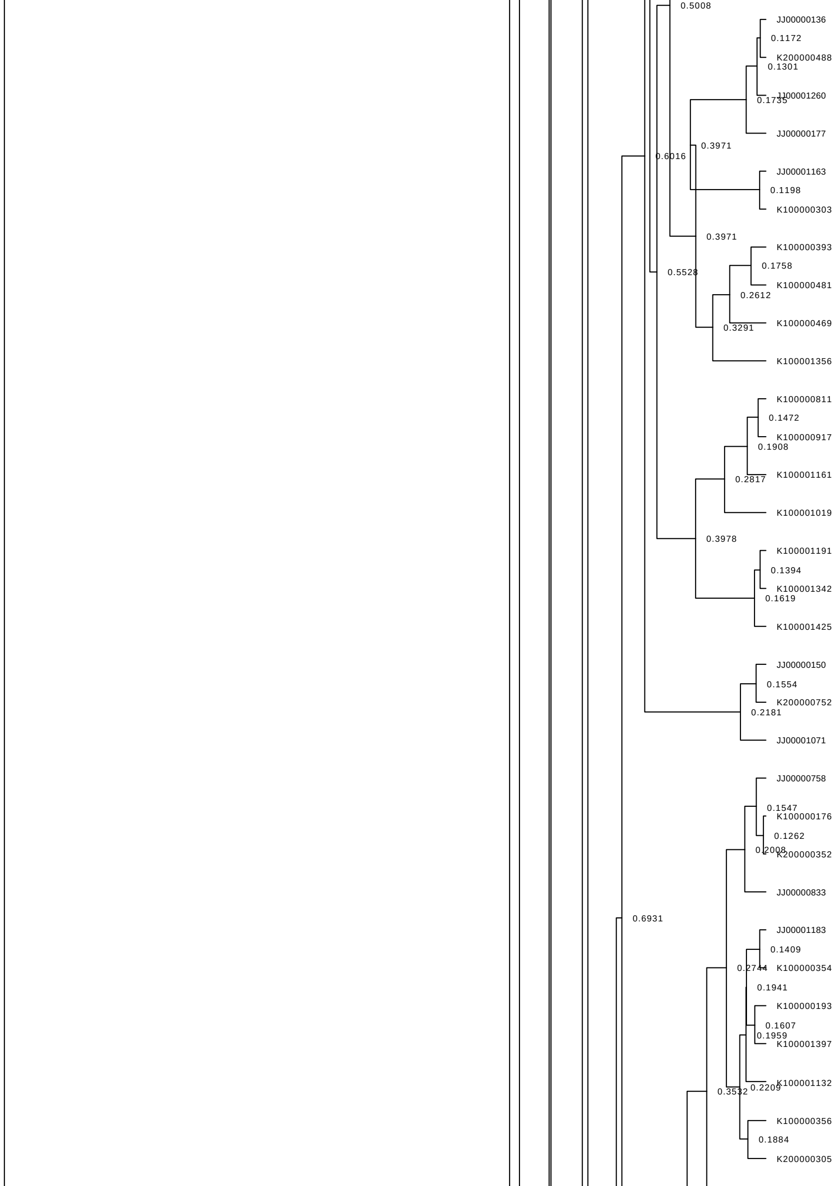
0.5008
JJ00000136
0.1172
K200000488
0.1301
JJ00001260
0.1735
JJ00000177
0.3971
0.6016
JJ00001163
0.1198
K100000303
0.3971
K100000393
0.1758
0.5528
K100000481
0.2612
K100000469
0.3291
K100001356
K100000811
0.1472
K100000917
0.1908
K100001161
0.2817
K100001019
0.3978
K100001191
0.1394
K100001342
0.1619
K100001425
JJ00000150
0.1554
K200000752
0.2181
JJ00001071
JJ00000758
0.1547
K100000176
0.1262
0.2008
K200000352
JJ00000833
0.6931
JJ00001183
0.1409
0.2744
K100000354
0.1941
K100000193
0.1607
0.1959
K100001397
K100001132
0.2209
0.3532
K100000356
0.1884
K200000305

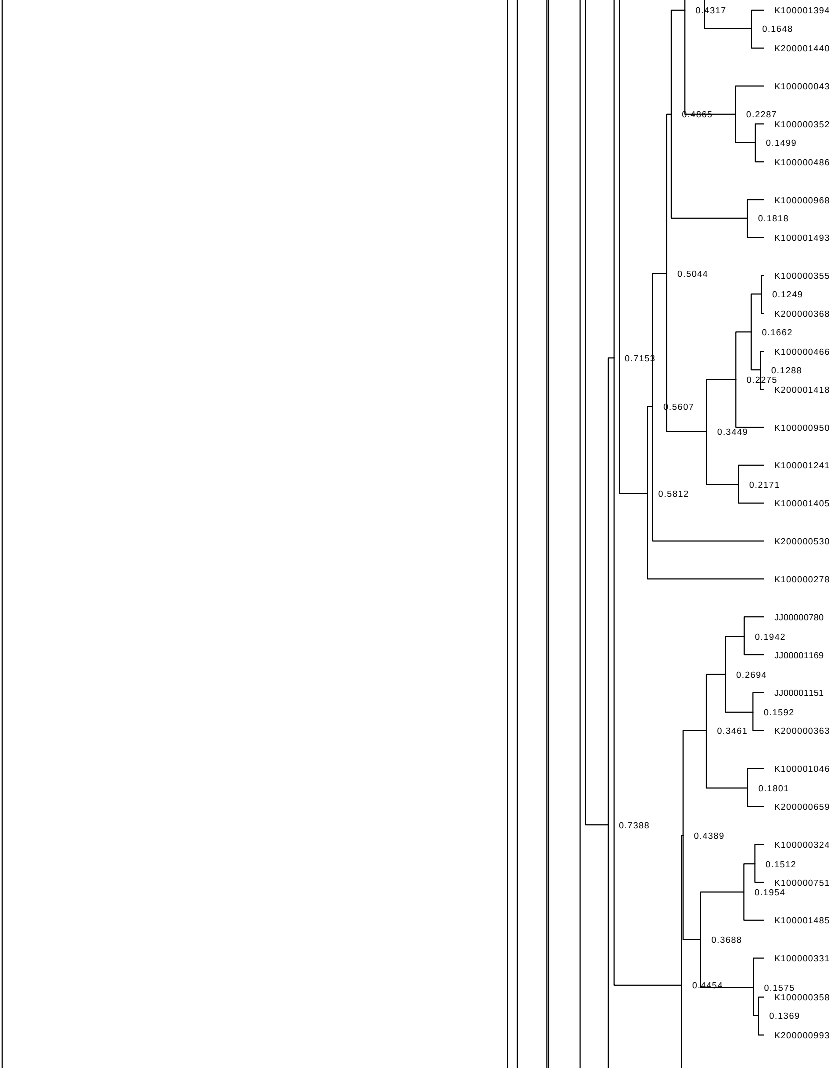
0.4317
K100001394
0.1648
K200001440
K100000043
0.4865
0.2287
K100000352
0.1499
K100000486
K100000968
0.1818
K100001493
0.5044
K100000355
0.1249
K200000368
0.1662
K100000466
0.7153
0.1288
0.2275
K200001418
0.5607
K100000950
0.3449
K100001241
0.2171
0.5812
K100001405
K200000530
K100000278
JJ00000780
0.1942
JJ00001169
0.2694
JJ00001151
0.1592
0.3461
K200000363
K100001046
0.1801
K200000659
0.7388
0.4389
K100000324
0.1512
K100000751
0.1954
K100001485
0.3688
K100000331
0.4454
0.1575
K100000358
0.1369
K200000993

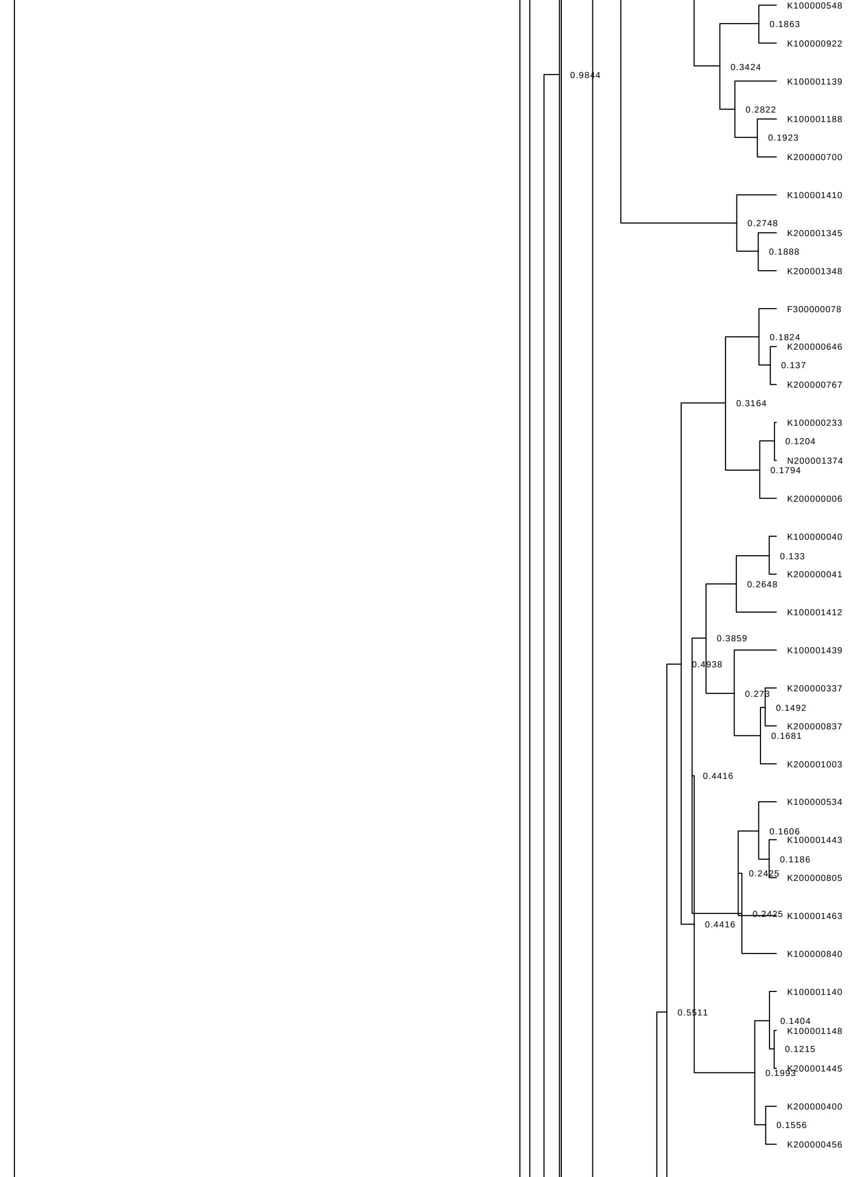
K100000548
0.1863
K100000922
0.9844
0.3424
K100001139
0.2822
K100001188
0.1923
K200000700
K100001410
0.2748
K200001345
0.1888
K200001348
F300000078
0.1824
K200000646
0.137
K200000767
0.3164
K100000233
0.1204
N200001374
0.1794
K200000006
K100000040
0.133
K200000041
0.2648
K100001412
0.3859
K100001439
0.4938
K200000337
0.273
0.1492
K200000837
0.1681
K200001003
0.4416
K100000534
0.1606
K100001443
0.1186
0.2425
K200000805
0.2425
K100001463
0.4416
K100000840
K100001140
0.5511
0.1404
K100001148
0.1215
K200001445
0.1993
K200000400
0.1556
K200000456

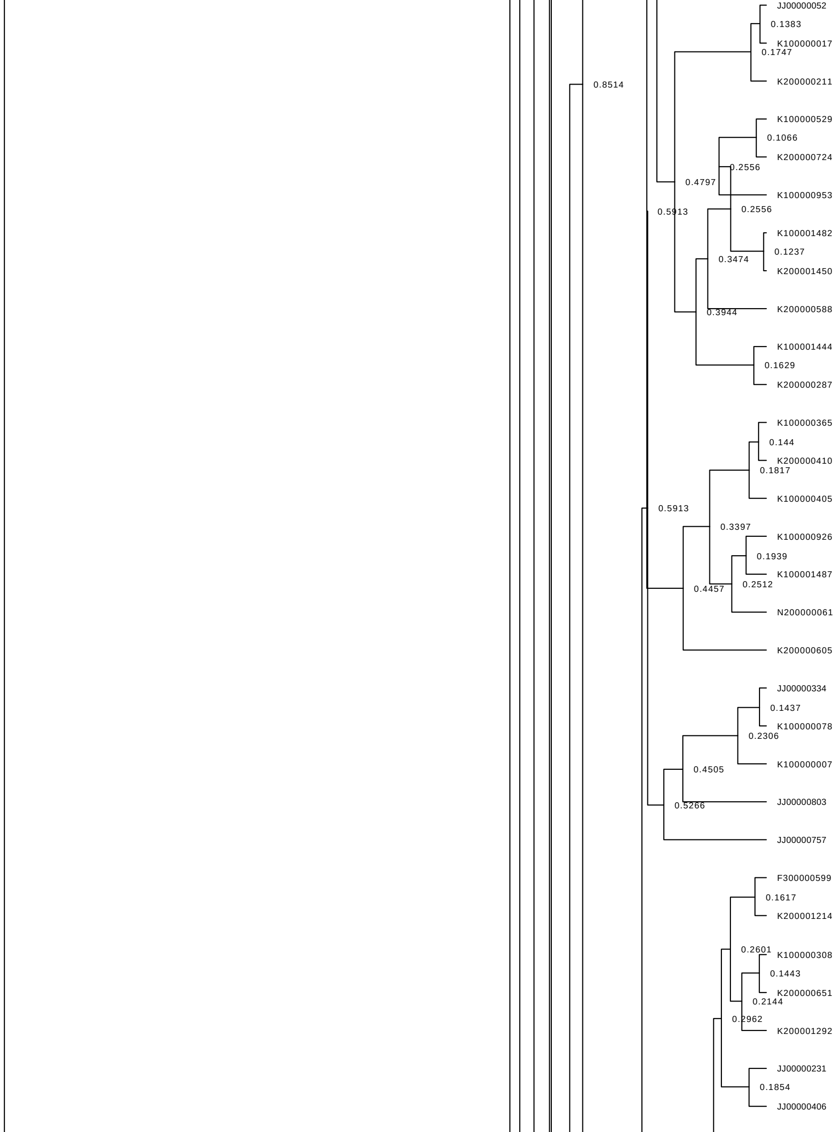

JJ00000052
0.1383
K100000017
0.1747
K200000211
0.8514
K100000529
0.1066
K200000724
0.2556
0.4797
K100000953
0.2556
0.5913
K100001482
0.1237
K200001450
0.3474
K200000588
0.3944
K100001444
0.1629
K200000287
K100000365
0.144
K200000410
0.1817
K100000405
0.5913
0.3397
K100000926
0.1939
K100001487
0.2512
0.4457
N200000061
K200000605
JJ00000334
0.1437
K100000078
0.2306
K100000007
0.4505
JJ00000803
0.5266
JJ00000757
F300000599
0.1617
K200001214
0.2601
K100000308
0.1443
K200000651
0.2144
0.2962
K200001292
JJ00000231
0.1854
JJ00000406

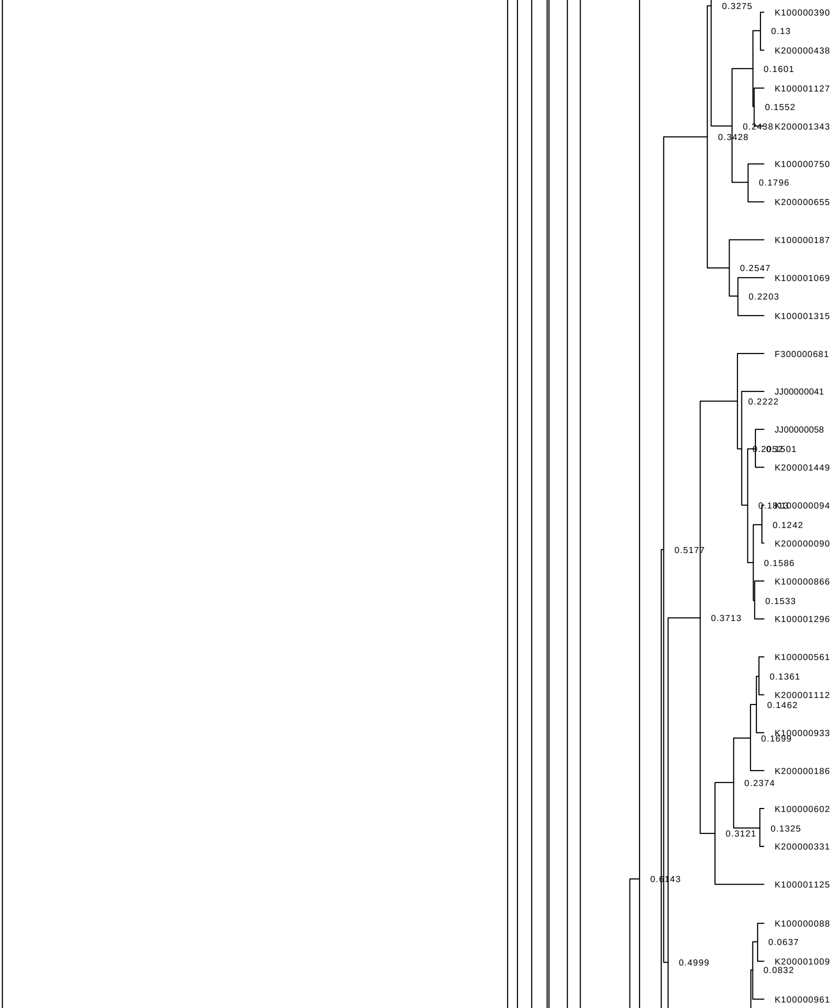
0.3275
K100000390
0.13
K200000438
0.1601
K100001127
0.1552
0.2438
K200001343
0.3428
K100000750
0.1796
K200000655
K100000187
0.2547
K100001069
0.2203
K100001315
F300000681
JJ00000041
0.2222
JJ00000058
K200001449
K100000094
0.1242
K200000090
0.5177
0.1586
K100000866
0.1533
0.3713
K100001296
K100000561
0.1361
K200001112
0.1462
K100000933
0.1699
K200000186
0.2374
K100000602
0.1325
0.3121
K200000331
0.6143
K100001125
K100000088
0.0637
0.4999
K200001009
0.0832
K100000961

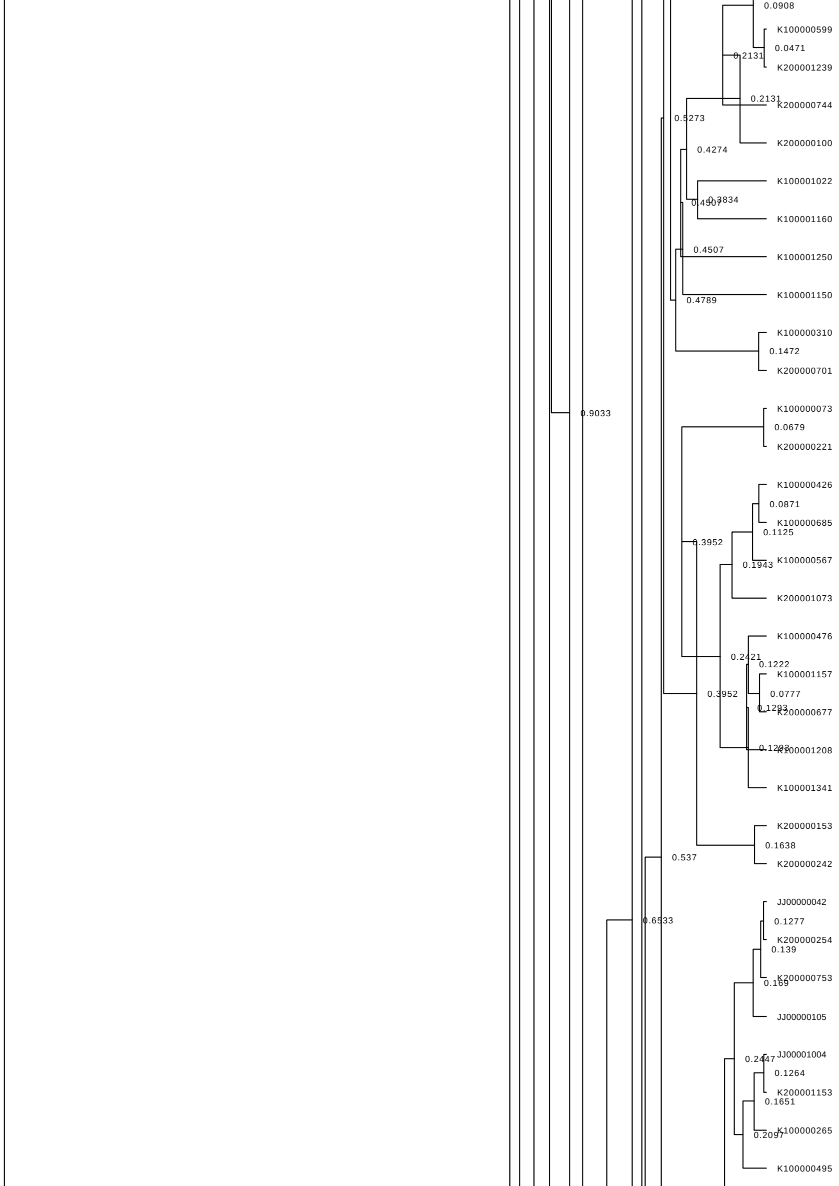
0.0908
K100000599
0.0471
0.2131
K200001239
0.2131
K200000744
0.5273
K200000100
0.4274
K100001022
0.3834
0.4507
K100001160
0.4507
K100001250
K100001150
0.4789
K100000310
0.1472
K200000701
K100000073
0.9033
0.0679
K200000221
K100000426
0.0871
K100000685
0.1125
0.3952
K100000567
0.1943
K200001073
K100000476
0.2421
0.1222
K100001157
0.3952
0.0777
0.1293
K200000677
0.1293
K100001208
K100001341
K200000153
0.1638
0.537
K200000242
JJ00000042
0.6533
0.1277
K200000254
0.139
K200000753
0.169
JJ00000105
JJ00001004
0.2447
0.1264
K200001153
0.1651
K100000265
0.2097
K100000495

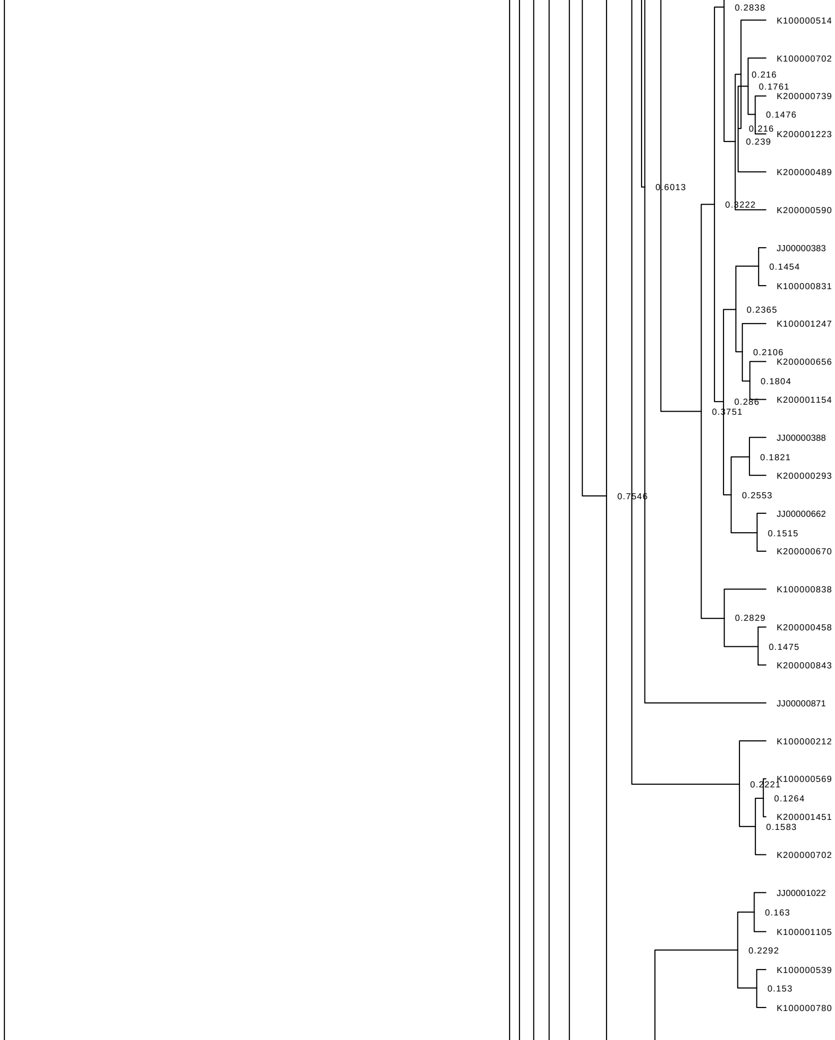

0.2838
K100000514
K100000702
0.216
0.1761
K200000739
0.1476
0.216
K200001223
0.239
K200000489
0.6013
0.3222
K200000590
JJ00000383
0.1454
K100000831
0.2365
K100001247
0.2106
K200000656
0.1804
K200001154
0.286
0.3751
JJ00000388
0.1821
K200000293
0.7546
0.2553
JJ00000662
0.1515
K200000670
K100000838
0.2829
K200000458
0.1475
K200000843
JJ00000871
K100000212
K100000569
0.2221
0.1264
K200001451
0.1583
K200000702
JJ00001022
0.163
K100001105
0.2292
K100000539
0.153
K100000780

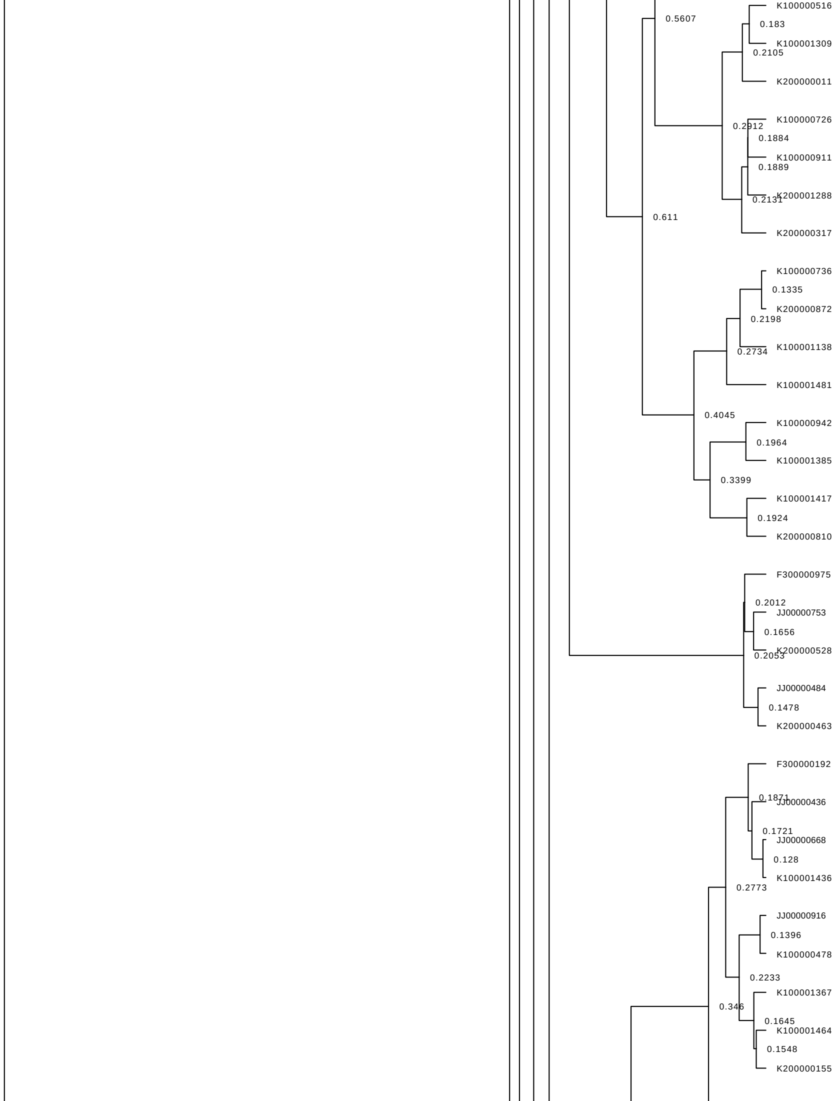
K100000516
0.5607
0.183
K100001309
0.2105
K200000011
K100000726
0.2912
0.1884
K100000911
0.1889
K200001288
0.2131
0.611
K200000317
K100000736
0.1335
K200000872
0.2198
K100001138
0.2734
K100001481
0.4045
K100000942
0.1964
K100001385
0.3399
K100001417
0.1924
K200000810
F300000975
0.2012
JJ00000753
0.1656
K200000528
0.2053
JJ00000484
0.1478
K200000463
F300000192
0.1871
JJ00000436
0.1721
JJ00000668
0.128
K100001436
0.2773
JJ00000916
0.1396
K100000478
0.2233
K100001367
0.346
0.1645
K100001464
0.1548
K200000155

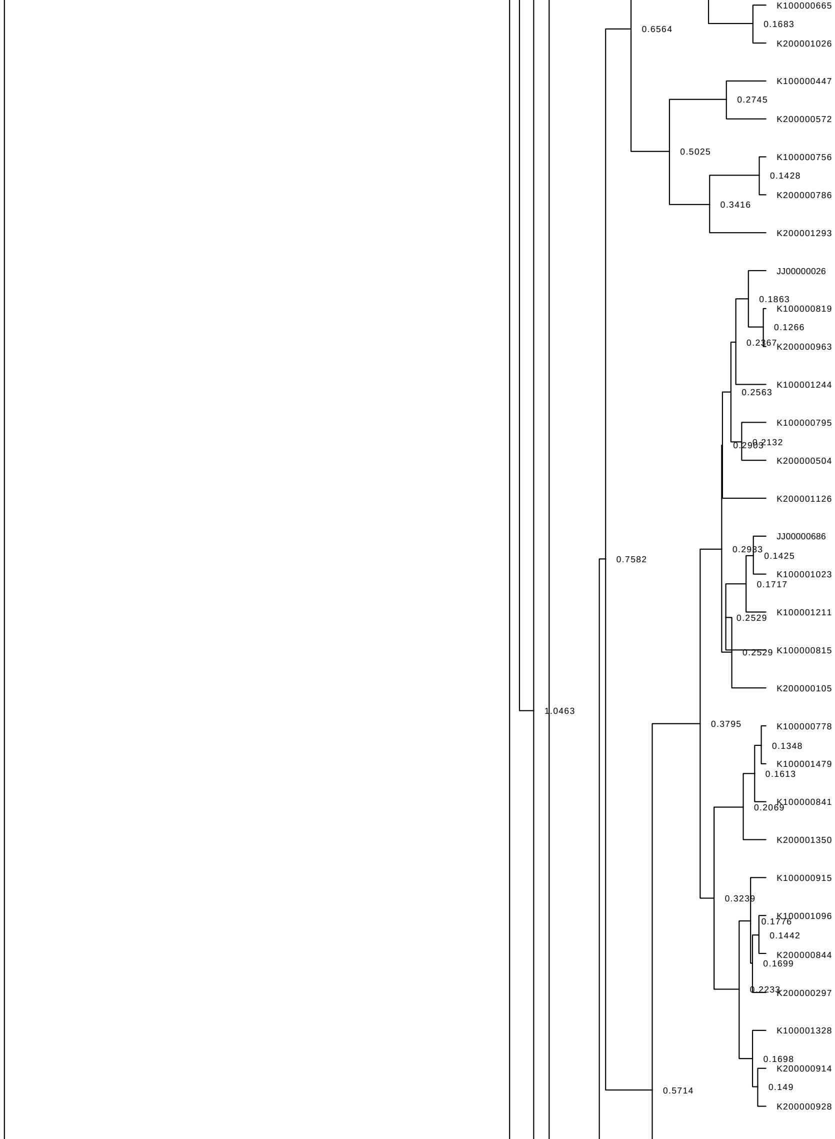
K100000665
0.1683
0.6564
K200001026
K100000447
0.2745
K200000572
0.5025
K100000756
0.1428
K200000786
0.3416
K200001293
JJ00000026
0.1863
K100000819
0.1266
0.2367
K200000963
K100001244
0.2563
K100000795
0.2903
0.2132
K200000504
K200001126
JJ00000686
0.2933
0.1425
0.7582
K100001023
0.1717
K100001211
0.2529
K100000815
0.2529
K200000105
1.0463
0.3795
K100000778
0.1348
K100001479
0.1613
K100000841
0.2069
K200001350
K100000915
0.3239
K100001096
0.1776
0.1442
K200000844
0.1699
0.2233
K200000297
K100001328
0.1698
K200000914
0.149
0.5714
K200000928

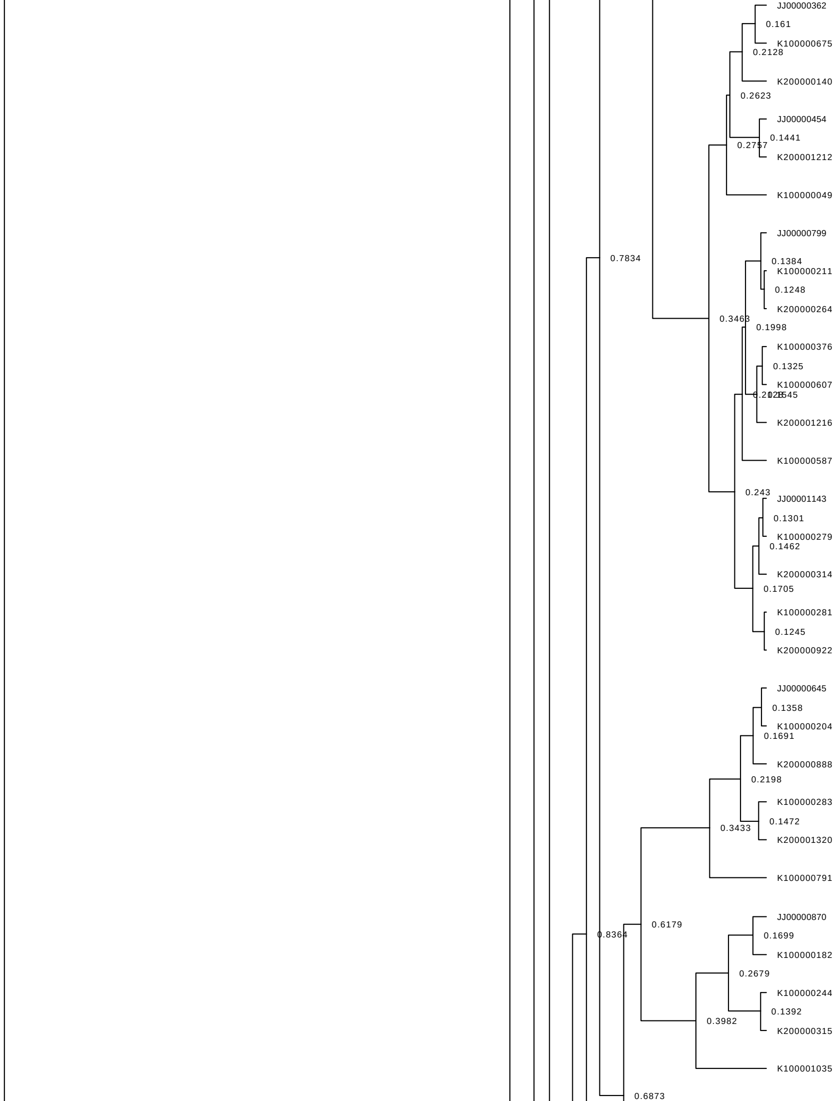
JJ00000362
0.161
K100000675
0.2128
K200000140
0.2623
JJ00000454
0.1441
0.2757
K200001212
K100000049
JJ00000799
0.7834
0.1384
K100000211
0.1248
K200000264
0.3463
0.1998
K100000376
0.1325
K100000607
0.1545
K200001216
K100000587
0.243
JJ00001143
0.1301
K100000279
0.1462
K200000314
0.1705
K100000281
0.1245
K200000922
JJ00000645
0.1358
K100000204
0.1691
K200000888
0.2198
K100000283
0.1472
0.3433
K200001320
K100000791
JJ00000870
0.6179
0.8364
0.1699
K100000182
0.2679
K100000244
0.1392
0.3982
K200000315
K100001035
0.6873

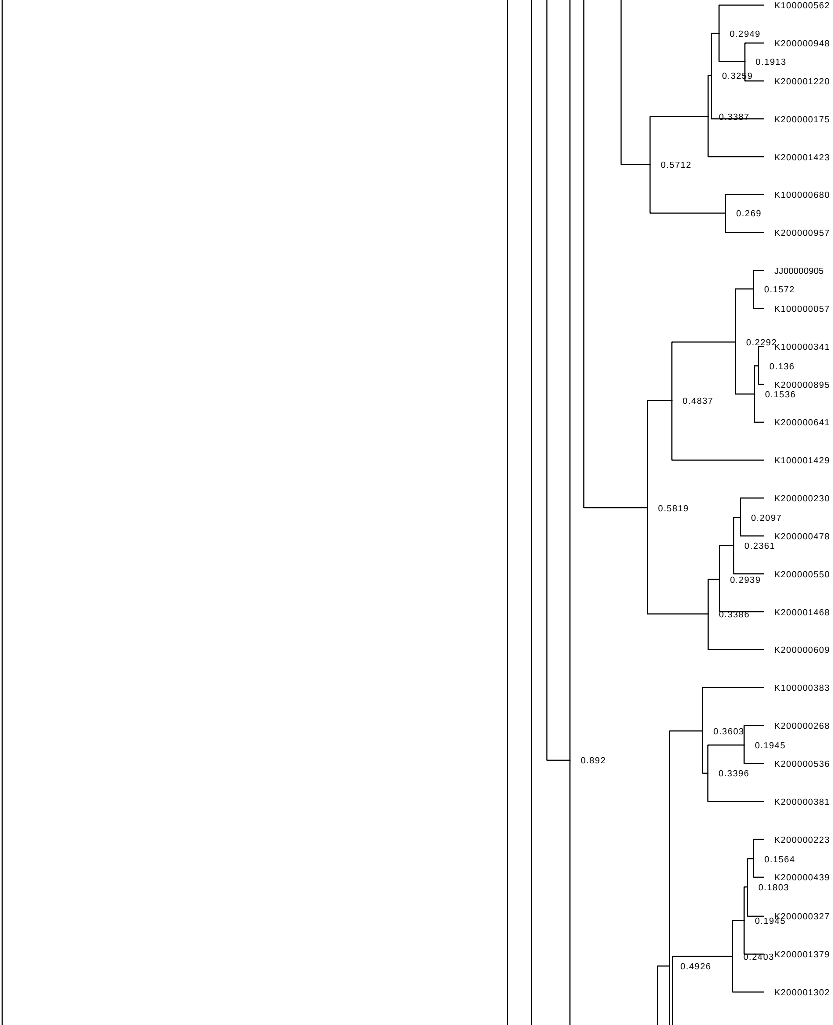
K100000562
0.2949
K200000948
0.1913
0.3259
K200001220
0.3387
K200000175
K200001423
0.5712
K100000680
0.269
K200000957
JJ00000905
0.1572
K100000057
0.2292
K100000341
0.136
K200000895
0.1536
0.4837
K200000641
K100001429
K200000230
0.5819
0.2097
K200000478
0.2361
K200000550
0.2939
K200001468
0.3386
K200000609
K100000383
0.3603
K200000268
0.1945
0.892
K200000536
0.3396
K200000381
K200000223
0.1564
K200000439
0.1803
K200000327
0.1945
K200001379
0.2403
0.4926
K200001302

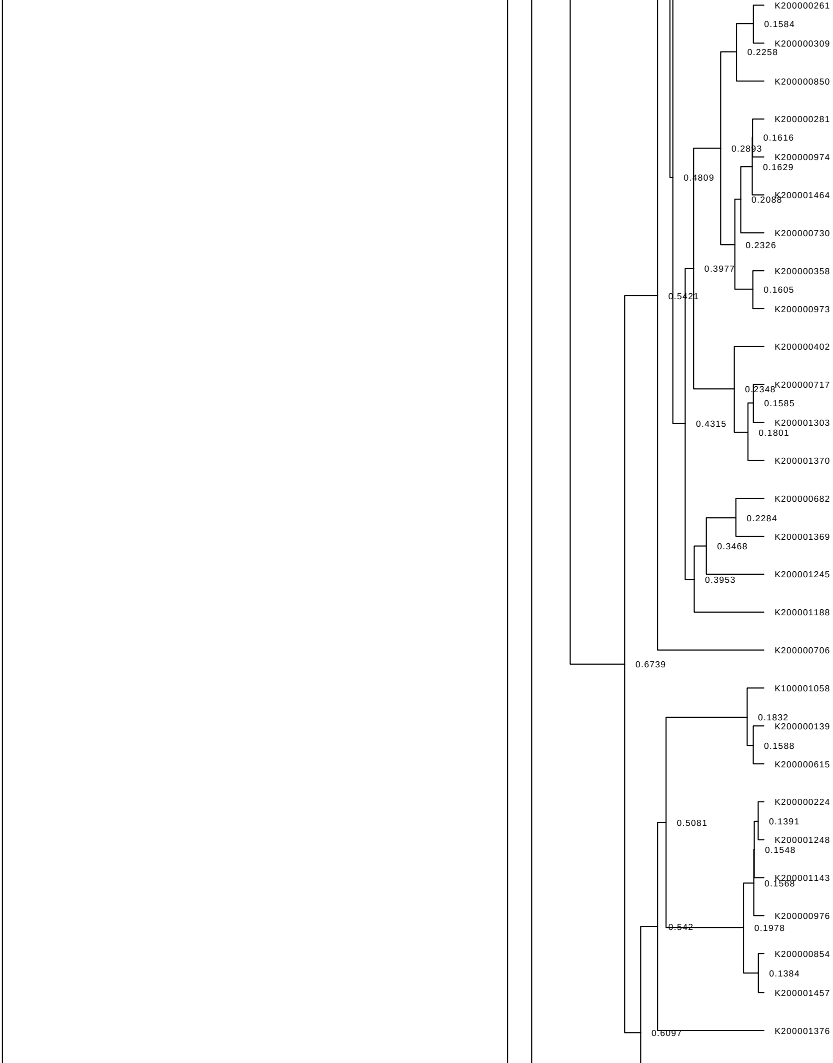
K200000261
0.1584
K200000309
0.2258
K200000850
K200000281
0.1616
0.2893
K200000974
0.1629
0.4809
K200001464
0.2088
K200000730
0.2326
0.3977
K200000358
0.1605
0.5421
K200000973
K200000402
0.2348
K200000717
0.1585
K200001303
0.4315
0.1801
K200001370
K200000682
0.2284
K200001369
0.3468
K200001245
0.3953
K200001188
K200000706
0.6739
K100001058
0.1832
K200000139
0.1588
K200000615
K200000224
0.1391
0.5081
K200001248
0.1548
K200001143
0.1568
K200000976
0.542
0.1978
K200000854
0.1384
K200001457
K200001376
0.6097

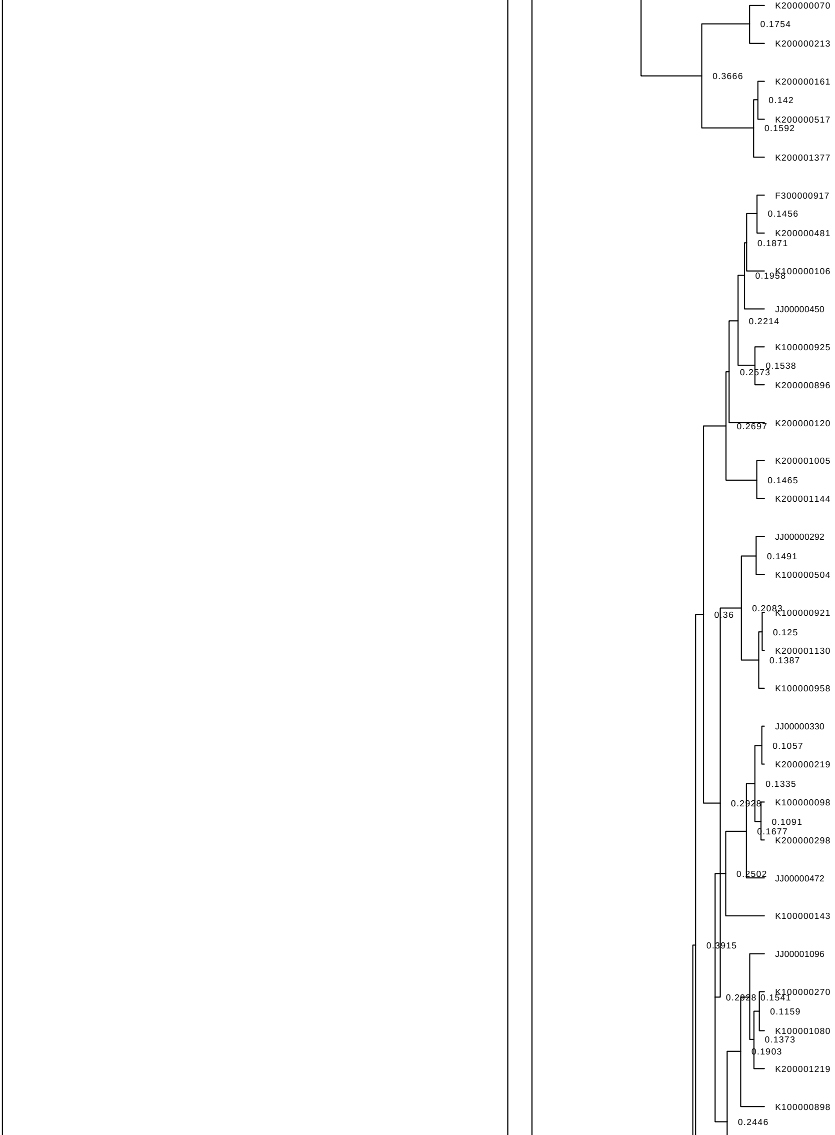
K200000070
0.1754
K200000213
0.3666
K200000161
0.142
K200000517
0.1592
K200001377
F300000917
0.1456
K200000481
0.1871
K100000106
0.1958
JJ00000450
0.2214
K100000925
0.1538
0.2573
K200000896
K200000120
0.2697
K200001005
0.1465
K200001144
JJ00000292
0.1491
K100000504
0.2083
0.36
K100000921
0.125
K200001130
0.1387
K100000958
JJ00000330
0.1057
K200000219
0.1335
0.2928
K100000098
0.1091
0.1677
K200000298
0.2502
JJ00000472
K100000143
0.3915
JJ00001096
K100000270
0.2928
0.1541
0.1159
K100001080
0.1373
0.1903
K200001219
K100000898
0.2446

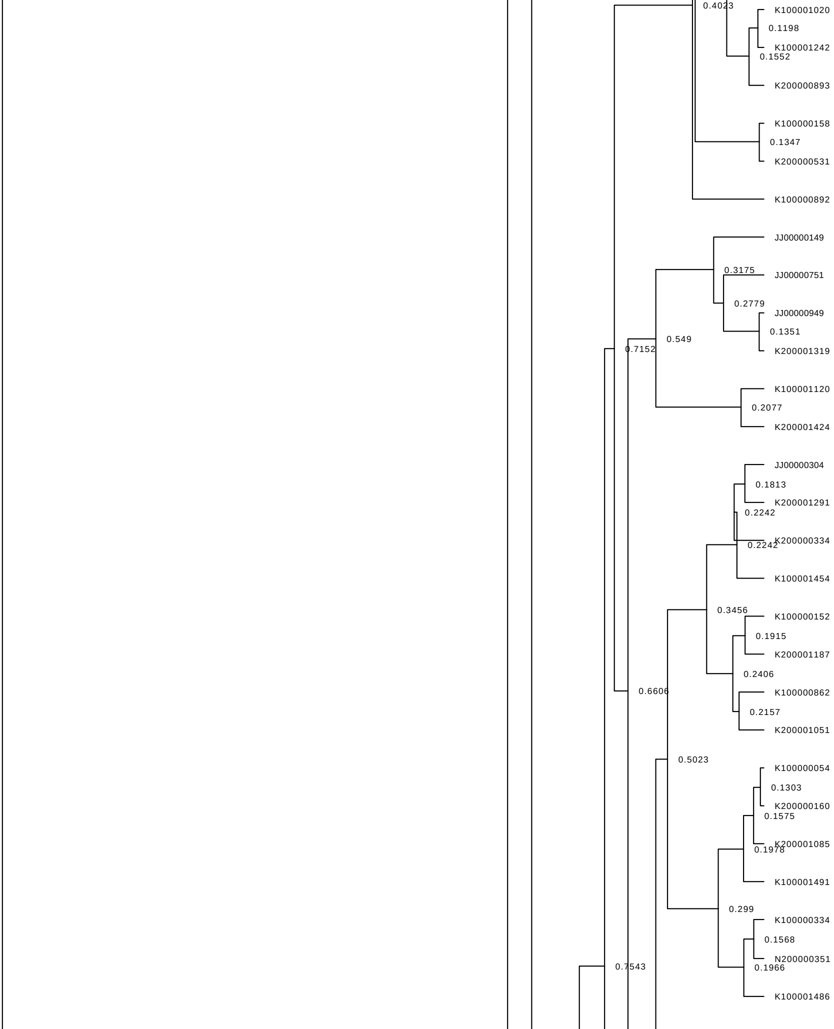
0.4023
K100001020
0.1198
K100001242
0.1552
K200000893
K100000158
0.1347
K200000531
K100000892
JJ00000149
0.3175
JJ00000751
0.2779
JJ00000949
0.1351
0.549
0.7152
K200001319
K100001120
0.2077
K200001424
JJ00000304
0.1813
K200001291
0.2242
K200000334
0.2242
K100001454
0.3456
K100000152
0.1915
K200001187
0.2406
0.6606
K100000862
0.2157
K200001051
0.5023
K100000054
0.1303
K200000160
0.1575
K200001085
0.1978
K100001491
0.299
K100000334
0.1568
N200000351
0.1966
0.7543
K100001486

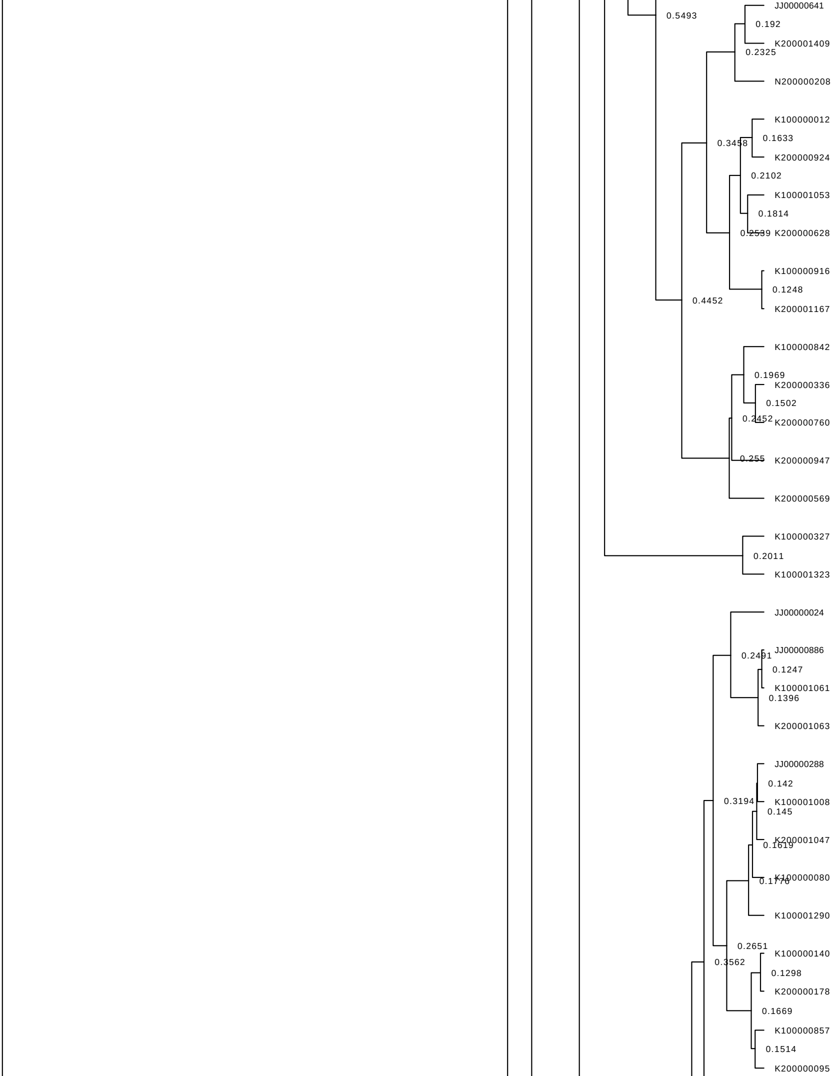

JJ00000641
0.5493
0.192
K200001409
0.2325
N200000208
K100000012
0.1633
0.3458
K200000924
0.2102
K100001053
0.1814
0.2539
K200000628
K100000916
0.1248
0.4452
K200001167
K100000842
0.1969
K200000336
0.1502
0.2452
K200000760
0.255
K200000947
K200000569
K100000327
0.2011
K100001323
JJ00000024
0.2491
JJ00000886
0.1247
K100001061
0.1396
K200001063
JJ00000288
0.142
0.3194
K100001008
0.145
K200001047
0.1619
K100000080
0.1776
K100001290
0.2651
K100000140
0.3562
0.1298
K200000178
0.1669
K100000857
0.1514
K200000095

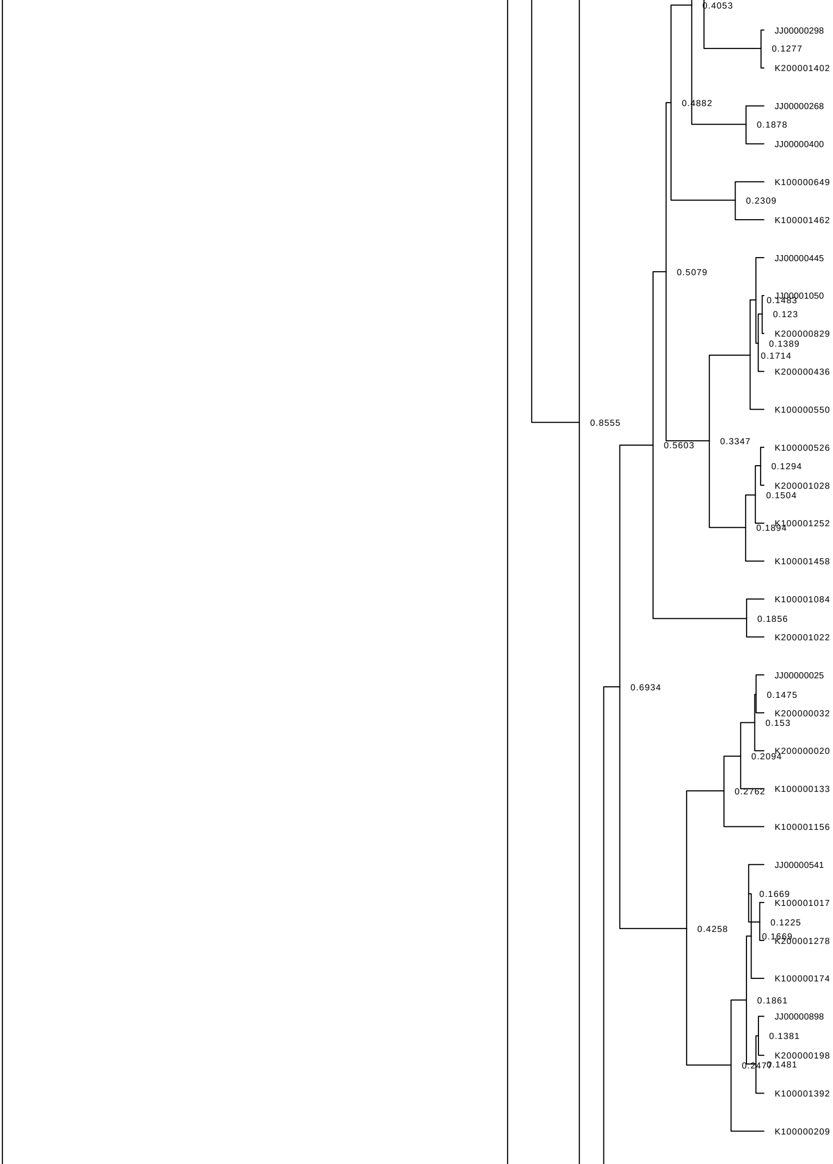
0.4053
JJ00000298
0.1277
K200001402
0.4882
JJ00000268
0.1878
JJ00000400
K100000649
0.2309
K100001462
JJ00000445
0.5079
JJ00001050
0.1483
0.123
K200000829
0.1389
0.1714
K200000436
K100000550
0.8555
0.3347
0.5603
K100000526
0.1294
K200001028
0.1504
K100001252
0.1894
K100001458
K100001084
0.1856
K200001022
JJ00000025
0.6934
0.1475
K200000032
0.153
K200000020
0.2094
K100000133
0.2762
K100001156
JJ00000541
0.1669
K100001017
0.1225
0.4258
0.1669
K200001278
K100000174
0.1861
JJ00000898
0.1381
K200000198
0.2470
0.1481
K100001392
K100000209

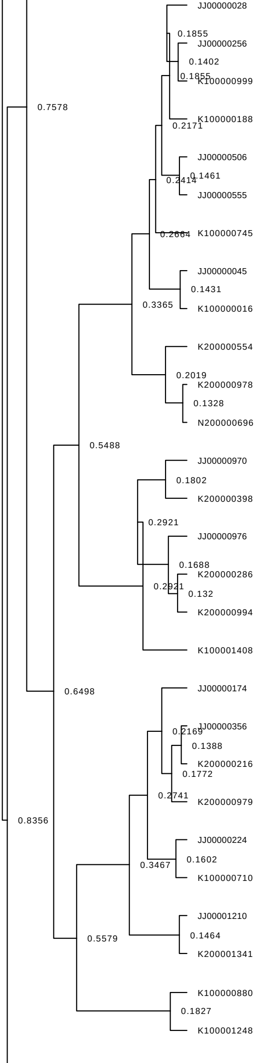
JJ00000028
0.1855
JJ00000256
0.1402
0.1855
K100000999
0.7578
K100000188
0.2171
JJ00000506
0.2414
0.1461
JJ00000555
0.2664
K100000745
JJ00000045
0.1431
0.3365
K100000016
K200000554
0.2019
K200000978
0.1328
N200000696
0.5488
JJ00000970
0.1802
K200000398
0.2921
JJ00000976
0.1688
K200000286
0.2921
0.132
K200000994
K100001408
0.6498
JJ00000174
JJ00000356
0.2169
0.1388
K200000216
0.1772
0.2741
K200000979
0.8356
JJ00000224
0.3467
0.1602
K100000710
JJ00001210
0.1464
0.5579
K200001341
K100000880
0.1827
K100001248

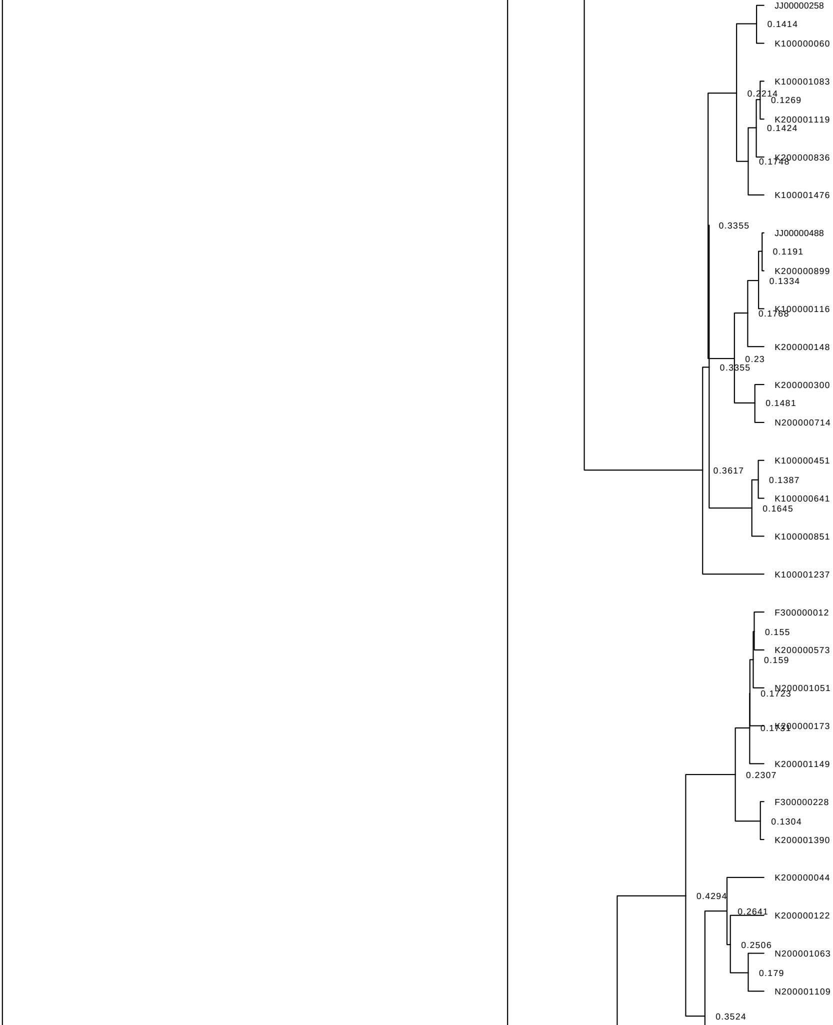

JJ00000258
0.1414
K100000060
K100001083
0.2214
0.1269
K200001119
0.1424
K200000836
0.1748
K100001476
0.3355
JJ00000488
0.1191
K200000899
0.1334
K100000116
0.1768
K200000148
0.23
0.3355
K200000300
0.1481
N200000714
K100000451
0.3617
0.1387
K100000641
0.1645
K100000851
K100001237
F300000012
0.155
K200000573
0.159
N200001051
0.1723
K200000173
0.1731
K200001149
0.2307
F300000228
0.1304
K200001390
K200000044
0.4294
0.2641
K200000122
0.2506
N200001063
0.179
N200001109
0.3524

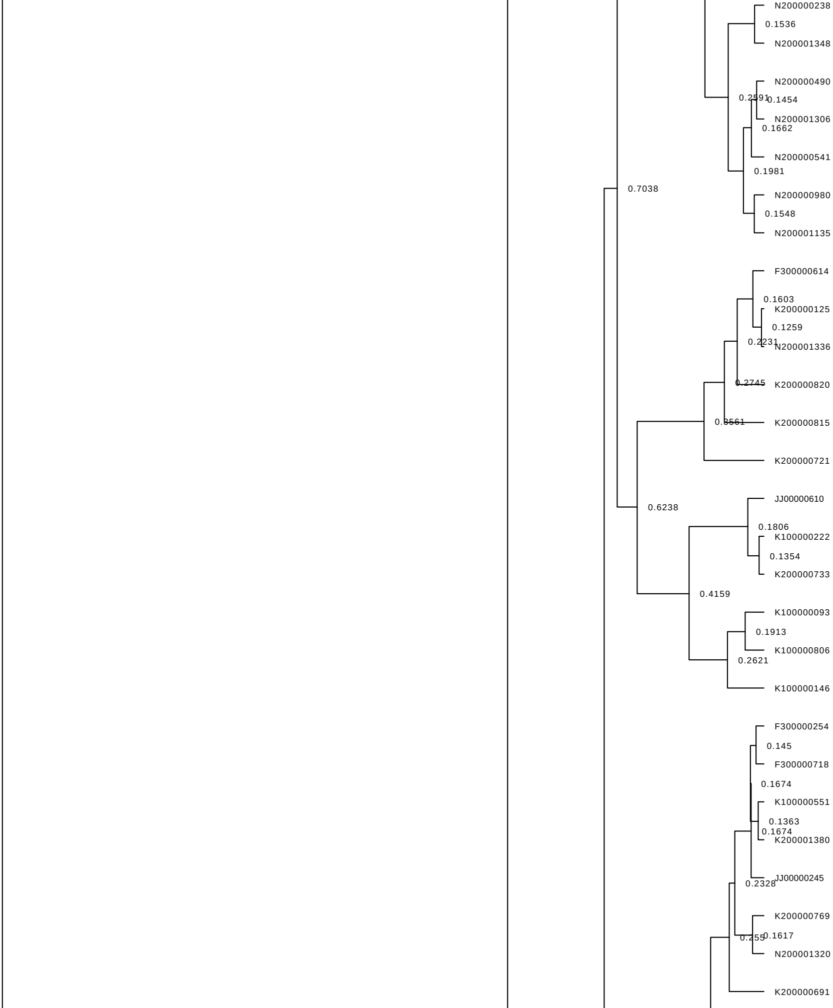

N200000238
0.1536
N200001348
N200000490
0.2591
0.1454
N200001306
0.1662
N200000541
0.1981
0.7038
N200000980
0.1548
N200001135
F300000614
0.1603
K200000125
0.1259
0.2231
N200001336
0.2745
K200000820
0.3561
K200000815
K200000721
JJ00000610
0.6238
0.1806
K100000222
0.1354
K200000733
0.4159
K100000093
0.1913
K100000806
0.2621
K100000146
F300000254
0.145
F300000718
0.1674
K100000551
0.1363
0.1674
K200001380
JJ00000245
0.2328
K200000769
0.255
0.1617
N200001320
K200000691

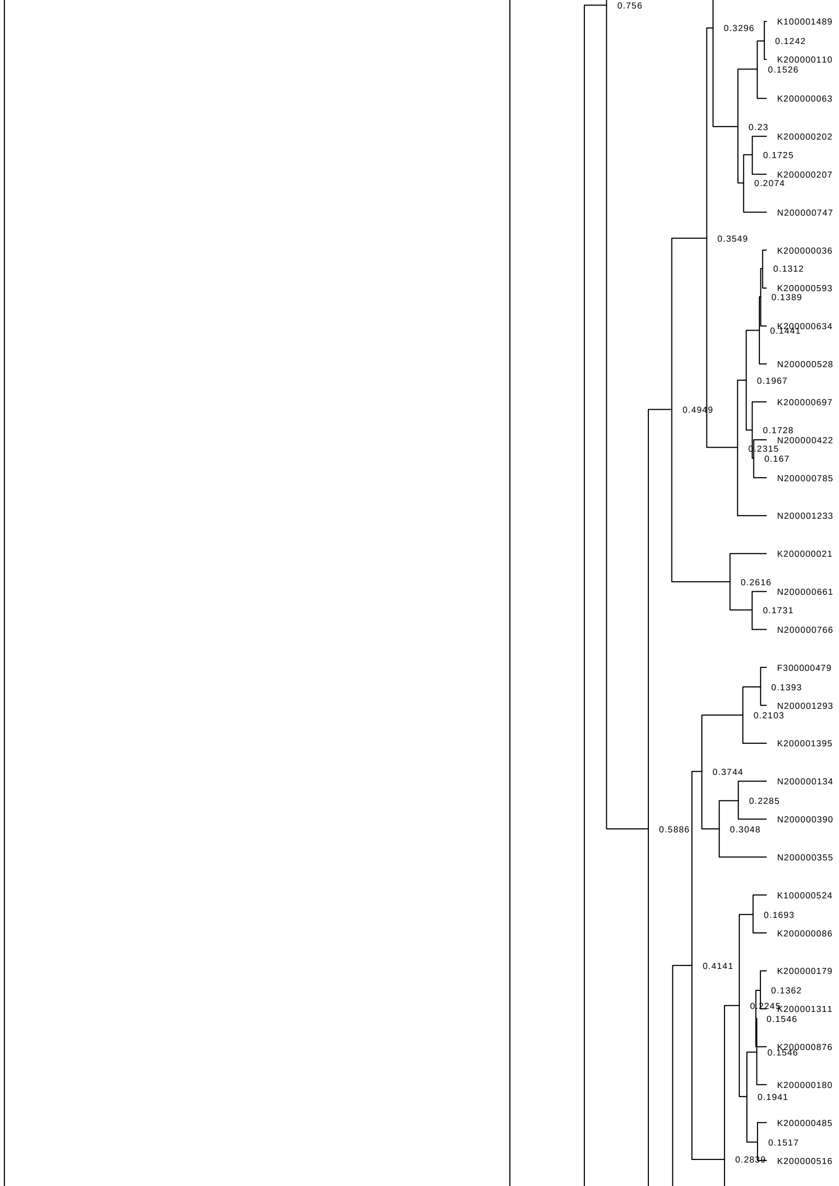
0.756
K100001489
0.3296
0.1242
K200000110
0.1526
K200000063
0.23
K200000202
0.1725
K200000207
0.2074
N200000747
0.3549
K200000036
0.1312
K200000593
0.1389
K200000634
0.1441
N200000528
0.1967
K200000697
0.4949
0.1728
N200000422
0.2315
0.167
N200000785
N200001233
K200000021
0.2616
N200000661
0.1731
N200000766
F300000479
0.1393
N200001293
0.2103
K200001395
0.3744
N200000134
0.2285
N200000390
0.5886
0.3048
N200000355
K100000524
0.1693
K200000086
0.4141
K200000179
0.1362
0.2245
K200001311
0.1546
K200000876
0.1546
K200000180
0.1941
K200000485
0.1517
0.2839
K200000516

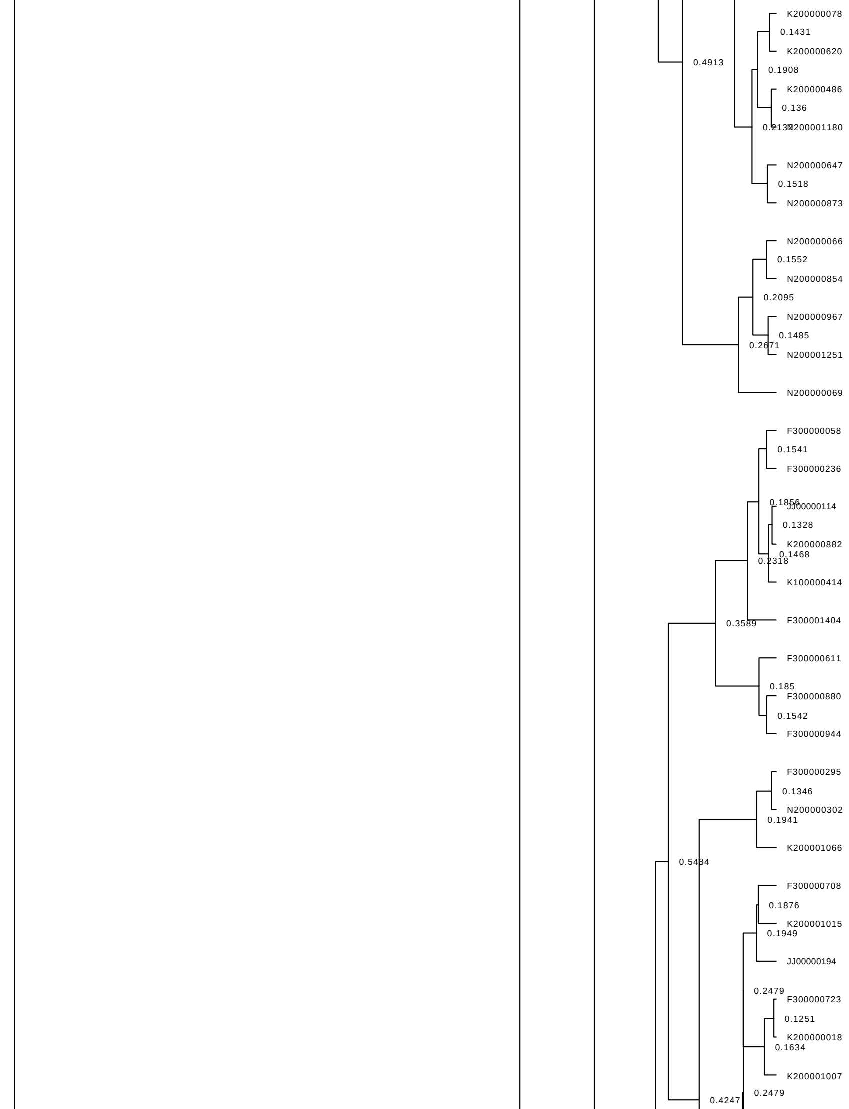

K200000078
0.1431
K200000620
0.1908
K200000486
0.136
0.213
N200001180
N200000647
0.1518
N200000873
0.4913
N200000066
0.1552
N200000854
0.2095
N200000967
0.1485
0.2671
N200001251
N200000069
F300000058
0.1541
F300000236
0.1856
JJ00000114
0.1328
K200000882
0.1468
0.2318
K100000414
F300001404
0.3589
F300000611
0.185
F300000880
0.1542
F300000944
F300000295
0.1346
N200000302
0.1941
K200001066
0.5484
F300000708
0.1876
K200001015
0.1949
JJ00000194
0.2479
F300000723
0.1251
K200000018
0.1634
K200001007
0.2479
0.4247

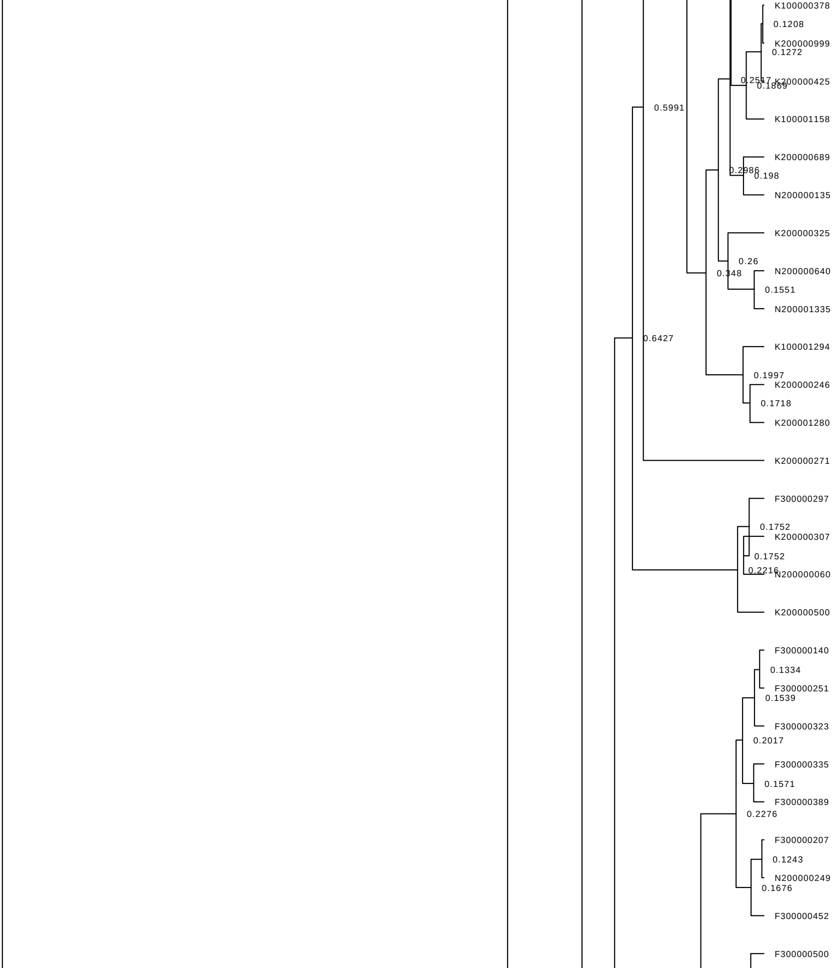
K100000378
0.1208
K200000999
0.1272
0.2547
K200000425
0.1869
0.5991
K100001158
K200000689
0.2986
0.198
N200000135
K200000325
0.26
0.348
N200000640
0.1551
N200001335
0.6427
K100001294
0.1997
K200000246
0.1718
K200001280
K200000271
F300000297
0.1752
K200000307
0.1752
0.2216
N200000060
K200000500
F300000140
0.1334
F300000251
0.1539
F300000323
0.2017
F300000335
0.1571
F300000389
0.2276
F300000207
0.1243
N200000249
0.1676
F300000452
F300000500

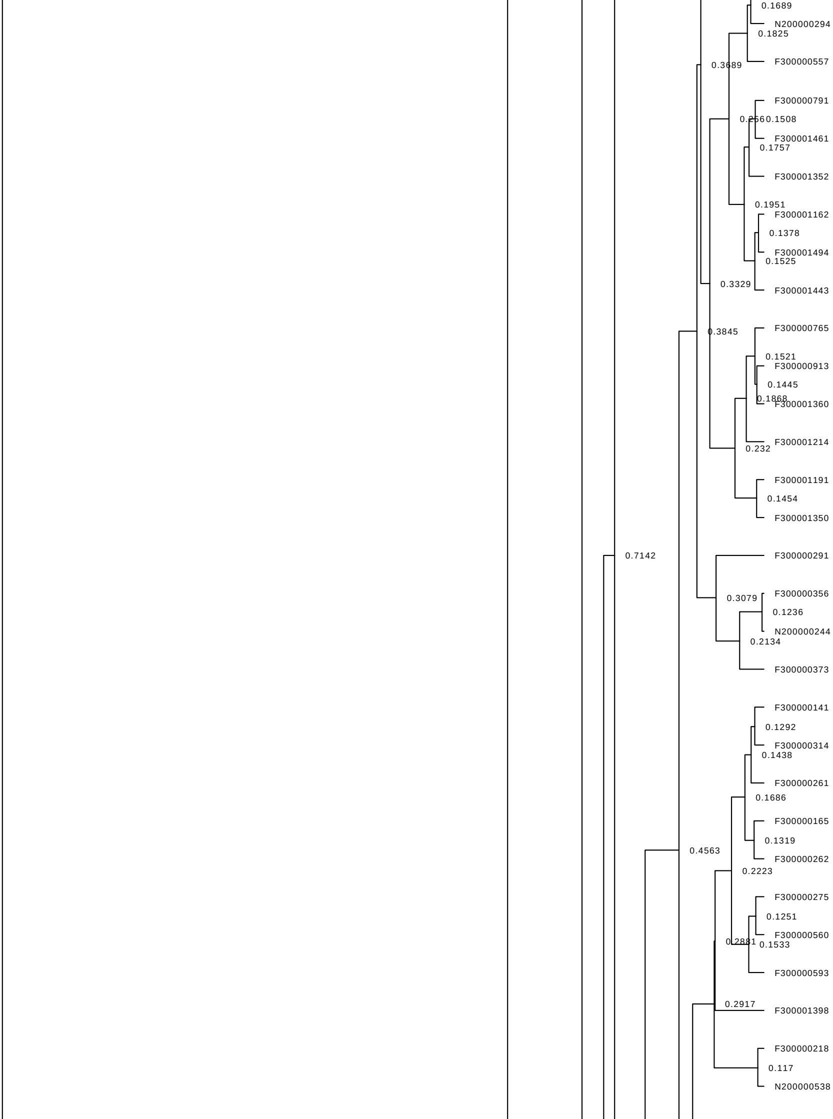
0.1689
N200000294
0.1825
F300000557
0.3689
F300000791
0.256
0.1508
F300001461
0.1757
F300001352
0.1951
F300001162
0.1378
F300001494
0.1525
0.3329
F300001443
F300000765
0.3845
0.1521
F300000913
0.1445
0.1868
F300001360
F300001214
0.232
F300001191
0.1454
F300001350
0.7142
F300000291
F300000356
0.3079
0.1236
N200000244
0.2134
F300000373
F300000141
0.1292
F300000314
0.1438
F300000261
0.1686
F300000165
0.1319
0.4563
F300000262
0.2223
F300000275
0.1251
F300000560
0.2881
0.1533
F300000593
0.2917
F300001398
F300000218
0.117
N200000538

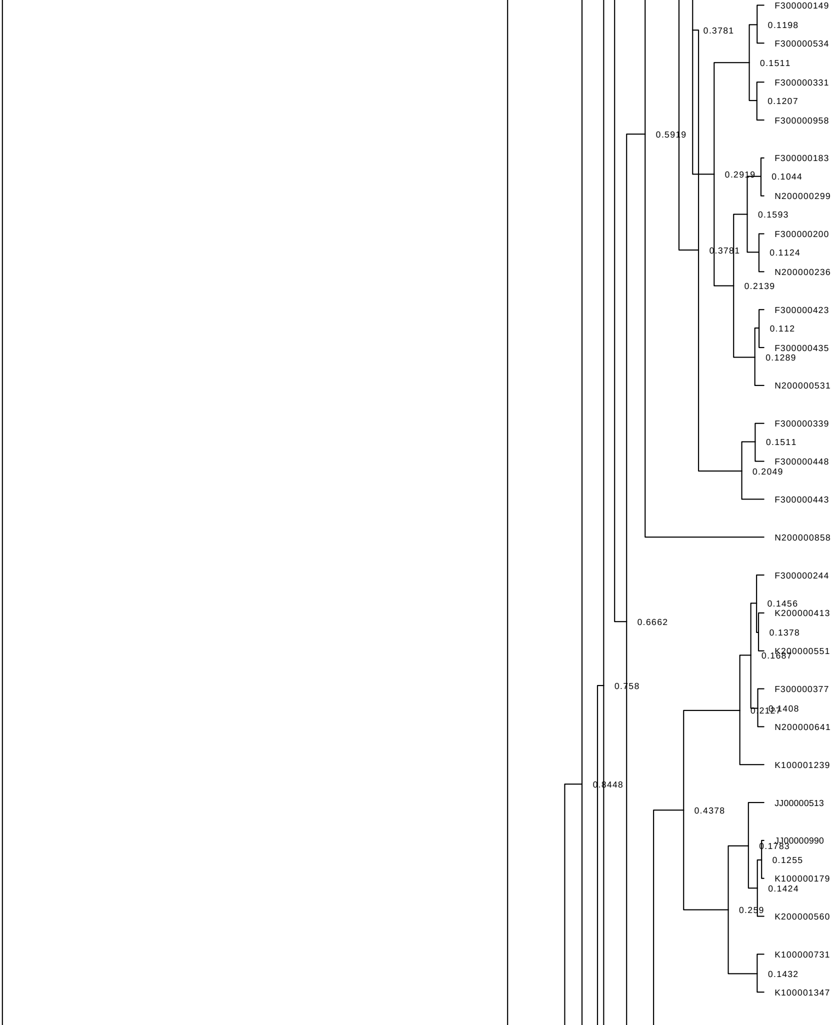
F300000149
0.3781
0.1198
F300000534
0.1511
F300000331
0.1207
F300000958
0.5919
F300000183
0.2919
0.1044
N200000299
0.1593
F300000200
0.3781
0.1124
N200000236
0.2139
F300000423
0.112
F300000435
0.1289
N200000531
F300000339
0.1511
F300000448
0.2049
F300000443
N200000858
F300000244
0.1456
K200000413
0.6662
0.1378
K200000551
0.1687
0.758
F300000377
0.2127
0.1408
N200000641
K100001239
0.8448
JJ00000513
0.4378
JJ00000990
0.1783
0.1255
K100000179
0.1424
0.259
K200000560
K100000731
0.1432
K100001347

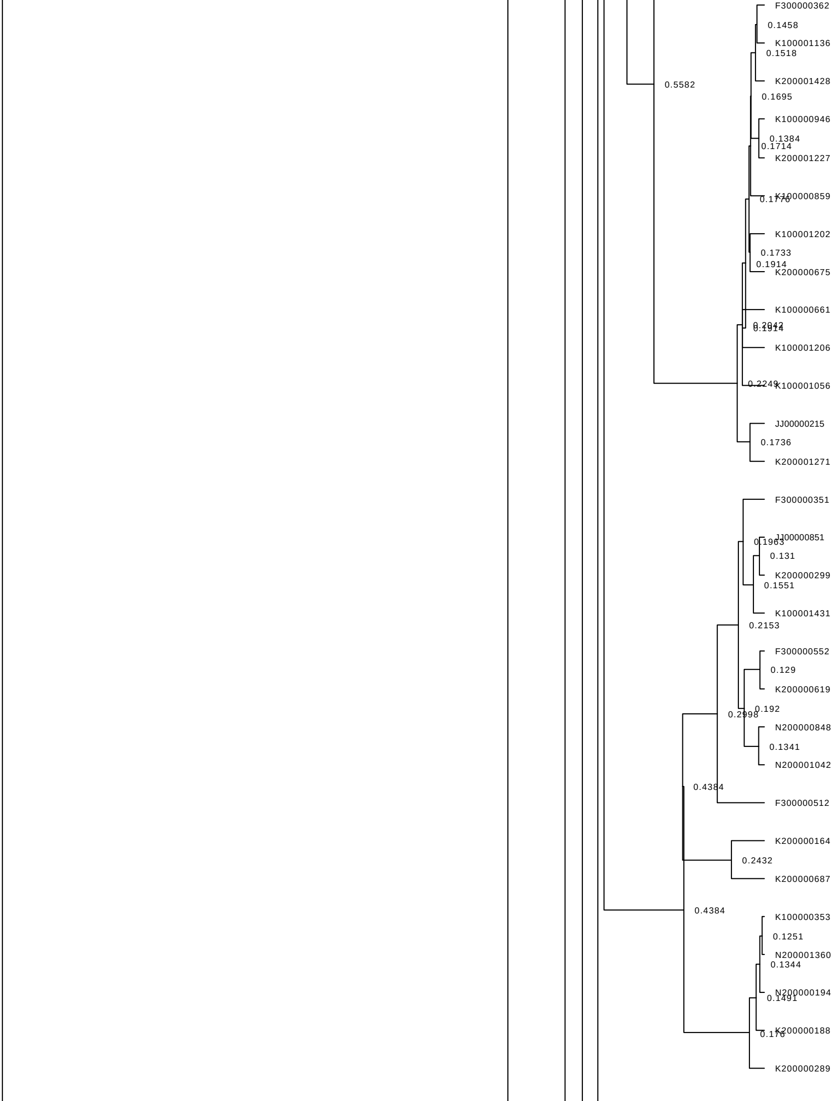
F300000362
0.1458
K100001136
0.1518
K200001428
0.5582
0.1695
K100000946
0.1384
0.1714
K200001227
K100000859
0.1776
K100001202
0.1733
0.1914
K200000675
K100000661
0.2042
0.1914
K100001206
0.2249
K100001056
JJ00000215
0.1736
K200001271
F300000351
JJ00000851
0.1963
0.131
K200000299
0.1551
K100001431
0.2153
F300000552
0.129
K200000619
0.192
0.2998
N200000848
0.1341
N200001042
0.4384
F300000512
K200000164
0.2432
K200000687
0.4384
K100000353
0.1251
N200001360
0.1344
N200000194
0.1491
K200000188
0.176
K200000289

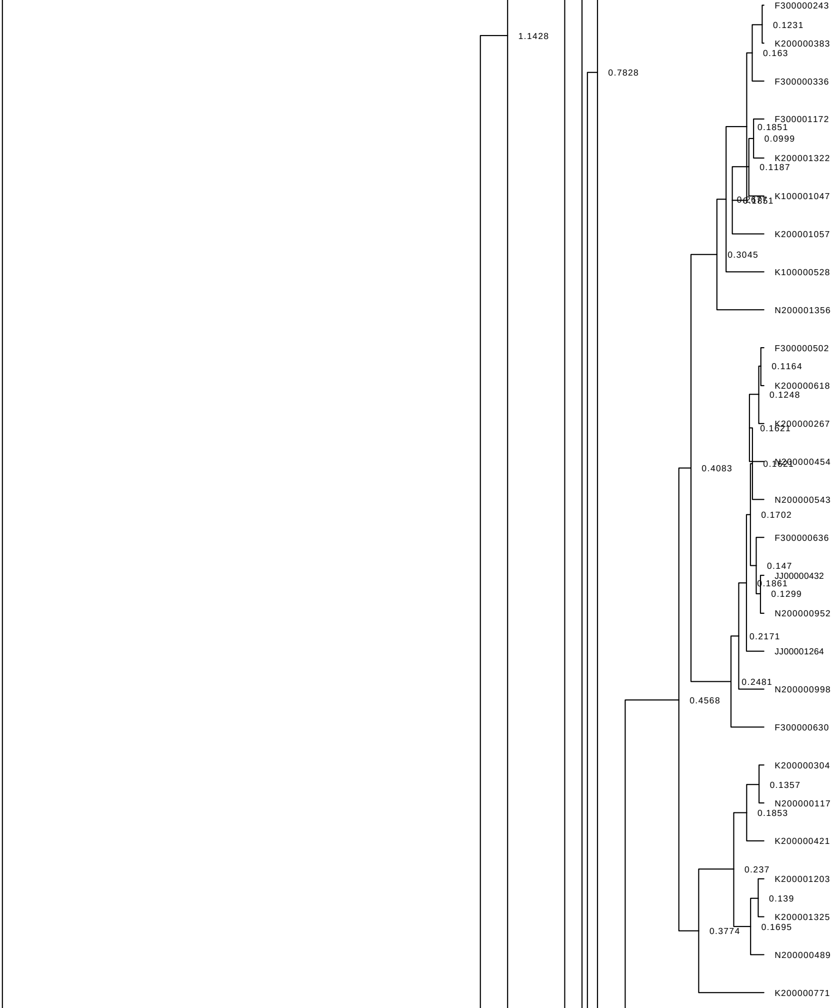

1.1428
0.7828
F300000243
0.1231
K200000383
0.163
F300000336
F300001172
0.1851
0.0999
K200001322
0.1187
K100001047
K200001057
0.3045
K100000528
N200001356
F300000502
0.1164
K200000618
0.1248
K200000267
0.1621
N200000454
0.1621
N200000543
0.1702
F300000636
0.147
JJ00000432
0.1861
0.1299
N200000952
0.2171
JJ00001264
0.2481
N200000998
0.4083
0.4568
F300000630
K200000304
0.1357
N200000117
0.1853
K200000421
0.237
K200001203
0.139
K200001325
0.1695
N200000489
0.3774
K200000771

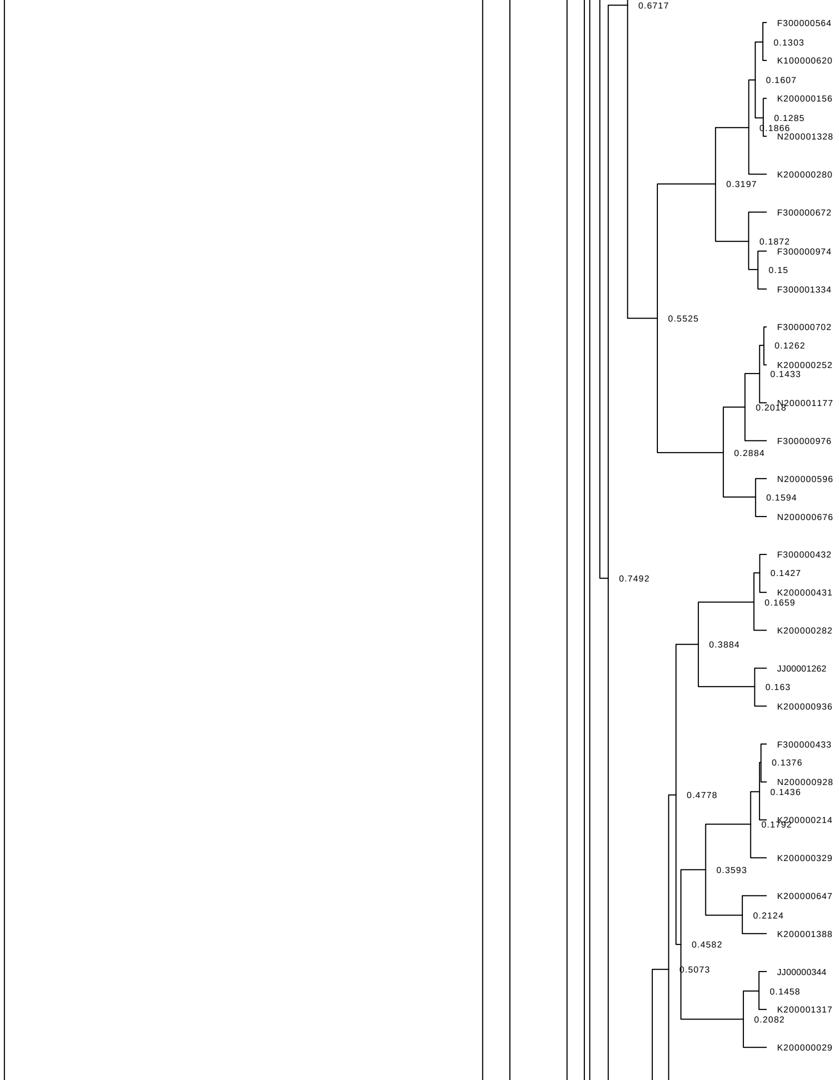
0.6717
F300000564
0.1303
K100000620
0.1607
K200000156
0.1285
0.1866
N200001328
K200000280
0.3197
F300000672
0.1872
F300000974
0.15
F300001334
0.5525
F300000702
0.1262
K200000252
0.1433
N200001177
0.2018
F300000976
0.2884
N200000596
0.1594
N200000676
F300000432
0.1427
0.7492
K200000431
0.1659
K200000282
0.3884
JJ00001262
0.163
K200000936
F300000433
0.1376
N200000928
0.4778
0.1436
K200000214
0.1792
K200000329
0.3593
K200000647
0.2124
K200001388
0.4582
0.5073
JJ00000344
0.1458
K200001317
0.2082
K200000029

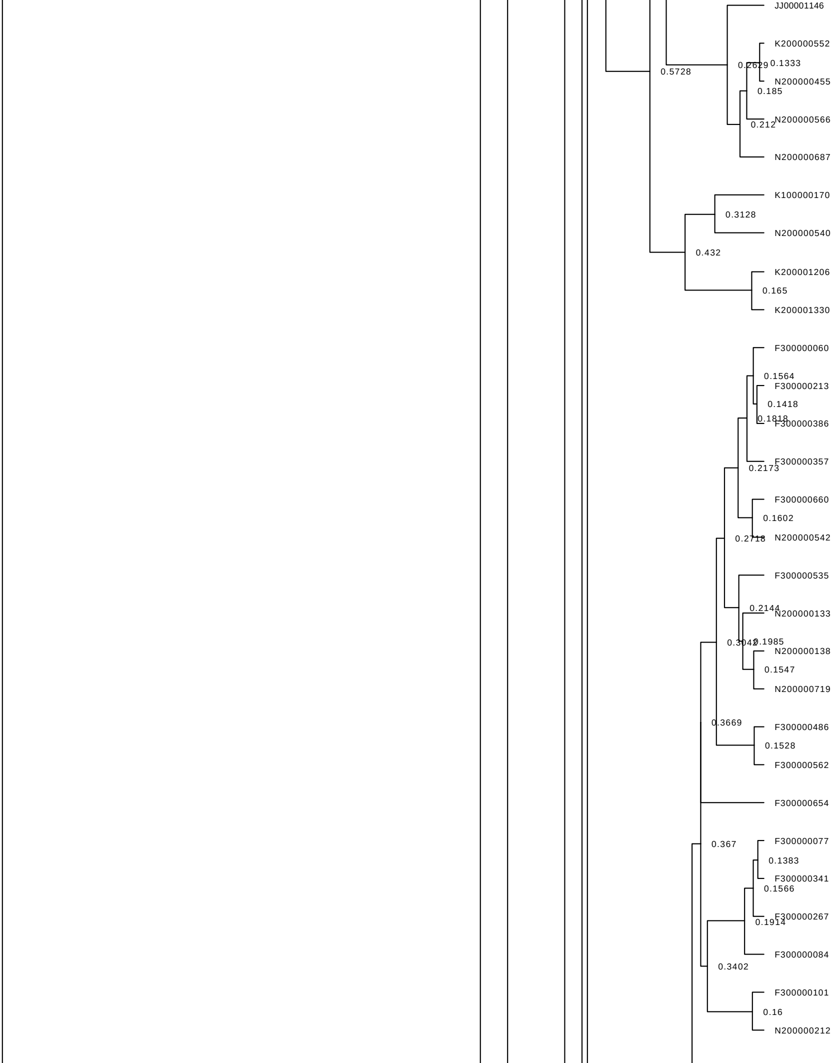
JJ00001146
K200000552
0.2629
0.1333
0.5728
N200000455
0.185
N200000566
0.212
N200000687
K100000170
0.3128
N200000540
0.432
K200001206
0.165
K200001330
F300000060
0.1564
F300000213
0.1418
0.1818
F300000386
F300000357
0.2173
F300000660
0.1602
0.2718
N200000542
F300000535
0.2144
N200000133
0.3042
0.1985
N200000138
0.1547
N200000719
0.3669
F300000486
0.1528
F300000562
F300000654
0.367
F300000077
0.1383
F300000341
0.1566
F300000267
0.1914
F300000084
0.3402
F300000101
0.16
N200000212

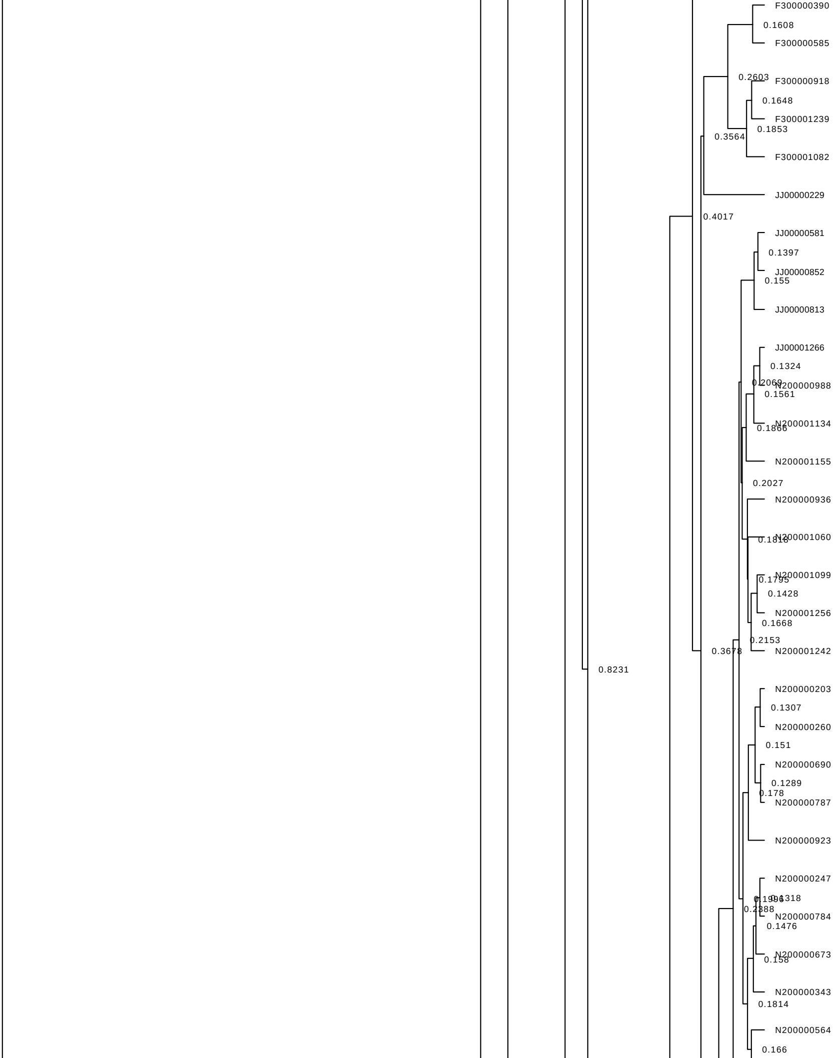
F300000390
0.1608
F300000585
0.2603
F300000918
0.1648
F300001239
0.1853
0.3564
F300001082
JJ00000229
0.4017
JJ00000581
0.1397
JJ00000852
0.155
JJ00000813
JJ00001266
0.1324
0.2069
N200000988
0.1561
N200001134
0.1866
N200001155
0.2027
N200000936
N200001060
0.1818
N200001099
0.1795
0.1428
N200001256
0.1668
0.2153
N200001242
0.3678
0.8231
N200000203
0.1307
N200000260
0.151
N200000690
0.1289
0.178
N200000787
N200000923
N200000247
0.1996
0.1318
0.2388
N200000784
0.1476
N200000673
0.158
N200000343
0.1814
N200000564
0.166

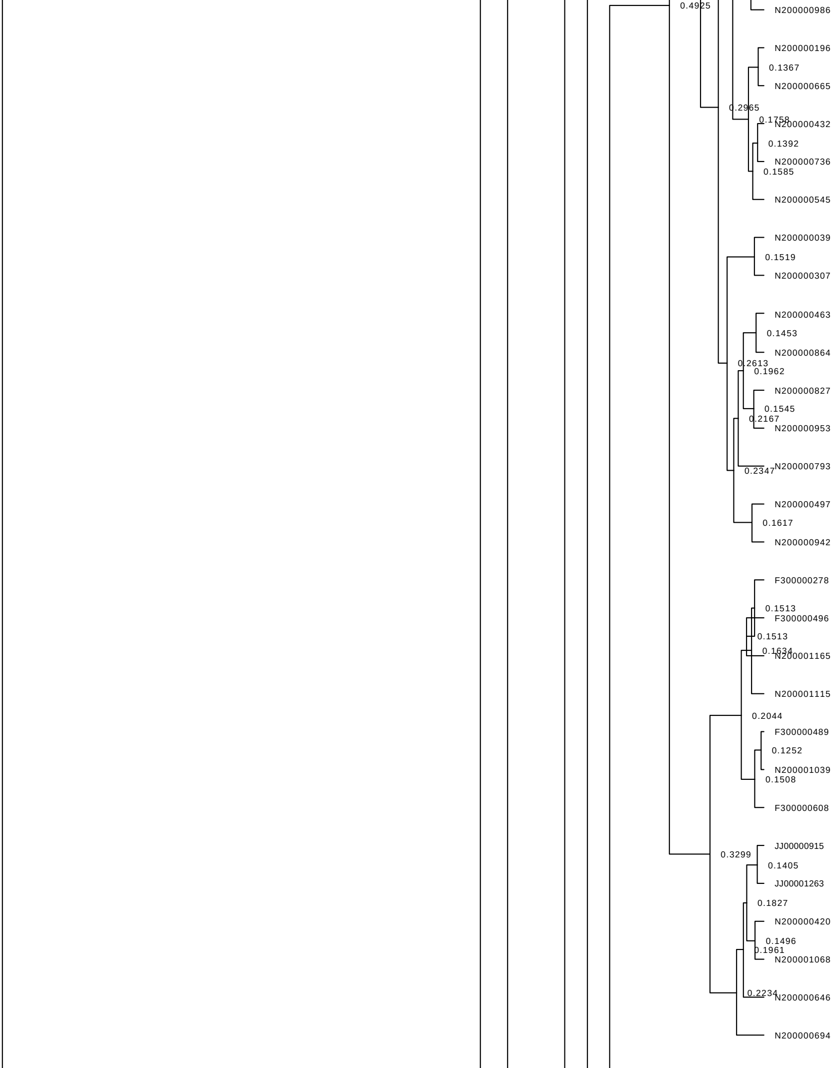
0.4925
N200000986
N200000196
0.1367
N200000665
0.2965
0.1758
N200000432
0.1392
N200000736
0.1585
N200000545
N200000039
0.1519
N200000307
N200000463
0.1453
N200000864
0.2613
0.1962
N200000827
0.1545
0.2167
N200000953
0.2347
N200000793
N200000497
0.1617
N200000942
F300000278
0.1513
F300000496
0.1513
0.1634
N200001165
N200001115
0.2044
F300000489
0.1252
N200001039
0.1508
F300000608
0.3299
JJ00000915
0.1405
JJ00001263
0.1827
N200000420
0.1496
0.1961
N200001068
0.2234
N200000646
N200000694

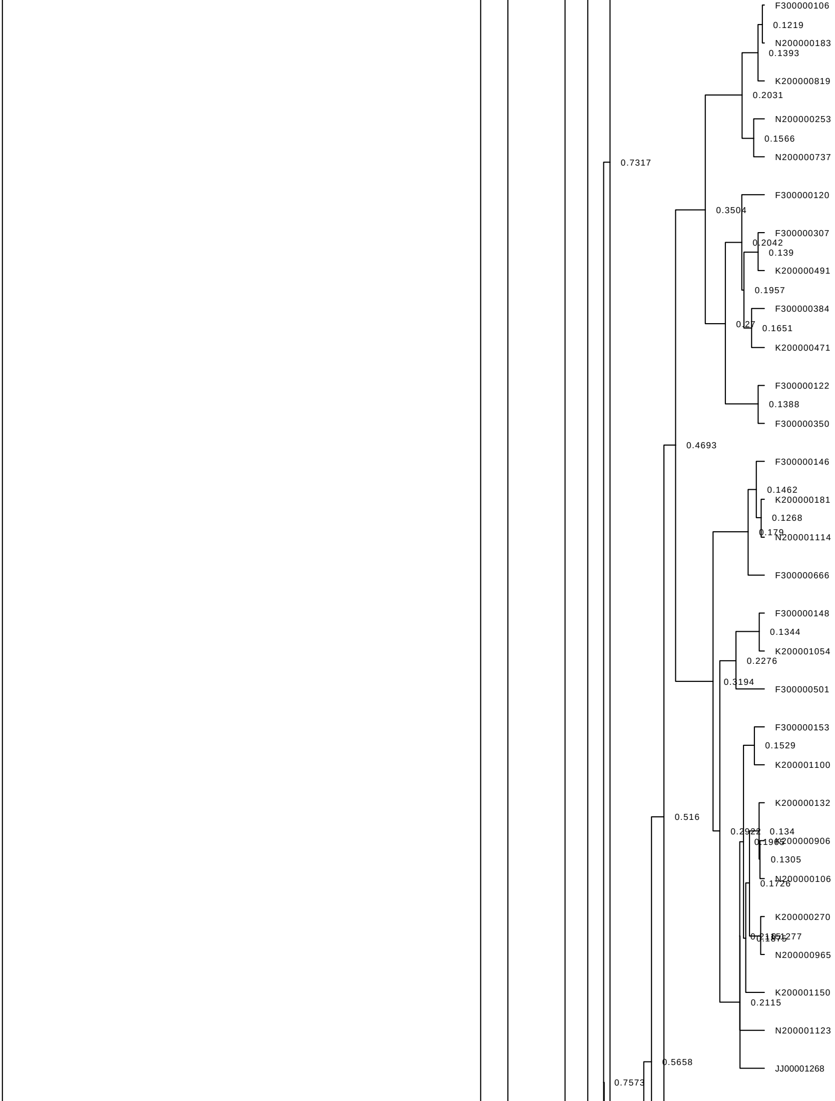
F300000106
0.1219
N200000183
0.1393
K200000819
0.2031
N200000253
0.1566
N200000737
0.7317
F300000120
0.3504
F300000307
0.2042
0.139
K200000491
0.1957
F300000384
0.27
0.1651
K200000471
F300000122
0.1388
F300000350
0.4693
F300000146
0.1462
K200000181
0.1268
0.179
N200001114
F300000666
F300000148
0.1344
K200001054
0.2276
0.3194
F300000501
F300000153
0.1529
K200001100
K200000132
0.516
0.2922
0.134
0.1963
K200000906
0.1305
N200000106
0.1726
K200000270
N200000965
K200001150
0.2115
N200001123
0.5658
JJ00001268
0.7573

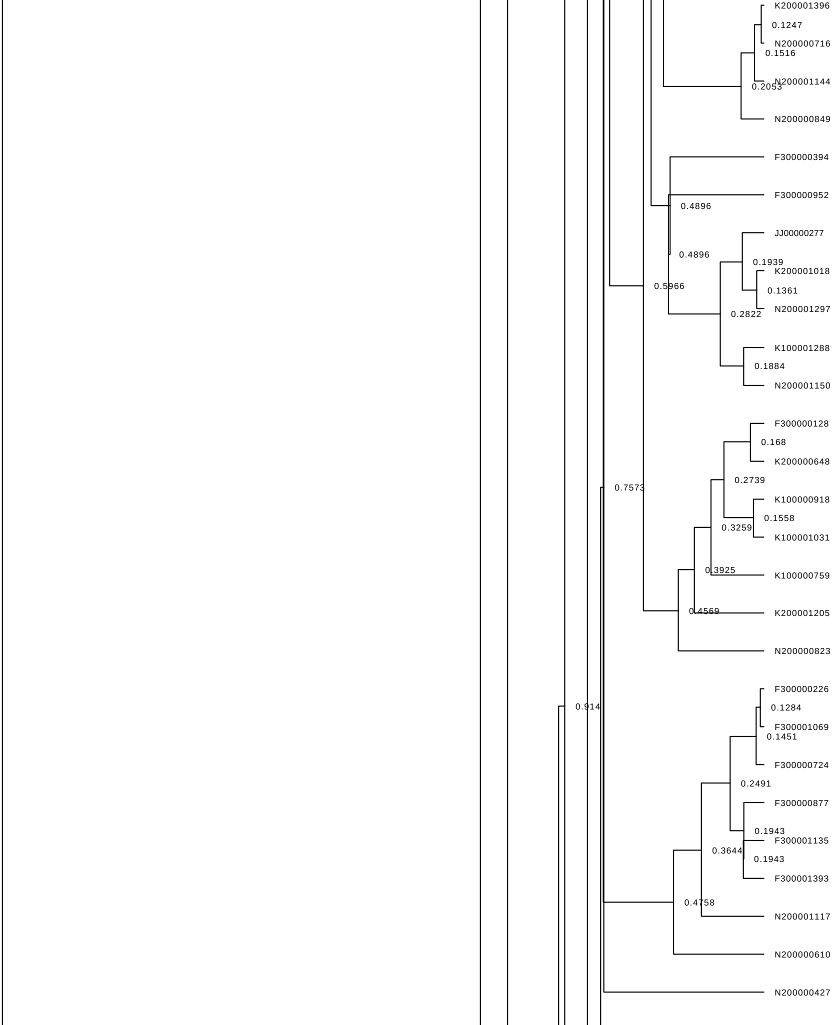
K200001396
0.1247
N200000716
0.1516
N200001144
0.2053
N200000849
F300000394
F300000952
0.4896
JJ00000277
0.4896
0.1939
K200001018
0.5966
0.1361
N200001297
0.2822
K100001288
0.1884
N200001150
F300000128
0.168
K200000648
0.2739
0.7573
K100000918
0.1558
0.3259
K100001031
0.3925
K100000759
0.4569
K200001205
N200000823
F300000226
0.914
0.1284
F300001069
0.1451
F300000724
0.2491
F300000877
0.1943
F300001135
0.3644
0.1943
F300001393
0.4758
N200001117
N200000610
N200000427

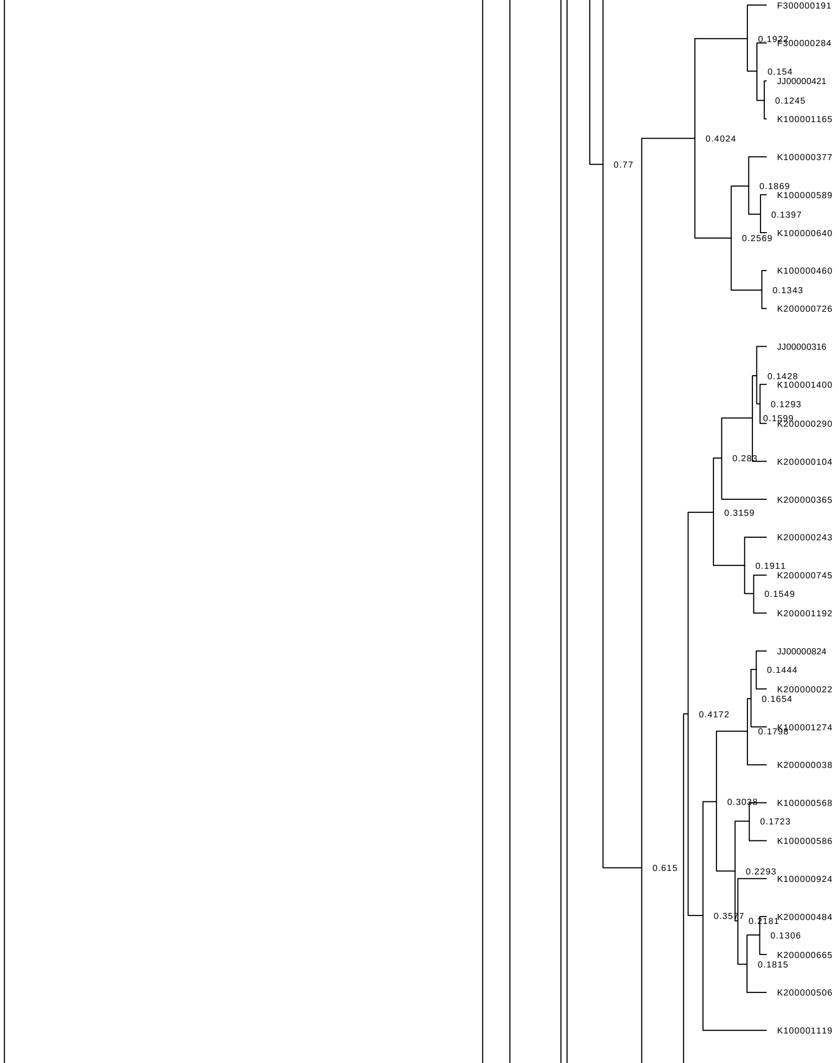
F300000191
0.1922
F300000284
0.154
JJ00000421
0.1245
K100001165
0.4024
0.77
K100000377
0.1869
K100000589
0.1397
K100000640
0.2569
K100000460
0.1343
K200000726
JJ00000316
0.1428
K100001400
0.1293
0.1599
K200000290
0.283
K200000104
K200000365
0.3159
K200000243
0.1911
K200000745
0.1549
K200001192
JJ00000824
0.1444
K200000022
0.1654
0.4172
K100001274
0.1798
K200000038
0.3038
K100000568
0.1723
K100000586
0.615
0.2293
K100000924
0.3577
0.2181
K200000484
0.1306
K200000665
0.1815
K200000506
K100001119

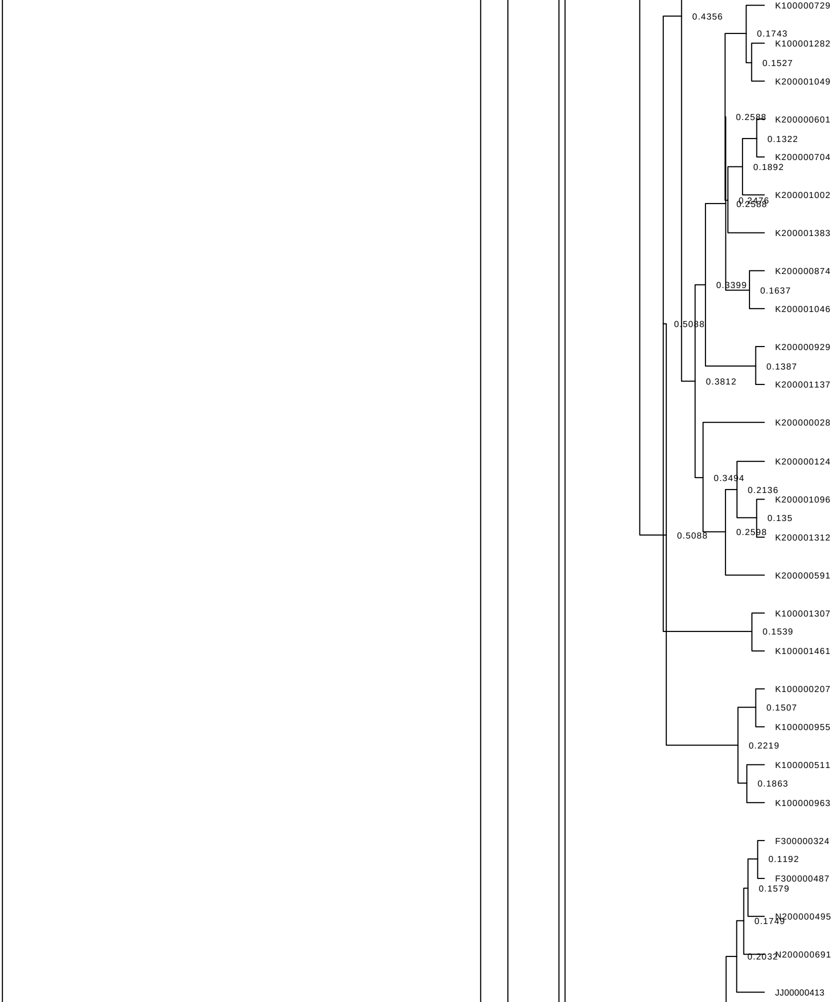

K100000729
0.4356
0.1743
K100001282
0.1527
K200001049
0.2588
K200000601
0.1322
K200000704
0.1892
K200001002
0.2476
0.2588
K200001383
K200000874
0.3399
0.1637
K200001046
0.5088
K200000929
0.1387
0.3812
K200001137
K200000028
K200000124
0.3494
0.2136
K200001096
0.135
0.2598
0.5088
K200001312
K200000591
K100001307
0.1539
K100001461
K100000207
0.1507
K100000955
0.2219
K100000511
0.1863
K100000963
F300000324
0.1192
F300000487
0.1579
N200000495
0.1749
N200000691
0.2032
JJ00000413

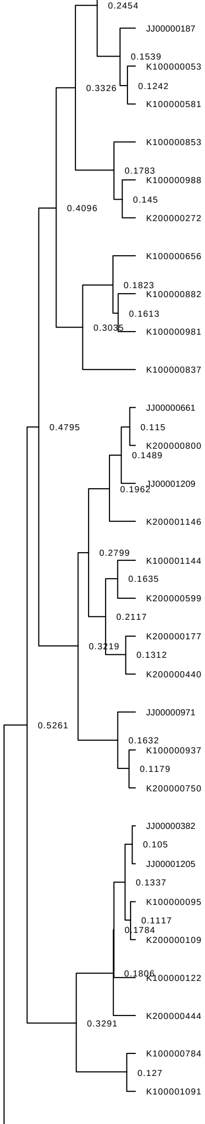
0.2454
JJ00000187
0.1539
K100000053
0.1242
0.3326
K100000581
K100000853
0.1783
K100000988
0.145
0.4096
K200000272
K100000656
0.1823
K100000882
0.1613
0.3035
K100000981
K100000837
JJ00000661
0.4795
0.115
K200000800
0.1489
JJ00001209
0.1962
K200001146
0.2799
K100001144
0.1635
K200000599
0.2117
K200000177
0.3219
0.1312
K200000440
JJ00000971
0.5261
0.1632
K100000937
0.1179
K200000750
JJ00000382
0.105
JJ00001205
0.1337
K100000095
0.1117
0.1784
K200000109
0.1806
K100000122
K200000444
0.3291
K100000784
0.127
K100001091

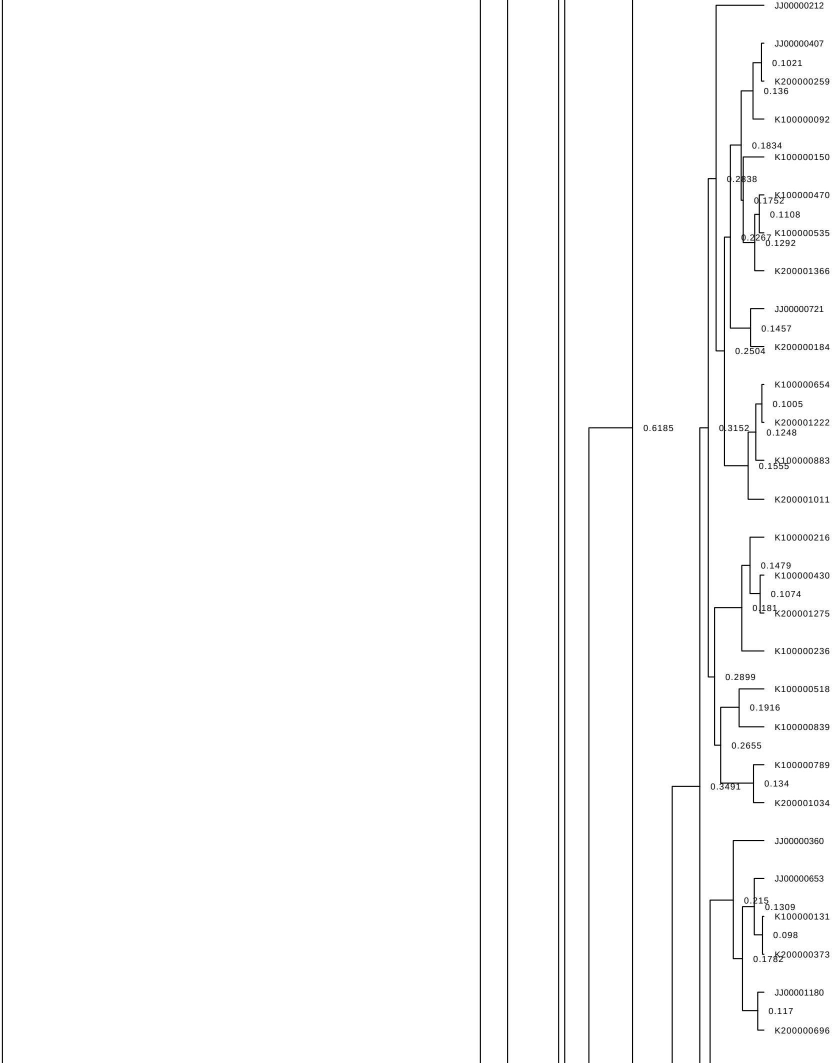

JJ00000212
JJ00000407
0.1021
K200000259
0.136
K100000092
0.1834
K100000150
0.2838
K100000470
0.1752
0.1108
K100000535
0.2267
0.1292
K200001366
JJ00000721
0.1457
K200000184
0.2504
K100000654
0.1005
K200001222
0.6185
0.3152
0.1248
K100000883
0.1555
K200001011
K100000216
0.1479
K100000430
0.1074
0.181
K200001275
K100000236
0.2899
K100000518
0.1916
K100000839
0.2655
K100000789
0.134
0.3491
K200001034
JJ00000360
JJ00000653
0.215
0.1309
K100000131
0.098
K200000373
0.1782
JJ00001180
0.117
K200000696

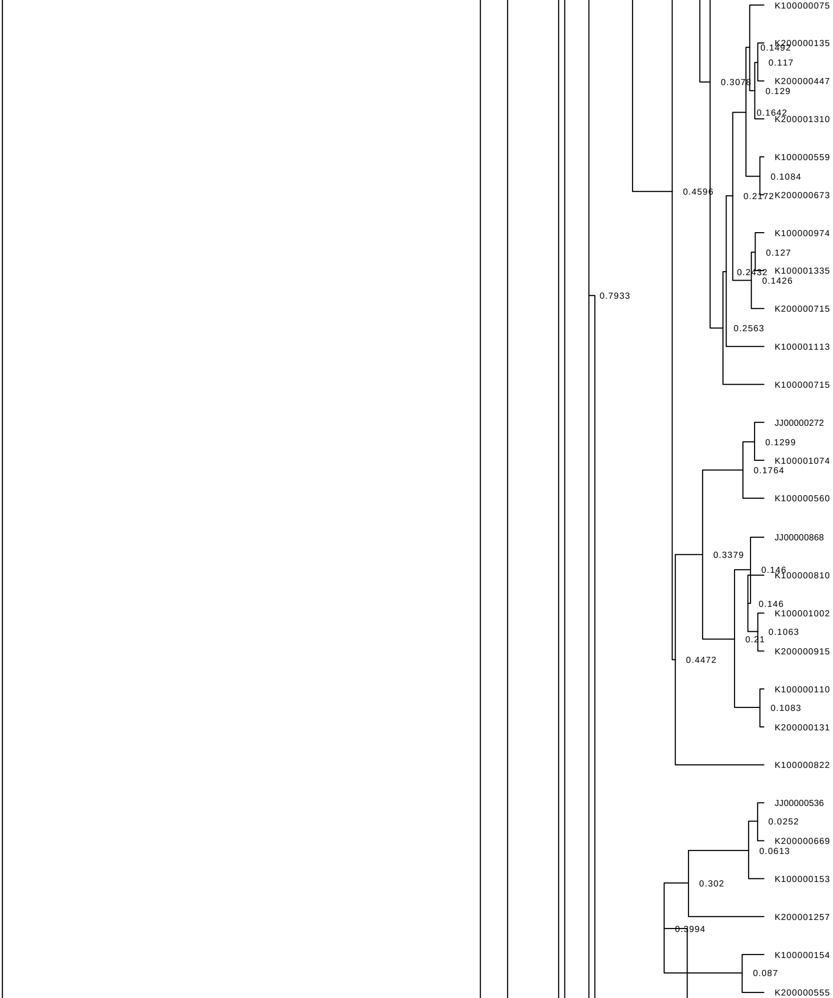
K100000075
K200000135
0.1492
0.117
0.3078
K200000447
0.129
0.1642
K200001310
K100000559
0.1084
0.4596
0.2172
K200000673
K100000974
0.127
0.2432
K100001335
0.1426
0.7933
K200000715
0.2563
K100001113
K100000715
JJ00000272
0.1299
K100001074
0.1764
K100000560
JJ00000868
0.3379
0.146
K100000810
0.146
K100001002
0.1063
0.21
K200000915
0.4472
K100000110
0.1083
K200000131
K100000822
JJ00000536
0.0252
K200000669
0.0613
K100000153
0.302
K200001257
0.3994
K100000154
0.087
K200000555

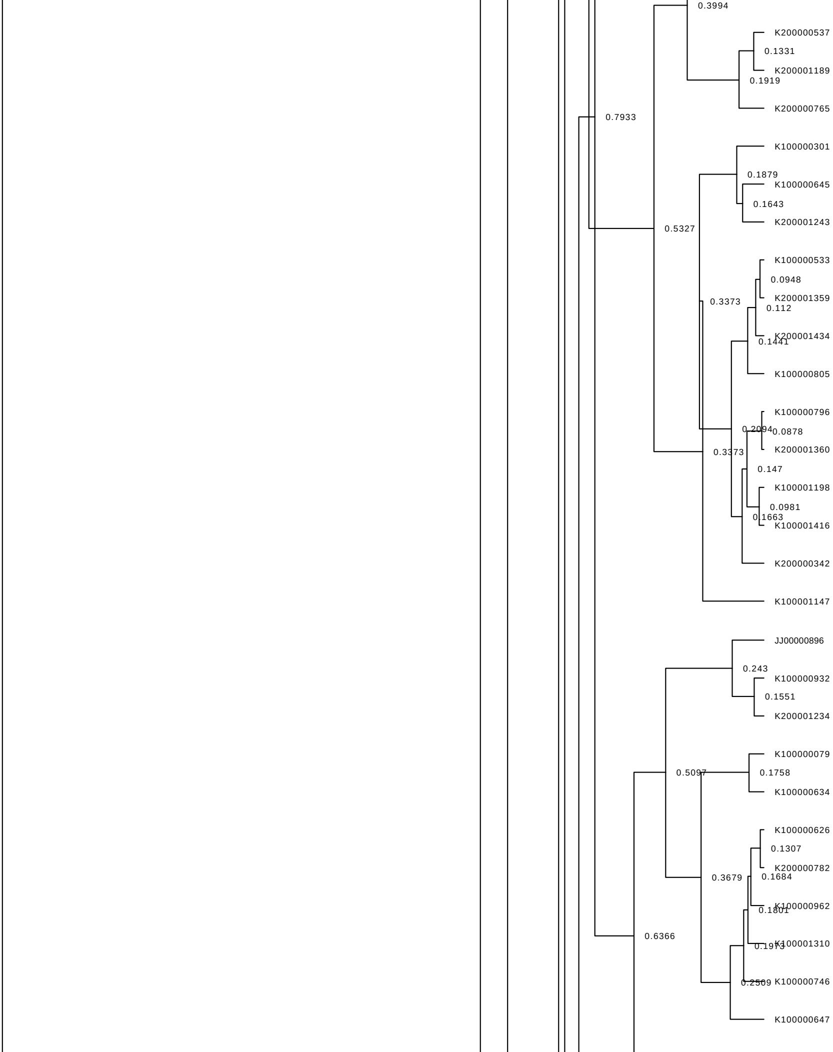
0.3994
K200000537
0.1331
K200001189
0.1919
K200000765
0.7933
K100000301
0.1879
K100000645
0.1643
K200001243
0.5327
K100000533
0.0948
K200001359
0.3373
0.112
K200001434
0.1441
K100000805
K100000796
0.2094
0.0878
K200001360
0.3373
0.147
K100001198
0.0981
0.1663
K100001416
K200000342
K100001147
JJ00000896
0.243
K100000932
0.1551
K200001234
K100000079
0.5097
0.1758
K100000634
K100000626
0.1307
K200000782
0.3679
0.1684
K100000962
0.1801
0.6366
K100001310
0.1973
0.2509
K100000746
K100000647

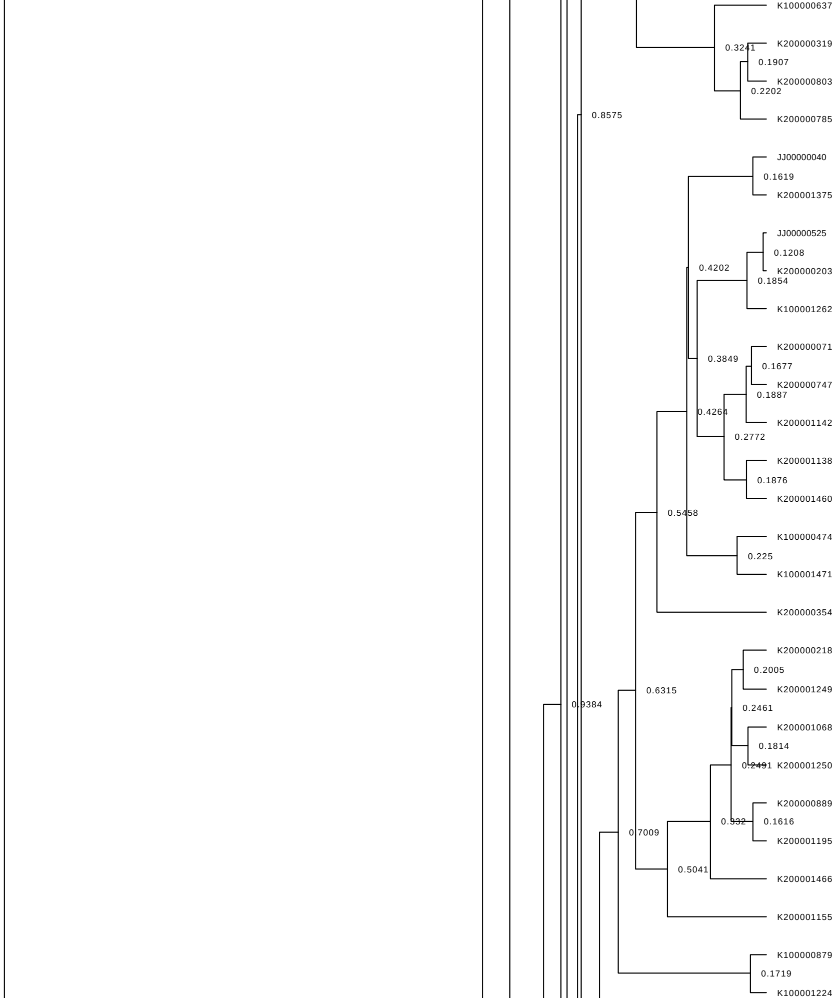

K100000637
0.3241
K200000319
0.1907
K200000803
0.2202
0.8575
K200000785
JJ00000040
0.1619
K200001375
JJ00000525
0.1208
0.4202
K200000203
0.1854
K100001262
K200000071
0.3849
0.1677
K200000747
0.1887
0.4264
K200001142
0.2772
K200001138
0.1876
K200001460
0.5458
K100000474
0.225
K100001471
K200000354
K200000218
0.2005
0.6315
K200001249
0.9384
0.2461
K200001068
0.1814
0.2491
K200001250
K200000889
0.332
0.1616
0.7009
K200001195
0.5041
K200001466
K200001155
K100000879
0.1719
K100001224

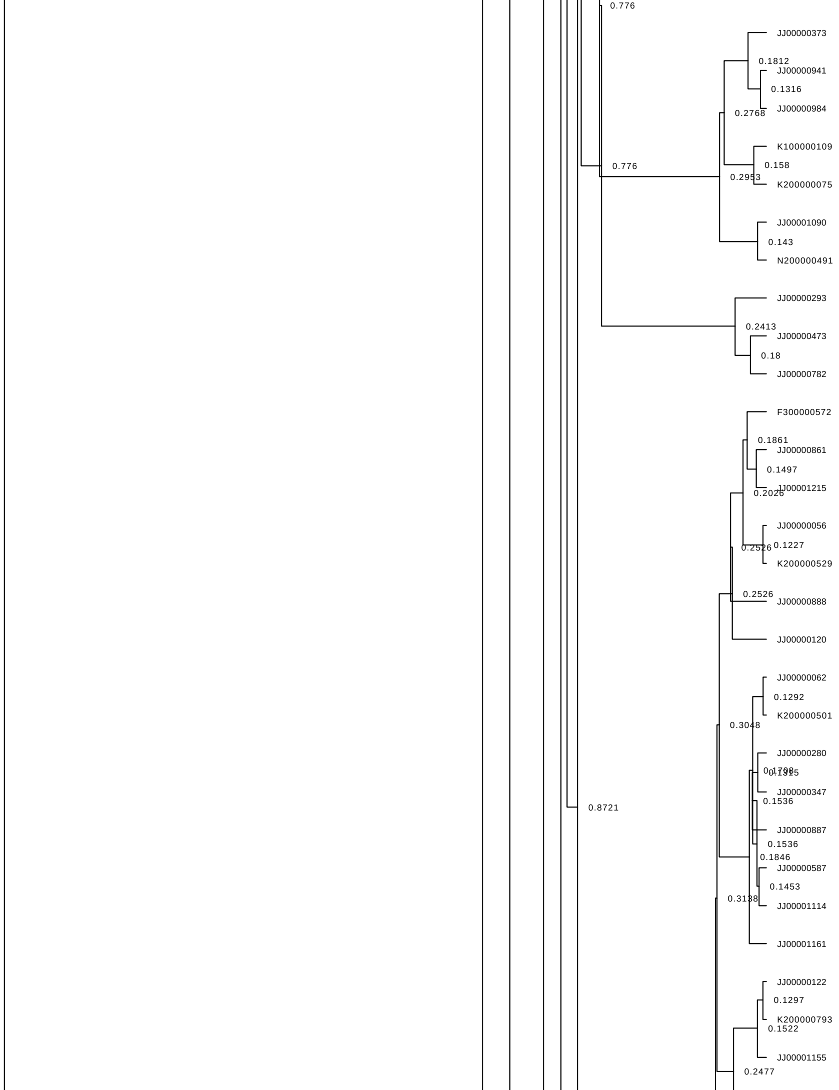
0.776
JJ00000373
0.1812
JJ00000941
0.1316
JJ00000984
0.2768
K100000109
0.776
0.158
0.2953
K200000075
JJ00001090
0.143
N200000491
JJ00000293
0.2413
JJ00000473
0.18
JJ00000782
F300000572
0.1861
JJ00000861
0.1497
JJ00001215
0.2026
JJ00000056
0.1227
0.2526
K200000529
0.2526
JJ00000888
JJ00000120
JJ00000062
0.1292
K200000501
0.3048
JJ00000280
0.1315
JJ00000347
0.1536
0.8721
JJ00000887
0.1536
0.1846
JJ00000587
0.1453
0.3138
JJ00001114
JJ00001161
JJ00000122
0.1297
K200000793
0.1522
JJ00001155
0.2477

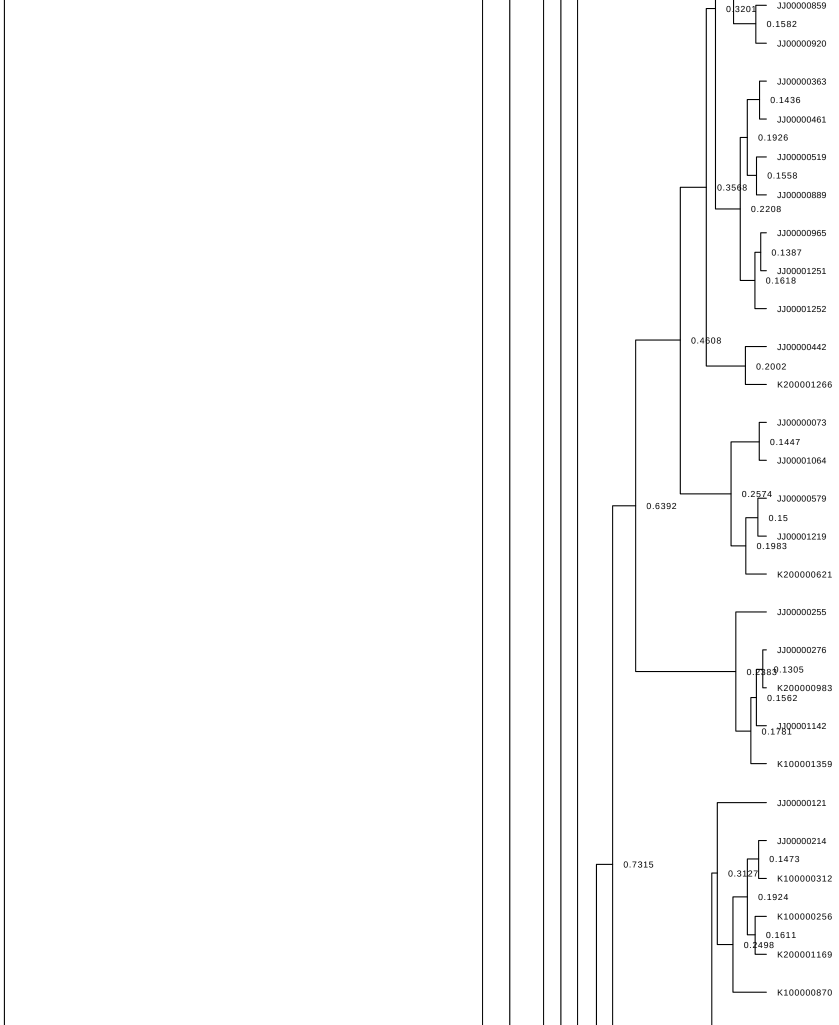
JJ00000859
0.3201
0.1582
JJ00000920
JJ00000363
0.1436
JJ00000461
0.1926
JJ00000519
0.1558
JJ00000889
0.3568
0.2208
JJ00000965
0.1387
JJ00001251
0.1618
JJ00001252
0.4608
JJ00000442
0.2002
K200001266
JJ00000073
0.1447
JJ00001064
0.2574
JJ00000579
0.6392
0.15
JJ00001219
0.1983
K200000621
JJ00000255
JJ00000276
0.2383
0.1305
K200000983
0.1562
JJ00001142
0.1781
K100001359
JJ00000121
JJ00000214
0.1473
0.7315
0.3127
K100000312
0.1924
K100000256
0.1611
0.2498
K200001169
K100000870

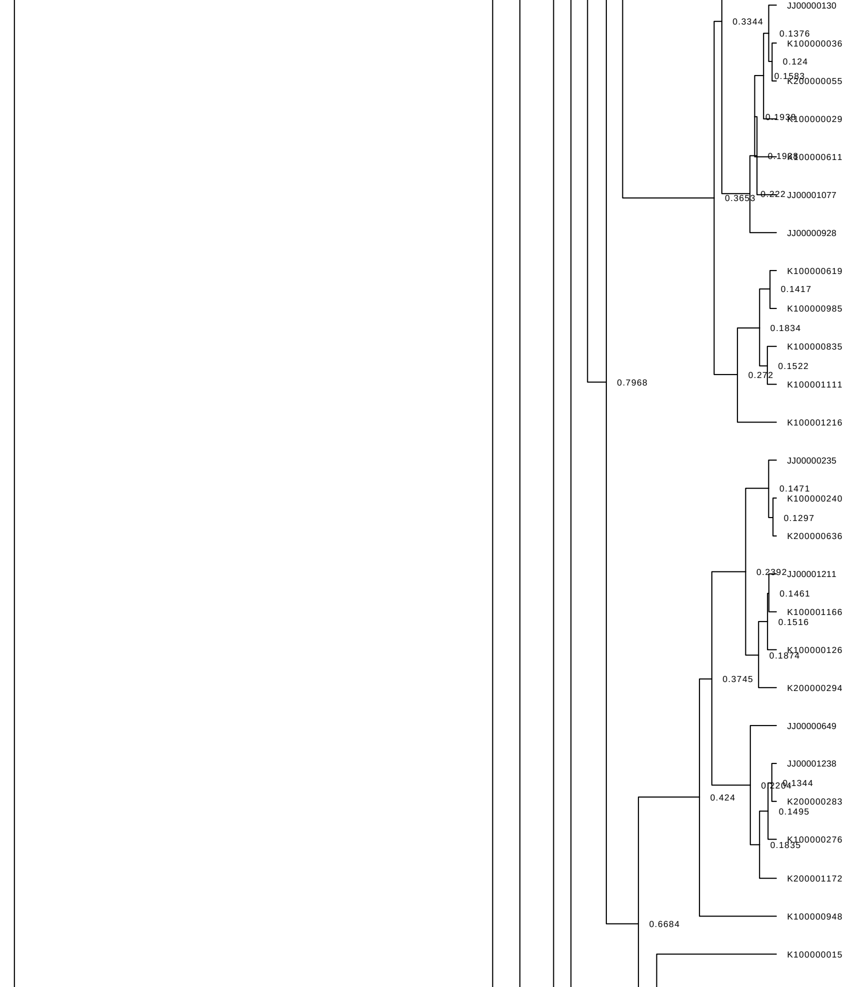
JJ00000130
0.3344
0.1376
K100000036
0.124
0.1583
K200000055
0.1938
K100000029
0.1968
K100000611
0.222
JJ00001077
0.3653
JJ00000928
K100000619
0.1417
K100000985
0.1834
K100000835
0.1522
0.272
K100001111
0.7968
K100001216
JJ00000235
0.1471
K100000240
0.1297
K200000636
0.2392
JJ00001211
0.1461
K100001166
0.1516
K100000126
0.1874
0.3745
K200000294
JJ00000649
JJ00001238
0.2204
0.1344
K200000283
0.1495
0.424
K100000276
0.1835
K200001172
K100000948
0.6684
K100000015

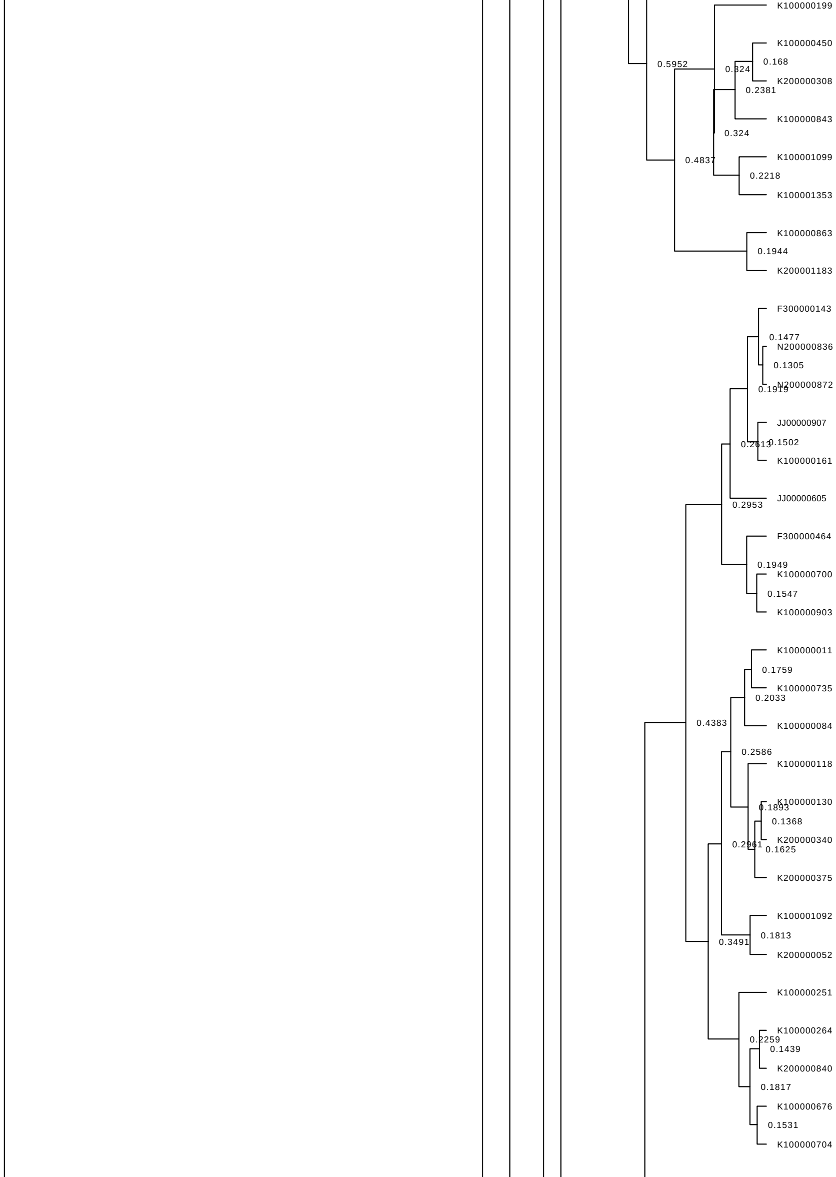
K100000199
K100000450
0.5952
0.168
0.324
K200000308
0.2381
K100000843
0.324
K100001099
0.4837
0.2218
K100001353
K100000863
0.1944
K200001183
F300000143
0.1477
N200000836
0.1305
N200000872
0.1919
JJ00000907
0.2613
0.1502
K100000161
JJ00000605
0.2953
F300000464
0.1949
K100000700
0.1547
K100000903
K100000011
0.1759
K100000735
0.2033
0.4383
K100000084
0.2586
K100000118
K100000130
0.1893
0.1368
K200000340
0.2961
0.1625
K200000375
K100001092
0.1813
0.3491
K200000052
K100000251
K100000264
0.2259
0.1439
K200000840
0.1817
K100000676
0.1531
K100000704

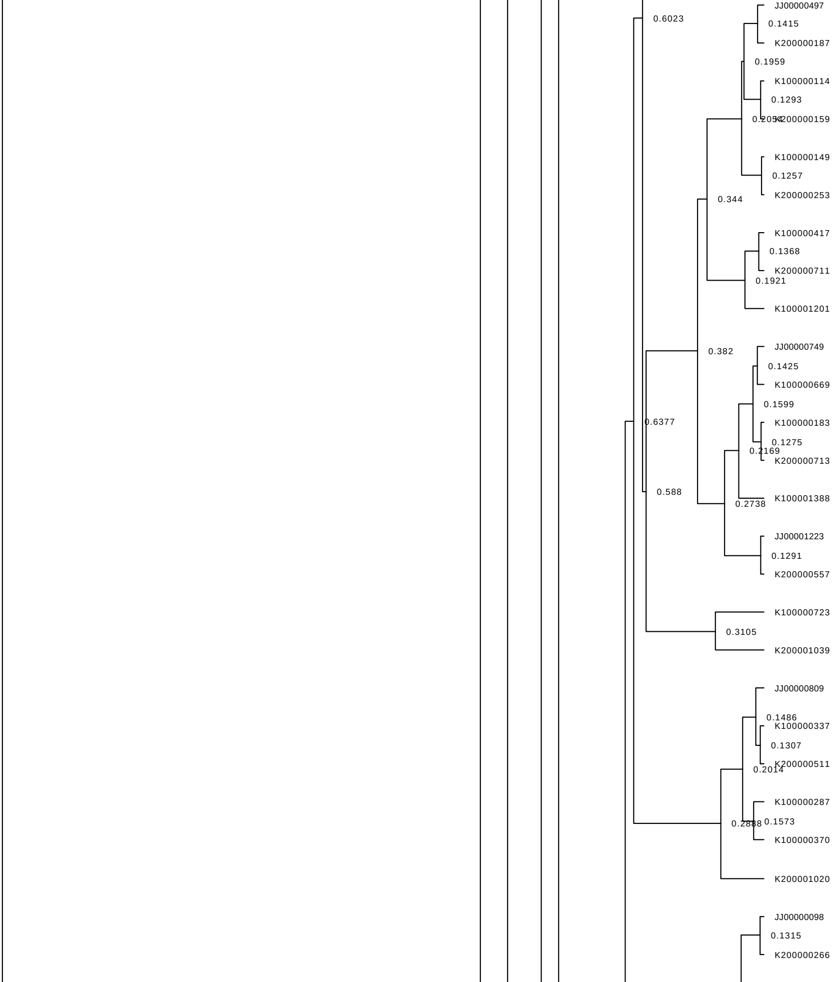
JJ00000497
0.6023
0.1415
K200000187
0.1959
K100000114
0.1293
0.2054
K200000159
K100000149
0.1257
K200000253
0.344
K100000417
0.1368
K200000711
0.1921
K100001201
JJ00000749
0.382
0.1425
K100000669
0.1599
K100000183
0.6377
0.1275
0.2169
K200000713
0.588
K100001388
0.2738
JJ00001223
0.1291
K200000557
K100000723
0.3105
K200001039
JJ00000809
0.1486
K100000337
0.1307
K200000511
0.2014
K100000287
0.2888
0.1573
K100000370
K200001020
JJ00000098
0.1315
K200000266

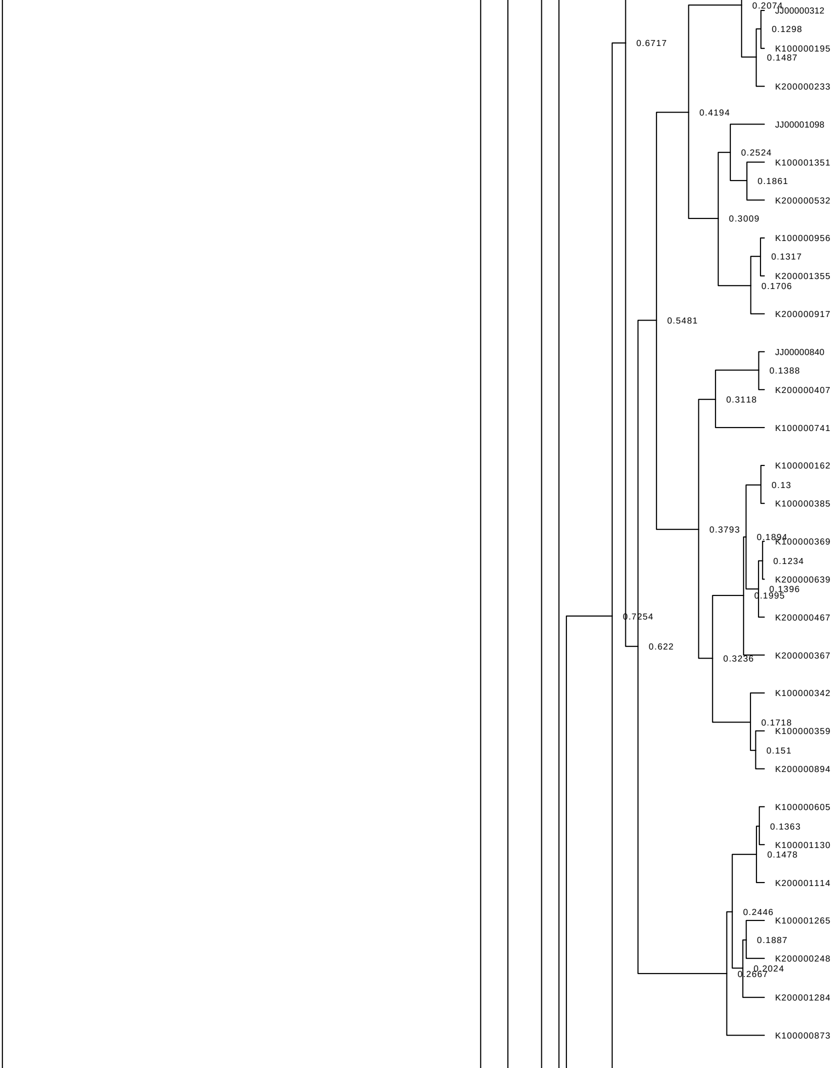

0.2074
JJ00000312
0.1298
K100000195
0.1487
K200000233
0.6717
0.4194
JJ00001098
0.2524
K100001351
0.1861
K200000532
0.3009
K100000956
0.1317
K200001355
0.1706
K200000917
0.5481
JJ00000840
0.1388
K200000407
0.3118
K100000741
K100000162
0.13
K100000385
0.3793
0.1894
K100000369
0.1234
K200000639
0.1396
0.1995
K200000467
0.7254
0.622
K200000367
0.3236
K100000342
0.1718
K100000359
0.151
K200000894
K100000605
0.1363
K100001130
0.1478
K200001114
0.2446
K100001265
0.1887
K200000248
0.2024
0.2667
K200001284
K100000873

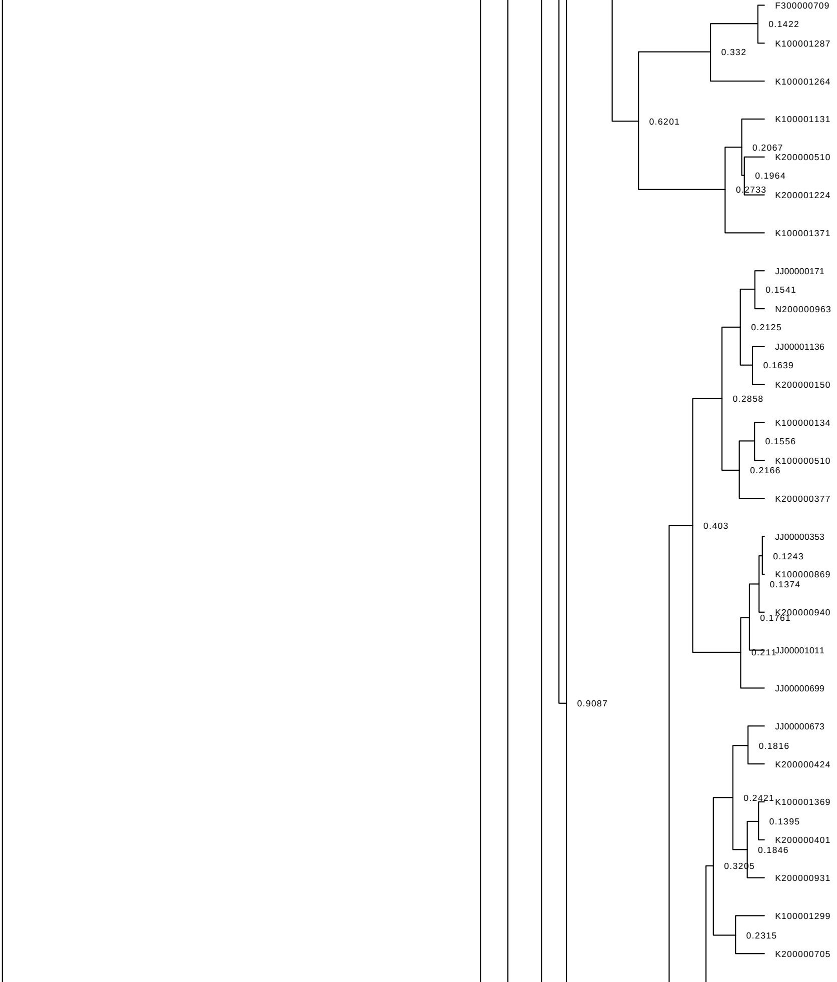
F300000709
0.1422
K100001287
0.332
K100001264
0.6201
K100001131
0.2067
K200000510
0.1964
0.2733
K200001224
K100001371
JJ00000171
0.1541
N200000963
0.2125
JJ00001136
0.1639
K200000150
0.2858
K100000134
0.1556
K100000510
0.2166
K200000377
0.403
JJ00000353
0.1243
K100000869
0.1374
K200000940
0.1761
0.211
JJ00001011
JJ00000699
0.9087
JJ00000673
0.1816
K200000424
0.2421
K100001369
0.1395
K200000401
0.1846
0.3205
K200000931
K100001299
0.2315
K200000705

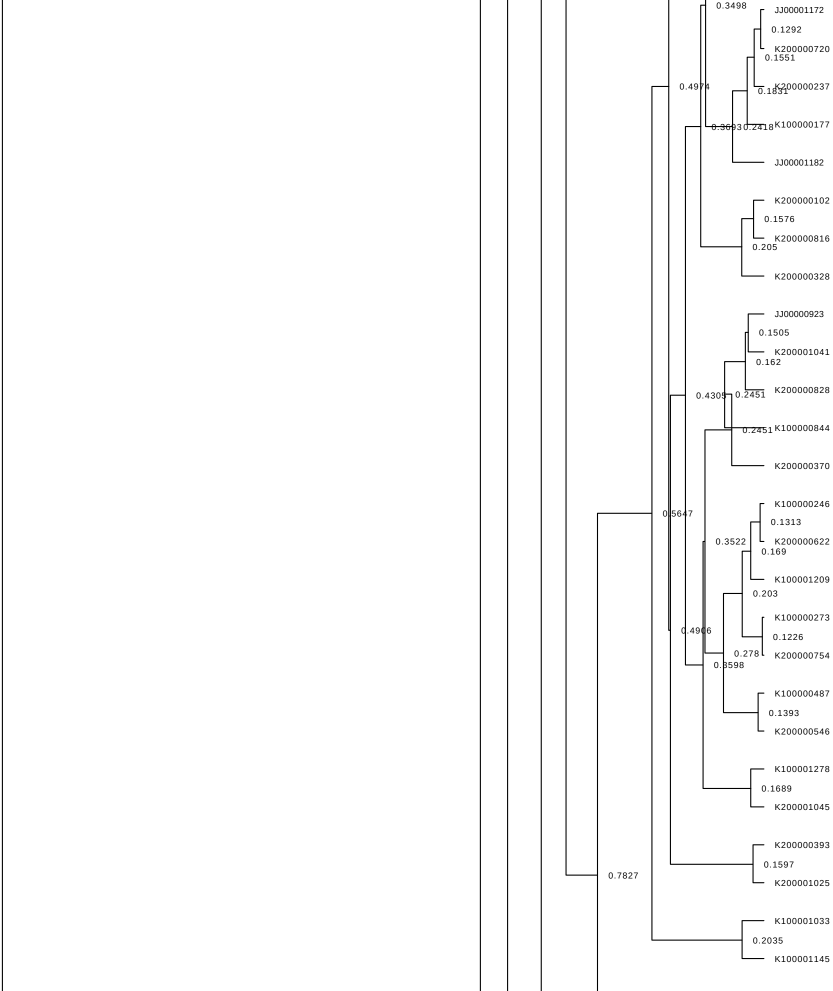

JJ00001172
0.3498
0.1292
K200000720
0.1551
0.4974
K200000237
0.1831
K100000177
0.3693
0.2418
JJ00001182
K200000102
0.1576
K200000816
0.205
K200000328
JJ00000923
0.1505
K200001041
0.162
K200000828
0.4305
0.2451
K100000844
0.2451
K200000370
K100000246
0.1313
0.5647
K200000622
0.3522
0.169
K100001209
0.203
K100000273
0.1226
0.4906
K200000754
0.278
0.3598
K100000487
0.1393
K200000546
K100001278
0.1689
K200001045
K200000393
0.1597
K200001025
0.7827
K100001033
0.2035
K100001145

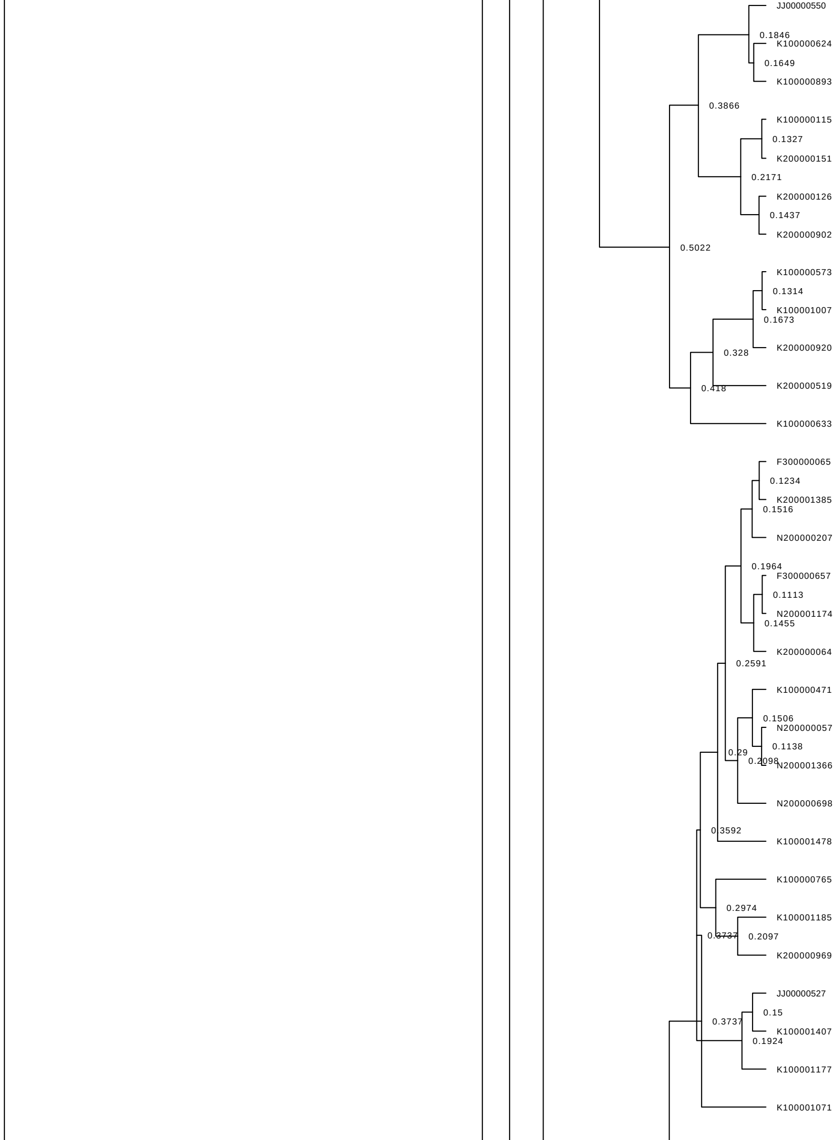

JJ00000550
0.1846
K100000624
0.1649
K100000893
0.3866
K100000115
0.1327
K200000151
0.2171
K200000126
0.1437
K200000902
0.5022
K100000573
0.1314
K100001007
0.1673
K200000920
0.328
K200000519
0.418
K100000633
F300000065
0.1234
K200001385
0.1516
N200000207
0.1964
F300000657
0.1113
N200001174
0.1455
K200000064
0.2591
K100000471
0.1506
N200000057
0.1138
0.29
0.2098
N200001366
N200000698
0.3592
K100001478
K100000765
0.2974
K100001185
0.3737
0.2097
K200000969
JJ00000527
0.15
0.3737
K100001407
0.1924
K100001177
K100001071

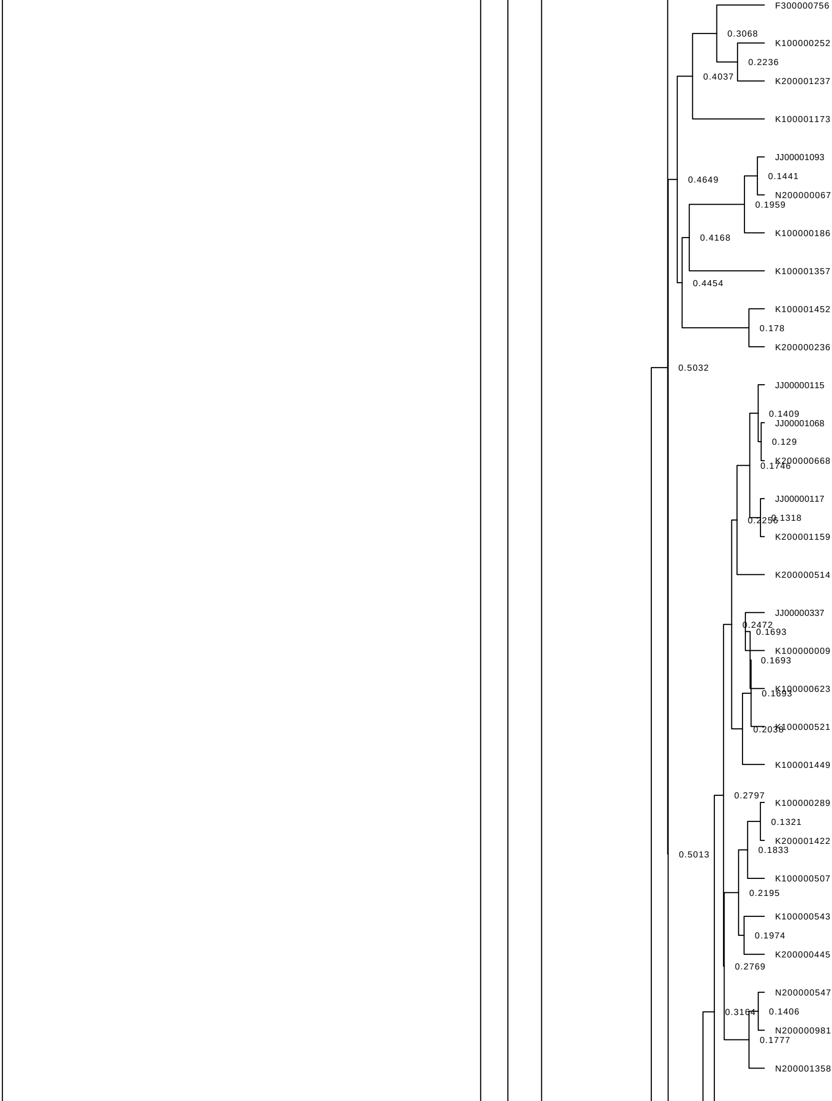
F300000756
0.3068
K100000252
0.2236
0.4037
K200001237
K100001173
JJ00001093
0.1441
0.4649
N200000067
0.1959
0.4168
K100000186
K100001357
0.4454
K100001452
0.178
K200000236
0.5032
JJ00000115
0.1409
JJ00001068
0.129
K200000668
0.1746
JJ00000117
0.1318
0.2256
K200001159
K200000514
JJ00000337
0.2472
0.1693
K100000009
0.1693
K100000623
0.1693
K100000521
0.2038
K100001449
0.2797
K100000289
0.1321
K200001422
0.1833
0.5013
K100000507
0.2195
K100000543
0.1974
K200000445
0.2769
N200000547
0.3164
0.1406
N200000981
0.1777
N200001358

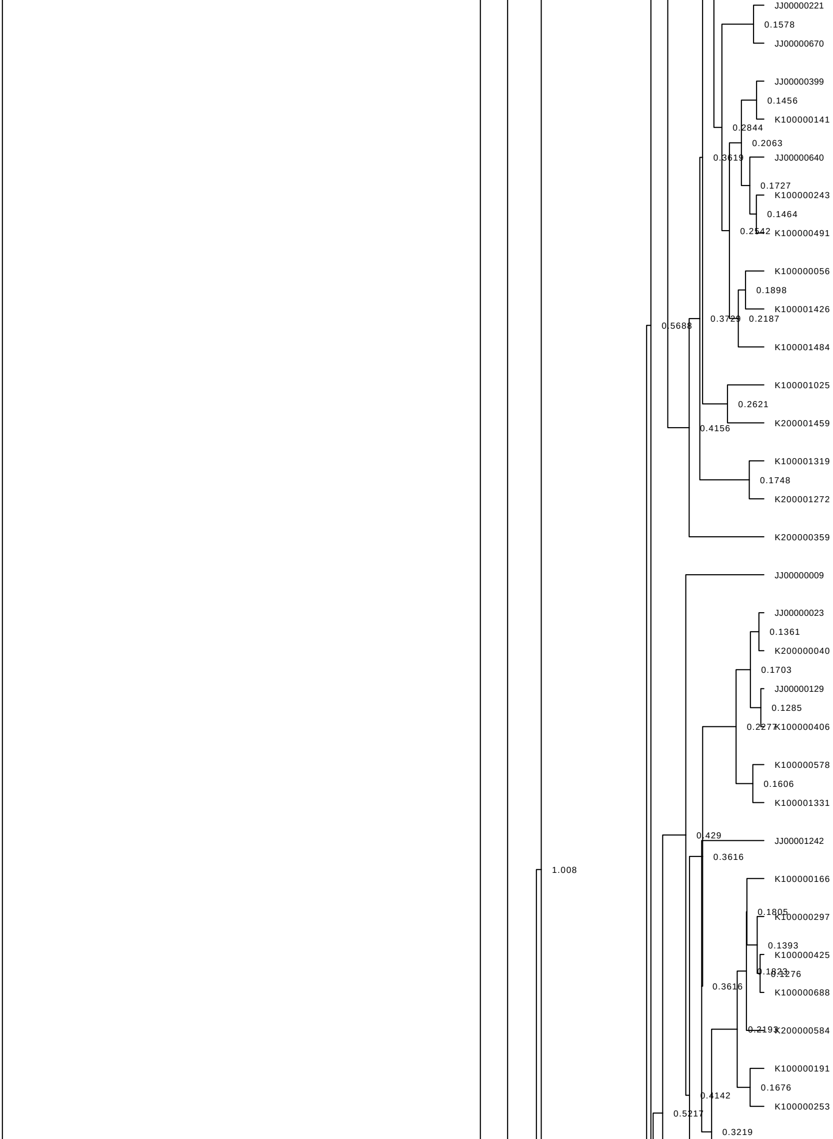
JJ00000221
0.1578
JJ00000670
JJ00000399
0.1456
K100000141
0.2844
0.2063
0.3619
JJ00000640
0.1727
K100000243
0.1464
0.2542
K100000491
K100000056
0.1898
K100001426
0.3729
0.2187
0.5688
K100001484
K100001025
0.2621
K200001459
0.4156
K100001319
0.1748
K200001272
K200000359
JJ00000009
JJ00000023
0.1361
K200000040
0.1703
JJ00000129
0.1285
0.2277
K100000406
K100000578
0.1606
K100001331
0.429
JJ00001242
0.3616
1.008
K100000166
0.1805
K100000297
0.1393
K100000425
0.1823
0.1276
0.3616
K100000688
0.2193
K200000584
K100000191
0.1676
0.4142
K100000253
0.5217
0.3219

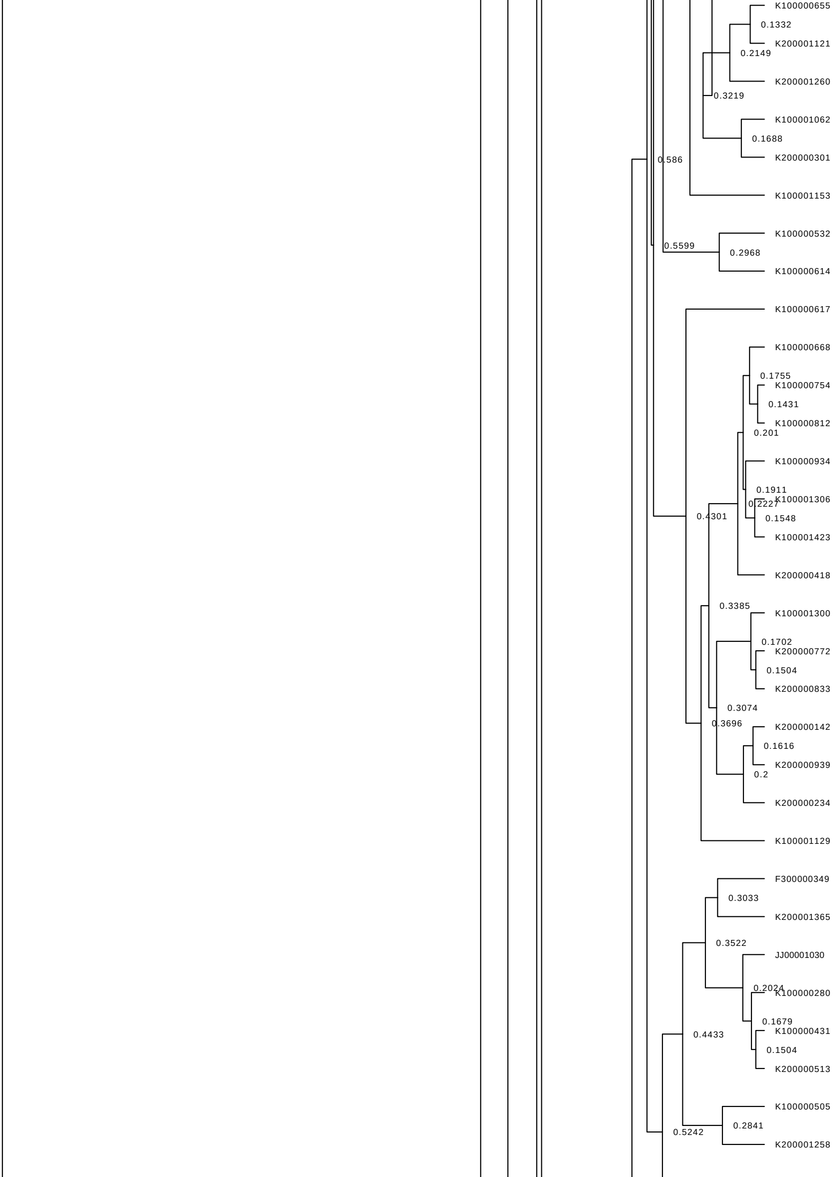
K100000655
0.1332
K200001121
0.2149
K200001260
0.3219
K100001062
0.1688
K200000301
0.586
K100001153
K100000532
0.5599
0.2968
K100000614
K100000617
K100000668
0.1755
K100000754
0.1431
K100000812
0.201
K100000934
0.1911
K100001306
0.2227
0.4301
0.1548
K100001423
K200000418
0.3385
K100001300
0.1702
K200000772
0.1504
K200000833
0.3074
0.3696
K200000142
0.1616
K200000939
0.2
K200000234
K100001129
F300000349
0.3033
K200001365
0.3522
JJ00001030
0.2024
K100000280
0.1679
K100000431
0.4433
0.1504
K200000513
K100000505
0.2841
0.5242
K200001258

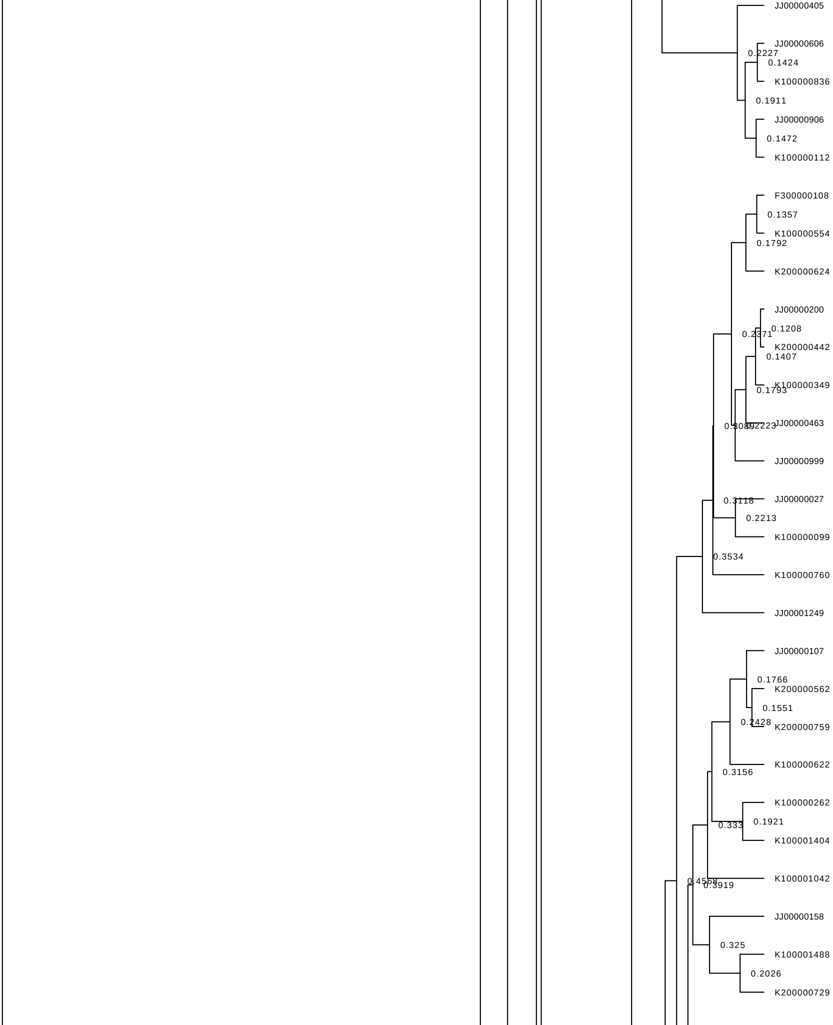
JJ00000405
JJ00000606
0.2227
0.1424
K100000836
0.1911
JJ00000906
0.1472
K100000112
F300000108
0.1357
K100000554
0.1792
K200000624
JJ00000200
0.1208
0.2371
K200000442
0.1407
K100000349
0.1793
JJ00000463
0.3089
0.2223
JJ00000999
JJ00000027
0.3118
0.2213
K100000099
0.3534
K100000760
JJ00001249
JJ00000107
0.1766
K200000562
0.1551
0.2428
K200000759
K100000622
0.3156
K100000262
0.1921
0.333
K100001404
K100001042
0.4568
0.3919
JJ00000158
0.325
K100001488
0.2026
K200000729

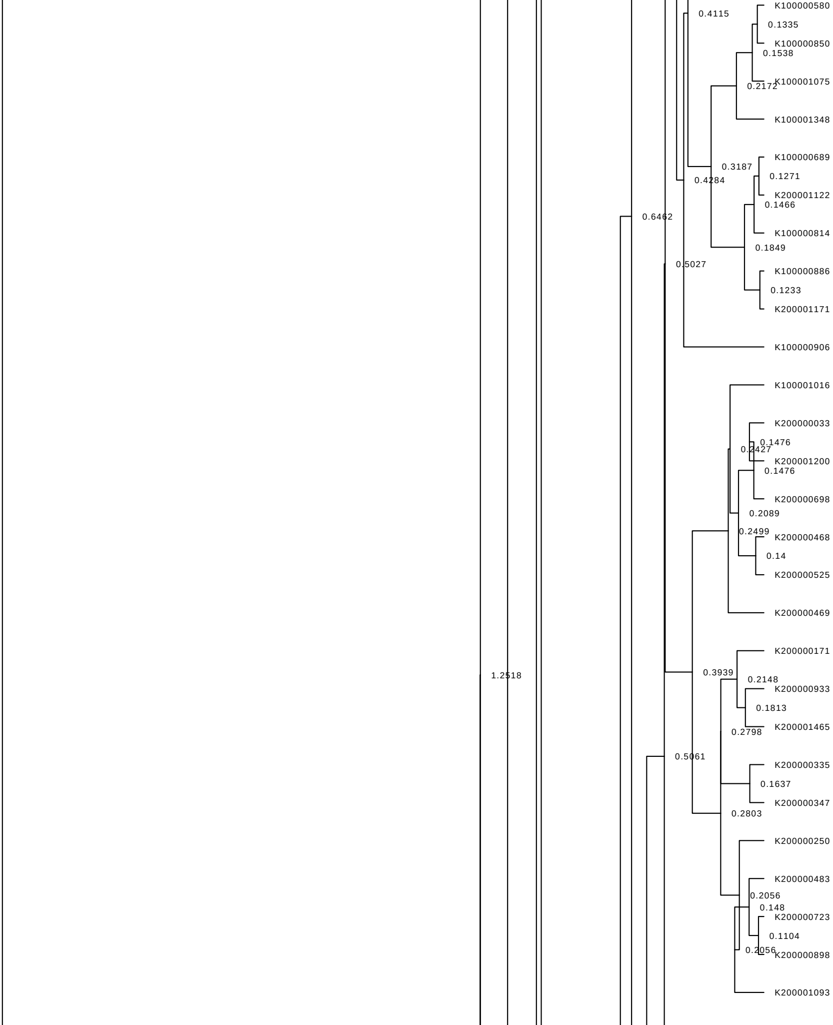
K100000580
0.4115
0.1335
K100000850
0.1538
K100001075
0.2172
K100001348
K100000689
0.3187
0.1271
0.4284
K200001122
0.1466
0.6462
K100000814
0.1849
0.5027
K100000886
0.1233
K200001171
K100000906
K100001016
K200000033
0.1476
0.2427
K200001200
0.1476
K200000698
0.2089
0.2499
K200000468
0.14
K200000525
K200000469
K200000171
0.3939
1.2518
0.2148
K200000933
0.1813
K200001465
0.2798
0.5061
K200000335
0.1637
K200000347
0.2803
K200000250
K200000483
0.2056
0.148
K200000723
0.1104
0.2056
K200000898
K200001093

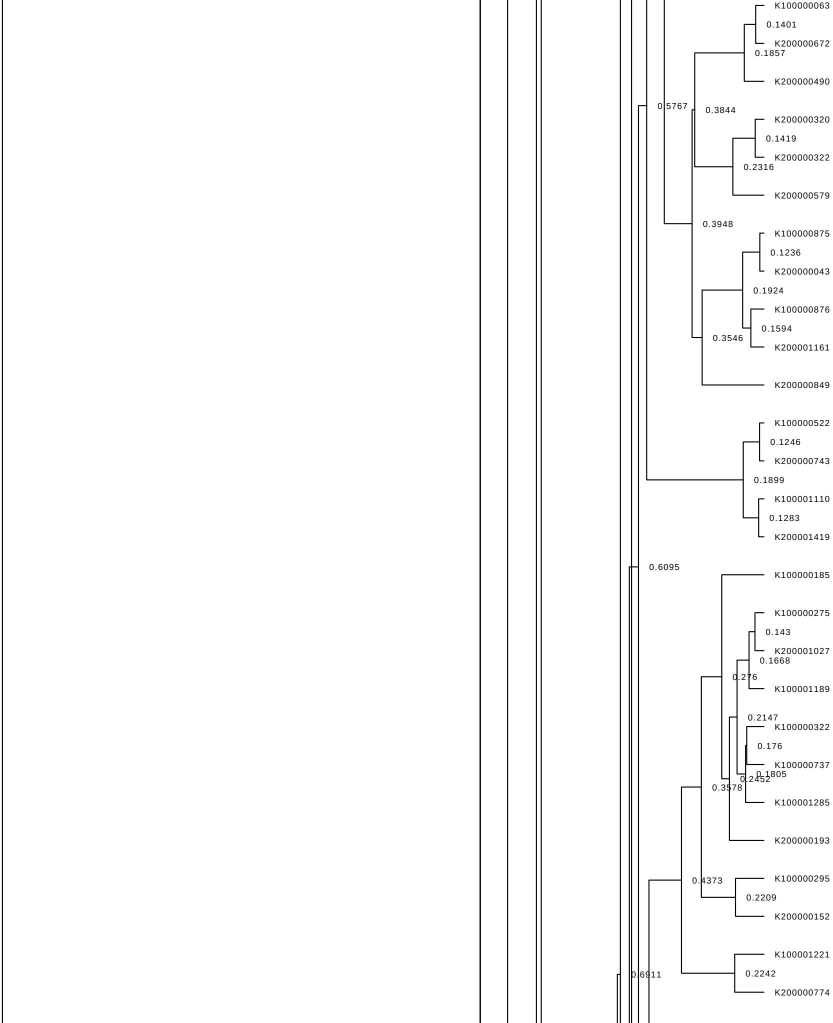
K100000063
0.1401
K200000672
0.1857
K200000490
0.5767
0.3844
K200000320
0.1419
K200000322
0.2316
K200000579
0.3948
K100000875
0.1236
K200000043
0.1924
K100000876
0.1594
0.3546
K200001161
K200000849
K100000522
0.1246
K200000743
0.1899
K100001110
0.1283
K200001419
0.6095
K100000185
K100000275
0.143
K200001027
0.1668
0.276
K100001189
0.2147
K100000322
0.176
K100000737
0.1805
0.2452
0.3578
K100001285
K200000193
K100000295
0.4373
0.2209
K200000152
K100001221
0.6911
0.2242
K200000774

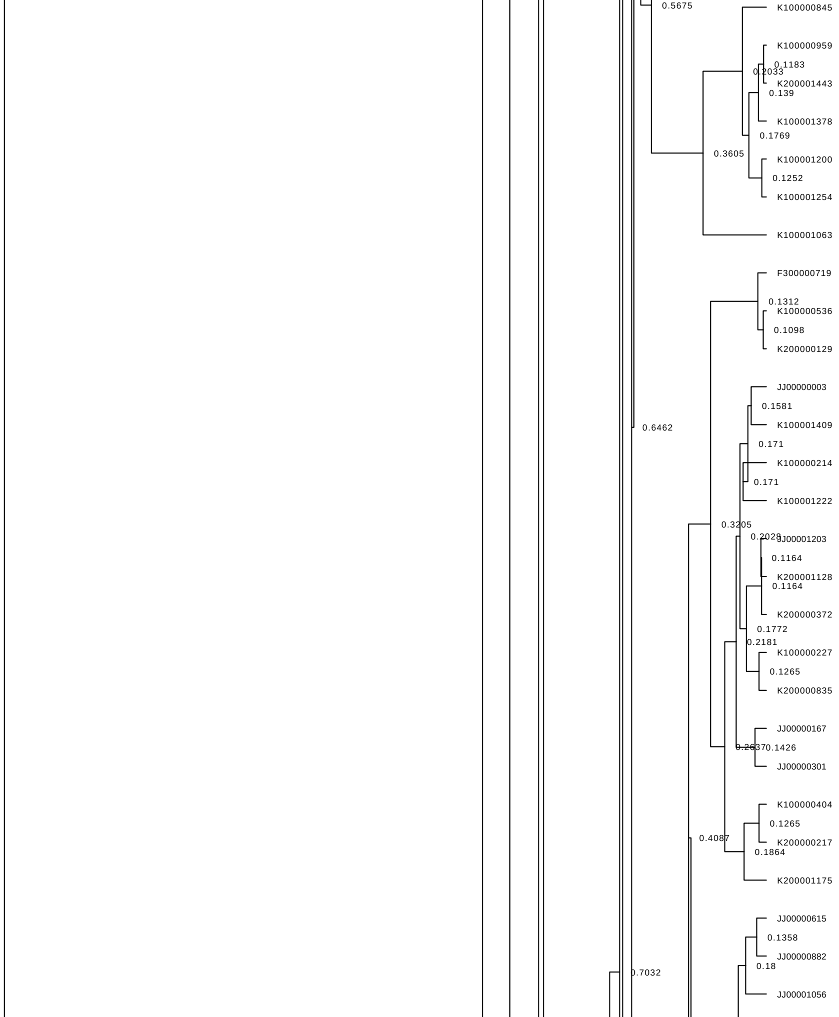

0.5675
K100000845
K100000959
0.1183
0.2033
K200001443
0.139
K100001378
0.1769
0.3605
K100001200
0.1252
K100001254
K100001063
F300000719
0.1312
K100000536
0.1098
K200000129
JJ00000003
0.1581
0.6462
K100001409
0.171
K100000214
0.171
K100001222
0.3205
0.2028
JJ00001203
0.1164
K200001128
0.1164
K200000372
0.1772
0.2181
K100000227
0.1265
K200000835
JJ00000167
0.2637
0.1426
JJ00000301
K100000404
0.1265
0.4087
K200000217
0.1864
K200001175
JJ00000615
0.1358
JJ00000882
0.18
0.7032
JJ00001056

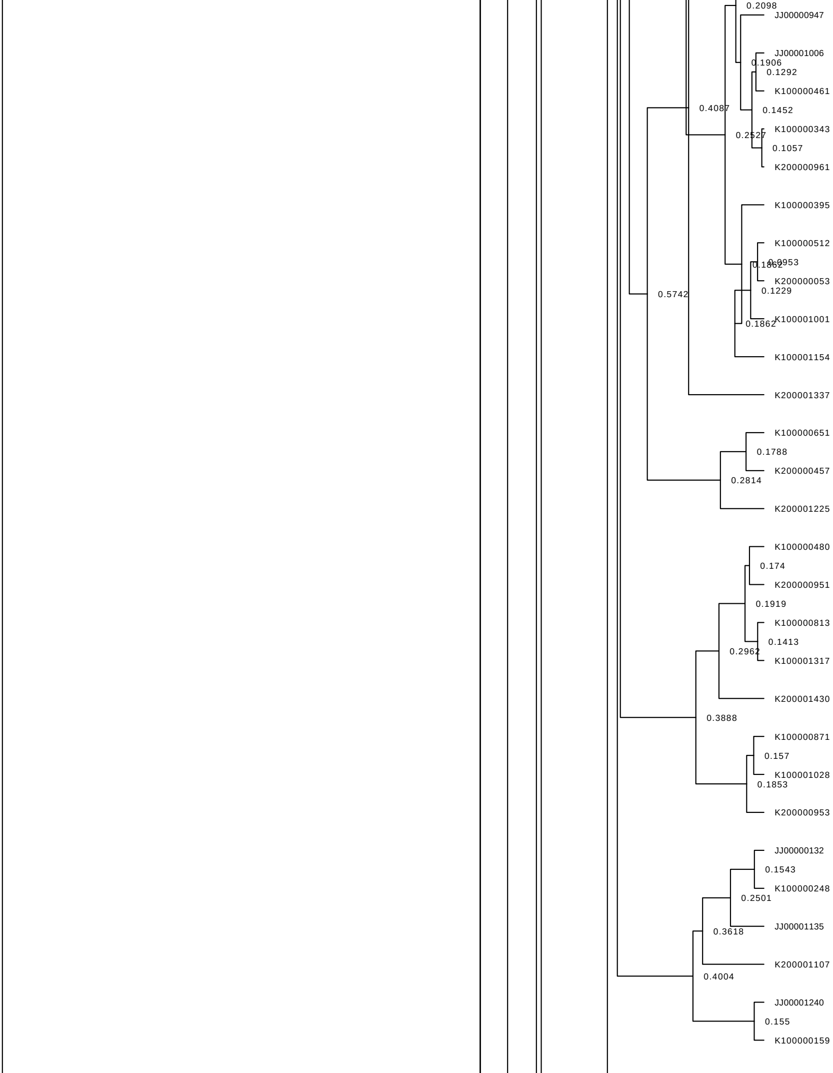
0.2098
JJ00000947
JJ00001006
0.1906
0.1292
K100000461
0.4087
0.1452
K100000343
0.2527
0.1057
K200000961
K100000395
K100000512
0.1862
0.0953
K200000053
0.5742
0.1229
K100001001
0.1862
K100001154
K200001337
K100000651
0.1788
K200000457
0.2814
K200001225
K100000480
0.174
K200000951
0.1919
K100000813
0.1413
0.2962
K100001317
K200001430
0.3888
K100000871
0.157
K100001028
0.1853
K200000953
JJ00000132
0.1543
K100000248
0.2501
JJ00001135
0.3618
K200001107
0.4004
JJ00001240
0.155
K100000159

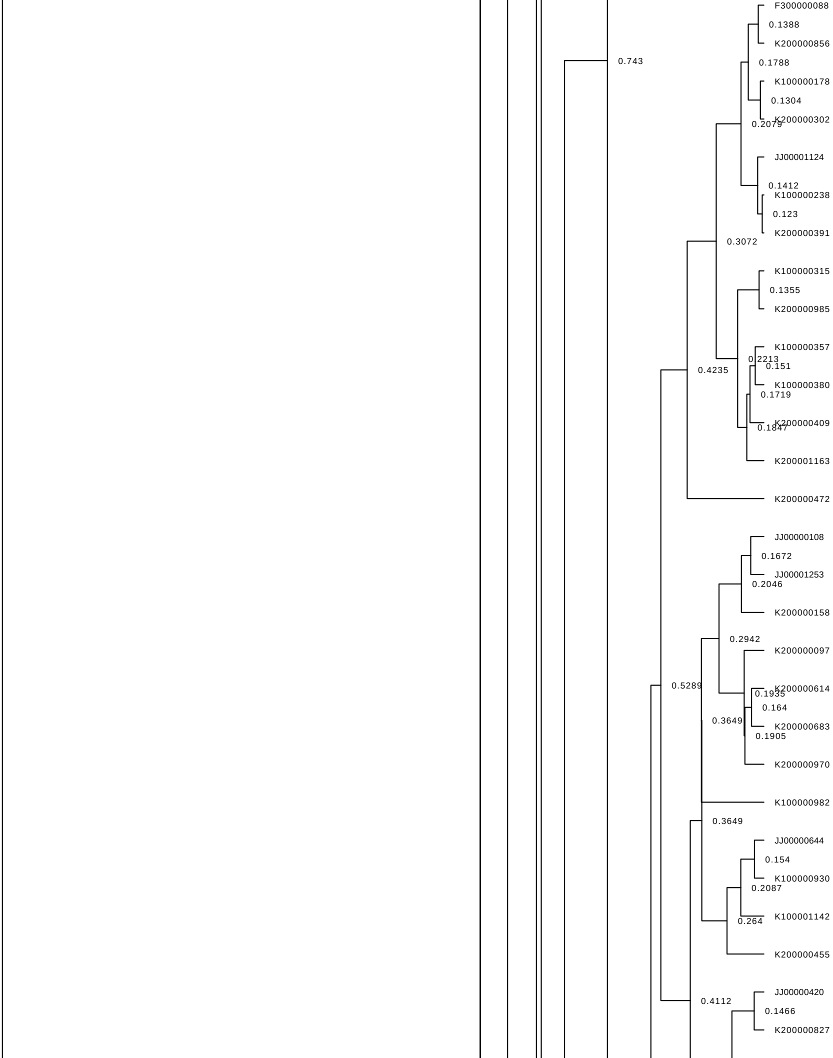
F300000088
0.1388
K200000856
0.743
0.1788
K100000178
0.1304
K200000302
0.2079
JJ00001124
0.1412
K100000238
0.123
K200000391
0.3072
K100000315
0.1355
K200000985
K100000357
0.2213
0.151
0.4235
K100000380
0.1719
K200000409
0.1847
K200001163
K200000472
JJ00000108
0.1672
JJ00001253
0.2046
K200000158
0.2942
K200000097
0.5289
K200000614
0.1935
0.164
0.3649
K200000683
0.1905
K200000970
K100000982
0.3649
JJ00000644
0.154
K100000930
0.2087
K100001142
0.264
K200000455
JJ00000420
0.4112
0.1466
K200000827

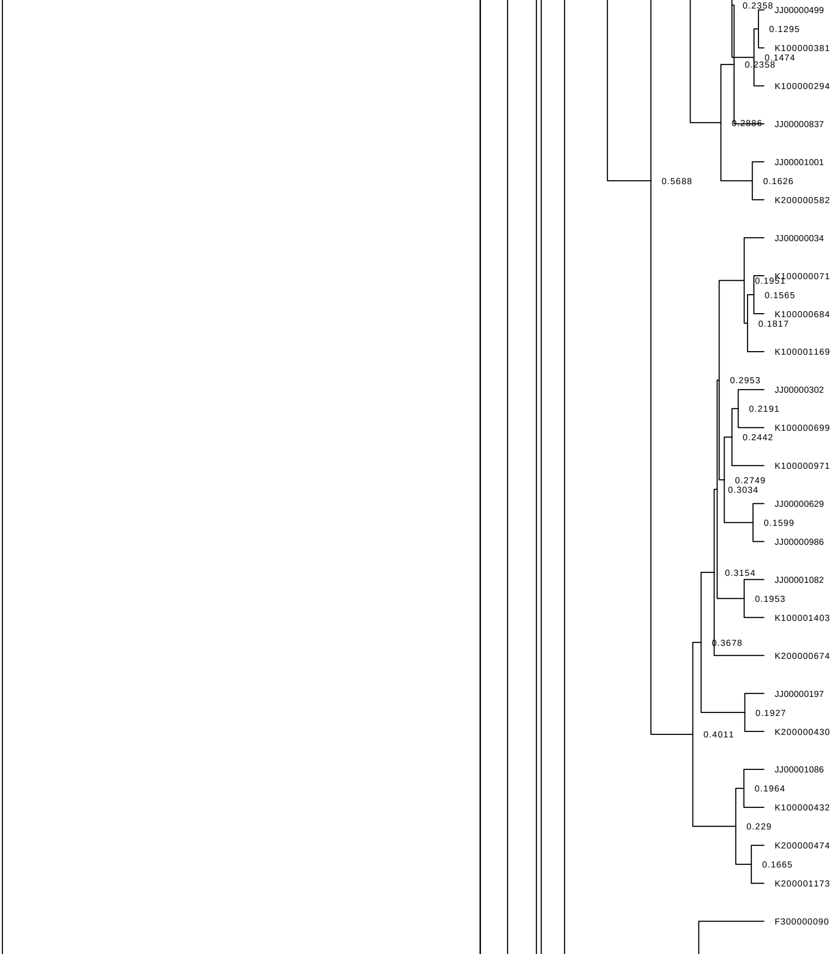
0.2358
JJ00000499
0.1295
K100000381
0.1474
0.2358
K100000294
0.2886
JJ00000837
JJ00001001
0.5688
0.1626
K200000582
JJ00000034
K100000071
0.1951
0.1565
K100000684
0.1817
K100001169
0.2953
JJ00000302
0.2191
K100000699
0.2442
K100000971
0.2749
0.3034
JJ00000629
0.1599
JJ00000986
0.3154
JJ00001082
0.1953
K100001403
0.3678
K200000674
JJ00000197
0.1927
K200000430
0.4011
JJ00001086
0.1964
K100000432
0.229
K200000474
0.1665
K200001173
F300000090

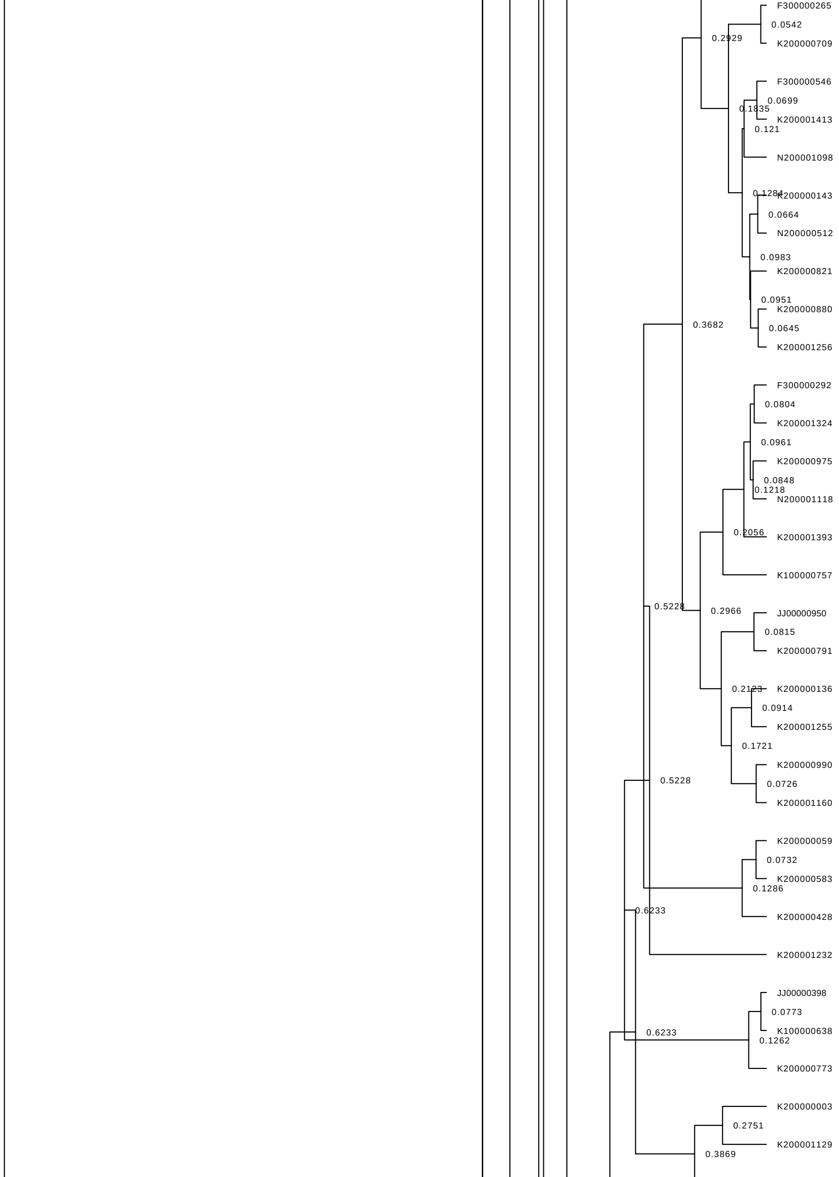
F300000265
0.0542
K200000709
0.2929
F300000546
0.0699
0.1835
K200001413
0.121
N200001098
0.1284
K200000143
0.0664
N200000512
0.0983
K200000821
0.0951
K200000880
0.3682
0.0645
K200001256
F300000292
0.0804
K200001324
0.0961
K200000975
0.0848
0.1218
N200001118
0.2056
K200001393
K100000757
0.5228
0.2966
JJ00000950
0.0815
K200000791
0.2123
K200000136
0.0914
K200001255
0.1721
K200000990
0.5228
0.0726
K200001160
K200000059
0.0732
K200000583
0.1286
0.6233
K200000428
K200001232
JJ00000398
0.0773
0.6233
K100000638
0.1262
K200000773
K200000003
0.2751
K200001129
0.3869

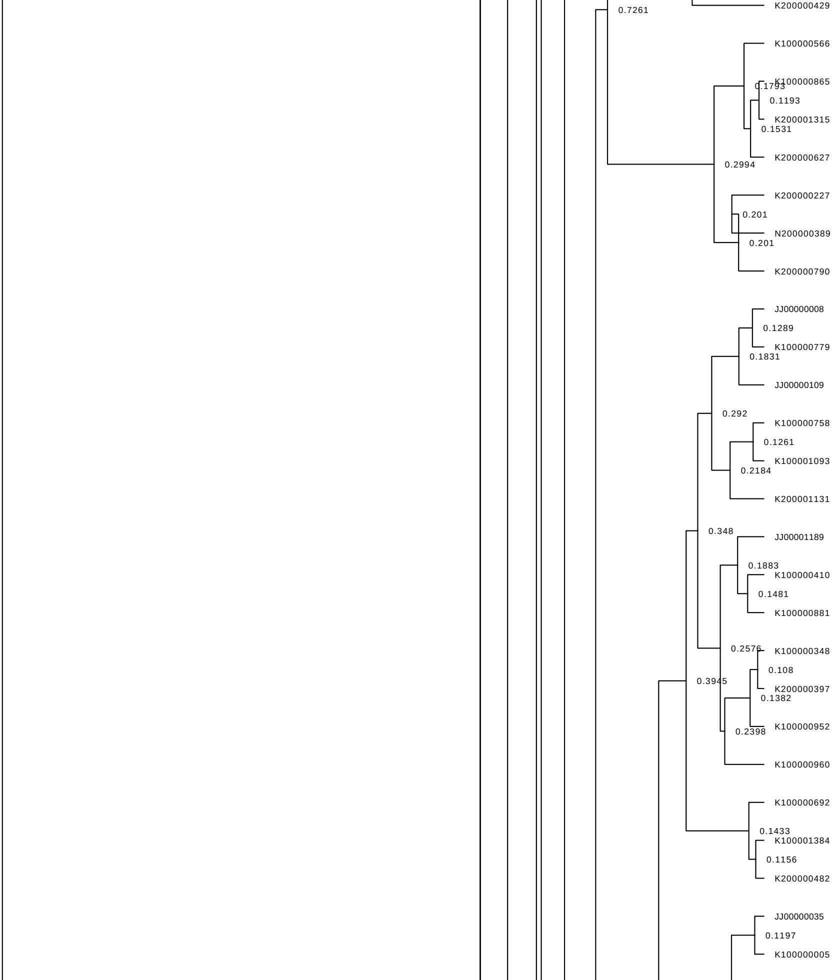
0.7261
K200000429
K100000566
K100000865
0.1793
0.1193
K200001315
0.1531
K200000627
0.2994
K200000227
0.201
N200000389
0.201
K200000790
JJ00000008
0.1289
K100000779
0.1831
JJ00000109
0.292
K100000758
0.1261
K100001093
0.2184
K200001131
0.348
JJ00001189
0.1883
K100000410
0.1481
K100000881
0.2576
K100000348
0.108
0.3945
K200000397
0.1382
K100000952
0.2398
K100000960
K100000692
0.1433
K100001384
0.1156
K200000482
JJ00000035
0.1197
K100000005

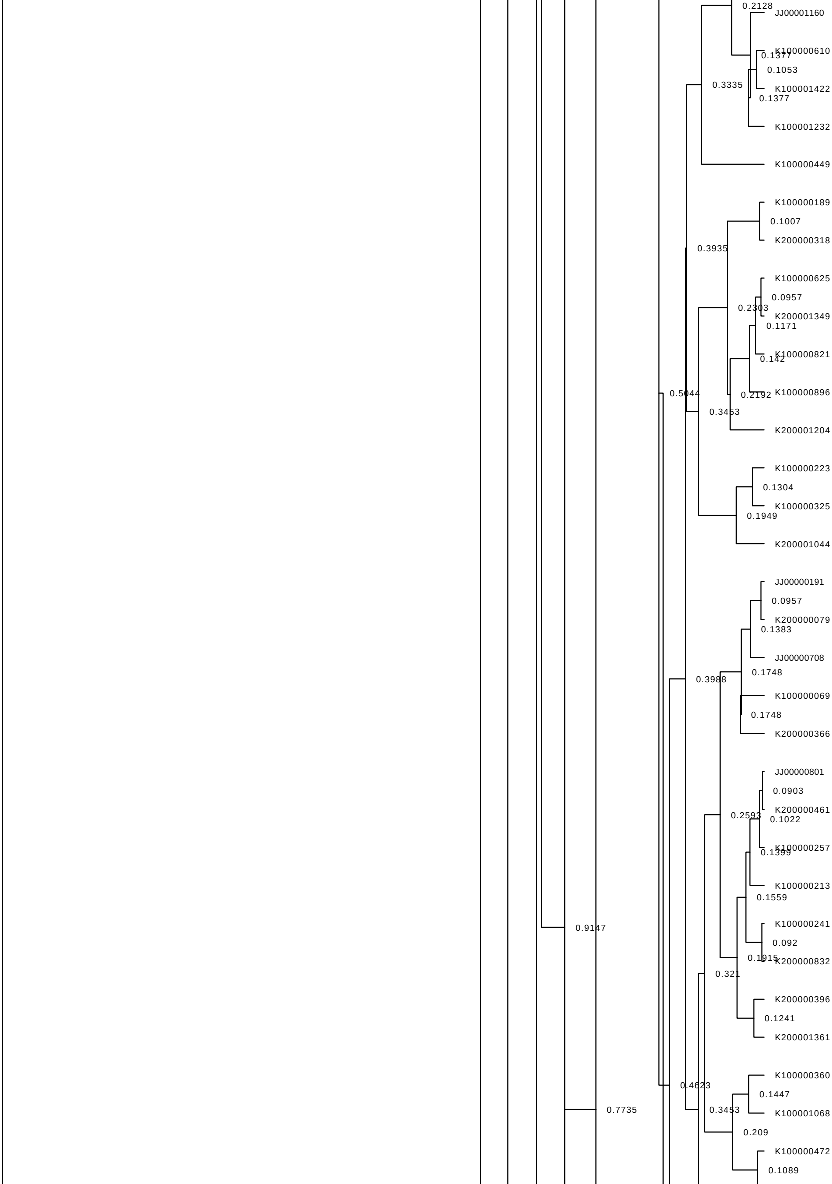

0.2128
JJ00001160
K100000610
0.1377
0.1053
0.3335
K100001422
0.1377
K100001232
K100000449
K100000189
0.1007
K200000318
0.3935
K100000625
0.0957
0.2303
K200001349
0.1171
K100000821
0.142
0.5044
0.2192
K100000896
0.3453
K200001204
K100000223
0.1304
K100000325
0.1949
K200001044
JJ00000191
0.0957
K200000079
0.1383
JJ00000708
0.1748
0.3988
K100000069
0.1748
K200000366
JJ00000801
0.0903
K200000461
0.2593
0.1022
K100000257
0.1399
K100000213
0.1559
0.9147
K100000241
0.092
0.1915
K200000832
0.321
K200000396
0.1241
K200001361
K100000360
0.4623
0.1447
0.7735
0.3453
K100001068
0.209
K100000472
0.1089

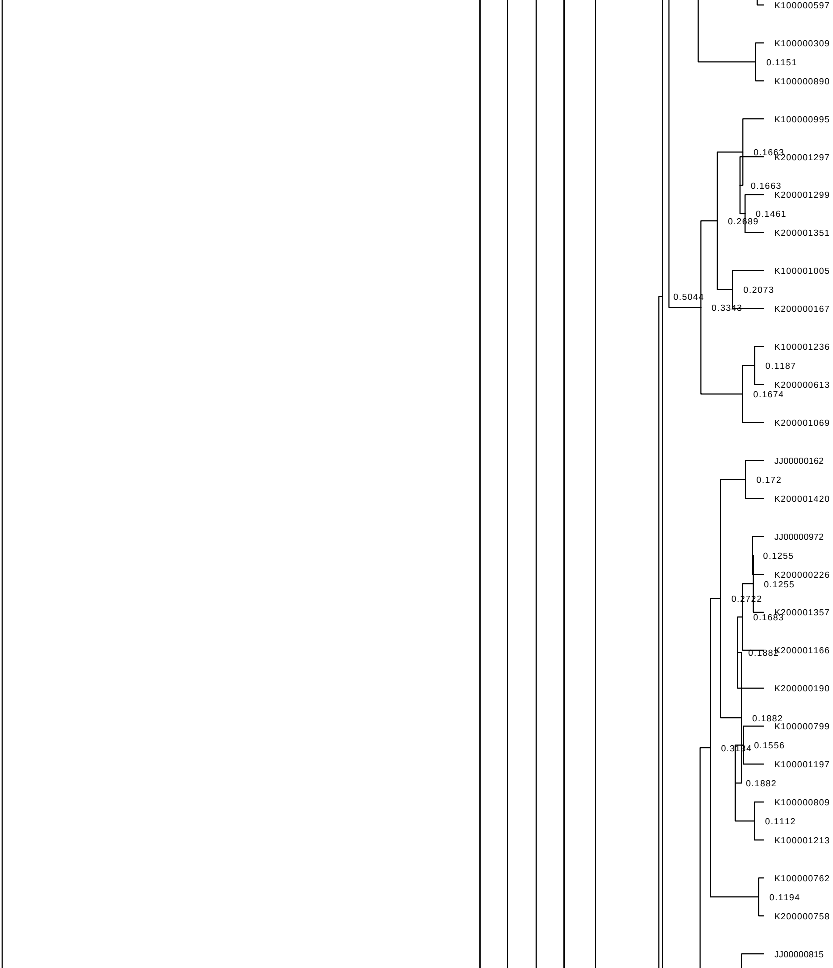
K100000597
K100000309
0.1151
K100000890
K100000995
0.1663
K200001297
0.1663
K200001299
0.1461
0.2689
K200001351
K100001005
0.2073
0.5044
0.3343
K200000167
K100001236
0.1187
K200000613
0.1674
K200001069
JJ00000162
0.172
K200001420
JJ00000972
0.1255
K200000226
0.1255
0.2722
K200001357
0.1683
K200001166
0.1882
K200000190
0.1882
K100000799
0.3134
0.1556
K100001197
0.1882
K100000809
0.1112
K100001213
K100000762
0.1194
K200000758
JJ00000815

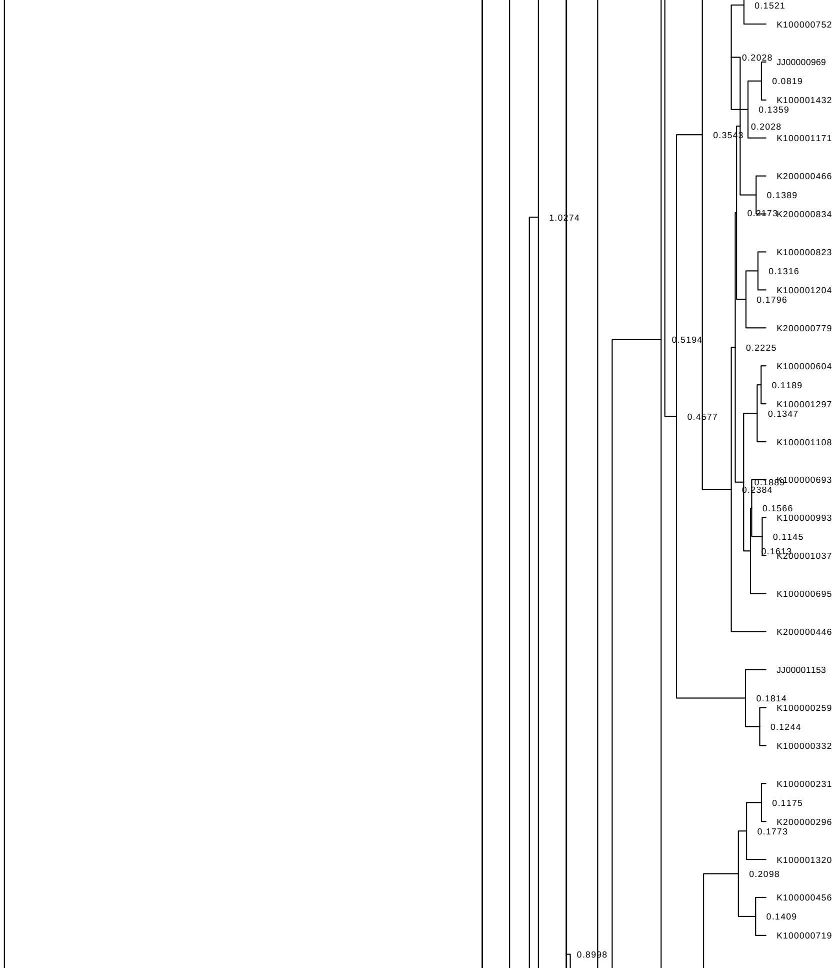

0.1521
K100000752
0.2028
JJ00000969
0.0819
K100001432
0.1359
0.2028
0.3543
K100001171
K200000466
0.1389
0.2173
K200000834
1.0274
K100000823
0.1316
K100001204
0.1796
K200000779
0.5194
0.2225
K100000604
0.1189
K100001297
0.4577
0.1347
K100001108
0.1889
K100000693
0.2384
0.1566
K100000993
0.1145
0.1613
K200001037
K100000695
K200000446
JJ00001153
0.1814
K100000259
0.1244
K100000332
K100000231
0.1175
K200000296
0.1773
K100001320
0.2098
K100000456
0.1409
K100000719
0.8998

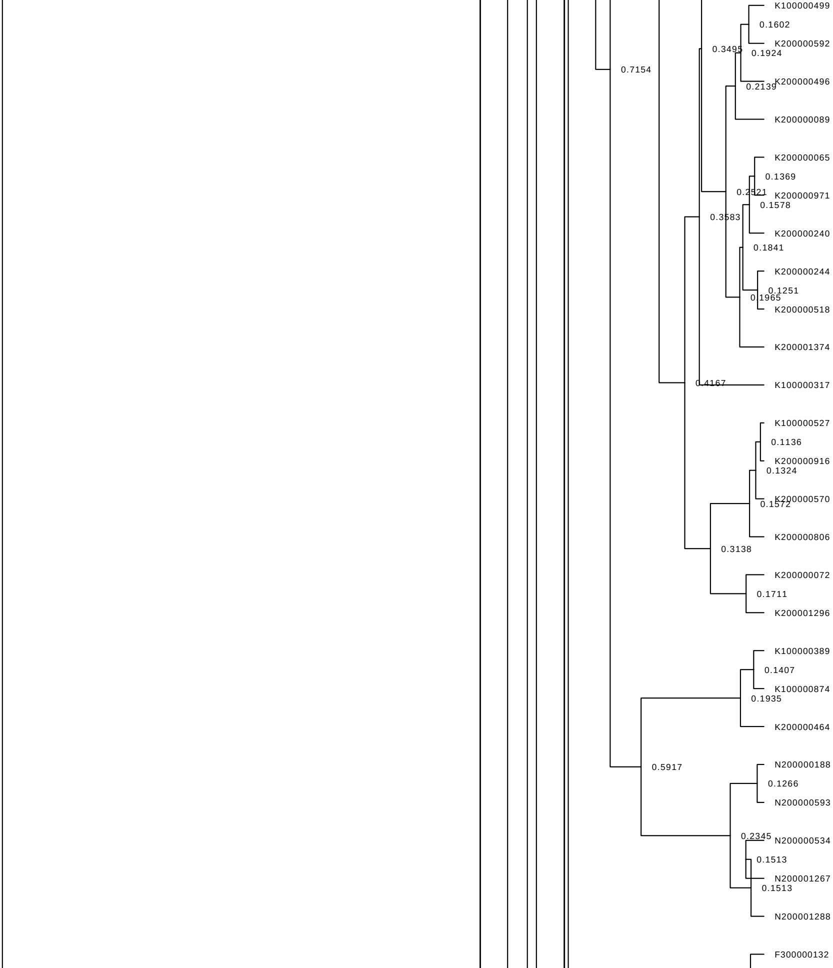

K100000499
0.1602
K200000592
0.3495
0.1924
0.7154
K200000496
0.2139
K200000089
K200000065
0.1369
0.2521
K200000971
0.1578
0.3583
K200000240
0.1841
K200000244
0.1251
0.1965
K200000518
K200001374
0.4167
K100000317
K100000527
0.1136
K200000916
0.1324
K200000570
0.1572
K200000806
0.3138
K200000072
0.1711
K200001296
K100000389
0.1407
K100000874
0.1935
K200000464
N200000188
0.5917
0.1266
N200000593
0.2345
N200000534
0.1513
N200001267
0.1513
N200001288
F300000132

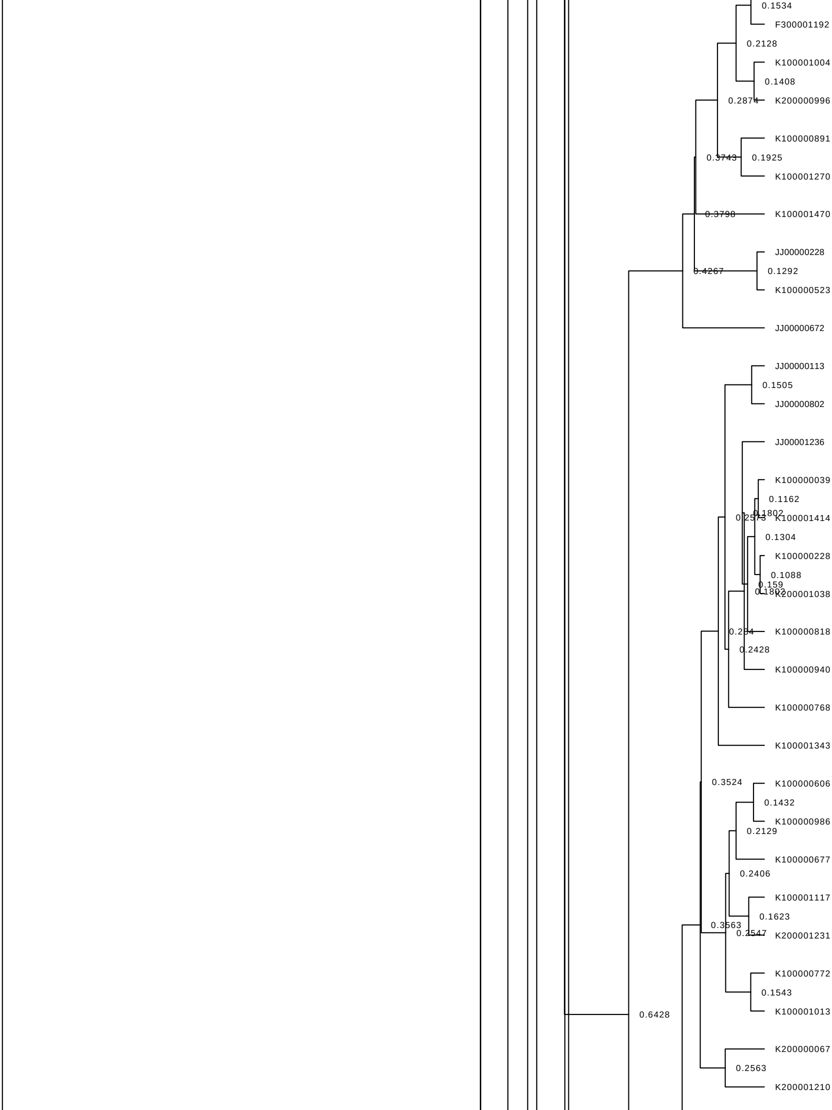

0.1534
F300001192
0.2128
K100001004
0.1408
0.2874
K200000996
K100000891
0.3743
0.1925
K100001270
0.3798
K100001470
JJ00000228
0.4267
0.1292
K100000523
JJ00000672
JJ00000113
0.1505
JJ00000802
JJ00001236
K100000039
0.1162
0.1802
0.2573
K100001414
0.1304
K100000228
0.1088
0.159
0.1802
K200001038
0.284
K100000818
0.2428
K100000940
K100000768
K100001343
0.3524
K100000606
0.1432
K100000986
0.2129
K100000677
0.2406
K100001117
0.1623
0.3563
0.2547
K200001231
K100000772
0.1543
K100001013
0.6428
K200000067
0.2563
K200001210

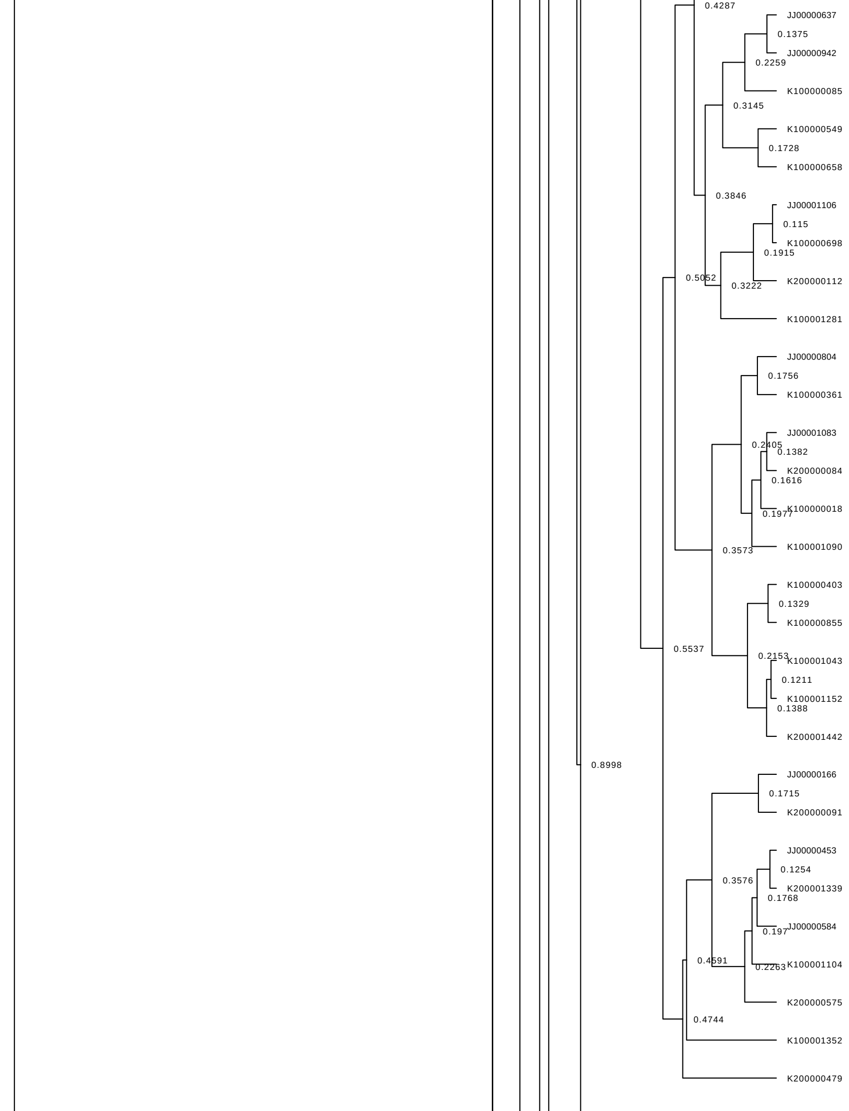
0.4287
JJ00000637
0.1375
JJ00000942
0.2259
K100000085
0.3145
K100000549
0.1728
K100000658
0.3846
JJ00001106
0.115
K100000698
0.1915
0.5052
K200000112
0.3222
K100001281
JJ00000804
0.1756
K100000361
JJ00001083
0.2405
0.1382
K200000084
0.1616
K100000018
0.1977
K100001090
0.3573
K100000403
0.1329
K100000855
0.5537
0.2153
K100001043
0.1211
K100001152
0.1388
K200001442
0.8998
JJ00000166
0.1715
K200000091
JJ00000453
0.1254
0.3576
K200001339
0.1768
JJ00000584
0.197
0.4591
K100001104
0.2263
K200000575
0.4744
K100001352
K200000479

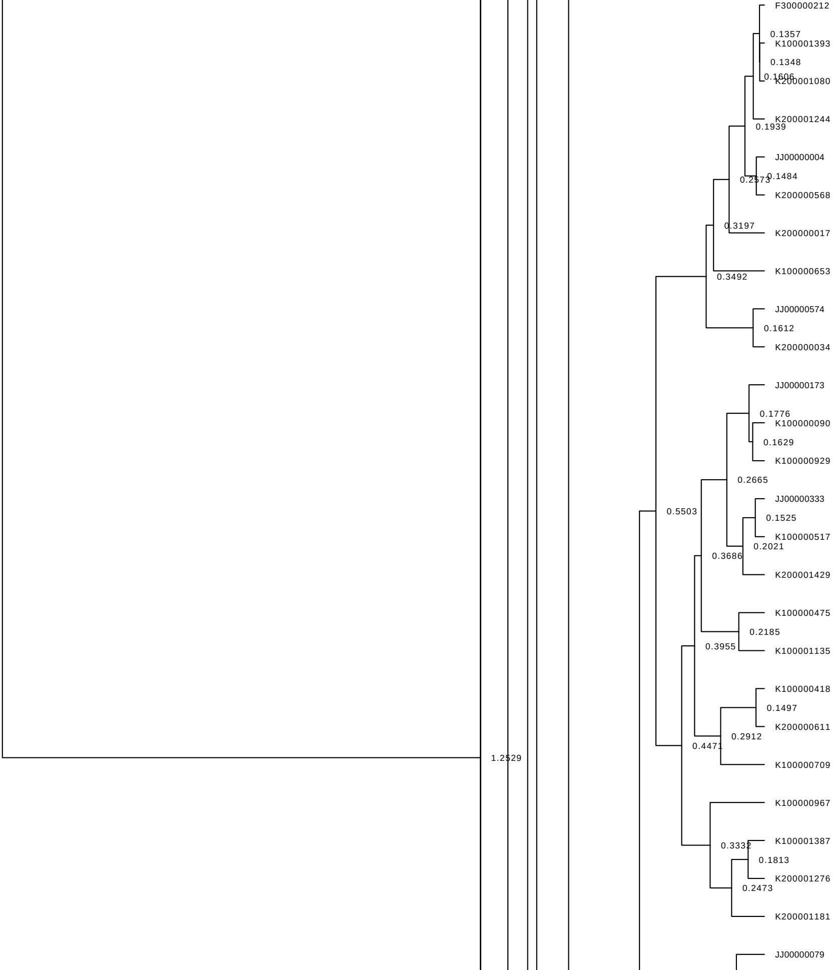

F300000212
0.1357
K100001393
0.1348
0.1606
K200001080
K200001244
0.1939
JJ00000004
0.2573
0.1484
K200000568
0.3197
K200000017
K100000653
0.3492
JJ00000574
0.1612
K200000034
JJ00000173
0.1776
K100000090
0.1629
K100000929
0.2665
JJ00000333
0.1525
0.5503
K100000517
0.2021
0.3686
K200001429
K100000475
0.2185
0.3955
K100001135
K100000418
0.1497
K200000611
0.2912
0.4471
1.2529
K100000709
K100000967
0.3332
K100001387
0.1813
K200001276
0.2473
K200001181
JJ00000079

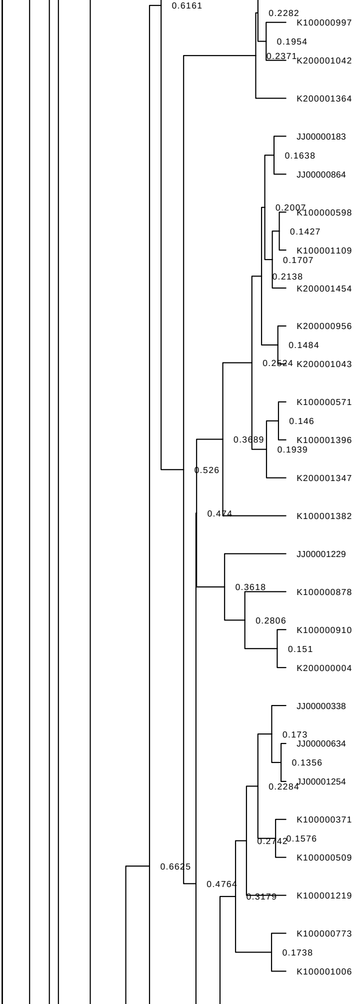
0.6161
0.2282
K100000997
0.1954
0.2371
K200001042
K200001364
JJ00000183
0.1638
JJ00000864
0.2007
K100000598
0.1427
K100001109
0.1707
0.2138
K200001454
K200000956
0.1484
0.2524
K200001043
K100000571
0.146
K100001396
0.3689
0.1939
K200001347
0.526
0.474
K100001382
JJ00001229
0.3618
K100000878
0.2806
K100000910
0.151
K200000004
JJ00000338
0.173
JJ00000634
0.1356
JJ00001254
0.2284
K100000371
0.1576
0.2742
K100000509
0.6625
0.4764
K100001219
0.3179
K100000773
0.1738
K100001006

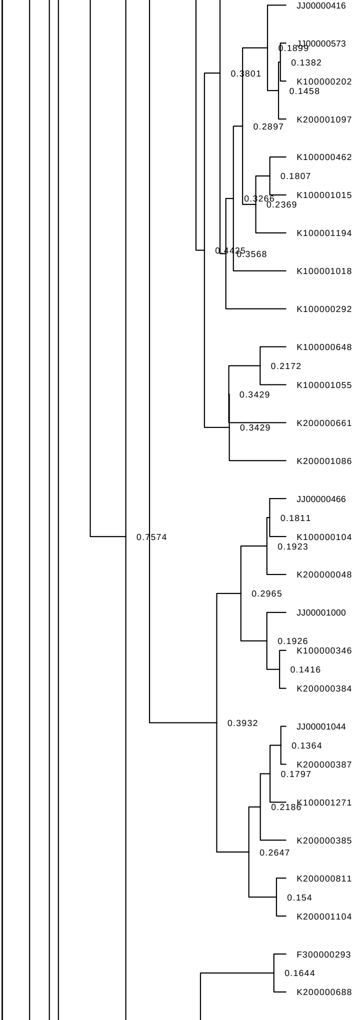

JJ00000416
JJ00000573
0.1899
0.1382
K100000202
0.3801
0.1458
K200001097
0.2897
K100000462
0.1807
K100001015
0.3266
0.2369
K100001194
0.4425
0.3568
K100001018
K100000292
K100000648
0.2172
K100001055
0.3429
K200000661
0.3429
K200001086
JJ00000466
0.1811
K100000104
0.7574
0.1923
K200000048
0.2965
JJ00001000
0.1926
K100000346
0.1416
K200000384
0.3932
JJ00001044
0.1364
K200000387
0.1797
K100001271
0.2186
K200000385
0.2647
K200000811
0.154
K200001104
F300000293
0.1644
K200000688

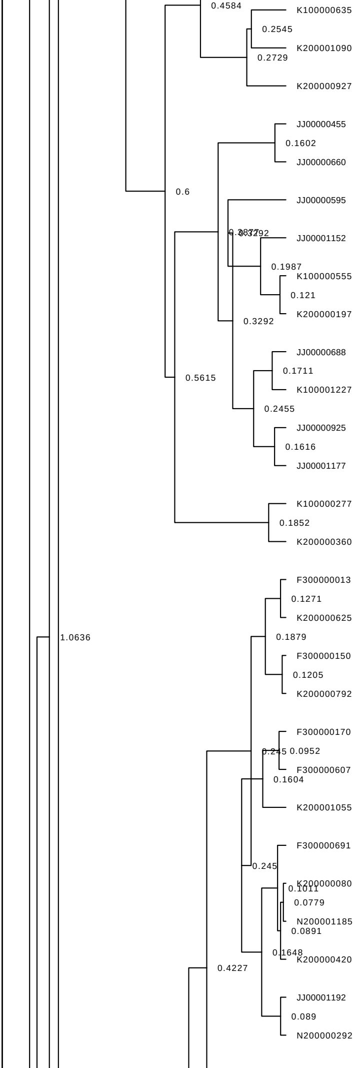
0.4584
K100000635
0.2545
K200001090
0.2729
K200000927
JJ00000455
0.1602
JJ00000660
0.6
JJ00000595
0.3877
0.3292
JJ00001152
0.1987
K100000555
0.121
K200000197
0.3292
JJ00000688
0.1711
K100001227
0.5615
0.2455
JJ00000925
0.1616
JJ00001177
K100000277
0.1852
K200000360
F300000013
0.1271
K200000625
1.0636
0.1879
F300000150
0.1205
K200000792
F300000170
0.245
0.0952
F300000607
0.1604
K200001055
F300000691
0.245
K200000080
0.1011
0.0779
N200001185
0.0891
0.1648
K200000420
0.4227
JJ00001192
0.089
N200000292

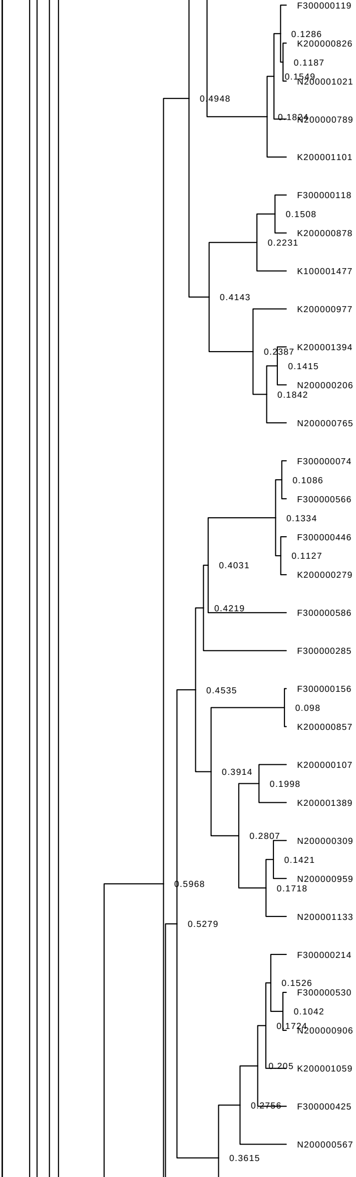
F300000119
0.1286
K200000826
0.1187
0.1549
N200001021
0.4948
0.1824
N200000789
K200001101
F300000118
0.1508
K200000878
0.2231
K100001477
0.4143
K200000977
K200001394
0.2387
0.1415
N200000206
0.1842
N200000765
F300000074
0.1086
F300000566
0.1334
F300000446
0.1127
0.4031
K200000279
0.4219
F300000586
F300000285
F300000156
0.4535
0.098
K200000857
K200000107
0.3914
0.1998
K200001389
0.2807
N200000309
0.1421
N200000959
0.5968
0.1718
N200001133
0.5279
F300000214
0.1526
F300000530
0.1042
0.1724
N200000906
0.205
K200001059
0.2756
F300000425
N200000567
0.3615

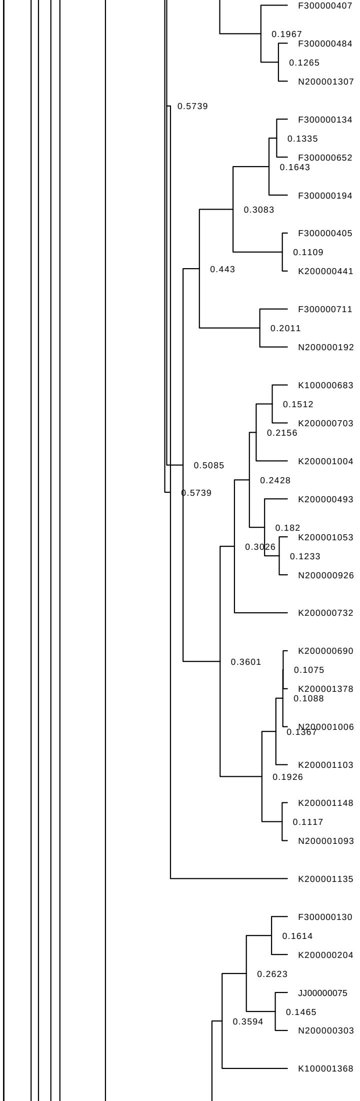
F300000407
0.1967
F300000484
0.1265
N200001307
0.5739
F300000134
0.1335
F300000652
0.1643
F300000194
0.3083
F300000405
0.1109
K200000441
0.443
F300000711
0.2011
N200000192
K100000683
0.1512
K200000703
0.2156
K200001004
0.5085
0.2428
0.5739
K200000493
0.182
K200001053
0.3026
0.1233
N200000926
K200000732
K200000690
0.3601
0.1075
K200001378
0.1088
N200001006
0.1367
K200001103
0.1926
K200001148
0.1117
N200001093
K200001135
F300000130
0.1614
K200000204
0.2623
JJ00000075
0.1465
0.3594
N200000303
K100001368

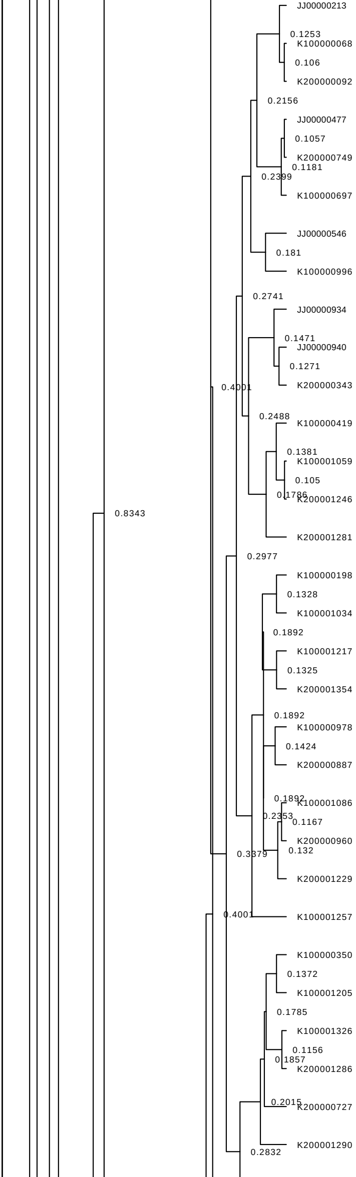
JJ00000213
0.1253
K100000068
0.106
K200000092
0.2156
JJ00000477
0.1057
K200000749
0.1181
0.2399
K100000697
JJ00000546
0.181
K100000996
0.2741
JJ00000934
0.1471
JJ00000940
0.1271
K200000343
0.4001
0.2488
K100000419
0.1381
K100001059
0.105
0.1786
K200001246
0.8343
K200001281
0.2977
K100000198
0.1328
K100001034
0.1892
K100001217
0.1325
K200001354
0.1892
K100000978
0.1424
K200000887
0.1892
K100001086
0.2353
0.1167
K200000960
0.132
0.3379
K200001229
0.4001
K100001257
K100000350
0.1372
K100001205
0.1785
K100001326
0.1156
0.1857
K200001286
0.2015
K200000727
K200001290
0.2832

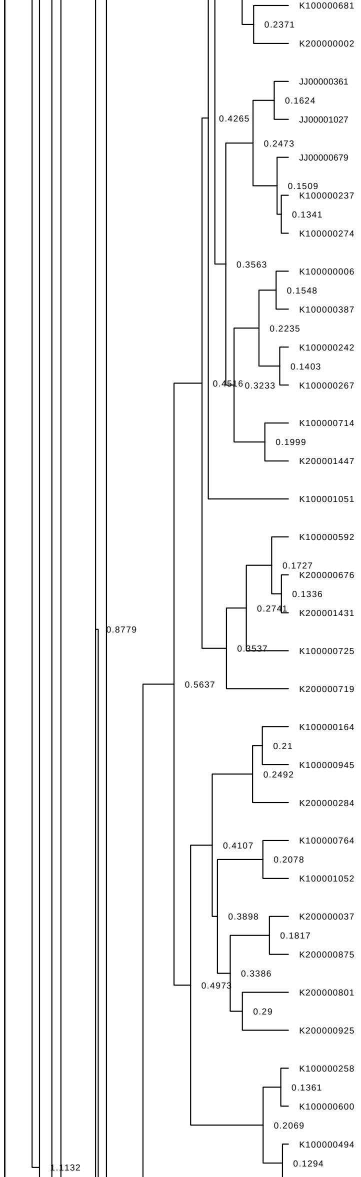
K100000681
0.2371
K200000002
JJ00000361
0.1624
0.4265
JJ00001027
0.2473
JJ00000679
0.1509
K100000237
0.1341
K100000274
0.3563
K100000006
0.1548
K100000387
0.2235
K100000242
0.1403
0.4516
0.3233
K100000267
K100000714
0.1999
K200001447
K100001051
K100000592
0.1727
K200000676
0.1336
0.2741
K200001431
0.8779
0.3537
K100000725
0.5637
K200000719
K100000164
0.21
K100000945
0.2492
K200000284
K100000764
0.4107
0.2078
K100001052
0.3898
K200000037
0.1817
K200000875
0.3386
0.4973
K200000801
0.29
K200000925
K100000258
0.1361
K100000600
0.2069
K100000494
0.1294
1.1132

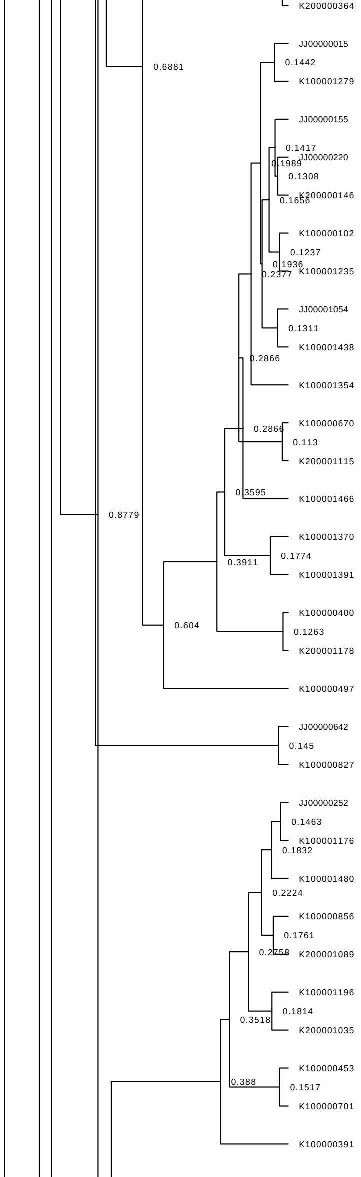
K200000364
JJ00000015
0.1442
K100001279
0.6881
JJ00000155
0.1417
JJ00000220
0.1989
0.1308
K200000146
0.1656
K100000102
0.1237
0.1936
K100001235
0.2377
JJ00001054
0.1311
K100001438
0.2866
K100001354
K100000670
0.2866
0.113
K200001115
0.3595
K100001466
0.8779
K100001370
0.1774
0.3911
K100001391
K100000400
0.604
0.1263
K200001178
K100000497
JJ00000642
0.145
K100000827
JJ00000252
0.1463
K100001176
0.1832
K100001480
0.2224
K100000856
0.1761
0.2758
K200001089
K100001196
0.1814
0.3518
K200001035
K100000453
0.388
0.1517
K100000701
K100000391

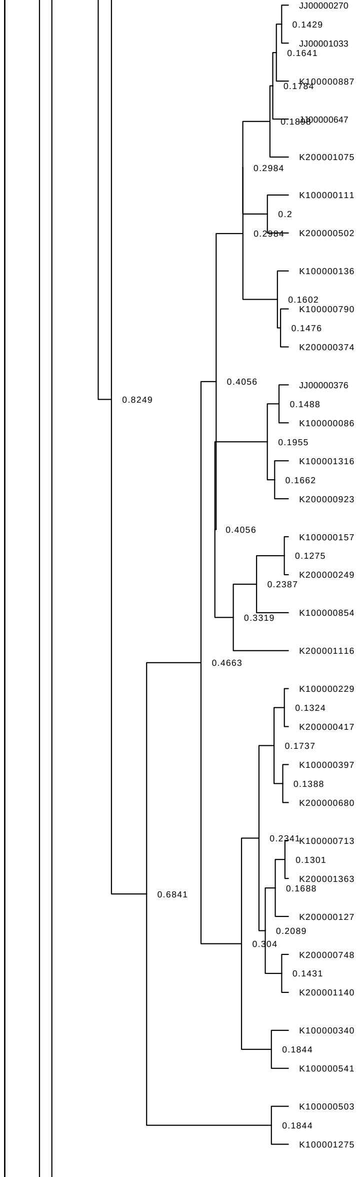
JJ00000270
0.1429
JJ00001033
0.1641
K100000887
0.1784
JJ00000647
0.1898
K200001075
0.2984
K100000111
0.2
K200000502
0.2984
K100000136
0.1602
K100000790
0.1476
K200000374
0.4056
JJ00000376
0.8249
0.1488
K100000086
0.1955
K100001316
0.1662
K200000923
0.4056
K100000157
0.1275
K200000249
0.2387
K100000854
0.3319
K200001116
0.4663
K100000229
0.1324
K200000417
0.1737
K100000397
0.1388
K200000680
0.2341
K100000713
0.1301
K200001363
0.1688
0.6841
K200000127
0.2089
0.304
K200000748
0.1431
K200001140
K100000340
0.1844
K100000541
K100000503
0.1844
K100001275

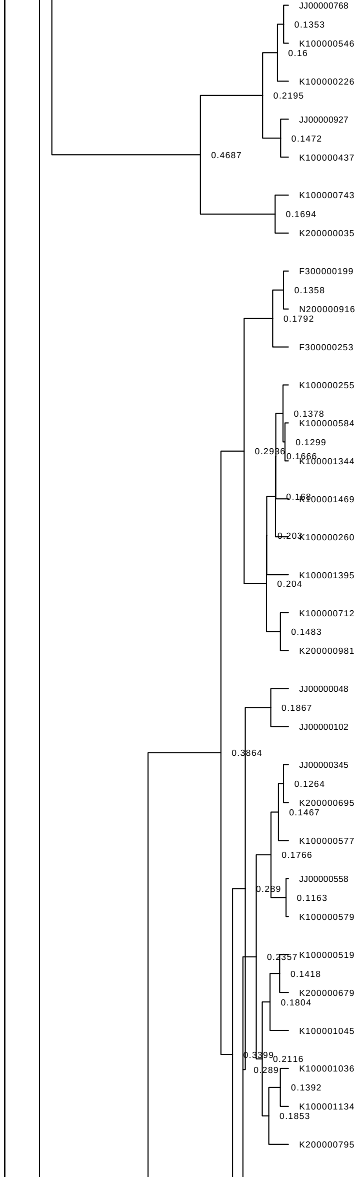
JJ00000768
0.1353
K100000546
0.16
K100000226
0.2195
JJ00000927
0.1472
K100000437
0.4687
K100000743
0.1694
K200000035
F300000199
0.1358
N200000916
0.1792
F300000253
K100000255
0.1378
K100000584
0.1299
0.2936
0.1666
K100001344
0.168
K100001469
0.203
K100000260
K100001395
0.204
K100000712
0.1483
K200000981
JJ00000048
0.1867
JJ00000102
0.3864
JJ00000345
0.1264
K200000695
0.1467
K100000577
0.1766
JJ00000558
0.289
0.1163
K100000579
0.2357
K100000519
0.1418
K200000679
0.1804
K100001045
0.3399
0.2116
0.289
K100001036
0.1392
K100001134
0.1853
K200000795

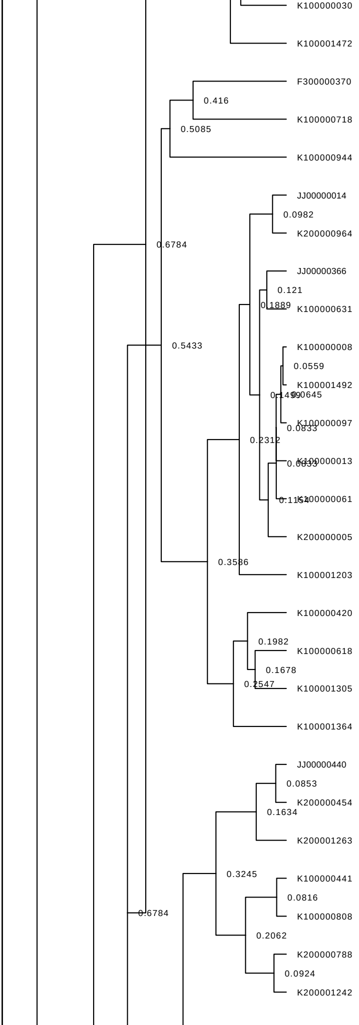

K100000030
K100001472
F300000370
0.416
K100000718
0.5085
K100000944
JJ00000014
0.0982
K200000964
0.6784
JJ00000366
0.121
0.1889
K100000631
0.5433
K100000008
0.0559
K100001492
K100000097
0.0833
0.2312
K100000013
K100000061
K200000005
0.3586
K100001203
K100000420
0.1982
K100000618
0.1678
0.2547
K100001305
K100001364
JJ00000440
0.0853
K200000454
0.1634
K200001263
0.3245
K100000441
0.0816
K100000808
0.6784
0.2062
K200000788
0.0924
K200001242

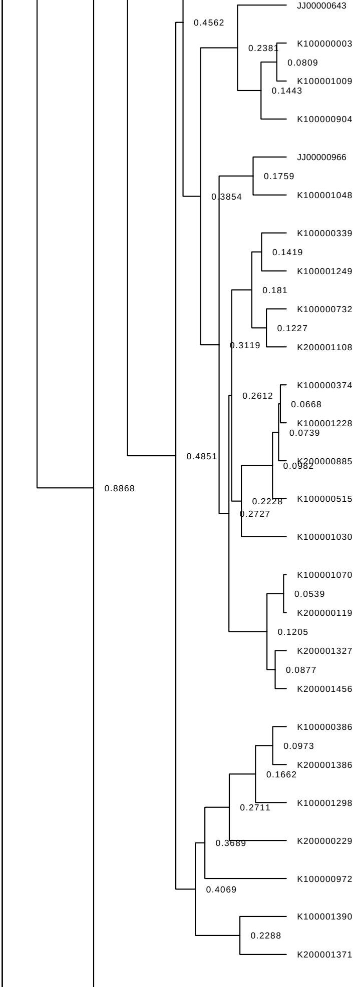
JJ00000643
0.4562
K100000003
0.2381
0.0809
K100001009
0.1443
K100000904
JJ00000966
0.1759
K100001048
0.3854
K100000339
0.1419
K100001249
0.181
K100000732
0.1227
K200001108
0.3119
K100000374
0.2612
0.0668
K100001228
0.0739
K200000885
0.0982
0.4851
0.8868
K100000515
0.2228
0.2727
K100001030
K100001070
0.0539
K200000119
0.1205
K200001327
0.0877
K200001456
K100000386
0.0973
K200001386
0.1662
K100001298
0.2711
K200000229
0.3689
K100000972
0.4069
K100001390
0.2288
K200001371

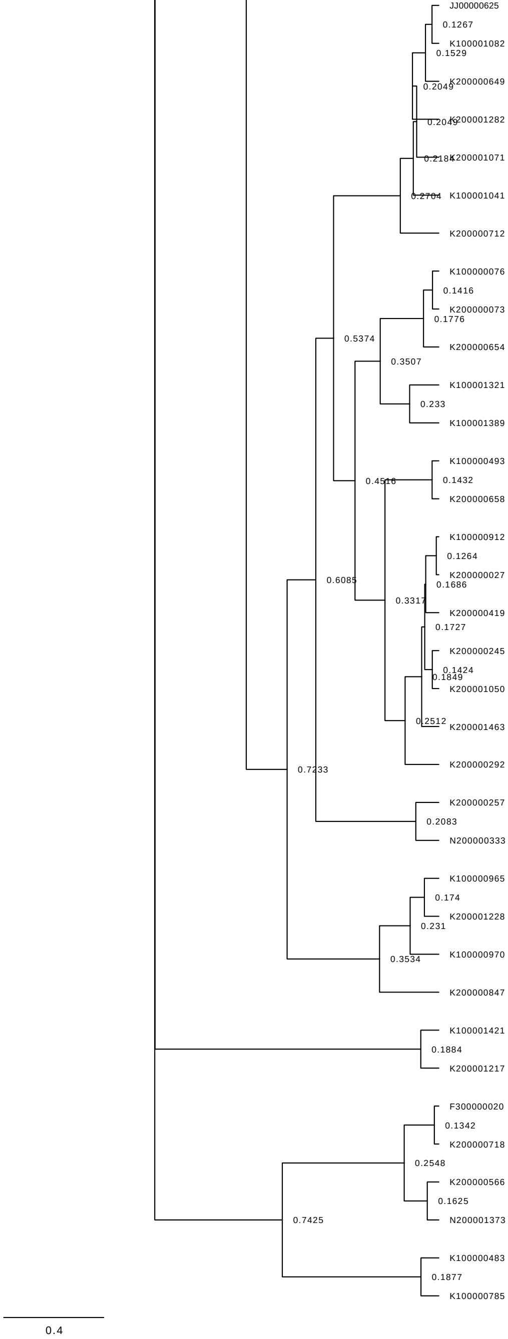
JJ00000625
0.1267
K100001082
0.1529
K200000649
0.2049
K200001282
0.2049
K200001071
0.2184
K100001041
0.2704
K200000712
K100000076
0.1416
K200000073
0.1776
0.5374
K200000654
0.3507
K100001321
0.233
K100001389
K100000493
0.1432
0.4516
K200000658
K100000912
0.1264
K200000027
0.1686
0.6085
0.3317
K200000419
0.1727
K200000245
0.1424
0.1849
K200001050
0.2512
K200001463
K200000292
0.7233
K200000257
0.2083
N200000333
K100000965
0.174
K200001228
0.231
K100000970
0.3534
K200000847
K100001421
0.1884
K200001217
F300000020
0.1342
K200000718
0.2548
K200000566
0.1625
0.7425
N200001373
K100000483
0.1877
K100000785
0.4
