# Supplementary material for: Dispersal history of Miniopterus fuliginosus bats and their associated viruses in east Asia
Source: PLoS One. 2021 Jan 14;16(1):e0244006. doi: 10.1371/journal.pone.0244006 (PMC7808576; doi:10.1371/journal.pone.0244006)
Supplement: S4 Fig — The nucleotide substitution rate was 20% per site per million years. The time-scaled phylogeny was summarized from all MCMC phylogenies of the D-loop DNA data set analyzed under HKY+G and Coalescent constant size models in BEAST version 2.6.0. The number indicates the earliest divergence time (million years ago). (PDF) [file pone.0244006.s004.pdf]

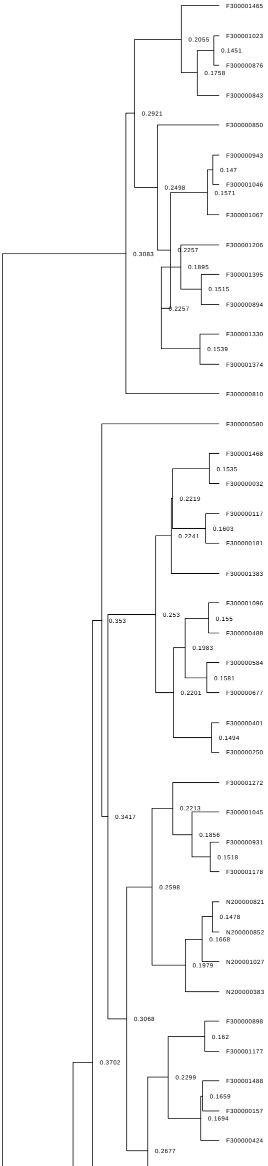
F300001465
0.2055
F300001023
0.1451
F300000876
0.1758
F300000843
0.2921
F300000850
F300000943
0.147
F300001046
0.2498
0.1571
F300001067
F300001206
0.2257
0.1895
F300001395
0.1515
F300000894
0.2257
F300001330
0.1539
F300001374
0.3083
F300000810
F300000580
F300001468
0.1535
F300000032
0.2219
F300000117
0.1603
F300000181
0.2241
F300001383
F300001096
0.253
0.155
0.353
F300000488
0.1983
F300000584
0.1581
0.2201
F300000677
F300000401
0.1494
F300000250
F300001272
0.2213
F300001045
0.3417
0.1856
F300000931
0.1518
F300001178
0.2598
N200000821
0.1478
N200000852
0.1668
N200001027
0.1979
N200000383
0.3068
F300000898
0.162
F300001177
0.3702
F300001488
0.2299
0.1659
F300000157
0.1694
F300000424
0.2677

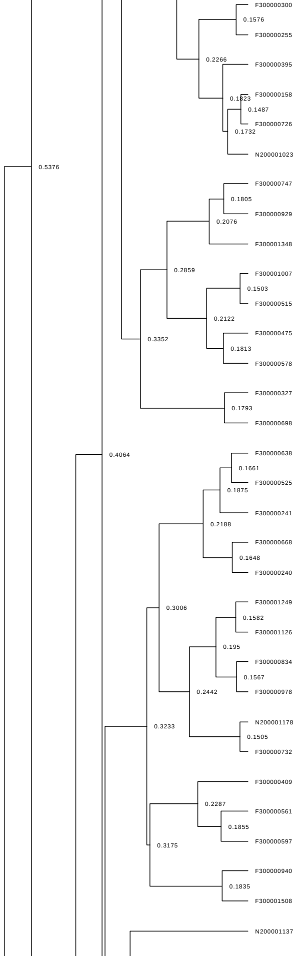
F300000300
0.1576
F300000255
0.2266
F300000395
F300000158
0.1823
0.1487
F300000726
0.1732
N200001023
0.5376
F300000747
0.1805
F300000929
0.2076
F300001348
0.2859
F300001007
0.1503
F300000515
0.2122
F300000475
0.3352
0.1813
F300000578
F300000327
0.1793
F300000698
0.4064
F300000638
0.1661
F300000525
0.1875
F300000241
0.2188
F300000668
0.1648
F300000240
F300001249
0.3006
0.1582
F300001126
0.195
F300000834
0.1567
F300000978
0.2442
N200001178
0.3233
0.1505
F300000732
F300000409
0.2287
F300000561
0.1855
F300000597
0.3175
F300000940
0.1835
F300001508
N200001137

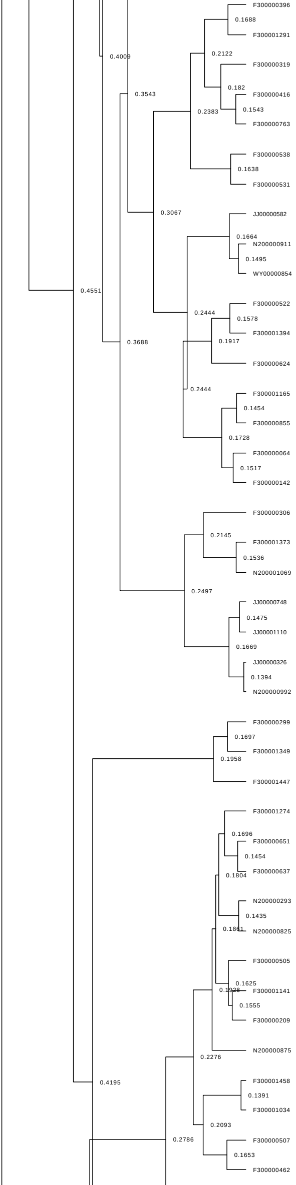
F300000396
0.1688
F300001291
0.4009
0.2122
F300000319
0.3543
0.182
F300000416
0.2383
0.1543
F300000763
F300000538
0.1638
F300000531
0.3067
JJ00000582
0.1664
N200000911
0.1495
WY00000854
0.4551
F300000522
0.2444
0.1578
F300001394
0.3688
0.1917
F300000624
0.2444
F300001165
0.1454
F300000855
0.1728
F300000064
0.1517
F300000142
F300000306
0.2145
F300001373
0.1536
N200001069
0.2497
JJ00000748
0.1475
JJ00001110
0.1669
JJ00000326
0.1394
N200000992
F300000299
0.1697
F300001349
0.1958
F300001447
F300001274
0.1696
F300000651
0.1454
F300000637
0.1804
N200000293
0.1435
N200000825
F300000505
0.1625
F300001141
0.1555
F300000209
N200000875
0.2276
F300001458
0.4195
0.1391
F300001034
0.2093
0.2786
F300000507
0.1653
F300000462

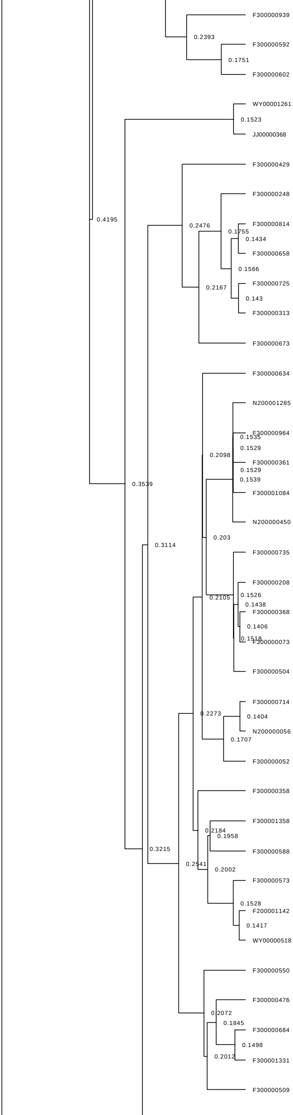
F300000939
0.2393
F300000592
0.1751
F300000602
WY00001261
0.1523
JJ00000368
F300000429
F300000248
0.4195
F300000814
0.2476
0.1755
0.1434
F300000658
0.1566
F300000725
0.2167
0.143
F300000313
F300000673
F300000634
N200001285
F300000964
0.1535
0.1529
0.2098
F300000361
0.1529
0.1539
0.3539
F300001084
N200000450
0.203
0.3114
F300000735
F300000208
0.1526
0.2105
0.1438
F300000368
0.1406
0.1518
F300000073
F300000504
F300000714
0.2273
0.1404
N200000056
0.1707
F300000052
F300000358
F300001358
0.2184
0.1958
F300000588
0.3215
0.2541
0.2002
F300000573
0.1528
F200001142
0.1417
WY00000518
F300000550
F300000476
0.2072
0.1845
F300000684
0.1498
0.2012
F300001331
F300000509

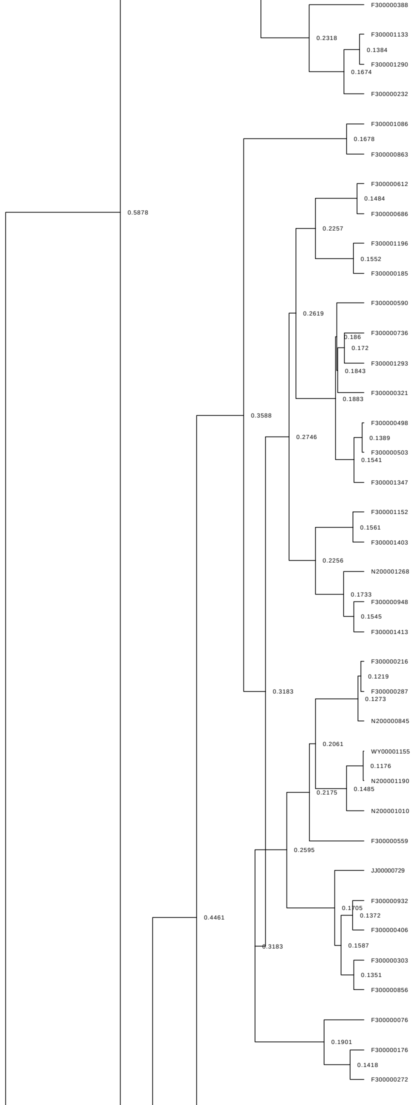
F300000388
0.2318
F300001133
0.1384
F300001290
0.1674
F300000232
F300001086
0.1678
F300000863
F300000612
0.1484
F300000686
0.5878
0.2257
F300001196
0.1552
F300000185
F300000590
0.2619
F300000736
0.186
0.172
F300001293
0.1843
F300000321
0.1883
0.3588
F300000498
0.1389
0.2746
F300000503
0.1541
F300001347
F300001152
0.1561
F300001403
0.2256
N200001268
0.1733
F300000948
0.1545
F300001413
F300000216
0.1219
F300000287
0.3183
0.1273
N200000845
0.2061
WY00001155
0.1176
N200001190
0.1485
0.2175
N200001010
F300000559
0.2595
JJ00000729
F300000932
0.1705
0.1372
0.4461
F300000406
0.3183
0.1587
F300000303
0.1351
F300000856
F300000076
0.1901
F300000176
0.1418
F300000272

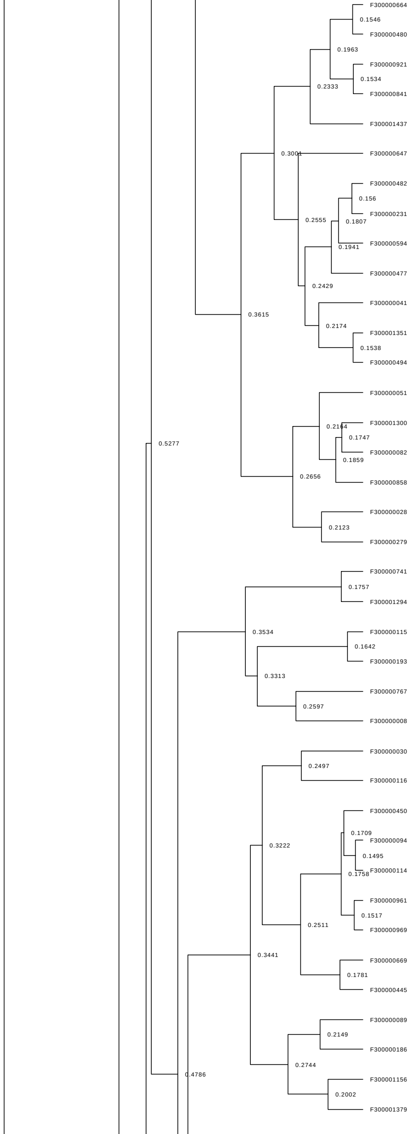

F300000664
0.1546
F300000480
0.1963
F300000921
0.1534
0.2333
F300000841
F300001437
0.3001
F300000647
F300000482
0.156
F300000231
0.2555
0.1807
0.1941
F300000594
F300000477
0.2429
F300000041
0.3615
0.2174
F300001351
0.1538
F300000494
F300000051
0.2164
F300001300
0.1747
0.5277
F300000082
0.1859
F300000858
0.2656
F300000028
0.2123
F300000279
F300000741
0.1757
F300001294
0.3534
F300000115
0.1642
F300000193
0.3313
F300000767
0.2597
F300000008
F300000030
0.2497
F300000116
F300000450
0.1709
F300000094
0.3222
0.1495
F300000114
0.1758
F300000961
0.1517
0.2511
F300000969
0.3441
F300000669
0.1781
F300000445
F300000089
0.2149
F300000186
0.2744
0.4786
F300001156
0.2002
F300001379

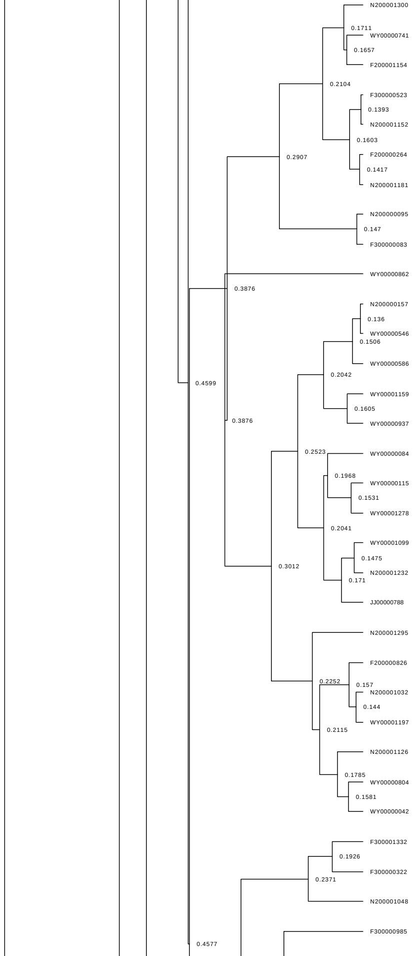
N200001300
0.1711
WY00000741
0.1657
F200001154
0.2104
F300000523
0.1393
N200001152
0.1603
F200000264
0.2907
0.1417
N200001181
N200000095
0.147
F300000083
WY00000862
0.3876
N200000157
0.136
WY00000546
0.1506
WY00000586
0.2042
0.4599
WY00001159
0.1605
0.3876
WY00000937
0.2523
WY00000084
0.1968
WY00000115
0.1531
WY00001278
0.2041
WY00001099
0.1475
0.3012
N200001232
0.171
JJ00000788
N200001295
F200000826
0.2252
0.157
N200001032
0.144
WY00001197
0.2115
N200001126
0.1785
WY00000804
0.1581
WY00000042
F300001332
0.1926
F300000322
0.2371
N200001048
F300000985
0.4577

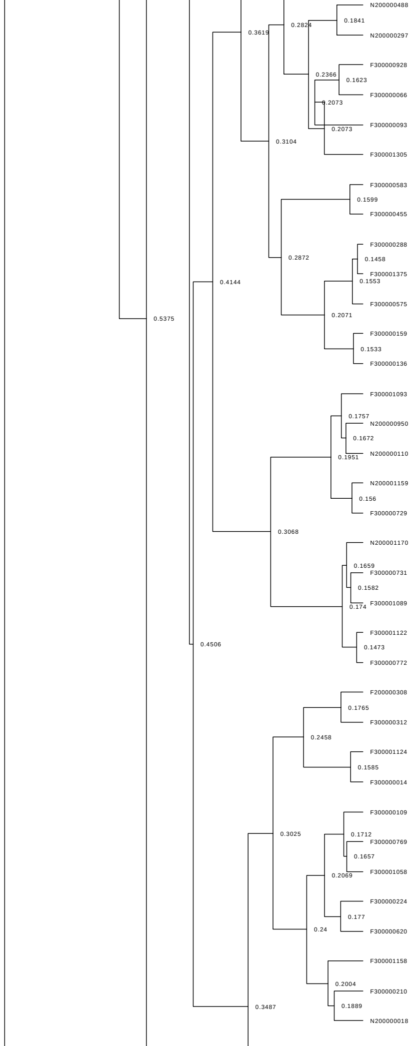
N200000488
0.1841
N200000297
0.2824
0.3619
F300000928
0.2366
0.1623
F300000066
0.2073
F300000093
0.2073
0.3104
F300001305
F300000583
0.1599
F300000455
F300000288
0.1458
0.2872
F300001375
0.1553
0.4144
F300000575
0.5375
0.2071
F300000159
0.1533
F300000136
F300001093
0.1757
N200000950
0.1672
N200000110
0.1951
N200001159
0.156
F300000729
0.3068
N200001170
0.1659
F300000731
0.1582
F300001089
0.174
F300001122
0.4506
0.1473
F300000772
F200000308
0.1765
F300000312
0.2458
F300001124
0.1585
F300000014
F300000109
0.3025
0.1712
F300000769
0.1657
F300001058
0.2069
F300000224
0.177
F300000620
0.24
F300001158
0.2004
F300000210
0.1889
0.3487
N200000018

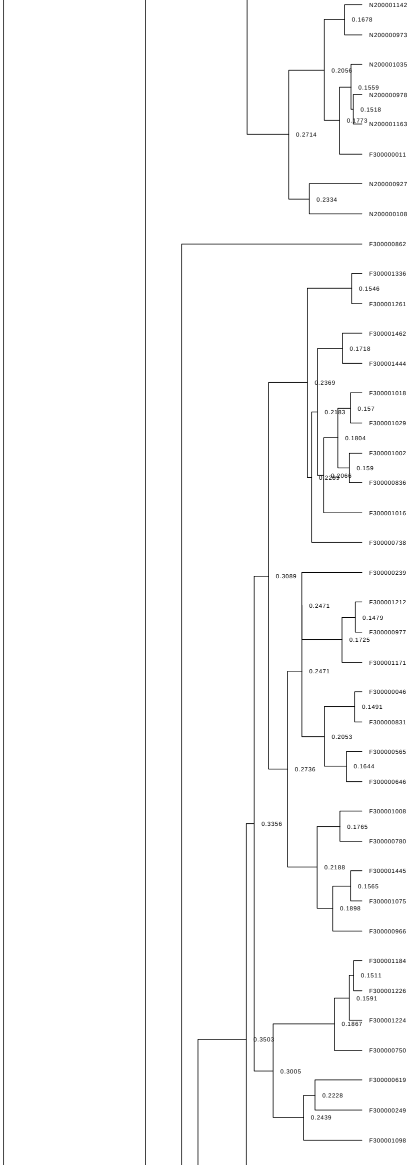
N200001142
0.1678
N200000973
N200001035
0.2056
0.1559
N200000978
0.1518
0.1773
N200001163
0.2714
F300000011
N200000927
0.2334
N200000108
F300000862
F300001336
0.1546
F300001261
F300001462
0.1718
F300001444
0.2369
F300001018
0.2183
0.157
F300001029
0.1804
F300001002
0.159
0.2066
0.2289
F300000836
F300001016
F300000738
F300000239
0.3089
0.2471
F300001212
0.1479
F300000977
0.1725
F300001171
0.2471
F300000046
0.1491
F300000831
0.2053
F300000565
0.1644
0.2736
F300000646
F300001008
0.3356
0.1765
F300000780
0.2188
F300001445
0.1565
F300001075
0.1898
F300000966
F300001184
0.1511
F300001226
0.1591
F300001224
0.1867
0.3503
F300000750
0.3005
F300000619
0.2228
F300000249
0.2439
F300001098

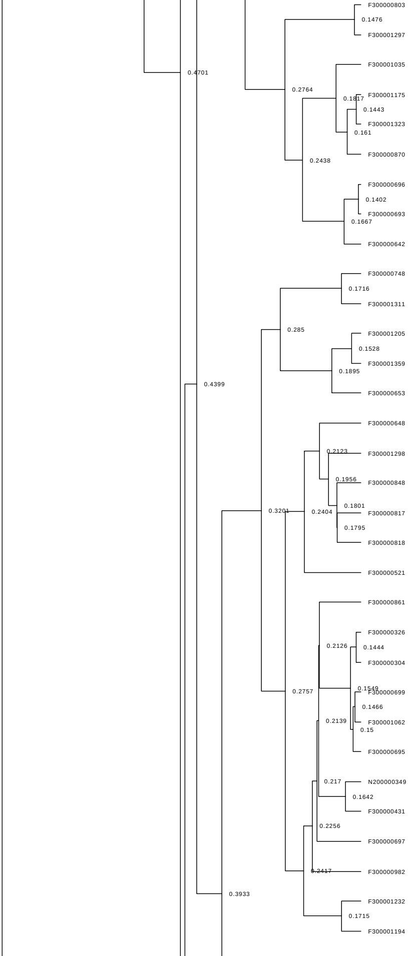

F300000803
0.1476
F300001297
0.4701
F300001035
0.2764
F300001175
0.1817
0.1443
F300001323
0.161
F300000870
0.2438
F300000696
0.1402
F300000693
0.1667
F300000642
F300000748
0.1716
F300001311
0.285
F300001205
0.1528
F300001359
0.1895
0.4399
F300000653
F300000648
0.2123
F300001298
0.1956
F300000848
0.1801
0.3201
0.2404
F300000817
0.1795
F300000818
F300000521
F300000861
F300000326
0.2126
0.1444
F300000304
0.1549
F300000699
0.2757
0.1466
0.2139
F300001062
0.15
F300000695
0.217
N200000349
0.1642
F300000431
0.2256
F300000697
0.2417
F300000982
0.3933
F300001232
0.1715
F300001194

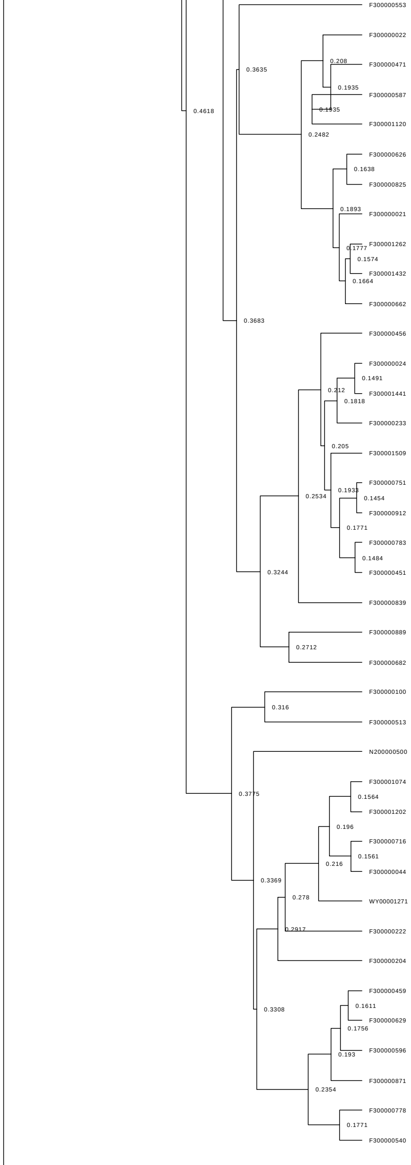
F300000553
0.3635
F300000022
0.208
F300000471
0.1935
F300000587
0.1935
0.4618
F300001120
0.2482
F300000626
0.1638
F300000825
0.1893
F300000021
F300001262
0.1777
0.1574
F300001432
0.1664
F300000662
0.3683
F300000456
F300000024
0.1491
F300001441
0.212
0.1818
F300000233
0.205
F300001509
F300000751
0.1933
0.1454
0.2534
F300000912
0.1771
F300000783
0.1484
F300000451
0.3244
F300000839
F300000889
0.2712
F300000682
F300000100
0.316
F300000513
N200000500
F300001074
0.3775
0.1564
F300001202
0.196
F300000716
0.1561
0.216
F300000044
0.3369
0.278
WY00001271
0.2917
F300000222
F300000204
F300000459
0.1611
0.3308
F300000629
0.1756
F300000596
0.193
F300000871
0.2354
F300000778
0.1771
F300000540

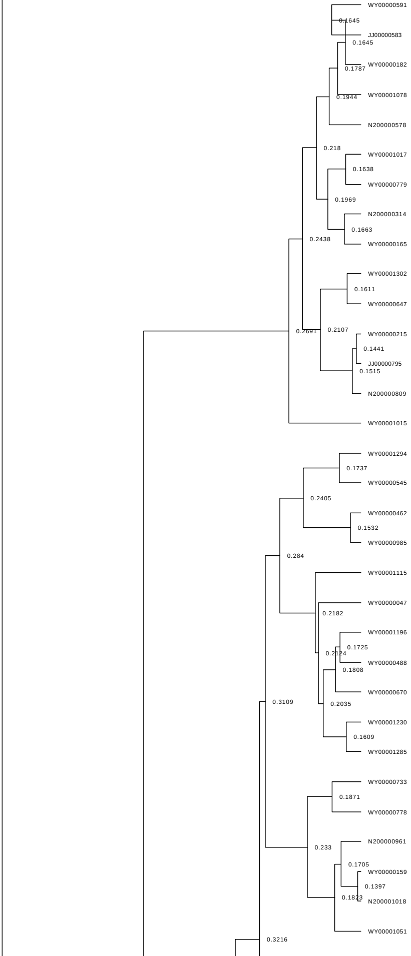

WY00000591
0.1645
JJ00000583
0.1645
WY00000182
0.1787
WY00001078
0.1944
N200000578
0.218
WY00001017
0.1638
WY00000779
0.1969
N200000314
0.1663
WY00000165
0.2438
WY00001302
0.1611
WY00000647
0.2691
0.2107
WY00000215
0.1441
JJ00000795
0.1515
N200000809
WY00001015
WY00001294
0.1737
WY00000545
0.2405
WY00000462
0.1532
WY00000985
0.284
WY00001115
WY00000047
0.2182
WY00001196
0.1725
0.2124
WY00000488
0.1808
WY00000670
0.3109
0.2035
WY00001230
0.1609
WY00001285
WY00000733
0.1871
WY00000778
N200000961
0.233
0.1705
WY00000159
0.1397
0.1823
N200001018
WY00001051
0.3216

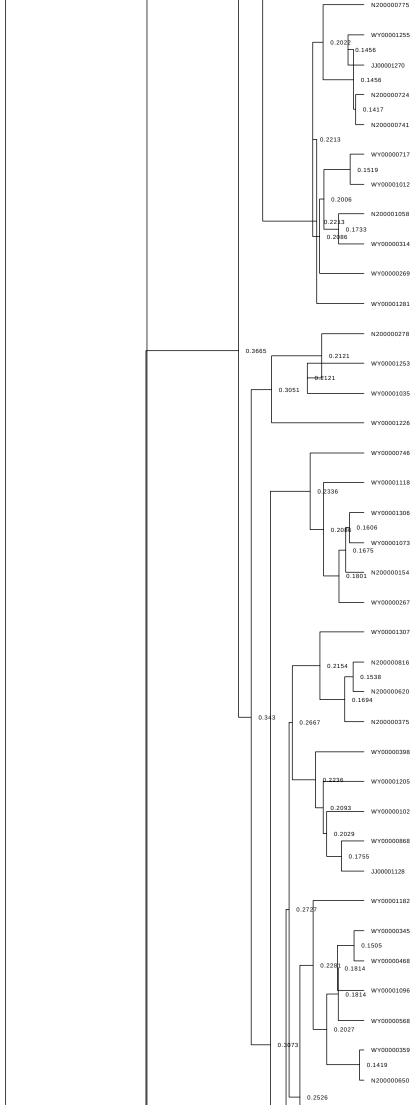
N200000775
WY00001255
0.2022
0.1456
JJ00001270
0.1456
N200000724
0.1417
N200000741
0.2213
WY00000717
0.1519
WY00001012
0.2006
N200001058
0.2213
0.1733
0.2086
WY00000314
WY00000269
WY00001281
N200000278
0.3665
0.2121
WY00001253
0.2121
0.3051
WY00001035
WY00001226
WY00000746
WY00001118
0.2336
WY00001306
0.1606
0.2086
WY00001073
0.1675
N200000154
0.1801
WY00000267
WY00001307
0.2154
N200000816
0.1538
N200000620
0.1694
0.343
0.2667
N200000375
WY00000398
0.2236
WY00001205
0.2093
WY00000102
0.2029
WY00000868
0.1755
JJ00001128
WY00001182
0.2727
WY00000345
0.1505
WY00000468
0.2281
0.1814
WY00001096
0.1814
WY00000568
0.2027
0.3073
WY00000359
0.1419
N200000650
0.2526

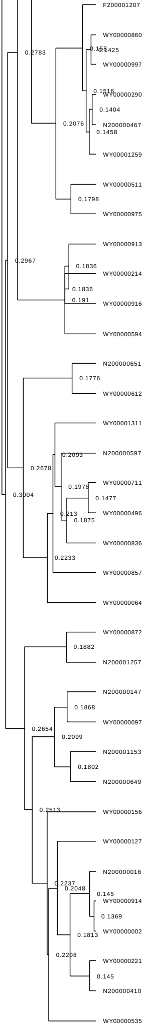
F200001207
WY00000860
0.158
0.1425
WY00000997
0.2783
0.1516
WY00000290
0.1404
N200000467
0.2076
0.1458
WY00001259
WY00000511
0.1798
WY00000975
WY00000913
0.2967
0.1836
WY00000214
0.1836
0.191
WY00000916
WY00000594
N200000651
0.1776
WY00000612
WY00001311
N200000597
0.2093
0.2678
WY00000711
0.1978
0.3004
0.1477
WY00000496
0.213
0.1875
WY00000836
0.2233
WY00000857
WY00000064
WY00000872
0.1882
N200001257
N200000147
0.1868
WY00000097
0.2654
0.2099
N200001153
0.1802
N200000649
0.2513
WY00000156
WY00000127
N200000016
0.2237
0.2048
0.145
WY00000914
0.1369
WY00000002
0.1813
0.2208
WY00000221
0.145
N200000410
WY00000535

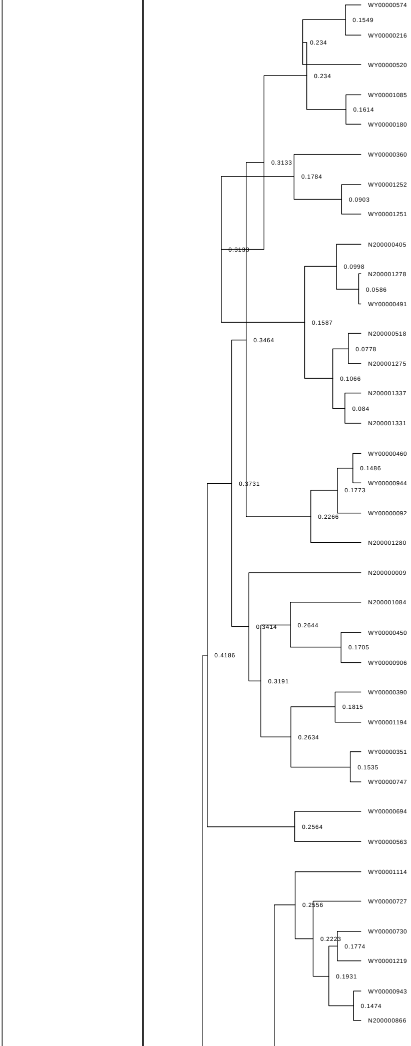
WY00000574
0.1549
WY00000216
0.234
WY00000520
0.234
WY00001085
0.1614
WY00000180
WY00000360
0.3133
0.1784
WY00001252
0.0903
WY00001251
N200000405
0.3133
0.0998
N200001278
0.0586
WY00000491
0.1587
N200000518
0.3464
0.0778
N200001275
0.1066
N200001337
0.084
N200001331
WY00000460
0.1486
WY00000944
0.3731
0.1773
WY00000092
0.2266
N200001280
N200000009
N200001084
0.2644
0.3414
WY00000450
0.1705
0.4186
WY00000906
0.3191
WY00000390
0.1815
WY00001194
0.2634
WY00000351
0.1535
WY00000747
WY00000694
0.2564
WY00000563
WY00001114
WY00000727
0.2556
WY00000730
0.2223
0.1774
WY00001219
0.1931
WY00000943
0.1474
N200000866

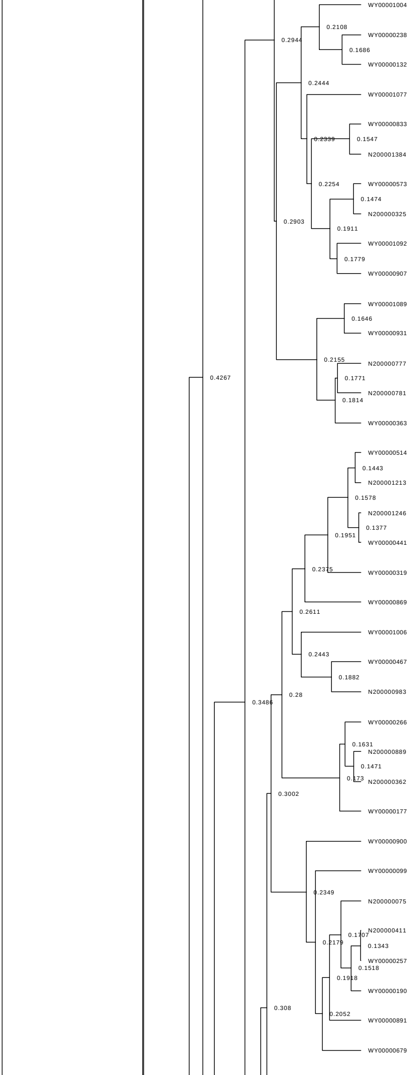

WY00001004
0.2108
WY00000238
0.2944
0.1686
WY00000132
0.2444
WY00001077
WY00000833
0.2339
0.1547
N200001384
0.2254
WY00000573
0.1474
N200000325
0.2903
0.1911
WY00001092
0.1779
WY00000907
WY00001089
0.1646
WY00000931
0.2155
N200000777
0.4267
0.1771
N200000781
0.1814
WY00000363
WY00000514
0.1443
N200001213
0.1578
N200001246
0.1377
0.1951
WY00000441
0.2375
WY00000319
WY00000869
0.2611
WY00001006
0.2443
WY00000467
0.1882
N200000983
0.28
0.3486
WY00000266
0.1631
N200000889
0.1471
0.173
N200000362
0.3002
WY00000177
WY00000900
WY00000099
0.2349
N200000075
N200000411
0.1707
0.2179
0.1343
WY00000257
0.1518
0.1918
WY00000190
0.308
0.2052
WY00000891
WY00000679

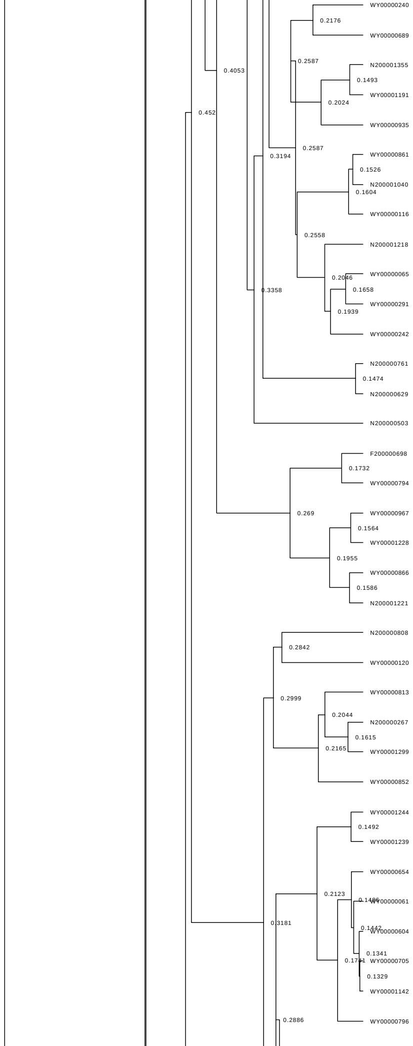

WY00000240
0.2176
WY00000689
0.2587
N200001355
0.1493
WY00001191
0.2024
WY00000935
0.4053
0.452
0.2587
0.3194
WY00000861
0.1526
N200001040
0.1604
WY00000116
0.2558
N200001218
WY00000065
0.2046
0.1658
0.3358
WY00000291
0.1939
WY00000242
N200000761
0.1474
N200000629
N200000503
F200000698
0.1732
WY00000794
0.269
WY00000967
0.1564
WY00001228
0.1955
WY00000866
0.1586
N200001221
N200000808
0.2842
WY00000120
0.2999
WY00000813
0.2044
N200000267
0.1615
0.2165
WY00001299
WY00000852
WY00001244
0.1492
WY00001239
WY00000654
0.2123
WY00000061
0.3181
0.1442
WY00000604
0.1341
WY00000705
0.1329
WY00001142
0.2886
WY00000796

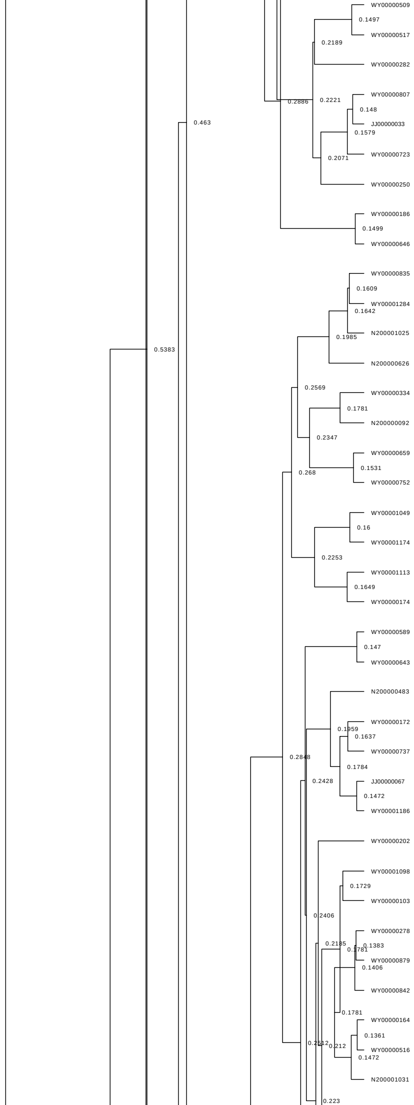
WY00000509
0.1497
WY00000517
0.2189
WY00000282
WY00000807
0.2886
0.2221
0.148
JJ00000033
0.463
0.1579
WY00000723
0.2071
WY00000250
WY00000186
0.1499
WY00000646
WY00000835
0.1609
WY00001284
0.1642
N200001025
0.1985
0.5383
N200000626
0.2569
WY00000334
0.1781
N200000092
0.2347
WY00000659
0.1531
0.268
WY00000752
WY00001049
0.16
WY00001174
0.2253
WY00001113
0.1649
WY00000174
WY00000589
0.147
WY00000643
N200000483
WY00000172
0.1959
0.1637
WY00000737
0.2848
0.1784
JJ00000067
0.2428
0.1472
WY00001186
WY00000202
WY00001098
0.1729
WY00000103
0.2406
WY00000278
0.2135
0.1383
0.1781
WY00000879
0.1406
WY00000842
0.1781
WY00000164
0.1361
0.2512
0.212
WY00000516
0.1472
N200001031
0.223

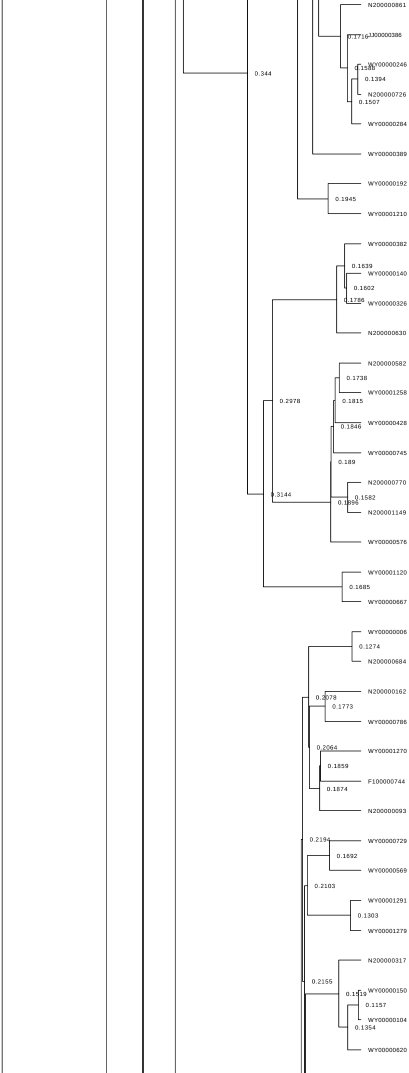
N200000861
0.1716
JJ00000386
WY00000246
0.1588
0.1394
N200000726
0.1507
WY00000284
0.344
WY00000389
WY00000192
0.1945
WY00001210
WY00000382
0.1639
WY00000140
0.1602
0.1786
WY00000326
N200000630
N200000582
0.1738
WY00001258
0.2978
0.1815
WY00000428
0.1846
WY00000745
0.189
N200000770
0.3144
0.1582
0.1896
N200001149
WY00000576
WY00001120
0.1685
WY00000667
WY00000006
0.1274
N200000684
N200000162
0.2078
0.1773
WY00000786
0.2064
WY00001270
0.1859
F100000744
0.1874
N200000093
0.2194
WY00000729
0.1692
WY00000569
0.2103
WY00001291
0.1303
WY00001279
N200000317
0.2155
WY00000150
0.1519
0.1157
WY00000104
0.1354
WY00000620

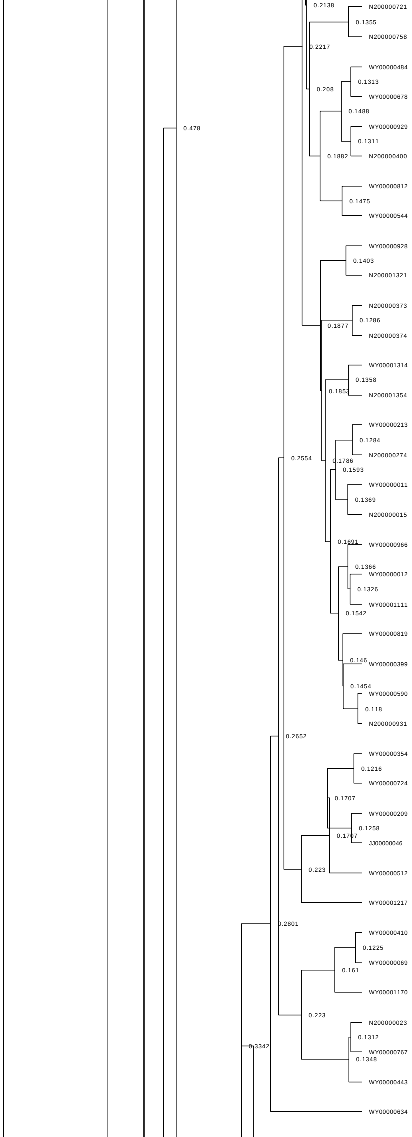
0.2138
N200000721
0.1355
N200000758
0.2217
WY00000484
0.1313
0.208
WY00000678
0.1488
WY00000929
0.478
0.1311
0.1882
N200000400
WY00000812
0.1475
WY00000544
WY00000928
0.1403
N200001321
N200000373
0.1286
0.1877
N200000374
WY00001314
0.1358
0.1853
N200001354
WY00000213
0.1284
N200000274
0.2554
0.1786
0.1593
WY00000011
0.1369
N200000015
0.1691
WY00000966
0.1366
WY00000012
0.1326
WY00001111
0.1542
WY00000819
0.146
WY00000399
0.1454
WY00000590
0.118
N200000931
0.2652
WY00000354
0.1216
WY00000724
0.1707
WY00000209
0.1258
0.1707
JJ00000046
0.223
WY00000512
WY00001217
0.2801
WY00000410
0.1225
WY00000069
0.161
WY00001170
0.223
N200000023
0.1312
WY00000767
0.1348
WY00000443
0.3342
WY00000634

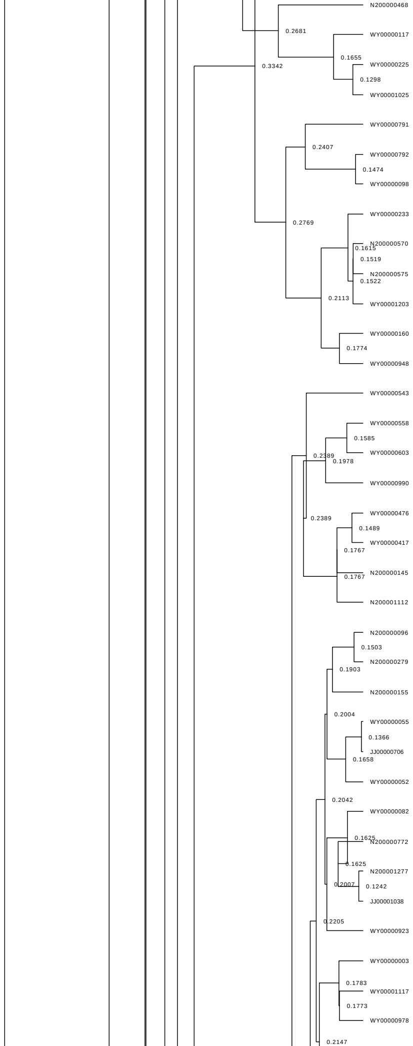
N200000468
0.2681
WY00000117
0.1655
WY00000225
0.3342
0.1298
WY00001025
WY00000791
0.2407
WY00000792
0.1474
WY00000098
WY00000233
0.2769
N200000570
0.1615
0.1519
N200000575
0.1522
0.2113
WY00001203
WY00000160
0.1774
WY00000948
WY00000543
WY00000558
0.1585
WY00000603
0.2389
0.1978
WY00000990
WY00000476
0.2389
0.1489
WY00000417
0.1767
N200000145
0.1767
N200001112
N200000096
0.1503
N200000279
0.1903
N200000155
0.2004
WY00000055
0.1366
JJ00000706
0.1658
WY00000052
0.2042
WY00000082
0.1625
N200000772
0.1625
N200001277
0.2007
0.1242
JJ00001038
0.2205
WY00000923
WY00000003
0.1783
WY00001117
0.1773
WY00000978
0.2147

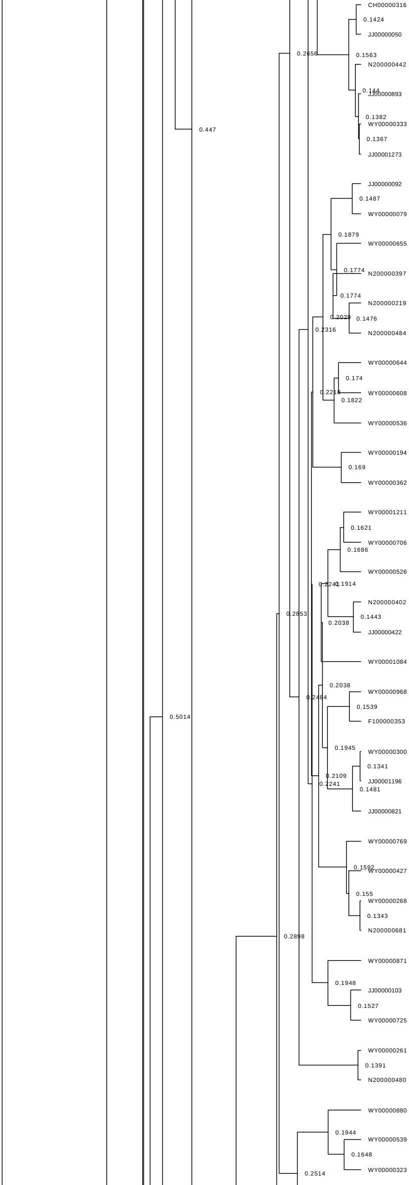

CH00000316
0.1424
JJ00000050
0.2656
0.1563
N200000442
0.144
JJ00000893
0.1382
WY00000333
0.1367
JJ00001273
0.447
JJ00000092
0.1487
WY00000079
0.1879
WY00000655
0.1774
N200000397
0.1774
N200000219
0.2029
0.1476
0.2316
N200000484
WY00000644
0.174
0.2218
WY00000608
0.1822
WY00000536
WY00000194
0.169
WY00000362
WY00001211
0.1621
WY00000706
0.1686
WY00000526
0.2241
0.1914
N200000402
0.2853
0.1443
0.2038
JJ00000422
WY00001084
0.2038
WY00000968
0.2484
0.1539
0.5014
F100000353
0.1945
WY00000300
0.1341
0.2109
JJ00001196
0.2241
0.1481
JJ00000821
WY00000769
0.1592
WY00000427
0.155
WY00000268
0.1343
N200000681
0.2898
WY00000871
0.1948
JJ00000103
0.1527
WY00000725
WY00000261
0.1391
N200000480
WY00000880
0.1944
WY00000539
0.1648
WY00000323
0.2514

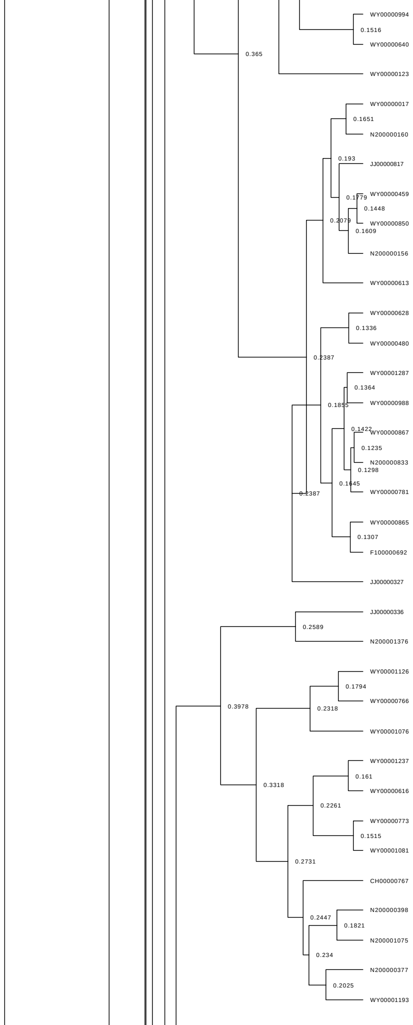
WY00000994
0.1516
WY00000640
0.365
WY00000123
WY00000017
0.1651
N200000160
0.193
JJ00000817
WY00000459
0.1779
0.1448
WY00000850
0.2079
0.1609
N200000156
WY00000613
WY00000628
0.1336
WY00000480
0.2387
WY00001287
0.1364
WY00000988
0.1855
0.1422
WY00000867
0.1235
N200000833
0.1298
0.1645
0.2387
WY00000781
WY00000865
0.1307
F100000692
JJ00000327
JJ00000336
0.2589
N200001376
WY00001126
0.1794
WY00000766
0.3978
0.2318
WY00001076
WY00001237
0.161
WY00000616
0.3318
0.2261
WY00000773
0.1515
WY00001081
0.2731
CH00000767
N200000398
0.2447
0.1821
N200001075
0.234
N200000377
0.2025
WY00001193

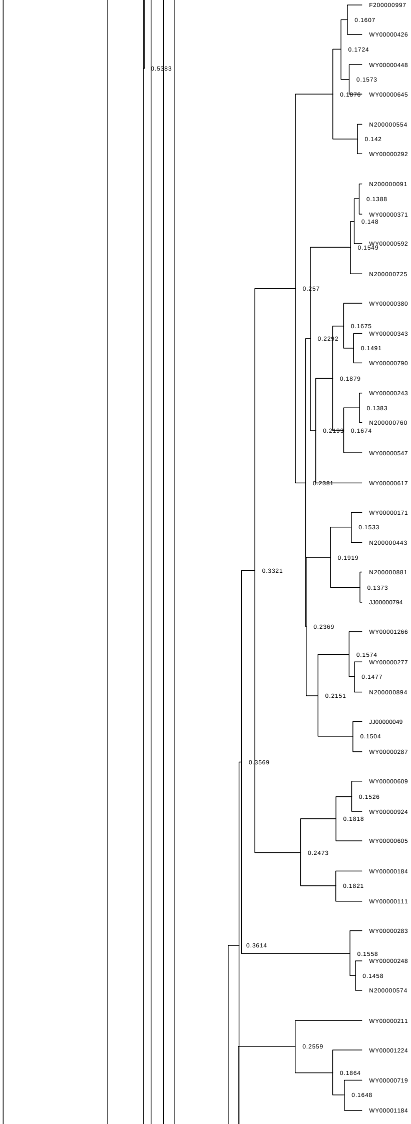
F200000997
0.5383
0.1607
WY00000426
0.1724
WY00000448
0.1573
0.1876
WY00000645
N200000554
0.142
WY00000292
N200000091
0.1388
WY00000371
0.148
WY00000592
0.1549
N200000725
0.257
WY00000380
0.1675
WY00000343
0.2292
0.1491
WY00000790
0.1879
WY00000243
0.1383
N200000760
0.2193
0.1674
WY00000547
0.2381
WY00000617
WY00000171
0.1533
N200000443
0.1919
N200000881
0.3321
0.1373
JJ00000794
0.2369
WY00001266
0.1574
WY00000277
0.1477
N200000894
0.2151
JJ00000049
0.1504
WY00000287
0.3569
WY00000609
0.1526
WY00000924
0.1818
WY00000605
0.2473
WY00000184
0.1821
WY00000111
WY00000283
0.3614
0.1558
WY00000248
0.1458
N200000574
WY00000211
0.2559
WY00001224
0.1864
WY00000719
0.1648
WY00001184

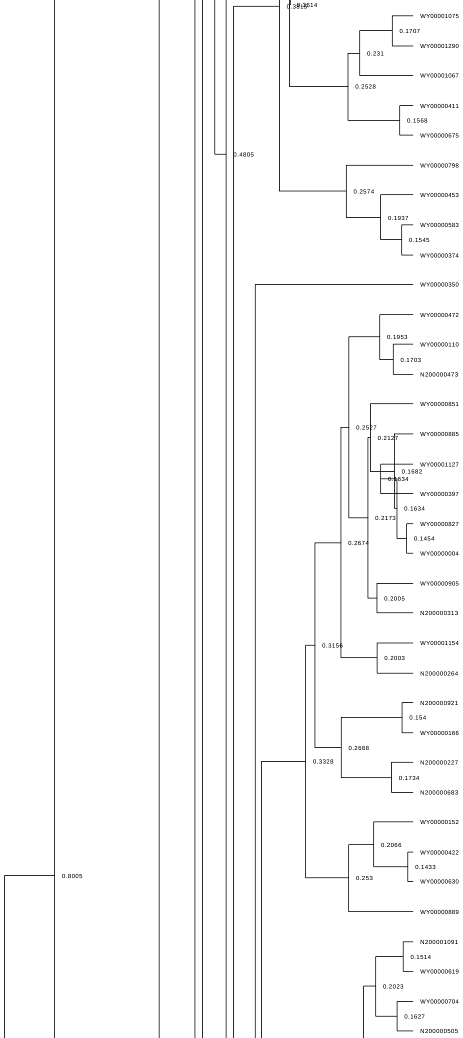
0.3815
0.3614
WY00001075
0.1707
WY00001290
0.231
WY00001067
0.2528
WY00000411
0.1568
WY00000675
0.4805
WY00000798
0.2574
WY00000453
0.1937
WY00000583
0.1545
WY00000374
WY00000350
WY00000472
0.1953
WY00000110
0.1703
N200000473
WY00000851
0.2527
0.2127
WY00000885
WY00001127
0.1682
0.1634
WY00000397
0.1634
0.2173
WY00000827
0.1454
0.2674
WY00000004
WY00000905
0.2005
N200000313
WY00001154
0.3156
0.2003
N200000264
N200000921
0.154
WY00000166
0.2668
0.3328
N200000227
0.1734
N200000683
WY00000152
0.2066
WY00000422
0.1433
0.8005
0.253
WY00000630
WY00000889
N200001091
0.1514
WY00000619
0.2023
WY00000704
0.1627
N200000505

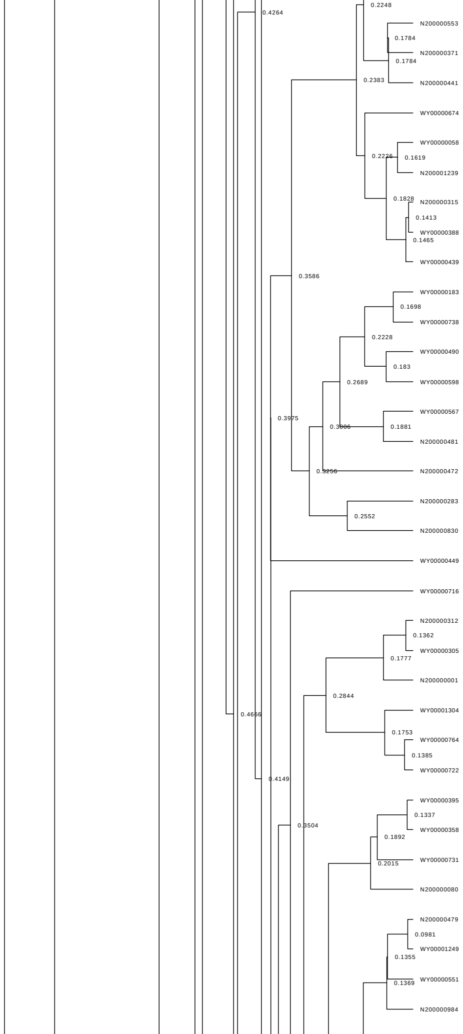
0.2248
0.4264
N200000553
0.1784
N200000371
0.1784
0.2383
N200000441
WY00000674
WY00000058
0.2226
0.1619
N200001239
0.1828
N200000315
0.1413
WY00000388
0.1465
WY00000439
0.3586
WY00000183
0.1698
WY00000738
0.2228
WY00000490
0.183
0.2689
WY00000598
WY00000567
0.3975
0.3006
0.1881
N200000481
0.3256
N200000472
N200000283
0.2552
N200000830
WY00000449
WY00000716
N200000312
0.1362
WY00000305
0.1777
N200000001
0.2844
WY00001304
0.4666
0.1753
WY00000764
0.1385
WY00000722
0.4149
WY00000395
0.1337
0.3504
WY00000358
0.1892
WY00000731
0.2015
N200000080
N200000479
0.0981
WY00001249
0.1355
WY00000551
0.1369
N200000984

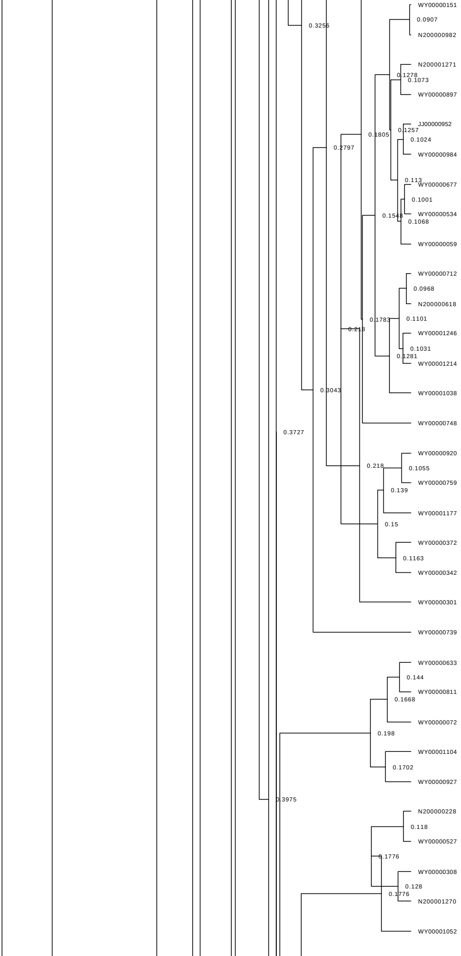

WY00000151
0.0907
N200000982
0.3256
N200001271
0.1278
0.1073
WY00000897
JJ00000952
0.1257
0.1805
0.1024
0.2797
WY00000984
0.113
WY00000677
0.1001
WY00000534
0.1548
0.1068
WY00000059
WY00000712
0.0968
N200000618
0.1101
0.1783
0.218
WY00001246
0.1031
0.1281
WY00001214
WY00001038
0.3043
WY00000748
0.3727
WY00000920
0.218
0.1055
WY00000759
0.139
WY00001177
0.15
WY00000372
0.1163
WY00000342
WY00000301
WY00000739
WY00000633
0.144
WY00000811
0.1668
WY00000072
0.198
WY00001104
0.1702
WY00000927
0.3975
N200000228
0.118
WY00000527
0.1776
WY00000308
0.128
0.1776
N200001270
WY00001052

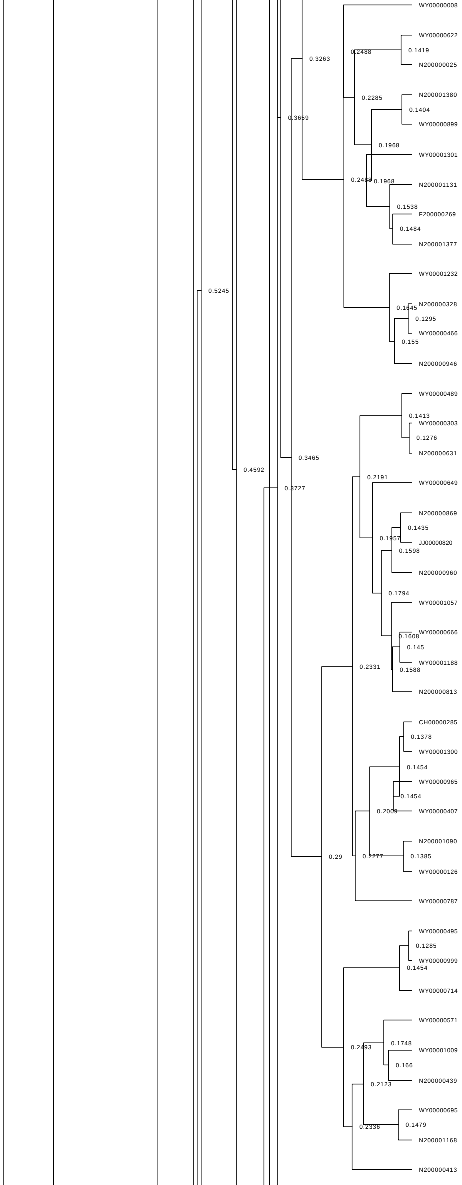
WY00000008
WY00000622
0.2488
0.1419
0.3263
N200000025
N200001380
0.2285
0.1404
0.3659
WY00000899
0.1968
WY00001301
0.2488
0.1968
N200001131
0.1538
F200000269
0.1484
N200001377
WY00001232
0.5245
N200000328
0.1645
0.1295
WY00000466
0.155
N200000946
WY00000489
0.1413
WY00000303
0.1276
N200000631
0.3465
0.4592
0.2191
WY00000649
0.3727
N200000869
0.1435
0.1957
JJ00000820
0.1598
N200000960
0.1794
WY00001057
WY00000666
0.1608
0.145
WY00001188
0.2331
0.1588
N200000813
CH00000285
0.1378
WY00001300
0.1454
WY00000965
0.1454
0.2009
WY00000407
N200001090
0.29
0.2277
0.1385
WY00000126
WY00000787
WY00000495
0.1285
WY00000999
0.1454
WY00000714
WY00000571
0.1748
0.2493
WY00001009
0.166
N200000439
0.2123
WY00000695
0.1479
0.2336
N200001168
N200000413

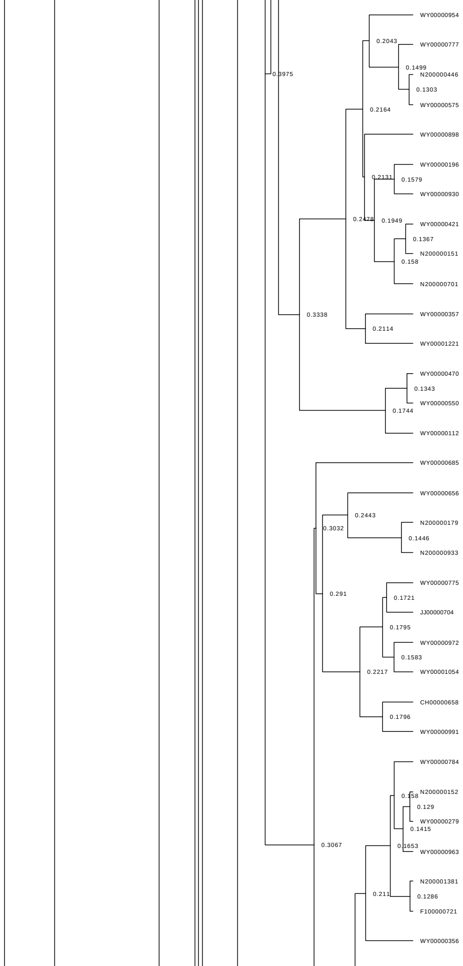
WY00000954
0.2043
WY00000777
0.1499
N200000446
0.3975
0.1303
WY00000575
0.2164
WY00000898
WY00000196
0.2131
0.1579
WY00000930
0.2478
0.1949
WY00000421
0.1367
N200000151
0.158
N200000701
WY00000357
0.3338
0.2114
WY00001221
WY00000470
0.1343
WY00000550
0.1744
WY00000112
WY00000685
WY00000656
0.2443
0.3032
N200000179
0.1446
N200000933
WY00000775
0.291
0.1721
JJ00000704
0.1795
WY00000972
0.1583
0.2217
WY00001054
CH00000658
0.1796
WY00000991
WY00000784
0.158
N200000152
0.129
WY00000279
0.1415
0.3067
0.1653
WY00000963
N200001381
0.211
0.1286
F100000721
WY00000356

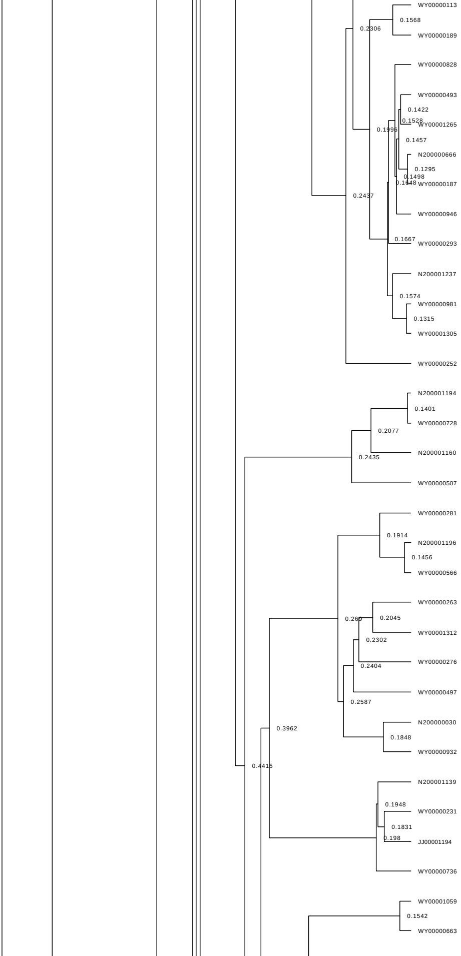
WY00000113
0.1568
0.2306
WY00000189
WY00000828
WY00000493
0.1422
0.1528
WY00001265
0.1996
0.1457
N200000666
0.1295
0.1498
0.1648
WY00000187
0.2437
WY00000946
0.1667
WY00000293
N200001237
0.1574
WY00000981
0.1315
WY00001305
WY00000252
N200001194
0.1401
WY00000728
0.2077
N200001160
0.2435
WY00000507
WY00000281
0.1914
N200001196
0.1456
WY00000566
WY00000263
0.269
0.2045
WY00001312
0.2302
WY00000276
0.2404
WY00000497
0.2587
N200000030
0.3962
0.1848
WY00000932
0.4415
N200001139
0.1948
WY00000231
0.1831
0.198
JJ00001194
WY00000736
WY00001059
0.1542
WY00000663

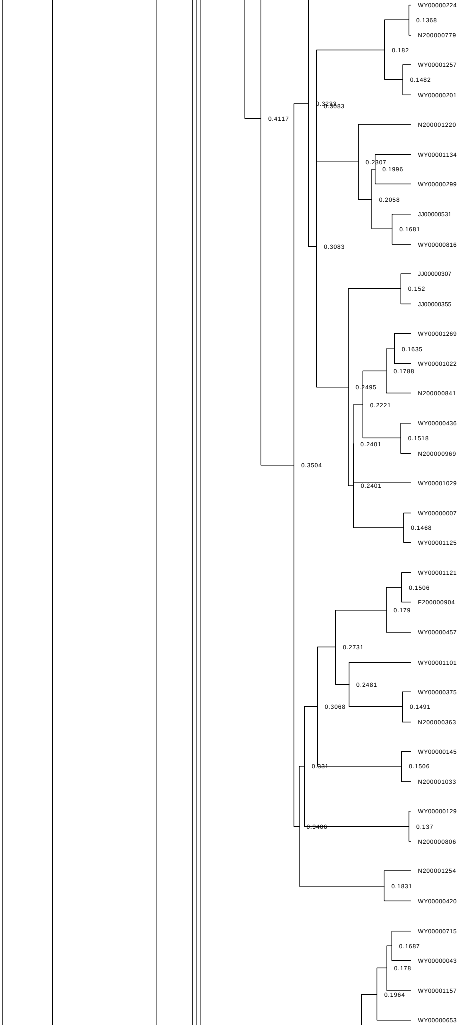
WY00000224
0.1368
N200000779
0.182
WY00001257
0.1482
WY00000201
0.3233
0.3083
0.4117
N200001220
WY00001134
0.2307
0.1996
WY00000299
0.2058
JJ00000531
0.1681
WY00000816
0.3083
JJ00000307
0.152
JJ00000355
WY00001269
0.1635
WY00001022
0.1788
0.2495
N200000841
0.2221
WY00000436
0.1518
0.2401
N200000969
0.3504
WY00001029
0.2401
WY00000007
0.1468
WY00001125
WY00001121
0.1506
F200000904
0.179
WY00000457
0.2731
WY00001101
0.2481
WY00000375
0.3068
0.1491
N200000363
WY00000145
0.331
0.1506
N200001033
WY00000129
0.137
0.3406
N200000806
N200001254
0.1831
WY00000420
WY00000715
0.1687
WY00000043
0.178
WY00001157
0.1964
WY00000653

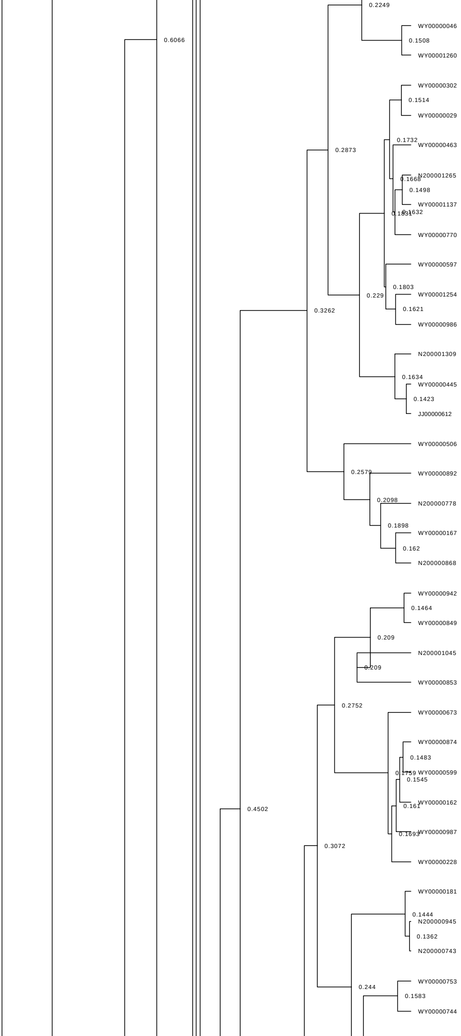

0.6066
0.2249
WY00000046
0.1508
WY00001260
WY00000302
0.1514
WY00000029
0.1732
WY00000463
0.2873
N200001265
0.1668
0.1498
WY00001137
0.1632
WY00000770
WY00000597
0.1803
WY00001254
0.229
0.1621
0.3262
WY00000986
N200001309
0.1634
WY00000445
0.1423
JJ00000612
WY00000506
0.2579
WY00000892
0.2098
N200000778
0.1898
WY00000167
0.162
N200000868
WY00000942
0.1464
WY00000849
0.209
N200001045
0.209
WY00000853
0.2752
WY00000673
WY00000874
0.1483
0.1759
WY00000599
0.1545
WY00000162
0.161
0.4502
WY00000987
0.1693
0.3072
WY00000228
WY00000181
0.1444
N200000945
0.1362
N200000743
WY00000753
0.244
0.1583
WY00000744

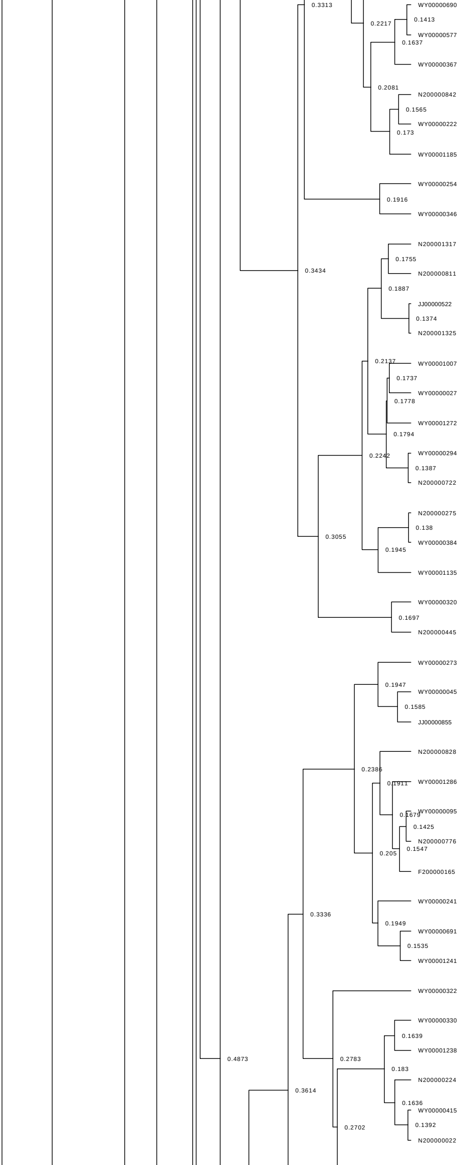
WY00000690
0.3313
0.1413
0.2217
WY00000577
0.1637
WY00000367
0.2081
N200000842
0.1565
WY00000222
0.173
WY00001185
WY00000254
0.1916
WY00000346
N200001317
0.1755
0.3434
N200000811
0.1887
JJ00000522
0.1374
N200001325
0.2137
WY00001007
0.1737
WY00000027
0.1778
WY00001272
0.1794
WY00000294
0.2242
0.1387
N200000722
N200000275
0.138
0.3055
WY00000384
0.1945
WY00001135
WY00000320
0.1697
N200000445
WY00000273
0.1947
WY00000045
0.1585
JJ00000855
N200000828
0.2386
WY00001286
0.1911
WY00000095
0.1679
0.1425
N200000776
0.1547
0.205
F200000165
WY00000241
0.3336
0.1949
WY00000691
0.1535
WY00001241
WY00000322
WY00000330
0.1639
WY00001238
0.4873
0.2783
0.183
N200000224
0.3614
0.1636
WY00000415
0.1392
0.2702
N200000022

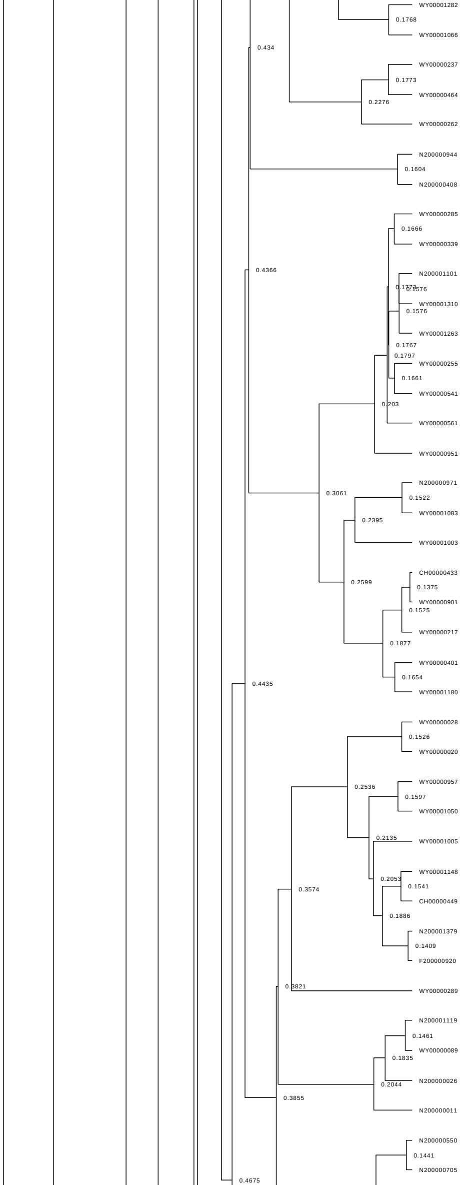

WY00001282
0.1768
WY00001066
0.434
WY00000237
0.1773
WY00000464
0.2276
WY00000262
N200000944
0.1604
N200000408
WY00000285
0.1666
WY00000339
0.4366
N200001101
0.1773
0.1576
WY00001310
0.1576
WY00001263
0.1767
0.1797
WY00000255
0.1661
WY00000541
0.203
WY00000561
WY00000951
N200000971
0.1522
0.3061
WY00001083
0.2395
WY00001003
CH00000433
0.1375
0.2599
WY00000901
0.1525
WY00000217
0.1877
WY00000401
0.1654
0.4435
WY00001180
WY00000028
0.1526
WY00000020
WY00000957
0.2536
0.1597
WY00001050
0.2135
WY00001005
WY00001148
0.2053
0.1541
0.3574
CH00000449
0.1886
N200001379
0.1409
F200000920
0.3821
WY00000289
N200001119
0.1461
WY00000089
0.1835
N200000026
0.2044
0.3855
N200000011
N200000550
0.1441
N200000705
0.4675

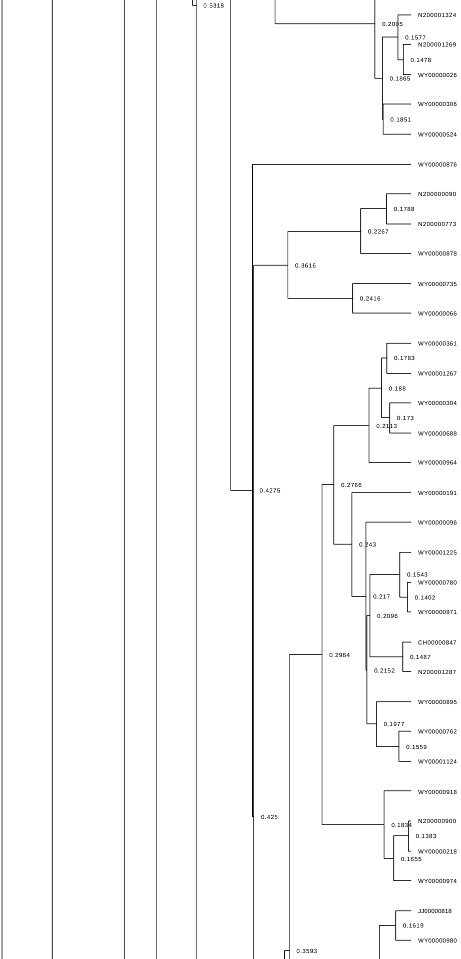

0.5318
N200001324
0.2005
0.1577
N200001269
0.1478
WY00000026
0.1865
WY00000306
0.1851
WY00000524
WY00000876
N200000090
0.1788
N200000773
0.2267
WY00000878
0.3616
WY00000735
0.2416
WY00000066
WY00000361
0.1783
WY00001267
0.188
WY00000304
0.173
0.2113
WY00000688
WY00000964
0.2766
0.4275
WY00000191
WY00000096
0.243
WY00001225
0.1543
WY00000780
0.217
0.1402
WY00000971
0.2096
CH00000847
0.2984
0.1487
0.2152
N200001287
WY00000895
0.1977
WY00000762
0.1559
WY00001124
WY00000918
0.425
N200000900
0.1834
0.1383
WY00000218
0.1655
WY00000974
JJ00000818
0.1619
WY00000980
0.3593

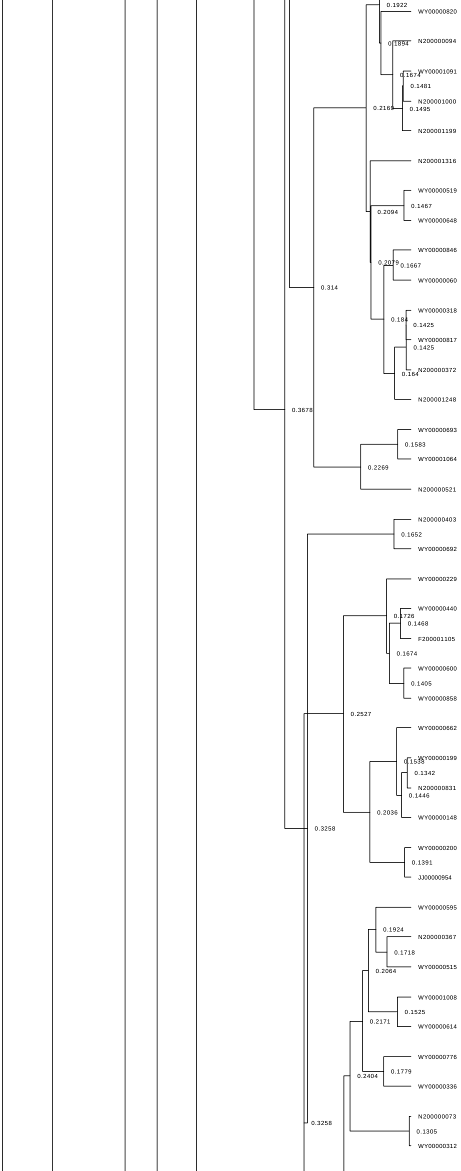

0.1922
WY00000820
0.1894
N200000094
WY00001091
0.1674
0.1481
N200001000
0.2169
0.1495
N200001199
N200001316
WY00000519
0.1467
0.2094
WY00000648
WY00000846
0.2079
0.1667
WY00000060
0.314
WY00000318
0.184
0.1425
WY00000817
0.1425
N200000372
0.164
N200001248
0.3678
WY00000693
0.1583
WY00001064
0.2269
N200000521
N200000403
0.1652
WY00000692
WY00000229
WY00000440
0.1726
0.1468
F200001105
0.1674
WY00000600
0.1405
WY00000858
0.2527
WY00000662
WY00000199
0.1538
0.1342
N200000831
0.1446
0.2036
WY00000148
0.3258
WY00000200
0.1391
JJ00000954
WY00000595
0.1924
N200000367
0.1718
WY00000515
0.2064
WY00001008
0.1525
0.2171
WY00000614
WY00000776
0.1779
0.2404
WY00000336
N200000073
0.3258
0.1305
WY00000312

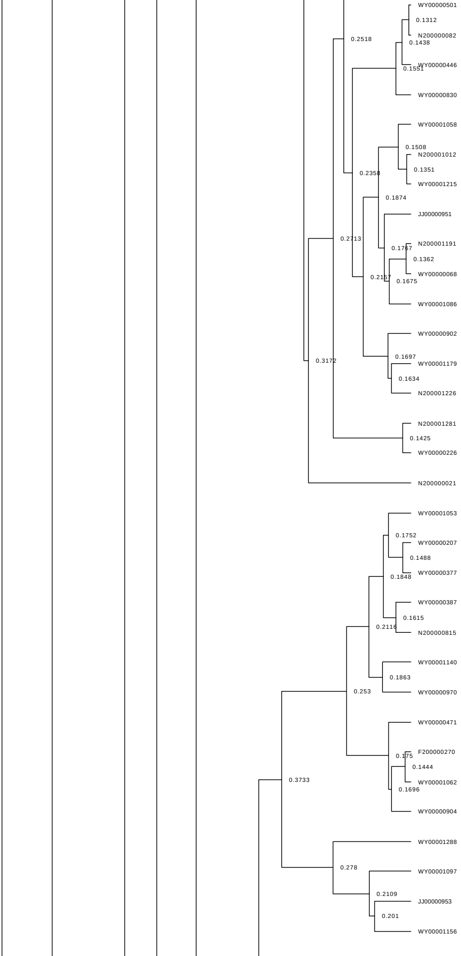

WY00000501
0.1312
N200000082
0.1438
0.2518
WY00000446
0.1551
WY00000830
WY00001058
0.1508
N200001012
0.1351
0.2358
WY00001215
0.1874
JJ00000951
0.2713
N200001191
0.1767
0.1362
WY00000068
0.2157
0.1675
WY00001086
WY00000902
0.1697
WY00001179
0.3172
0.1634
N200001226
N200001281
0.1425
WY00000226
N200000021
WY00001053
0.1752
WY00000207
0.1488
WY00000377
0.1848
WY00000387
0.1615
0.2116
N200000815
WY00001140
0.1863
0.253
WY00000970
WY00000471
F200000270
0.175
0.1444
0.3733
WY00001062
0.1696
WY00000904
WY00001288
0.278
WY00001097
0.2109
JJ00000953
0.201
WY00001156

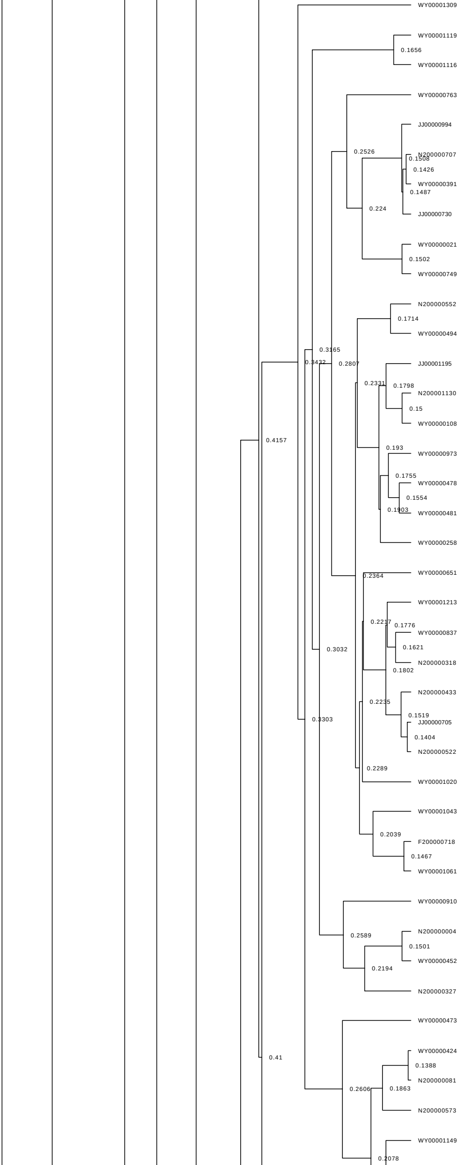

WY00001309
WY00001119
0.1656
WY00001116
WY00000763
JJ00000994
0.2526
N200000707
0.1508
0.1426
WY00000391
0.1487
0.224
JJ00000730
WY00000021
0.1502
WY00000749
N200000552
0.1714
WY00000494
0.3165
0.3432
0.2807
JJ00001195
0.2331
0.1798
N200001130
0.15
WY00000108
0.4157
0.193
WY00000973
0.1755
WY00000478
0.1554
0.1903
WY00000481
WY00000258
WY00000651
0.2364
WY00001213
0.2217
0.1776
WY00000837
0.1621
0.3032
N200000318
0.1802
N200000433
0.2235
0.1519
JJ00000705
0.3303
0.1404
N200000522
0.2289
WY00001020
WY00001043
0.2039
F200000718
0.1467
WY00001061
WY00000910
N200000004
0.2589
0.1501
WY00000452
0.2194
N200000327
WY00000473
WY00000424
0.41
0.1388
N200000081
0.2606
0.1863
N200000573
WY00001149
0.2078

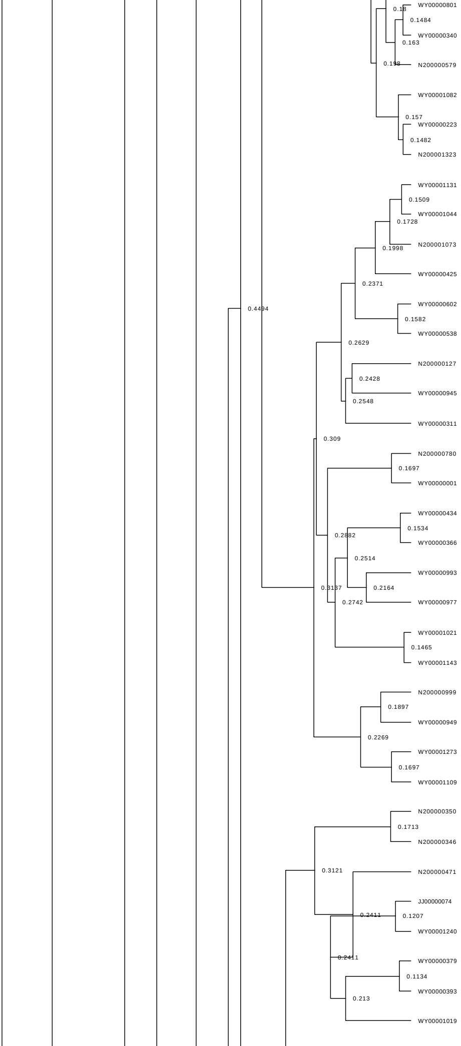
WY00000801
0.18
0.1484
WY00000340
0.163
N200000579
0.198
WY00001082
0.157
WY00000223
0.1482
N200001323
WY00001131
0.1509
WY00001044
0.1728
N200001073
0.1998
WY00000425
0.2371
0.4494
WY00000602
0.1582
WY00000538
0.2629
N200000127
0.2428
WY00000945
0.2548
WY00000311
0.309
N200000780
0.1697
WY00000001
WY00000434
0.1534
0.2882
WY00000366
0.2514
WY00000993
0.3137
0.2164
0.2742
WY00000977
WY00001021
0.1465
WY00001143
N200000999
0.1897
WY00000949
0.2269
WY00001273
0.1697
WY00001109
N200000350
0.1713
N200000346
0.3121
N200000471
JJ00000074
0.2411
0.1207
WY00001240
0.2411
WY00000379
0.1134
WY00000393
0.213
WY00001019

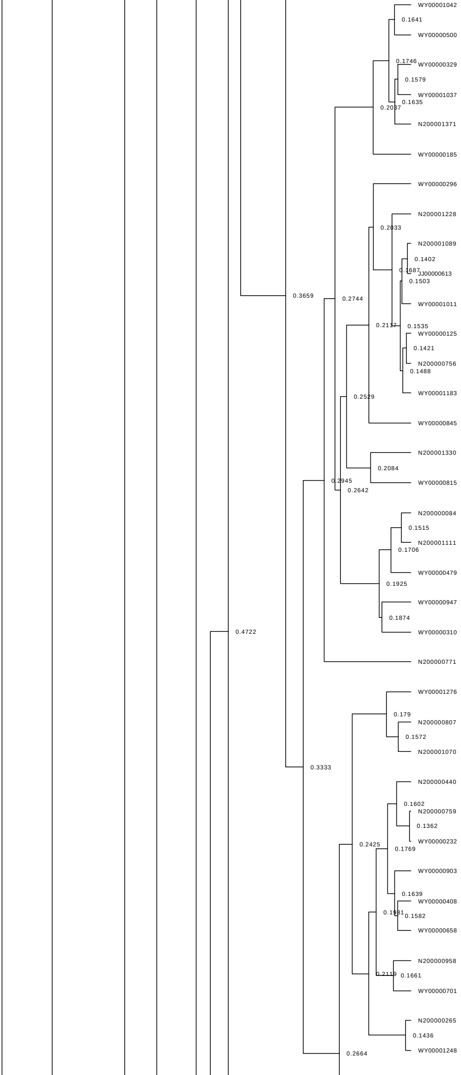
WY00001042
0.1641
WY00000500
0.1746
WY00000329
0.1579
WY00001037
0.1635
0.2037
N200001371
WY00000185
WY00000296
N200001228
0.2033
N200001089
0.1402
0.1687
JJ00000613
0.1503
WY00001011
0.3659
0.2744
0.2117
0.1535
WY00000125
0.1421
N200000756
0.1488
WY00001183
0.2529
WY00000845
N200001330
0.2084
0.2945
WY00000815
0.2642
N200000084
0.1515
N200001111
0.1706
WY00000479
0.1925
WY00000947
0.1874
0.4722
WY00000310
N200000771
WY00001276
0.179
N200000807
0.1572
N200001070
0.3333
N200000440
0.1602
N200000759
0.1362
WY00000232
0.2425
0.1769
WY00000903
0.1639
WY00000408
0.1981
0.1582
WY00000658
N200000958
0.2119
0.1661
WY00000701
N200000265
0.1436
WY00001248
0.2664

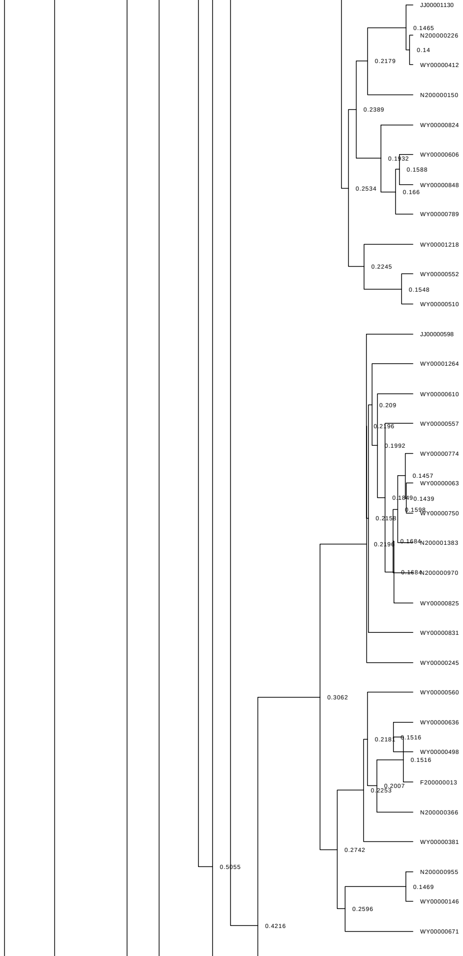
JJ00001130
0.1465
N200000226
0.14
0.2179
WY00000412
N200000150
0.2389
WY00000824
WY00000606
0.1932
0.1588
0.2534
WY00000848
0.166
WY00000789
WY00001218
0.2245
WY00000552
0.1548
WY00000510
JJ00000598
WY00001264
WY00000610
0.209
WY00000557
0.2196
0.1992
WY00000774
0.1457
WY00000063
0.1849
0.1439
0.1598
WY00000750
0.2158
0.1684
N200001383
0.2198
0.1684
N200000970
WY00000825
WY00000831
WY00000245
WY00000560
0.3062
WY00000636
0.2181
0.1516
WY00000498
0.1516
F200000013
0.2007
0.2253
N200000366
WY00000381
0.2742
0.5055
N200000955
0.1469
WY00000146
0.2596
0.4216
WY00000671

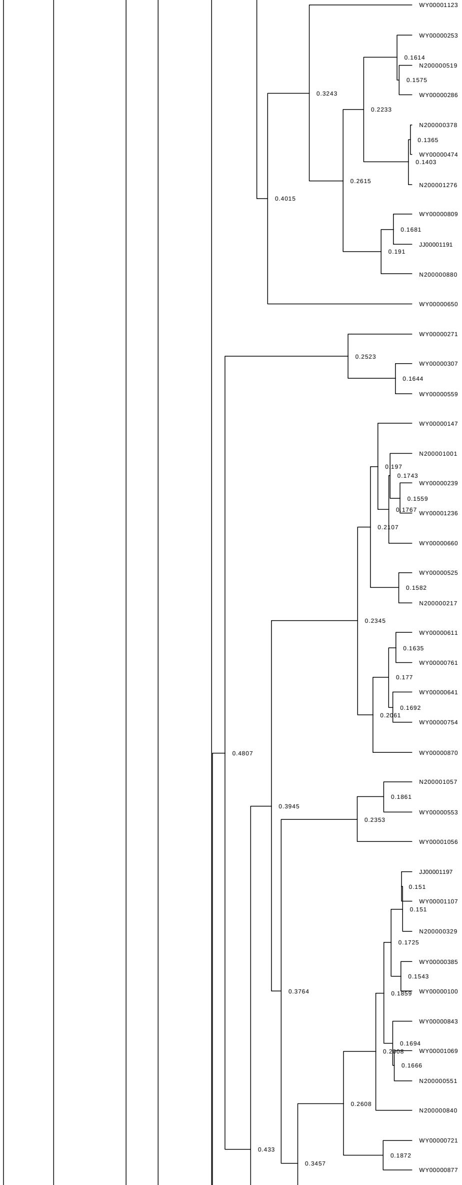
WY00001123
WY00000253
0.1614
N200000519
0.1575
WY00000286
0.3243
0.2233
N200000378
0.1365
WY00000474
0.1403
0.2615
N200001276
0.4015
WY00000809
0.1681
JJ00001191
0.191
N200000880
WY00000650
WY00000271
0.2523
WY00000307
0.1644
WY00000559
WY00000147
N200001001
0.197
0.1743
WY00000239
0.1559
0.1767
WY00001236
0.2107
WY00000660
WY00000525
0.1582
N200000217
0.2345
WY00000611
0.1635
WY00000761
0.177
WY00000641
0.1692
0.2061
WY00000754
0.4807
WY00000870
N200001057
0.1861
0.3945
WY00000553
0.2353
WY00001056
JJ00001197
0.151
WY00001107
0.151
N200000329
0.1725
WY00000385
0.1543
0.3764
0.1859
WY00000100
WY00000843
0.1694
0.2008
WY00001069
0.1666
N200000551
0.2608
N200000840
WY00000721
0.433
0.1872
0.3457
WY00000877

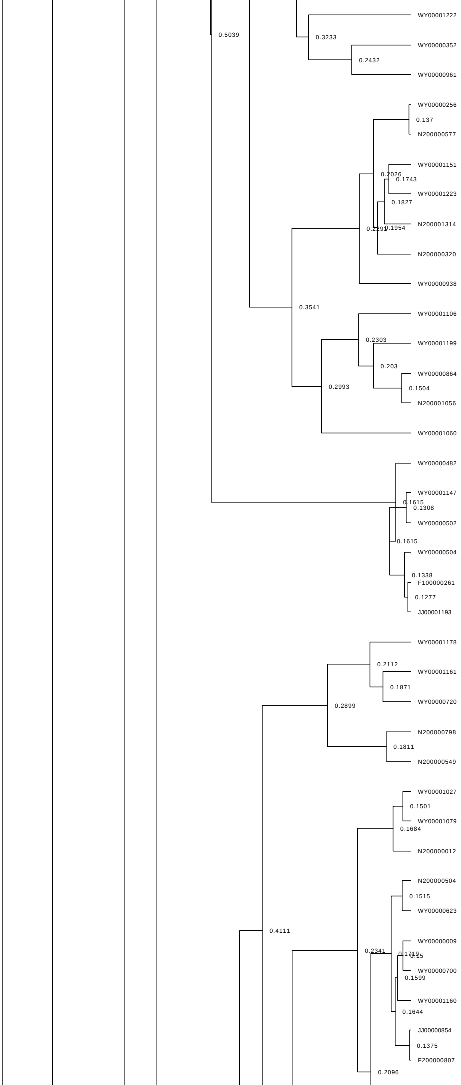

WY00001222
0.5039
0.3233
WY00000352
0.2432
WY00000961
WY00000256
0.137
N200000577
WY00001151
0.2026
0.1743
WY00001223
0.1827
N200001314
0.2291
0.1954
N200000320
WY00000938
0.3541
WY00001106
0.2303
WY00001199
0.203
WY00000864
0.2993
0.1504
N200001056
WY00001060
WY00000482
WY00001147
0.1615
0.1308
WY00000502
0.1615
WY00000504
0.1338
F100000261
0.1277
JJ00001193
WY00001178
0.2112
WY00001161
0.1871
WY00000720
0.2899
N200000798
0.1811
N200000549
WY00001027
0.1501
WY00001079
0.1684
N200000012
N200000504
0.1515
WY00000623
0.4111
WY00000009
0.2341
0.1719
0.15
WY00000700
0.1599
WY00001160
0.1644
JJ00000854
0.1375
F200000807
0.2096

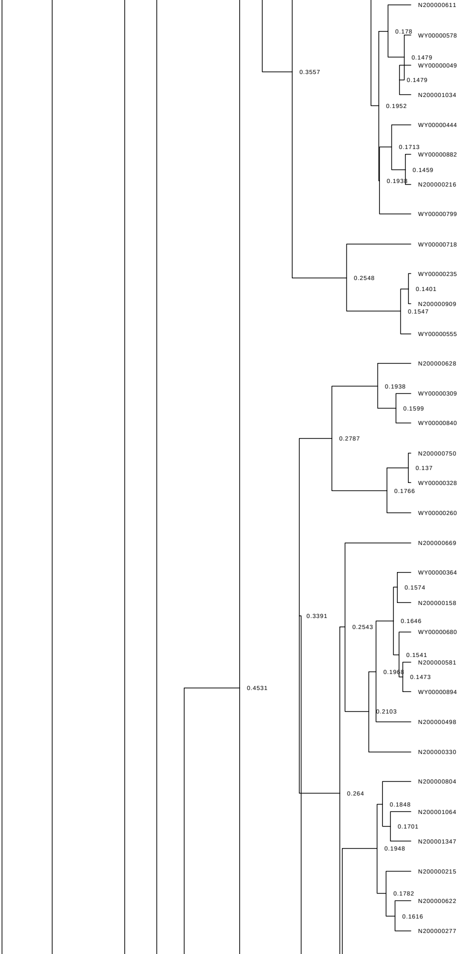

N200000611
0.178
WY00000578
0.1479
WY00000049
0.1479
N200001034
0.3557
0.1952
WY00000444
0.1713
WY00000882
0.1459
0.1938
N200000216
WY00000799
WY00000718
0.2548
WY00000235
0.1401
N200000909
0.1547
WY00000555
N200000628
0.1938
WY00000309
0.1599
WY00000840
0.2787
N200000750
0.137
WY00000328
0.1766
WY00000260
N200000669
WY00000364
0.1574
N200000158
0.3391
0.2543
0.1646
WY00000680
0.1541
N200000581
0.1968
0.1473
0.4531
WY00000894
0.2103
N200000498
N200000330
N200000804
0.264
0.1848
N200001064
0.1701
N200001347
0.1948
N200000215
0.1782
N200000622
0.1616
N200000277

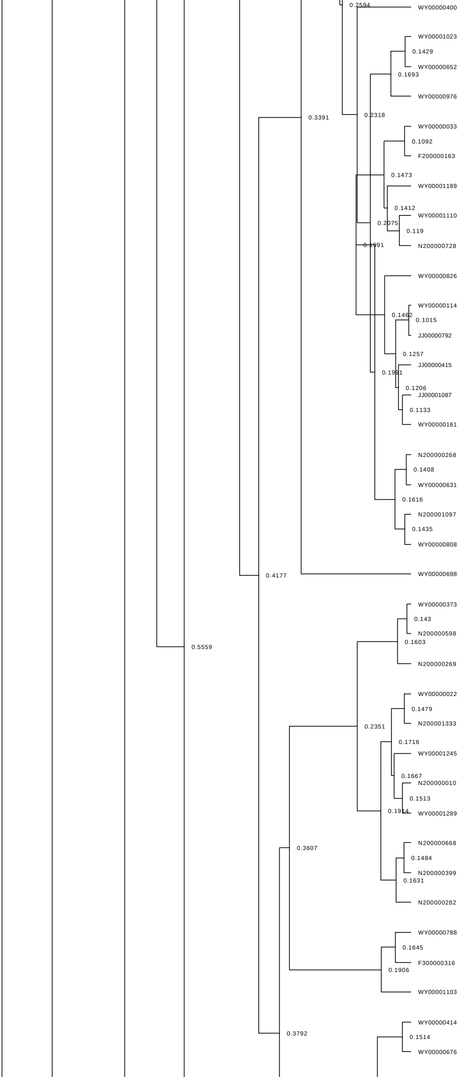

0.2594
WY00000400
WY00001023
0.1429
WY00000652
0.1693
WY00000976
0.3391
0.2318
WY00000033
0.1092
F200000163
0.1473
WY00001189
0.1412
WY00001110
0.2075
0.119
N200000728
0.1991
WY00000826
WY00000114
0.1462
0.1015
JJ00000792
0.1257
JJ00000415
0.1981
0.1206
JJ00001087
0.1133
WY00000161
N200000268
0.1408
WY00000631
0.1616
N200001097
0.1435
WY00000808
0.4177
WY00000698
WY00000373
0.143
N200000598
0.5559
0.1603
N200000269
WY00000022
0.1479
N200001333
0.2351
0.1716
WY00001245
0.1667
N200000010
0.1513
0.1914
WY00001289
0.3607
N200000668
0.1484
N200000399
0.1631
N200000282
WY00000788
0.1645
F300000316
0.1906
WY00001103
WY00000414
0.3792
0.1514
WY00000676

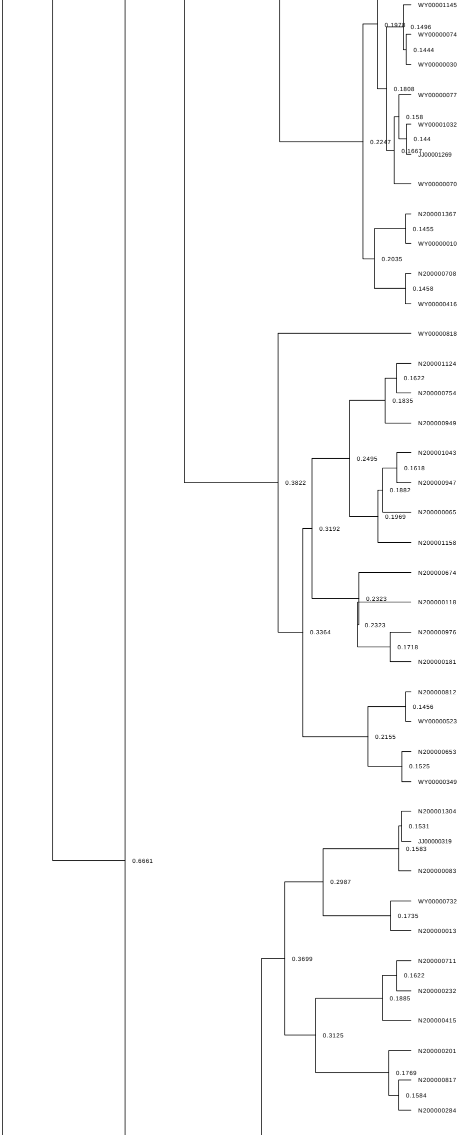
WY00001145
0.1978
0.1496
WY00000074
0.1444
WY00000030
0.1808
WY00000077
0.158
WY00001032
0.144
0.2247
0.1667
JJ00001269
WY00000070
N200001367
0.1455
WY00000010
0.2035
N200000708
0.1458
WY00000416
WY00000818
N200001124
0.1622
N200000754
0.1835
N200000949
N200001043
0.2495
0.1618
N200000947
0.3822
0.1882
N200000065
0.1969
0.3192
N200001158
N200000674
0.2323
N200000118
0.2323
N200000976
0.3364
0.1718
N200000181
N200000812
0.1456
WY00000523
0.2155
N200000653
0.1525
WY00000349
N200001304
0.1531
JJ00000319
0.1583
0.6661
N200000083
0.2987
WY00000732
0.1735
N200000013
0.3699
N200000711
0.1622
N200000232
0.1885
N200000415
0.3125
N200000201
0.1769
N200000817
0.1584
N200000284

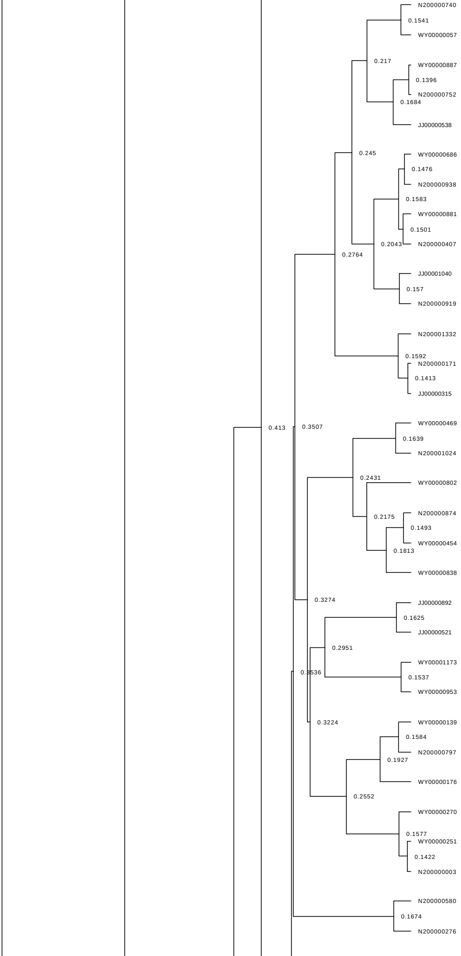
N200000740
0.1541
WY00000057
0.217
WY00000887
0.1396
N200000752
0.1684
JJ00000538
0.245
WY00000686
0.1476
N200000938
0.1583
WY00000881
0.1501
0.2043
N200000407
0.2764
JJ00001040
0.157
N200000919
N200001332
0.1592
N200000171
0.1413
JJ00000315
0.413
0.3507
WY00000469
0.1639
N200001024
0.2431
WY00000802
0.2175
N200000874
0.1493
WY00000454
0.1813
WY00000838
0.3274
JJ00000892
0.1625
JJ00000521
0.2951
WY00001173
0.1537
WY00000953
0.3224
WY00000139
0.1584
N200000797
0.1927
WY00000176
0.2552
WY00000270
0.1577
WY00000251
0.1422
N200000003
N200000580
0.1674
N200000276

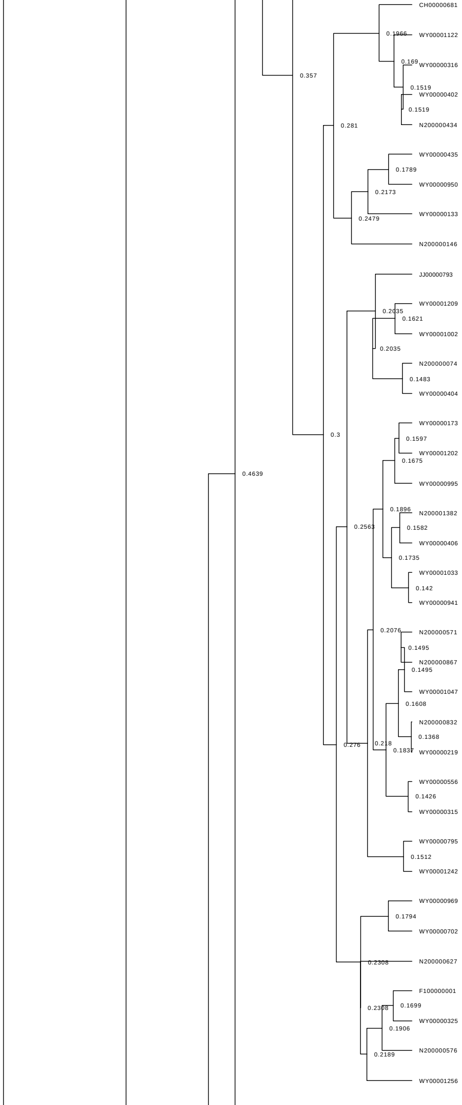
CH00000681
0.1966
WY00001122
0.169
WY00000316
0.1519
WY00000402
0.1519
N200000434
0.357
0.281
WY00000435
0.1789
WY00000950
0.2173
WY00000133
0.2479
N200000146
JJ00000793
WY00001209
0.2035
0.1621
WY00001002
0.2035
N200000074
0.1483
WY00000404
WY00000173
0.3
0.1597
WY00001202
0.1675
0.4639
WY00000995
0.1896
N200001382
0.1582
0.2563
WY00000406
0.1735
WY00001033
0.142
WY00000941
0.2076
N200000571
0.1495
N200000867
0.1495
WY00001047
0.1608
N200000832
0.1368
0.276
0.218
0.1837
WY00000219
WY00000556
0.1426
WY00000315
WY00000795
0.1512
WY00001242
WY00000969
0.1794
WY00000702
0.2308
N200000627
F100000001
0.1699
0.2308
WY00000325
0.1906
N200000576
0.2189
WY00001256

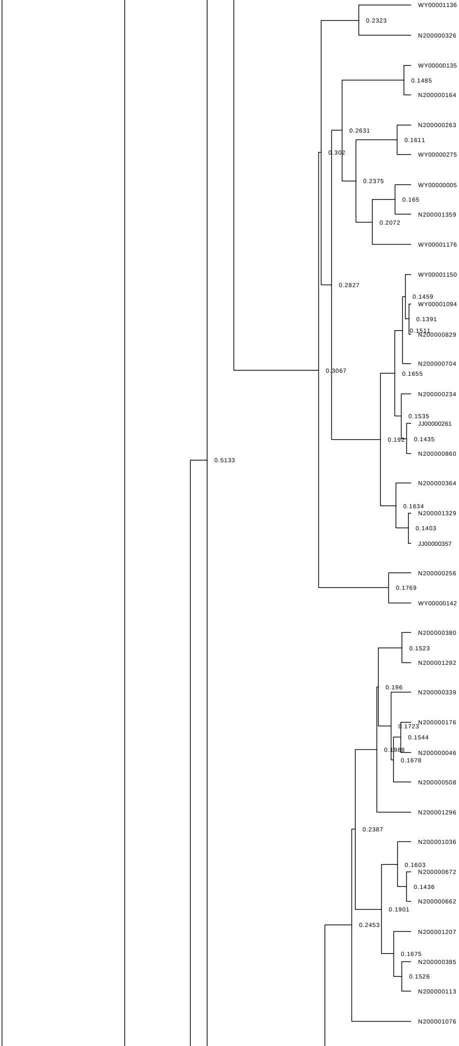
WY00001136
0.2323
N200000326
WY00000135
0.1485
N200000164
N200000263
0.2631
0.1611
0.302
WY00000275
0.2375
WY00000005
0.165
N200001359
0.2072
WY00001176
WY00001150
0.2827
0.1459
WY00001094
0.1391
0.1511
N200000829
N200000704
0.3067
0.1655
N200000234
0.1535
JJ00000261
0.192
0.1435
N200000860
0.5133
N200000364
0.1634
N200001329
0.1403
JJ00000357
N200000256
0.1769
WY00000142
N200000380
0.1523
N200001292
0.196
N200000339
N200000176
0.1723
0.1544
0.1988
N200000046
0.1678
N200000508
N200001296
0.2387
N200001036
0.1603
N200000672
0.1436
N200000662
0.1901
0.2453
N200001207
0.1675
N200000385
0.1526
N200000113
N200001076

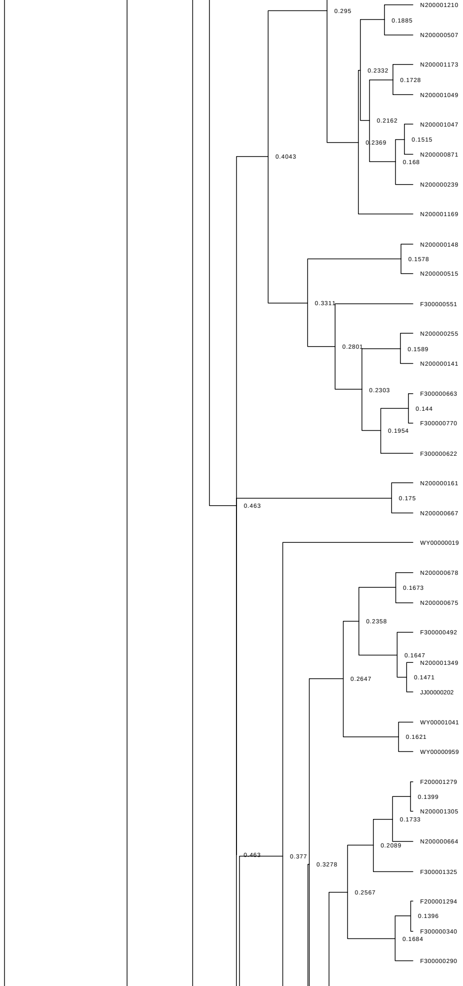
N200001210
0.295
0.1885
N200000507
N200001173
0.2332
0.1728
N200001049
0.2162
N200001047
0.1515
0.2369
N200000871
0.4043
0.168
N200000239
N200001169
N200000148
0.1578
N200000515
0.3311
F300000551
N200000255
0.2801
0.1589
N200000141
0.2303
F300000663
0.144
F300000770
0.1954
F300000622
N200000161
0.175
0.463
N200000667
WY00000019
N200000678
0.1673
N200000675
0.2358
F300000492
0.1647
N200001349
0.2647
0.1471
JJ00000202
WY00001041
0.1621
WY00000959
F200001279
0.1399
N200001305
0.1733
0.2089
N200000664
0.463
0.377
0.3278
F300001325
0.2567
F200001294
0.1396
F300000340
0.1684
F300000290

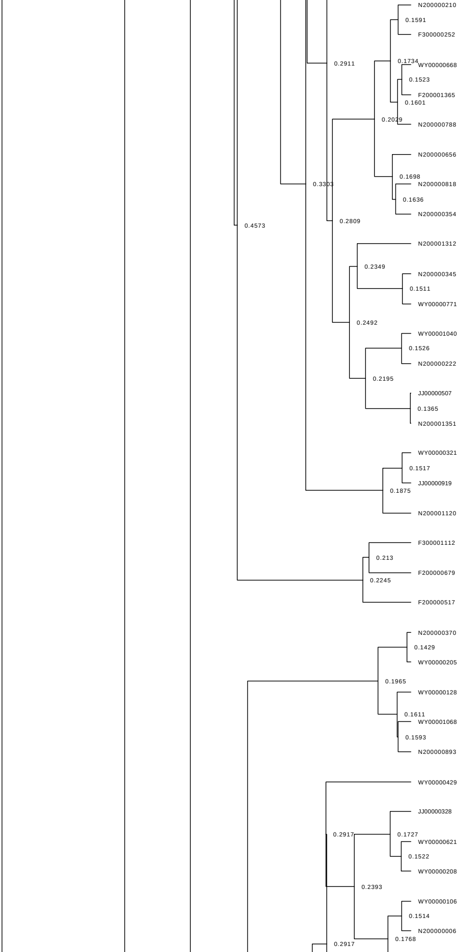

N200000210
0.1591
F300000252
0.2911
0.1734
WY00000668
0.1523
F200001365
0.1601
0.2029
N200000788
N200000656
0.1698
0.3303
N200000818
0.1636
N200000354
0.4573
0.2809
N200001312
0.2349
N200000345
0.1511
WY00000771
0.2492
WY00001040
0.1526
N200000222
0.2195
JJ00000507
0.1365
N200001351
WY00000321
0.1517
JJ00000919
0.1875
N200001120
F300001112
0.213
F200000679
0.2245
F200000517
N200000370
0.1429
WY00000205
0.1965
WY00000128
0.1611
WY00001068
0.1593
N200000893
WY00000429
JJ00000328
0.2917
0.1727
WY00000621
0.1522
WY00000208
0.2393
WY00000106
0.1514
N200000006
0.1768
0.2917

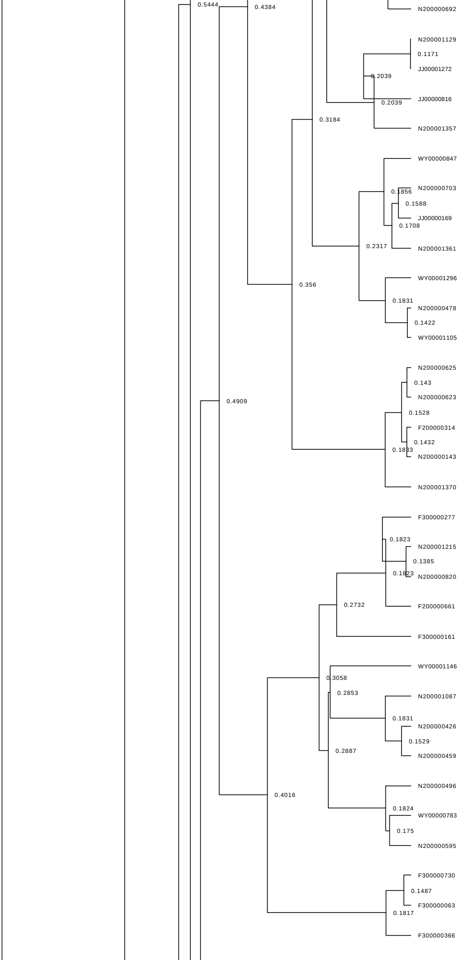
0.5444
0.4384
N200000692
N200001129
0.1171
JJ00001272
0.2039
JJ00000816
0.2039
0.3184
N200001357
WY00000847
0.1856
N200000703
0.1588
JJ00000169
0.1708
0.2317
N200001361
WY00001296
0.356
0.1831
N200000478
0.1422
WY00001105
N200000625
0.143
N200000623
0.4909
0.1528
F200000314
0.1432
0.1833
N200000143
N200001370
F300000277
0.1823
N200001215
0.1385
0.1823
N200000820
0.2732
F200000661
F300000161
WY00001146
0.3058
0.2853
N200001087
0.1831
N200000426
0.1529
0.2887
N200000459
N200000496
0.4016
0.1824
WY00000783
0.175
N200000595
F300000730
0.1487
F300000063
0.1817
F300000366

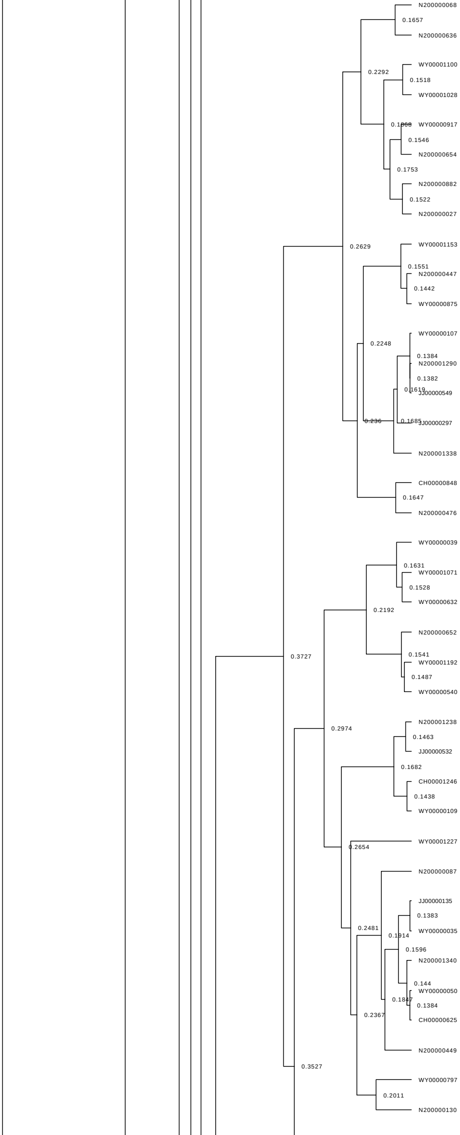
N200000068
0.1657
N200000636
WY00001100
0.1518
WY00001028
0.2292
WY00000917
0.1546
N200000654
0.1753
N200000882
0.1522
N200000027
0.2629
WY00001153
0.1551
N200000447
0.1442
WY00000875
0.2248
WY00000107
0.1384
N200001290
0.1382
JJ00000549
0.236
JJ00000297
N200001338
CH00000848
0.1647
N200000476
WY00000039
0.1631
WY00001071
0.1528
WY00000632
0.2192
N200000652
0.1541
WY00001192
0.1487
WY00000540
0.3727
N200001238
0.1463
JJ00000532
0.1682
CH00001246
0.1438
WY00000109
0.2974
WY00001227
0.2654
N200000087
JJ00000135
0.1383
WY00000035
0.2481
0.1596
N200001340
0.144
WY00000050
0.1384
CH00000625
0.2367
N200000449
0.3527
WY00000797
0.2011
N200000130

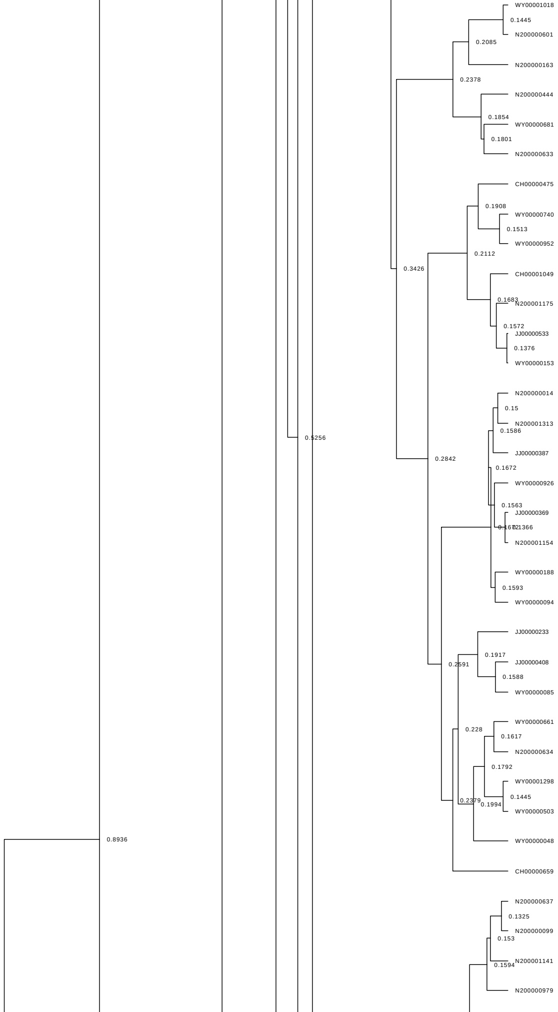
WY00001018
0.1445
N200000601
0.2085
N200000163
0.2378
N200000444
0.1854
WY00000681
0.1801
N200000633
CH00000475
0.1908
WY00000740
0.1513
WY00000952
0.2112
0.3426
CH00001049
0.1683
N200001175
0.1572
JJ00000533
0.1376
WY00000153
N200000014
0.15
N200001313
0.1586
0.5256
0.2842
JJ00000387
0.1672
WY00000926
0.1563
JJ00000369
0.1672
0.1366
N200001154
WY00000188
0.1593
WY00000094
JJ00000233
0.1917
0.2591
JJ00000408
0.1588
WY00000085
WY00000661
0.228
0.1617
N200000634
0.1792
WY00001298
0.1445
0.2379
0.1994
WY00000503
0.8936
WY00000048
CH00000659
N200000637
0.1325
N200000099
0.153
N200001141
0.1594
N200000979

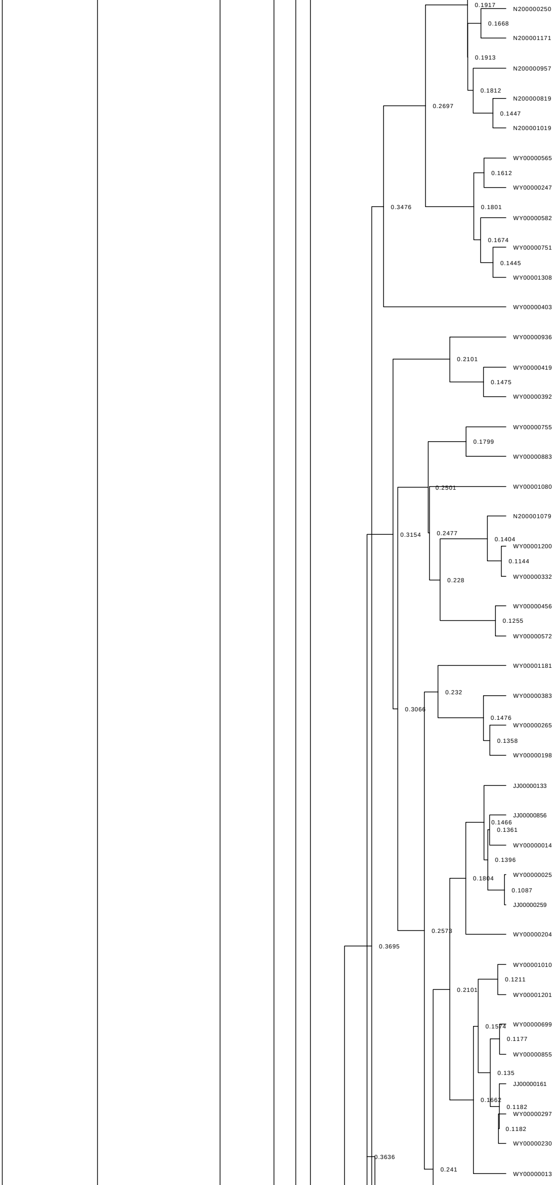

N200000250
N200001171
N200000957
N200000819
N200001019
WY00000565
WY00000247
WY00000582
WY00000751
WY00001308
WY00000403
WY00000936
WY00000419
WY00000392
WY00000755
WY00000883
WY00001080
N200001079
WY00001200
WY00000332
WY00000456
WY00000572
WY00001181
WY00000383
WY00000265
WY00000198
JJ00000133
JJ00000856
WY00000014
WY00000025
JJ00000259
WY00000204
WY00001010
WY00001201
WY00000699
WY00000855
JJ00000161
WY00000297
WY00000230
WY00000013
0.1917
0.1668
0.1913
0.1812
0.1447
0.2697
0.1612
0.3476
0.1801
0.1674
0.1445
0.2101
0.1475
0.1799
0.2501
0.2477
0.1404
0.3154
0.1144
0.228
0.1255
0.232
0.3066
0.1476
0.1358
0.1466
0.1361
0.1396
0.1804
0.1087
0.2573
0.3695
0.1211
0.2101
0.1574
0.1177
0.135
0.1662
0.1182
0.1182
0.3636
0.241

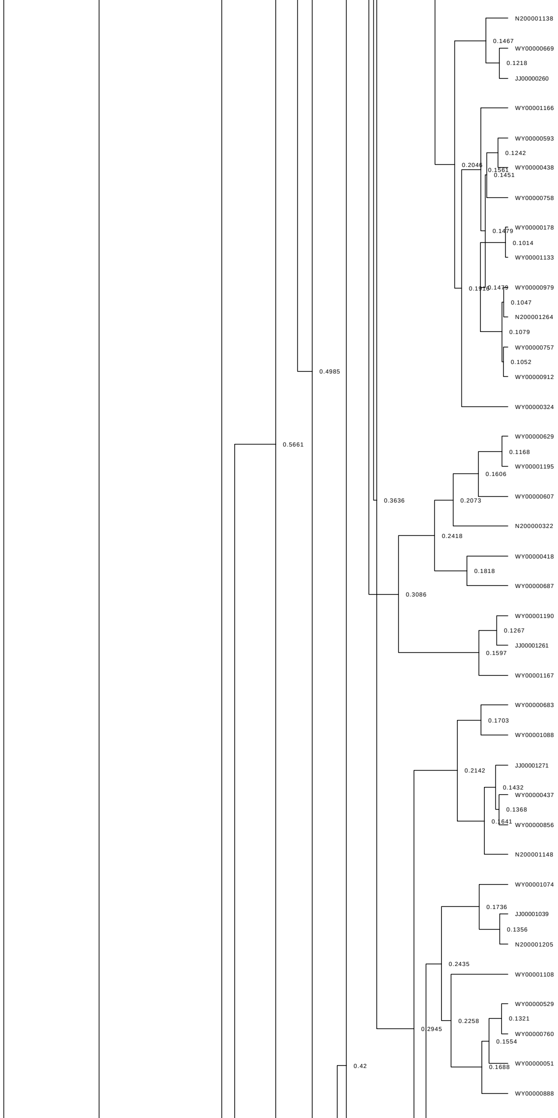
N200001138
0.1467
WY00000669
0.1218
JJ00000260
WY00001166
WY00000593
0.1242
0.2046
0.1561
WY00000438
0.1451
WY00000758
WY00000178
0.1479
0.1014
WY00001133
0.1916
0.1479
WY00000979
0.1047
N200001264
0.1079
WY00000757
0.1052
WY00000912
0.4985
WY00000324
WY00000629
0.5661
0.1168
WY00001195
0.1606
WY00000607
0.3636
0.2073
N200000322
0.2418
WY00000418
0.1818
WY00000687
0.3086
WY00001190
0.1267
JJ00001261
0.1597
WY00001167
WY00000683
0.1703
WY00001088
0.2142
JJ00001271
0.1432
WY00000437
0.1368
0.1641
WY00000856
N200001148
WY00001074
0.1736
JJ00001039
0.1356
N200001205
0.2435
WY00001108
WY00000529
0.1321
0.2258
0.2945
WY00000760
0.1554
WY00000051
0.42
0.1688
WY00000888

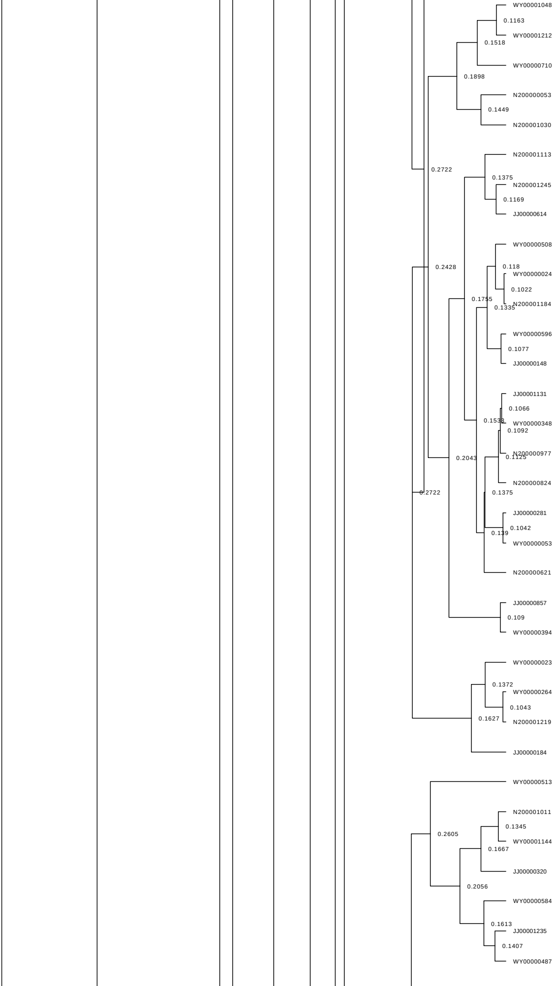
WY00001048
0.1163
WY00001212
0.1518
WY00000710
0.1898
N200000053
0.1449
N200001030
N200001113
0.2722
0.1375
N200001245
0.1169
JJ00000614
WY00000508
0.2428
0.118
WY00000024
0.1022
0.1755
N200001184
0.1335
WY00000596
0.1077
JJ00000148
JJ00001131
0.1066
0.1538
WY00000348
0.1092
N200000977
0.2043
0.1125
N200000824
0.2722
0.1375
JJ00000281
0.1042
0.139
WY00000053
N200000621
JJ00000857
0.109
WY00000394
WY00000023
0.1372
WY00000264
0.1043
0.1627
N200001219
JJ00000184
WY00000513
N200001011
0.1345
0.2605
WY00001144
0.1667
JJ00000320
0.2056
WY00000584
0.1613
JJ00001235
0.1407
WY00000487

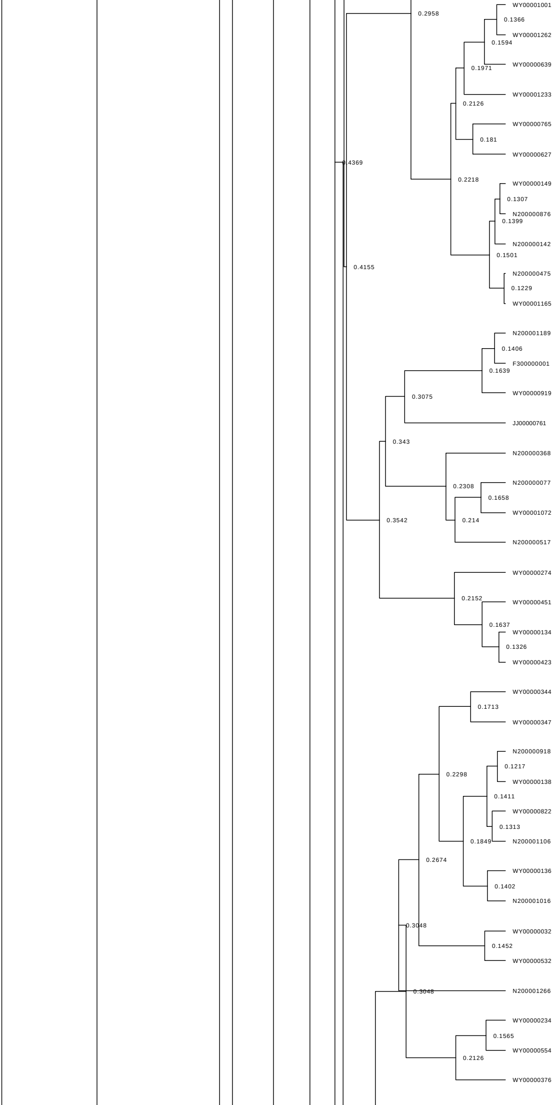
WY00001001
0.2958
0.1366
WY00001262
0.1594
WY00000639
0.1971
WY00001233
0.2126
WY00000765
0.181
WY00000627
0.4369
0.2218
WY00000149
0.1307
N200000876
0.1399
N200000142
0.1501
0.4155
N200000475
0.1229
WY00001165
N200001189
0.1406
F300000001
0.1639
WY00000919
0.3075
JJ00000761
0.343
N200000368
N200000077
0.2308
0.1658
WY00001072
0.3542
0.214
N200000517
WY00000274
0.2152
WY00000451
0.1637
WY00000134
0.1326
WY00000423
WY00000344
0.1713
WY00000347
N200000918
0.1217
0.2298
WY00000138
0.1411
WY00000822
0.1313
0.1849
N200001106
0.2674
WY00000136
0.1402
N200001016
0.3048
WY00000032
0.1452
WY00000532
0.3048
N200001266
WY00000234
0.1565
WY00000554
0.2126
WY00000376

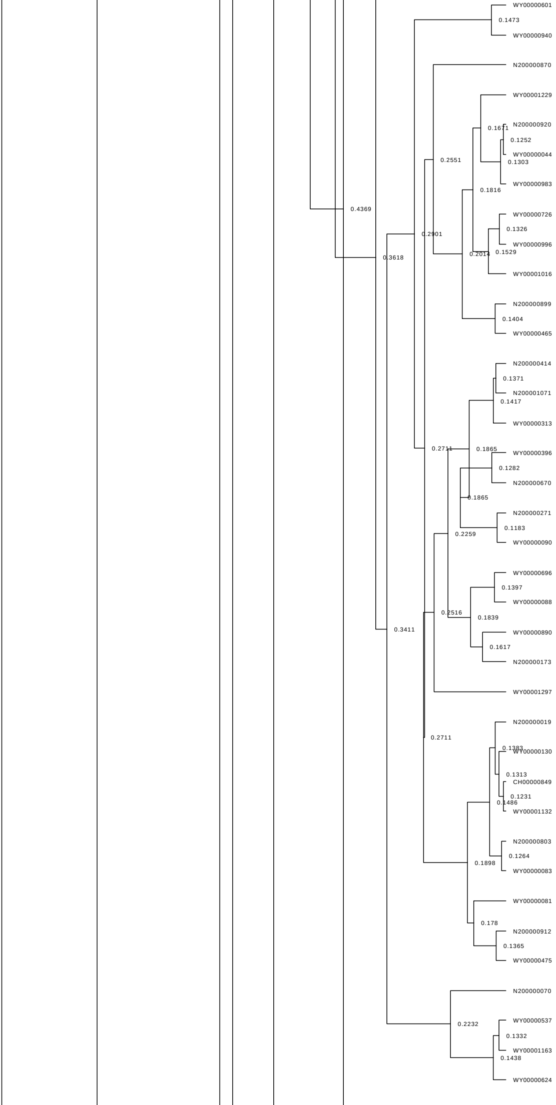
WY00000601
0.1473
WY00000940
N200000870
WY00001229
0.1671
N200000920
0.1252
WY00000044
0.1303
0.2551
WY00000983
0.1816
0.4369
WY00000726
0.1326
0.2901
WY00000996
0.1529
0.2014
0.3618
WY00001016
N200000899
0.1404
WY00000465
N200000414
0.1371
N200001071
0.1417
WY00000313
0.2711
0.1865
WY00000396
0.1282
N200000670
0.1865
N200000271
0.1183
0.2259
WY00000090
WY00000696
0.1397
WY00000088
0.2516
0.1839
0.3411
WY00000890
0.1617
N200000173
WY00001297
N200000019
0.2711
0.1383
WY00000130
0.1313
CH00000849
0.1231
0.1486
WY00001132
N200000803
0.1264
0.1898
WY00000083
WY00000081
0.178
N200000912
0.1365
WY00000475
N200000070
WY00000537
0.2232
0.1332
WY00001163
0.1438
WY00000624

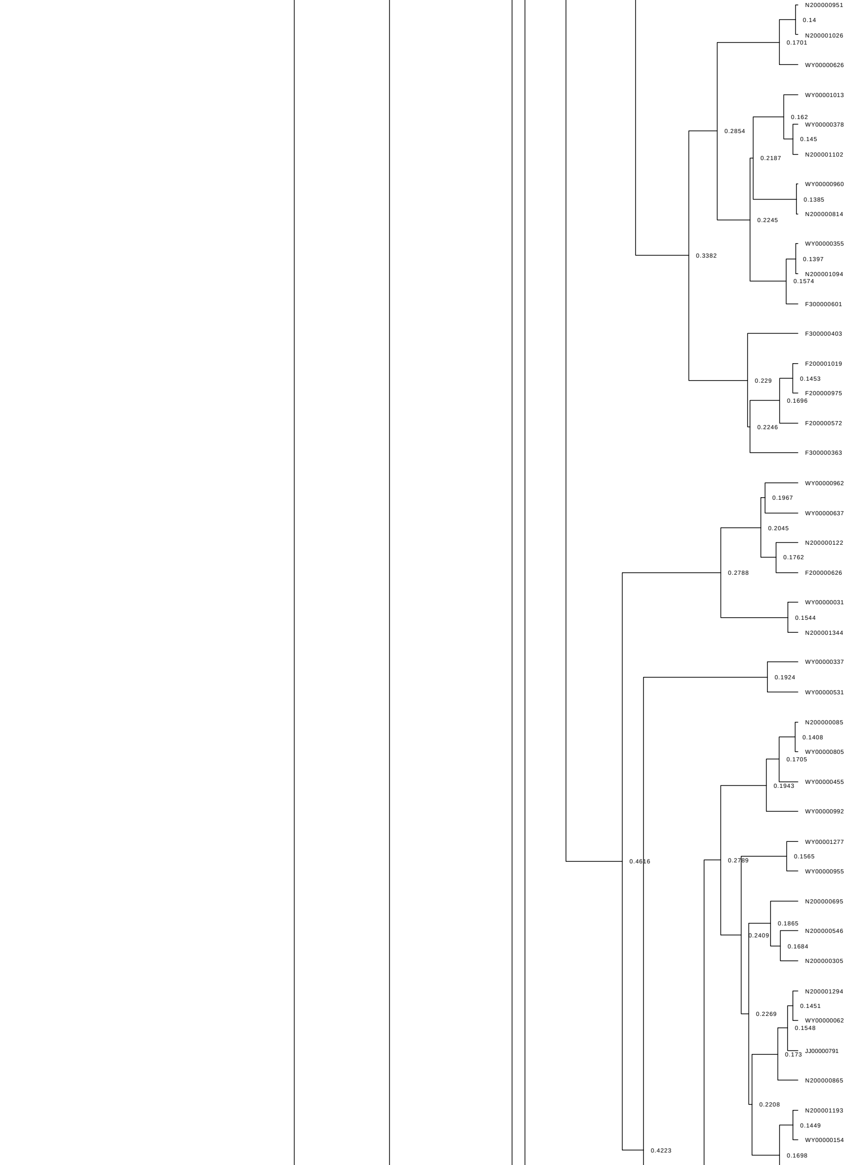
N200000951
0.14
N200001026
0.1701
WY00000626
WY00001013
0.162
WY00000378
0.145
N200001102
0.2854
0.2187
WY00000960
0.1385
N200000814
0.2245
WY00000355
0.1397
0.3382
N200001094
0.1574
F300000601
F300000403
F200001019
0.1453
0.229
F200000975
0.1696
F200000572
0.2246
F300000363
WY00000962
0.1967
WY00000637
0.2045
N200000122
0.1762
F200000626
0.2788
WY00000031
0.1544
N200001344
WY00000337
0.1924
WY00000531
N200000085
0.1408
WY00000805
0.1705
WY00000455
0.1943
WY00000992
WY00001277
0.1565
0.4616
0.2789
WY00000955
N200000695
0.1865
N200000546
0.2409
0.1684
N200000305
N200001294
0.1451
WY00000062
0.2269
0.1548
JJ00000791
0.173
N200000865
0.2208
N200001193
0.1449
WY00000154
0.4223
0.1698

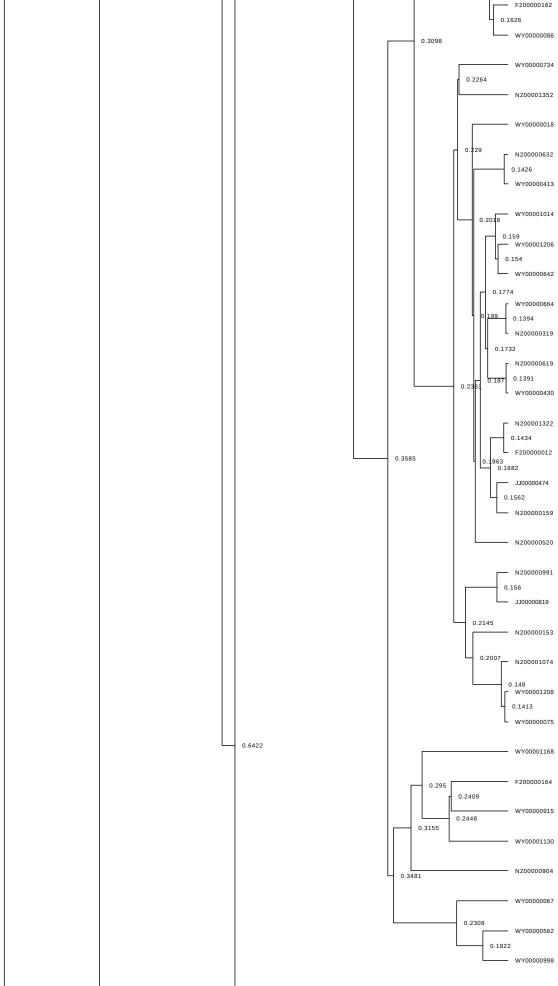
F200000162
0.1626
WY00000086
0.3098
WY00000734
0.2264
N200001352
WY00000018
0.229
N200000632
0.1426
WY00000413
WY00001014
0.2018
0.159
WY00001206
0.154
WY00000642
0.1774
WY00000664
0.199
0.1394
N200000319
0.1732
N200000619
0.1391
0.187
0.2361
WY00000430
N200001322
0.1434
F200000012
0.1963
0.1682
JJ00000474
0.1562
N200000159
0.3585
N200000520
N200000991
0.156
JJ00000819
0.2145
N200000153
0.2007
N200001074
0.148
WY00001208
0.1413
WY00000075
0.6422
WY00001168
F200000164
0.295
0.2409
WY00000915
0.2448
0.3155
WY00001130
N200000904
0.3481
WY00000067
0.2308
WY00000562
0.1822
WY00000998

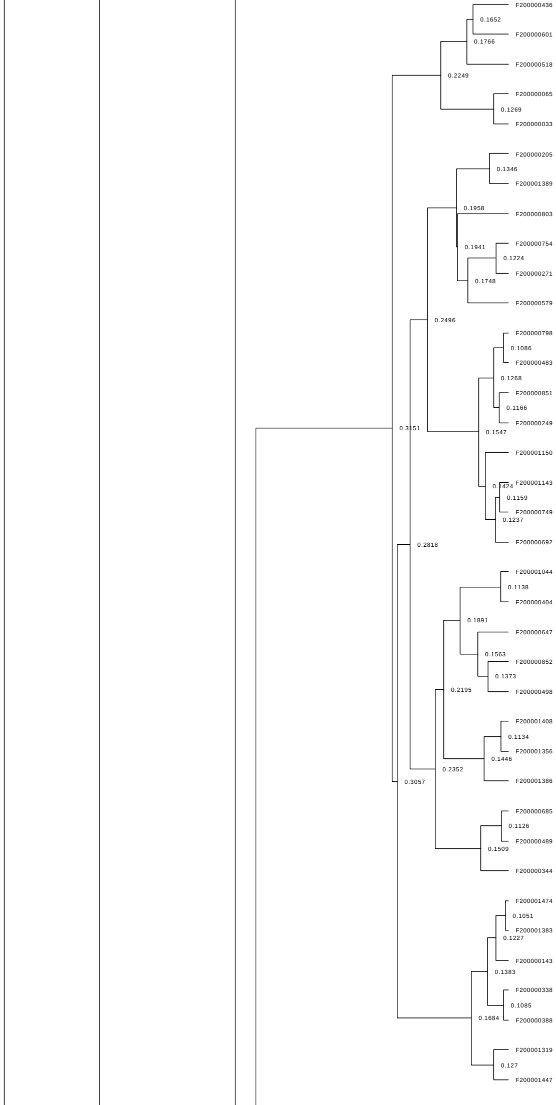
F200000436
0.1652
F200000601
0.1766
F200000518
0.2249
F200000065
0.1269
F200000033
F200000205
0.1346
F200001389
0.1958
F200000803
0.1941
F200000754
0.1224
F200000271
0.1748
F200000579
0.2496
F200000798
0.1086
F200000483
0.1268
F200000851
0.1166
F200000249
0.3151
0.1547
F200001150
F200001143
0.1424
0.1159
F200000749
0.1237
F200000692
0.2818
F200001044
0.1138
F200000404
0.1891
F200000647
0.1563
F200000852
0.1373
F200000498
0.2195
F200001408
0.1134
F200001356
0.1446
0.2352
F200001386
0.3057
F200000685
0.1126
F200000489
0.1509
F200000344
F200001474
0.1051
F200001383
0.1227
F200000143
0.1383
F200000338
0.1085
F200000388
0.1684
F200001319
0.127
F200001447

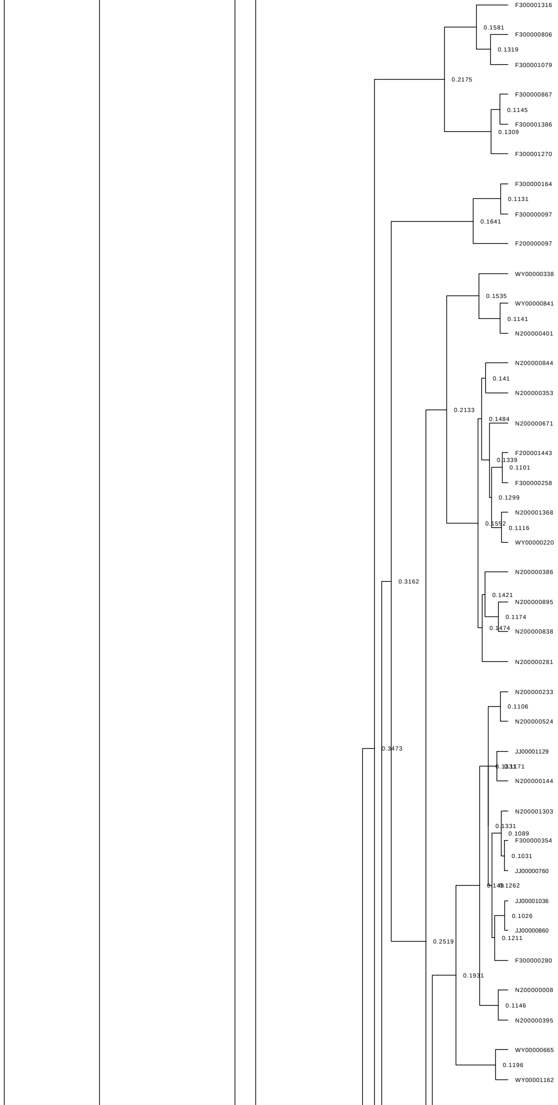
F300001316
0.1581
F300000806
0.1319
F300001079
0.2175
F300000867
0.1145
F300001386
0.1309
F300001270
F300000164
0.1131
F300000097
0.1641
F200000097
WY00000338
0.1535
WY00000841
0.1141
N200000401
N200000844
0.141
N200000353
0.2133
0.1484
N200000671
F200001443
0.1339
0.1101
F300000258
0.1299
N200001368
0.1552
0.1116
WY00000220
N200000386
0.3162
0.1421
N200000895
0.1174
0.1474
N200000838
N200000281
N200000233
0.1106
N200000524
JJ00001129
0.3473
N200000144
N200001303
0.1331
0.1089
F300000354
0.1031
JJ00000760
0.1262
JJ00001036
0.1026
JJ00000860
0.1211
0.2519
F300000280
0.1931
N200000008
0.1146
N200000395
WY00000665
0.1196
WY00001162

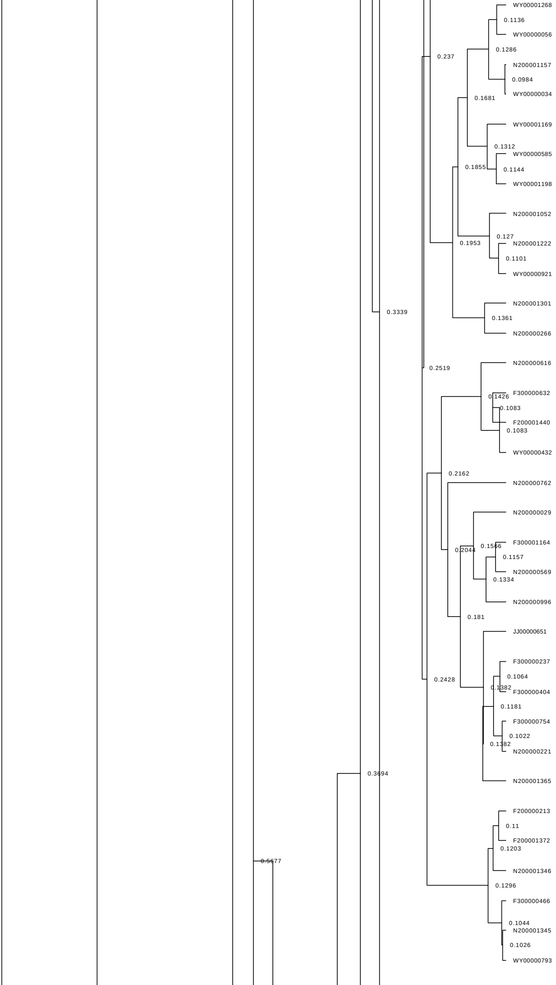
WY00001268
0.1136
WY00000056
0.1286
0.237
N200001157
0.0984
WY00000034
0.1681
WY00001169
0.1312
WY00000585
0.1855
0.1144
WY00001198
N200001052
0.127
0.1953
N200001222
0.1101
WY00000921
0.3339
N200001301
0.1361
N200000266
N200000616
0.2519
F300000632
0.1426
0.1083
F200001440
0.1083
WY00000432
0.2162
N200000762
N200000029
F300001164
0.1566
0.2044
0.1157
N200000569
0.1334
N200000996
0.181
JJ00000651
F300000237
0.1064
0.2428
0.1382
F300000404
0.1181
F300000754
0.1022
0.1382
N200000221
N200001365
0.3694
F200000213
0.11
F200001372
0.1203
0.5677
N200001346
0.1296
F300000466
0.1044
N200001345
0.1026
WY00000793

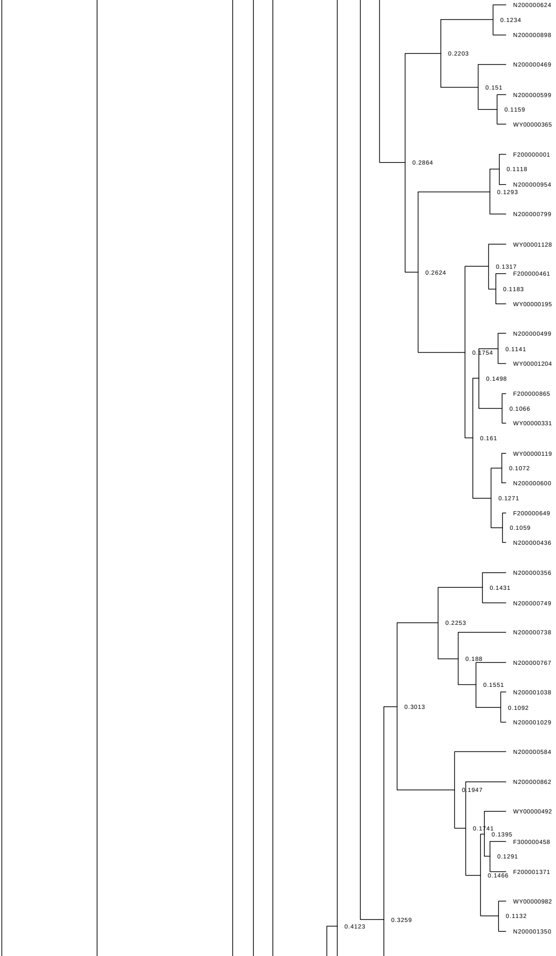

N200000624
0.1234
N200000898
0.2203
N200000469
0.151
N200000599
0.1159
WY00000365
F200000001
0.1118
0.2864
N200000954
0.1293
N200000799
WY00001128
0.1317
0.2624
F200000461
0.1183
WY00000195
N200000499
0.1141
0.1754
WY00001204
0.1498
F200000865
0.1066
WY00000331
0.161
WY00000119
0.1072
N200000600
0.1271
F200000649
0.1059
N200000436
N200000356
0.1431
N200000749
0.2253
N200000738
0.188
N200000767
0.1551
N200001038
0.3013
0.1092
N200001029
N200000584
N200000862
0.1947
WY00000492
0.1741
0.1395
F300000458
0.1291
F200001371
0.1466
WY00000982
0.1132
N200001350
0.3259
0.4123

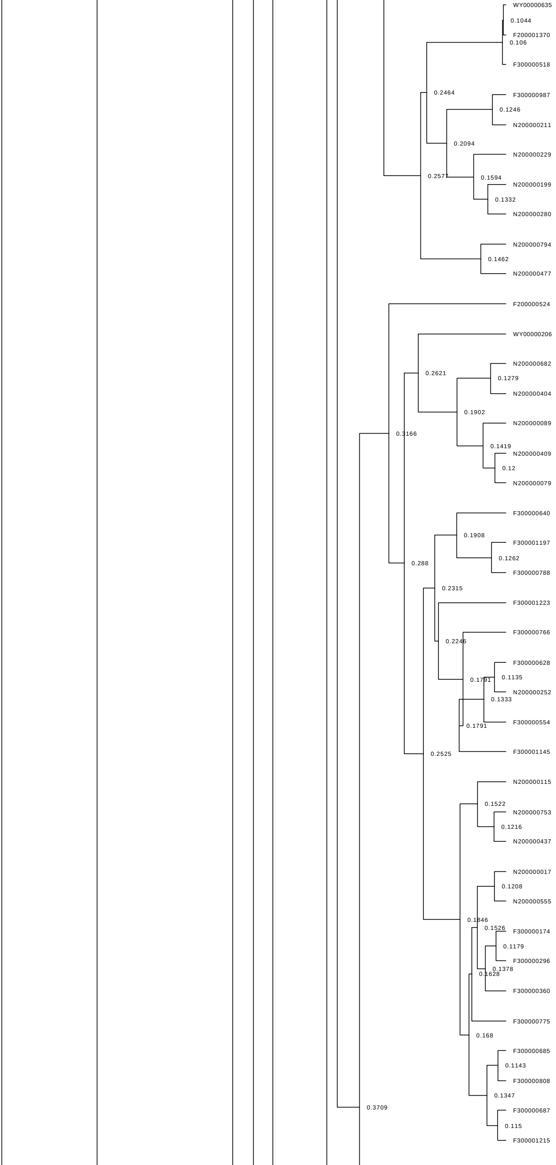

WY00000635
0.1044
F200001370
0.106
F300000518
0.2464
F300000987
0.1246
N200000211
0.2094
N200000229
0.2577
0.1594
N200000199
0.1332
N200000280
N200000794
0.1462
N200000477
F200000524
WY00000206
0.2621
N200000682
0.1279
N200000404
0.1902
N200000089
0.3166
0.1419
N200000409
0.12
N200000079
F300000640
0.1908
F300001197
0.1262
0.288
F300000788
0.2315
F300001223
F300000766
0.2246
F300000628
0.1135
0.1791
N200000252
0.1333
F300000554
0.1791
F300001145
0.2525
N200000115
0.1522
N200000753
0.1216
N200000437
N200000017
0.1208
N200000555
0.1846
0.1526
F300000174
0.1179
F300000296
0.1378
0.1628
F300000360
F300000775
0.168
F300000685
0.1143
F300000808
0.1347
F300000687
0.3709
0.115
F300001215

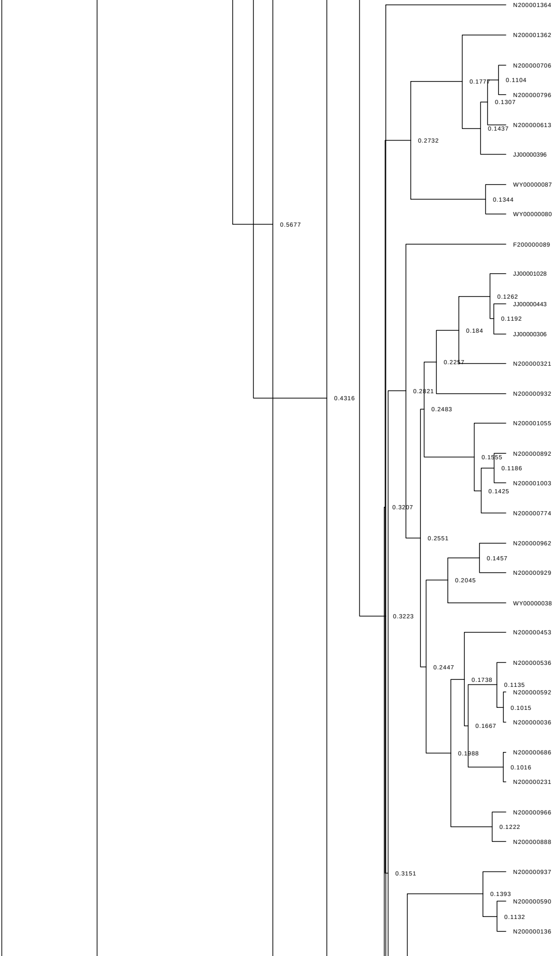
N200001364
N200001362
N200000706
0.1104
N200000796
0.1777
0.1307
N200000613
0.1437
0.2732
JJ00000396
WY00000087
0.1344
WY00000080
0.5677
F200000089
JJ00001028
0.1262
JJ00000443
0.1192
JJ00000306
0.184
0.2257
N200000321
0.2821
N200000932
0.2483
0.4316
N200001055
N200000892
0.1555
0.1186
N200001003
0.1425
0.3207
N200000774
0.2551
N200000962
0.1457
N200000929
0.2045
WY00000038
0.3223
N200000453
N200000536
0.2447
0.1738
0.1135
N200000592
0.1015
N200000036
0.1667
N200000686
0.1988
0.1016
N200000231
N200000966
0.1222
N200000888
N200000937
0.3151
0.1393
N200000590
0.1132
N200000136

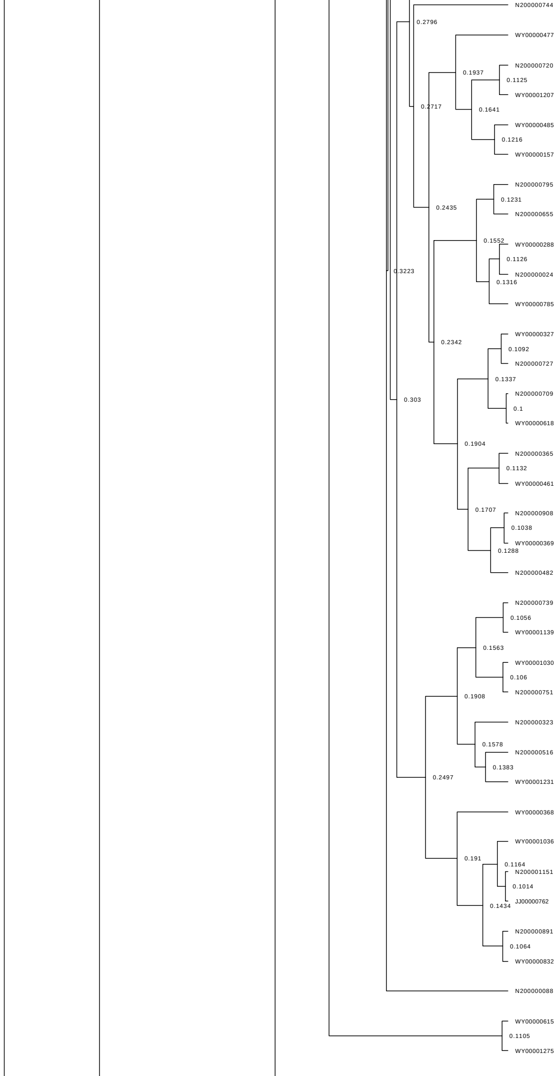

N200000744
0.2796
WY00000477
N200000720
0.1937
0.1125
WY00001207
0.2717
0.1641
WY00000485
0.1216
WY00000157
N200000795
0.1231
0.2435
N200000655
0.1552
WY00000288
0.1126
0.3223
N200000024
0.1316
WY00000785
WY00000327
0.2342
0.1092
N200000727
0.1337
N200000709
0.303
0.1
WY00000618
0.1904
N200000365
0.1132
WY00000461
0.1707
N200000908
0.1038
WY00000369
0.1288
N200000482
N200000739
0.1056
WY00001139
0.1563
WY00001030
0.106
N200000751
0.1908
N200000323
0.1578
N200000516
0.1383
0.2497
WY00001231
WY00000368
WY00001036
0.191
0.1164
N200001151
0.1014
JJ00000762
0.1434
N200000891
0.1064
WY00000832
N200000088
WY00000615
0.1105
WY00001275

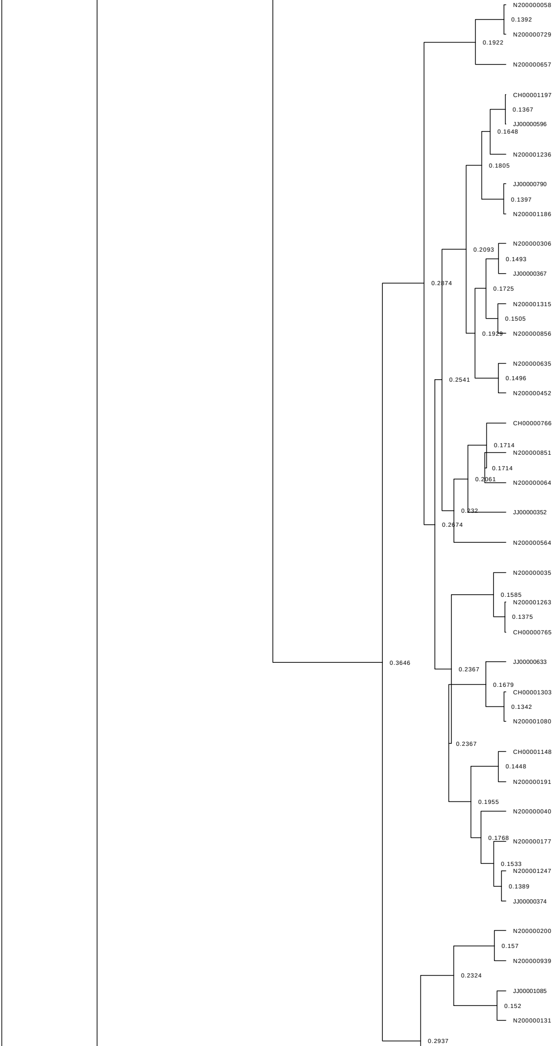
N200000058
0.1392
N200000729
0.1922
N200000657
CH00001197
0.1367
JJ00000596
0.1648
N200001236
0.1805
JJ00000790
0.1397
N200001186
N200000306
0.2093
0.1493
JJ00000367
0.2874
0.1725
N200001315
0.1505
0.1929
N200000856
N200000635
0.1496
0.2541
N200000452
CH00000766
0.1714
N200000851
0.1714
0.2061
N200000064
0.232
JJ00000352
0.2674
N200000564
N200000035
0.1585
N200001263
0.1375
CH00000765
0.3646
JJ00000633
0.2367
0.1679
CH00001303
0.1342
N200001080
0.2367
CH00001148
0.1448
N200000191
0.1955
N200000040
0.1768
N200000177
0.1533
N200001247
0.1389
JJ00000374
N200000200
0.157
N200000939
0.2324
JJ00001085
0.152
N200000131
0.2937

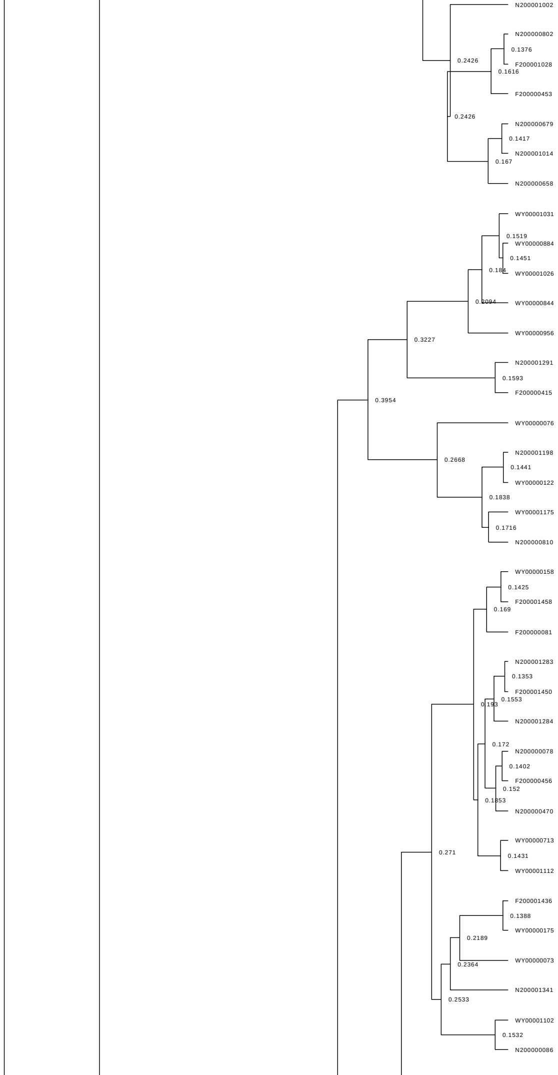

N200001002
N200000802
0.1376
F200001028
0.2426
0.1616
F200000453
0.2426
N200000679
0.1417
N200001014
0.167
N200000658
WY00001031
0.1519
WY00000884
0.1451
0.184
WY00001026
0.2094
WY00000844
WY00000956
0.3227
N200001291
0.1593
F200000415
0.3954
WY00000076
0.2668
N200001198
0.1441
WY00000122
0.1838
WY00001175
0.1716
N200000810
WY00000158
0.1425
F200001458
0.169
F200000081
N200001283
0.1353
F200001450
0.1553
0.193
N200001284
0.172
N200000078
0.1402
F200000456
0.152
0.1853
N200000470
WY00000713
0.271
0.1431
WY00001112
F200001436
0.1388
WY00000175
0.2189
WY00000073
0.2364
N200001341
0.2533
WY00001102
0.1532
N200000086

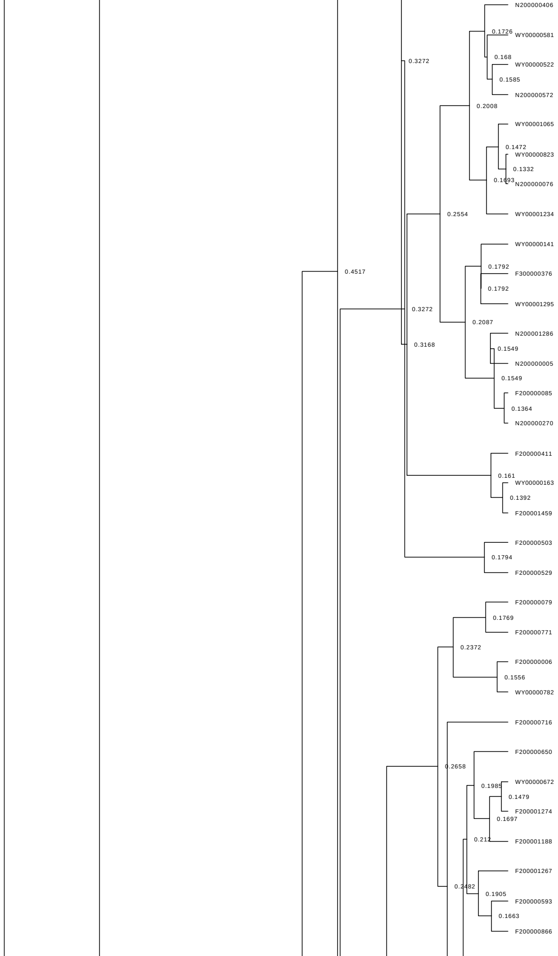

N200000406
0.1726
WY00000581
0.168
0.3272
WY00000522
0.1585
N200000572
0.2008
WY00001065
0.1472
WY00000823
0.1332
0.1693
N200000076
0.2554
WY00001234
WY00000141
0.1792
0.4517
F300000376
0.1792
WY00001295
0.3272
0.2087
N200001286
0.3168
0.1549
N200000005
0.1549
F200000085
0.1364
N200000270
F200000411
0.161
WY00000163
0.1392
F200001459
F200000503
0.1794
F200000529
F200000079
0.1769
F200000771
0.2372
F200000006
0.1556
WY00000782
F200000716
F200000650
0.2658
WY00000672
0.1985
0.1479
F200001274
0.1697
0.212
F200001188
F200001267
0.2482
0.1905
F200000593
0.1663
F200000866

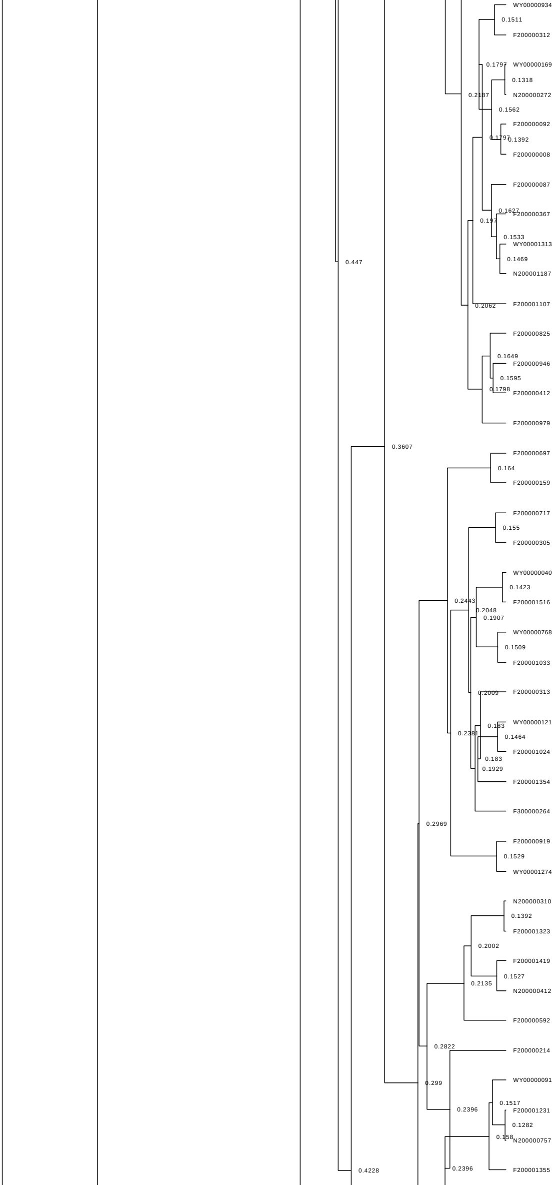

WY00000934
0.1511
F200000312
0.1797
WY00000169
0.1318
N200000272
0.2187
0.1562
F200000092
0.1797
0.1392
F200000008
F200000087
0.1627
F200000367
0.197
0.1533
WY00001313
0.1469
N200001187
0.447
0.2062
F200001107
F200000825
0.1649
F200000946
0.1595
0.1798
F200000412
F200000979
0.3607
F200000697
0.164
F200000159
F200000717
0.155
F200000305
WY00000040
0.1423
0.2443
F200001516
0.2048
0.1907
WY00000768
0.1509
F200001033
0.2009
F200000313
0.183
WY00000121
0.2381
0.1464
F200001024
0.183
0.1929
F200001354
F300000264
0.2969
F200000919
0.1529
WY00001274
N200000310
0.1392
F200001323
0.2002
F200001419
0.1527
0.2135
N200000412
F200000592
0.2822
F200000214
0.299
WY00000091
0.1517
0.2396
F200001231
0.1282
0.158
N200000757
0.4228
0.2396
F200001355

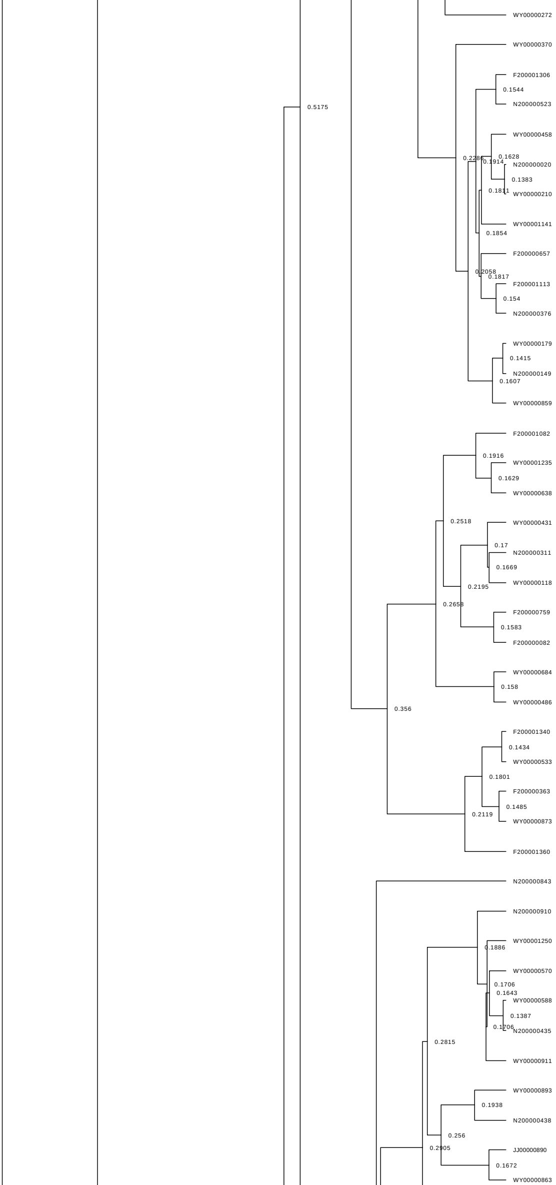
WY00000272
WY00000370
F200001306
0.1544
N200000523
WY00000458
0.1628
0.2286
0.1914
N200000020
0.1383
0.1811
WY00000210
WY00001141
0.1854
F200000657
0.2058
0.1817
F200001113
0.154
N200000376
WY00000179
0.1415
N200000149
0.1607
WY00000859
0.5175
F200001082
0.1916
WY00001235
0.1629
WY00000638
0.2518
WY00000431
0.17
N200000311
0.1669
WY00000118
0.2195
0.2658
F200000759
0.1583
F200000082
WY00000684
0.158
WY00000486
0.356
F200001340
0.1434
WY00000533
0.1801
F200000363
0.1485
0.2119
WY00000873
F200001360
N200000843
N200000910
WY00001250
0.1886
WY00000570
0.1706
0.1643
WY00000588
0.1387
0.1706
N200000435
0.2815
WY00000911
WY00000893
0.1938
N200000438
0.256
0.2905
JJ00000890
0.1672
WY00000863

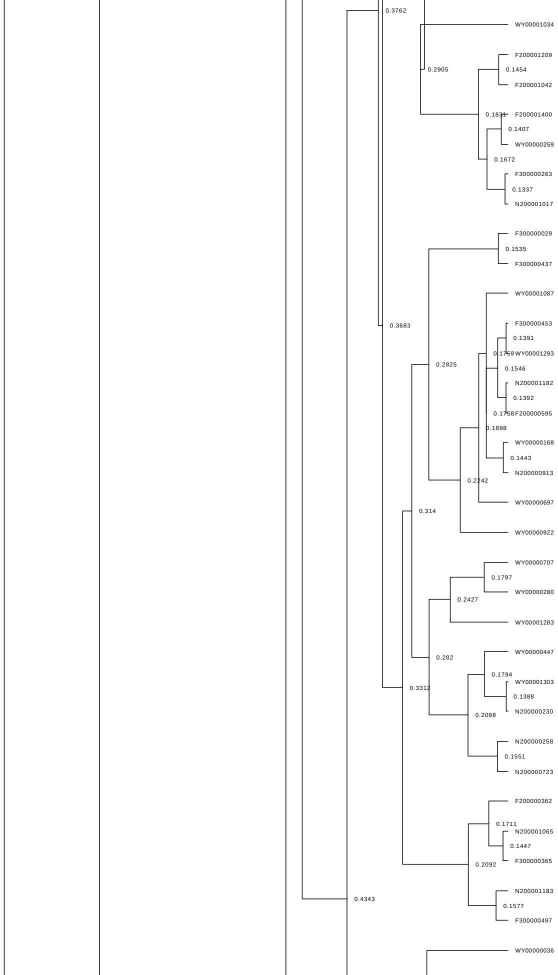

0.3762
WY00001034
F200001209
0.1454
0.2905
F200001042
0.1831
F200001400
0.1407
WY00000259
0.1672
F300000263
0.1337
N200001017
F300000029
0.1535
F300000437
WY00001087
0.3683
F300000453
0.1391
0.1759
WY00001293
0.2825
0.1546
N200001182
0.1392
0.1758
F200000595
0.1898
WY00000168
0.1443
N200000913
0.2242
WY00000697
0.314
WY00000922
WY00000707
0.1797
WY00000280
0.2427
WY00001283
WY00000447
0.282
0.1794
WY00001303
0.3312
0.1388
N200000230
0.2098
N200000258
0.1551
N200000723
F200000362
0.1711
N200001065
0.1447
F300000365
0.2092
N200001183
0.4343
0.1577
F300000497
WY00000036

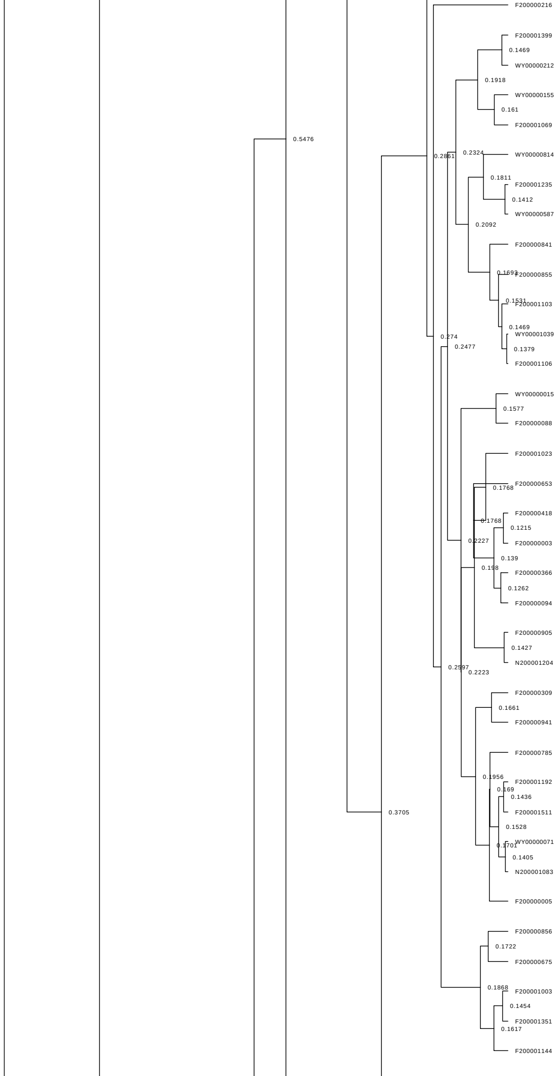
F200000216
F200001399
0.1469
WY00000212
0.1918
WY00000155
0.161
F200001069
0.5476
0.2324
WY00000814
0.2861
0.1811
F200001235
0.1412
WY00000587
0.2092
F200000841
0.1692
F200000855
0.1531
F200001103
0.1469
WY00001039
0.274
0.2477
0.1379
F200001106
WY00000015
0.1577
F200000088
F200001023
F200000653
0.1768
F200000418
0.1768
0.1215
0.2227
F200000003
0.139
0.198
F200000366
0.1262
F200000094
F200000905
0.1427
N200001204
0.2597
0.2223
F200000309
0.1661
F200000941
F200000785
0.1956
F200001192
0.169
0.1436
F200001511
0.3705
0.1528
WY00000071
0.1701
0.1405
N200001083
F200000005
F200000856
0.1722
F200000675
0.1868
F200001003
0.1454
F200001351
0.1617
F200001144

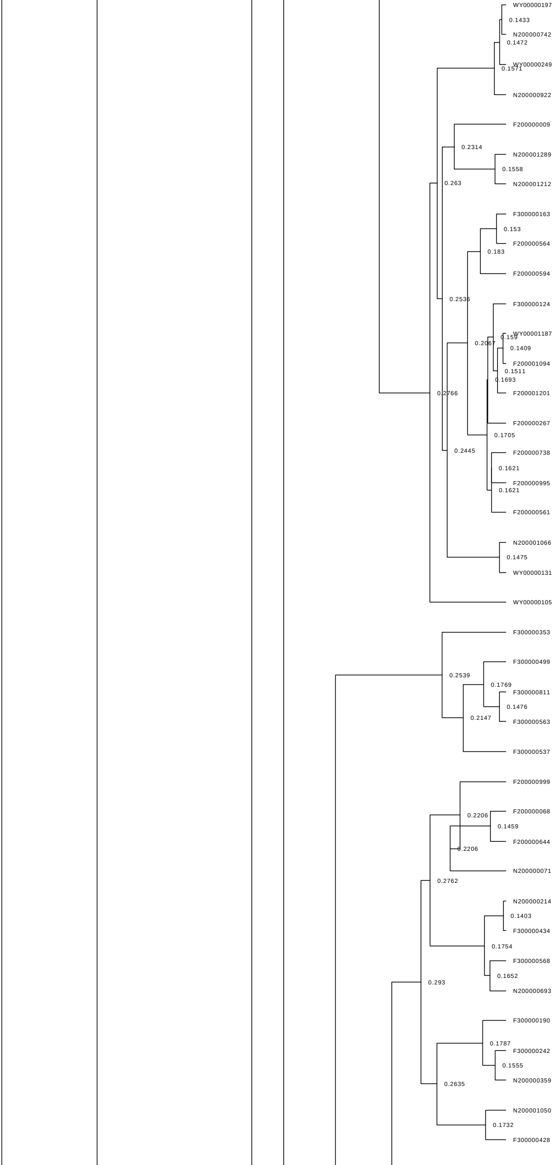
WY00000197
0.1433
N200000742
0.1472
WY00000249
0.1571
N200000922
F200000009
0.2314
N200001289
0.1558
N200001212
0.263
F300000163
0.153
F200000564
0.183
F200000594
0.2536
F300000124
WY00001187
0.159
0.2067
0.1409
F200001094
0.1511
0.1693
0.2766
F200001201
F200000267
0.1705
0.2445
F200000738
0.1621
F200000995
0.1621
F200000561
N200001066
0.1475
WY00000131
WY00000105
F300000353
F300000499
0.2539
0.1769
F300000811
0.1476
0.2147
F300000563
F300000537
F200000999
0.2206
F200000068
0.1459
F200000644
0.2206
N200000071
0.2762
N200000214
0.1403
F300000434
0.1754
F300000568
0.1652
0.293
N200000693
F300000190
0.1787
F300000242
0.1555
N200000359
0.2635
N200001050
0.1732
F300000428

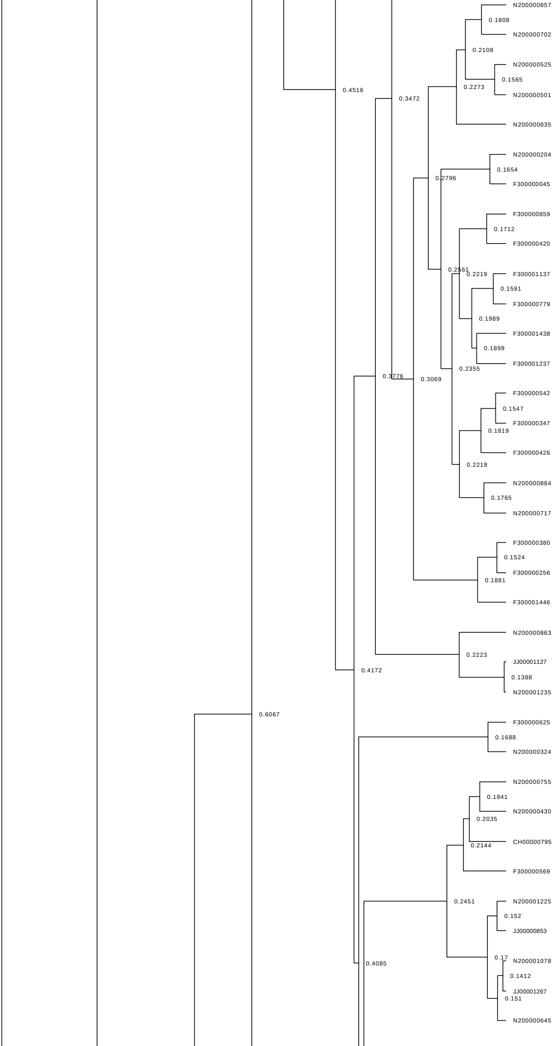

N200000857
0.1808
N200000702
0.2108
N200000525
0.1565
0.2273
N200000501
0.4516
0.3472
N200000835
N200000204
0.1654
0.2796
F300000045
F300000859
0.1712
F300000420
0.2561
0.2219
F300001137
0.1591
F300000779
0.1989
F300001438
0.1899
F300001237
0.2355
0.3776
0.3069
F300000542
0.1547
F300000347
0.1819
F300000426
0.2218
N200000884
0.1765
N200000717
F300000380
0.1524
F300000256
0.1881
F300001446
N200000863
0.2223
JJ00001127
0.4172
0.1388
N200001235
0.6067
F300000625
0.1688
N200000324
N200000755
0.1841
N200000430
0.2035
CH00000795
0.2144
F300000569
0.2451
N200001225
0.152
JJ00000853
0.17
N200001078
0.4085
0.1412
JJ00001267
0.151
N200000645

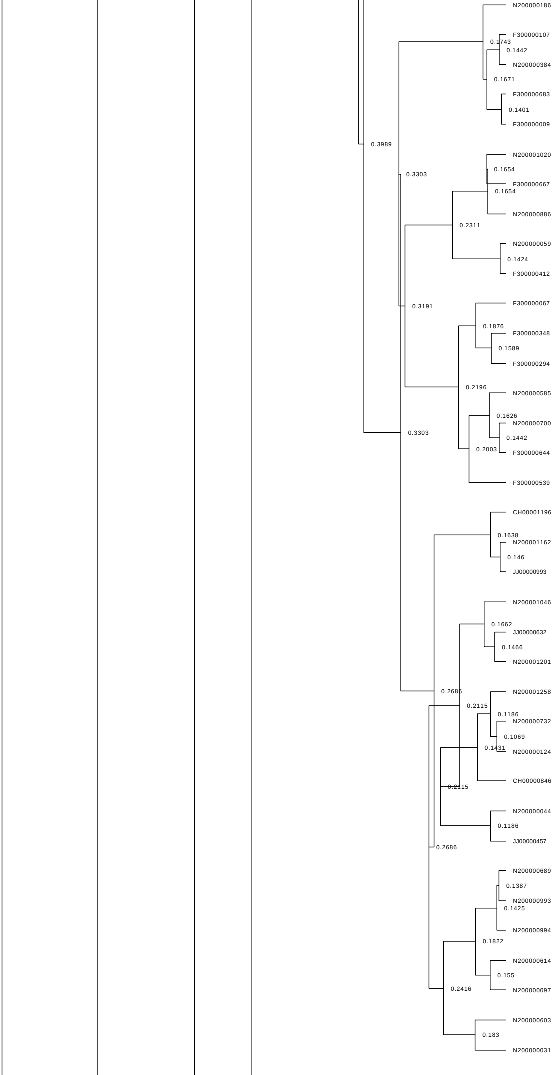
N200000186
F300000107
0.1743
0.1442
N200000384
0.1671
F300000683
0.1401
F300000009
0.3989
N200001020
0.3303
0.1654
F300000667
0.1654
N200000886
0.2311
N200000059
0.1424
F300000412
F300000067
0.3191
0.1876
F300000348
0.1589
F300000294
0.2196
N200000585
0.1626
N200000700
0.3303
0.1442
0.2003
F300000644
F300000539
CH00001196
0.1638
N200001162
0.146
JJ00000993
N200001046
0.1662
JJ00000632
0.1466
N200001201
0.2686
N200001258
0.2115
0.1186
N200000732
0.1069
0.1431
N200000124
CH00000846
0.2115
N200000044
0.1186
JJ00000457
0.2686
N200000689
0.1387
N200000993
0.1425
N200000994
0.1822
N200000614
0.155
0.2416
N200000097
N200000603
0.183
N200000031

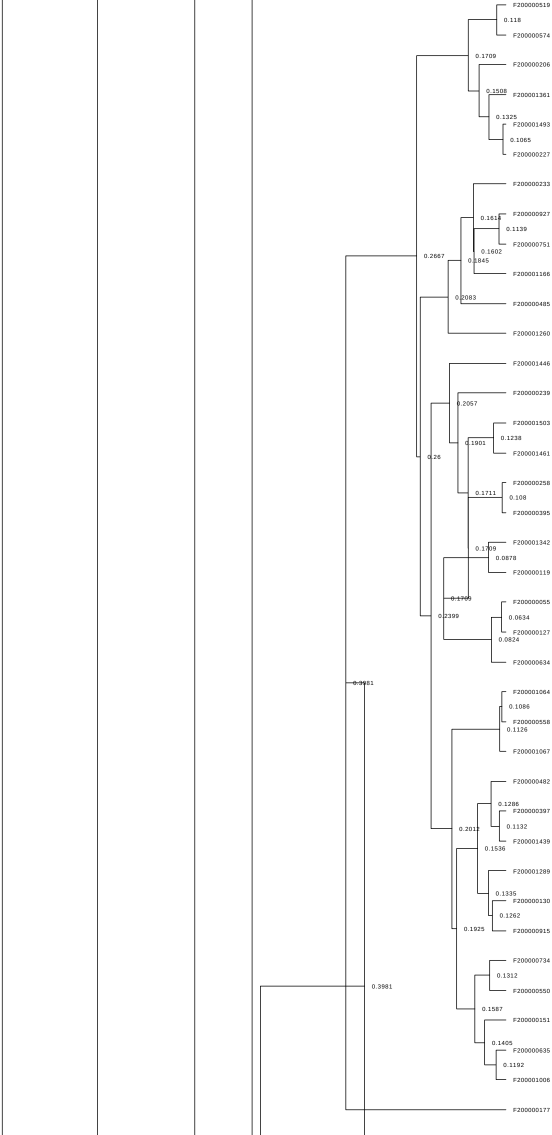

F200000519
0.118
F200000574
0.1709
F200000206
0.1508
F200001361
0.1325
F200001493
0.1065
F200000227
F200000233
F200000927
0.1614
0.1139
F200000751
0.1602
0.2667
0.1845
F200001166
0.2083
F200000485
F200001260
F200001446
F200000239
0.2057
F200001503
0.1238
0.1901
F200001461
0.26
F200000258
0.1711
0.108
F200000395
F200001342
0.1709
0.0878
F200000119
0.1709
F200000055
0.2399
0.0634
F200000127
0.0824
F200000634
0.3981
F200001064
0.1086
F200000558
0.1126
F200001067
F200000482
0.1286
F200000397
0.1132
0.2012
F200001439
0.1536
F200001289
0.1335
F200000130
0.1262
0.1925
F200000915
F200000734
0.1312
0.3981
F200000550
0.1587
F200000151
0.1405
F200000635
0.1192
F200001006
F200000177

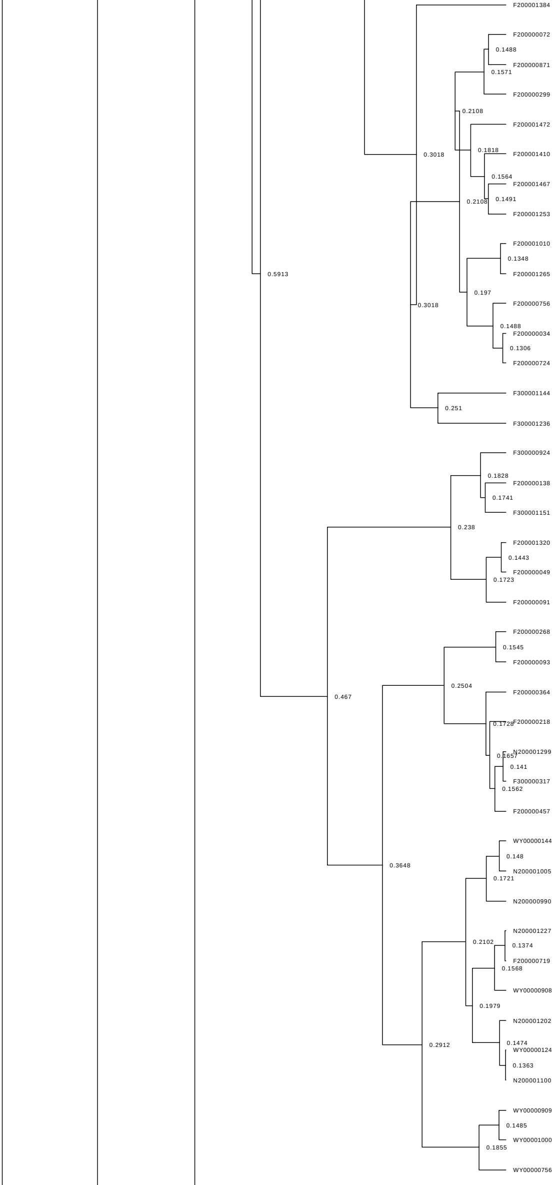
F200001384
F200000072
0.1488
F200000871
0.1571
F200000299
0.2108
F200001472
0.1818
F200001410
0.3018
0.1564
F200001467
0.1491
0.2108
F200001253
F200001010
0.1348
F200001265
0.5913
0.197
0.3018
F200000756
0.1488
F200000034
0.1306
F200000724
F300001144
0.251
F300001236
F300000924
0.1828
F200000138
0.1741
F300001151
0.238
F200001320
0.1443
F200000049
0.1723
F200000091
F200000268
0.1545
F200000093
0.2504
F200000364
0.467
0.1728
F200000218
N200001299
0.1657
0.141
F300000317
0.1562
F200000457
WY00000144
0.148
N200001005
0.1721
0.3648
N200000990
N200001227
0.2102
0.1374
F200000719
0.1568
WY00000908
0.1979
N200001202
0.1474
WY00000124
0.2912
0.1363
N200001100
WY00000909
0.1485
WY00001000
0.1855
WY00000756

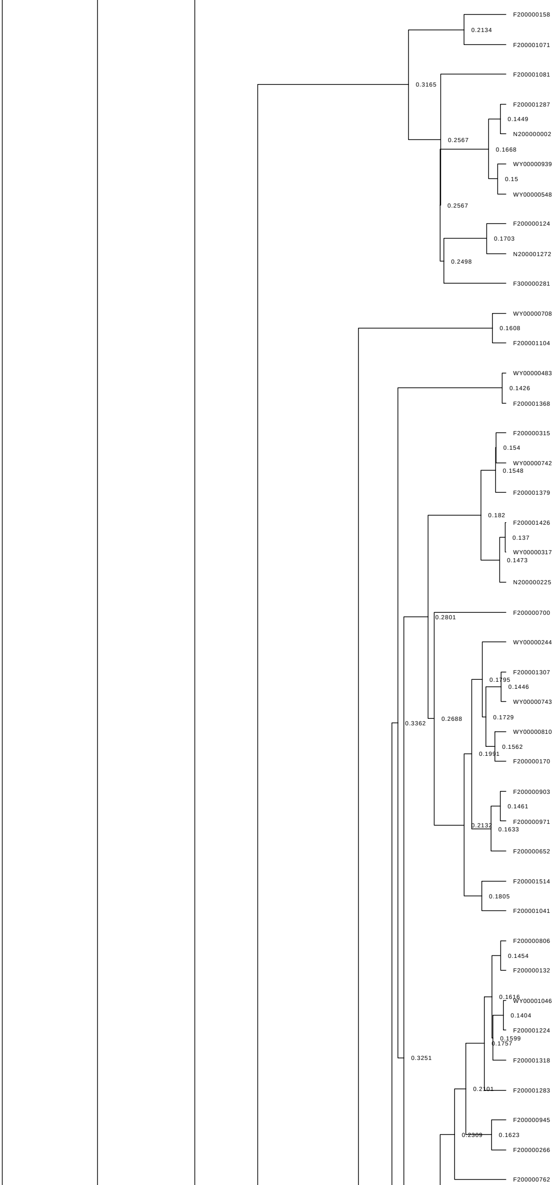
F200000158
0.2134
F200001071
F200001081
0.3165
F200001287
0.1449
N200000002
0.2567
0.1668
WY00000939
0.15
WY00000548
0.2567
F200000124
0.1703
N200001272
0.2498
F300000281
WY00000708
0.1608
F200001104
WY00000483
0.1426
F200001368
F200000315
0.154
WY00000742
0.1548
F200001379
0.182
F200001426
0.137
WY00000317
0.1473
N200000225
F200000700
0.2801
WY00000244
F200001307
0.1795
0.1446
WY00000743
0.1729
0.2688
0.3362
WY00000810
0.1562
0.1991
F200000170
F200000903
0.1461
F200000971
0.2132
0.1633
F200000652
F200001514
0.1805
F200001041
F200000806
0.1454
F200000132
0.1616
WY00001046
0.1404
F200001224
0.1599
0.1757
0.3251
F200001318
0.2101
F200001283
F200000945
0.2309
0.1623
F200000266
F200000762

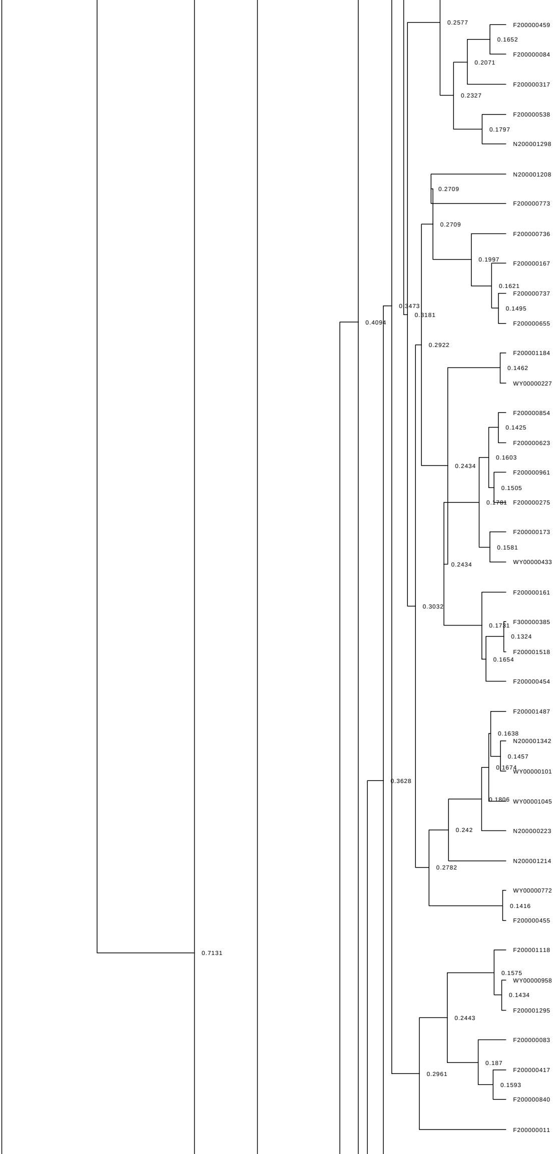

0.2577
F200000459
0.1652
F200000084
0.2071
F200000317
0.2327
F200000538
0.1797
N200001298
N200001208
0.2709
F200000773
0.2709
F200000736
0.1997
F200000167
0.1621
F200000737
0.1495
F200000655
0.3473
0.3181
0.4094
0.2922
F200001184
0.1462
WY00000227
F200000854
0.1425
F200000623
0.1603
F200000961
0.1505
0.1781
F200000275
0.2434
F200000173
0.1581
WY00000433
0.2434
F200000161
0.3032
0.1731
F300000385
0.1324
F200001518
0.1654
F200000454
F200001487
0.1638
N200001342
0.1457
0.1674
WY00000101
0.3628
0.1806
WY00001045
0.242
N200000223
N200001214
0.2782
WY00000772
0.1416
F200000455
0.7131
F200001118
0.1575
WY00000958
0.1434
F200001295
0.2443
F200000083
0.187
F200000417
0.2961
0.1593
F200000840
F200000011

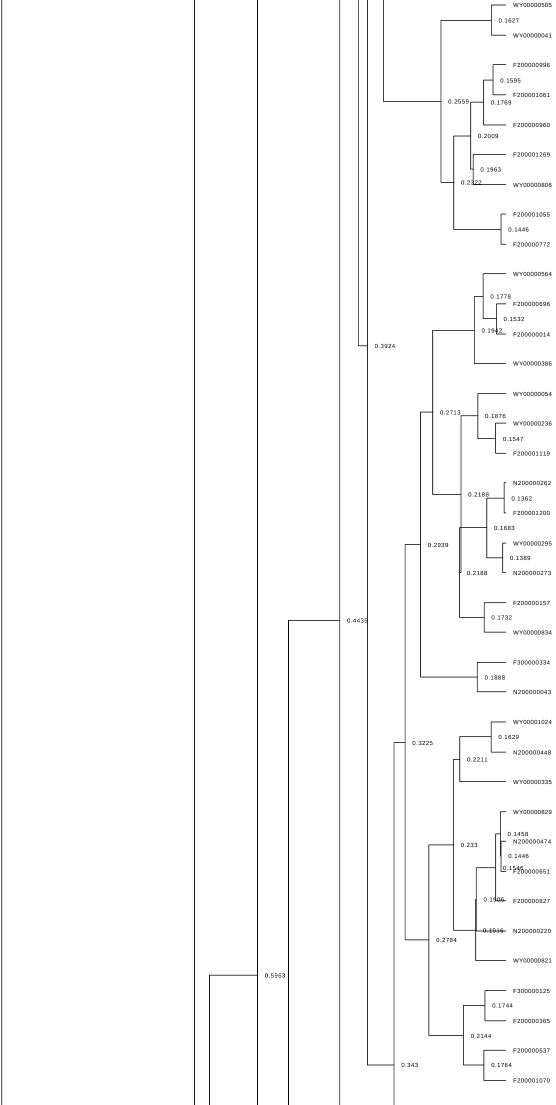
WY00000505
0.1627
WY00000041
F200000996
0.1595
F200001061
0.2559
0.1769
F200000960
0.2009
F200001269
0.1963
0.2322
WY00000806
F200001055
0.1446
F200000772
WY00000564
0.1778
F200000696
0.1532
0.1942
F200000014
0.3924
WY00000386
WY00000054
0.2713
0.1876
WY00000236
0.1547
F200001119
N200000262
0.2188
0.1362
F200001200
0.1683
WY00000295
0.2939
0.1389
0.2188
N200000273
F200000157
0.4435
0.1732
WY00000834
F300000334
0.1888
N200000043
WY00001024
0.1629
0.3225
N200000448
0.2211
WY00000335
WY00000829
0.1458
N200000474
0.233
0.1446
0.1546
F200000651
0.1906
F200000827
0.1916
N200000220
0.2784
WY00000821
0.5963
F300000125
0.1744
F200000365
0.2144
F200000537
0.343
0.1764
F200001070

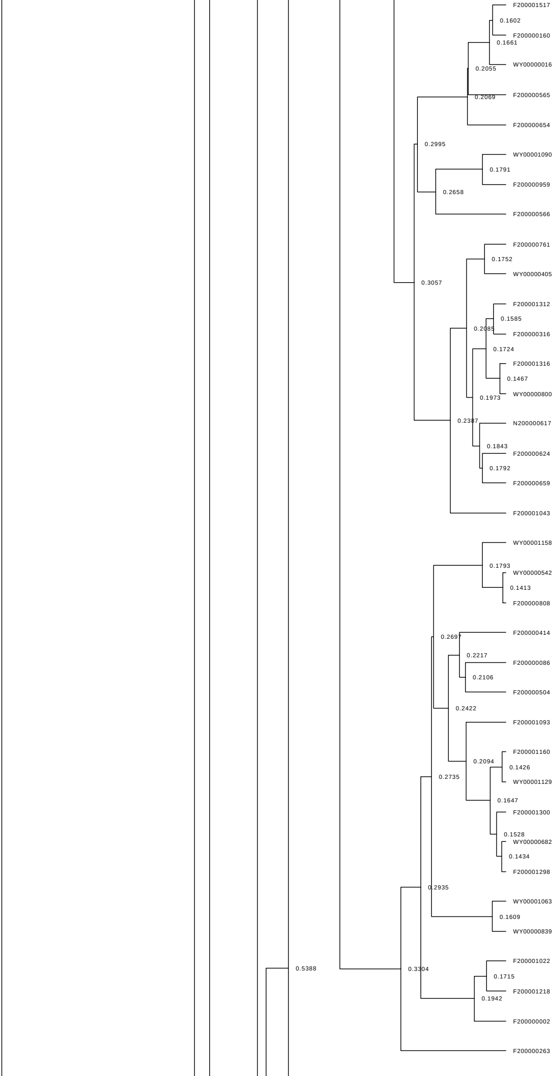
F200001517
0.1602
F200000160
0.1661
WY00000016
0.2055
F200000565
0.2069
F200000654
0.2995
WY00001090
0.1791
F200000959
0.2658
F200000566
F200000761
0.1752
WY00000405
0.3057
F200001312
0.1585
0.2085
F200000316
0.1724
F200001316
0.1467
WY00000800
0.1973
0.2387
N200000617
0.1843
F200000624
0.1792
F200000659
F200001043
WY00001158
0.1793
WY00000542
0.1413
F200000808
F200000414
0.2697
0.2217
F200000086
0.2106
F200000504
0.2422
F200001093
F200001160
0.2094
0.1426
WY00001129
0.2735
0.1647
F200001300
0.1528
WY00000682
0.1434
F200001298
0.2935
WY00001063
0.1609
WY00000839
F200001022
0.5388
0.3304
0.1715
F200001218
0.1942
F200000002
F200000263

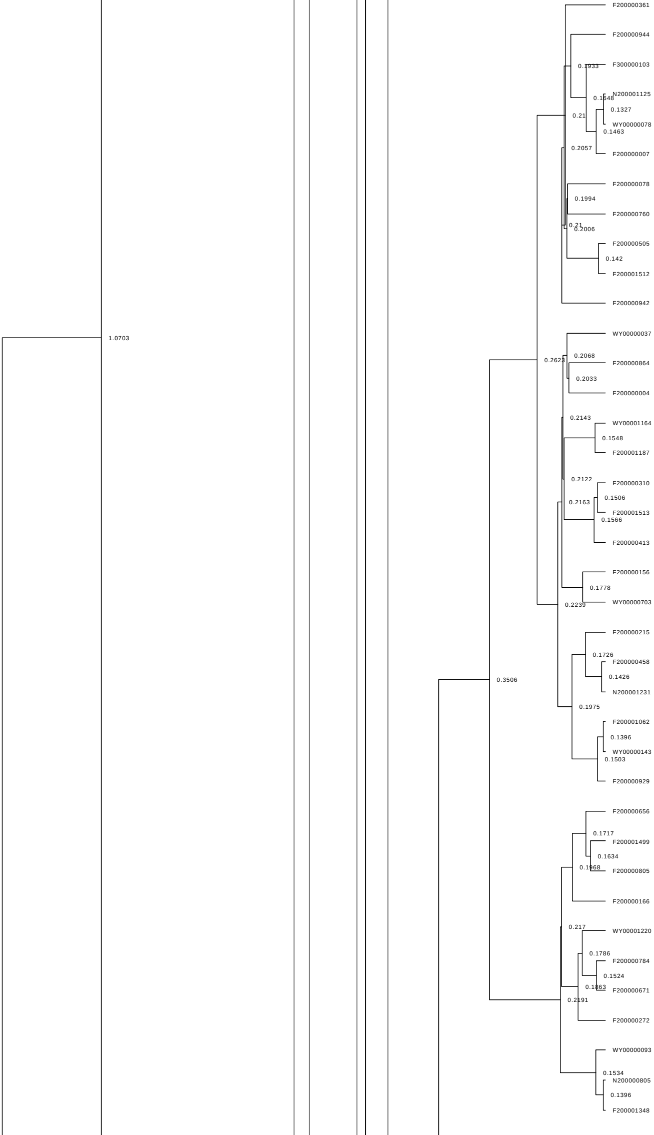
F200000361
F200000944
0.1933
F300000103
N200001125
0.1548
0.1327
0.21
WY00000078
0.1463
0.2057
F200000007
F200000078
0.1994
F200000760
0.21
0.2006
F200000505
0.142
F200001512
F200000942
1.0703
WY00000037
0.2068
0.2623
F200000864
0.2033
F200000004
0.2143
WY00001164
0.1548
F200001187
0.2122
F200000310
0.1506
0.2163
F200001513
0.1566
F200000413
F200000156
0.1778
WY00000703
0.2239
F200000215
0.1726
F200000458
0.3506
0.1426
N200001231
0.1975
F200001062
0.1396
WY00000143
0.1503
F200000929
F200000656
0.1717
F200001499
0.1634
0.1968
F200000805
F200000166
0.217
WY00001220
0.1786
F200000784
0.1524
0.1863
F200000671
0.2191
F200000272
WY00000093
0.1534
N200000805
0.1396
F200001348

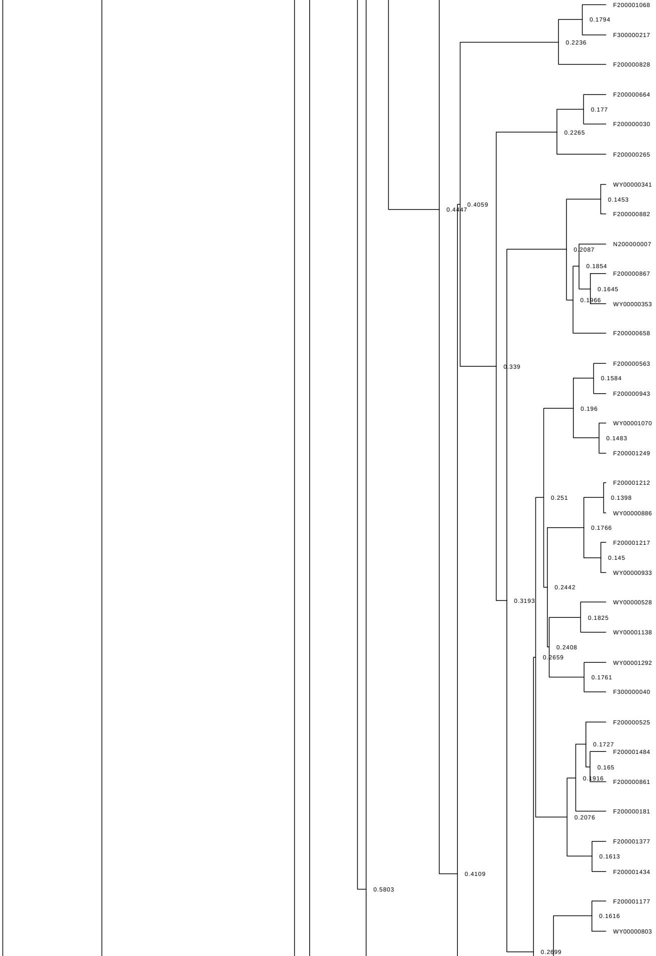
F200001068
0.1794
F300000217
0.2236
F200000828
F200000664
0.177
F200000030
0.2265
F200000265
WY00000341
0.1453
F200000882
0.4059
0.4447
N200000007
0.2087
0.1854
F200000867
0.1645
WY00000353
0.1966
F200000658
0.339
F200000563
0.1584
F200000943
0.196
WY00001070
0.1483
F200001249
F200001212
0.251
0.1398
WY00000886
0.1766
F200001217
0.145
WY00000933
0.2442
0.3193
WY00000528
0.1825
WY00001138
0.2408
0.2659
WY00001292
0.1761
F300000040
F200000525
0.1727
F200001484
0.165
0.1916
F200000861
F200000181
0.2076
F200001377
0.1613
F200001434
0.4109
0.5803
F200001177
0.1616
WY00000803
0.2699

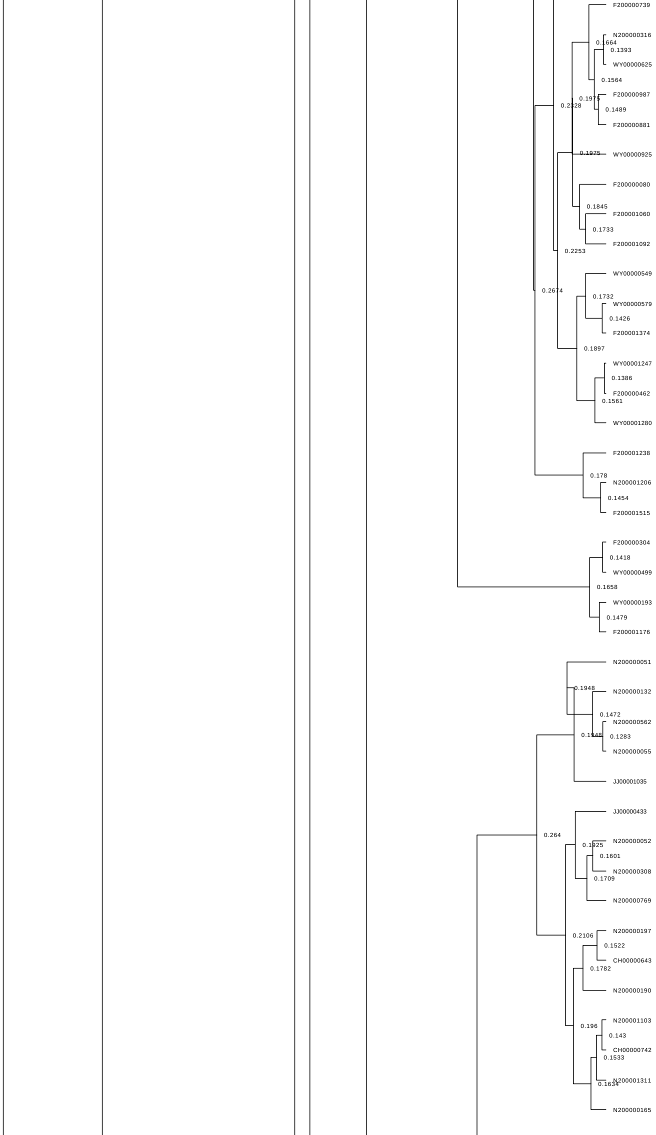
F200000739
N200000316
0.1664
0.1393
WY00000625
0.1564
F200000987
0.1975
0.2328
0.1489
F200000881
0.1975
WY00000925
F200000080
0.1845
F200001060
0.1733
F200001092
0.2253
WY00000549
0.2674
0.1732
WY00000579
0.1426
F200001374
0.1897
WY00001247
0.1386
F200000462
0.1561
WY00001280
F200001238
0.178
N200001206
0.1454
F200001515
F200000304
0.1418
WY00000499
0.1658
WY00000193
0.1479
F200001176
N200000051
0.1948
N200000132
0.1472
N200000562
0.1948
0.1283
N200000055
JJ00001035
JJ00000433
0.264
N200000052
0.1925
0.1601
N200000308
0.1709
N200000769
N200000197
0.2106
0.1522
CH00000643
0.1782
N200000190
N200001103
0.196
0.143
CH00000742
0.1533
N200001311
0.1634
N200000165

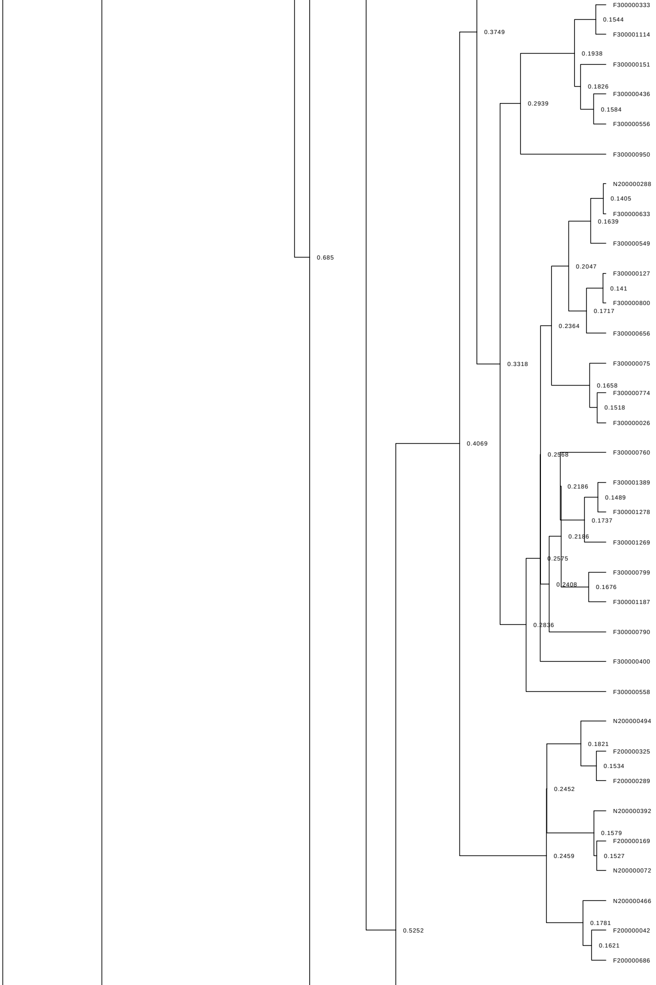
F300000333
0.1544
0.3749
F300001114
0.1938
F300000151
0.1826
F300000436
0.2939
0.1584
F300000556
F300000950
N200000288
0.1405
F300000633
0.1639
F300000549
0.685
0.2047
F300000127
0.141
F300000800
0.1717
0.2364
F300000656
F300000075
0.3318
0.1658
F300000774
0.1518
F300000026
0.4069
F300000760
0.2568
0.2186
F300001389
0.1489
F300001278
0.1737
0.2186
F300001269
0.2575
F300000799
0.2408
0.1676
F300001187
0.2836
F300000790
F300000400
F300000558
N200000494
0.1821
F200000325
0.1534
F200000289
0.2452
N200000392
0.1579
F200000169
0.2459
0.1527
N200000072
N200000466
0.1781
0.5252
F200000042
0.1621
F200000686

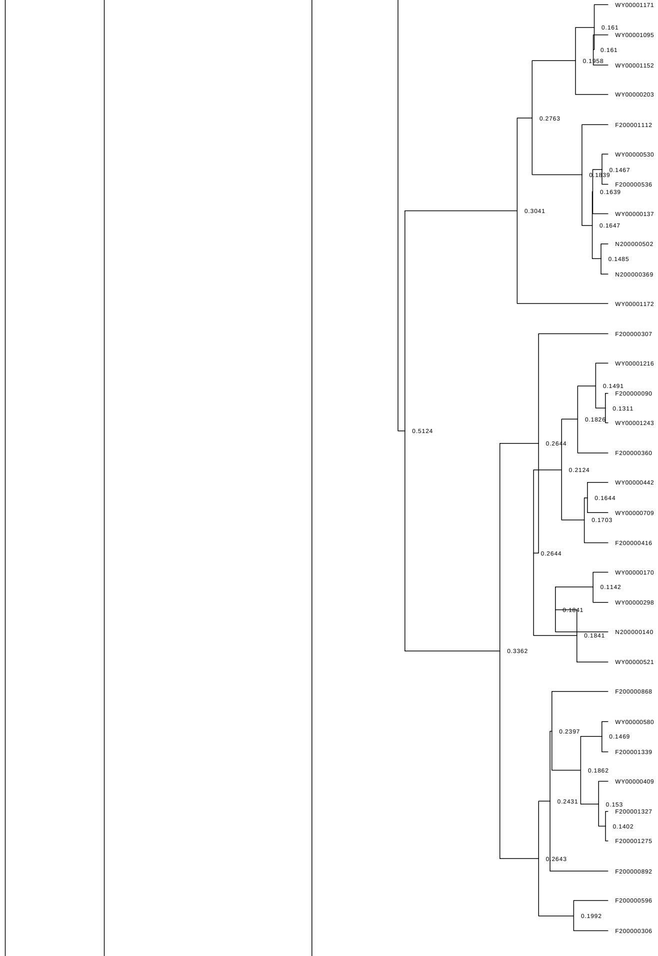
WY00001171
0.161
WY00001095
0.161
0.1958
WY00001152
WY00000203
0.2763
F200001112
WY00000530
0.1467
0.1839
F200000536
0.1639
0.3041
WY00000137
0.1647
N200000502
0.1485
N200000369
WY00001172
F200000307
WY00001216
0.1491
F200000090
0.1311
0.1826
WY00001243
0.5124
0.2644
F200000360
0.2124
WY00000442
0.1644
WY00000709
0.1703
F200000416
0.2644
WY00000170
0.1142
WY00000298
0.1841
N200000140
0.1841
0.3362
WY00000521
F200000868
WY00000580
0.2397
0.1469
F200001339
0.1862
WY00000409
0.2431
0.153
F200001327
0.1402
F200001275
0.2643
F200000892
F200000596
0.1992
F200000306

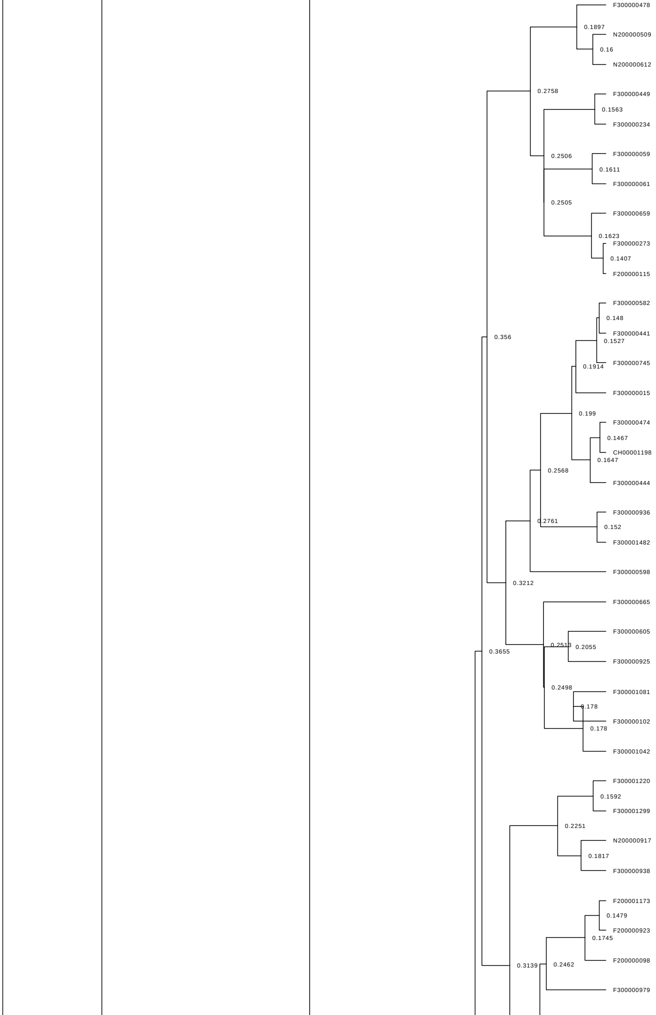
F300000478
0.1897
N200000509
0.16
N200000612
0.2758
F300000449
0.1563
F300000234
0.2506
F300000059
0.1611
F300000061
0.2505
F300000659
0.1623
F300000273
0.1407
F200000115
F300000582
0.148
F300000441
0.1527
F300000745
0.1914
F300000015
0.199
F300000474
0.1467
CH00001198
0.1647
F300000444
0.356
0.2568
F300000936
0.2761
0.152
F300001482
F300000598
0.3212
F300000665
F300000605
0.2513
0.2055
F300000925
0.3655
0.2498
F300001081
0.178
F300000102
0.178
F300001042
F300001220
0.1592
F300001299
0.2251
N200000917
0.1817
F300000938
F200001173
0.1479
F200000923
0.1745
F200000098
0.3139
0.2462
F300000979

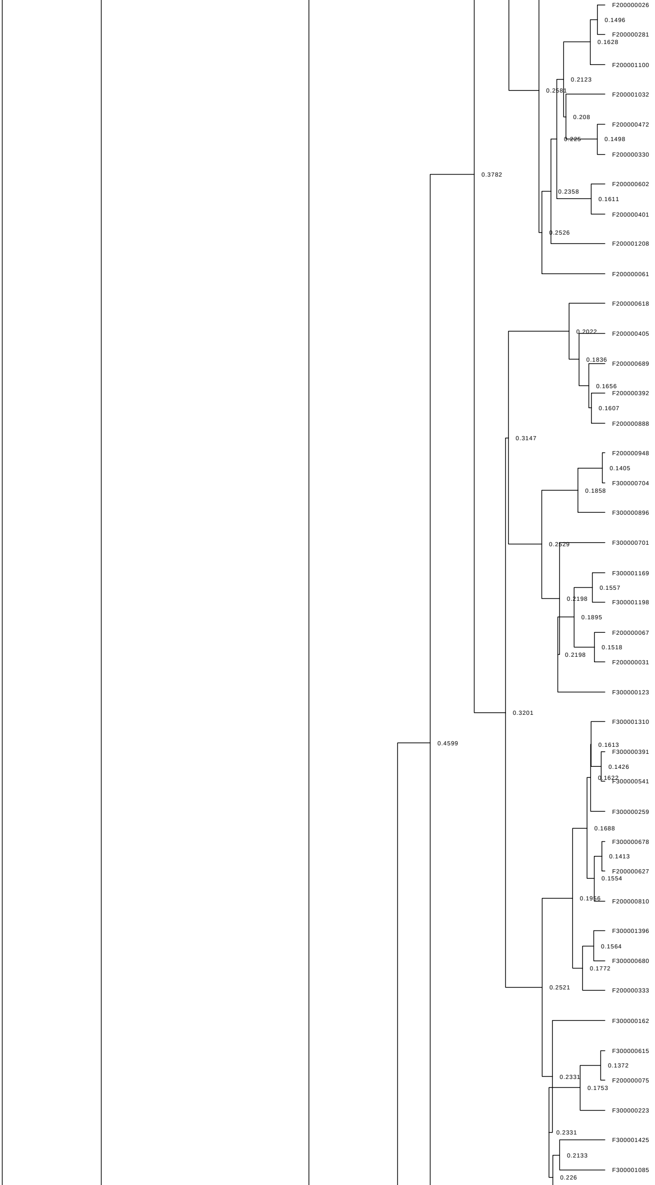
F200000026
0.1496
F200000281
0.1628
F200001100
0.2123
0.2581
F200001032
0.208
F200000472
0.225
0.1498
F200000330
0.3782
F200000602
0.2358
0.1611
F200000401
0.2526
F200001208
F200000061
F200000618
0.2022
F200000405
0.1836
F200000689
0.1656
F200000392
0.1607
F200000888
0.3147
F200000948
0.1405
F300000704
0.1858
F300000896
0.2529
F300000701
F300001169
0.1557
0.2198
F300001198
0.1895
F200000067
0.1518
0.2198
F200000031
F300000123
0.3201
F300001310
0.4599
0.1613
F300000391
0.1426
0.1622
F300000541
F300000259
0.1688
F300000678
0.1413
F200000627
0.1554
0.1956
F200000810
F300001396
0.1564
F300000680
0.1772
0.2521
F200000333
F300000162
F300000615
0.1372
0.2331
F200000075
0.1753
F300000223
0.2331
F300001425
0.2133
F300001085
0.226

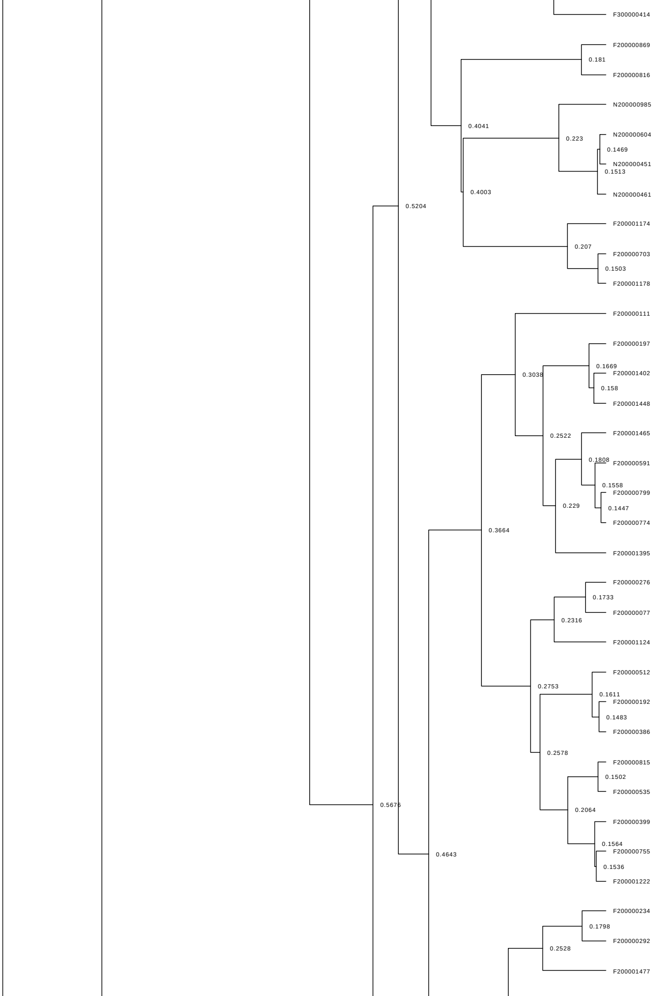
F300000414
F200000869
0.181
F200000816
N200000985
0.4041
N200000604
0.223
0.1469
N200000451
0.1513
0.4003
N200000461
0.5204
F200001174
0.207
F200000703
0.1503
F200001178
F200000111
F200000197
0.1669
F200001402
0.3038
0.158
F200001448
F200001465
0.2522
0.1808
F200000591
0.1558
F200000799
0.229
0.1447
F200000774
0.3664
F200001395
F200000276
0.1733
F200000077
0.2316
F200001124
F200000512
0.2753
0.1611
F200000192
0.1483
F200000386
0.2578
F200000815
0.1502
F200000535
0.5676
0.2064
F200000399
0.1564
F200000755
0.4643
0.1536
F200001222
F200000234
0.1798
F200000292
0.2528
F200001477

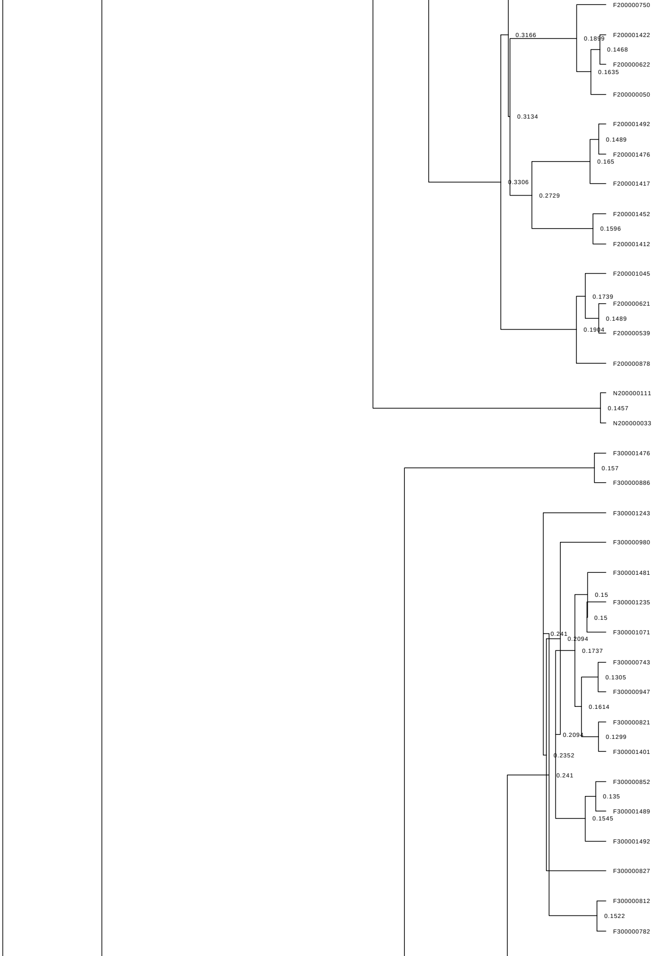

F200000750
0.3166
0.1899
F200001422
0.1468
F200000622
0.1635
F200000050
0.3134
F200001492
0.1489
F200001476
0.165
0.3306
F200001417
0.2729
F200001452
0.1596
F200001412
F200001045
0.1739
F200000621
0.1489
0.1904
F200000539
F200000878
N200000111
0.1457
N200000033
F300001476
0.157
F300000886
F300001243
F300000980
F300001481
0.15
F300001235
0.15
F300001071
0.241
0.2094
0.1737
F300000743
0.1305
F300000947
0.1614
F300000821
0.2094
0.1299
F300001401
0.2352
0.241
F300000852
0.135
F300001489
0.1545
F300001492
F300000827
F300000812
0.1522
F300000782

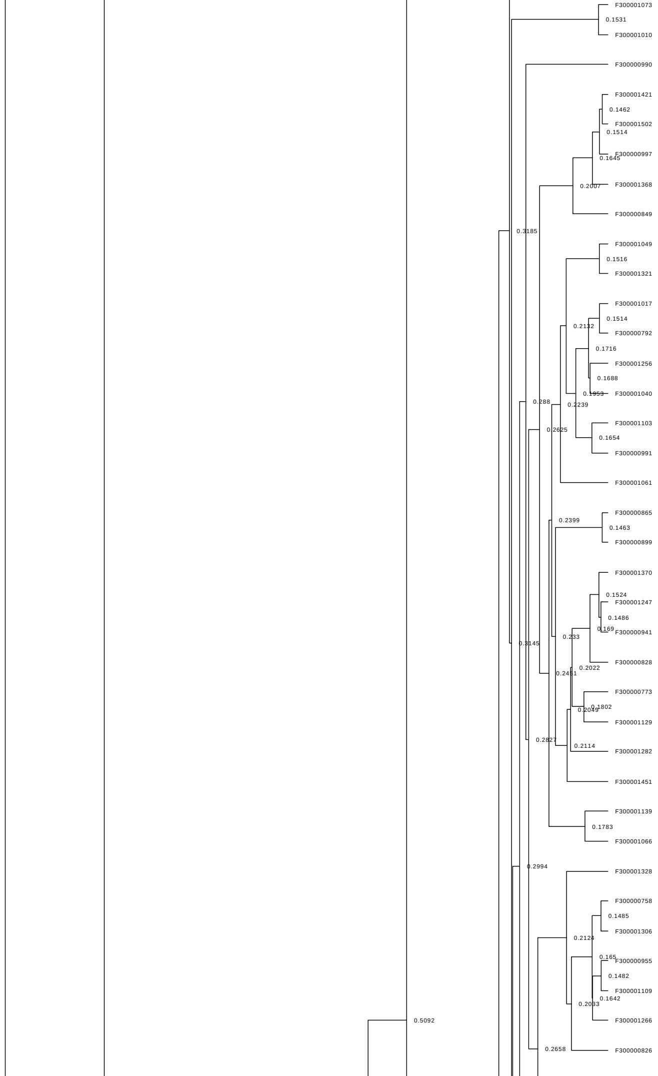
F300001073
0.1531
F300001010
F300000990
F300001421
0.1462
F300001502
0.1514
F300000997
0.1645
F300001368
0.2007
F300000849
0.3185
F300001049
0.1516
F300001321
F300001017
0.1514
0.2132
F300000792
0.1716
F300001256
0.1688
F300001040
0.1953
0.288
0.2239
F300001103
0.2625
0.1654
F300000991
F300001061
F300000865
0.2399
0.1463
F300000899
F300001370
0.1524
F300001247
0.1486
0.169
F300000941
0.233
0.3145
F300000828
0.2022
0.2451
F300000773
0.1802
0.2049
F300001129
0.2827
0.2114
F300001282
F300001451
F300001139
0.1783
F300001066
0.2994
F300001328
F300000758
0.1485
F300001306
0.2124
0.165
F300000955
0.1482
F300001109
0.1642
0.2033
F300001266
0.5092
0.2658
F300000826

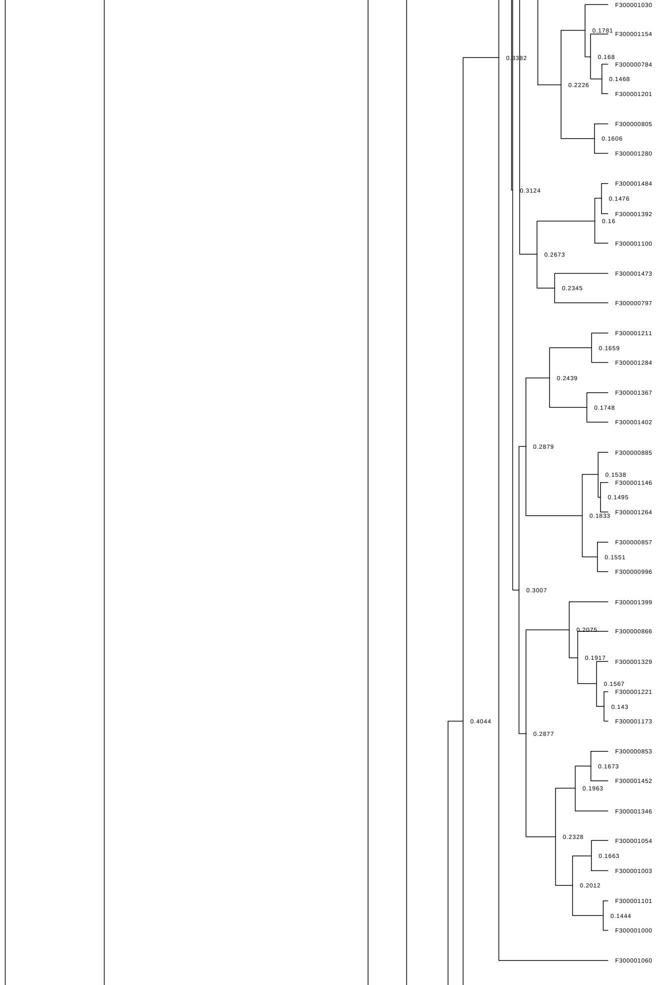

F300001030
0.1781
F300001154
0.168
F300000784
0.1468
F300001201
0.3382
0.2226
F300000805
0.1606
F300001280
0.3124
F300001484
0.1476
F300001392
0.16
F300001100
0.2673
F300001473
0.2345
F300000797
F300001211
0.1659
F300001284
0.2439
F300001367
0.1748
F300001402
0.2879
F300000885
0.1538
F300001146
0.1495
F300001264
0.1833
F300000857
0.1551
F300000996
0.3007
F300001399
0.2075
F300000866
0.1917
F300001329
0.1567
F300001221
0.143
F300001173
0.4044
0.2877
F300000853
0.1673
F300001452
0.1963
F300001346
0.2328
F300001054
0.1663
F300001003
0.2012
F300001101
0.1444
F300001000
F300001060

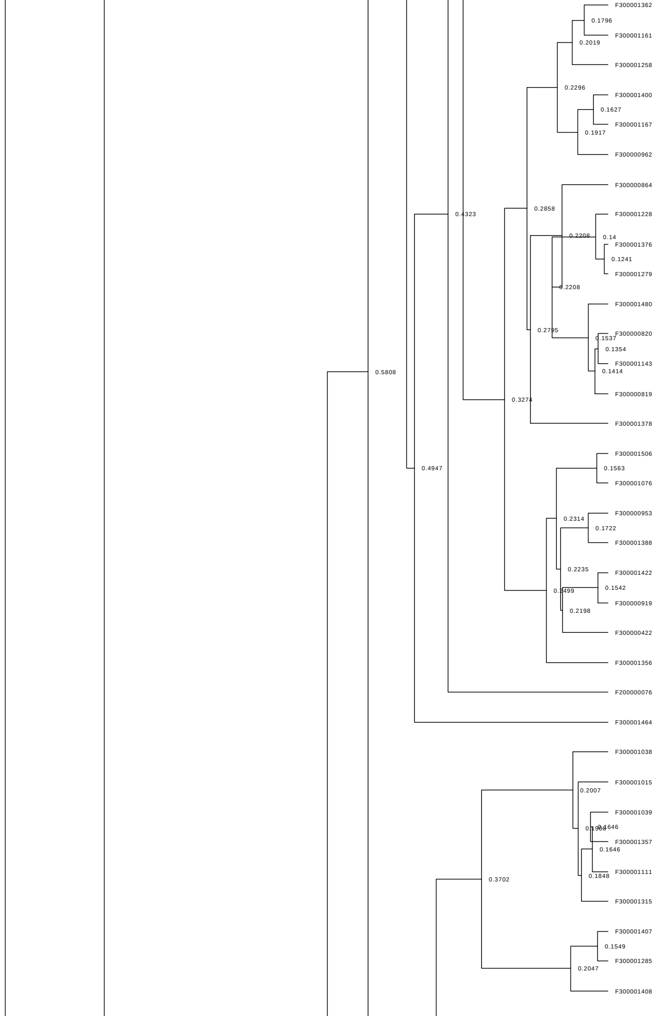

F300001362
0.1796
F300001161
0.2019
F300001258
0.2296
F300001400
0.1627
F300001167
0.1917
F300000962
F300000864
0.4323
F300001228
0.2858
0.2208
0.14
F300001376
0.1241
F300001279
0.2208
F300001480
0.2795
F300000820
0.1537
0.1354
F300001143
0.5808
0.1414
F300000819
0.3274
F300001378
F300001506
0.4947
0.1563
F300001076
F300000953
0.2314
0.1722
F300001388
0.2235
F300001422
0.1542
0.2499
F300000919
0.2198
F300000422
F300001356
F200000076
F300001464
F300001038
F300001015
0.2007
F300001039
0.1646
F300001357
0.1646
F300001111
0.3702
0.1848
F300001315
F300001407
0.1549
F300001285
0.2047
F300001408

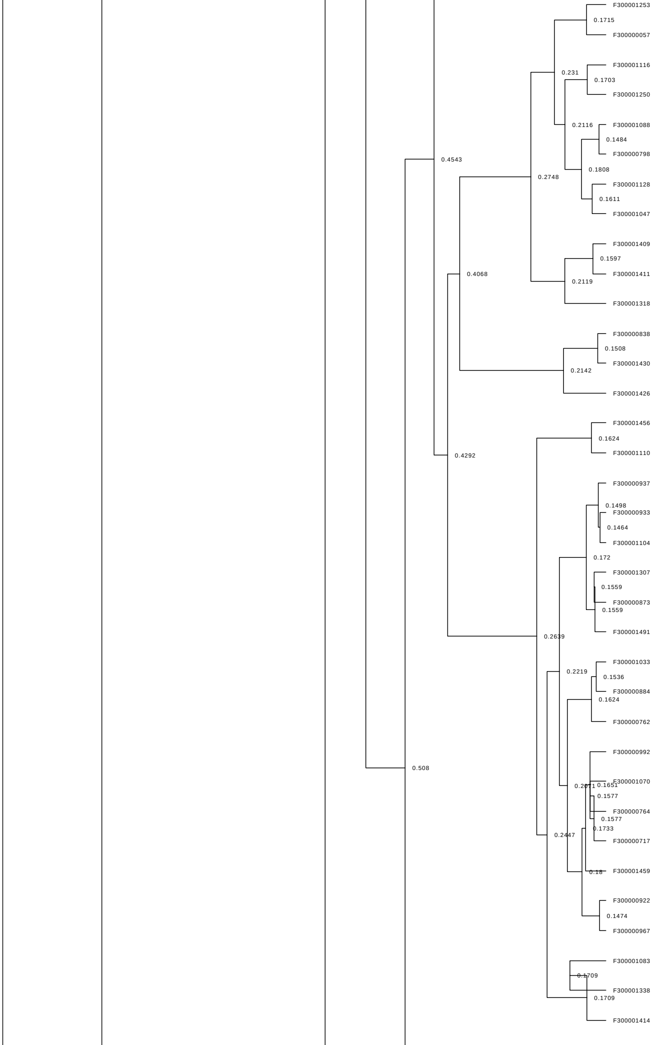
F300001253
0.1715
F300000057
F300001116
0.231
0.1703
F300001250
0.2116
F300001088
0.1484
F300000798
0.4543
0.1808
0.2748
F300001128
0.1611
F300001047
F300001409
0.1597
0.4068
F300001411
0.2119
F300001318
F300000838
0.1508
F300001430
0.2142
F300001426
F300001456
0.1624
F300001110
0.4292
F300000937
0.1498
F300000933
0.1464
F300001104
0.172
F300001307
0.1559
F300000873
0.1559
F300001491
0.2639
F300001033
0.2219
0.1536
F300000884
0.1624
F300000762
F300000992
0.508
F300001070
0.1651
0.1577
F300000764
0.1577
0.1733
0.2447
F300000717
0.18
F300001459
F300000922
0.1474
F300000967
F300001083
0.1709
F300001338
0.1709
F300001414

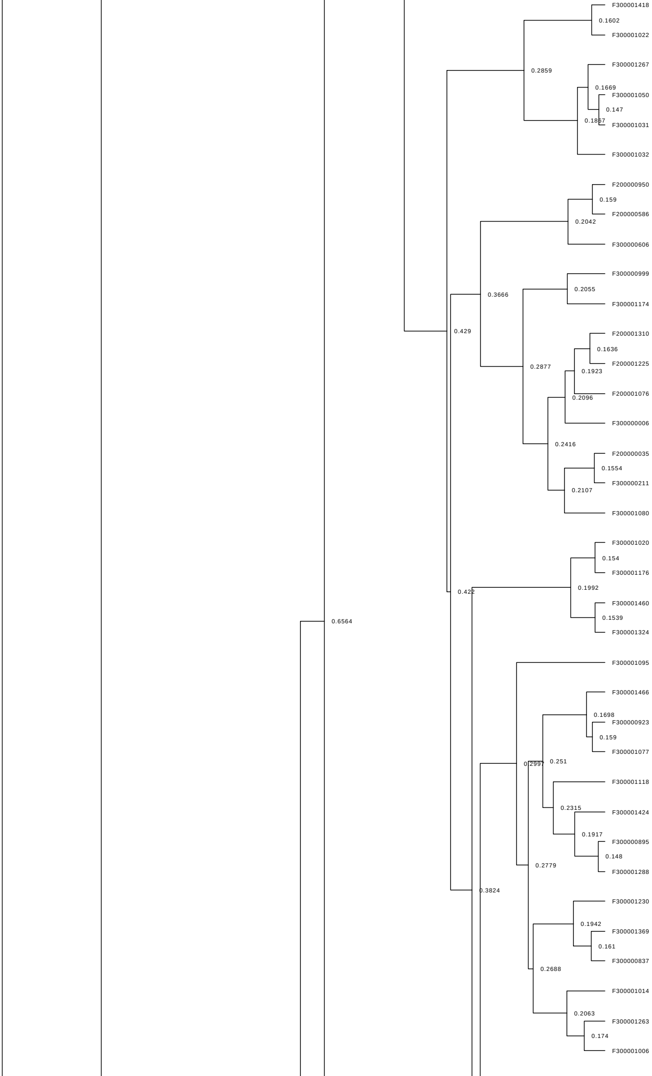
F300001418
0.1602
F300001022
F300001267
0.2859
0.1669
F300001050
0.147
0.1867
F300001031
F300001032
F200000950
0.159
F200000586
0.2042
F300000606
F300000999
0.2055
0.3666
F300001174
0.429
F200001310
0.1636
F200001225
0.2877
0.1923
F200001076
0.2096
F300000006
0.2416
F200000035
0.1554
F300000211
0.2107
F300001080
F300001020
0.154
F300001176
0.1992
0.422
F300001460
0.6564
0.1539
F300001324
F300001095
F300001466
0.1698
F300000923
0.159
F300001077
0.2997
0.251
F300001118
0.2315
F300001424
0.1917
F300000895
0.148
0.2779
F300001288
0.3824
F300001230
0.1942
F300001369
0.161
F300000837
0.2688
F300001014
0.2063
F300001263
0.174
F300001006

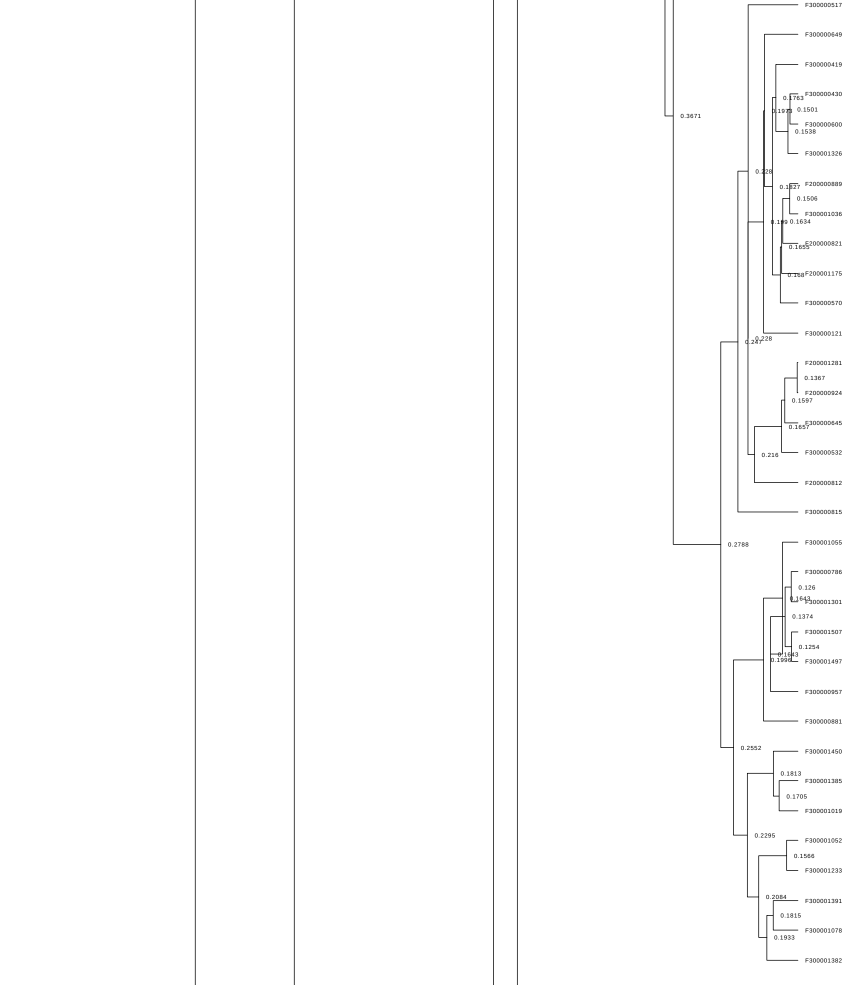

F300000517
F300000649
F300000419
F300000430
0.1763
0.1973
0.1501
F300000600
0.1538
F300001326
0.3671
0.228
F200000889
0.1506
F300001036
0.1634
F200000821
0.1655
F200001175
0.168
F300000570
F300000121
0.228
0.247
F200001281
0.1367
F200000924
0.1597
F300000645
0.1657
F300000532
0.216
F200000812
F300000815
F300001055
0.2788
F300000786
0.126
0.1643
F300001301
0.1374
F300001507
0.1254
0.1643
0.1996
F300001497
F300000957
F300000881
0.2552
F300001450
0.1813
F300001385
0.1705
F300001019
0.2295
F300001052
0.1566
F300001233
0.2084
F300001391
0.1815
F300001078
0.1933
F300001382

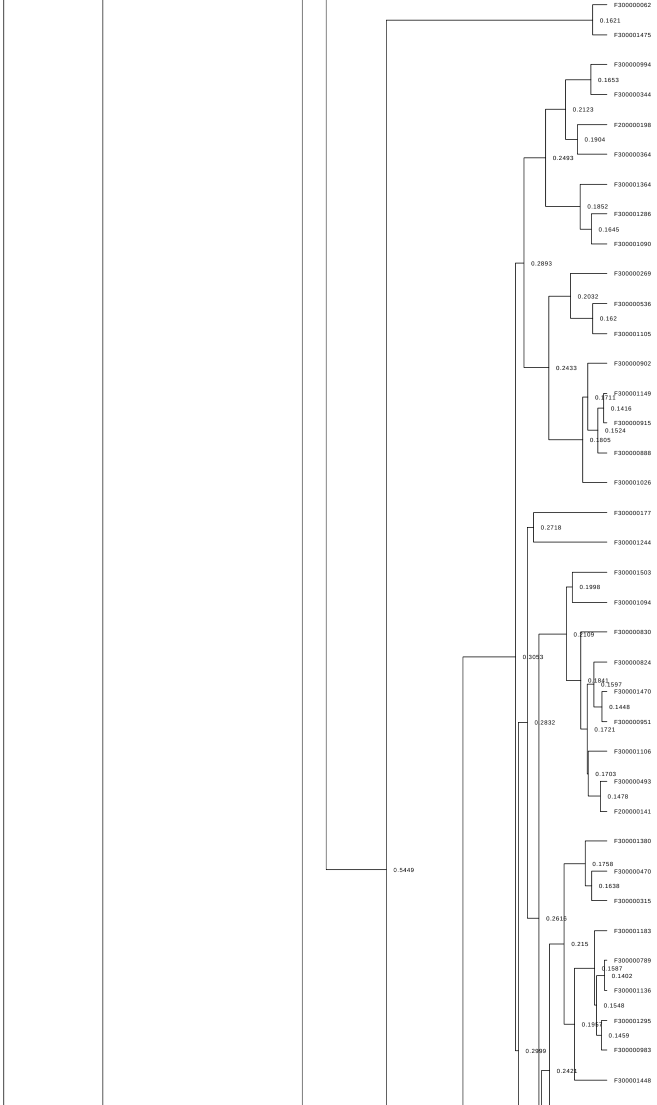
F300000062
0.1621
F300001475
F300000994
0.1653
F300000344
0.2123
F200000198
0.1904
F300000364
0.2493
F300001364
0.1852
F300001286
0.1645
F300001090
0.2893
F300000269
0.2032
F300000536
0.162
F300001105
F300000902
0.2433
F300001149
0.1711
0.1416
F300000915
0.1524
0.1805
F300000888
F300001026
F300000177
0.2718
F300001244
F300001503
0.1998
F300001094
F300000830
0.2109
0.3053
F300000824
0.1841
0.1597
F300001470
0.1448
F300000951
0.2832
0.1721
F300001106
0.1703
F300000493
0.1478
F200000141
F300001380
0.1758
F300000470
0.5449
0.1638
F300000315
0.2616
F300001183
0.215
F300000789
0.1587
0.1402
F300001136
0.1548
F300001295
0.1957
0.1459
F300000983
0.2999
0.2421
F300001448

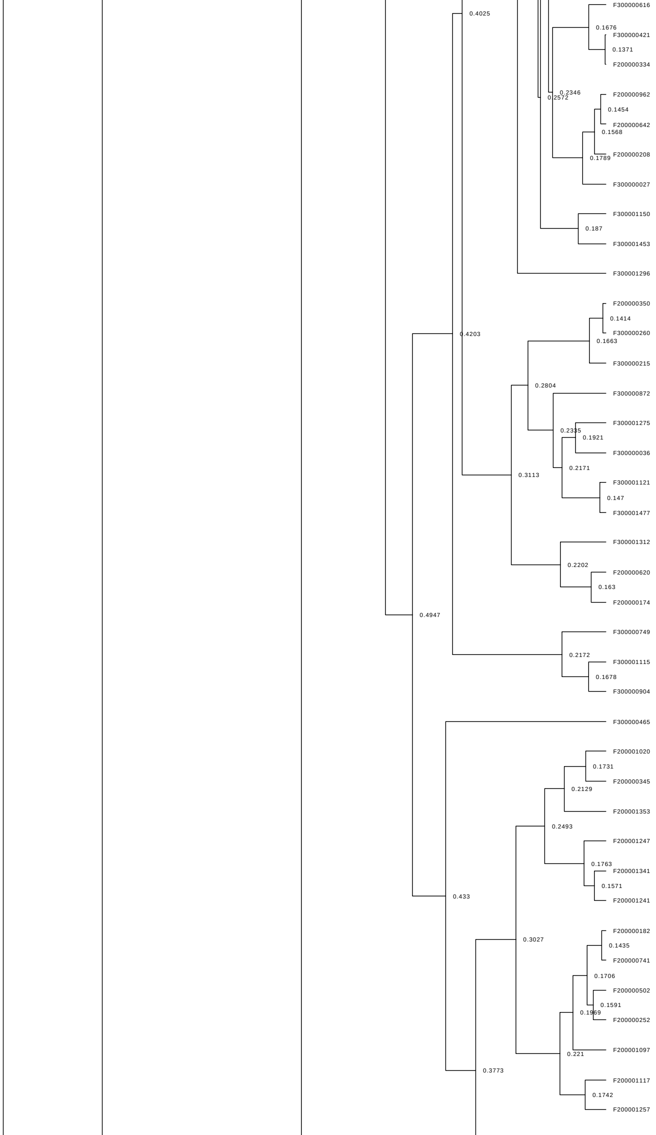
F300000616
0.4025
0.1676
F300000421
0.1371
F200000334
0.2346
0.2572
F200000962
0.1454
F200000642
0.1568
F200000208
0.1789
F300000027
F300001150
0.187
F300001453
F300001296
F200000350
0.1414
F300000260
0.4203
0.1663
F300000215
0.2804
F300000872
F300001275
0.2335
0.1921
F300000036
0.2171
0.3113
F300001121
0.147
F300001477
F300001312
0.2202
F200000620
0.163
F200000174
0.4947
F300000749
0.2172
F300001115
0.1678
F300000904
F300000465
F200001020
0.1731
F200000345
0.2129
F200001353
0.2493
F200001247
0.1763
F200001341
0.1571
F200001241
0.433
F200000182
0.3027
0.1435
F200000741
0.1706
F200000502
0.1591
0.1969
F200000252
F200001097
0.221
0.3773
F200001117
0.1742
F200001257

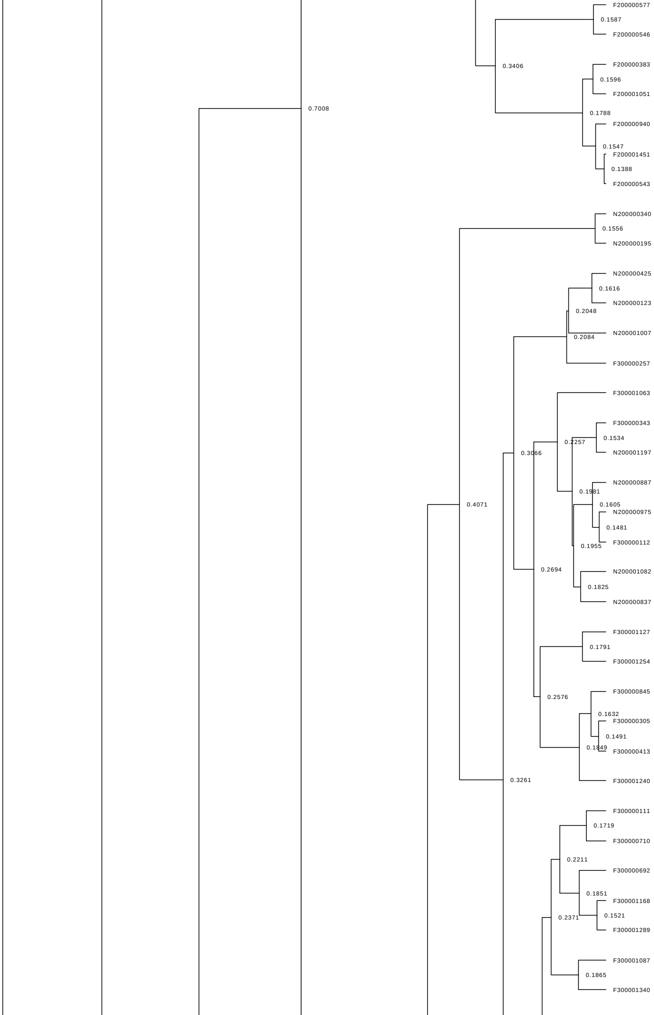
F200000577
0.1587
F200000546
0.3406
F200000383
0.1596
F200001051
0.7008
0.1788
F200000940
0.1547
F200001451
0.1388
F200000543
N200000340
0.1556
N200000195
N200000425
0.1616
N200000123
0.2048
N200001007
0.2084
F300000257
F300001063
F300000343
0.2257
0.1534
0.3066
N200001197
N200000887
0.1981
0.4071
0.1605
N200000975
0.1481
F300000112
0.1955
N200001082
0.2694
0.1825
N200000837
F300001127
0.1791
F300001254
F300000845
0.2576
0.1632
F300000305
0.1491
0.1849
F300000413
0.3261
F300001240
F300000111
0.1719
F300000710
0.2211
F300000692
0.1851
F300001168
0.1521
0.2371
F300001289
F300001087
0.1865
F300001340

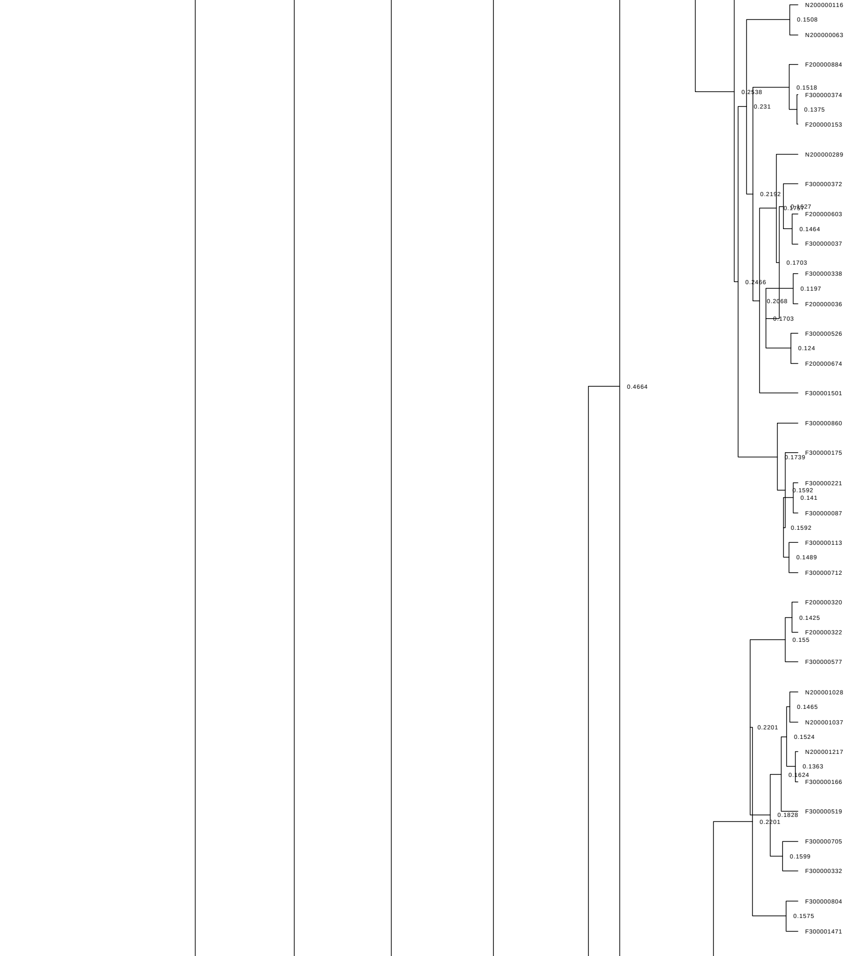

N200000116
0.1508
N200000063
F200000884
0.1518
0.2538
F300000374
0.231
0.1375
F200000153
N200000289
F300000372
0.2192
F200000603
0.1464
F300000037
0.1703
F300000338
0.2466
0.1197
0.2068
F200000036
0.1703
F300000526
0.124
F200000674
0.4664
F300001501
F300000860
F300000175
0.1739
F300000221
0.1592
0.141
F300000087
0.1592
F300000113
0.1489
F300000712
F200000320
0.1425
F200000322
0.155
F300000577
N200001028
0.1465
N200001037
0.2201
0.1524
N200001217
0.1363
0.1624
F300000166
F300000519
0.1828
0.2201
F300000705
0.1599
F300000332
F300000804
0.1575
F300001471

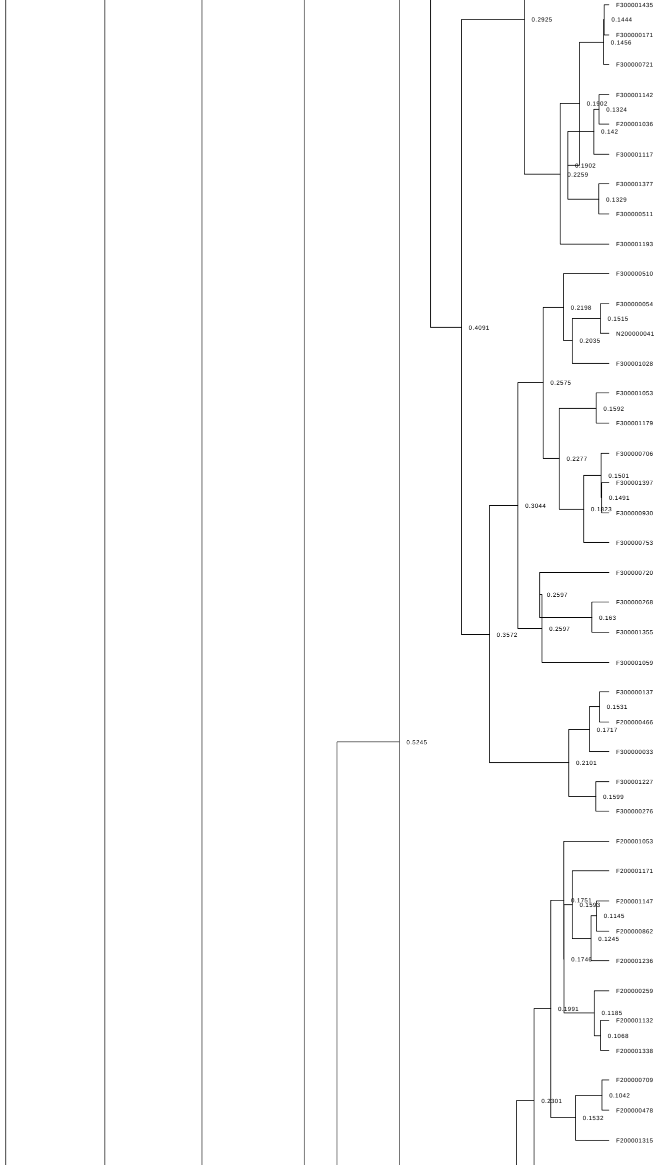
F300001435
0.2925
0.1444
F300000171
0.1456
F300000721
F300001142
0.1902
0.1324
F200001036
0.142
F300001117
0.1902
0.2259
F300001377
0.1329
F300000511
F300001193
F300000510
F300000054
0.2198
0.1515
0.4091
N200000041
0.2035
F300001028
0.2575
F300001053
0.1592
F300001179
0.2277
F300000706
0.1501
F300001397
0.1491
0.1823
F300000930
0.3044
F300000753
F300000720
0.2597
F300000268
0.163
0.2597
F300001355
0.3572
F300001059
F300000137
0.1531
F200000466
0.1717
0.5245
F300000033
0.2101
F300001227
0.1599
F300000276
F200001053
F200001171
0.1751
0.1593
F200001147
0.1145
F200000862
0.1245
0.1746
F200001236
F200000259
0.1991
0.1185
F200001132
0.1068
F200001338
F200000709
0.1042
0.2301
F200000478
0.1532
F200001315

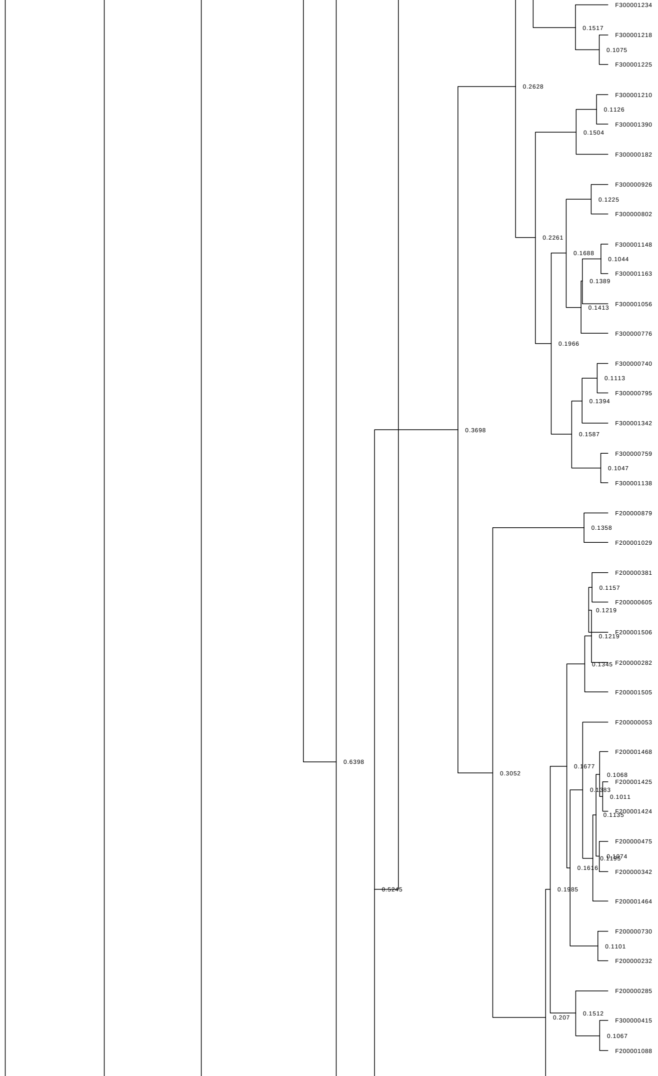

F300001234
0.1517
F300001218
0.1075
F300001225
0.2628
F300001210
0.1126
F300001390
0.1504
F300000182
F300000926
0.1225
F300000802
0.2261
F300001148
0.1688
0.1044
F300001163
0.1389
F300001056
0.1413
F300000776
0.1966
F300000740
0.1113
F300000795
0.1394
F300001342
0.3698
0.1587
F300000759
0.1047
F300001138
F200000879
0.1358
F200001029
F200000381
0.1157
F200000605
0.1219
F200001506
0.1219
F200000282
0.1345
F200001505
F200000053
F200001468
0.6398
0.3052
0.1677
0.1068
F200001425
0.1383
0.1011
F200001424
0.1135
F200000475
0.1074
0.1195
0.1616
F200000342
0.5245
0.1985
F200001464
F200000730
0.1101
F200000232
F200000285
0.1512
0.207
F300000415
0.1067
F200001088

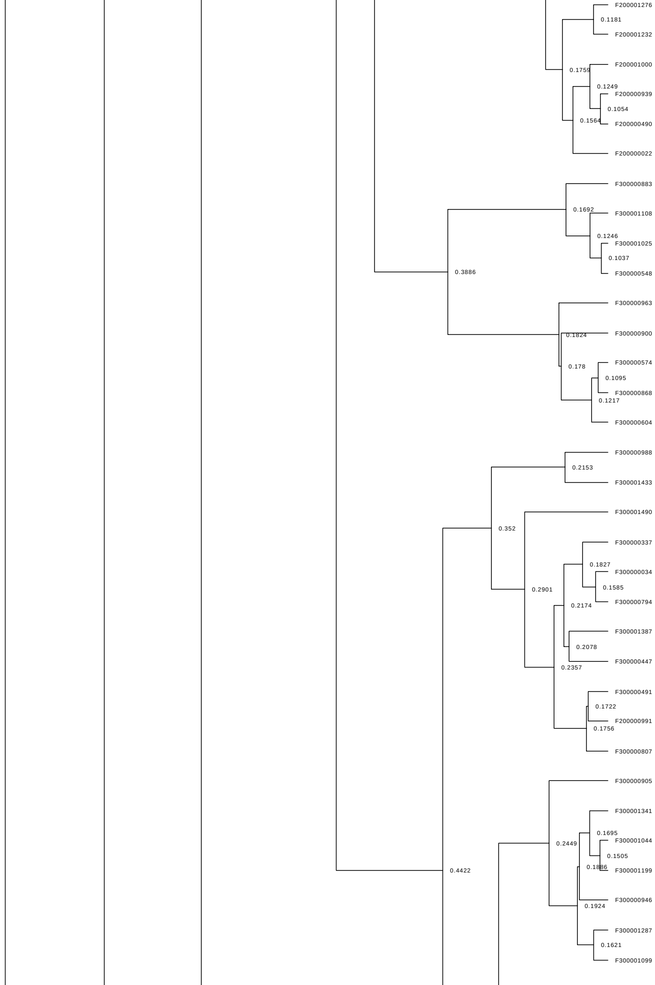
F200001276
0.1181
F200001232
F200001000
0.1759
0.1249
F200000939
0.1054
0.1564
F200000490
F200000022
F300000883
0.1692
F300001108
0.1246
F300001025
0.1037
0.3886
F300000548
F300000963
F300000900
0.1824
F300000574
0.178
0.1095
F300000868
0.1217
F300000604
F300000988
0.2153
F300001433
F300001490
0.352
F300000337
0.1827
F300000034
0.1585
0.2901
F300000794
0.2174
F300001387
0.2078
F300000447
0.2357
F300000491
0.1722
F200000991
0.1756
F300000807
F300000905
F300001341
0.1695
0.2449
F300001044
0.1505
0.1886
0.4422
F300001199
F300000946
0.1924
F300001287
0.1621
F300001099

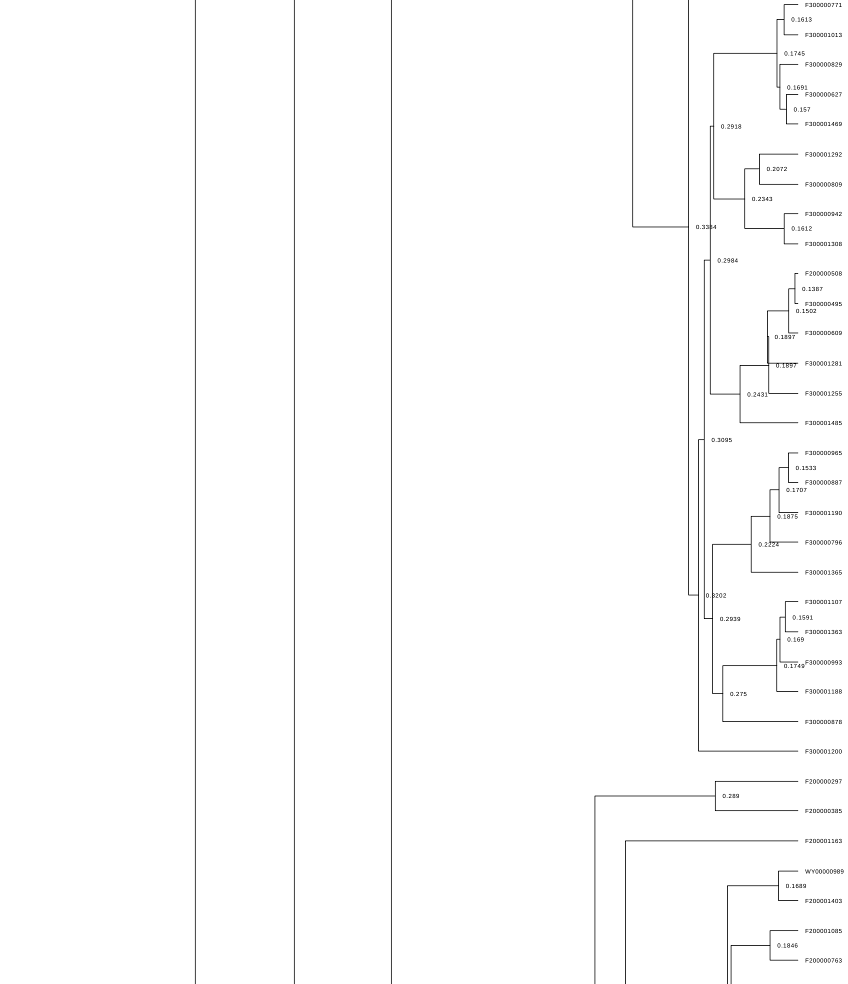

F300000771
0.1613
F300001013
0.1745
F300000829
0.1691
F300000627
0.157
F300001469
0.2918
F300001292
0.2072
F300000809
0.2343
F300000942
0.3384
0.1612
F300001308
0.2984
F200000508
0.1387
F300000495
0.1502
F300000609
0.1897
F300001281
0.1897
F300001255
0.2431
F300001485
0.3095
F300000965
0.1533
F300000887
0.1707
F300001190
0.1875
F300000796
0.2224
F300001365
0.3202
F300001107
0.2939
0.1591
F300001363
0.169
F300000993
0.1749
F300001188
0.275
F300000878
F300001200
F200000297
0.289
F200000385
F200001163
WY00000989
0.1689
F200001403
F200001085
0.1846
F200000763

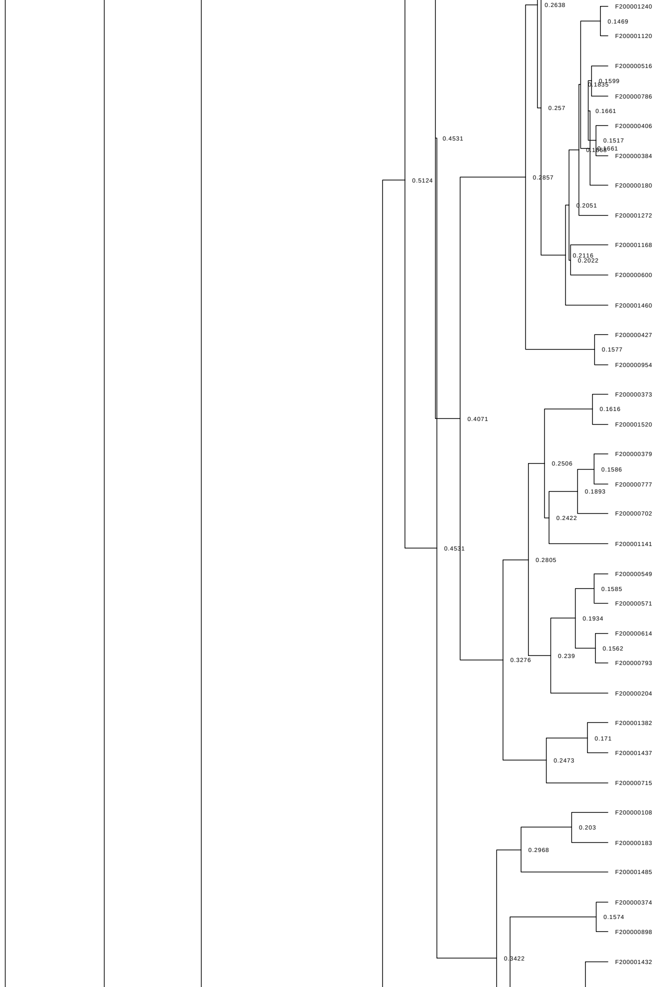

F200001240
0.2638
0.1469
F200001120
F200000516
0.1599
0.1835
F200000786
0.257
0.1661
F200000406
0.4531
0.1517
0.1661
F200000384
0.5124
0.2857
F200000180
0.2051
F200001272
F200001168
0.2116
0.2022
F200000600
F200001460
F200000427
0.1577
F200000954
F200000373
0.1616
0.4071
F200001520
F200000379
0.2506
0.1586
F200000777
0.1893
F200000702
0.2422
F200001141
0.4531
0.2805
F200000549
0.1585
F200000571
0.1934
F200000614
0.1562
0.239
0.3276
F200000793
F200000204
F200001382
0.171
F200001437
0.2473
F200000715
F200000108
0.203
F200000183
0.2968
F200001485
F200000374
0.1574
F200000898
0.3422
F200001432

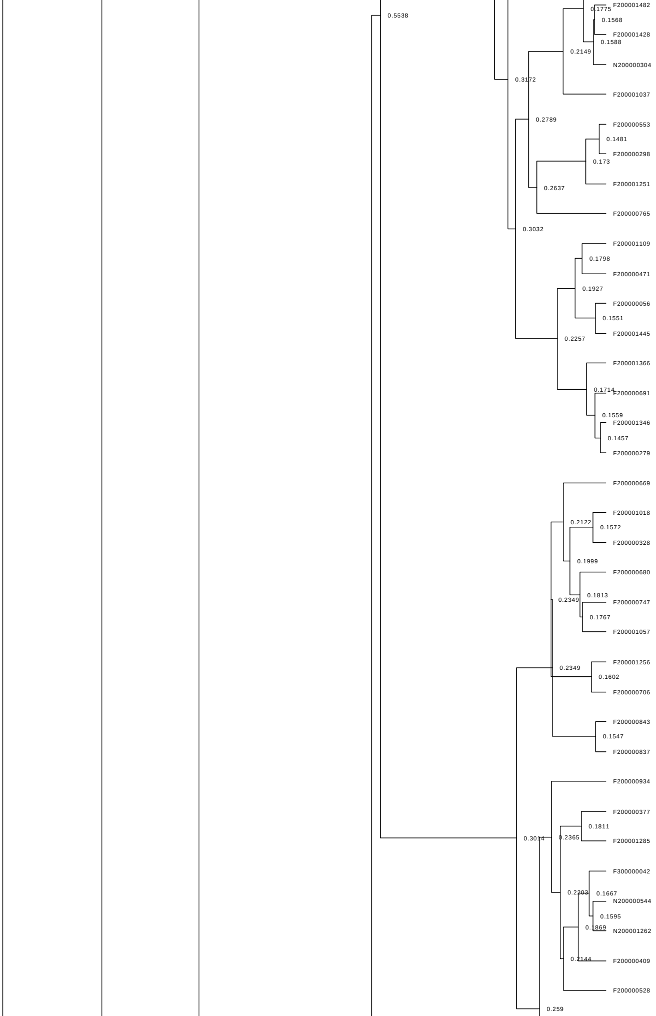
0.5538
F200001482
0.1775
0.1568
F200001428
0.1588
0.2149
N200000304
0.3172
F200001037
0.2789
F200000553
0.1481
F200000298
0.173
F200001251
0.2637
F200000765
0.3032
F200001109
0.1798
F200000471
0.1927
F200000056
0.1551
F200001445
0.2257
F200001366
0.1714
F200000691
0.1559
F200001346
0.1457
F200000279
F200000669
F200001018
0.2122
0.1572
F200000328
0.1999
F200000680
0.1813
0.2349
F200000747
0.1767
F200001057
F200001256
0.2349
0.1602
F200000706
F200000843
0.1547
F200000837
F200000934
F200000377
0.1811
0.3014
0.2365
F200001285
F300000042
0.2203
0.1667
N200000544
0.1595
0.1869
N200001262
0.2144
F200000409
F200000528
0.259

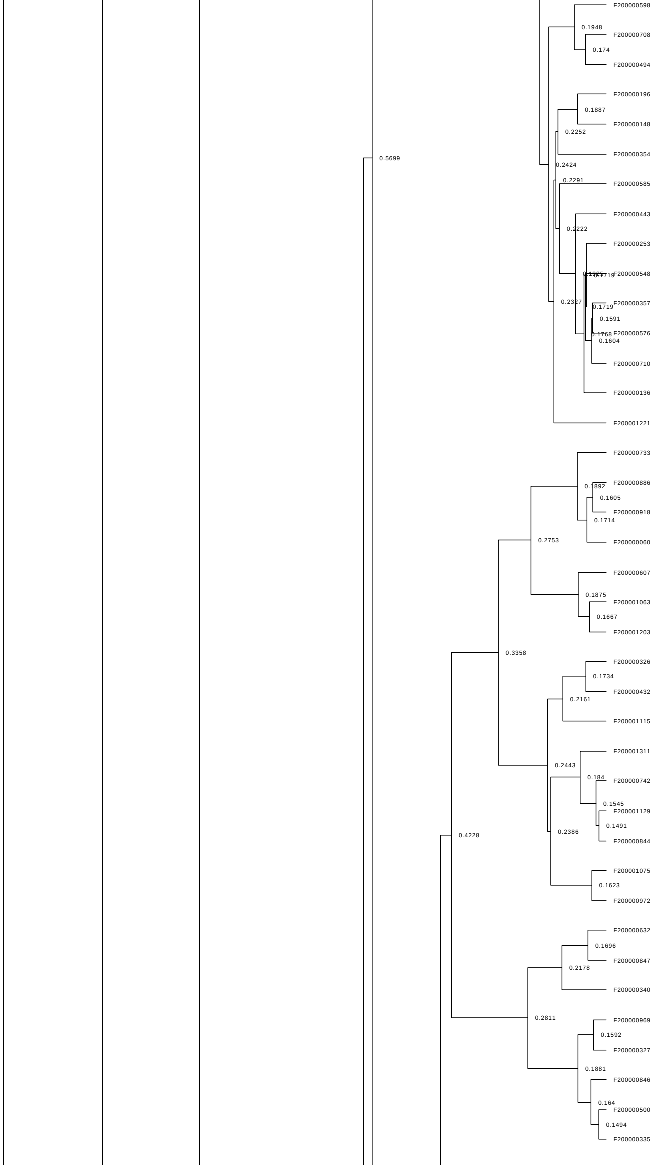

F200000598
0.1948
F200000708
0.174
F200000494
F200000196
0.1887
F200000148
0.2252
F200000354
0.5699
0.2424
0.2291
F200000585
F200000443
0.2222
F200000253
F200000548
F200000357
0.2327
0.1719
0.1591
F200000576
0.1768
0.1604
F200000710
F200000136
F200001221
F200000733
F200000886
0.1892
0.1605
F200000918
0.1714
0.2753
F200000060
F200000607
0.1875
F200001063
0.1667
F200001203
0.3358
F200000326
0.1734
F200000432
0.2161
F200001115
F200001311
0.2443
0.184
F200000742
0.1545
F200001129
0.1491
0.2386
F200000844
0.4228
F200001075
0.1623
F200000972
F200000632
0.1696
F200000847
0.2178
F200000340
0.2811
F200000969
0.1592
F200000327
0.1881
F200000846
0.164
F200000500
0.1494
F200000335

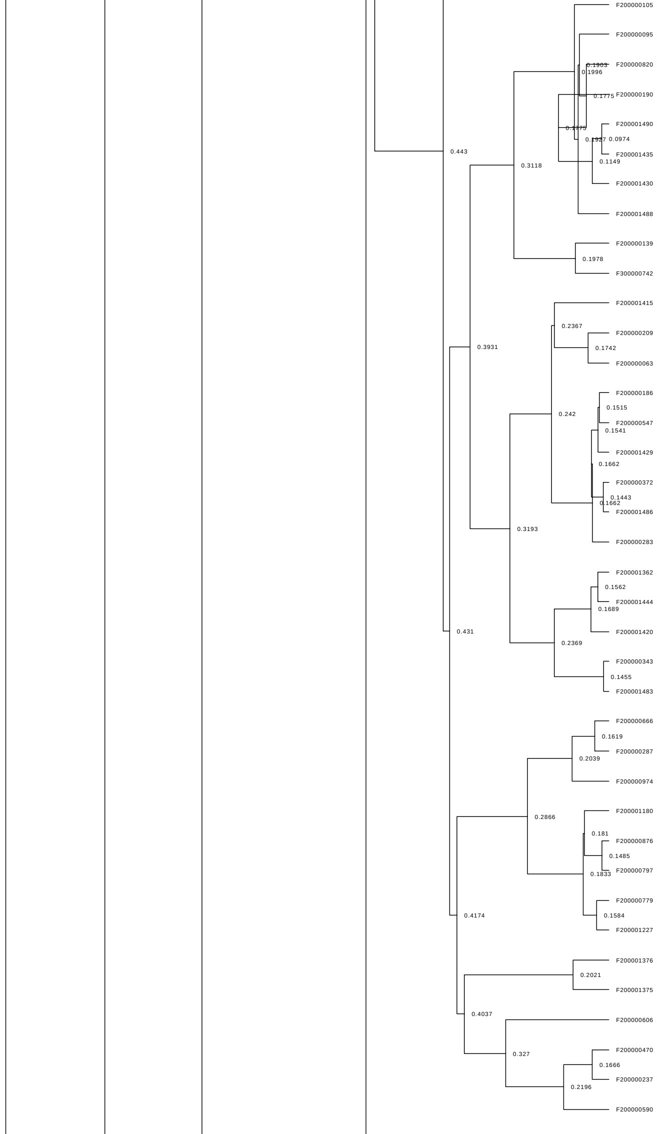

F200000105
F200000095
F200000820
F200000190
F200001490
F200001435
F200001430
F200001488
F200000139
F300000742
F200001415
F200000209
F200000063
F200000186
F200000547
F200001429
F200000372
F200001486
F200000283
F200001362
F200001444
F200001420
F200000343
F200001483
F200000666
F200000287
F200000974
F200001180
F200000876
F200000797
F200000779
F200001227
F200001376
F200001375
F200000606
F200000470
F200000237
F200000590
0.443
0.3118
0.3931
0.3193
0.431
0.4174
0.4037
0.327
0.2866
0.2367
0.1742
0.242
0.1515
0.1541
0.1662
0.1443
0.1662
0.1562
0.1689
0.2369
0.1455
0.1619
0.2039
0.181
0.1485
0.1833
0.1584
0.2021
0.1666
0.2196
0.1978
0.1996
0.1775
0.0974
0.1149

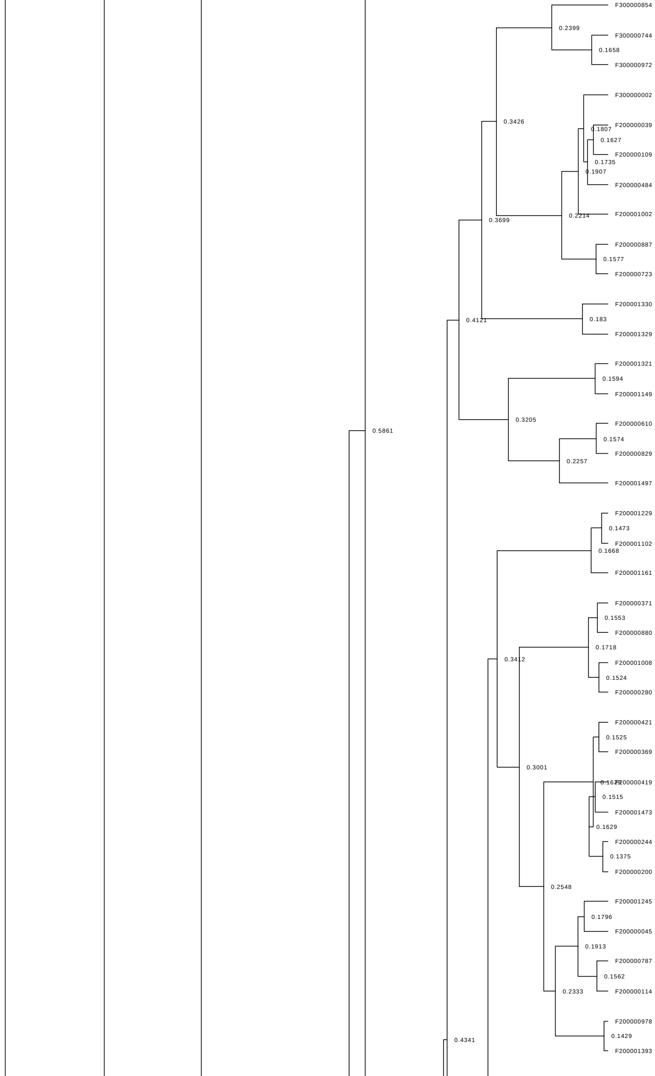

F300000854
0.2399
F300000744
0.1658
F300000972
F300000002
F200000039
0.1807
0.1627
F200000109
0.1735
0.1907
F200000484
F200001002
0.3426
0.2214
0.3699
F200000887
0.1577
F200000723
F200001330
0.183
0.4121
F200001329
F200001321
0.1594
F200001149
0.3205
0.5861
F200000610
0.1574
F200000829
0.2257
F200001497
F200001229
0.1473
F200001102
0.1668
F200001161
F200000371
0.1553
F200000880
0.1718
0.3412
F200001008
0.1524
F200000280
F200000421
0.1525
F200000369
0.3001
F200000419
0.1515
F200001473
0.1629
F200000244
0.1375
F200000200
0.2548
F200001245
0.1796
F200000045
0.1913
F200000787
0.1562
F200000114
0.2333
F200000978
0.1429
0.4341
F200001393

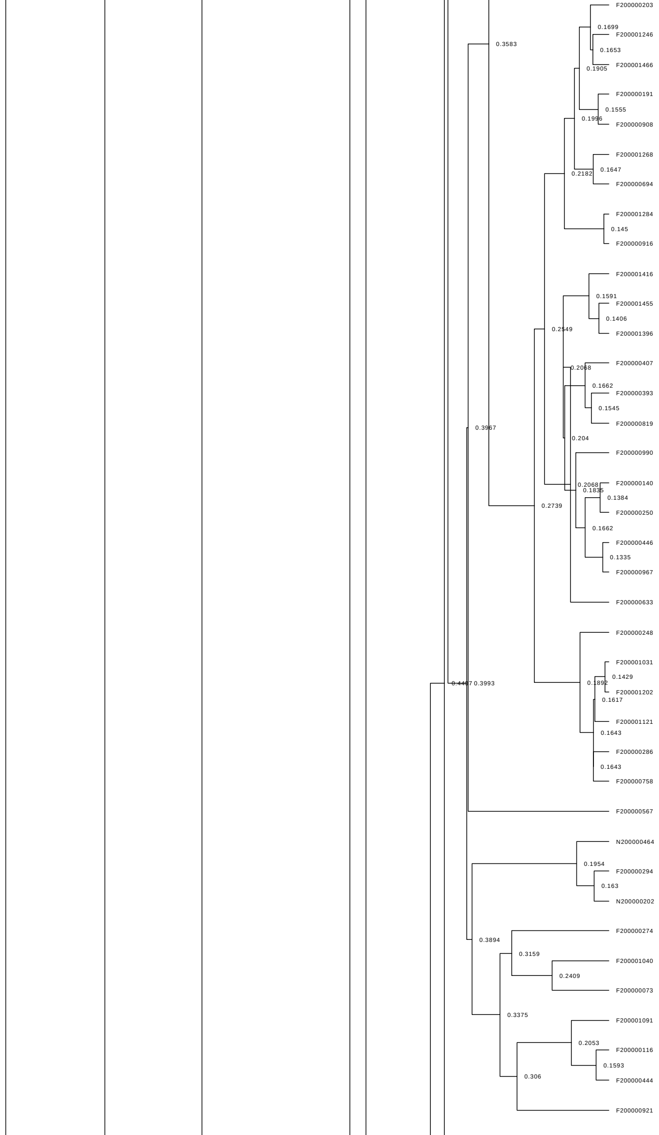
F200000203
0.1699
F200001246
0.1653
F200001466
0.1905
0.3583
F200000191
0.1555
0.1996
F200000908
F200001268
0.1647
0.2182
F200000694
F200001284
0.145
F200000916
F200001416
0.1591
F200001455
0.1406
F200001396
0.2549
F200000407
0.2068
0.1662
F200000393
0.1545
F200000819
0.3967
0.204
F200000990
F200000140
0.2068
0.1835
0.1384
F200000250
0.2739
0.1662
F200000446
0.1335
F200000967
F200000633
F200000248
F200001031
0.1429
0.4467
0.3993
0.1892
F200001202
0.1617
F200001121
0.1643
F200000286
0.1643
F200000758
F200000567
N200000464
0.1954
F200000294
0.163
N200000202
F200000274
0.3894
0.3159
F200001040
0.2409
F200000073
0.3375
F200001091
0.2053
F200000116
0.1593
0.306
F200000444
F200000921

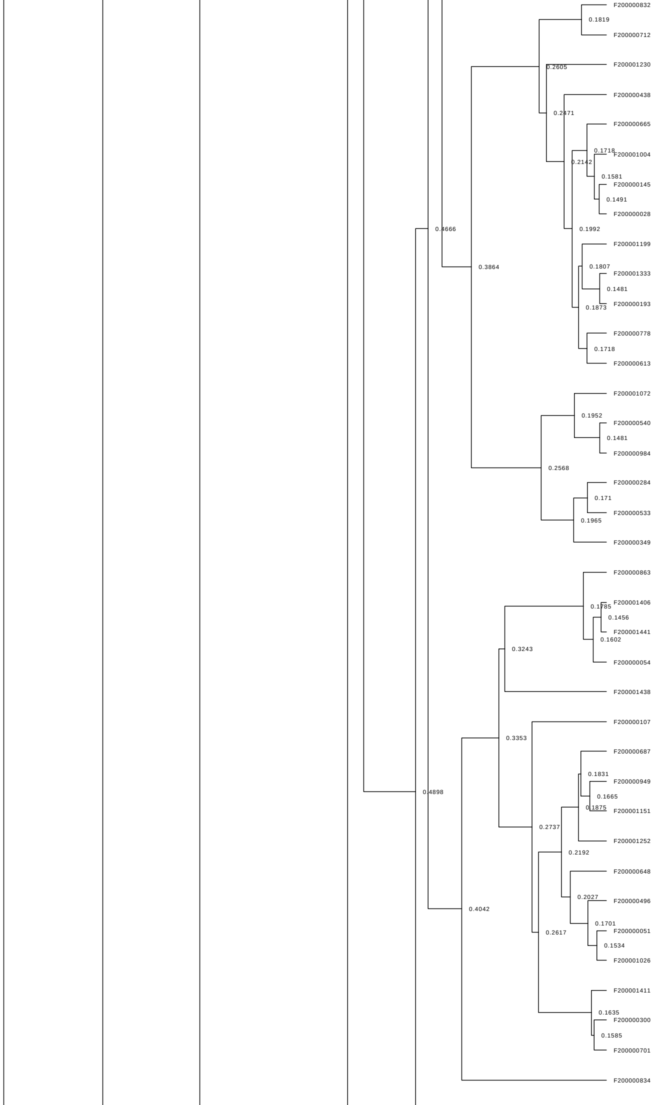
F200000832
0.1819
F200000712
F200001230
0.2605
F200000438
0.2471
F200000665
0.1718
F200001004
0.2142
0.1581
F200000145
0.1491
F200000028
0.4666
0.1992
F200001199
0.3864
0.1807
F200001333
0.1481
F200000193
0.1873
F200000778
0.1718
F200000613
F200001072
0.1952
F200000540
0.1481
F200000984
0.2568
F200000284
0.171
F200000533
0.1965
F200000349
F200000863
F200001406
0.1785
0.1456
F200001441
0.1602
0.3243
F200000054
F200001438
F200000107
0.3353
F200000687
0.1831
F200000949
0.1665
0.4898
0.1875
F200001151
0.2737
F200001252
0.2192
F200000648
0.2027
F200000496
0.4042
0.1701
F200000051
0.2617
0.1534
F200001026
F200001411
0.1635
F200000300
0.1585
F200000701
F200000834

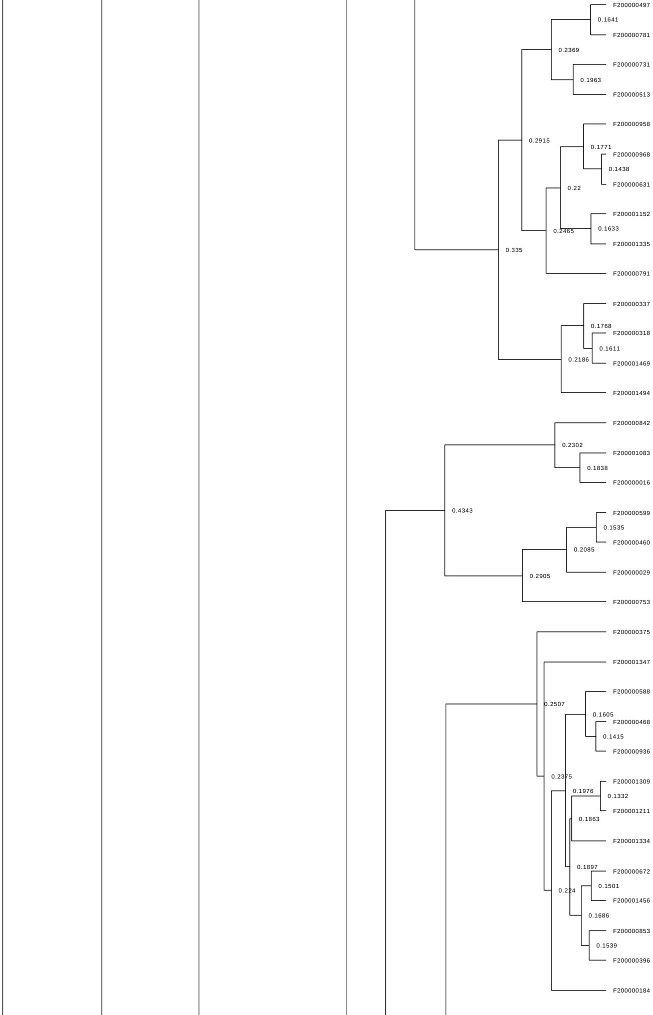
F200000497
0.1641
F200000781
0.2369
F200000731
0.1963
F200000513
F200000958
0.2915
0.1771
F200000968
0.1438
F200000631
0.22
F200001152
0.2465
0.1633
F200001335
0.335
F200000791
F200000337
0.1768
F200000318
0.1611
0.2186
F200001469
F200001494
F200000842
0.2302
F200001083
0.1838
F200000016
0.4343
F200000599
0.1535
F200000460
0.2085
F200000029
0.2905
F200000753
F200000375
F200001347
F200000588
0.2507
0.1605
F200000468
0.1415
F200000936
0.2375
F200001309
0.1976
0.1332
F200001211
0.1863
F200001334
0.1897
F200000672
0.1501
0.224
F200001456
0.1686
F200000853
0.1539
F200000396
F200000184

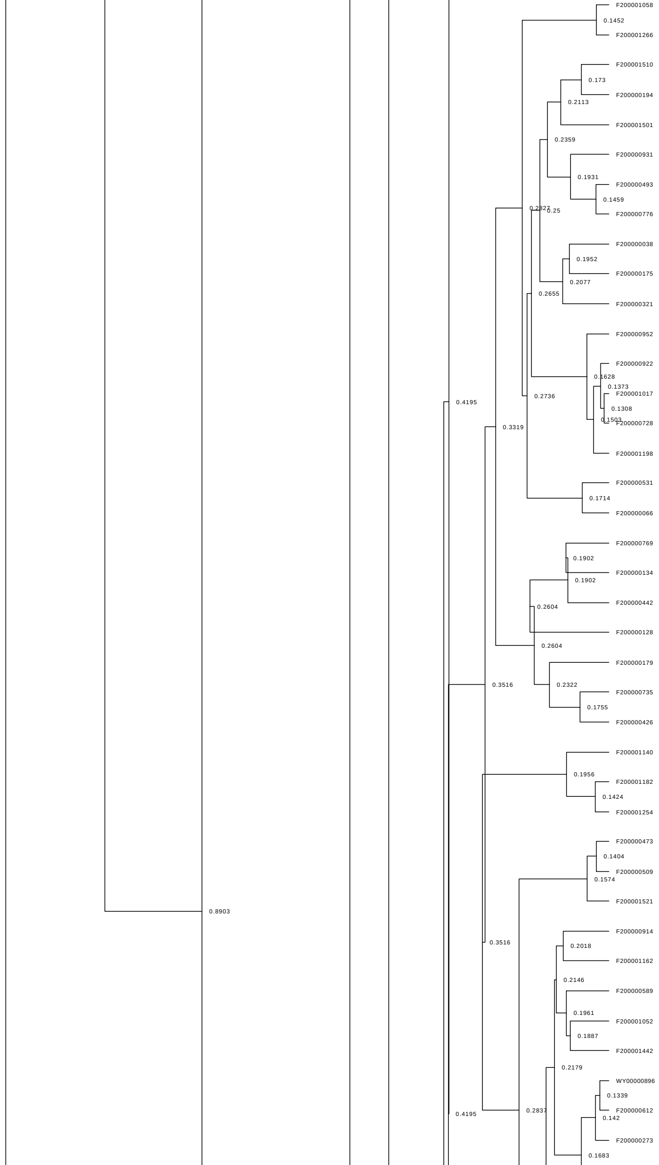
F200001058
0.1452
F200001266
F200001510
0.173
F200000194
0.2113
F200001501
0.2359
F200000931
0.1931
F200000493
0.1459
F200000776
0.2327
0.25
F200000038
0.1952
F200000175
0.2077
0.2655
F200000321
F200000952
F200000922
0.1628
0.1373
F200001017
0.1308
0.1503
F200000728
0.2736
F200001198
0.4195
0.3319
F200000531
0.1714
F200000066
F200000769
0.1902
F200000134
0.1902
F200000442
0.2604
F200000128
0.2604
F200000179
0.3516
0.2322
F200000735
0.1755
F200000426
F200001140
0.1956
F200001182
0.1424
F200001254
F200000473
0.1404
F200000509
0.1574
F200001521
0.8903
F200000914
0.3516
0.2018
F200001162
0.2146
F200000589
0.1961
F200001052
0.1887
F200001442
0.2179
WY00000896
0.1339
F200000612
0.2837
0.4195
0.142
F200000273
0.1683

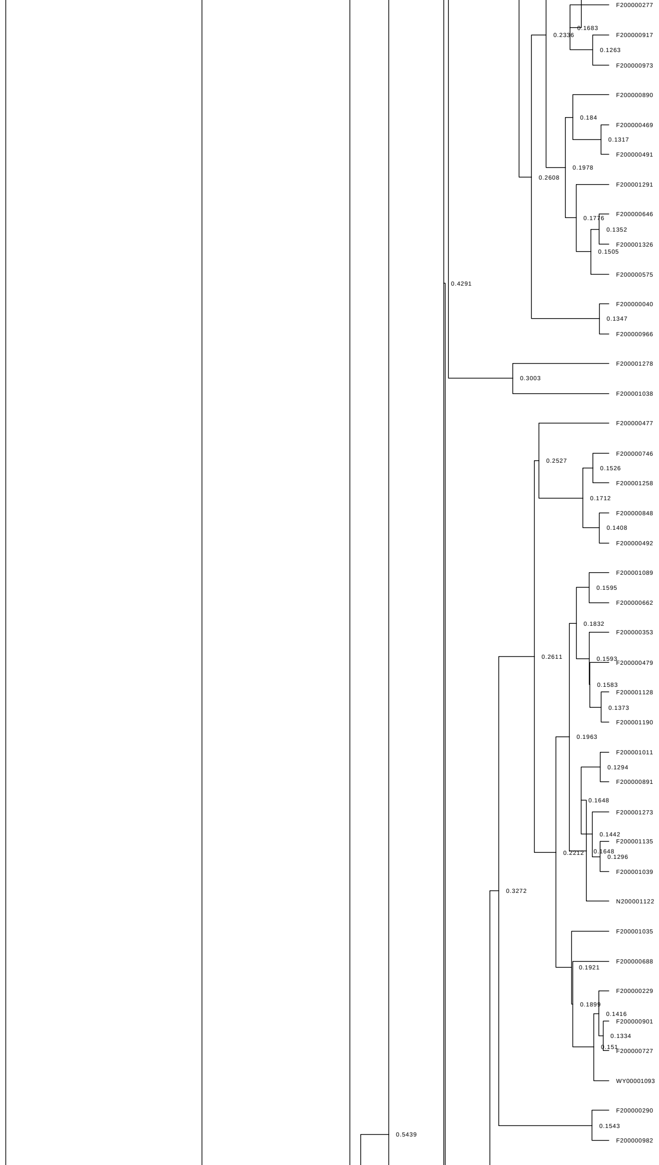

F200000277
0.1683
0.2336
F200000917
0.1263
F200000973
F200000890
0.184
F200000469
0.1317
F200000491
0.1978
0.2608
F200001291
F200000646
0.1776
0.1352
F200001326
0.1505
F200000575
0.4291
F200000040
0.1347
F200000966
F200001278
0.3003
F200001038
F200000477
F200000746
0.2527
0.1526
F200001258
0.1712
F200000848
0.1408
F200000492
F200001089
0.1595
F200000662
0.1832
F200000353
0.2611
0.1593
F200000479
0.1583
F200001128
0.1373
F200001190
0.1963
F200001011
0.1294
F200000891
0.1648
F200001273
0.1442
F200001135
0.2212
0.1648
0.1296
F200001039
0.3272
N200001122
F200001035
F200000688
0.1921
F200000229
0.1899
0.1416
F200000901
0.1334
0.151
F200000727
WY00001093
F200000290
0.1543
F200000982
0.5439

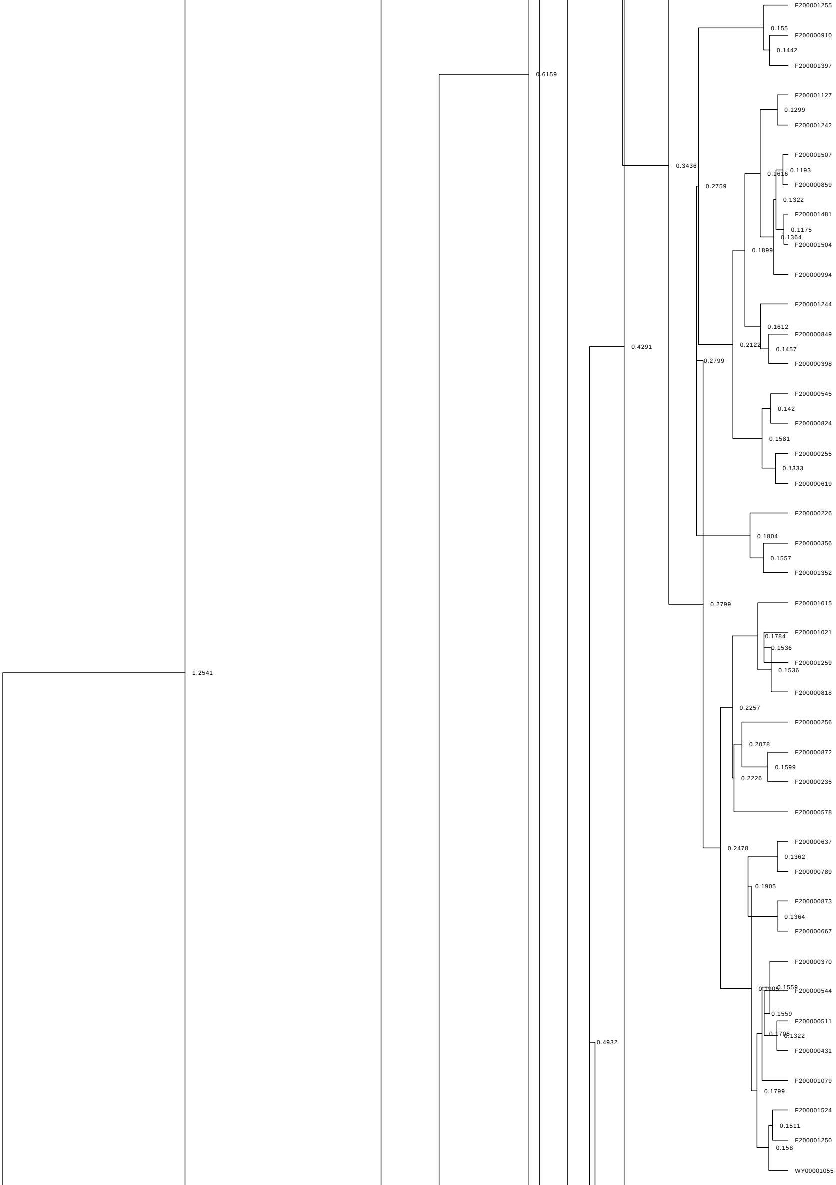
F200001255
0.155
F200000910
0.1442
F200001397
0.6159
F200001127
0.1299
F200001242
F200001507
0.3436
0.1193
0.1616
F200000859
0.2759
0.1322
F200001481
0.1175
0.1364
F200001504
0.1899
F200000994
F200001244
0.1612
F200000849
0.4291
0.2122
0.1457
0.2799
F200000398
F200000545
0.142
F200000824
0.1581
F200000255
0.1333
F200000619
F200000226
0.1804
F200000356
0.1557
F200001352
F200001015
0.2799
F200001021
0.1784
0.1536
F200001259
1.2541
0.1536
F200000818
0.2257
F200000256
0.2078
F200000872
0.1599
0.2226
F200000235
F200000578
F200000637
0.2478
0.1362
F200000789
0.1905
F200000873
0.1364
F200000667
F200000370
0.1905
0.1559
F200000544
0.1559
F200000511
0.1705
0.1322
F200000431
0.4932
F200001079
0.1799
F200001524
0.1511
F200001250
0.158
WY00001055

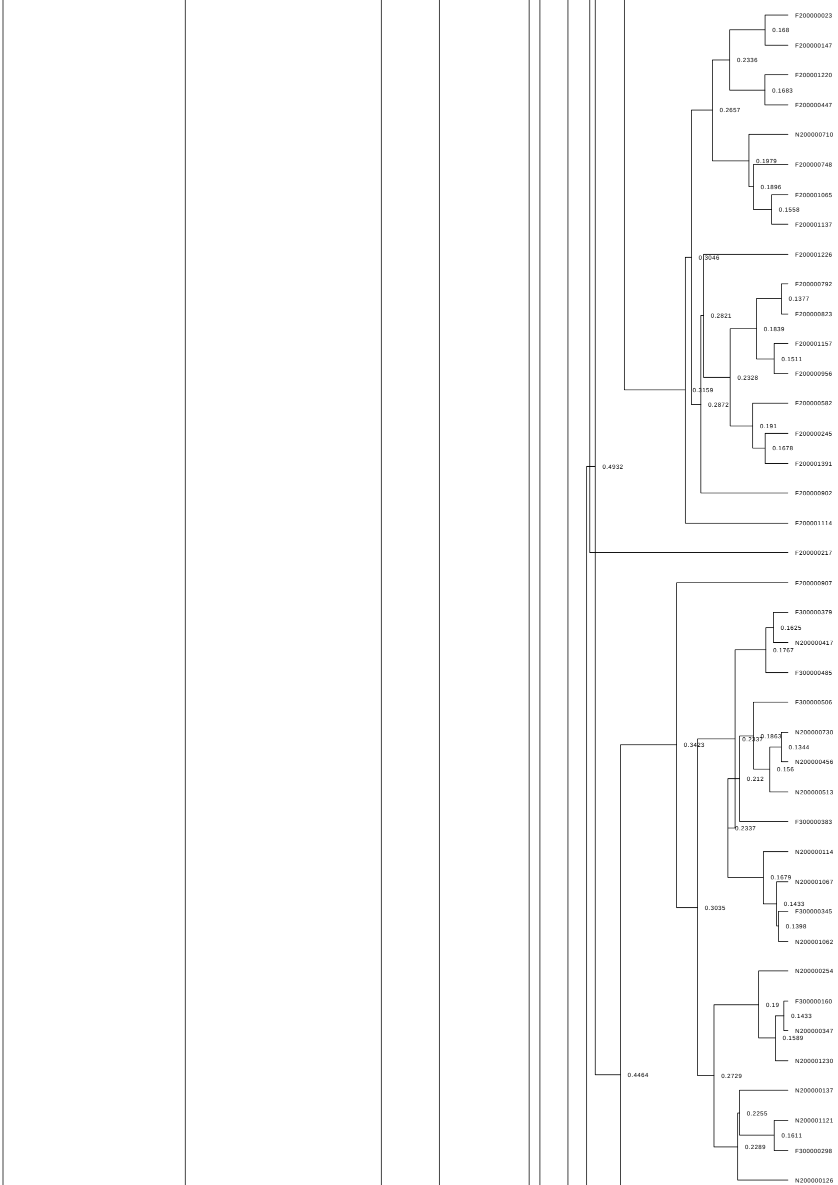
F200000023
0.168
F200000147
0.2336
F200001220
0.1683
F200000447
0.2657
N200000710
0.1979
F200000748
0.1896
F200001065
0.1558
F200001137
F200001226
0.3046
F200000792
0.1377
F200000823
0.2821
0.1839
F200001157
0.1511
F200000956
0.2328
0.3159
F200000582
0.2872
0.191
F200000245
0.1678
F200001391
0.4932
F200000902
F200001114
F200000217
F200000907
F300000379
0.1625
N200000417
0.1767
F300000485
F300000506
N200000730
0.1863
0.2337
0.3423
0.1344
N200000456
0.156
0.212
N200000513
F300000383
0.2337
N200000114
0.1679
N200001067
0.1433
F300000345
0.3035
0.1398
N200001062
N200000254
0.19
F300000160
0.1433
N200000347
0.1589
N200001230
0.4464
0.2729
N200000137
0.2255
N200001121
0.1611
0.2289
F300000298
N200000126

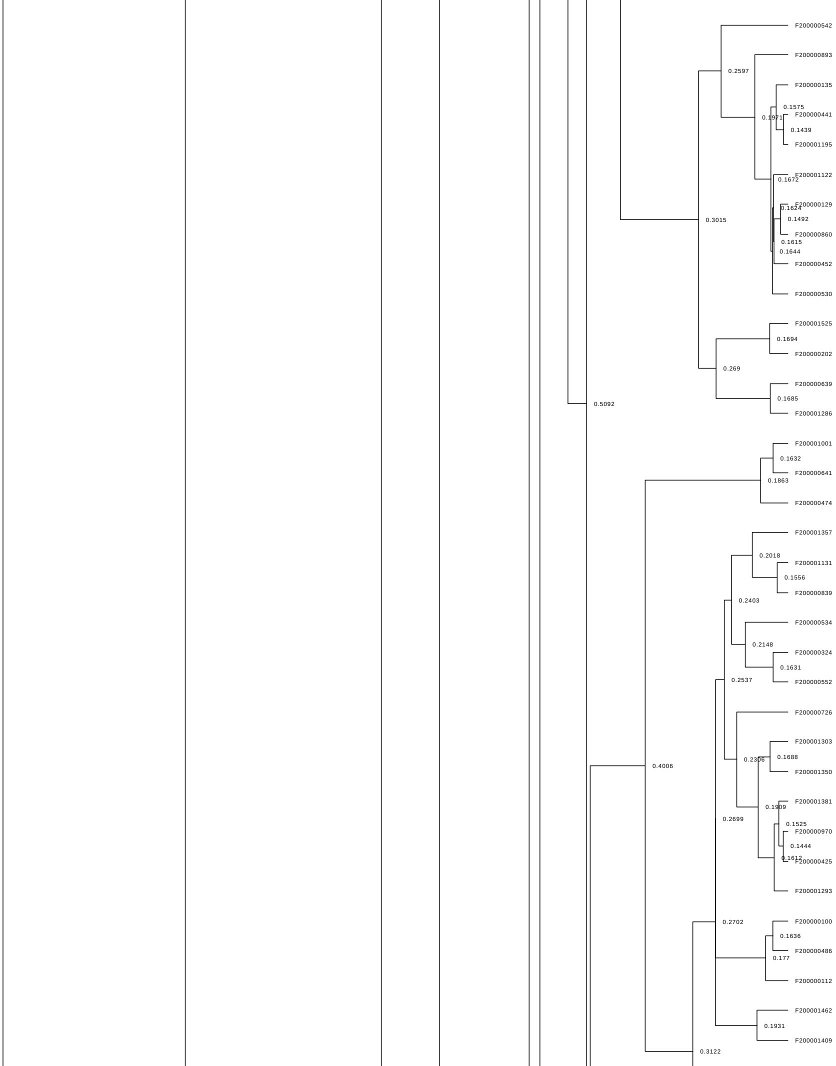
F200000542
F200000893
0.2597
F200000135
0.1575
0.1971
F200000441
0.1439
F200001195
F200001122
0.1672
F200000129
0.1624
0.1492
0.3015
F200000860
0.1615
0.1644
F200000452
F200000530
F200001525
0.1694
F200000202
0.269
F200000639
0.1685
0.5092
F200001286
F200001001
0.1632
F200000641
0.1863
F200000474
F200001357
0.2018
F200001131
0.1556
F200000839
0.2403
F200000534
0.2148
F200000324
0.1631
0.2537
F200000552
F200000726
F200001303
0.1688
0.2306
F200001350
0.4006
F200001381
0.1909
0.2699
0.1525
F200000970
0.1444
0.1612
F200000425
F200001293
F200000100
0.2702
0.1636
F200000486
0.177
F200000112
F200001462
0.1931
F200001409
0.3122

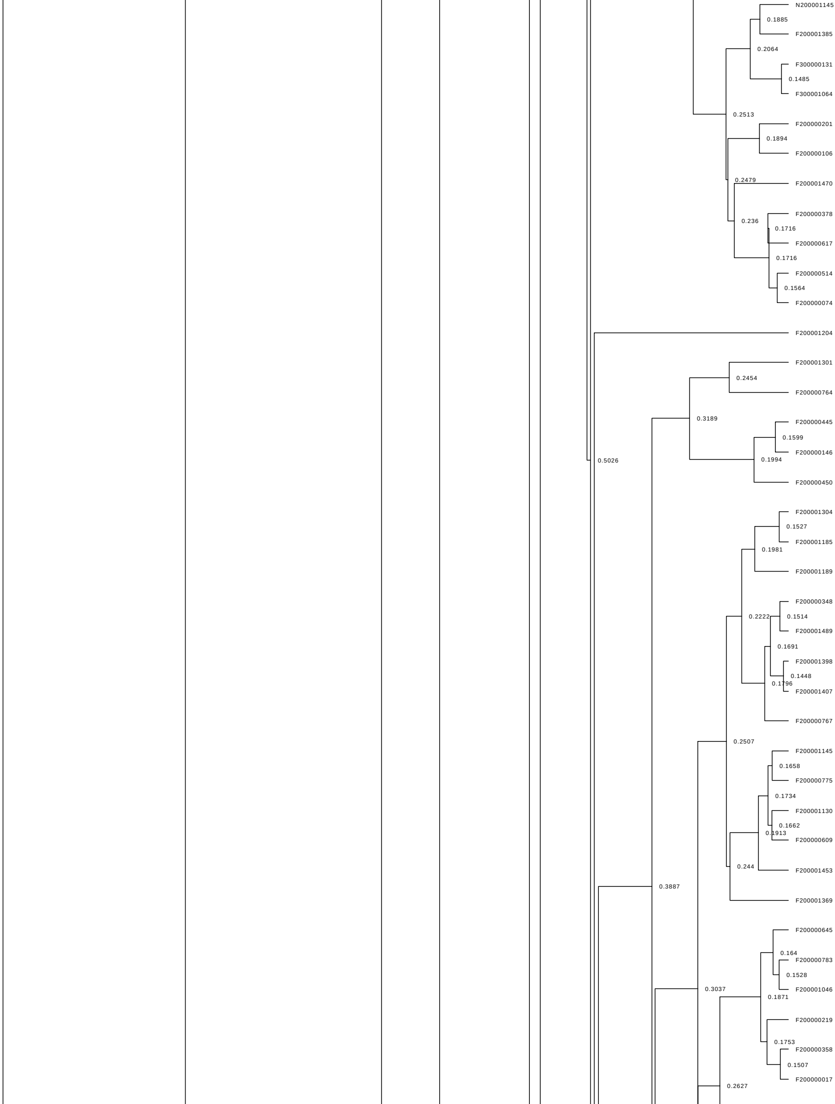
N200001145
0.1885
F200001385
0.2064
F300000131
0.1485
F300001064
0.2513
F200000201
0.1894
F200000106
0.2479
F200001470
F200000378
0.236
0.1716
F200000617
0.1716
F200000514
0.1564
F200000074
F200001204
F200001301
0.2454
F200000764
0.3189
F200000445
0.1599
F200000146
0.5026
0.1994
F200000450
F200001304
0.1527
F200001185
0.1981
F200001189
F200000348
0.2222
0.1514
F200001489
0.1691
F200001398
0.1448
0.1796
F200001407
F200000767
0.2507
F200001145
0.1658
F200000775
0.1734
F200001130
0.1662
0.1913
F200000609
0.244
F200001453
0.3887
F200001369
F200000645
0.164
F200000783
0.1528
0.3037
F200001046
0.1871
F200000219
0.1753
F200000358
0.1507
F200000017
0.2627

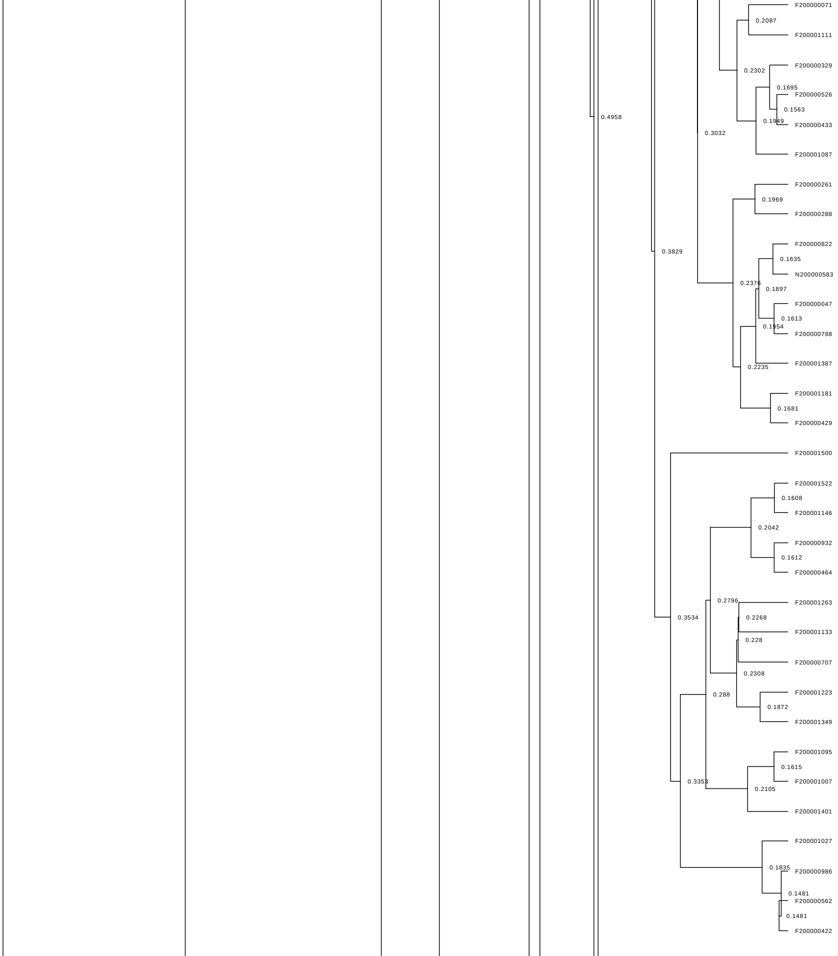
F200000071
0.2087
F200001111
F200000329
0.2302
0.1695
F200000526
0.1563
0.4958
0.1949
F200000433
0.3032
F200001087
F200000261
0.1969
F200000288
F200000822
0.3829
0.1635
N200000583
0.2376
0.1897
F200000047
0.1613
0.1954
F200000788
F200001387
0.2235
F200001181
0.1681
F200000429
F200001500
F200001522
0.1608
F200001146
0.2042
F200000932
0.1612
F200000464
0.2796
F200001263
0.3534
0.2268
F200001133
0.228
F200000707
0.2308
F200001223
0.288
0.1872
F200001349
F200001095
0.1615
0.3353
F200001007
0.2105
F200001401
F200001027
0.1835
F200000986
0.1481
F200000562
0.1481
F200000422

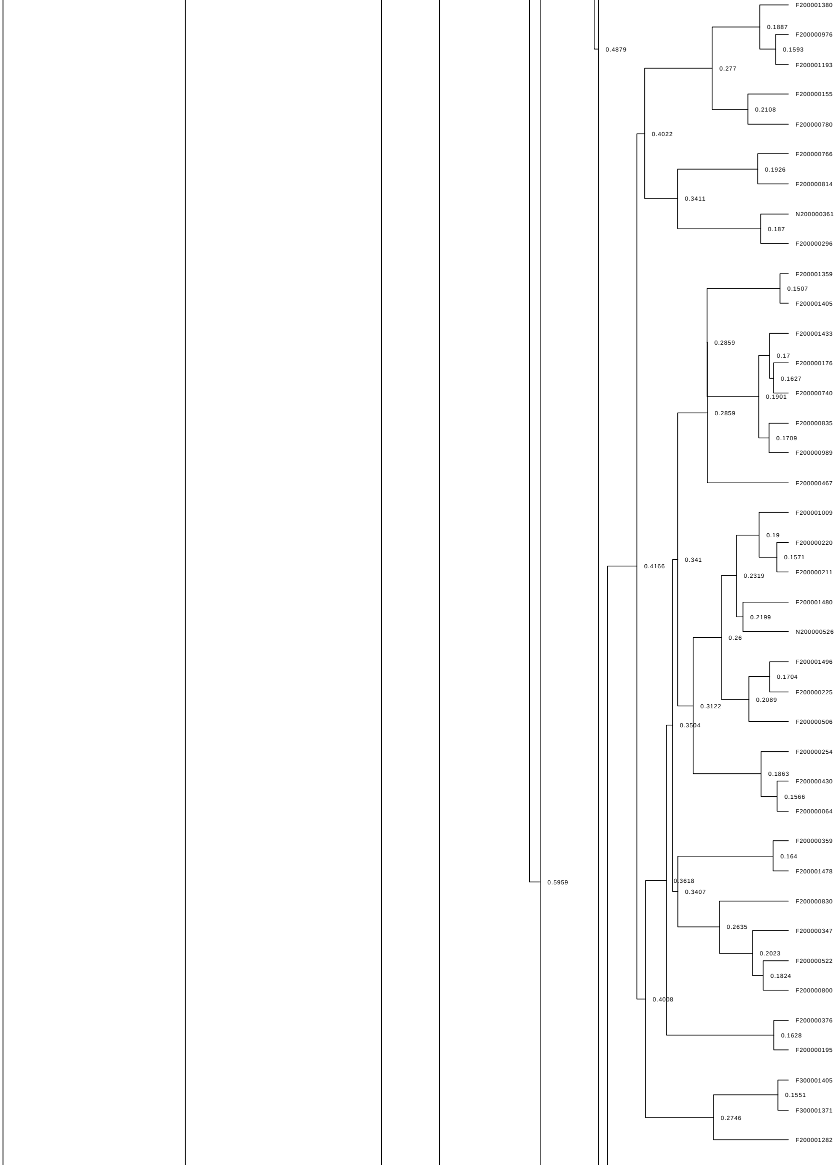

F200001380
0.1887
F200000976
0.4879
0.1593
F200001193
0.277
F200000155
0.2108
F200000780
0.4022
F200000766
0.1926
F200000814
0.3411
N200000361
0.187
F200000296
F200001359
0.1507
F200001405
F200001433
0.2859
0.17
F200000176
0.1627
F200000740
0.1901
0.2859
F200000835
0.1709
F200000989
F200000467
F200001009
0.19
F200000220
0.1571
0.341
0.4166
F200000211
0.2319
F200001480
0.2199
N200000526
0.26
F200001496
0.1704
F200000225
0.2089
0.3122
F200000506
0.3504
F200000254
0.1863
F200000430
0.1566
F200000064
F200000359
0.164
F200001478
0.5959
0.3618
0.3407
F200000830
0.2635
F200000347
0.2023
F200000522
0.1824
F200000800
0.4008
F200000376
0.1628
F200000195
F300001405
0.1551
F300001371
0.2746
F200001282

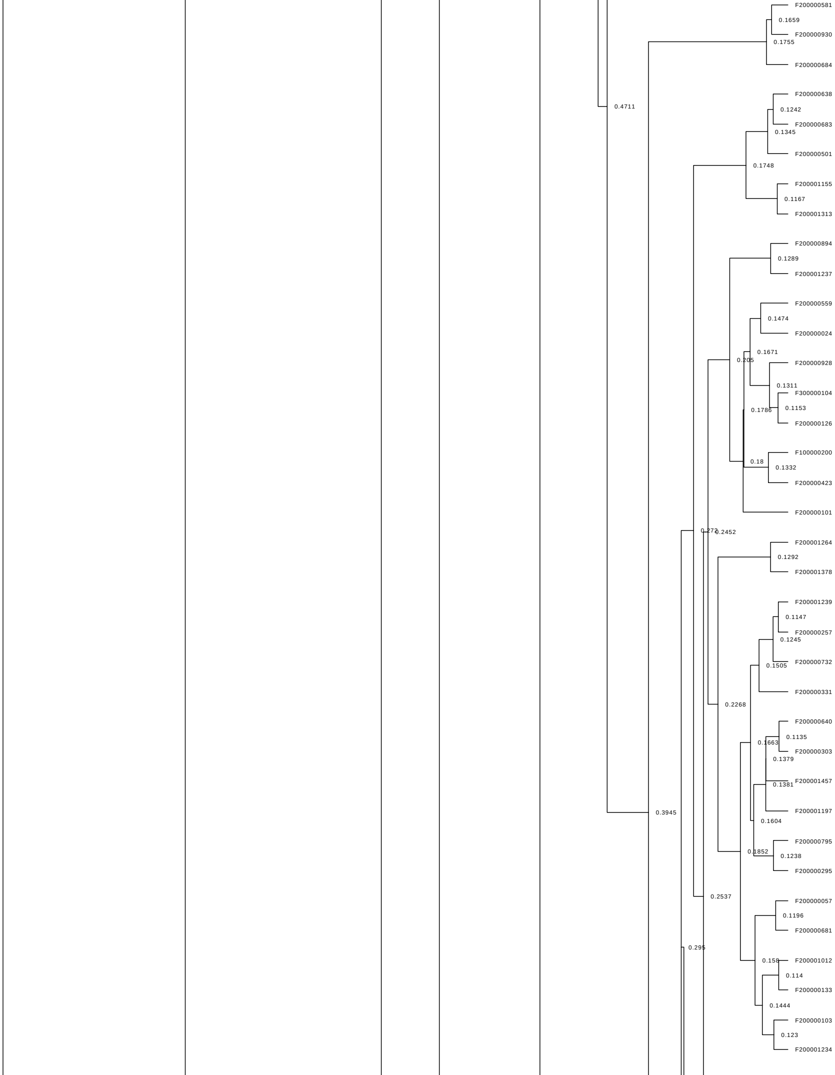

F200000581
0.1659
F200000930
0.1755
F200000684
0.4711
F200000638
0.1242
F200000683
0.1345
F200000501
0.1748
F200001155
0.1167
F200001313
F200000894
0.1289
F200001237
F200000559
0.1474
F200000024
0.1671
0.205
F200000928
0.1311
F300000104
0.1153
0.1786
F200000126
F100000200
0.18
0.1332
F200000423
F200000101
0.272
0.2452
F200001264
0.1292
F200001378
F200001239
0.1147
F200000257
0.1245
F200000732
0.1505
F200000331
0.2268
F200000640
0.1135
0.1663
F200000303
0.1379
F200001457
0.1381
F200001197
0.1604
F200000795
0.1852
0.1238
F200000295
0.3945
0.2537
F200000057
0.1196
F200000681
0.295
0.158
F200001012
0.114
F200000133
0.1444
F200000103
0.123
F200001234

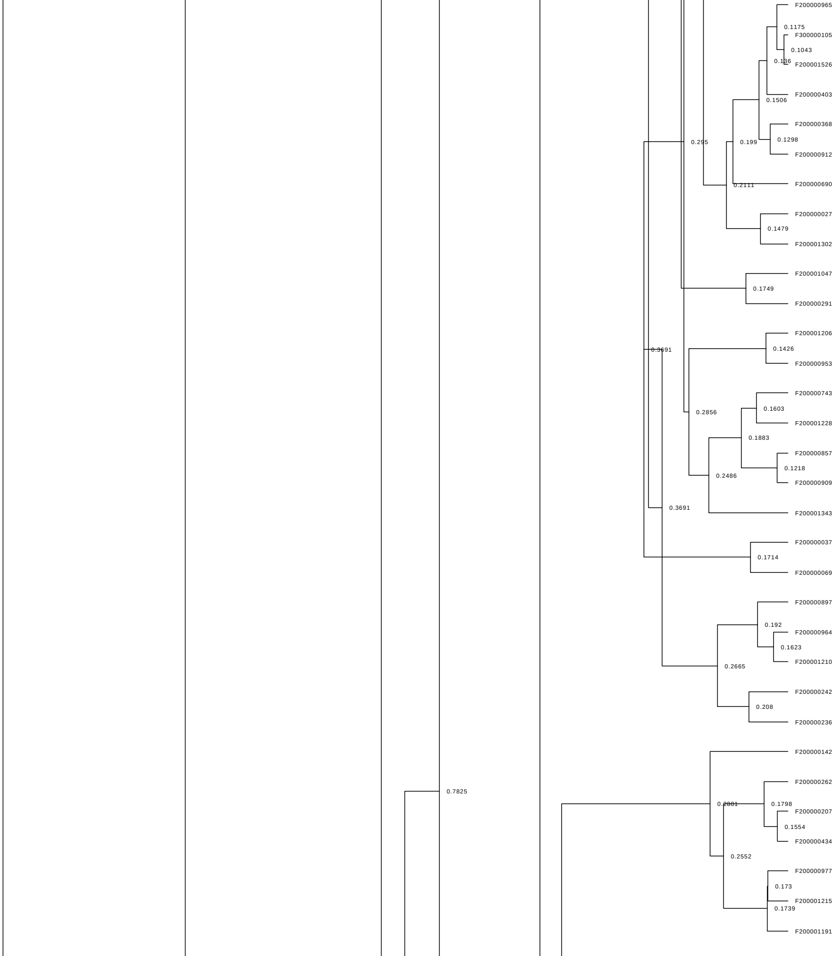

F200000965
0.1175
F300000105
0.1043
0.136
F200001526
F200000403
0.1506
F200000368
0.295
0.199
0.1298
F200000912
0.2111
F200000690
F200000027
0.1479
F200001302
F200001047
0.1749
F200000291
F200001206
0.3691
0.1426
F200000953
F200000743
0.2856
0.1603
F200001228
0.1883
F200000857
0.1218
0.2486
F200000909
0.3691
F200001343
F200000037
0.1714
F200000069
F200000897
0.192
F200000964
0.1623
0.2665
F200001210
F200000242
0.208
F200000236
F200000142
F200000262
0.7825
0.2801
0.1798
F200000207
0.1554
F200000434
0.2552
F200000977
0.173
F200001215
0.1739
F200001191

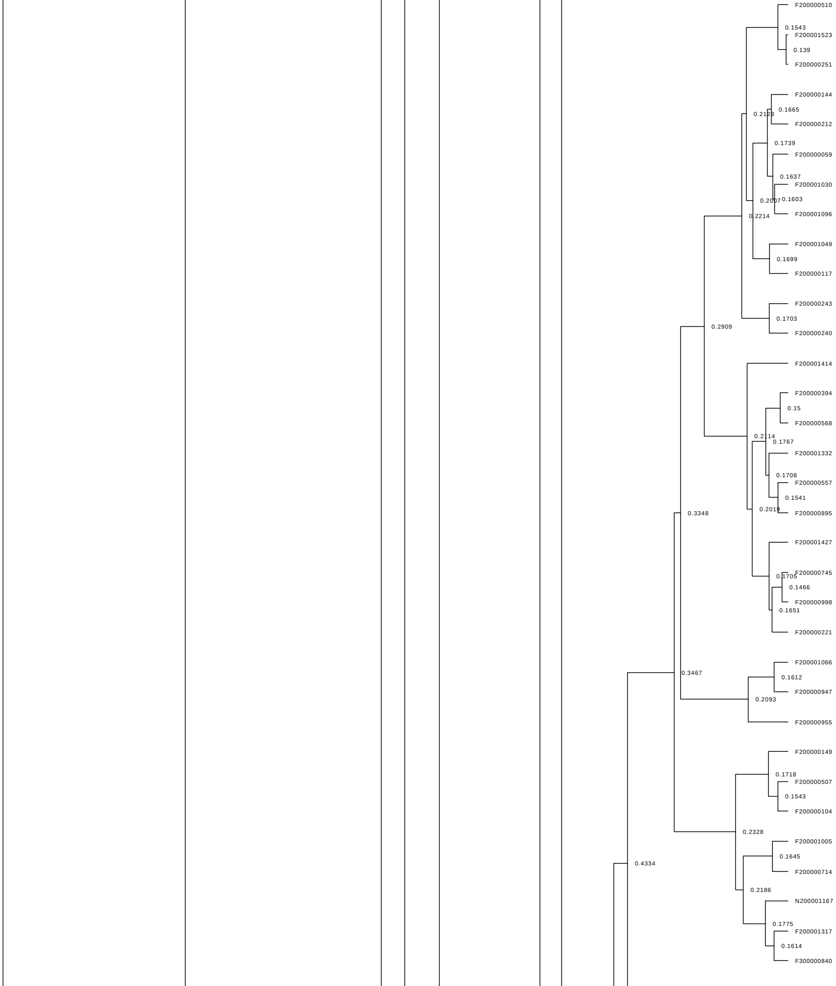

F200000510
0.1543
F200001523
0.139
F200000251
F200000144
0.2128
0.1665
F200000212
0.1739
F200000059
0.1637
F200001030
0.2007
0.1603
F200001096
0.2214
F200001049
0.1699
F200000117
F200000243
0.1703
F200000240
0.2909
F200001414
F200000394
0.15
F200000568
0.2114
0.1767
F200001332
0.1708
F200000557
0.1541
0.2019
F200000895
0.3348
F200001427
0.1705
F200000745
0.1466
F200000998
0.1651
F200000221
F200001066
0.3467
0.1612
F200000947
0.2093
F200000955
F200000149
0.1718
F200000507
0.1543
F200000104
0.2328
F200001005
0.1645
0.4334
F200000714
0.2186
N200001167
0.1775
F200001317
0.1614
F300000840

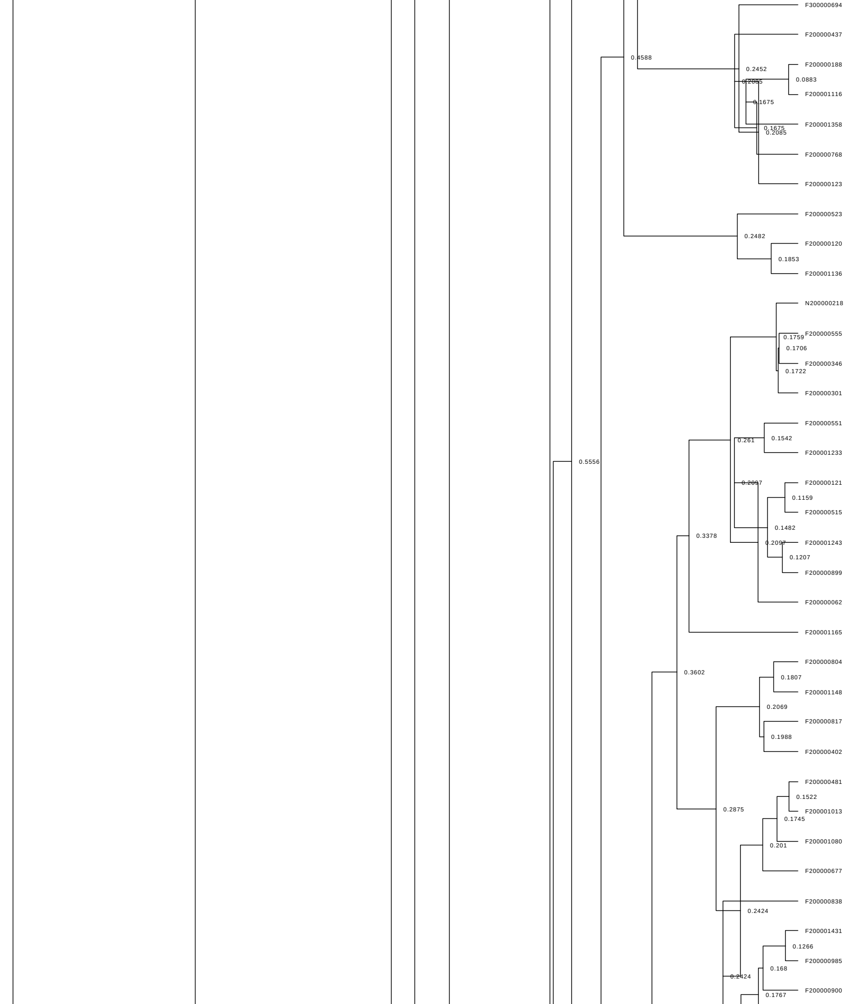
F300000694
F200000437
F200000188
F200001116
F200001358
F200000768
F200000123
F200000523
F200000120
F200001136
N200000218
F200000555
F200000346
F200000301
F200000551
F200001233
F200000121
F200000515
F200001243
F200000899
F200000062
F200001165
F200000804
F200001148
F200000817
F200000402
F200000481
F200001013
F200001080
F200000677
F200000838
F200001431
F200000985
F200000900
0.4588
0.2452
0.0883
0.2085
0.1675
0.1675
0.2085
0.2482
0.1853
0.5556
0.1759
0.1706
0.1722
0.261
0.1542
0.2097
0.1159
0.1482
0.2097
0.1207
0.3378
0.3602
0.1807
0.2069
0.1988
0.1522
0.2875
0.1745
0.201
0.2424
0.1266
0.168
0.2424
0.1767

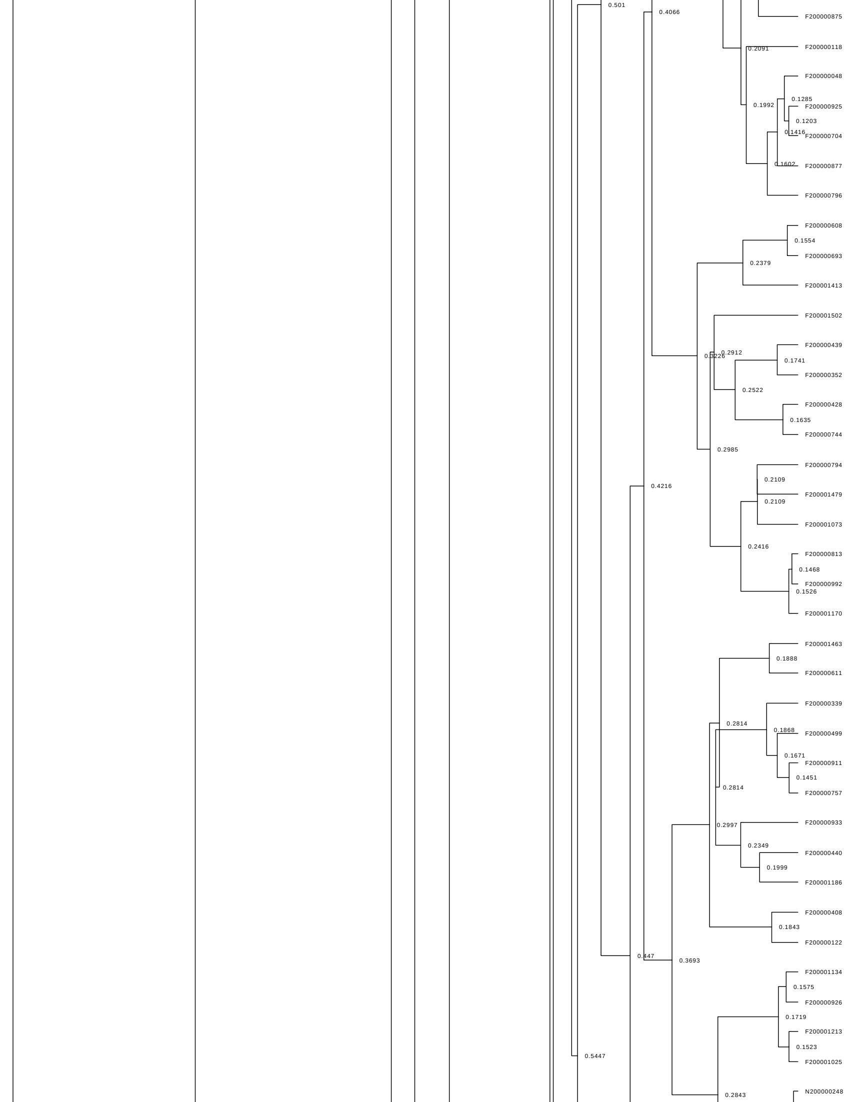
0.501
0.4066
F200000875
F200000118
0.2091
F200000048
0.1285
0.1992
F200000925
0.1203
0.1416
F200000704
0.1602
F200000877
F200000796
F200000608
0.1554
F200000693
0.2379
F200001413
F200001502
F200000439
0.2912
0.3226
0.1741
F200000352
0.2522
F200000428
0.1635
F200000744
0.2985
F200000794
0.2109
0.4216
F200001479
0.2109
F200001073
0.2416
F200000813
0.1468
F200000992
0.1526
F200001170
F200001463
0.1888
F200000611
F200000339
0.2814
0.1868
F200000499
0.1671
F200000911
0.1451
0.2814
F200000757
F200000933
0.2997
0.2349
F200000440
0.1999
F200001186
F200000408
0.1843
F200000122
0.447
0.3693
F200001134
0.1575
F200000926
0.1719
F200001213
0.1523
0.5447
F200001025
N200000248
0.2843

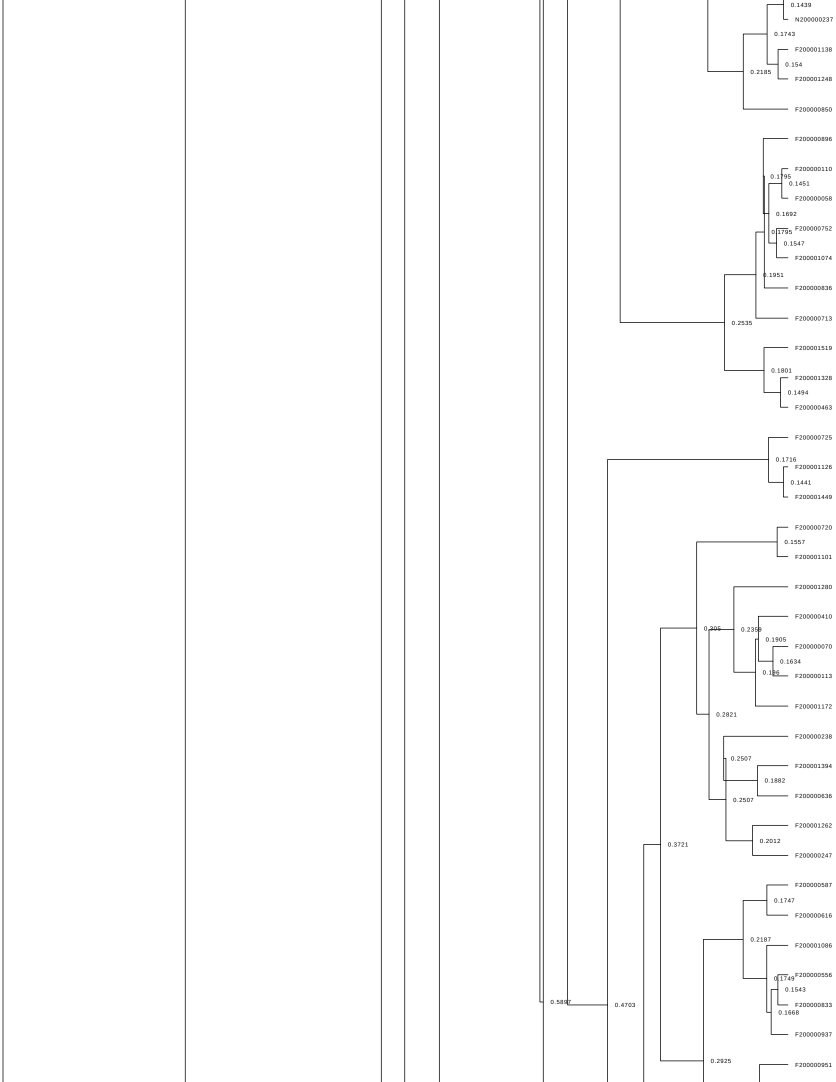

0.1439
N200000237
0.1743
F200001138
0.154
0.2185
F200001248
F200000850
F200000896
F200000110
0.1795
0.1451
F200000058
0.1692
F200000752
0.1795
0.1547
F200001074
0.1951
F200000836
F200000713
0.2535
F200001519
0.1801
F200001328
0.1494
F200000463
F200000725
0.1716
F200001126
0.1441
F200001449
F200000720
0.1557
F200001101
F200001280
F200000410
0.305
0.2359
0.1905
F200000070
0.1634
0.196
F200000113
F200001172
0.2821
F200000238
0.2507
F200001394
0.1882
F200000636
0.2507
F200001262
0.2012
0.3721
F200000247
F200000587
0.1747
F200000616
0.2187
F200001086
F200000556
0.1749
0.1543
0.5897
0.4703
F200000833
0.1668
F200000937
0.2925
F200000951

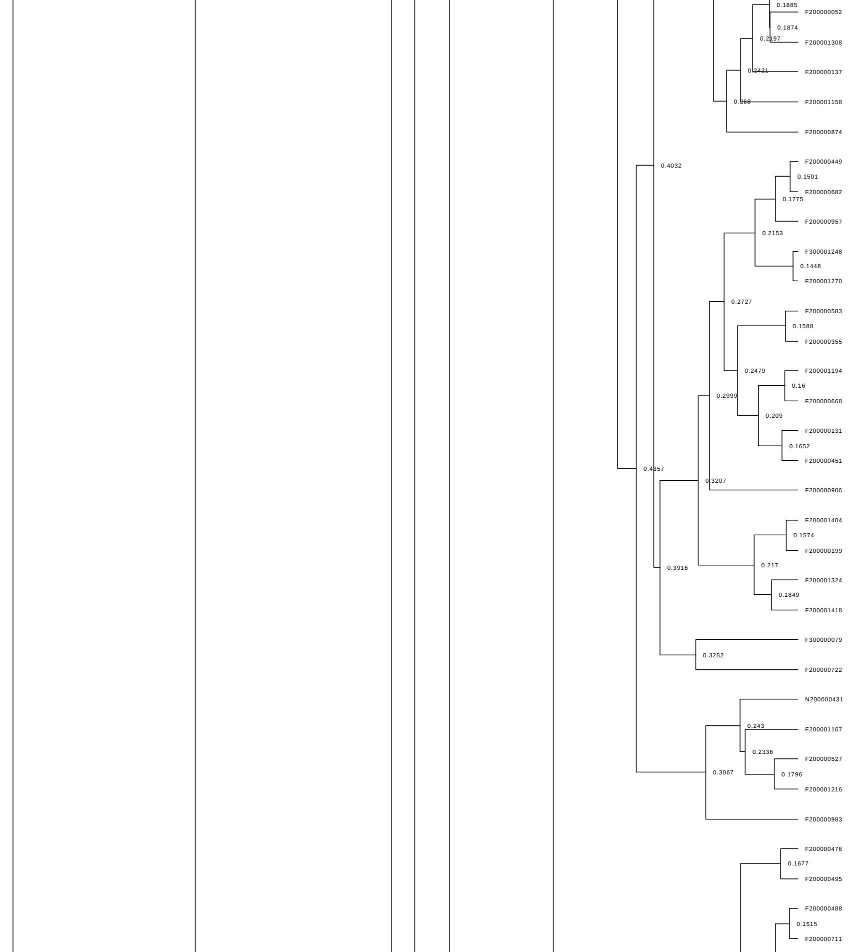
0.1885
F200000052
0.1874
0.2197
F200001308
0.2421
F200000137
0.268
F200001158
F200000874
0.4032
F200000449
0.1501
F200000682
0.1775
F200000957
0.2153
F300001248
0.1448
F200001270
0.2727
F200000583
0.1588
F200000355
0.2479
F200001194
0.16
F200000668
0.2999
0.209
F200000131
0.1652
F200000451
0.4357
0.3207
F200000906
F200001404
0.1574
F200000199
0.217
0.3916
F200001324
0.1849
F200001418
F300000079
0.3252
F200000722
N200000431
0.243
F200001167
0.2336
F200000527
0.3067
0.1796
F200001216
F200000983
F200000476
0.1677
F200000495
F200000488
0.1515
F200000711

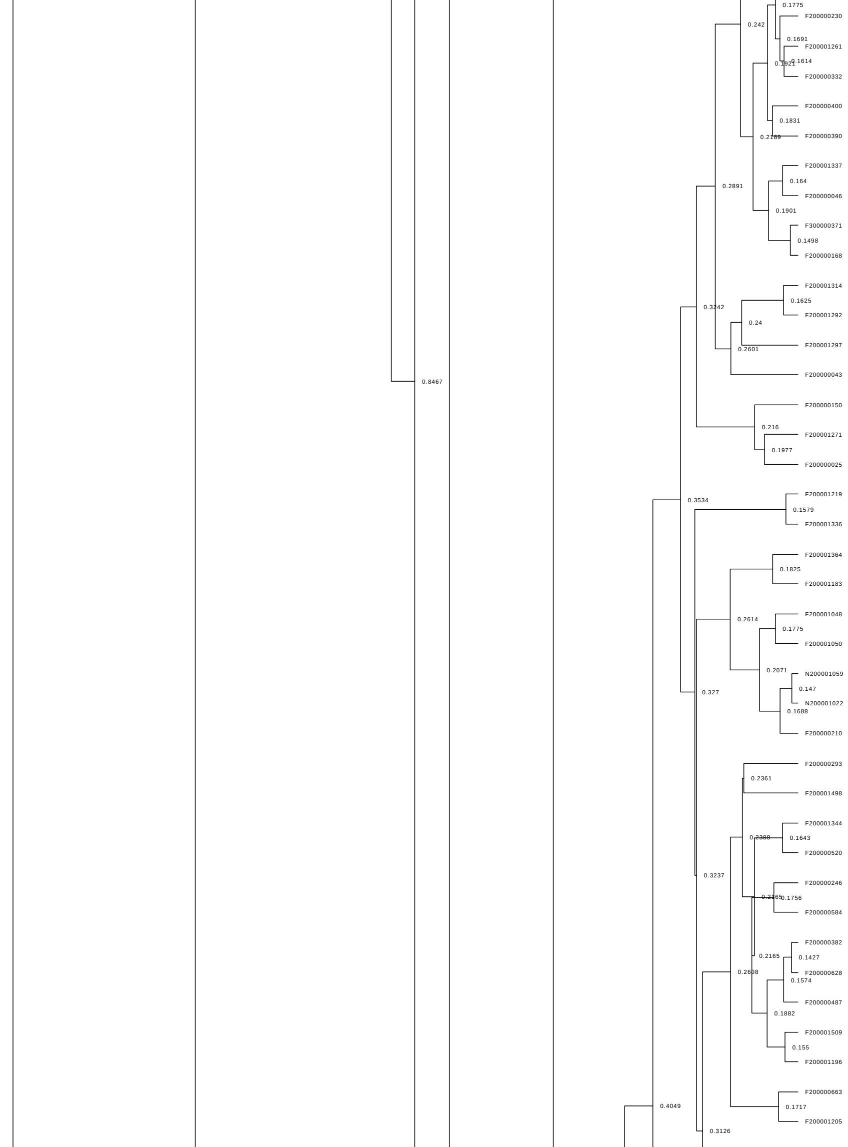
0.1775
F200000230
0.242
0.1691
F200001261
0.1921
0.1614
F200000332
F200000400
0.1831
0.2189
F200000390
F200001337
0.164
F200000046
0.2891
0.1901
F300000371
0.1498
F200000168
F200001314
0.1625
0.3242
F200001292
0.24
F200001297
0.2601
F200000043
0.8467
F200000150
0.216
F200001271
0.1977
F200000025
F200001219
0.3534
0.1579
F200001336
F200001364
0.1825
F200001183
0.2614
F200001048
0.1775
F200001050
0.2071
N200001059
0.147
0.327
N200001022
0.1688
F200000210
F200000293
0.2361
F200001498
F200001344
0.2388
0.1643
F200000520
0.3237
F200000246
0.2165
0.1756
F200000584
F200000382
0.2165
0.1427
F200000628
0.2608
0.1574
F200000487
0.1882
F200001509
0.155
F200001196
F200000663
0.4049
0.1717
F200001205
0.3126

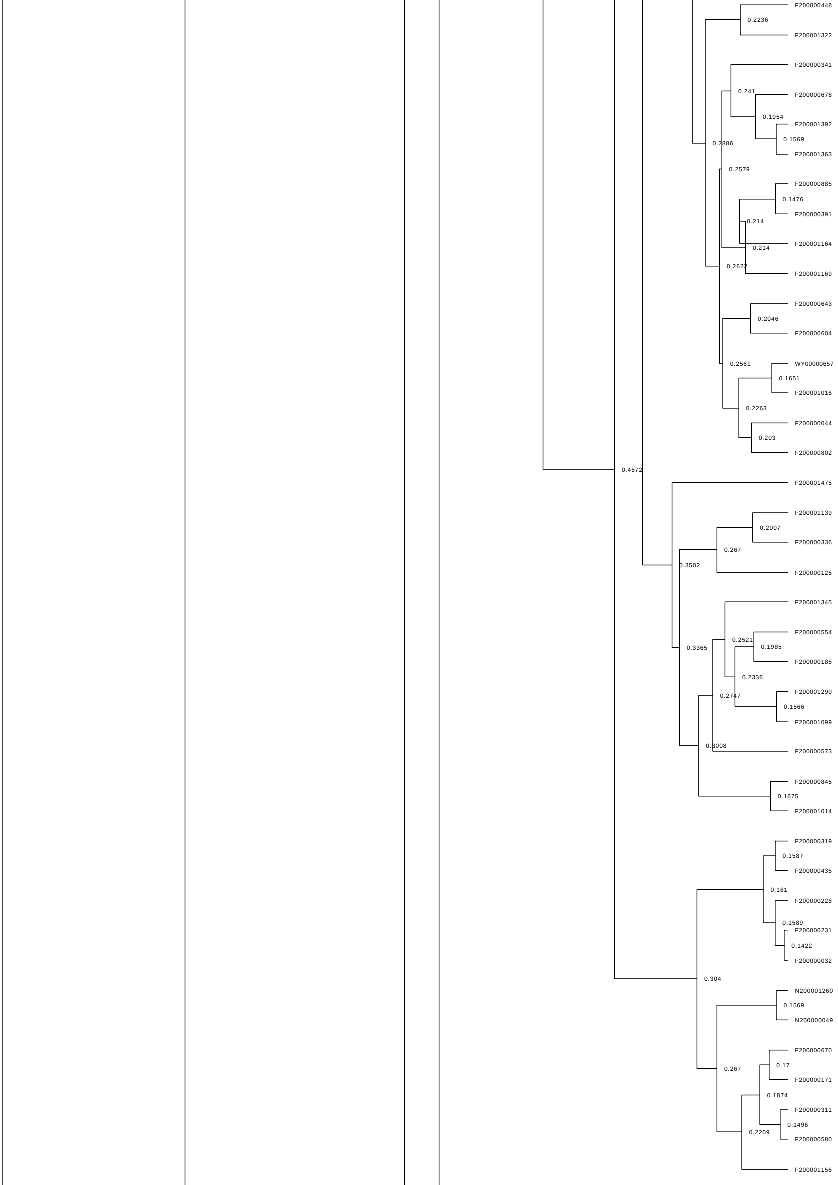
F200000448
0.2236
F200001322
F200000341
0.241
F200000678
0.1954
F200001392
0.1569
F200001363
0.2886
0.2579
F200000885
0.1476
F200000391
0.214
F200001164
0.214
0.2622
F200001169
F200000643
0.2046
F200000604
0.2561
WY00000657
0.1651
F200001016
0.2263
F200000044
0.203
F200000802
0.4572
F200001475
F200001139
0.2007
F200000336
0.267
0.3502
F200000125
F200001345
F200000554
0.2521
0.1985
0.3365
F200000185
0.2336
F200001290
0.2747
0.1566
F200001099
0.3008
F200000573
F200000845
0.1675
F200001014
F200000319
0.1587
F200000435
0.181
F200000228
0.1589
F200000231
0.1422
F200000032
0.304
N200001260
0.1569
N200000049
F200000670
0.17
0.267
F200000171
0.1874
F200000311
0.1496
0.2209
F200000580
F200001156

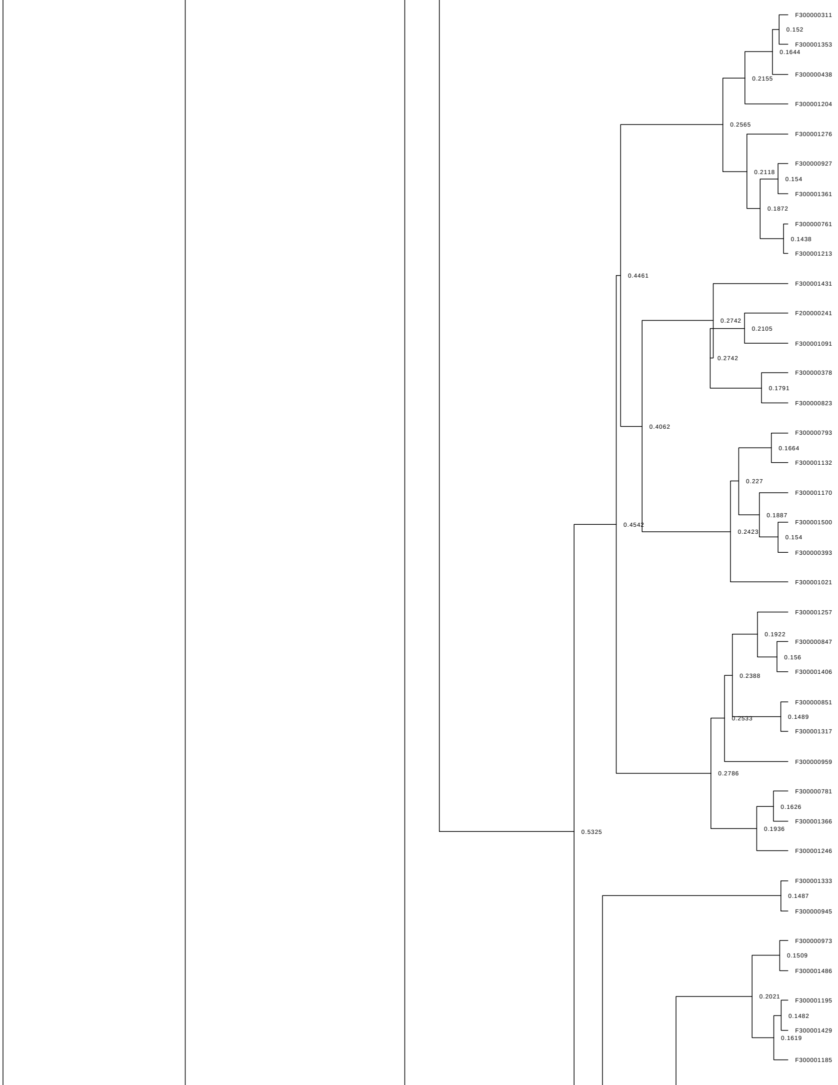
F300000311
0.152
F300001353
0.1644
F300000438
0.2155
F300001204
0.2565
F300001276
F300000927
0.2118
0.154
F300001361
0.1872
F300000761
0.1438
F300001213
0.4461
F300001431
0.2742
F200000241
0.2105
F300001091
0.2742
F300000378
0.1791
F300000823
0.4062
F300000793
0.1664
F300001132
0.227
F300001170
0.1887
F300001500
0.4542
0.2423
0.154
F300000393
F300001021
F300001257
0.1922
F300000847
0.156
F300001406
0.2388
F300000851
0.1489
0.2533
F300001317
F300000959
0.2786
F300000781
0.1626
F300001366
0.5325
0.1936
F300001246
F300001333
0.1487
F300000945
F300000973
0.1509
F300001486
0.2021
F300001195
0.1482
F300001429
0.1619
F300001185

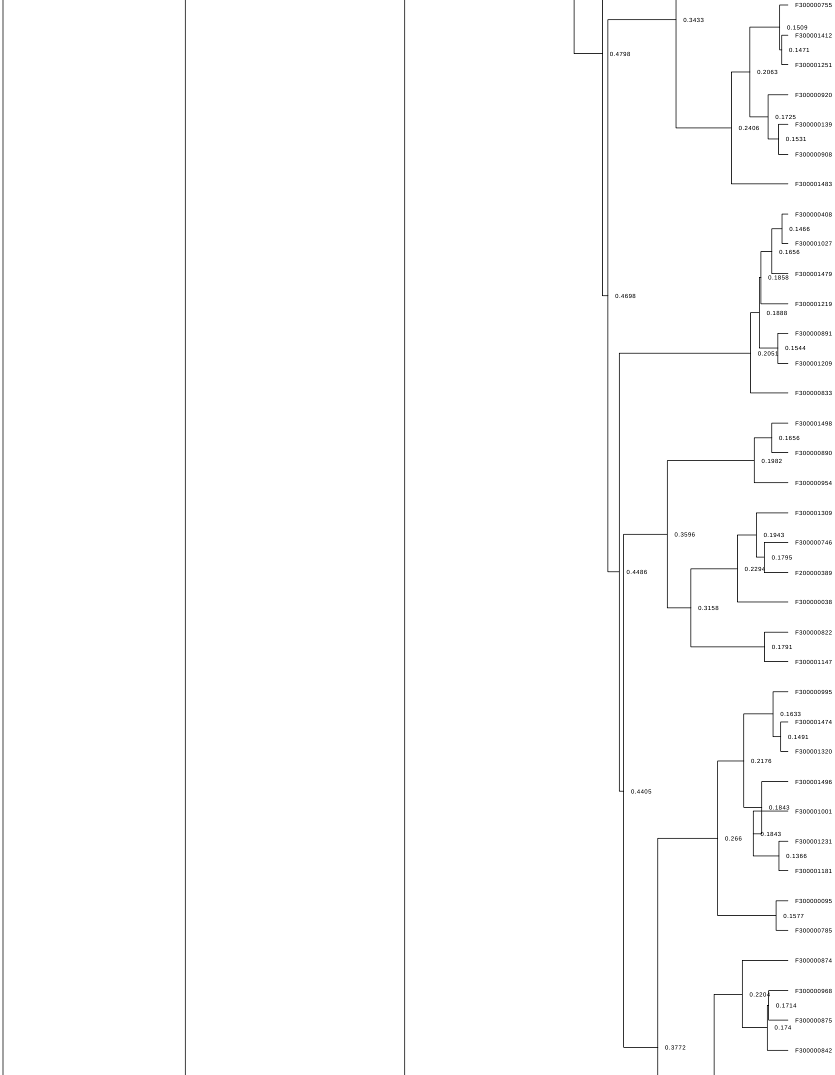

F300000755
0.3433
0.1509
F300001412
0.4798
0.1471
F300001251
0.2063
F300000920
0.1725
F300000139
0.2406
0.1531
F300000908
F300001483
F300000408
0.1466
F300001027
0.1656
F300001479
0.1858
0.4698
F300001219
0.1888
F300000891
0.1544
0.2051
F300001209
F300000833
F300001498
0.1656
F300000890
0.1982
F300000954
F300001309
0.3596
0.1943
F300000746
0.1795
0.4486
0.2294
F200000389
F300000038
0.3158
F300000822
0.1791
F300001147
F300000995
0.1633
F300001474
0.1491
F300001320
0.2176
F300001496
0.4405
0.1843
F300001001
0.1843
0.266
F300001231
0.1366
F300001181
F300000095
0.1577
F300000785
F300000874
F300000968
0.2204
0.1714
F300000875
0.174
0.3772
F300000842

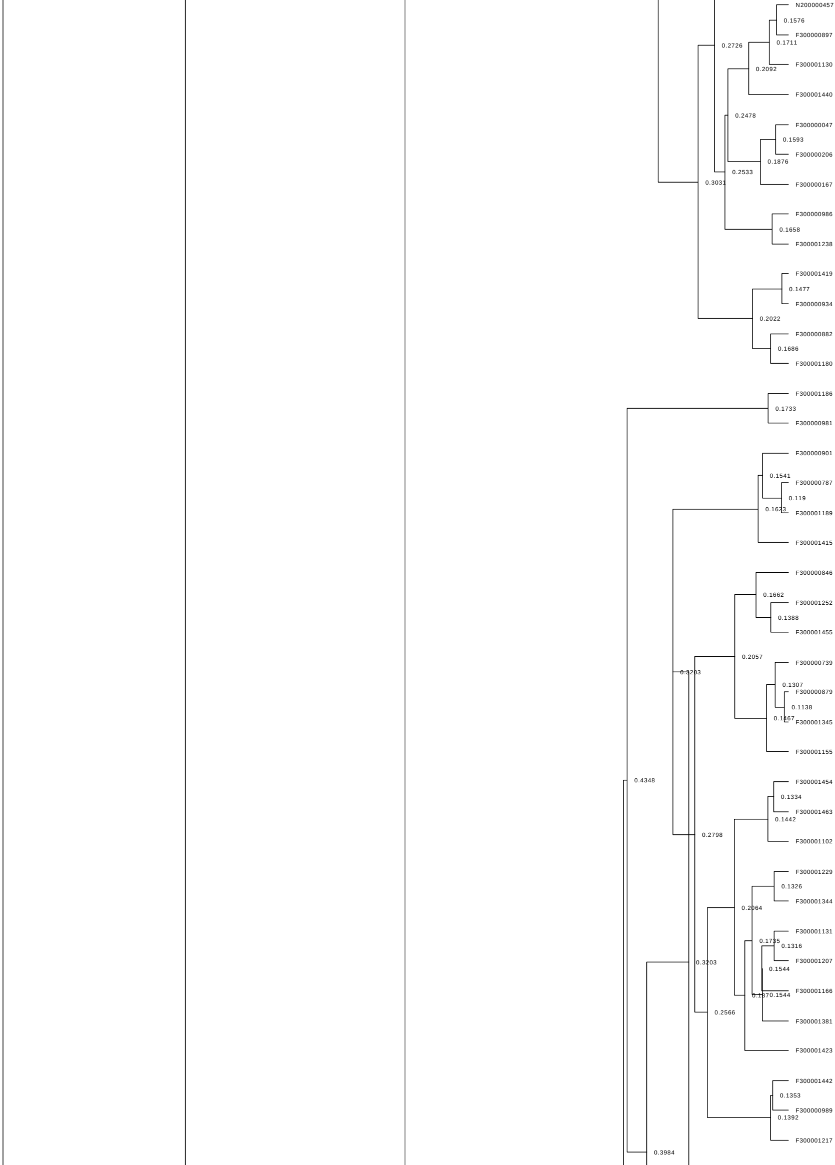

N200000457
0.1576
F300000897
0.1711
0.2726
F300001130
0.2092
F300001440
0.2478
F300000047
0.1593
F300000206
0.1876
0.2533
0.3031
F300000167
F300000986
0.1658
F300001238
F300001419
0.1477
F300000934
0.2022
F300000882
0.1686
F300001180
F300001186
0.1733
F300000981
F300000901
0.1541
F300000787
0.119
0.1623
F300001189
F300001415
F300000846
0.1662
F300001252
0.1388
F300001455
0.2057
F300000739
0.3203
0.1307
F300000879
0.1138
0.1467
F300001345
F300001155
0.4348
F300001454
0.1334
F300001463
0.1442
F300001102
0.2798
F300001229
0.1326
F300001344
0.2064
F300001131
0.1735
0.1316
F300001207
0.3203
0.1544
F300001166
0.137
0.1544
0.2566
F300001381
F300001423
F300001442
0.1353
F300000989
0.1392
F300001217
0.3984

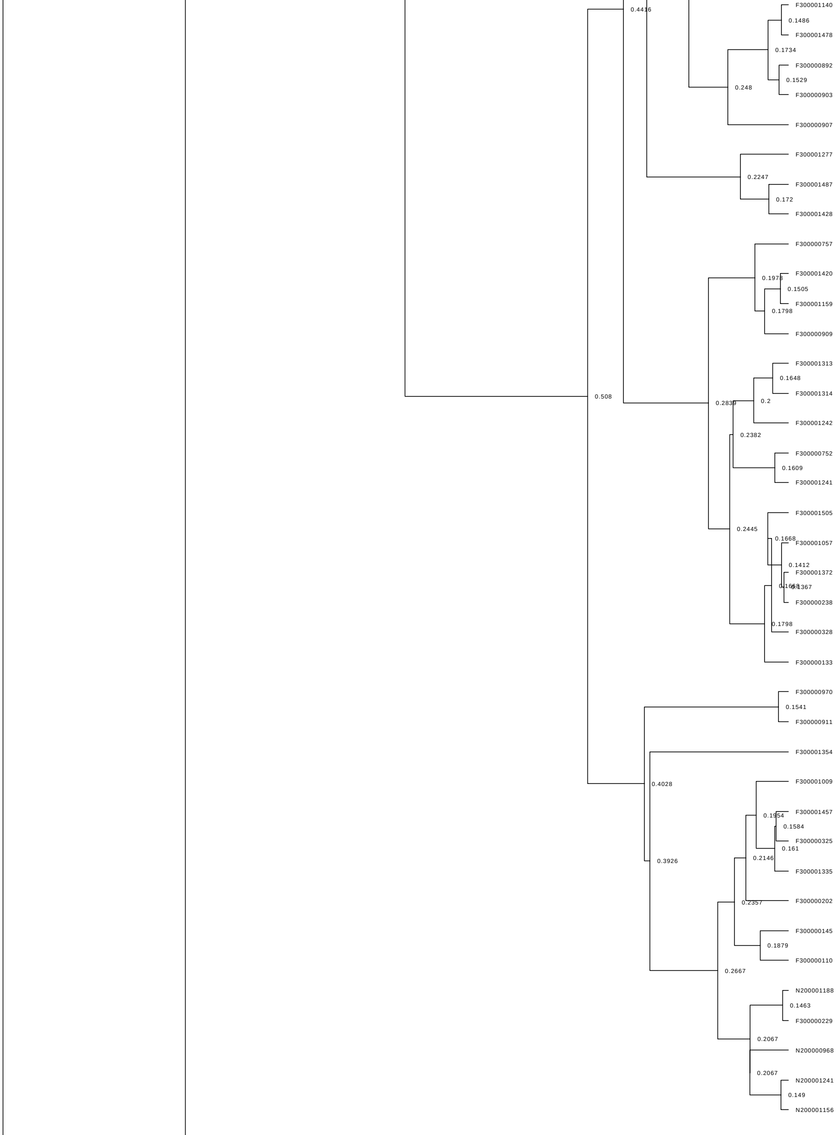
F300001140
0.4416
0.1486
F300001478
0.1734
F300000892
0.1529
0.248
F300000903
F300000907
F300001277
0.2247
F300001487
0.172
F300001428
F300000757
F300001420
0.1978
0.1505
F300001159
0.1798
F300000909
F300001313
0.1648
0.508
F300001314
0.2839
0.2
F300001242
0.2382
F300000752
0.1609
F300001241
F300001505
0.2445
0.1668
F300001057
0.1412
F300001372
0.1367
F300000238
0.1798
F300000328
F300000133
F300000970
0.1541
F300000911
F300001354
0.4028
F300001009
F300001457
0.1954
0.1584
F300000325
0.161
0.2146
0.3926
F300001335
F300000202
0.2357
F300000145
0.1879
F300000110
0.2667
N200001188
0.1463
F300000229
0.2067
N200000968
0.2067
N200001241
0.149
N200001156

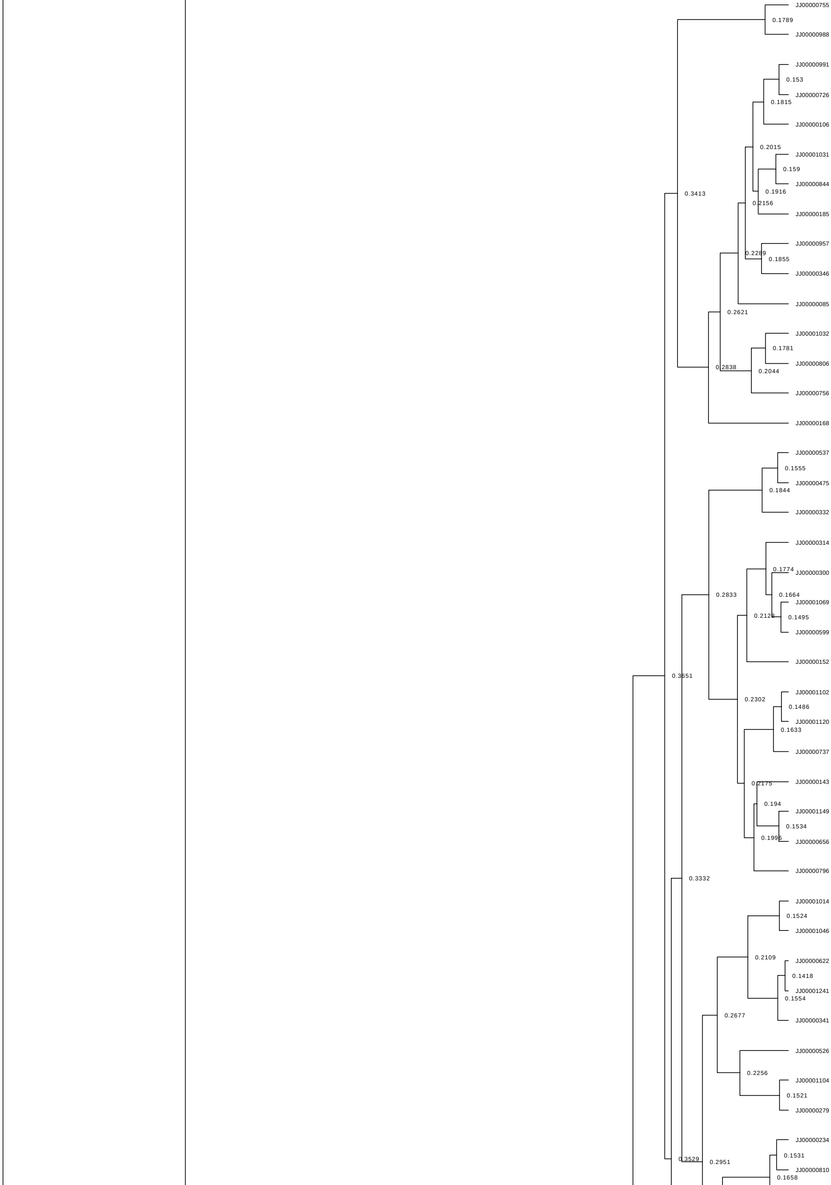
JJ00000755
0.1789
JJ00000988
JJ00000991
0.153
JJ00000726
0.1815
JJ00000106
0.2015
JJ00001031
0.159
JJ00000844
0.1916
0.2156
JJ00000185
JJ00000957
0.2289
0.1855
JJ00000346
JJ00000085
0.2621
JJ00001032
0.1781
JJ00000806
0.2838
0.2044
JJ00000756
0.3413
JJ00000168
JJ00000537
0.1555
JJ00000475
0.1844
JJ00000332
JJ00000314
0.1774
JJ00000300
0.2833
0.1664
JJ00001069
0.2128
0.1495
JJ00000599
JJ00000152
0.3651
JJ00001102
0.2302
0.1486
JJ00001120
0.1633
JJ00000737
0.2175
JJ00000143
0.194
JJ00001149
0.1534
0.1996
JJ00000656
JJ00000796
0.3332
JJ00001014
0.1524
JJ00001046
0.2109
JJ00000622
0.1418
JJ00001241
0.1554
0.2677
JJ00000341
JJ00000526
0.2256
JJ00001104
0.1521
JJ00000279
JJ00000234
0.1531
0.3529
0.2951
JJ00000810
0.1658

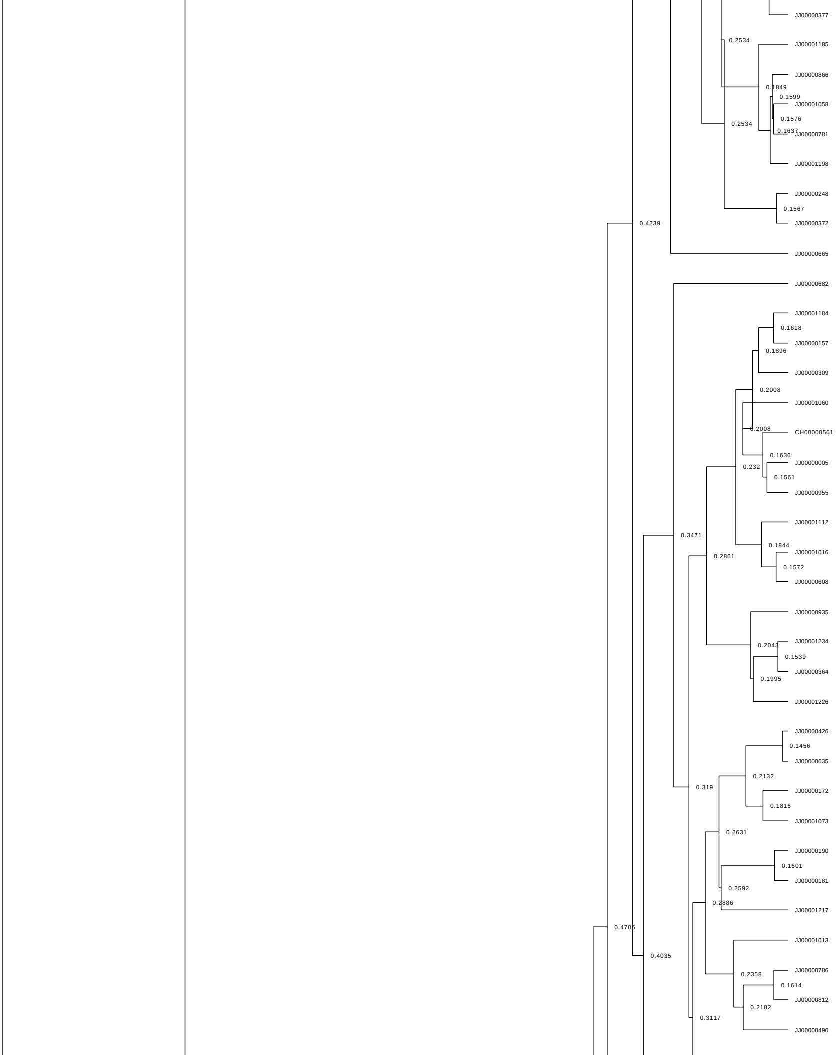
JJ00000377
0.2534
JJ00001185
JJ00000866
0.1849
0.1599
JJ00001058
0.1576
0.2534
0.1637
JJ00000781
JJ00001198
JJ00000248
0.1567
0.4239
JJ00000372
JJ00000665
JJ00000682
JJ00001184
0.1618
JJ00000157
0.1896
JJ00000309
0.2008
JJ00001060
0.2008
CH00000561
0.1636
JJ00000005
0.232
0.1561
JJ00000955
JJ00001112
0.3471
0.1844
0.2861
JJ00001016
0.1572
JJ00000608
JJ00000935
JJ00001234
0.2043
0.1539
JJ00000364
0.1995
JJ00001226
JJ00000426
0.1456
JJ00000635
0.2132
0.319
JJ00000172
0.1816
JJ00001073
0.2631
JJ00000190
0.1601
JJ00000181
0.2592
0.2886
JJ00001217
0.4706
JJ00001013
0.4035
JJ00000786
0.2358
0.1614
JJ00000812
0.2182
0.3117
JJ00000490

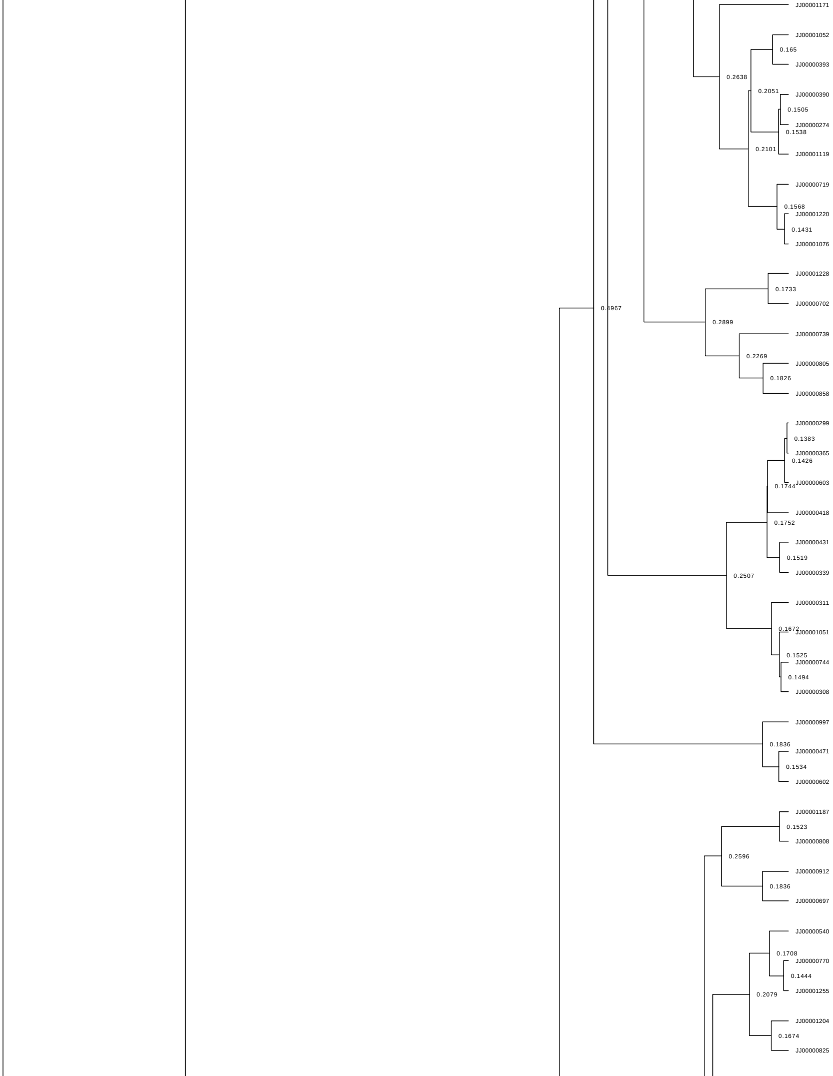

JJ00001171
JJ00001052
0.165
JJ00000393
0.2638
0.2051
JJ00000390
0.1505
JJ00000274
0.1538
0.2101
JJ00001119
JJ00000719
0.1568
JJ00001220
0.1431
JJ00001076
JJ00001228
0.1733
0.4967
JJ00000702
0.2899
JJ00000739
0.2269
JJ00000805
0.1826
JJ00000858
JJ00000299
0.1383
JJ00000365
0.1426
0.1744
JJ00000603
JJ00000418
0.1752
JJ00000431
0.1519
JJ00000339
0.2507
JJ00000311
0.1672
JJ00001051
0.1525
JJ00000744
0.1494
JJ00000308
JJ00000997
0.1836
JJ00000471
0.1534
JJ00000602
JJ00001187
0.1523
JJ00000808
0.2596
JJ00000912
0.1836
JJ00000697
JJ00000540
0.1708
JJ00000770
0.1444
JJ00001255
0.2079
JJ00001204
0.1674
JJ00000825

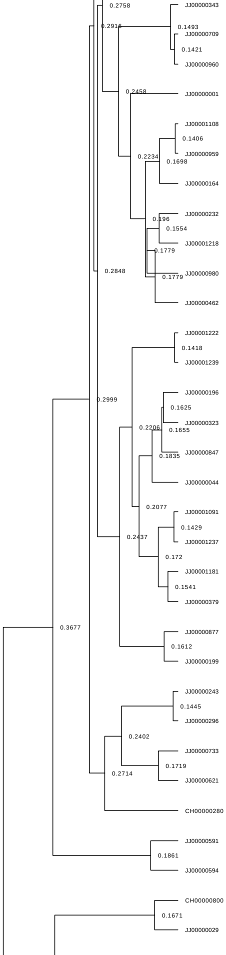

0.2758
JJ00000343
0.2916
0.1493
JJ00000709
0.1421
JJ00000960
0.2458
JJ00000001
JJ00001108
0.1406
JJ00000959
0.2234
0.1698
JJ00000164
JJ00000232
0.196
0.1554
JJ00001218
0.1779
0.2848
JJ00000980
0.1779
JJ00000462
JJ00001222
0.1418
JJ00001239
JJ00000196
0.2999
0.1625
JJ00000323
0.2206
0.1655
JJ00000847
0.1835
JJ00000044
0.2077
JJ00001091
0.1429
0.2437
JJ00001237
0.172
JJ00001181
0.1541
JJ00000379
0.3677
JJ00000877
0.1612
JJ00000199
JJ00000243
0.1445
JJ00000296
0.2402
JJ00000733
0.1719
0.2714
JJ00000621
CH00000280
JJ00000591
0.1861
JJ00000594
CH00000800
0.1671
JJ00000029

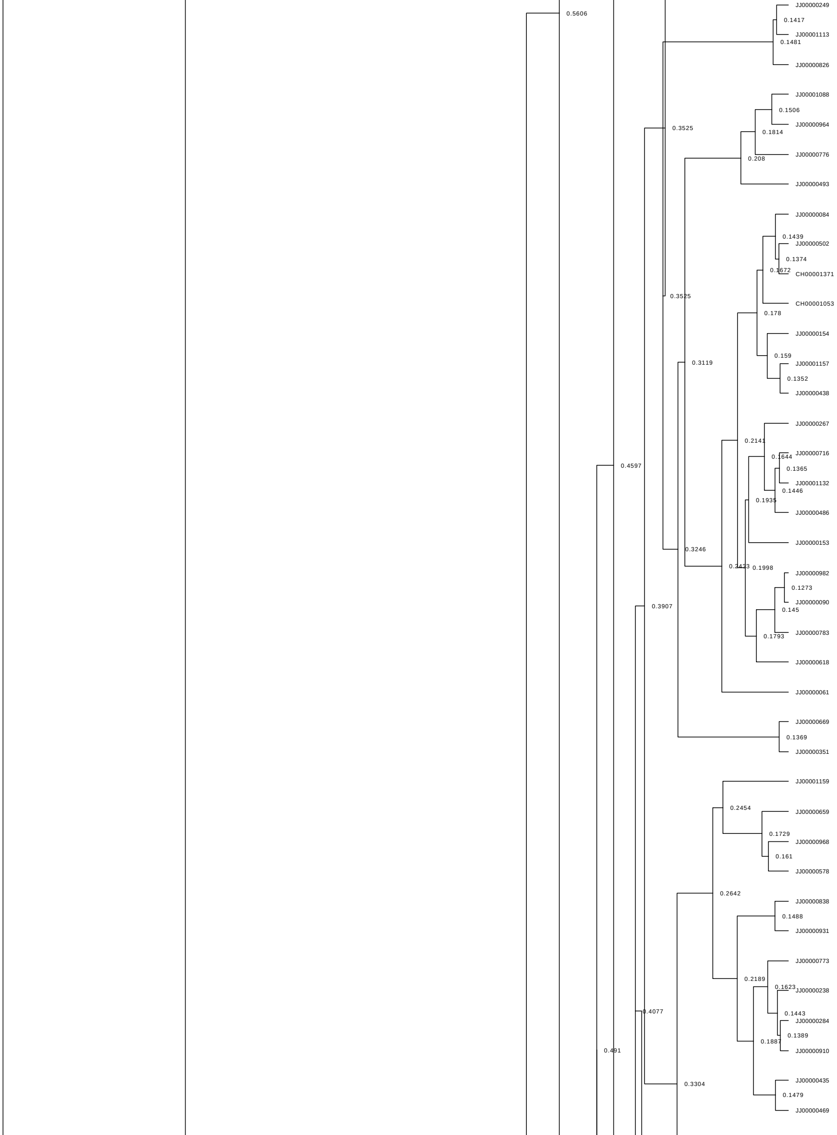
0.5606
JJ00000249
0.1417
JJ00001113
0.1481
JJ00000826
JJ00001088
0.1506
JJ00000964
0.3525
0.1814
JJ00000776
0.208
JJ00000493
JJ00000084
0.1439
JJ00000502
0.1374
0.1672
CH00001371
0.3525
CH00001053
0.178
JJ00000154
0.159
0.3119
JJ00001157
0.1352
JJ00000438
JJ00000267
0.2141
0.1644
JJ00000716
0.4597
0.1365
JJ00001132
0.1446
0.1935
JJ00000486
JJ00000153
0.3246
0.2433
0.1998
JJ00000982
0.1273
JJ00000090
0.3907
0.145
JJ00000783
0.1793
JJ00000618
JJ00000061
JJ00000669
0.1369
JJ00000351
JJ00001159
0.2454
JJ00000659
0.1729
JJ00000968
0.161
JJ00000578
0.2642
JJ00000838
0.1488
JJ00000931
JJ00000773
0.2189
0.1623
JJ00000238
0.4077
0.1443
JJ00000284
0.1389
0.1887
JJ00000910
0.491
0.3304
JJ00000435
0.1479
JJ00000469

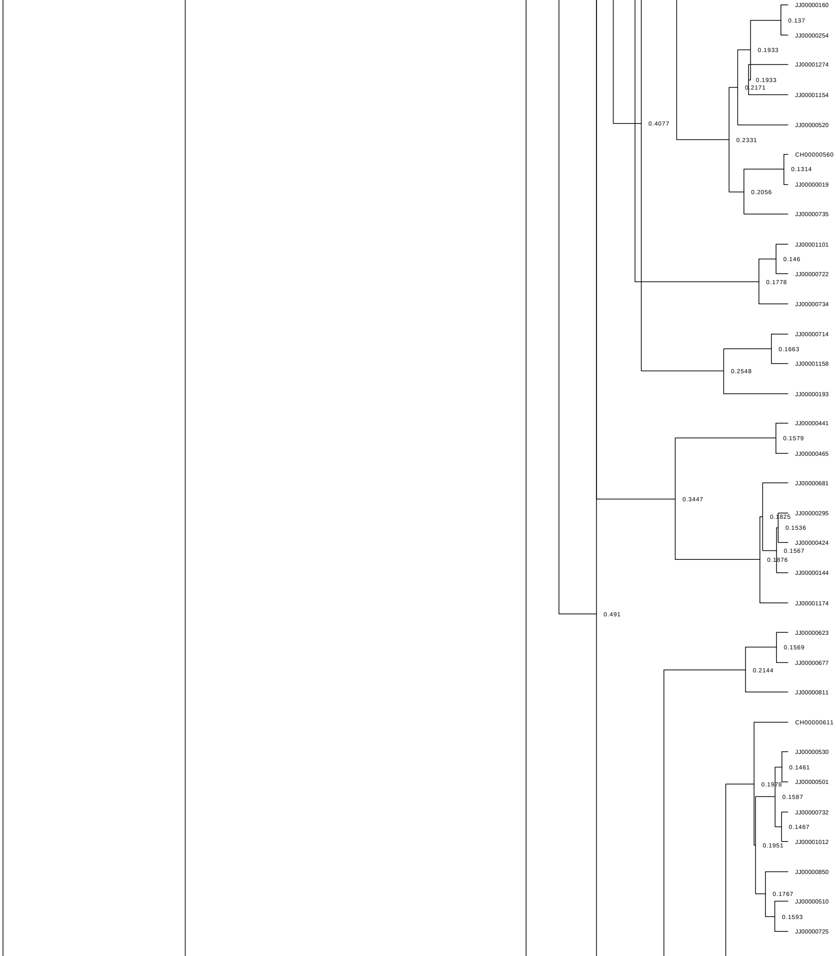
JJ00000160
0.137
JJ00000254
0.1933
JJ00001274
0.1933
0.2171
JJ00001154
0.4077
JJ00000520
0.2331
CH00000560
0.1314
JJ00000019
0.2056
JJ00000735
JJ00001101
0.146
JJ00000722
0.1778
JJ00000734
JJ00000714
0.1663
JJ00001158
0.2548
JJ00000193
JJ00000441
0.1579
JJ00000465
JJ00000681
0.3447
JJ00000295
0.1825
0.1536
JJ00000424
0.1567
0.1876
JJ00000144
JJ00001174
0.491
JJ00000623
0.1569
JJ00000677
0.2144
JJ00000811
CH00000611
JJ00000530
0.1461
JJ00000501
0.1978
0.1587
JJ00000732
0.1467
JJ00001012
0.1951
JJ00000850
0.1767
JJ00000510
0.1593
JJ00000725

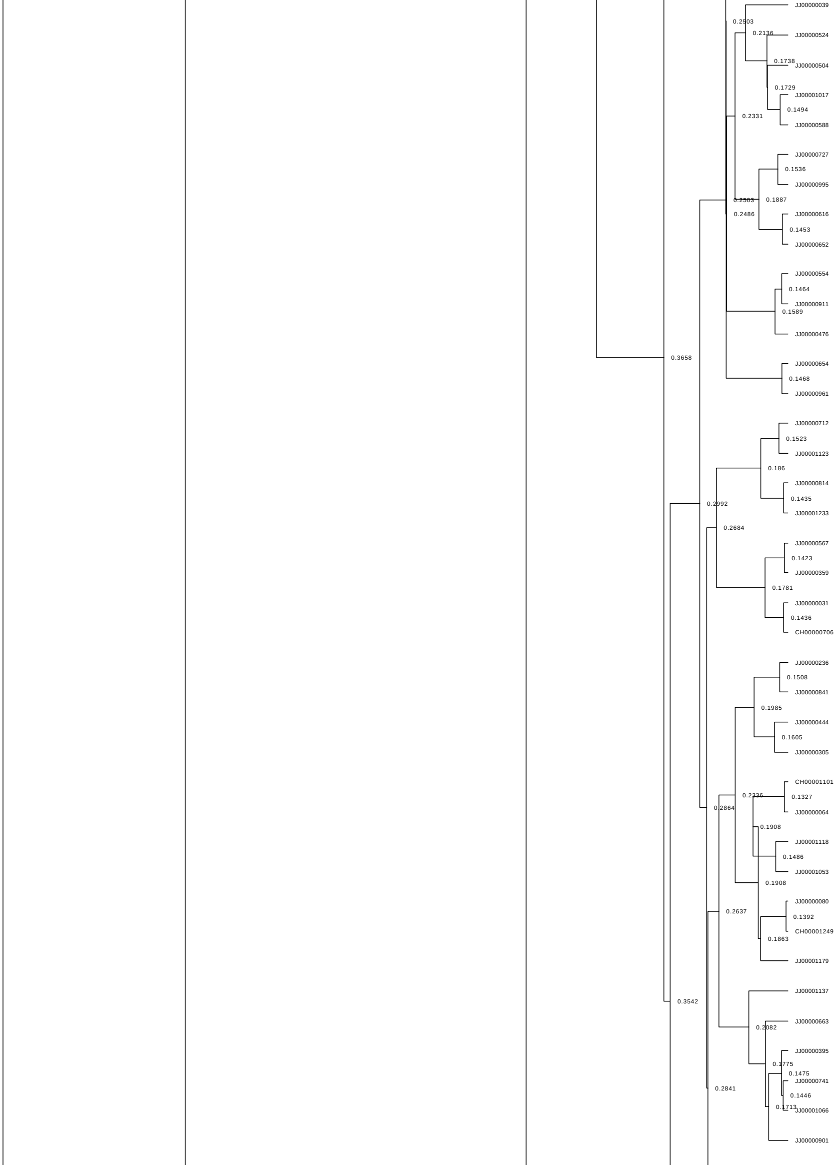

JJ00000039
0.2503
0.2136
JJ00000524
0.1738
JJ00000504
0.1729
JJ00001017
0.1494
0.2331
JJ00000588
JJ00000727
0.1536
JJ00000995
0.2503
0.1887
0.2486
JJ00000616
0.1453
JJ00000652
JJ00000554
0.1464
JJ00000911
0.1589
JJ00000476
0.3658
JJ00000654
0.1468
JJ00000961
JJ00000712
0.1523
JJ00001123
0.186
JJ00000814
0.1435
0.2992
JJ00001233
0.2684
JJ00000567
0.1423
JJ00000359
0.1781
JJ00000031
0.1436
CH00000706
JJ00000236
0.1508
JJ00000841
0.1985
JJ00000444
0.1605
JJ00000305
CH00001101
0.2336
0.1327
0.2864
JJ00000064
0.1908
JJ00001118
0.1486
JJ00001053
0.1908
JJ00000080
0.2637
0.1392
CH00001249
0.1863
JJ00001179
JJ00001137
0.3542
JJ00000663
0.2082
JJ00000395
0.1775
0.1475
JJ00000741
0.2841
0.1446
0.1713
JJ00001066
JJ00000901

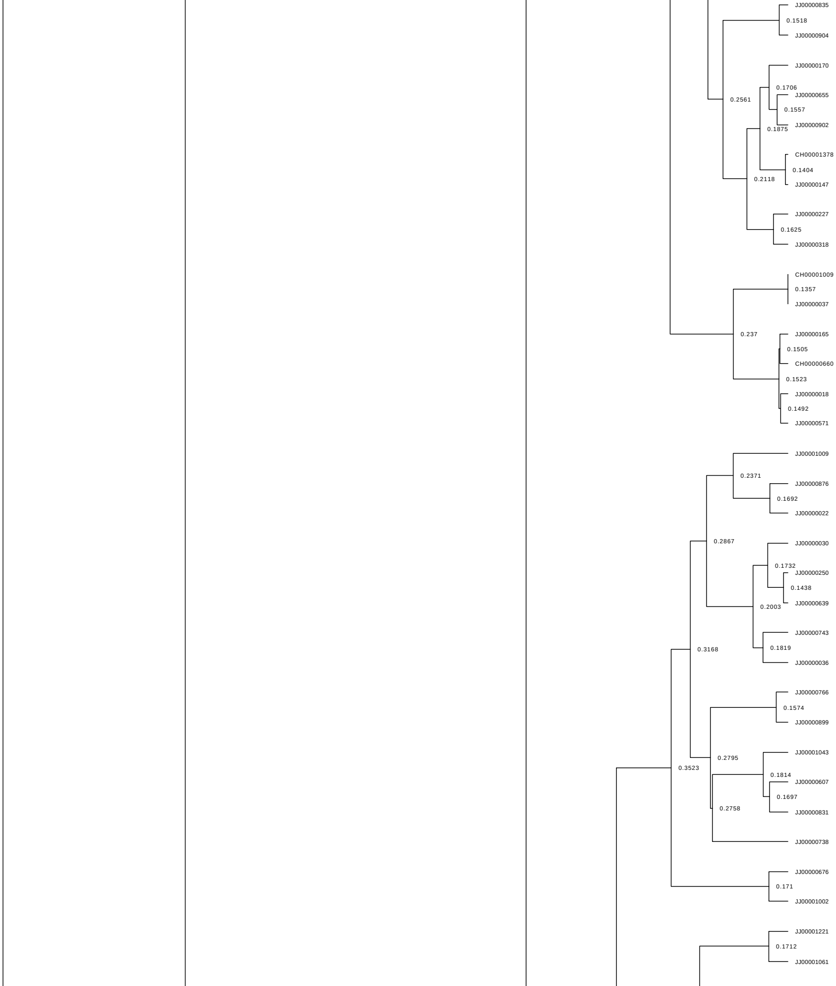

JJ00000835
0.1518
JJ00000904
JJ00000170
0.1706
JJ00000655
0.2561
0.1557
JJ00000902
0.1875
CH00001378
0.1404
0.2118
JJ00000147
JJ00000227
0.1625
JJ00000318
CH00001009
0.1357
JJ00000037
0.237
JJ00000165
0.1505
CH00000660
0.1523
JJ00000018
0.1492
JJ00000571
JJ00001009
0.2371
JJ00000876
0.1692
JJ00000022
0.2867
JJ00000030
0.1732
JJ00000250
0.1438
JJ00000639
0.2003
JJ00000743
0.3168
0.1819
JJ00000036
JJ00000766
0.1574
JJ00000899
JJ00001043
0.2795
0.1814
0.3523
JJ00000607
0.1697
0.2758
JJ00000831
JJ00000738
JJ00000676
0.171
JJ00001002
JJ00001221
0.1712
JJ00001061

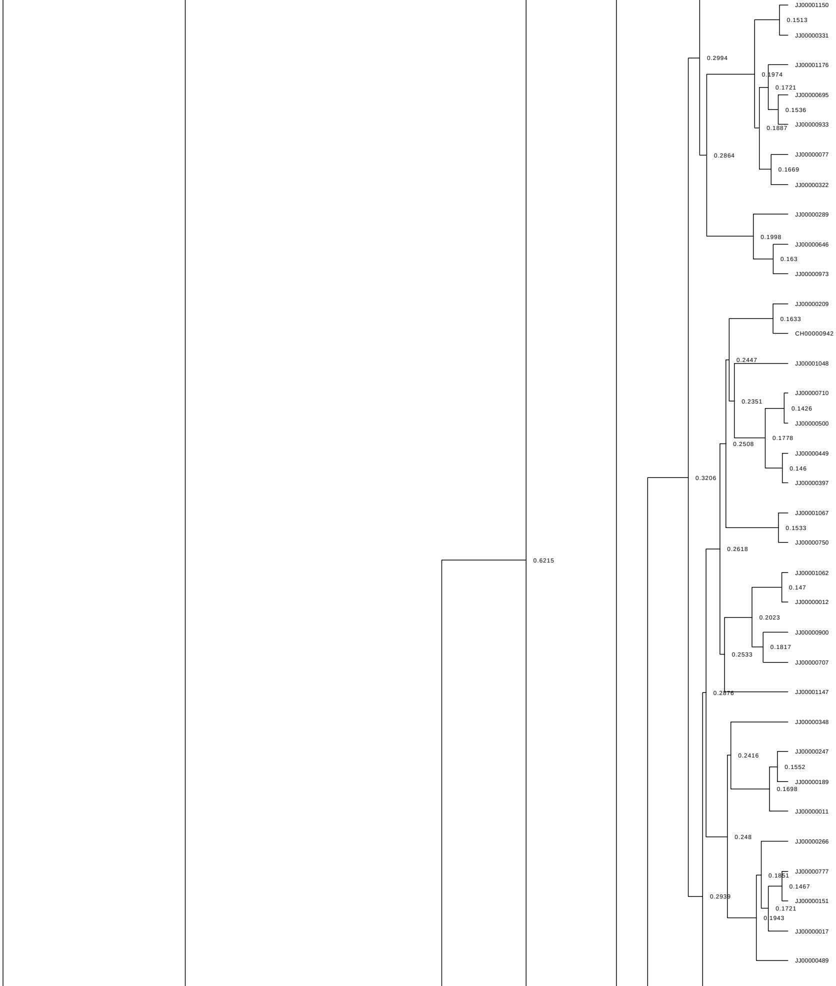
JJ00001150
0.1513
JJ00000331
0.2994
JJ00001176
0.1974
0.1721
JJ00000695
0.1536
JJ00000933
0.1887
0.2864
JJ00000077
0.1669
JJ00000322
JJ00000289
0.1998
JJ00000646
0.163
JJ00000973
JJ00000209
0.1633
CH00000942
0.2447
JJ00001048
0.2351
JJ00000710
0.1426
JJ00000500
0.1778
0.2508
JJ00000449
0.146
0.3206
JJ00000397
JJ00001067
0.1533
JJ00000750
0.6215
0.2618
JJ00001062
0.147
JJ00000012
0.2023
JJ00000900
0.1817
0.2533
JJ00000707
0.2876
JJ00001147
JJ00000348
0.2416
JJ00000247
0.1552
JJ00000189
0.1698
JJ00000011
0.248
JJ00000266
0.1851
JJ00000777
0.1467
0.2939
JJ00000151
0.1721
0.1943
JJ00000017
JJ00000489

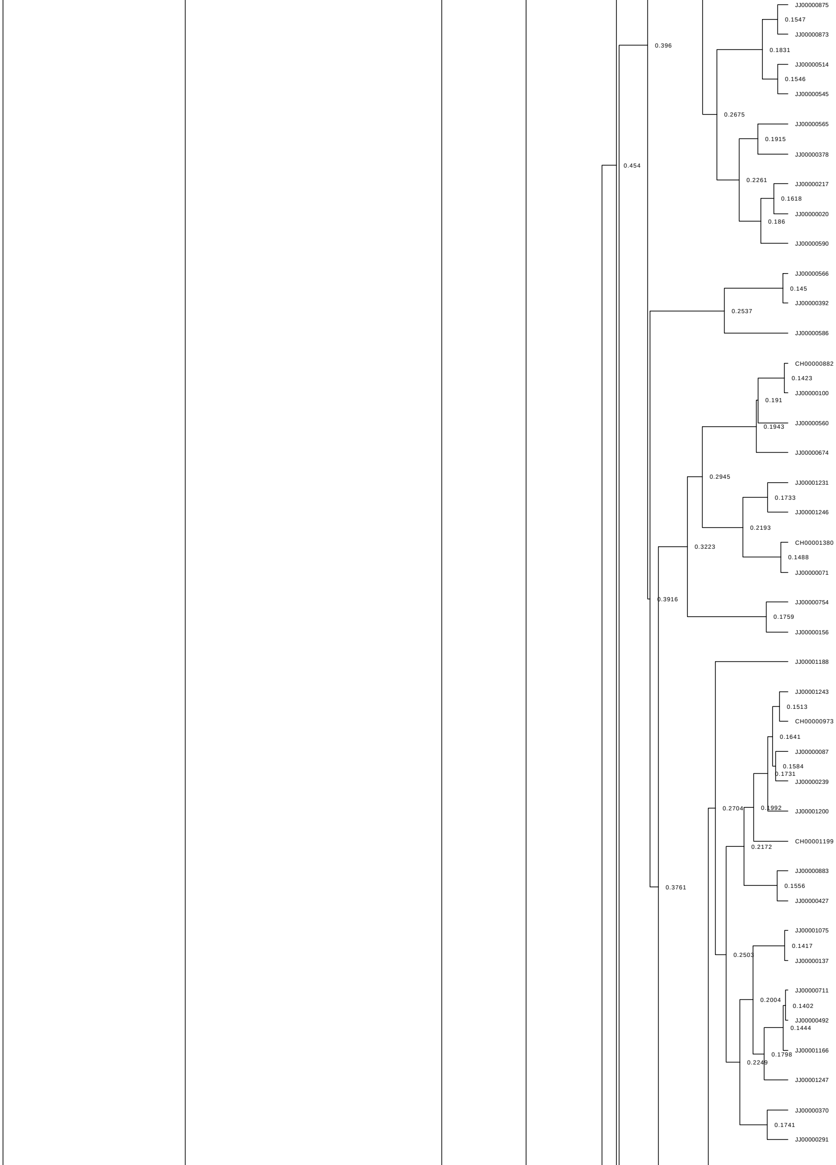
JJ00000875
0.1547
JJ00000873
0.396
0.1831
JJ00000514
0.1546
JJ00000545
0.2675
JJ00000565
0.1915
JJ00000378
0.454
0.2261
JJ00000217
0.1618
JJ00000020
0.186
JJ00000590
JJ00000566
0.145
JJ00000392
0.2537
JJ00000586
CH00000882
0.1423
JJ00000100
0.191
JJ00000560
0.1943
JJ00000674
0.2945
JJ00001231
0.1733
JJ00001246
0.2193
CH00001380
0.3223
0.1488
JJ00000071
0.3916
JJ00000754
0.1759
JJ00000156
JJ00001188
JJ00001243
0.1513
CH00000973
0.1641
JJ00000087
0.1584
0.1731
JJ00000239
0.2704
0.1992
JJ00001200
CH00001199
0.2172
JJ00000883
0.1556
0.3761
JJ00000427
JJ00001075
0.1417
0.2503
JJ00000137
JJ00000711
0.2004
0.1402
JJ00000492
0.1444
JJ00001166
0.1798
0.2249
JJ00001247
JJ00000370
0.1741
JJ00000291

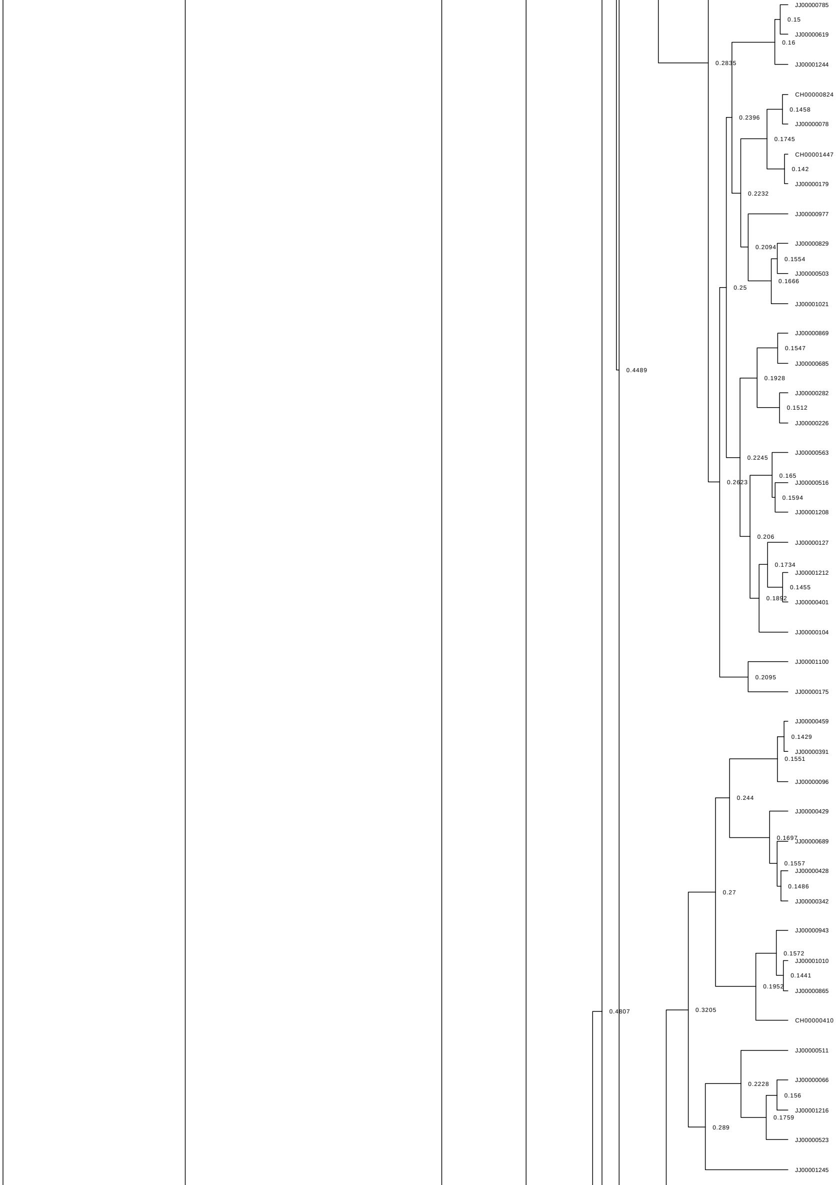

JJ00000785
0.15
JJ00000619
0.16
0.2835
JJ00001244
CH00000824
0.1458
0.2396
JJ00000078
0.1745
CH00001447
0.142
JJ00000179
0.2232
JJ00000977
JJ00000829
0.2094
0.1554
JJ00000503
0.1666
0.25
JJ00001021
JJ00000869
0.1547
JJ00000685
0.4489
0.1928
JJ00000282
0.1512
JJ00000226
JJ00000563
0.2245
0.165
JJ00000516
0.2623
0.1594
JJ00001208
0.206
JJ00000127
0.1734
JJ00001212
0.1455
0.1892
JJ00000401
JJ00000104
JJ00001100
0.2095
JJ00000175
JJ00000459
0.1429
JJ00000391
0.1551
JJ00000096
0.244
JJ00000429
0.1697
JJ00000689
0.1557
JJ00000428
0.1486
0.27
JJ00000342
JJ00000943
0.1572
JJ00001010
0.1441
0.1952
JJ00000865
0.4807
0.3205
CH00000410
JJ00000511
JJ00000066
0.2228
0.156
JJ00001216
0.1759
0.289
JJ00000523
JJ00001245

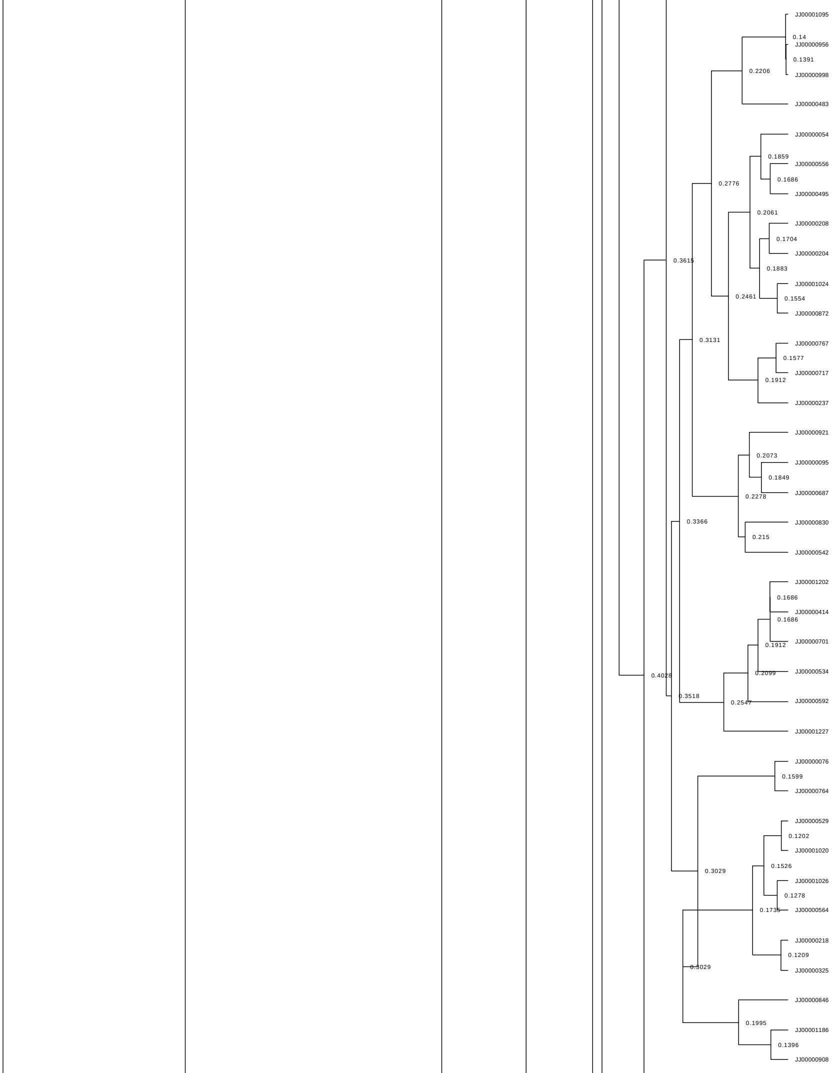

JJ00001095
0.14
JJ00000956
0.1391
JJ00000998
0.2206
JJ00000483
JJ00000054
0.1859
JJ00000556
0.1686
JJ00000495
0.2776
0.2061
JJ00000208
0.1704
JJ00000204
0.3615
0.1883
JJ00001024
0.2461
0.1554
JJ00000872
0.3131
JJ00000767
0.1577
JJ00000717
0.1912
JJ00000237
JJ00000921
0.2073
JJ00000095
0.1849
JJ00000687
0.2278
0.3366
JJ00000830
0.215
JJ00000542
JJ00001202
0.1686
JJ00000414
0.1686
JJ00000701
0.1912
0.4028
0.2099
JJ00000534
0.3518
0.2547
JJ00000592
JJ00001227
JJ00000076
0.1599
JJ00000764
JJ00000529
0.1202
JJ00001020
0.1526
0.3029
JJ00001026
0.1278
0.1735
JJ00000564
JJ00000218
0.1209
JJ00000325
0.3029
JJ00000846
0.1995
JJ00001186
0.1396
JJ00000908

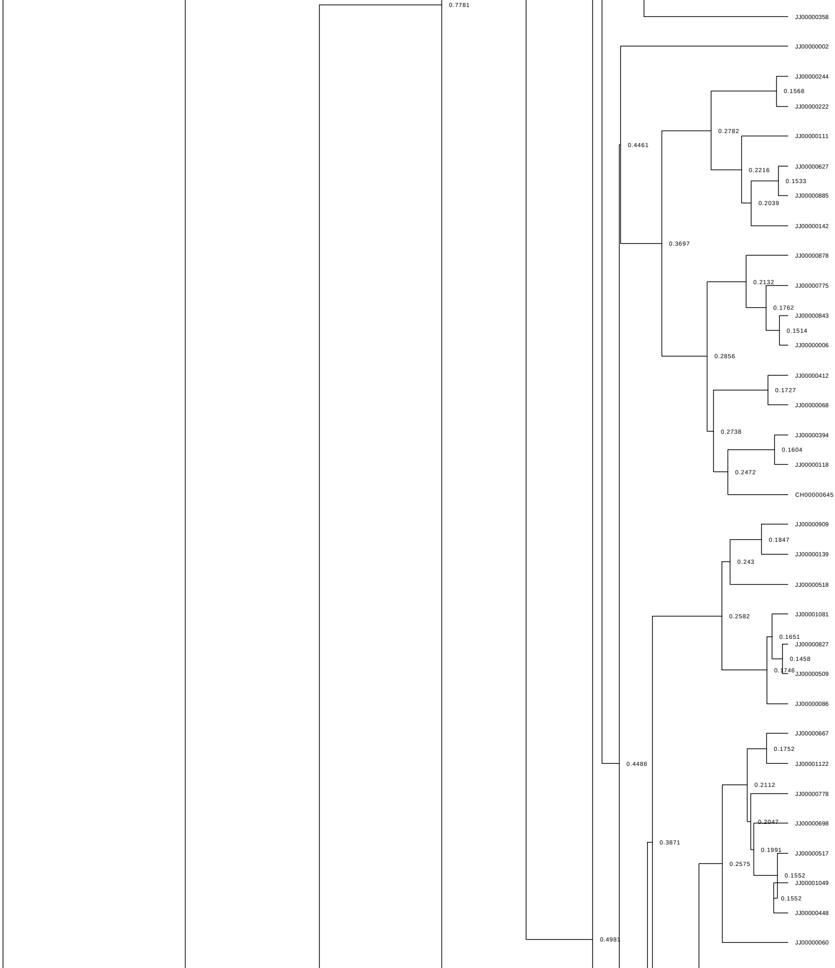
0.7781
JJ00000358
JJ00000002
JJ00000244
0.1568
JJ00000222
0.2782
JJ00000111
0.4461
JJ00000627
0.2216
0.1533
JJ00000885
0.2039
JJ00000142
0.3697
JJ00000878
0.2132
JJ00000775
0.1762
JJ00000843
0.1514
JJ00000006
0.2856
JJ00000412
0.1727
JJ00000068
0.2738
JJ00000394
0.1604
JJ00000118
0.2472
CH00000645
JJ00000909
0.1847
JJ00000139
0.243
JJ00000518
0.2582
JJ00001081
0.1651
JJ00000827
0.1458
0.1746
JJ00000509
JJ00000086
JJ00000667
0.1752
0.4486
JJ00001122
0.2112
JJ00000778
0.2047
JJ00000698
0.3871
0.1991
JJ00000517
0.2575
0.1552
JJ00001049
0.1552
JJ00000448
0.4981
JJ00000060

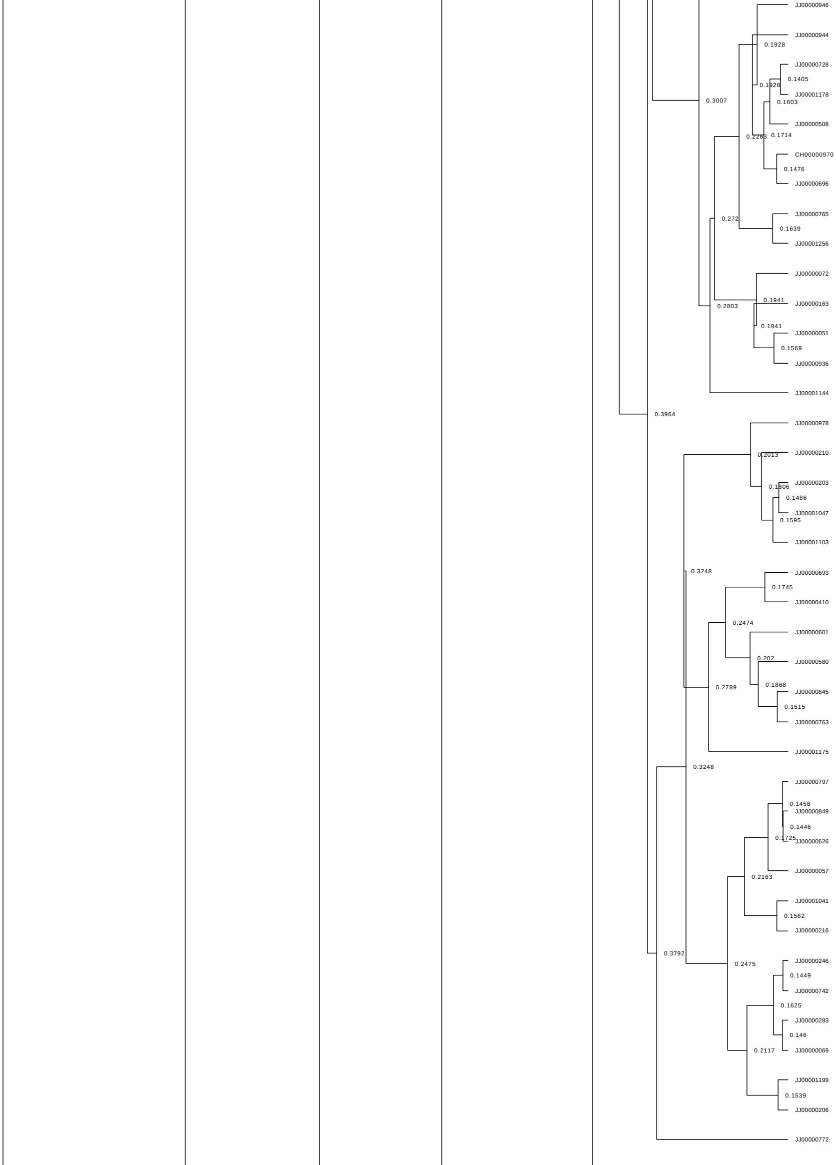
JJ00000946
JJ00000944
0.1928
JJ00000728
0.1405
0.1928
JJ00001178
0.3007
0.1603
JJ00000508
0.2263
0.1714
CH00000970
0.1476
JJ00000696
JJ00000765
0.272
0.1639
JJ00001256
JJ00000072
0.1941
JJ00000163
0.2803
0.1941
JJ00000051
0.1569
JJ00000936
JJ00001144
0.3964
JJ00000978
0.2013
JJ00000210
JJ00000203
0.1806
0.1486
JJ00001047
0.1595
JJ00001103
0.3248
JJ00000693
0.1745
JJ00000410
0.2474
JJ00000601
0.202
JJ00000580
0.1868
JJ00000845
0.2789
0.1515
JJ00000763
JJ00001175
0.3248
JJ00000797
0.1458
JJ00000849
0.1446
0.1725
JJ00000626
JJ00000057
0.2163
JJ00001041
0.1562
JJ00000216
0.3792
JJ00000246
0.2475
0.1449
JJ00000742
0.1625
JJ00000283
0.146
JJ00000069
0.2117
JJ00001199
0.1539
JJ00000206
JJ00000772

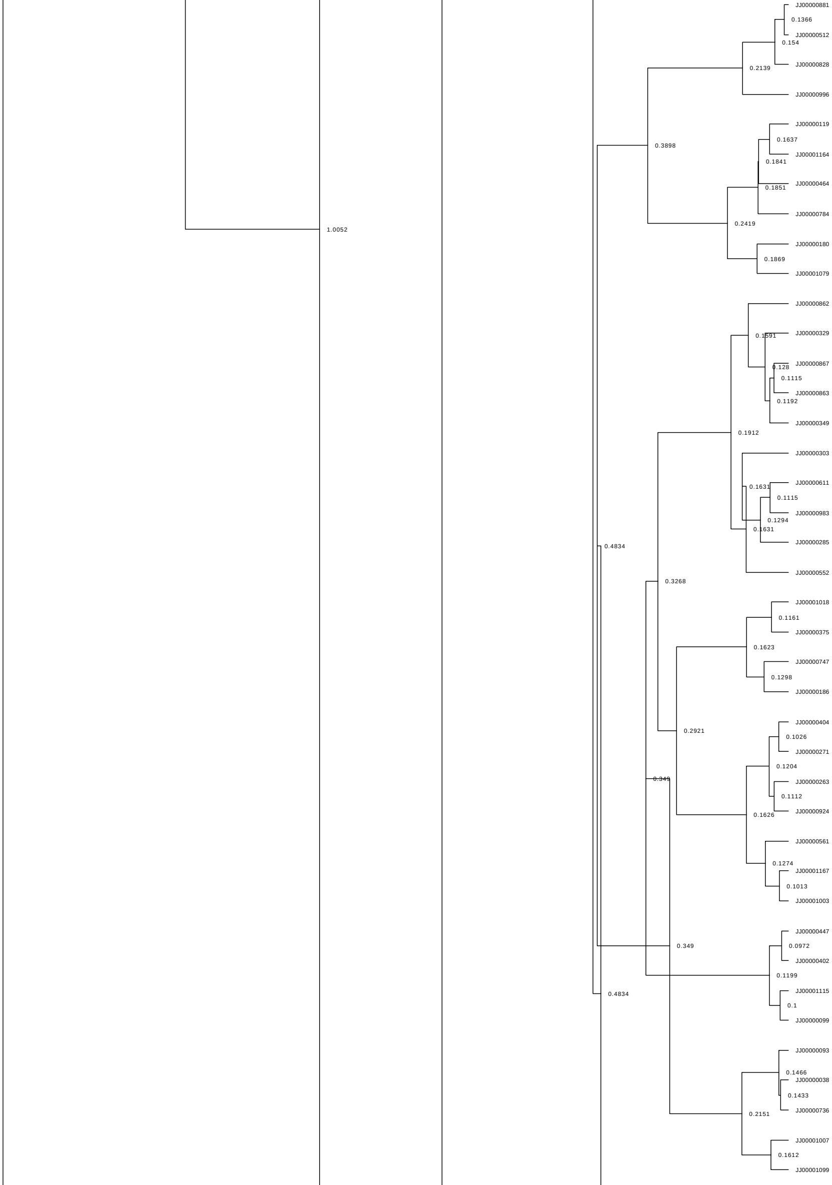
JJ00000881
0.1366
JJ00000512
0.154
JJ00000828
0.2139
JJ00000996
JJ00000119
0.1637
0.3898
JJ00001164
0.1841
JJ00000464
0.1851
JJ00000784
0.2419
1.0052
JJ00000180
0.1869
JJ00001079
JJ00000862
JJ00000329
0.1591
JJ00000867
0.128
0.1115
JJ00000863
0.1192
JJ00000349
0.1912
JJ00000303
JJ00000611
0.1631
0.1115
JJ00000983
0.1294
0.1631
JJ00000285
0.4834
JJ00000552
0.3268
JJ00001018
0.1161
JJ00000375
0.1623
JJ00000747
0.1298
JJ00000186
JJ00000404
0.2921
0.1026
JJ00000271
0.1204
0.349
JJ00000263
0.1112
JJ00000924
0.1626
JJ00000561
0.1274
JJ00001167
0.1013
JJ00001003
JJ00000447
0.0972
0.349
JJ00000402
0.1199
JJ00001115
0.4834
0.1
JJ00000099
JJ00000093
0.1466
JJ00000038
0.1433
JJ00000736
0.2151
JJ00001007
0.1612
JJ00001099

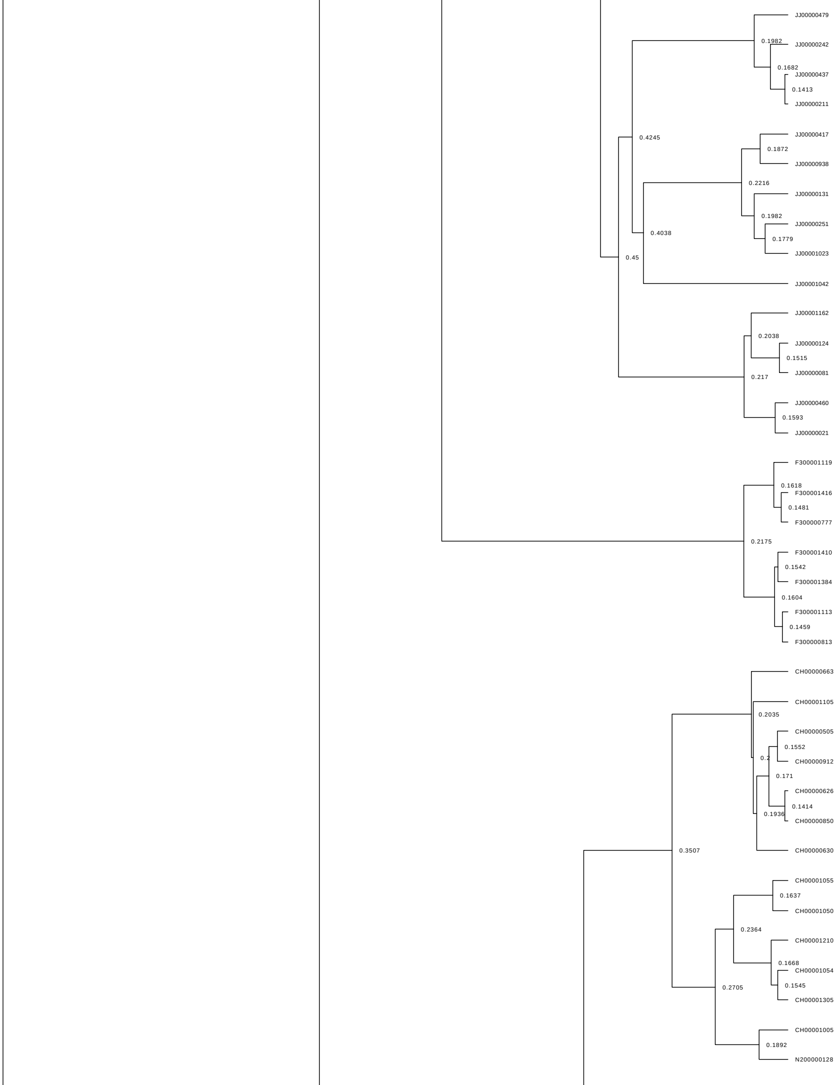
JJ00000479
0.1982
JJ00000242
0.1682
JJ00000437
0.1413
JJ00000211
JJ00000417
0.4245
0.1872
JJ00000938
0.2216
JJ00000131
0.1982
JJ00000251
0.4038
0.1779
0.45
JJ00001023
JJ00001042
JJ00001162
0.2038
JJ00000124
0.1515
JJ00000081
0.217
JJ00000460
0.1593
JJ00000021
F300001119
0.1618
F300001416
0.1481
F300000777
0.2175
F300001410
0.1542
F300001384
0.1604
F300001113
0.1459
F300000813
CH00000663
CH00001105
0.2035
CH00000505
0.1552
CH00000912
0.171
CH00000626
0.1414
0.1936
CH00000850
0.3507
CH00000630
CH00001055
0.1637
CH00001050
0.2364
CH00001210
0.1668
CH00001054
0.1545
0.2705
CH00001305
CH00001005
0.1892
N200000128

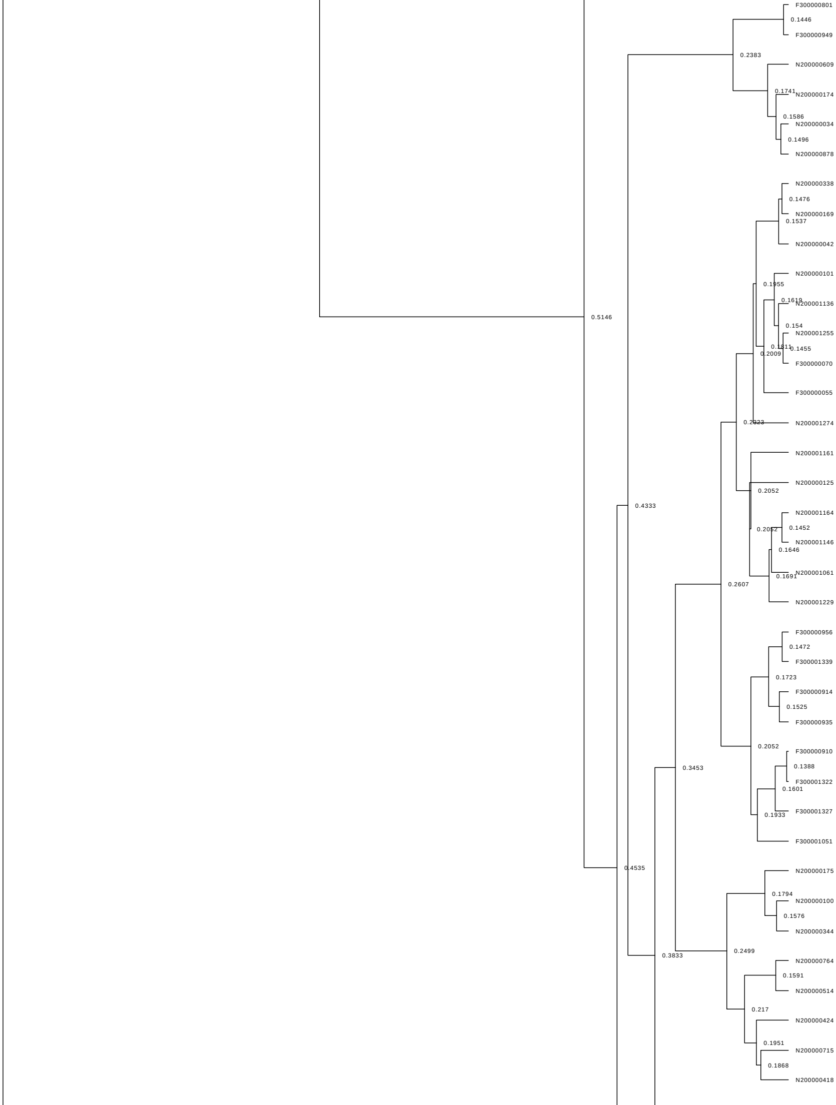
F300000801
0.1446
F300000949
0.2383
N200000609
0.1741
N200000174
0.1586
N200000034
0.1496
N200000878
N200000338
0.1476
N200000169
0.1537
N200000042
N200000101
0.1955
0.1619
N200001136
0.154
N200001255
0.1811
0.1455
0.2009
F300000070
0.5146
F300000055
0.2323
N200001274
N200001161
N200000125
0.2052
0.4333
N200001164
0.1452
0.2052
N200001146
0.1646
N200001061
0.1691
0.2607
N200001229
F300000956
0.1472
F300001339
0.1723
F300000914
0.1525
F300000935
0.2052
F300000910
0.1388
0.3453
F300001322
0.1601
F300001327
0.1933
F300001051
0.4535
N200000175
0.1794
N200000100
0.1576
N200000344
0.2499
0.3833
N200000764
0.1591
N200000514
0.217
N200000424
0.1951
N200000715
0.1868
N200000418

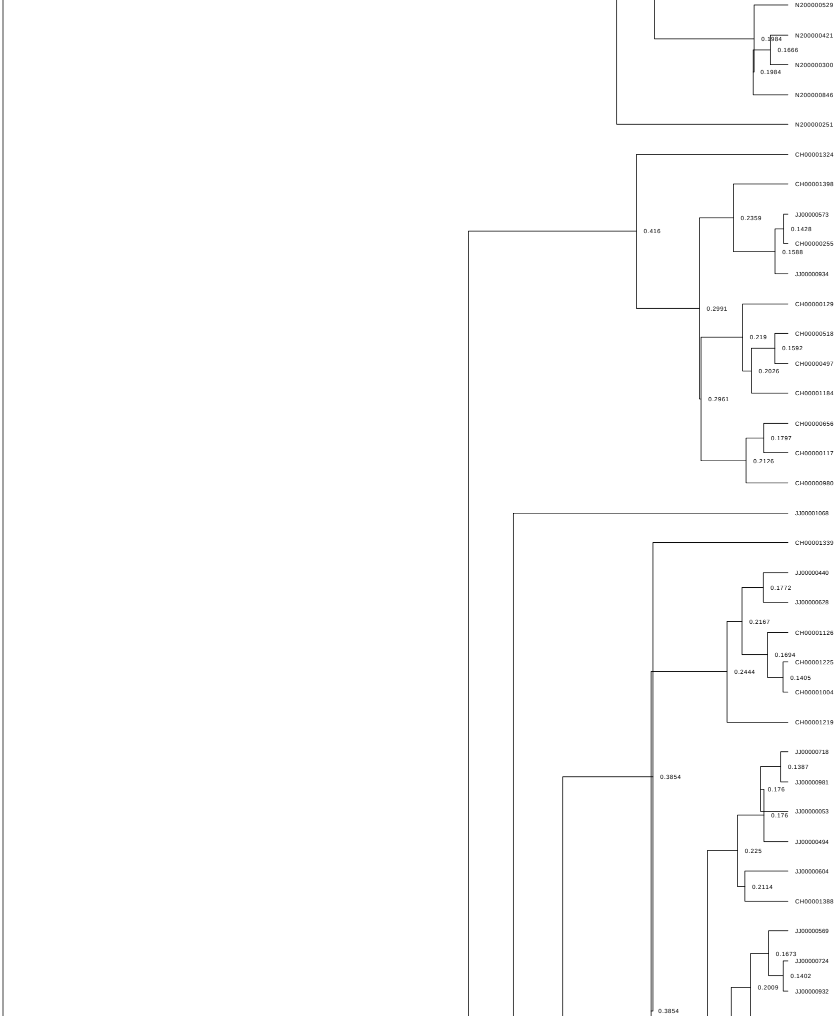
N200000529
0.1984
N200000421
0.1666
N200000300
0.1984
N200000846
N200000251
CH00001324
CH00001398
0.416
0.2359
JJ00000573
0.1428
CH00000255
0.1588
JJ00000934
CH00000129
0.2991
CH00000518
0.219
0.1592
CH00000497
0.2026
CH00001184
0.2961
CH00000656
0.1797
CH00000117
0.2126
CH00000980
JJ00001068
CH00001339
JJ00000440
0.1772
JJ00000628
0.2167
CH00001126
0.1694
CH00001225
0.1405
CH00001004
0.2444
CH00001219
JJ00000718
0.1387
JJ00000981
0.3854
0.176
JJ00000053
0.176
JJ00000494
0.225
JJ00000604
0.2114
CH00001388
JJ00000569
0.1673
JJ00000724
0.1402
JJ00000932
0.2009
0.3854

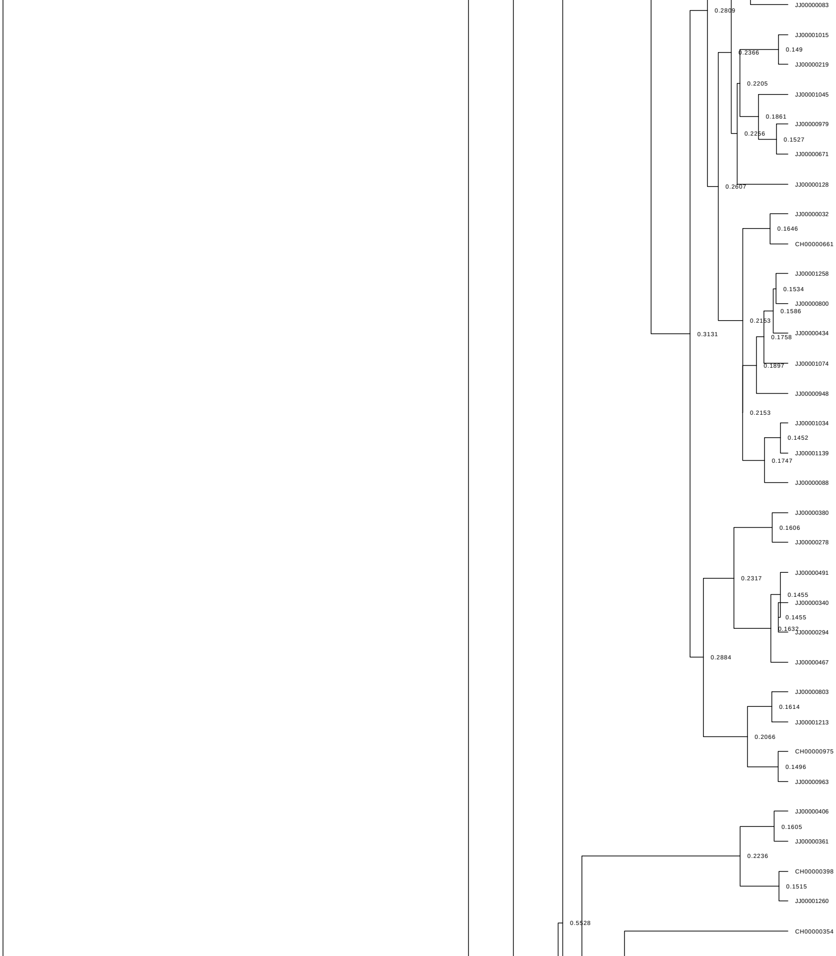
JJ00000083
0.2809
JJ00001015
0.149
0.2366
JJ00000219
0.2205
JJ00001045
0.1861
JJ00000979
0.2256
0.1527
JJ00000671
JJ00000128
0.2607
JJ00000032
0.1646
CH00000661
JJ00001258
0.1534
JJ00000800
0.1586
0.2153
JJ00000434
0.1758
0.3131
JJ00001074
0.1897
JJ00000948
0.2153
JJ00001034
0.1452
JJ00001139
0.1747
JJ00000088
JJ00000380
0.1606
JJ00000278
0.2317
JJ00000491
0.1455
JJ00000340
0.1455
0.1632
JJ00000294
0.2884
JJ00000467
JJ00000803
0.1614
JJ00001213
0.2066
CH00000975
0.1496
JJ00000963
JJ00000406
0.1605
JJ00000361
0.2236
CH00000398
0.1515
JJ00001260
0.5528
CH00000354

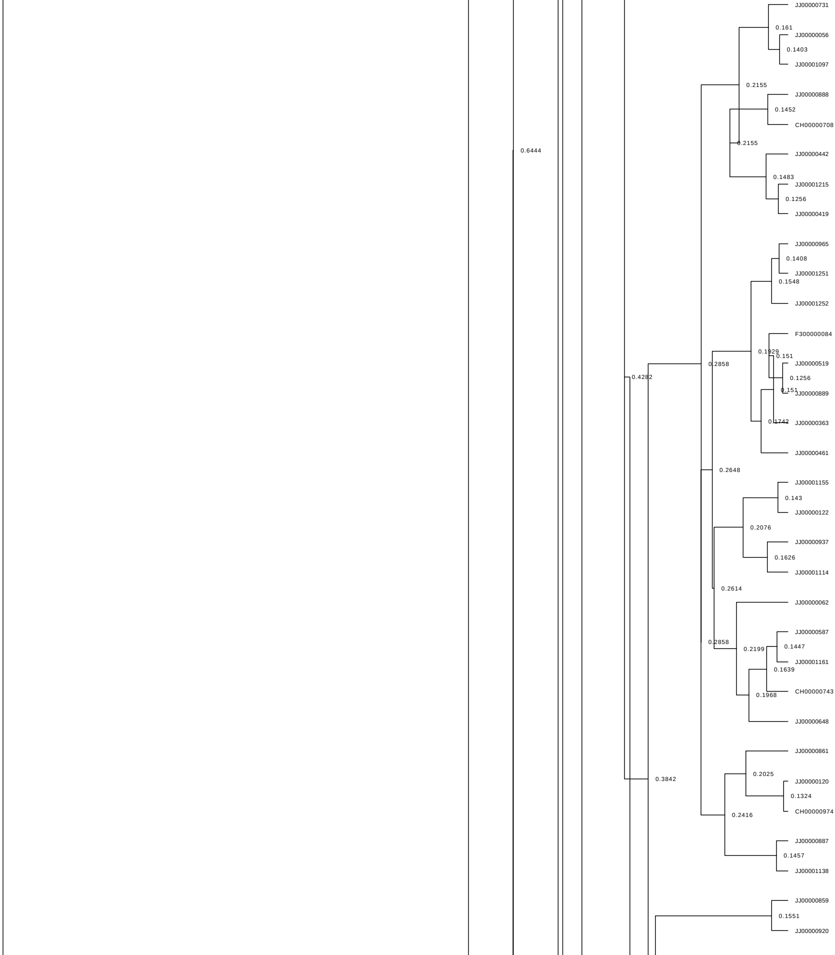
JJ00000731
0.161
JJ00000056
0.1403
JJ00001097
0.2155
JJ00000888
0.1452
CH00000708
0.6444
0.2155
JJ00000442
0.1483
JJ00001215
0.1256
JJ00000419
JJ00000965
0.1408
JJ00001251
0.1548
JJ00001252
F300000084
0.1929
0.151
JJ00000519
0.2858
0.4282
0.1256
0.151
JJ00000889
0.1742
JJ00000363
JJ00000461
0.2648
JJ00001155
0.143
JJ00000122
0.2076
JJ00000937
0.1626
JJ00001114
0.2614
JJ00000062
JJ00000587
0.1447
0.2858
0.2199
JJ00001161
0.1639
CH00000743
0.1968
JJ00000648
JJ00000861
0.2025
JJ00000120
0.3842
0.1324
CH00000974
0.2416
JJ00000887
0.1457
JJ00001138
JJ00000859
0.1551
JJ00000920

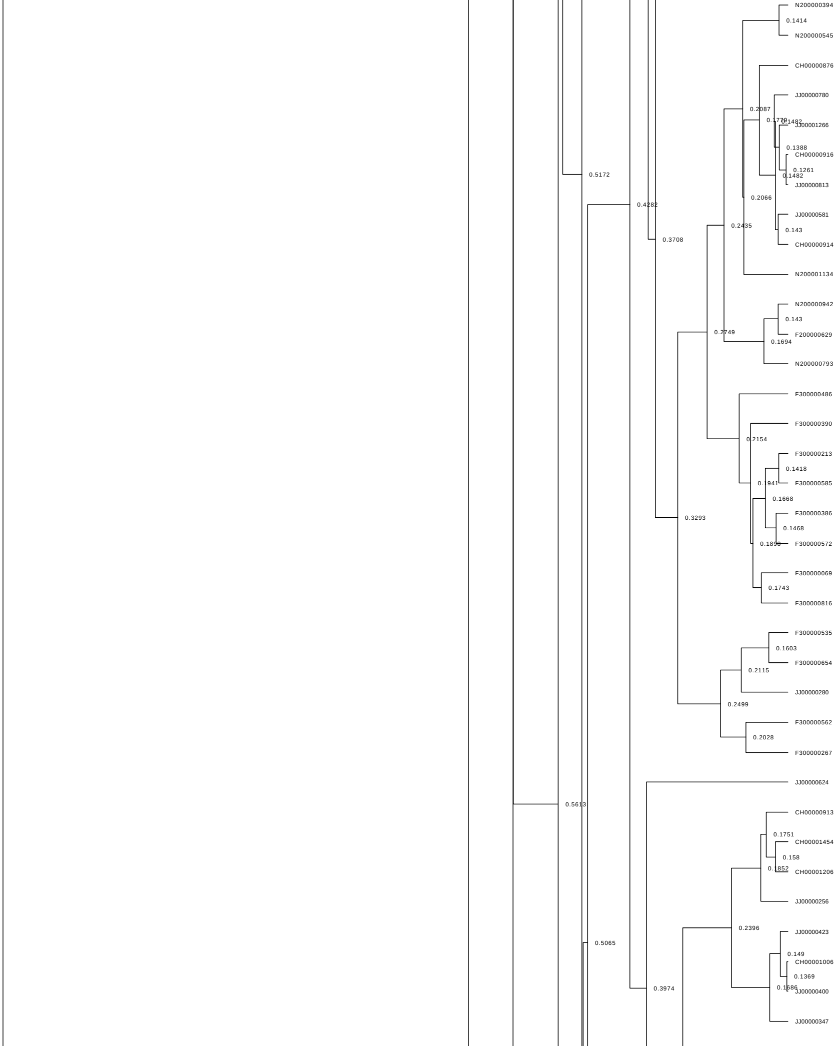

N200000394
0.1414
N200000545
CH00000876
JJ00000780
0.2087
0.1779
0.1482
JJ00001266
0.1388
CH00000916
0.1261
0.1482
JJ00000813
0.5172
0.4282
0.2066
JJ00000581
0.2435
0.143
0.3708
CH00000914
N200001134
N200000942
0.143
0.2749
F200000629
0.1694
N200000793
F300000486
F300000390
0.2154
F300000213
0.1418
0.1941
F300000585
0.1668
F300000386
0.3293
0.1468
0.1898
F300000572
F300000069
0.1743
F300000816
F300000535
0.1603
F300000654
0.2115
JJ00000280
0.2499
F300000562
0.2028
F300000267
JJ00000624
0.5613
CH00000913
0.1751
CH00001454
0.158
0.1852
CH00001206
JJ00000256
0.2396
0.5065
JJ00000423
0.149
CH00001006
0.1369
0.3974
0.1686
JJ00000400
JJ00000347

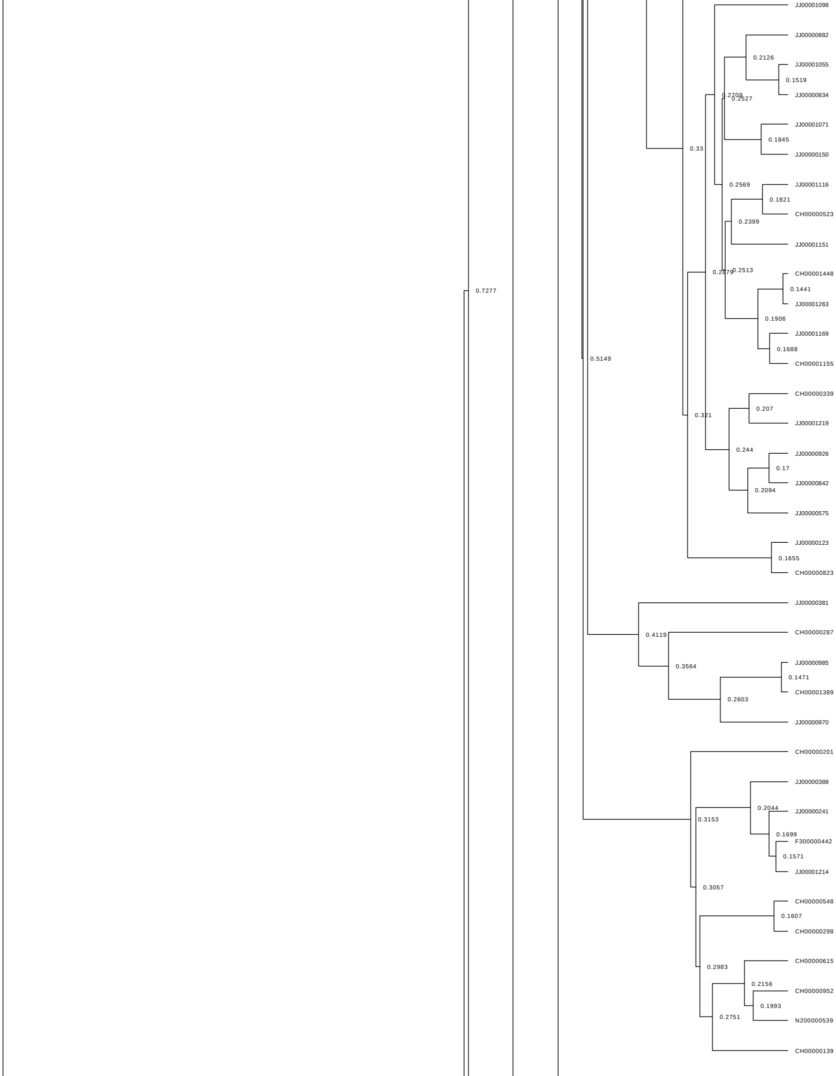
JJ00001098
JJ00000882
0.2126
JJ00001055
0.1519
JJ00000834
0.2709
0.2527
JJ00001071
0.1845
JJ00000150
0.33
0.2569
JJ00001116
0.1821
CH00000523
0.2399
JJ00001151
0.2879
0.2513
CH00001448
0.1441
JJ00001263
0.7277
0.1906
JJ00001169
0.1688
0.5149
CH00001155
CH00000339
0.207
0.321
JJ00001219
0.244
JJ00000926
0.17
JJ00000842
0.2094
JJ00000575
JJ00000123
0.1655
CH00000823
JJ00000381
CH00000287
0.4119
JJ00000985
0.3564
0.1471
CH00001389
0.2603
JJ00000970
CH00000201
JJ00000388
0.2044
JJ00000241
0.3153
0.1699
F300000442
0.1571
JJ00001214
0.3057
CH00000548
0.1607
CH00000298
CH00000615
0.2983
0.2156
CH00000952
0.1993
N200000539
0.2751
CH00000139

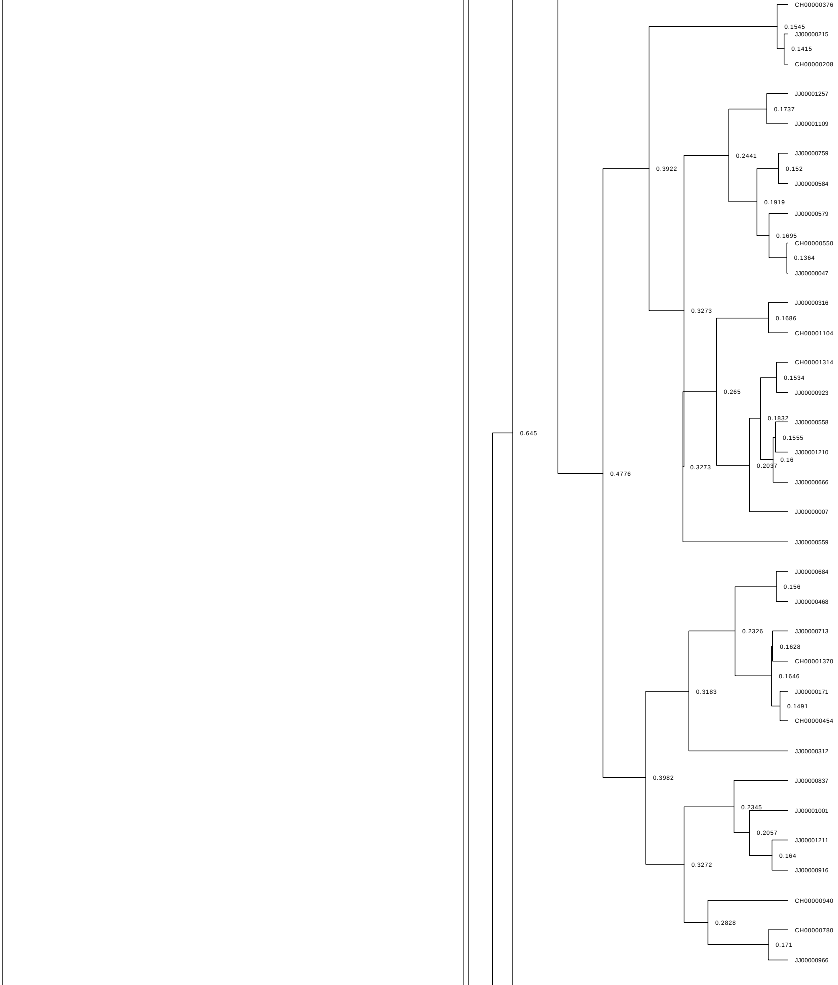

CH00000376
0.1545
JJ00000215
0.1415
CH00000208
JJ00001257
0.1737
JJ00001109
0.2441
JJ00000759
0.152
JJ00000584
0.3922
0.1919
JJ00000579
0.1695
CH00000550
0.1364
JJ00000047
JJ00000316
0.3273
0.1686
CH00001104
CH00001314
0.1534
JJ00000923
0.265
0.1832
JJ00000558
0.645
0.1555
JJ00001210
0.16
0.3273
0.2037
0.4776
JJ00000666
JJ00000007
JJ00000559
JJ00000684
0.156
JJ00000468
0.2326
JJ00000713
0.1628
CH00001370
0.1646
0.3183
JJ00000171
0.1491
CH00000454
JJ00000312
0.3982
JJ00000837
0.2345
JJ00001001
0.2057
JJ00001211
0.164
0.3272
JJ00000916
CH00000940
0.2828
CH00000780
0.171
JJ00000966

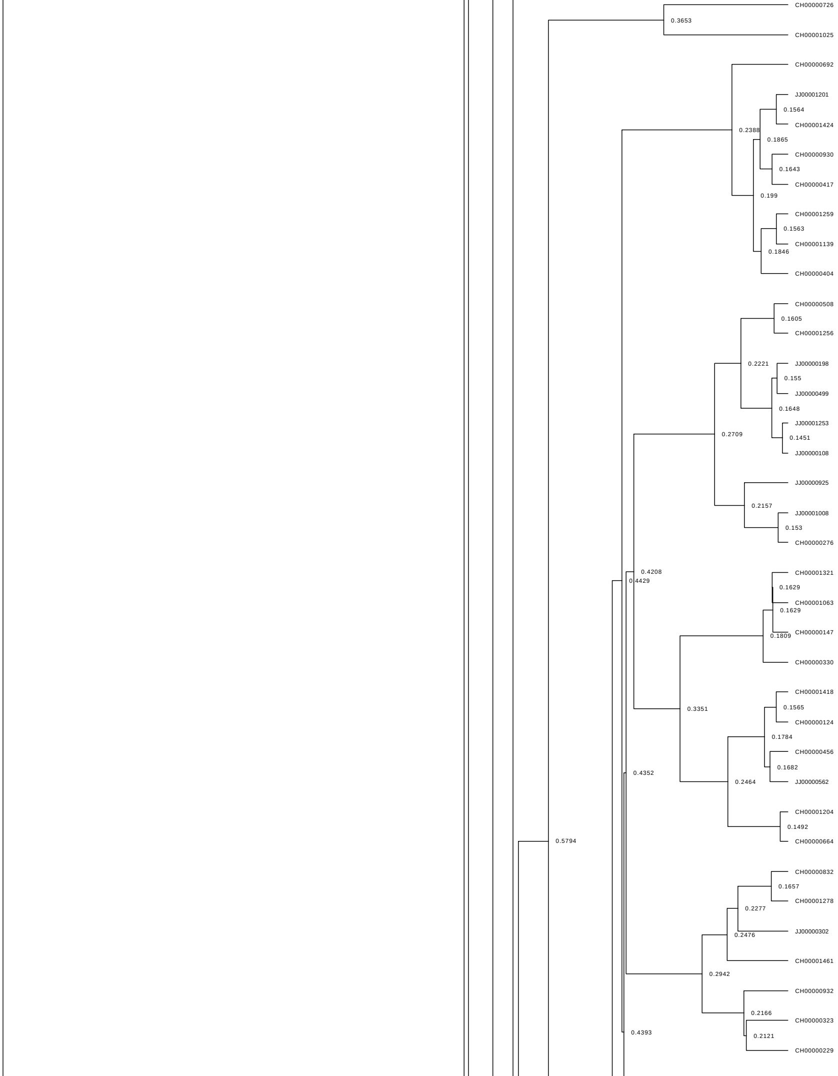
CH00000726
0.3653
CH00001025
CH00000692
JJ00001201
0.1564
CH00001424
0.2388
0.1865
CH00000930
0.1643
CH00000417
0.199
CH00001259
0.1563
CH00001139
0.1846
CH00000404
CH00000508
0.1605
CH00001256
0.2221
JJ00000198
0.155
JJ00000499
0.1648
JJ00001253
0.1451
0.2709
JJ00000108
JJ00000925
0.2157
JJ00001008
0.153
CH00000276
0.4208
0.4429
CH00001321
0.1629
CH00001063
0.1629
CH00000147
0.1809
CH00000330
CH00001418
0.1565
CH00000124
0.3351
0.1784
CH00000456
0.1682
0.4352
JJ00000562
0.2464
CH00001204
0.1492
CH00000664
0.5794
CH00000832
0.1657
CH00001278
0.2277
JJ00000302
0.2476
CH00001461
0.2942
CH00000932
0.2166
CH00000323
0.2121
0.4393
CH00000229

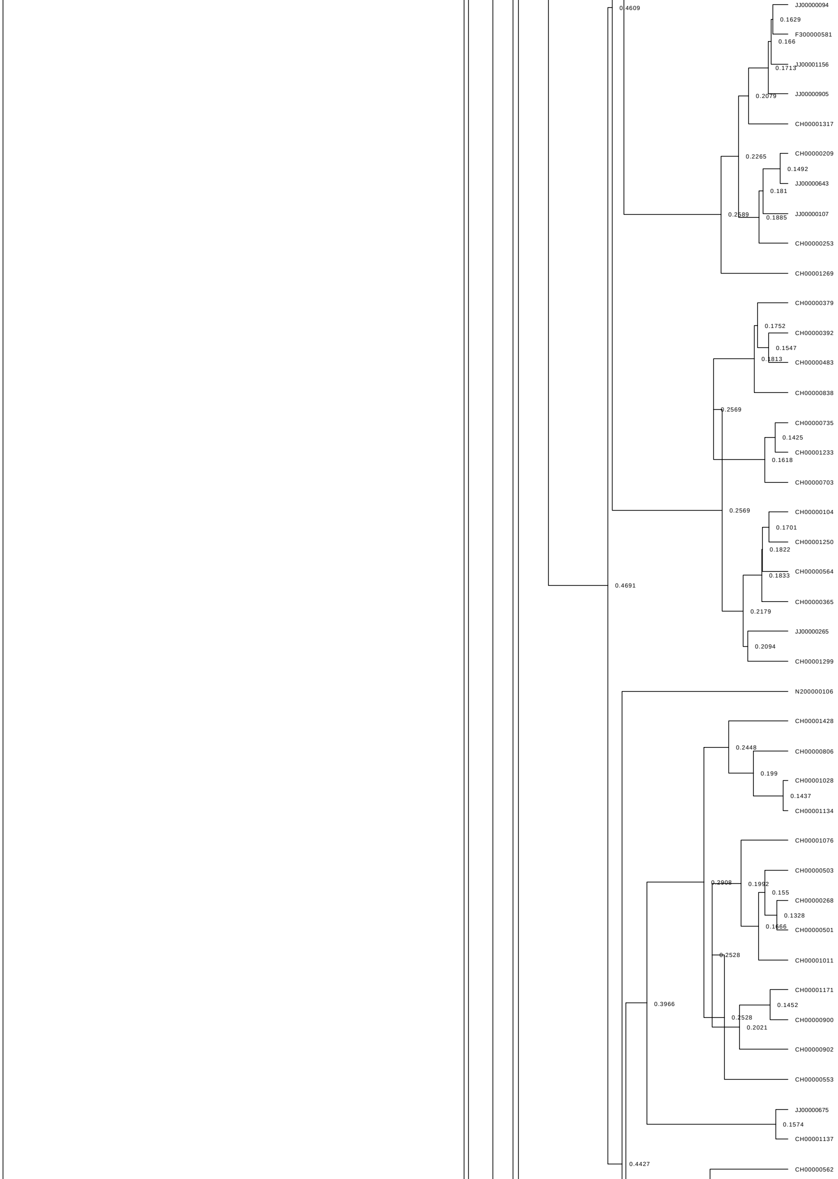

JJ00000094
0.4609
0.1629
F300000581
0.166
JJ00001156
0.1713
JJ00000905
0.2079
CH00001317
0.2265
CH00000209
0.1492
JJ00000643
0.181
JJ00000107
0.2589
0.1885
CH00000253
CH00001269
CH00000379
0.1752
CH00000392
0.1547
0.1813
CH00000483
CH00000838
0.2569
CH00000735
0.1425
CH00001233
0.1618
CH00000703
0.2569
CH00000104
0.1701
CH00001250
0.1822
CH00000564
0.1833
0.4691
CH00000365
0.2179
JJ00000265
0.2094
CH00001299
N200000106
CH00001428
0.2448
CH00000806
0.199
CH00001028
0.1437
CH00001134
CH00001076
CH00000503
0.2908
0.1992
0.155
CH00000268
0.1328
0.1666
CH00000501
0.2528
CH00001011
CH00001171
0.3966
0.1452
0.2528
CH00000900
0.2021
CH00000902
CH00000553
JJ00000675
0.1574
CH00001137
0.4427
CH00000562

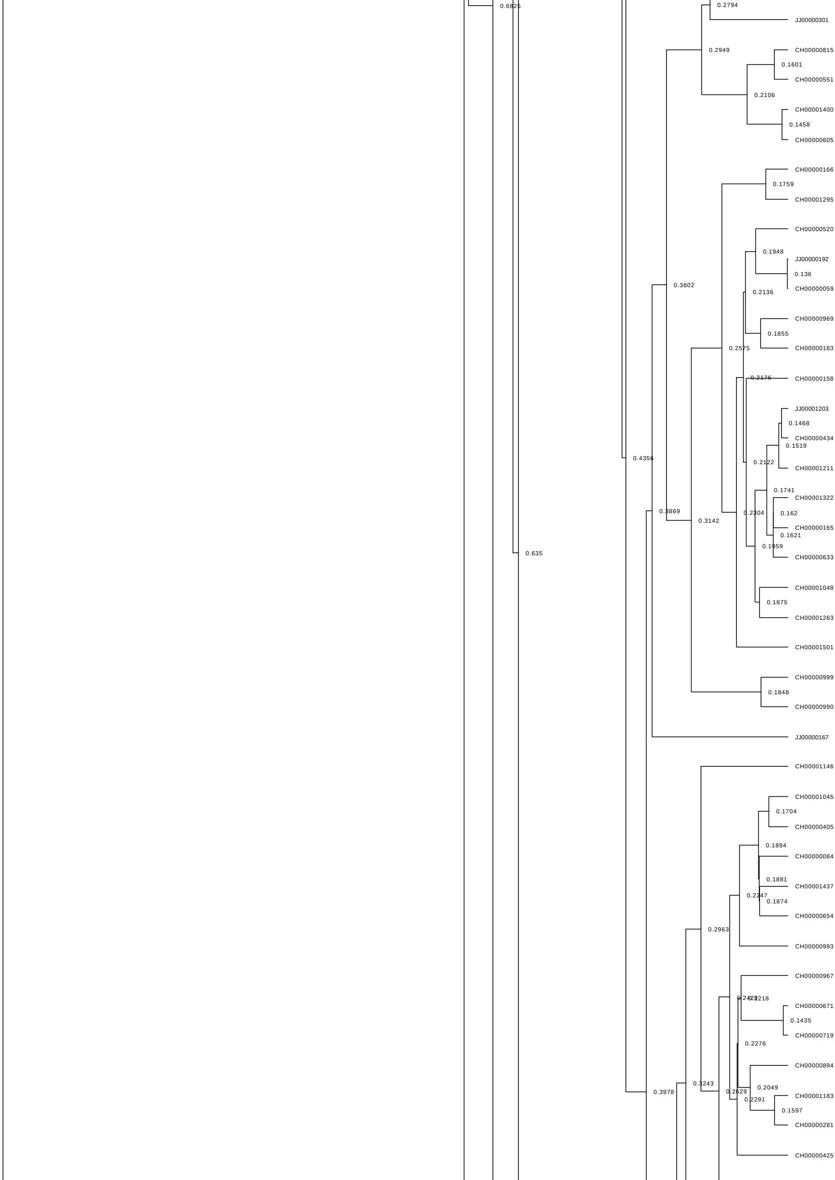

0.6825
0.2794
JJ00000301
0.2949
CH00000815
0.1601
CH00000551
0.2106
CH00001400
0.1458
CH00000605
CH00000166
0.1759
CH00001295
CH00000520
0.1948
JJ00000192
0.136
0.3602
CH00000059
0.2136
CH00000969
0.1855
CH00000183
0.2575
0.2176
CH00000158
JJ00001203
0.1468
CH00000434
0.1519
0.4356
0.2122
CH00001211
0.1741
CH00001322
0.3869
0.2304
0.162
0.3142
CH00000165
0.1621
0.635
0.1959
CH00000633
CH00001048
0.1875
CH00001263
CH00001501
CH00000999
0.1848
CH00000990
JJ00000167
CH00001146
CH00001045
0.1704
CH00000405
0.1894
CH00000084
0.1881
CH00001437
0.2247
0.1874
CH00000654
0.2963
CH00000993
CH00000967
0.2409
0.2218
CH00000671
0.1435
CH00000719
0.2276
CH00000894
0.2049
0.3243
0.3978
0.2629
0.2291
CH00001183
0.1597
CH00000281
CH00000425

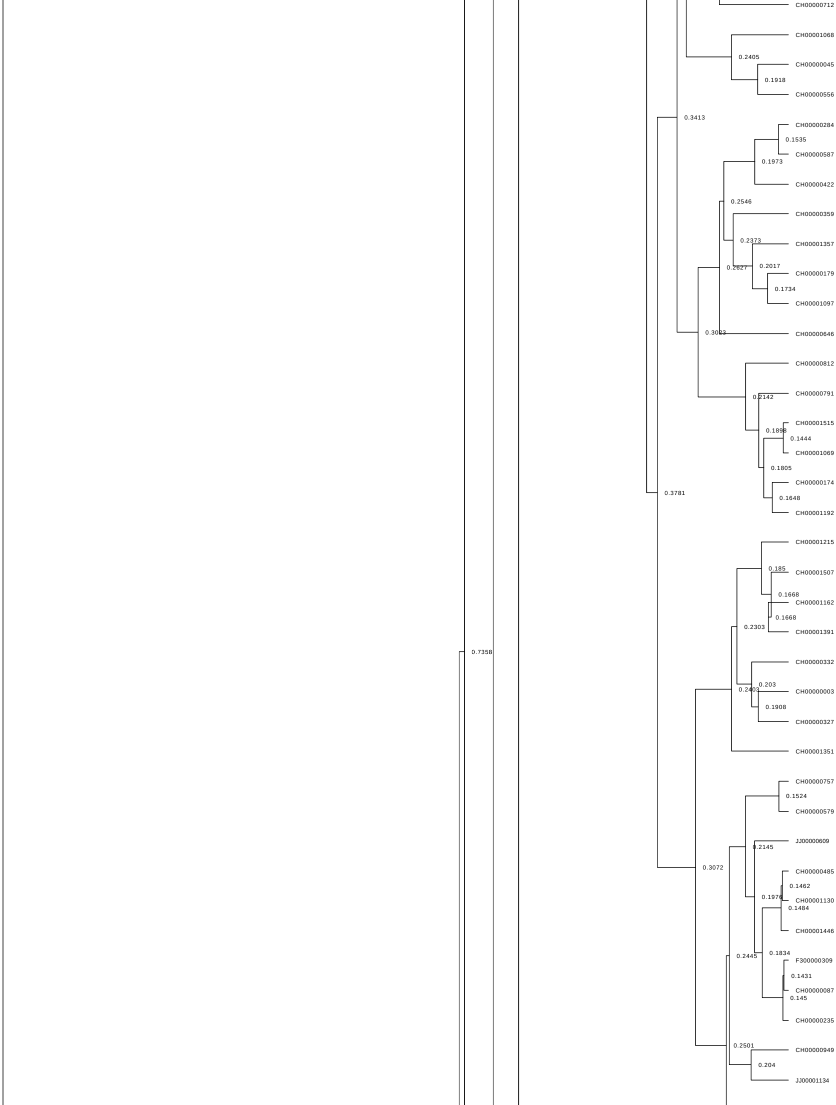
CH00000712
CH00001068
0.2405
CH00000045
0.1918
CH00000556
0.3413
CH00000284
0.1535
CH00000587
0.1973
CH00000422
0.2546
CH00000359
0.2373
CH00001357
0.2627
0.2017
CH00000179
0.1734
CH00001097
0.3023
CH00000646
CH00000812
CH00000791
0.2142
CH00001515
0.1898
0.1444
CH00001069
0.1805
CH00000174
0.3781
0.1648
CH00001192
CH00001215
0.185
CH00001507
0.1668
CH00001162
0.1668
0.2303
CH00001391
0.7358
CH00000332
0.203
0.2403
CH00000003
0.1908
CH00000327
CH00001351
CH00000757
0.1524
CH00000579
JJ00000609
0.2145
0.3072
CH00000485
0.1462
0.1976
CH00001130
0.1484
CH00001446
0.1834
0.2445
F300000309
0.1431
CH00000087
0.145
CH00000235
0.2501
CH00000949
0.204
JJ00001134

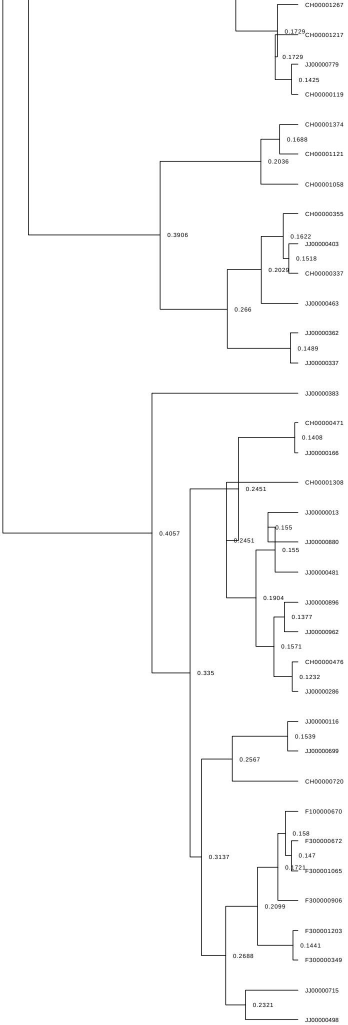
CH00001267
0.1729
CH00001217
0.1729
JJ00000779
0.1425
CH00000119
CH00001374
0.1688
CH00001121
0.2036
CH00001058
CH00000355
0.1622
JJ00000403
0.1518
0.3906
0.2029
CH00000337
JJ00000463
0.266
JJ00000362
0.1489
JJ00000337
JJ00000383
CH00000471
0.1408
JJ00000166
CH00001308
0.2451
JJ00000013
0.155
0.4057
0.2451
JJ00000880
0.155
JJ00000481
0.1904
JJ00000896
0.1377
JJ00000962
0.1571
CH00000476
0.335
0.1232
JJ00000286
JJ00000116
0.1539
JJ00000699
0.2567
CH00000720
F100000670
0.158
F300000672
0.147
0.3137
0.1721
F300001065
F300000906
0.2099
F300001203
0.1441
F300000349
0.2688
JJ00000715
0.2321
JJ00000498

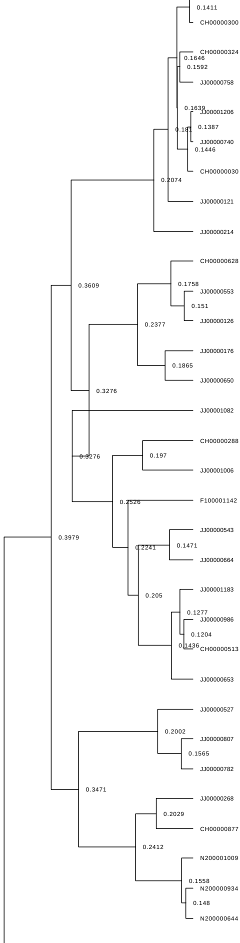
JJ00000102
0.1411
CH00000300
CH00000324
0.1646
0.1592
JJ00000758
0.1639
JJ00001206
0.181
0.1387
JJ00000740
0.1446
CH00000030
0.2074
JJ00000121
JJ00000214
CH00000628
0.3609
0.1758
JJ00000553
0.151
JJ00000126
0.2377
JJ00000176
0.1865
JJ00000650
0.3276
JJ00001082
CH00000288
0.3276
0.197
JJ00001006
F100001142
0.2526
JJ00000543
0.3979
0.2241
0.1471
JJ00000664
JJ00001183
0.205
0.1277
JJ00000986
0.1204
0.1436
CH00000513
JJ00000653
JJ00000527
0.2002
JJ00000807
0.1565
JJ00000782
0.3471
JJ00000268
0.2029
CH00000877
0.2412
N200001009
0.1558
N200000934
0.148
N200000644

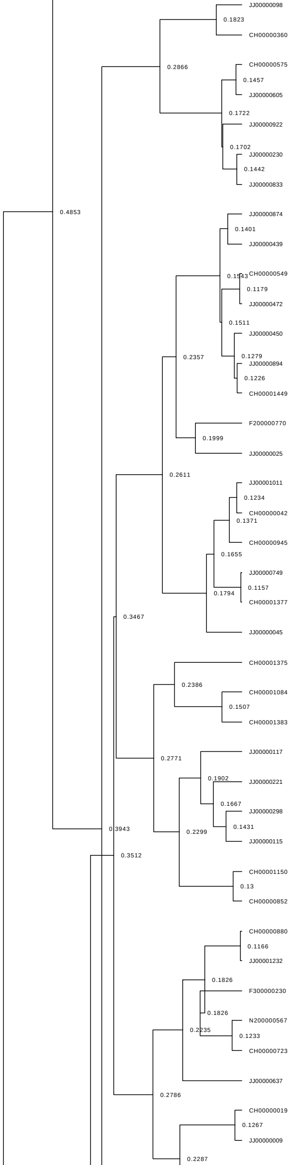
JJ00000098
0.1823
CH00000360
0.2866
CH00000575
0.1457
JJ00000605
0.1722
JJ00000922
0.1702
JJ00000230
0.1442
JJ00000833
0.4853
JJ00000874
0.1401
JJ00000439
0.1543
CH00000549
0.1179
JJ00000472
0.1511
JJ00000450
0.1279
JJ00000894
0.1226
CH00001449
0.2357
F200000770
0.1999
JJ00000025
0.2611
JJ00001011
0.1234
CH00000042
0.1371
CH00000945
0.1655
JJ00000749
0.1157
CH00001377
0.1794
0.3467
JJ00000045
CH00001375
0.2386
CH00001084
0.1507
CH00001383
0.2771
JJ00000117
0.1902
JJ00000221
0.1667
JJ00000298
0.1431
JJ00000115
0.3943
0.2299
0.3512
CH00001150
0.13
CH00000852
CH00000880
0.1166
JJ00001232
0.1826
F300000230
0.1826
N200000567
0.2235
0.1233
CH00000723
JJ00000637
0.2786
CH00000019
0.1267
JJ00000009
0.2287

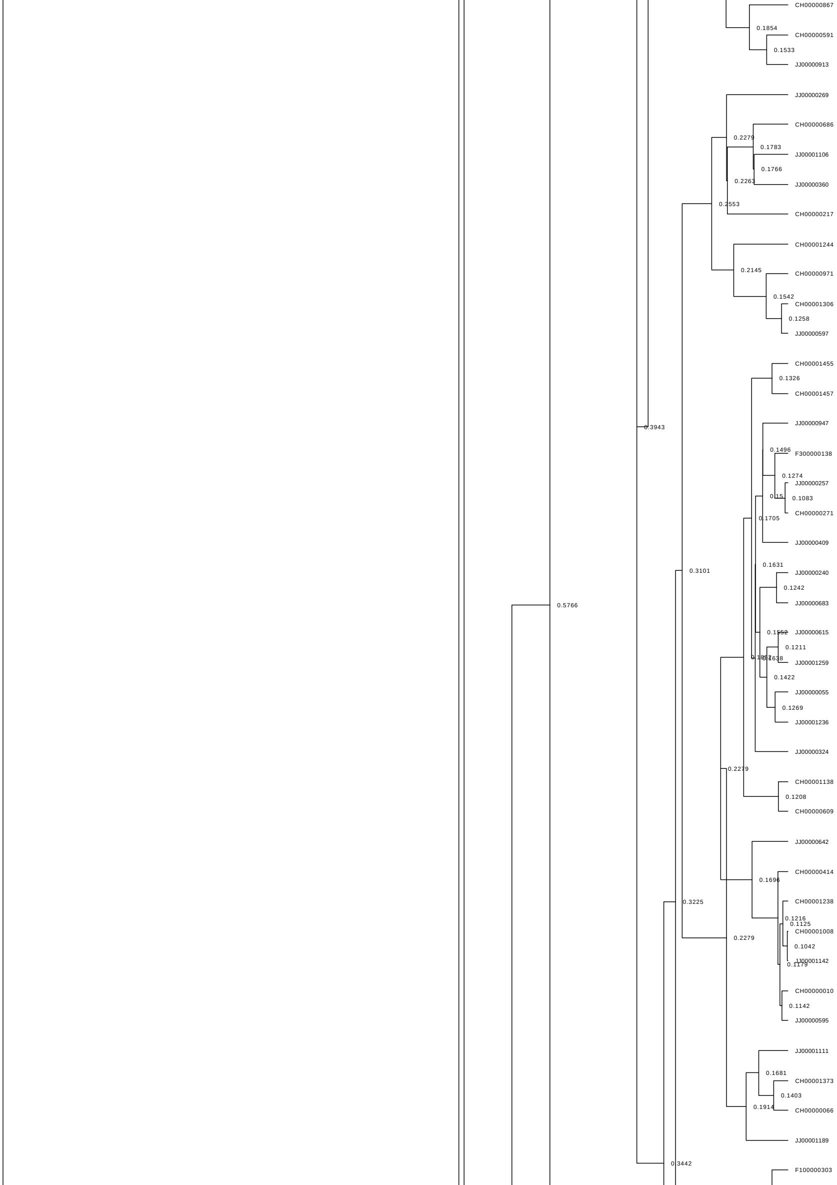

CH00000867
0.1854
CH00000591
0.1533
JJ00000913
JJ00000269
CH00000686
0.2279
0.1783
JJ00001106
0.1766
0.2263
JJ00000360
0.2553
CH00000217
CH00001244
0.2145
CH00000971
0.1542
CH00001306
0.1258
JJ00000597
CH00001455
0.1326
CH00001457
JJ00000947
0.3943
0.1496
F300000138
0.1274
JJ00000257
0.15
0.1083
CH00000271
0.1705
JJ00000409
0.3101
0.1631
JJ00000240
0.1242
JJ00000683
0.5766
JJ00000615
0.1211
JJ00001259
0.1422
JJ00000055
0.1269
JJ00001236
JJ00000324
0.2279
CH00001138
0.1208
CH00000609
JJ00000642
CH00000414
0.1696
CH00001238
0.3225
0.1216
0.1125
CH00001008
0.2279
0.1042
JJ00001142
0.1179
CH00000010
0.1142
JJ00000595
JJ00001111
0.1681
CH00001373
0.1403
0.1914
CH00000066
JJ00001189
0.3442
F100000303

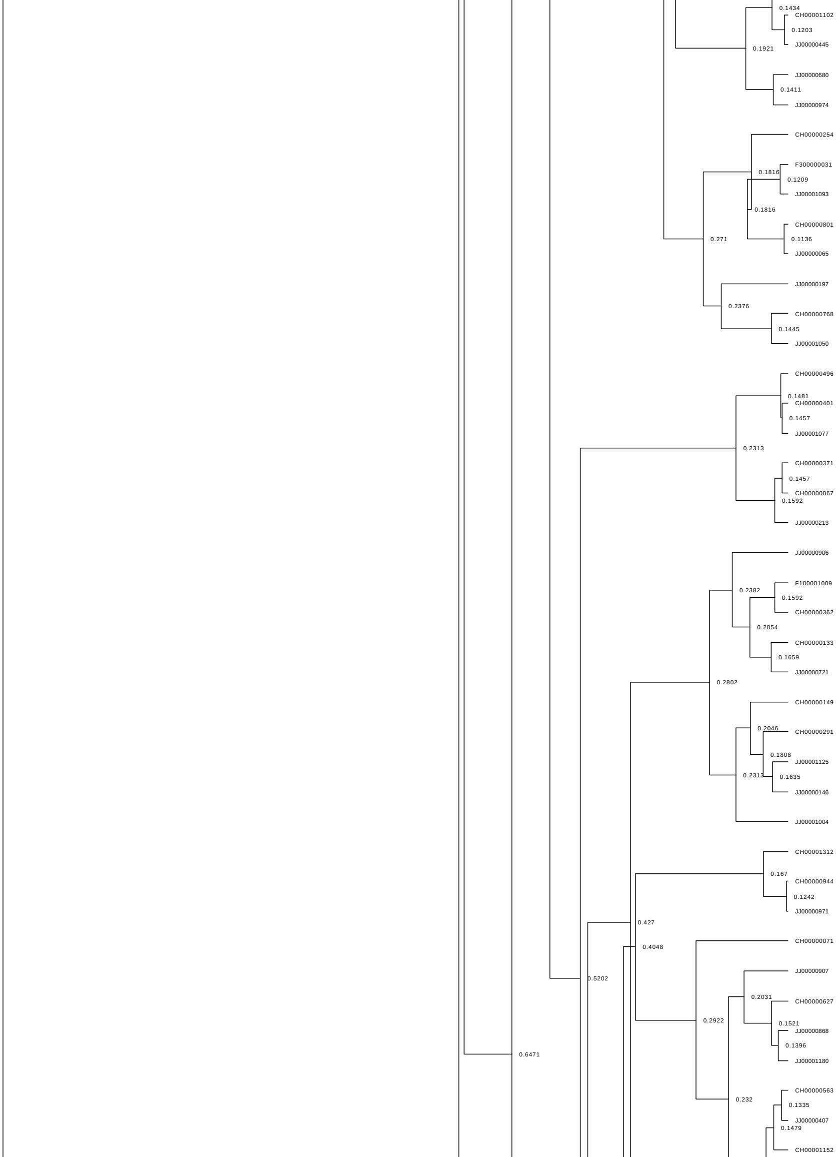
0.1434
CH00001102
0.1203
JJ00000445
0.1921
JJ00000680
0.1411
JJ00000974
CH00000254
F300000031
0.1816
0.1209
JJ00001093
0.1816
CH00000801
0.1136
0.271
JJ00000065
JJ00000197
0.2376
CH00000768
0.1445
JJ00001050
CH00000496
0.1481
CH00000401
0.1457
JJ00001077
0.2313
CH00000371
0.1457
CH00000067
0.1592
JJ00000213
JJ00000906
F100001009
0.2382
0.1592
CH00000362
0.2054
CH00000133
0.1659
JJ00000721
0.2802
CH00000149
0.2046
CH00000291
0.1808
JJ00001125
0.2313
0.1635
JJ00000146
JJ00001004
CH00001312
0.167
CH00000944
0.1242
JJ00000971
0.427
CH00000071
0.4048
JJ00000907
0.5202
0.2031
CH00000627
0.2922
0.1521
JJ00000868
0.1396
0.6471
JJ00001180
CH00000563
0.232
0.1335
JJ00000407
0.1479
CH00001152

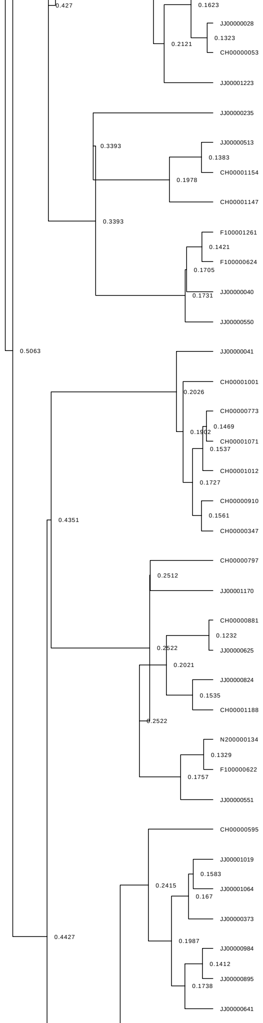
0.427
0.1623
JJ00000028
0.1323
CH00000053
0.2121
JJ00001223
JJ00000235
0.3393
JJ00000513
0.1383
CH00001154
0.1978
CH00001147
0.3393
F100001261
0.1421
F100000624
0.1705
JJ00000040
0.1731
JJ00000550
0.5063
JJ00000041
CH00001001
0.2026
CH00000773
0.1469
0.1902
CH00001071
0.1537
CH00001012
0.1727
CH00000910
0.1561
CH00000347
0.4351
CH00000797
0.2512
JJ00001170
CH00000881
0.1232
0.2522
JJ00000625
0.2021
JJ00000824
0.1535
CH00001188
0.2522
N200000134
0.1329
F100000622
0.1757
JJ00000551
CH00000595
JJ00001019
0.1583
0.2415
JJ00001064
0.167
JJ00000373
0.4427
0.1987
JJ00000984
0.1412
JJ00000895
0.1738
JJ00000641

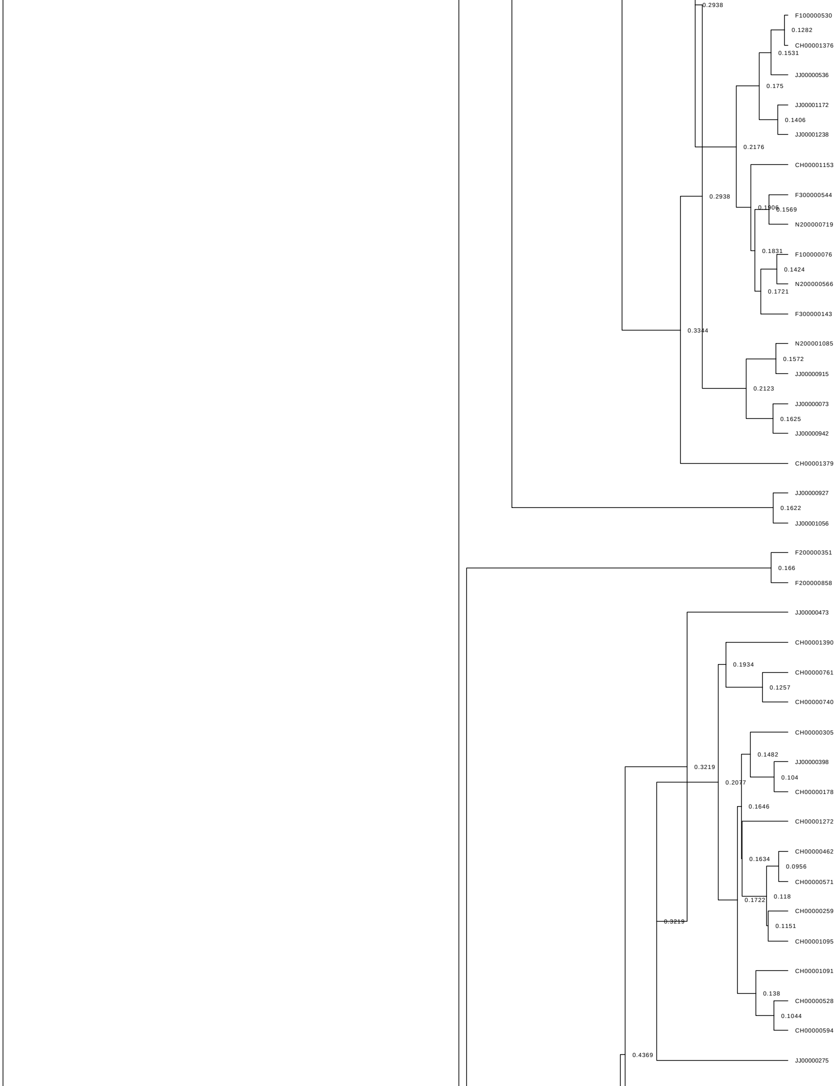

0.2938
F100000530
0.1282
CH00001376
0.1531
JJ00000536
0.175
JJ00001172
0.1406
JJ00001238
0.2176
CH00001153
F300000544
0.2938
0.1906
0.1569
N200000719
0.1831
F100000076
0.1424
N200000566
0.1721
F300000143
0.3344
N200001085
0.1572
JJ00000915
0.2123
JJ00000073
0.1625
JJ00000942
CH00001379
JJ00000927
0.1622
JJ00001056
F200000351
0.166
F200000858
JJ00000473
CH00001390
0.1934
CH00000761
0.1257
CH00000740
CH00000305
0.1482
JJ00000398
0.3219
0.104
0.2077
CH00000178
0.1646
CH00001272
CH00000462
0.1634
0.0956
CH00000571
0.118
0.1722
CH00000259
0.1151
0.3219
CH00001095
CH00001091
0.138
CH00000528
0.1044
CH00000594
0.4369
JJ00000275

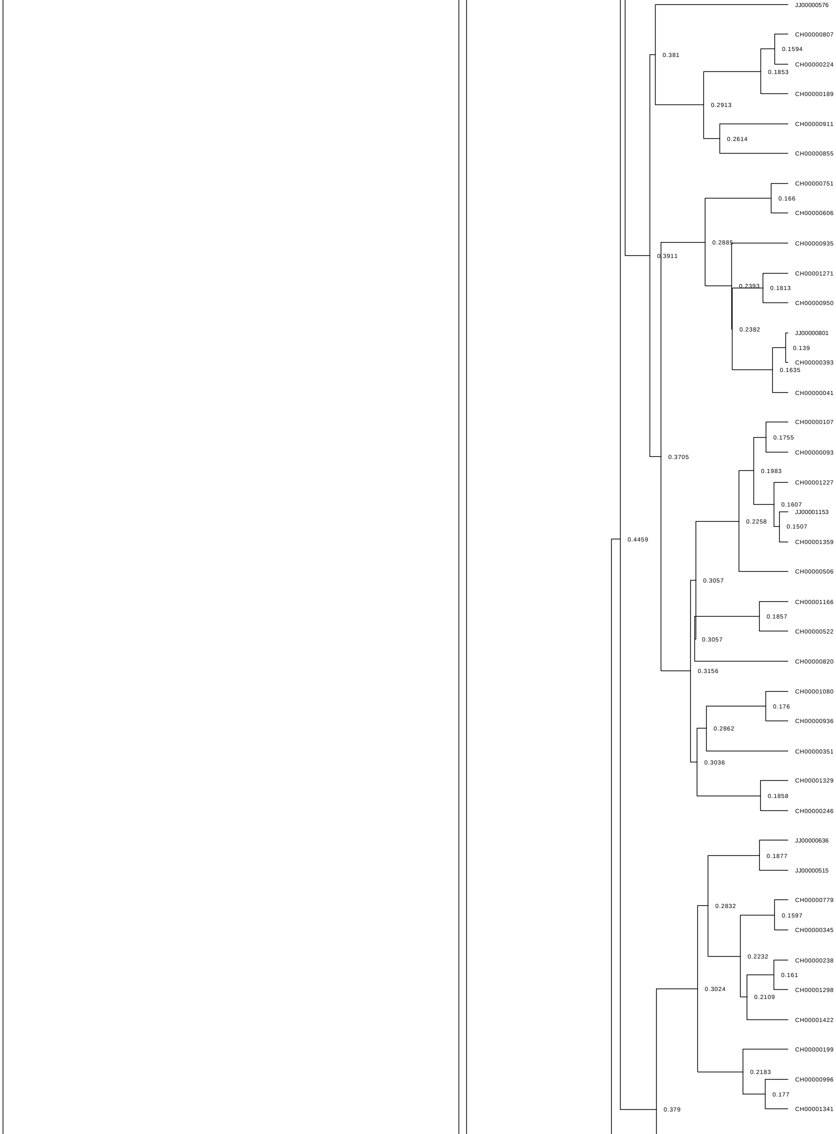
JJ00000576
CH00000807
0.1594
CH00000224
0.381
0.1853
CH00000189
0.2913
CH00000911
0.2614
CH00000855
CH00000751
0.166
CH00000606
0.2885
CH00000935
0.3911
CH00001271
0.2393
0.1813
CH00000950
0.2382
JJ00000801
0.139
CH00000393
0.1635
CH00000041
CH00000107
0.1755
CH00000093
0.3705
0.1983
CH00001227
0.1607
JJ00001153
0.2258
0.1507
0.4459
CH00001359
CH00000506
0.3057
CH00001166
0.1857
CH00000522
0.3057
CH00000820
0.3156
CH00001080
0.176
CH00000936
0.2862
CH00000351
0.3036
CH00001329
0.1858
CH00000246
JJ00000636
0.1877
JJ00000515
CH00000779
0.2832
0.1597
CH00000345
0.2232
CH00000238
0.161
CH00001298
0.3024
0.2109
CH00001422
CH00000199
0.2183
CH00000996
0.177
0.379
CH00001341

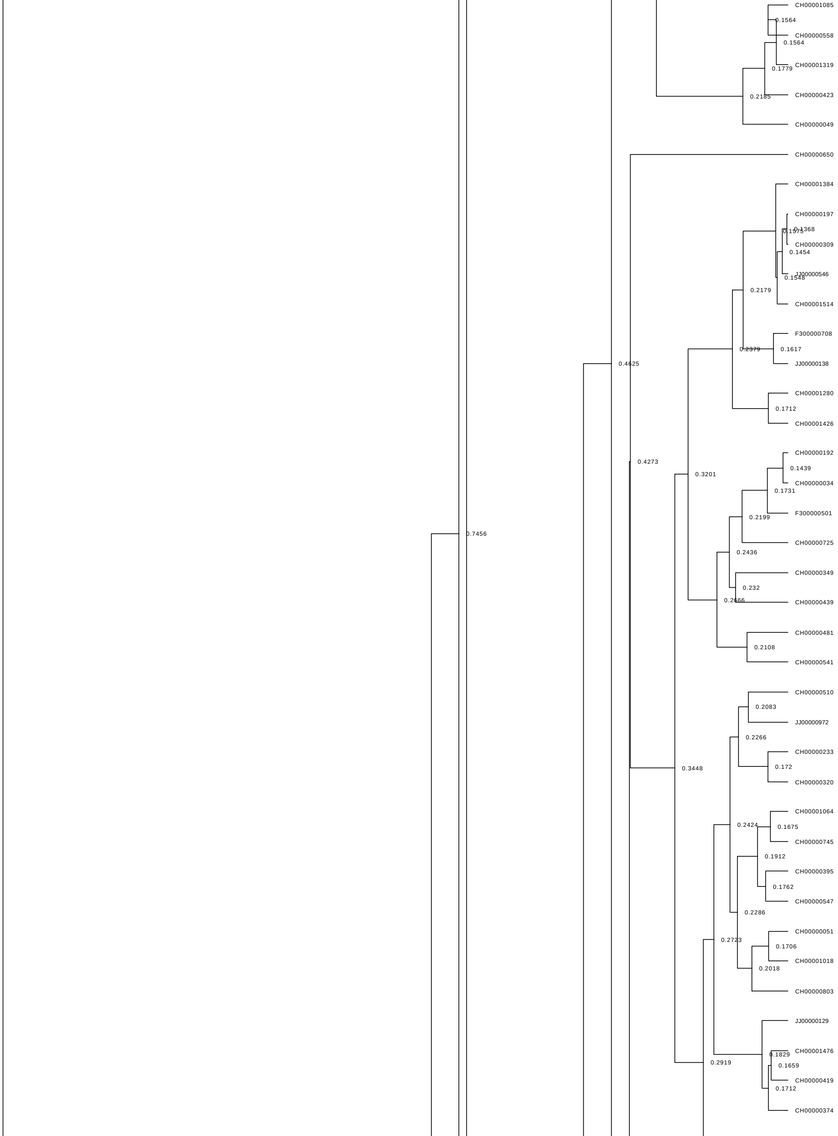

CH00001085
0.1564
CH00000558
0.1564
CH00001319
0.1779
CH00000423
0.2185
CH00000049
CH00000650
CH00001384
CH00000197
0.1368
0.1575
CH00000309
0.1454
JJ00000546
0.1548
0.2179
CH00001514
F300000708
0.2379
0.1617
JJ00000138
0.4625
CH00001280
0.1712
CH00001426
CH00000192
0.1439
0.4273
CH00000034
0.3201
0.1731
F300000501
0.2199
0.7456
CH00000725
0.2436
CH00000349
0.232
0.2666
CH00000439
CH00000481
0.2108
CH00000541
CH00000510
0.2083
JJ00000972
0.2266
CH00000233
0.172
0.3448
CH00000320
CH00001064
0.2424
0.1675
CH00000745
0.1912
CH00000395
0.1762
CH00000547
0.2286
CH00000051
0.2723
0.1706
CH00001018
0.2018
CH00000803
JJ00000129
CH00001476
0.1829
0.1659
0.2919
CH00000419
0.1712
CH00000374

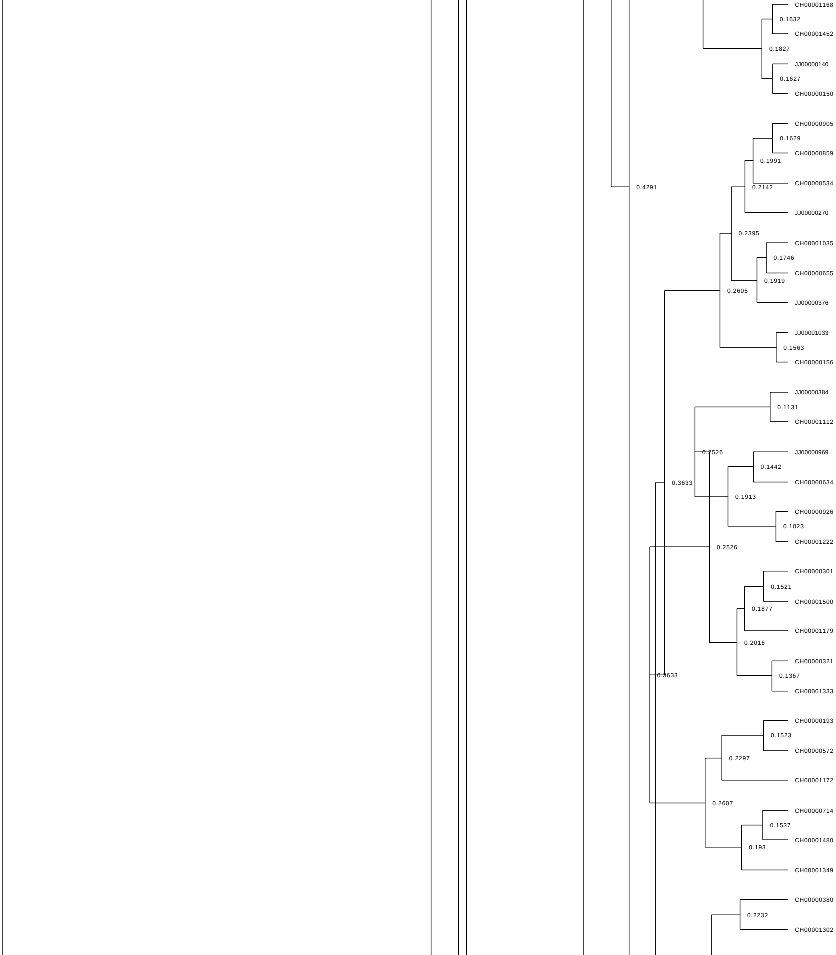
CH00001168
0.1632
CH00001452
0.1827
JJ00000140
0.1627
CH00000150
CH00000905
0.1629
CH00000859
0.1991
0.4291
CH00000534
0.2142
JJ00000270
0.2395
CH00001035
0.1746
CH00000655
0.1919
0.2605
JJ00000376
JJ00001033
0.1563
CH00000156
JJ00000384
0.1131
CH00001112
0.2526
JJ00000969
0.1442
0.3633
CH00000634
0.1913
CH00000926
0.1023
CH00001222
0.2526
CH00000301
0.1521
CH00001500
0.1877
CH00001179
0.2016
CH00000321
0.3633
0.1367
CH00001333
CH00000193
0.1523
CH00000572
0.2297
CH00001172
0.2607
CH00000714
0.1537
CH00001480
0.193
CH00001349
CH00000380
0.2232
CH00001302

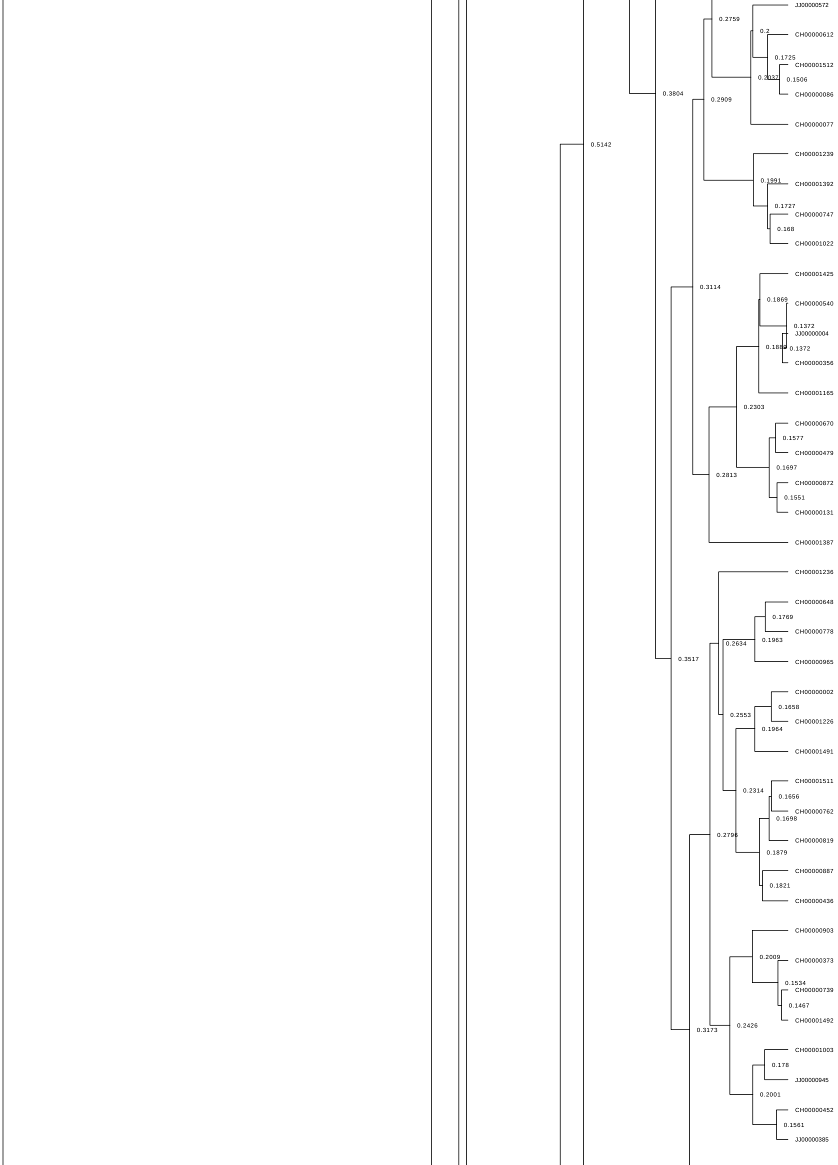
JJ00000572
0.2759
0.2
CH00000612
0.1725
CH00001512
0.2037
0.1506
0.3804
CH00000086
0.2909
CH00000077
0.5142
CH00001239
0.1991
CH00001392
0.1727
CH00000747
0.168
CH00001022
CH00001425
0.3114
0.1869
CH00000540
0.1372
JJ00000004
0.1889
0.1372
CH00000356
CH00001165
0.2303
CH00000670
0.1577
CH00000479
0.1697
0.2813
CH00000872
0.1551
CH00000131
CH00001387
CH00001236
CH00000648
0.1769
CH00000778
0.1963
0.2634
0.3517
CH00000965
CH00000002
0.1658
0.2553
CH00001226
0.1964
CH00001491
CH00001511
0.2314
0.1656
CH00000762
0.1698
0.2796
CH00000819
0.1879
CH00000887
0.1821
CH00000436
CH00000903
0.2009
CH00000373
0.1534
CH00000739
0.1467
CH00001492
0.2426
0.3173
CH00001003
0.178
JJ00000945
0.2001
CH00000452
0.1561
JJ00000385

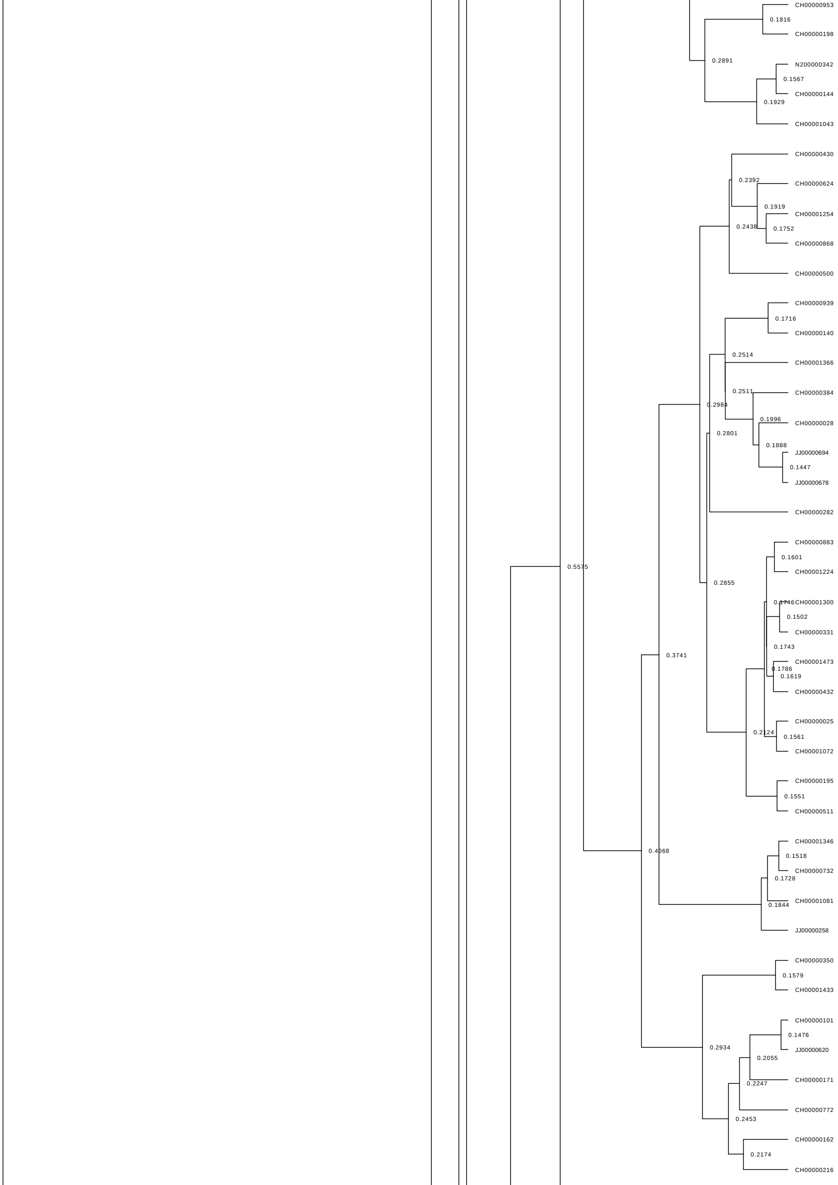

CH00000953
0.1816
CH00000198
0.2891
N200000342
0.1567
CH00000144
0.1929
CH00001043
CH00000430
0.2392
CH00000624
0.1919
CH00001254
0.2438
0.1752
CH00000868
CH00000500
CH00000939
0.1716
CH00000140
0.2514
CH00001366
0.2511
CH00000384
0.2984
0.1996
CH00000028
0.2801
0.1888
JJ00000694
0.1447
JJ00000678
CH00000282
CH00000883
0.1601
0.5575
CH00001224
0.2855
0.1746
CH00001300
0.1502
CH00000331
0.1743
0.3741
CH00001473
0.1786
0.1619
CH00000432
CH00000025
0.2124
0.1561
CH00001072
CH00000195
0.1551
CH00000511
CH00001346
0.1518
0.4068
CH00000732
0.1728
CH00001081
0.1844
JJ00000258
CH00000350
0.1579
CH00001433
CH00000101
0.1476
0.2934
JJ00000620
0.2055
CH00000171
0.2247
CH00000772
0.2453
CH00000162
0.2174
CH00000216

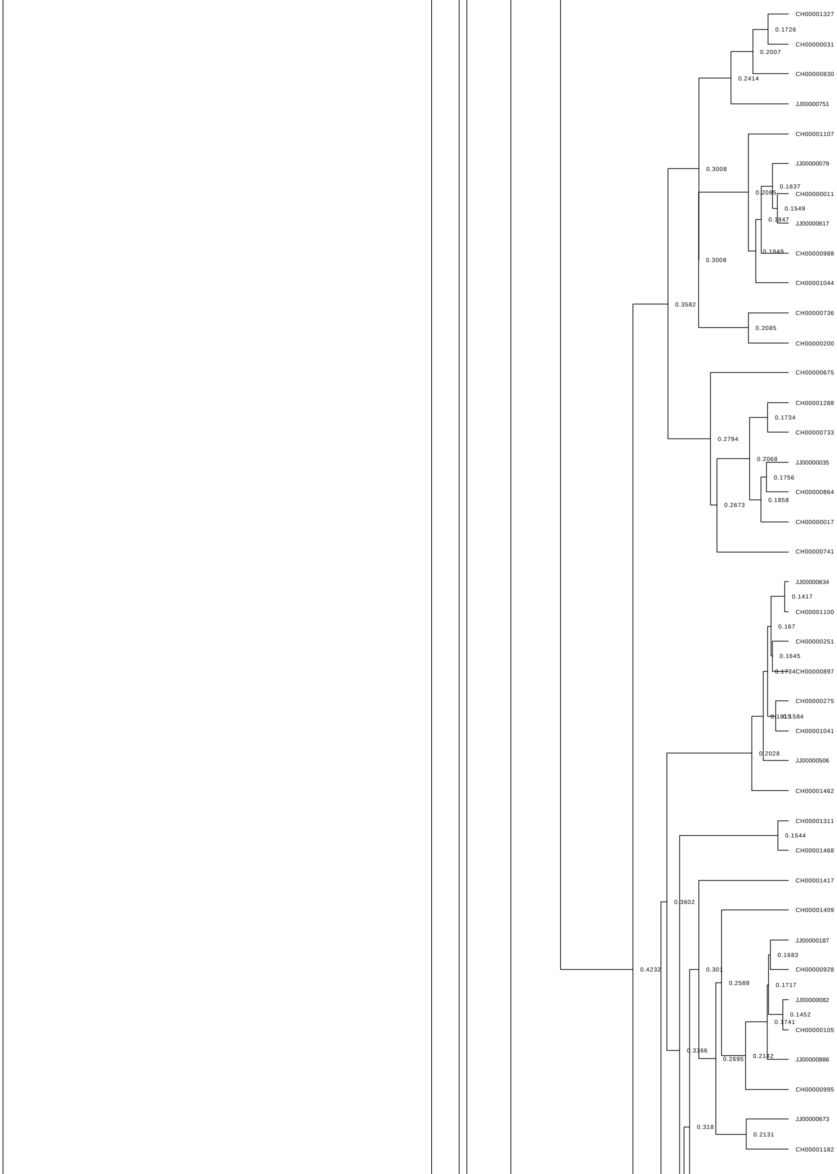

CH00001327
0.1726
CH00000031
0.2007
CH00000830
0.2414
JJ00000751
CH00001107
JJ00000079
0.3008
0.1637
0.2085
CH00000011
0.1549
0.1847
JJ00000617
0.1949
CH00000988
0.3008
CH00001044
0.3582
CH00000736
0.2085
CH00000200
CH00000675
CH00001288
0.1734
CH00000733
0.2794
0.2068
JJ00000035
0.1756
CH00000864
0.1858
0.2673
CH00000017
CH00000741
JJ00000634
0.1417
CH00001100
0.167
CH00000251
0.1645
0.1734
CH00000897
CH00000275
0.1584
CH00001041
0.2028
JJ00000506
CH00001462
CH00001311
0.1544
CH00001468
CH00001417
0.3602
CH00001409
JJ00000187
0.1683
0.4232
0.301
CH00000928
0.2588
0.1717
JJ00000082
0.1452
0.1741
CH00000105
0.3366
0.2695
0.2142
JJ00000886
CH00000995
JJ00000673
0.318
0.2131
CH00001182

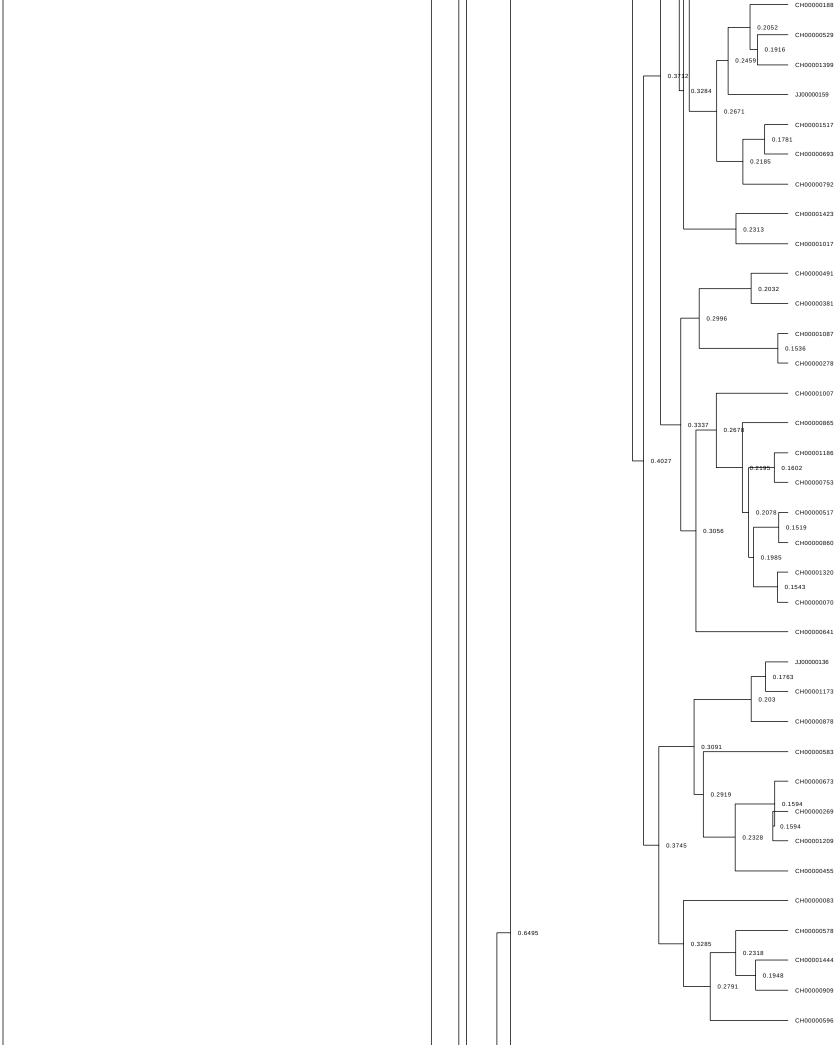
CH00000188
0.2052
CH00000529
0.1916
CH00001399
0.2459
0.3712
0.3284
JJ00000159
0.2671
CH00001517
0.1781
CH00000693
0.2185
CH00000792
CH00001423
0.2313
CH00001017
CH00000491
0.2032
CH00000381
0.2996
CH00001087
0.1536
CH00000278
CH00001007
CH00000865
0.3337
0.2678
CH00001186
0.4027
0.2195
0.1602
CH00000753
0.2078
CH00000517
0.1519
CH00000860
0.3056
0.1985
CH00001320
0.1543
CH00000070
CH00000641
JJ00000136
0.1763
CH00001173
0.203
CH00000878
0.3091
CH00000583
CH00000673
0.2919
0.1594
CH00000269
0.1594
CH00001209
0.2328
0.3745
CH00000455
CH00000083
0.6495
CH00000578
0.3285
0.2318
CH00001444
0.1948
CH00000909
0.2791
CH00000596

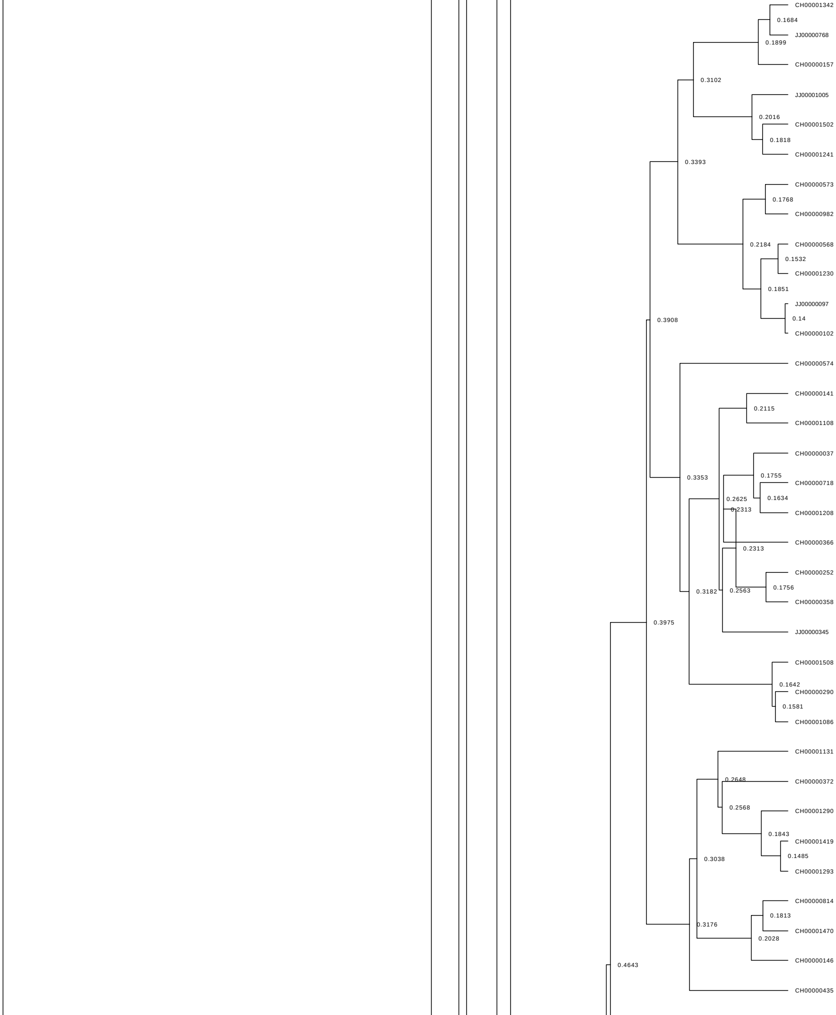
CH00001342
0.1684
JJ00000768
0.1899
CH00000157
0.3102
JJ00001005
0.2016
CH00001502
0.1818
CH00001241
0.3393
CH00000573
0.1768
CH00000982
0.2184
CH00000568
0.1532
CH00001230
0.1851
JJ00000097
0.14
CH00000102
0.3908
CH00000574
CH00000141
0.2115
CH00001108
CH00000037
0.1755
CH00000718
0.2625
0.1634
0.2313
CH00001208
CH00000366
0.2313
CH00000252
0.1756
0.3353
0.3182
0.2563
CH00000358
0.3975
JJ00000345
CH00001508
0.1642
CH00000290
0.1581
CH00001086
CH00001131
0.2648
CH00000372
0.2568
CH00001290
0.1843
CH00001419
0.1485
CH00001293
0.3038
CH00000814
0.1813
CH00001470
0.3176
0.2028
CH00000146
0.4643
CH00000435

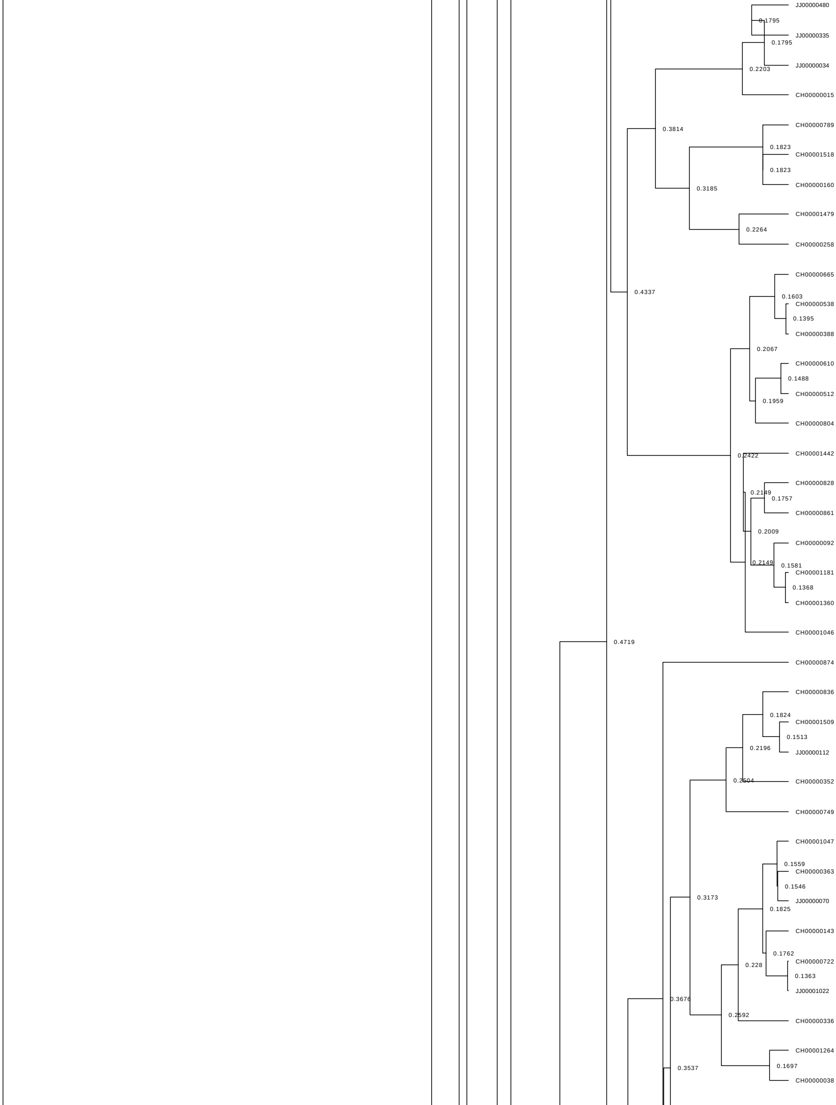

JJ00000480
0.1795
JJ00000335
0.1795
JJ00000034
0.2203
CH00000015
0.3814
CH00000789
0.1823
CH00001518
0.1823
CH00000160
0.3185
CH00001479
0.2264
CH00000258
CH00000665
0.4337
0.1603
CH00000538
0.1395
CH00000388
0.2067
CH00000610
0.1488
CH00000512
0.1959
CH00000804
CH00001442
0.2422
CH00000828
0.2149
0.1757
CH00000861
0.2009
CH00000092
0.2149
0.1581
CH00001181
0.1368
CH00001360
CH00001046
0.4719
CH00000874
CH00000836
0.1824
CH00001509
0.1513
JJ00000112
0.2196
0.2504
CH00000352
CH00000749
CH00001047
0.1559
CH00000363
0.1546
JJ00000070
0.3173
0.1825
CH00000143
0.1762
CH00000722
0.228
0.1363
JJ00001022
0.3676
0.2592
CH00000336
CH00001264
0.1697
0.3537
CH00000038

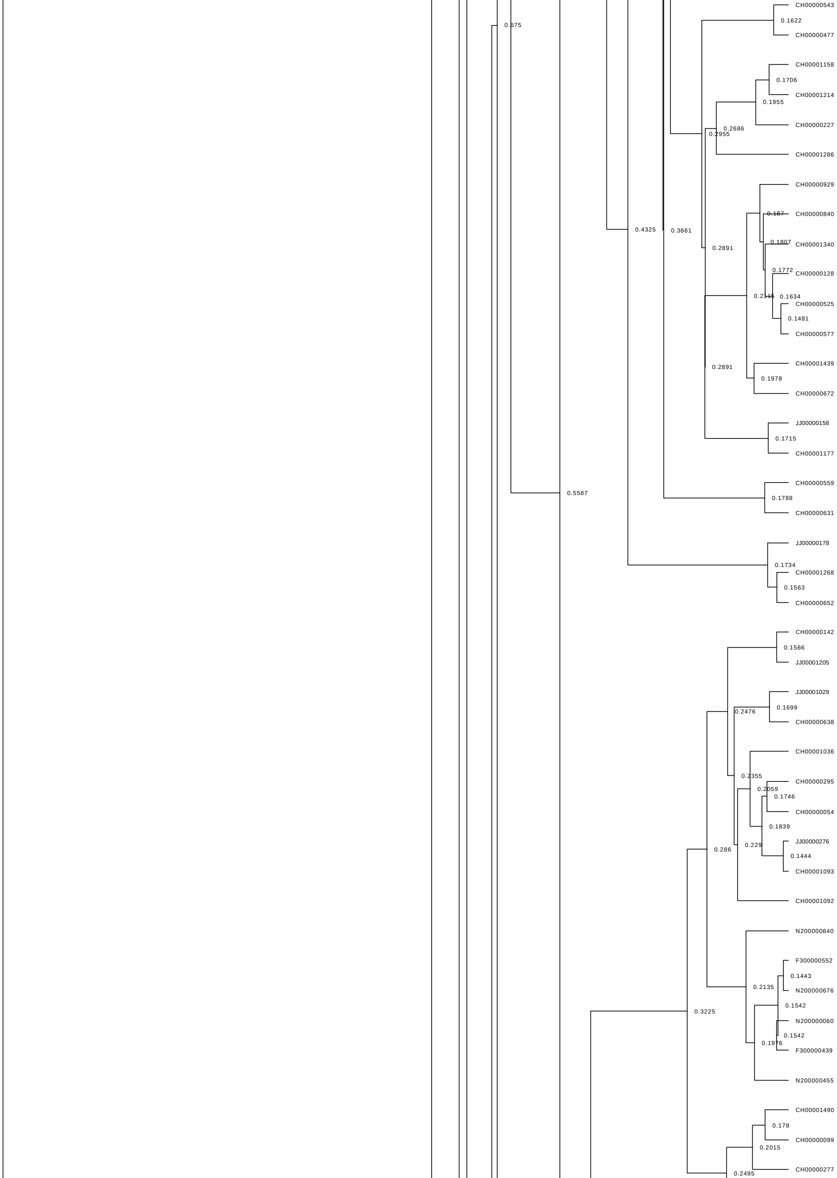
0.675
CH00000543
0.1622
CH00000477
CH00001158
0.1706
CH00001214
0.1955
CH00000227
0.2686
0.2955
CH00001286
CH00000929
0.187
CH00000840
0.4325
0.3661
0.1807
CH00001340
0.2891
0.1772
CH00000128
0.2116
0.1634
CH00000525
0.1481
CH00000577
CH00001439
0.2891
0.1978
CH00000672
JJ00000158
0.1715
CH00001177
CH00000559
0.5587
0.1788
CH00000631
JJ00000178
0.1734
CH00001268
0.1563
CH00000652
CH00000142
0.1566
JJ00001205
JJ00001029
0.1699
0.2476
CH00000638
CH00001036
0.2355
CH00000295
0.2059
0.1746
CH00000054
0.1839
JJ00000276
0.229
0.286
0.1444
CH00001093
CH00001092
N200000640
F300000552
0.1443
0.2135
N200000676
0.1542
0.3225
N200000060
0.1542
0.1976
F300000439
N200000455
CH00001490
0.178
CH00000099
0.2015
CH00000277
0.2495

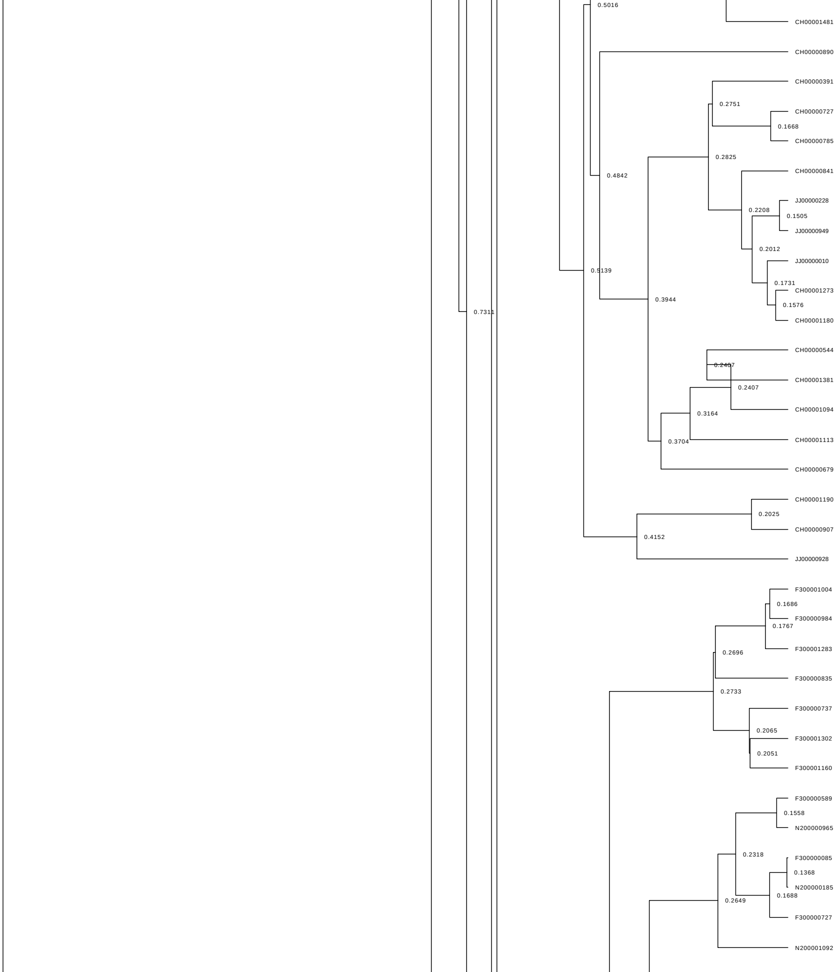
0.5016
CH00001481
CH00000890
CH00000391
0.2751
CH00000727
0.1668
CH00000785
0.2825
CH00000841
0.4842
JJ00000228
0.2208
0.1505
JJ00000949
0.2012
JJ00000010
0.5139
0.1731
CH00001273
0.3944
0.1576
0.7311
CH00001180
CH00000544
0.2407
CH00001381
0.2407
CH00001094
0.3164
CH00001113
0.3704
CH00000679
CH00001190
0.2025
CH00000907
0.4152
JJ00000928
F300001004
0.1686
F300000984
0.1767
F300001283
0.2696
F300000835
0.2733
F300000737
0.2065
F300001302
0.2051
F300001160
F300000589
0.1558
N200000965
0.2318
F300000085
0.1368
N200000185
0.1688
0.2649
F300000727
N200001092

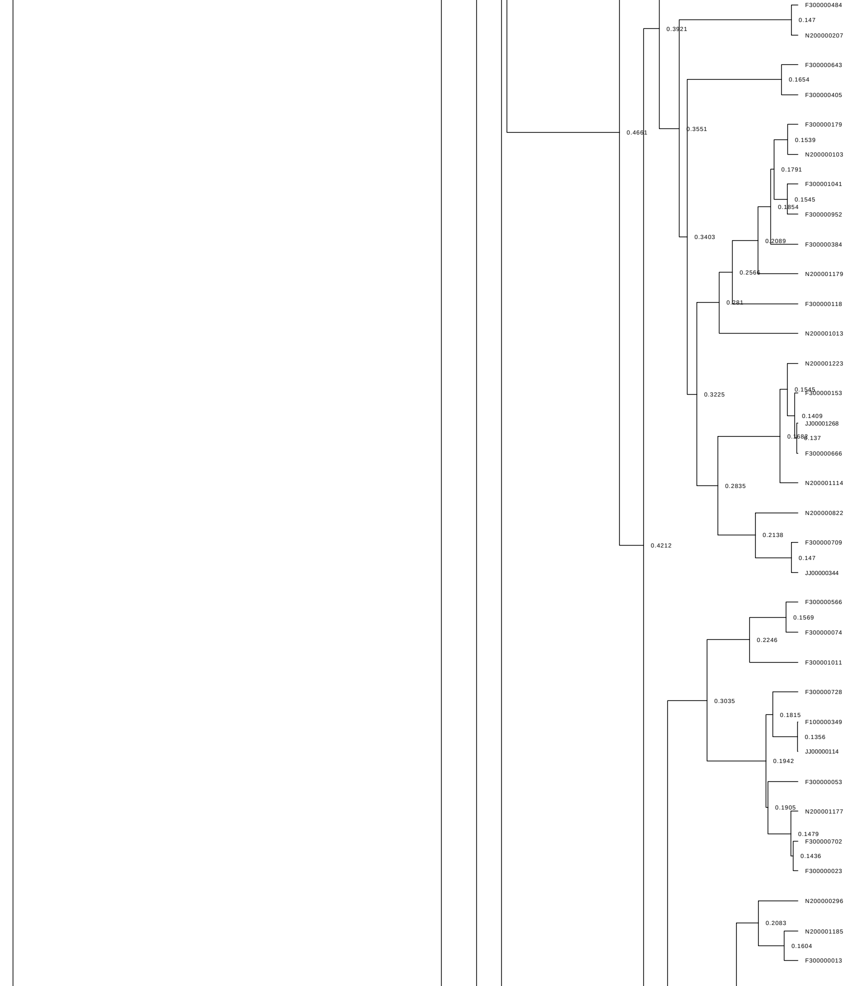
F300000484
0.147
N200000207
0.3921
F300000643
0.1654
F300000405
0.4661
0.3551
F300000179
0.1539
N200000103
0.1791
F300001041
0.1545
0.1854
F300000952
0.3403
0.2089
F300000384
0.2566
N200001179
0.281
F300000118
N200001013
N200001223
0.1545
F300000153
0.3225
0.1409
JJ00001268
0.1682
0.137
F300000666
N200001114
0.2835
N200000822
0.2138
F300000709
0.4212
0.147
JJ00000344
F300000566
0.1569
F300000074
0.2246
F300001011
0.3035
F300000728
0.1815
F100000349
0.1356
JJ00000114
0.1942
F300000053
0.1905
N200001177
0.1479
F300000702
0.1436
F300000023
N200000296
0.2083
N200001185
0.1604
F300000013

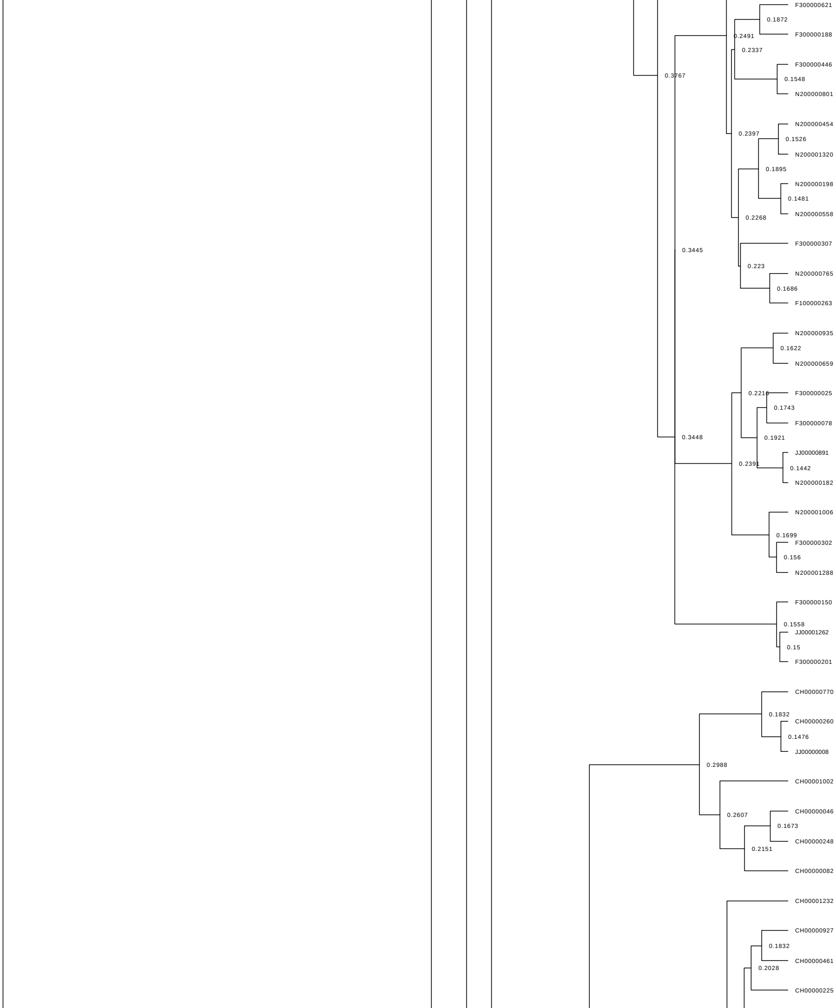
F300000621
0.1872
F300000188
0.2491
0.2337
F300000446
0.1548
N200000801
0.3767
N200000454
0.2397
0.1526
N200001320
0.1895
N200000198
0.1481
N200000558
0.2268
F300000307
0.3445
0.223
N200000765
0.1686
F100000263
N200000935
0.1622
N200000659
0.2216
F300000025
0.1743
F300000078
0.3448
0.1921
JJ00000891
0.2391
0.1442
N200000182
N200001006
0.1699
F300000302
0.156
N200001288
F300000150
0.1558
JJ00001262
0.15
F300000201
CH00000770
0.1832
CH00000260
0.1476
JJ00000008
0.2988
CH00001002
0.2607
CH00000046
0.1673
CH00000248
0.2151
CH00000082
CH00001232
CH00000927
0.1832
CH00000461
0.2028
CH00000225

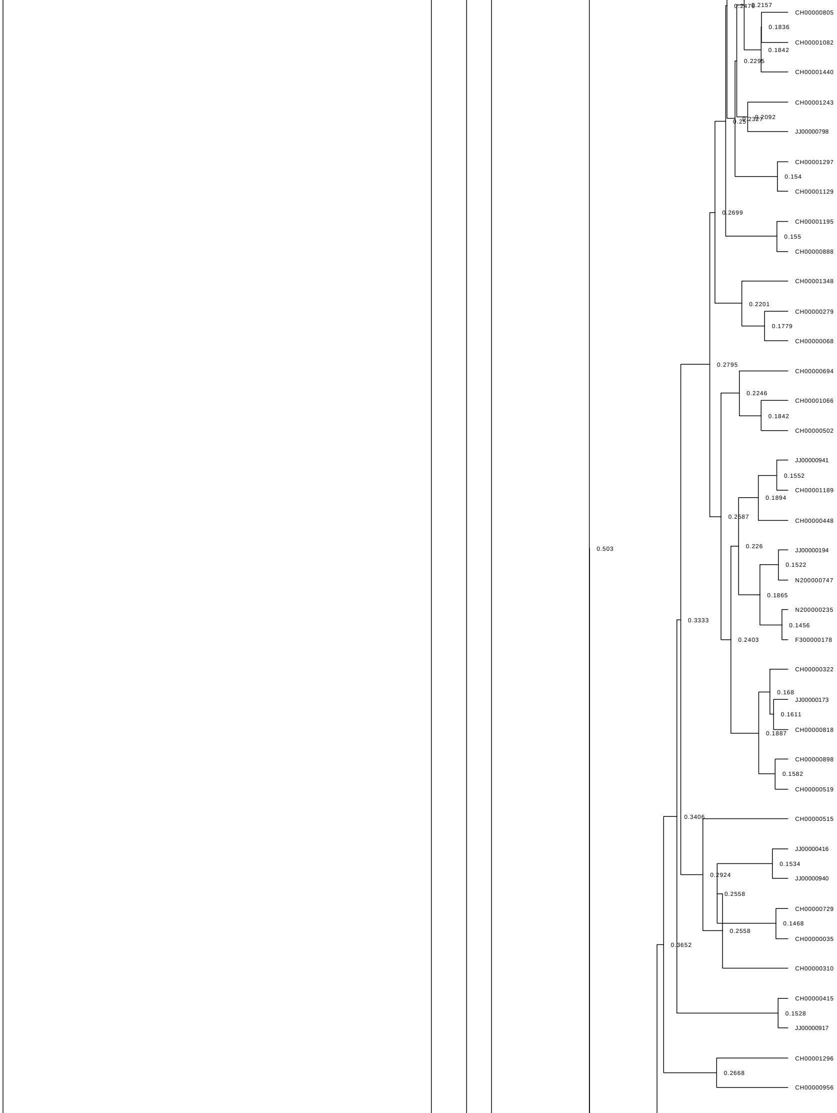
0.2157
CH00000805
0.1836
CH00001082
0.1842
0.2295
CH00001440
CH00001243
0.2092
0.2327
JJ00000798
CH00001297
0.154
CH00001129
0.2699
CH00001195
0.155
CH00000888
CH00001348
0.2201
CH00000279
0.1779
CH00000068
0.2795
CH00000694
0.2246
CH00001066
0.1842
CH00000502
JJ00000941
0.1552
CH00001189
0.1894
CH00000448
0.226
JJ00000194
0.1522
N200000747
0.503
0.1865
N200000235
0.3333
0.1456
0.2403
F300000178
CH00000322
0.168
JJ00000173
0.1611
CH00000818
0.1887
CH00000898
0.1582
CH00000519
0.3406
CH00000515
JJ00000416
0.1534
0.2924
JJ00000940
0.2558
CH00000729
0.1468
0.2558
CH00000035
0.3652
CH00000310
CH00000415
0.1528
JJ00000917
CH00001296
0.2668
CH00000956

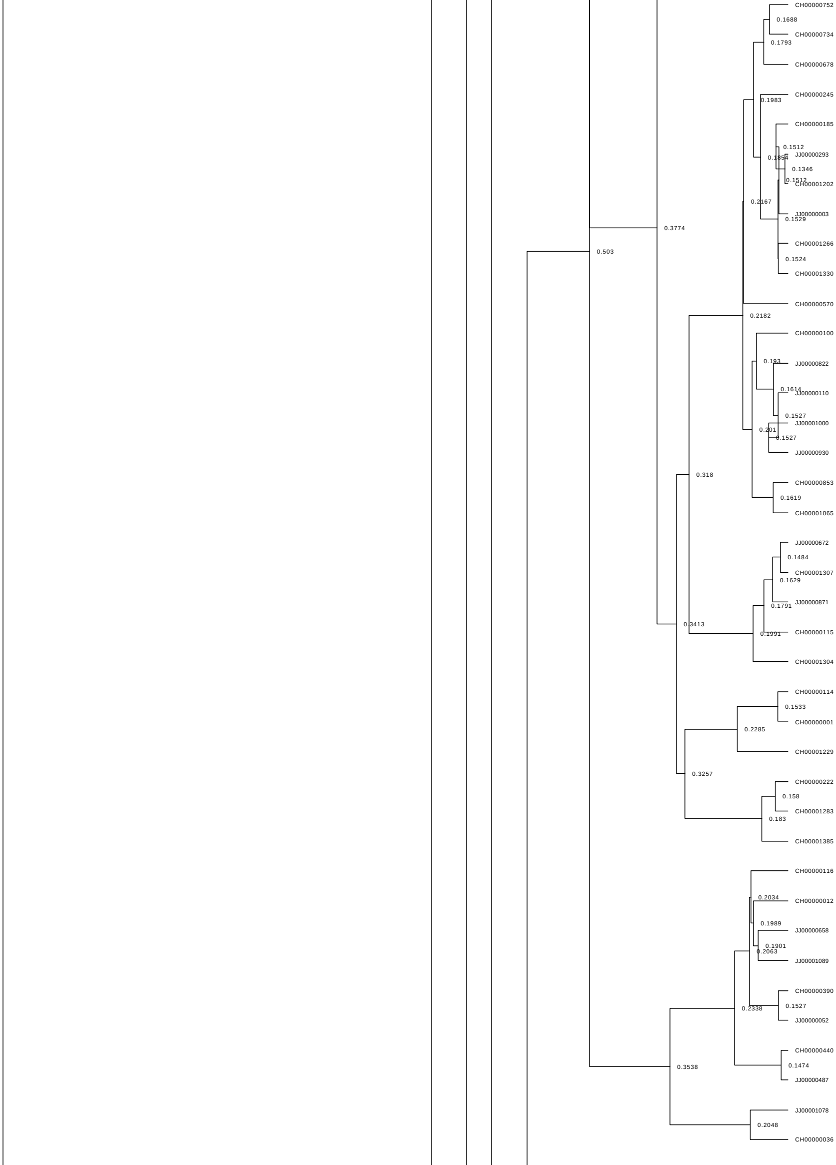

CH00000752
0.1688
CH00000734
0.1793
CH00000678
CH00000245
0.1983
CH00000185
0.1512
0.1854
JJ00000293
0.1346
0.1512
CH00001202
0.2167
JJ00000003
0.1529
0.3774
CH00001266
0.503
0.1524
CH00001330
CH00000570
0.2182
CH00000100
0.193
JJ00000822
0.1614
JJ00000110
0.1527
JJ00001000
0.201
0.1527
JJ00000930
0.318
CH00000853
0.1619
CH00001065
JJ00000672
0.1484
CH00001307
0.1629
JJ00000871
0.1791
0.3413
CH00000115
0.1991
CH00001304
CH00000114
0.1533
CH00000001
0.2285
CH00001229
0.3257
CH00000222
0.158
CH00001283
0.183
CH00001385
CH00000116
0.2034
CH00000012
0.1989
JJ00000658
0.1901
0.2063
JJ00001089
CH00000390
0.1527
0.2338
JJ00000052
CH00000440
0.1474
0.3538
JJ00000487
JJ00001078
0.2048
CH00000036

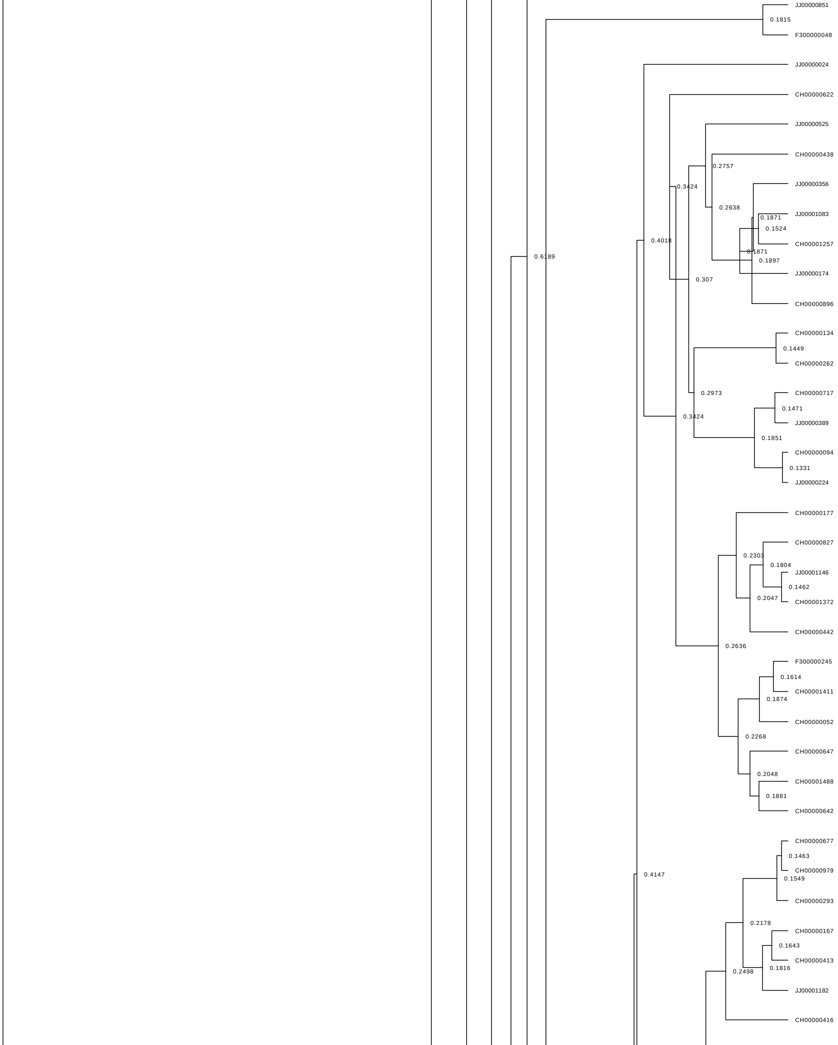
JJ00000851
0.1815
F300000048
JJ00000024
CH00000622
JJ00000525
CH00000438
0.2757
0.3424
JJ00000356
0.2638
JJ00001083
0.1871
0.1524
CH00001257
0.4018
0.1871
0.6189
0.1897
JJ00000174
0.307
CH00000896
CH00000134
0.1449
CH00000262
CH00000717
0.2973
0.1471
0.3424
JJ00000389
0.1851
CH00000094
0.1331
JJ00000224
CH00000177
CH00000827
0.2303
0.1804
JJ00001146
0.1462
0.2047
CH00001372
CH00000442
0.2636
F300000245
0.1614
CH00001411
0.1874
CH00000052
0.2268
CH00000647
0.2048
CH00001488
0.1881
CH00000642
CH00000677
0.1463
CH00000979
0.4147
0.1549
CH00000293
0.2178
CH00000167
0.1643
CH00000413
0.1816
0.2498
JJ00001182
CH00000416

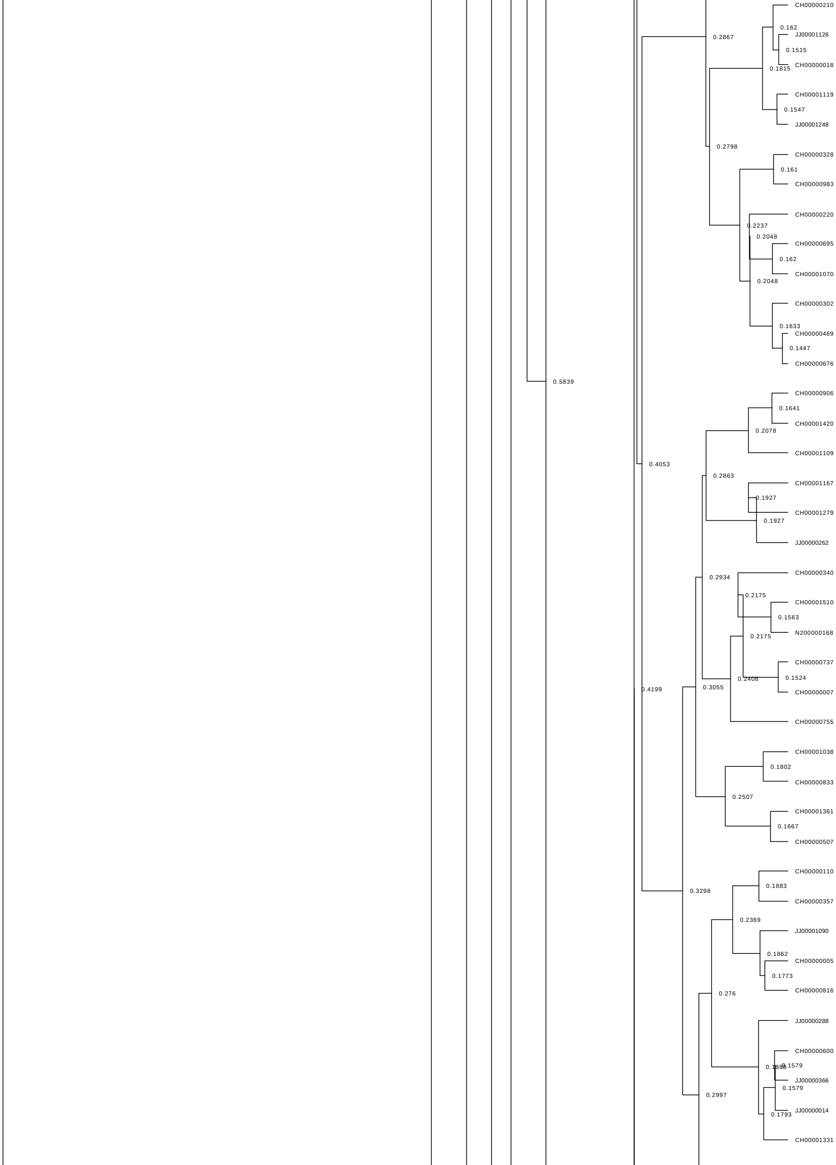

CH00000210
0.162
JJ00001126
0.1515
CH00000018
0.1815
0.2867
CH00001119
0.1547
JJ00001248
0.2798
CH00000328
0.161
CH00000983
CH00000220
0.2237
0.2048
CH00000695
0.162
CH00001070
0.2048
CH00000302
0.1633
CH00000469
0.1447
CH00000676
0.5839
CH00000906
0.1641
CH00001420
0.2078
CH00001109
0.4053
0.2863
CH00001167
0.1927
CH00001279
0.1927
JJ00000262
CH00000340
0.2934
0.2175
CH00001510
0.1563
N200000168
0.2175
CH00000737
0.1524
0.2408
CH00000007
0.3055
0.4199
CH00000755
CH00001038
0.1802
CH00000833
0.2507
CH00001361
0.1667
CH00000507
CH00000110
0.1883
0.3298
CH00000357
0.2369
JJ00001090
0.1862
CH00000005
0.1773
CH00000816
0.276
JJ00000288
CH00000600
0.1579
JJ00000366
0.1579
0.2997
JJ00000014
0.1793
CH00001331

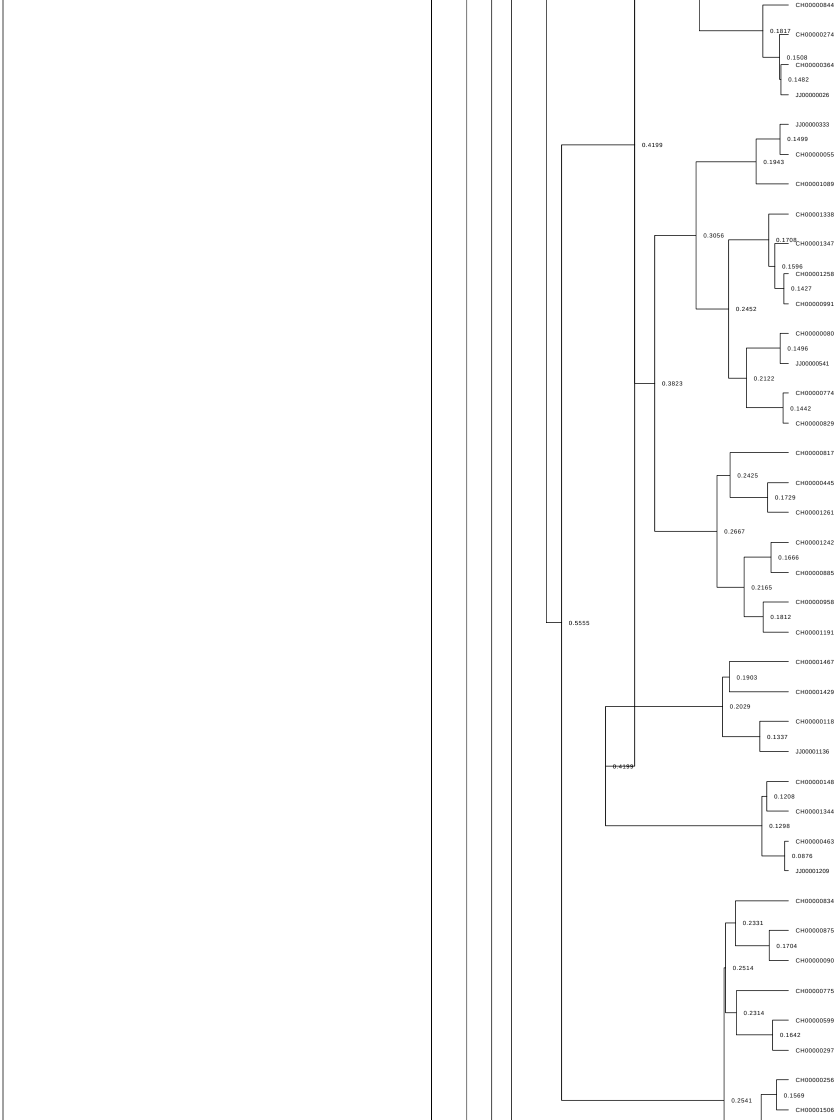

CH00000844
0.1817
CH00000274
0.1508
CH00000364
0.1482
JJ00000026
JJ00000333
0.1499
CH00000055
0.4199
0.1943
CH00001089
CH00001338
0.3056
0.1708
CH00001347
0.1596
CH00001258
0.1427
CH00000991
0.2452
CH00000080
0.1496
JJ00000541
0.2122
0.3823
CH00000774
0.1442
CH00000829
CH00000817
0.2425
CH00000445
0.1729
CH00001261
0.2667
CH00001242
0.1666
CH00000885
0.2165
CH00000958
0.1812
0.5555
CH00001191
CH00001467
0.1903
CH00001429
0.2029
CH00000118
0.1337
JJ00001136
0.4199
CH00000148
0.1208
CH00001344
0.1298
CH00000463
0.0876
JJ00001209
CH00000834
0.2331
CH00000875
0.1704
CH00000090
0.2514
CH00000775
0.2314
CH00000599
0.1642
CH00000297
CH00000256
0.1569
0.2541
CH00001506

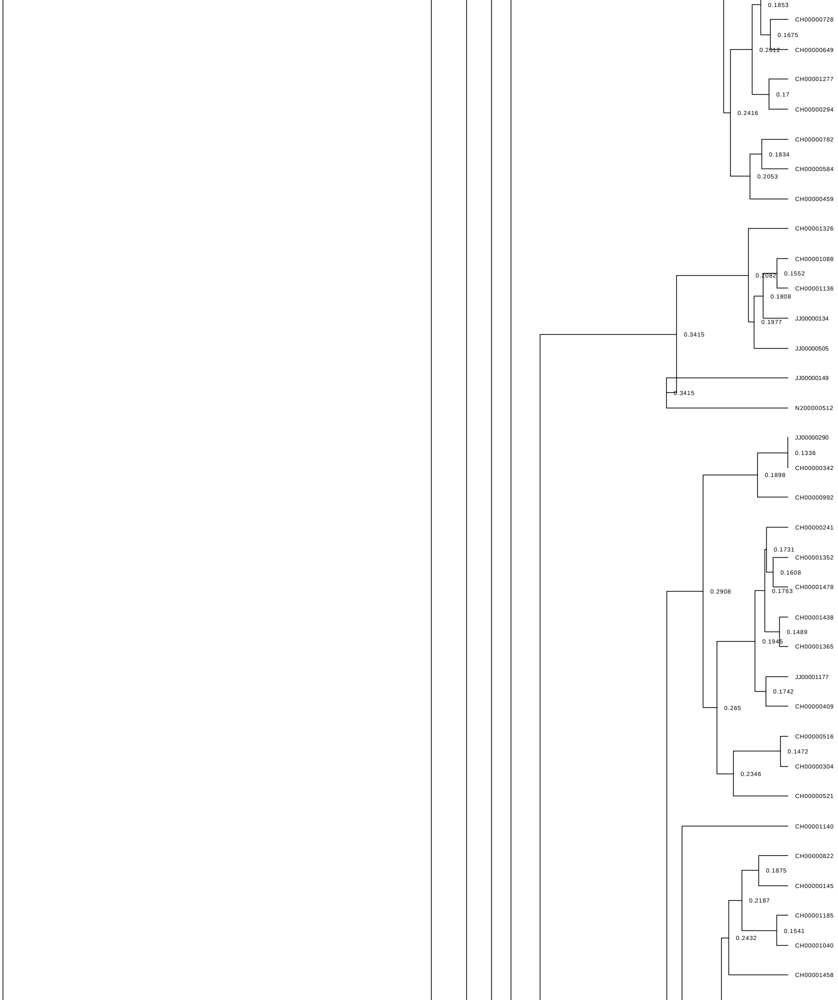

0.1853
CH00000728
0.1675
0.2012
CH00000649
CH00001277
0.17
CH00000294
0.2416
CH00000782
0.1834
CH00000584
0.2053
CH00000459
CH00001326
CH00001088
0.2082
0.1552
CH00001136
0.1808
0.1977
JJ00000134
0.3415
JJ00000505
JJ00000149
0.3415
N200000512
JJ00000290
0.1336
CH00000342
0.1898
CH00000992
CH00000241
0.1731
CH00001352
0.1608
CH00001478
0.2908
0.1763
CH00001438
0.1489
0.1945
CH00001365
JJ00001177
0.1742
CH00000409
0.265
CH00000516
0.1472
CH00000304
0.2346
CH00000521
CH00001140
CH00000822
0.1875
CH00000145
0.2187
CH00001185
0.1541
0.2432
CH00001040
CH00001458

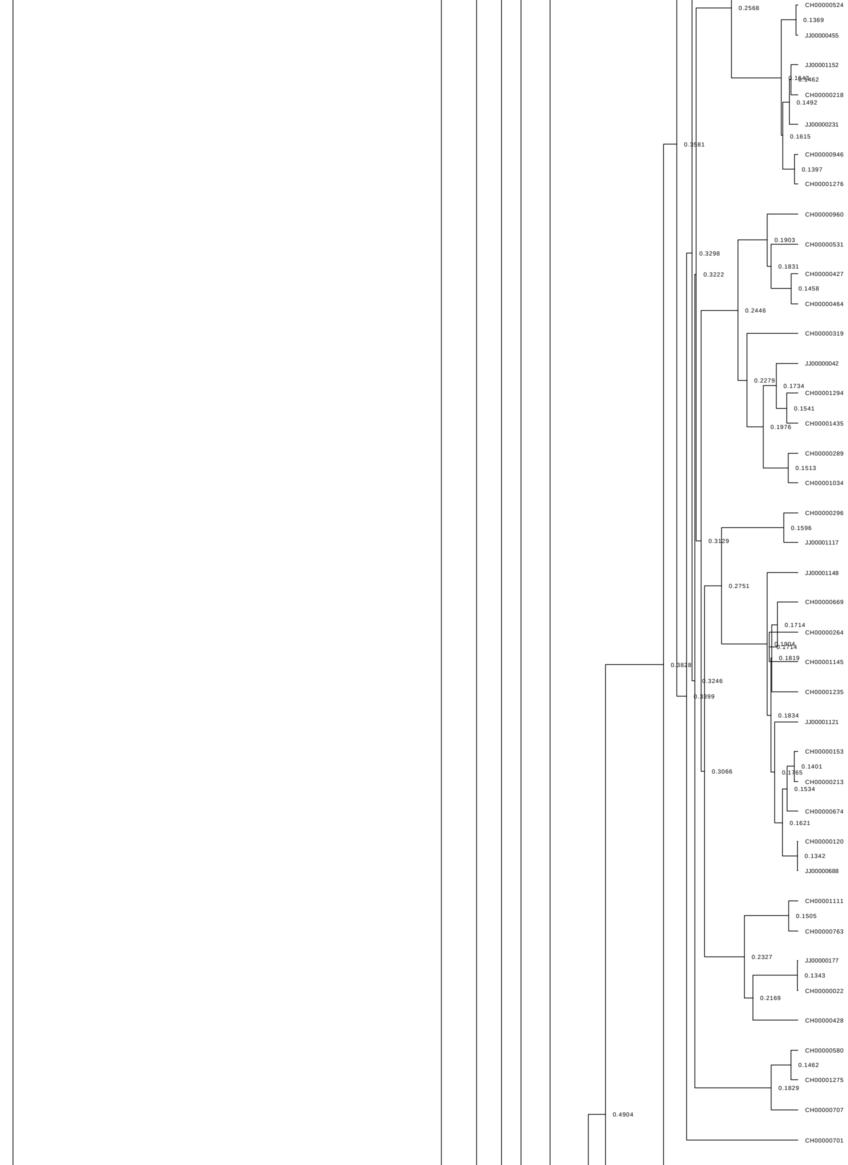
0.2568
CH00000524
0.1369
JJ00000455
JJ00001152
0.1643
0.1462
CH00000218
0.1492
JJ00000231
0.1615
0.3581
CH00000946
0.1397
CH00001276
CH00000960
0.1903
CH00000531
0.3298
0.1831
0.3222
CH00000427
0.1458
CH00000464
0.2446
CH00000319
JJ00000042
0.2279
0.1734
CH00001294
0.1541
CH00001435
0.1976
CH00000289
0.1513
CH00001034
CH00000296
0.1596
0.3129
JJ00001117
JJ00001148
0.2751
CH00000669
0.1714
CH00000264
0.1904
0.1714
0.1819
CH00001145
0.3828
0.3246
CH00001235
0.3399
0.1834
JJ00001121
CH00000153
0.1401
0.3066
0.1765
CH00000213
0.1534
CH00000674
0.1621
CH00000120
0.1342
JJ00000688
CH00001111
0.1505
CH00000763
0.2327
JJ00000177
0.1343
CH00000022
0.2169
CH00000428
CH00000580
0.1462
CH00001275
0.1829
CH00000707
0.4904
CH00000701

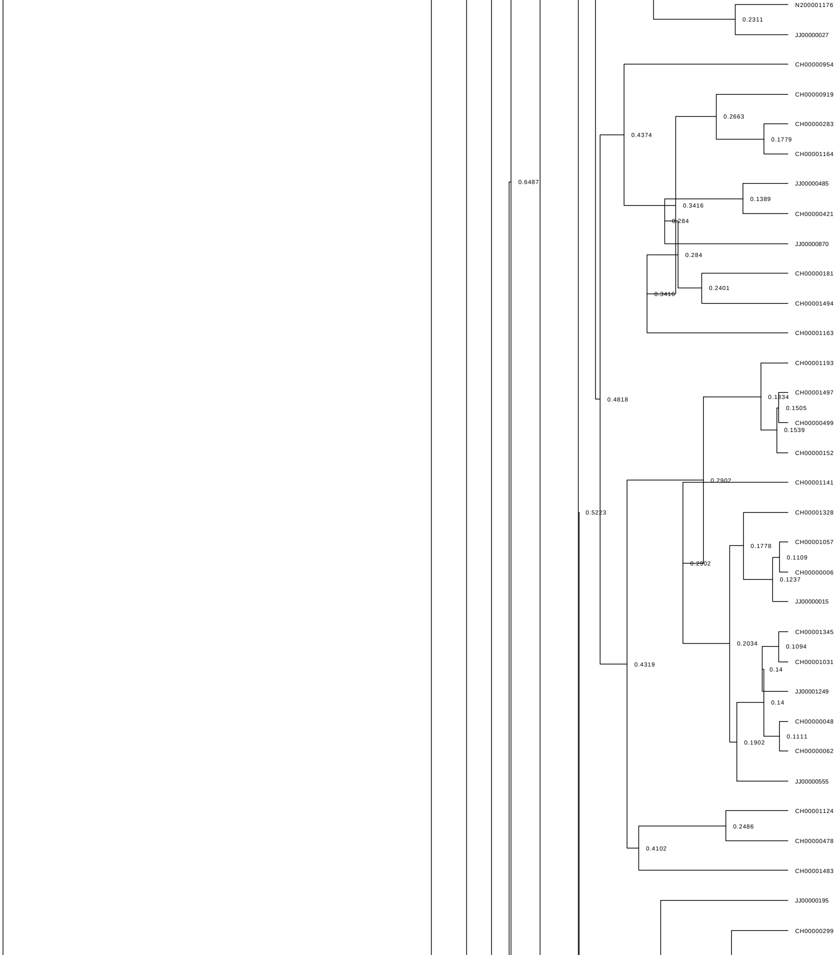
N200001176
0.2311
JJ00000027
CH00000954
CH00000919
0.2663
CH00000283
0.4374
0.1779
CH00001164
0.6487
JJ00000485
0.1389
0.3416
CH00000421
0.284
JJ00000870
0.284
CH00000181
0.2401
0.3416
CH00001494
CH00001163
CH00001193
CH00001497
0.4818
0.1834
0.1505
CH00000499
0.1539
CH00000152
0.2902
CH00001141
0.5223
CH00001328
CH00001057
0.1778
0.1109
0.2902
CH00000006
0.1237
JJ00000015
CH00001345
0.2034
0.1094
0.4319
CH00001031
0.14
JJ00001249
0.14
CH00000048
0.1111
0.1902
CH00000062
JJ00000555
CH00001124
0.2486
CH00000478
0.4102
CH00001483
JJ00000195
CH00000299

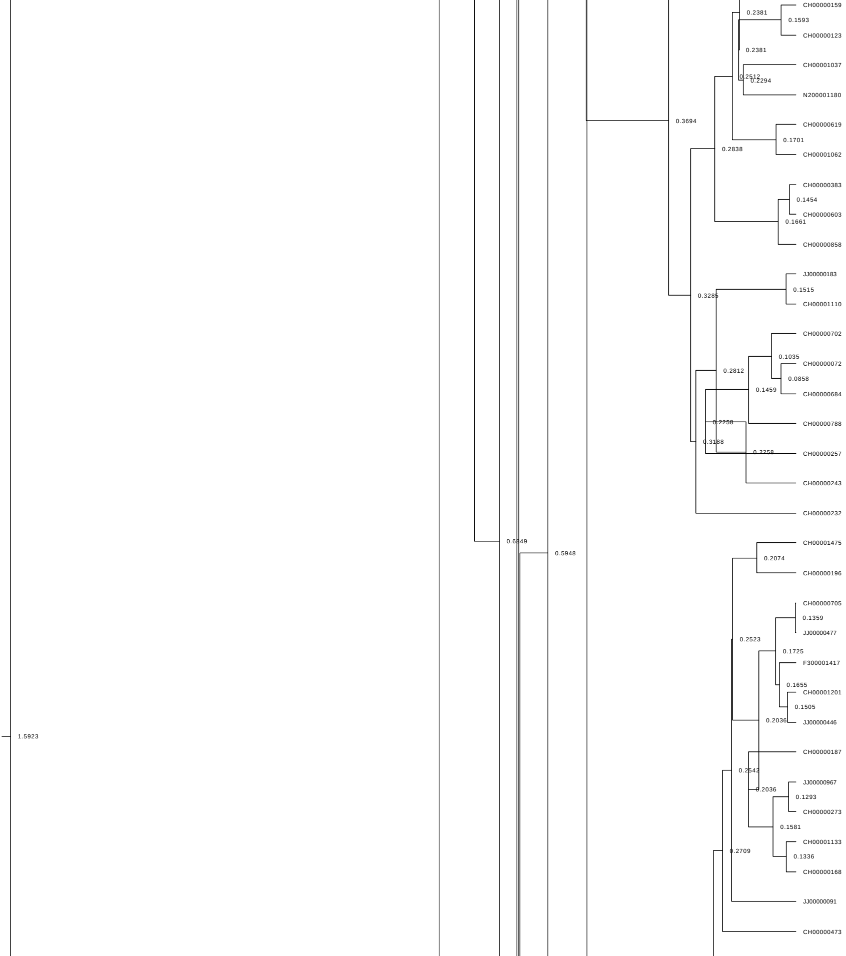
CH00000159
0.2381
0.1593
CH00000123
0.2381
CH00001037
0.2512
0.2294
N200001180
0.3694
CH00000619
0.1701
0.2838
CH00001062
CH00000383
0.1454
CH00000603
0.1661
CH00000858
JJ00000183
0.1515
0.3285
CH00001110
CH00000702
0.1035
CH00000072
0.2812
0.0858
0.1459
CH00000684
0.2258
CH00000788
0.3188
0.2258
CH00000257
CH00000243
CH00000232
0.6849
CH00001475
0.5948
0.2074
CH00000196
CH00000705
0.1359
JJ00000477
0.2523
0.1725
F300001417
0.1655
CH00001201
0.1505
0.2036
JJ00000446
CH00000187
0.2542
JJ00000967
0.2036
0.1293
CH00000273
0.1581
CH00001133
0.2709
0.1336
CH00000168
1.5923
JJ00000091
CH00000473

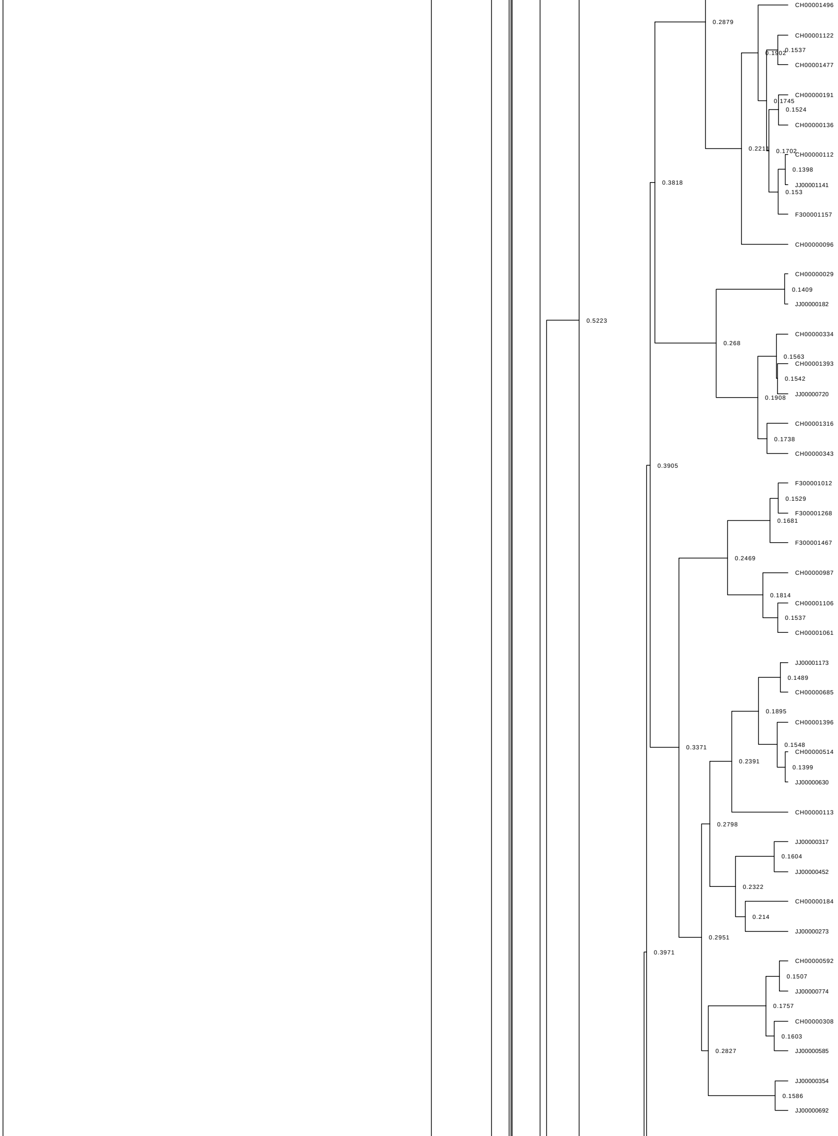
CH00001496
0.2879
CH00001122
0.1537
0.1902
CH00001477
CH00000191
0.1745
0.1524
CH00000136
0.2211
0.1702
CH00000112
0.1398
JJ00001141
0.153
0.3818
F300001157
CH00000096
CH00000029
0.1409
JJ00000182
0.5223
CH00000334
0.268
0.1563
CH00001393
0.1542
JJ00000720
0.1908
CH00001316
0.1738
CH00000343
0.3905
F300001012
0.1529
F300001268
0.1681
F300001467
0.2469
CH00000987
0.1814
CH00001106
0.1537
CH00001061
JJ00001173
0.1489
CH00000685
0.1895
CH00001396
0.1548
0.3371
CH00000514
0.2391
0.1399
JJ00000630
CH00000113
0.2798
JJ00000317
0.1604
JJ00000452
0.2322
CH00000184
0.214
JJ00000273
0.2951
0.3971
CH00000592
0.1507
JJ00000774
0.1757
CH00000308
0.1603
JJ00000585
0.2827
JJ00000354
0.1586
JJ00000692

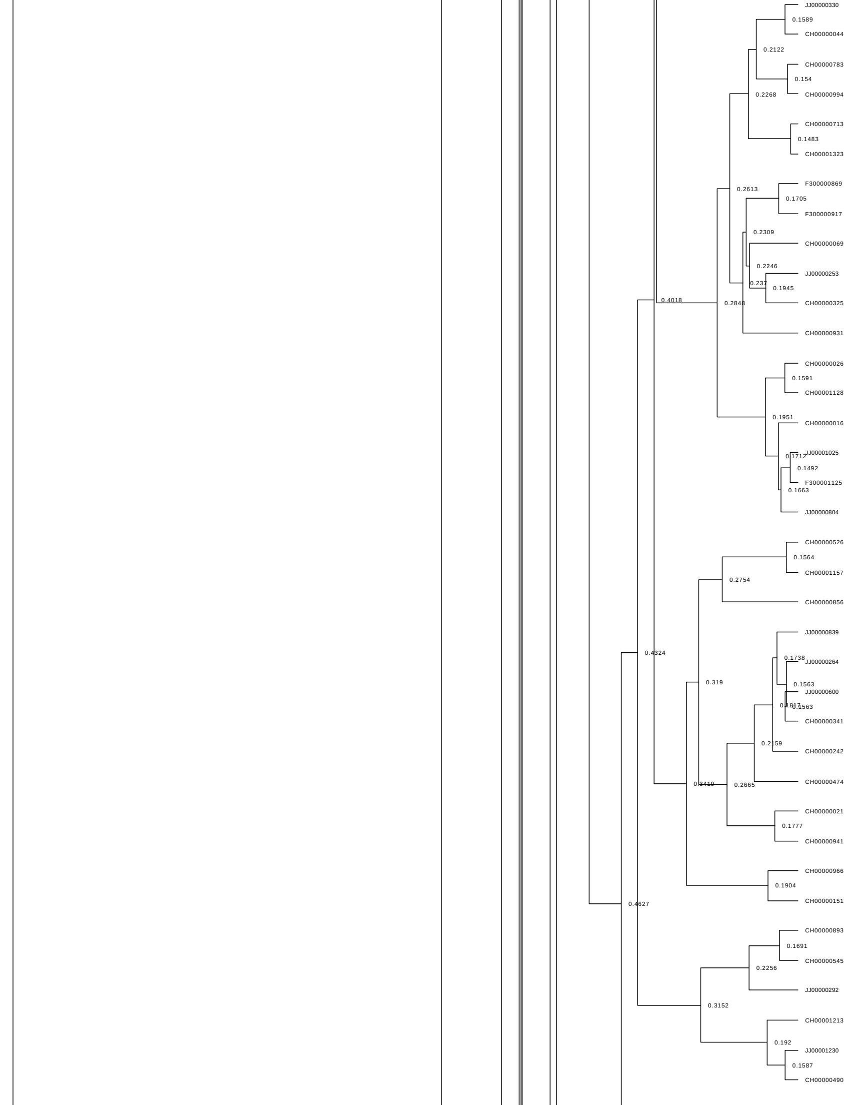
JJ00000330
0.1589
CH00000044
0.2122
CH00000783
0.154
0.2268
CH00000994
CH00000713
0.1483
CH00001323
F300000869
0.2613
0.1705
F300000917
0.2309
CH00000069
0.2246
JJ00000253
0.237
0.1945
0.4018
0.2848
CH00000325
CH00000931
CH00000026
0.1591
CH00001128
0.1951
CH00000016
JJ00001025
0.1712
0.1492
F300001125
0.1663
JJ00000804
CH00000526
0.1564
CH00001157
0.2754
CH00000856
JJ00000839
0.4324
0.1738
JJ00000264
0.319
0.1563
JJ00000600
0.1817
0.1563
CH00000341
0.2159
CH00000242
0.3419
0.2665
CH00000474
CH00000021
0.1777
CH00000941
CH00000966
0.1904
CH00000151
0.4627
CH00000893
0.1691
CH00000545
0.2256
JJ00000292
0.3152
CH00001213
0.192
JJ00001230
0.1587
CH00000490

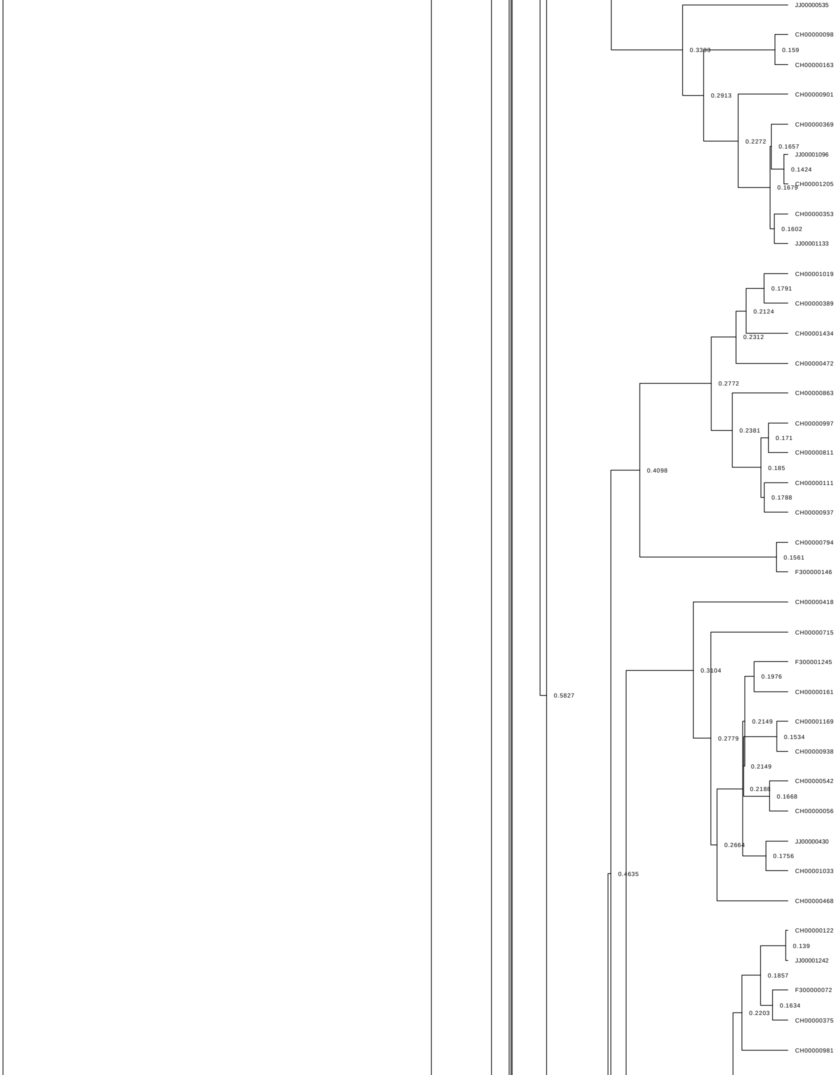

JJ00000535
CH00000098
0.3303
0.159
CH00000163
0.2913
CH00000901
CH00000369
0.2272
0.1657
JJ00001096
0.1424
CH00001205
0.1679
CH00000353
0.1602
JJ00001133
CH00001019
0.1791
CH00000389
0.2124
CH00001434
0.2312
CH00000472
0.2772
CH00000863
0.2381
CH00000997
0.171
CH00000811
0.4098
0.185
CH00000111
0.1788
CH00000937
CH00000794
0.1561
F300000146
CH00000418
CH00000715
0.3104
F300001245
0.1976
0.5827
CH00000161
0.2149
CH00001169
0.2779
0.1534
CH00000938
0.2149
CH00000542
0.2188
0.1668
CH00000056
JJ00000430
0.2664
0.1756
CH00001033
0.4635
CH00000468
CH00000122
0.139
JJ00001242
0.1857
F300000072
0.1634
0.2203
CH00000375
CH00000981

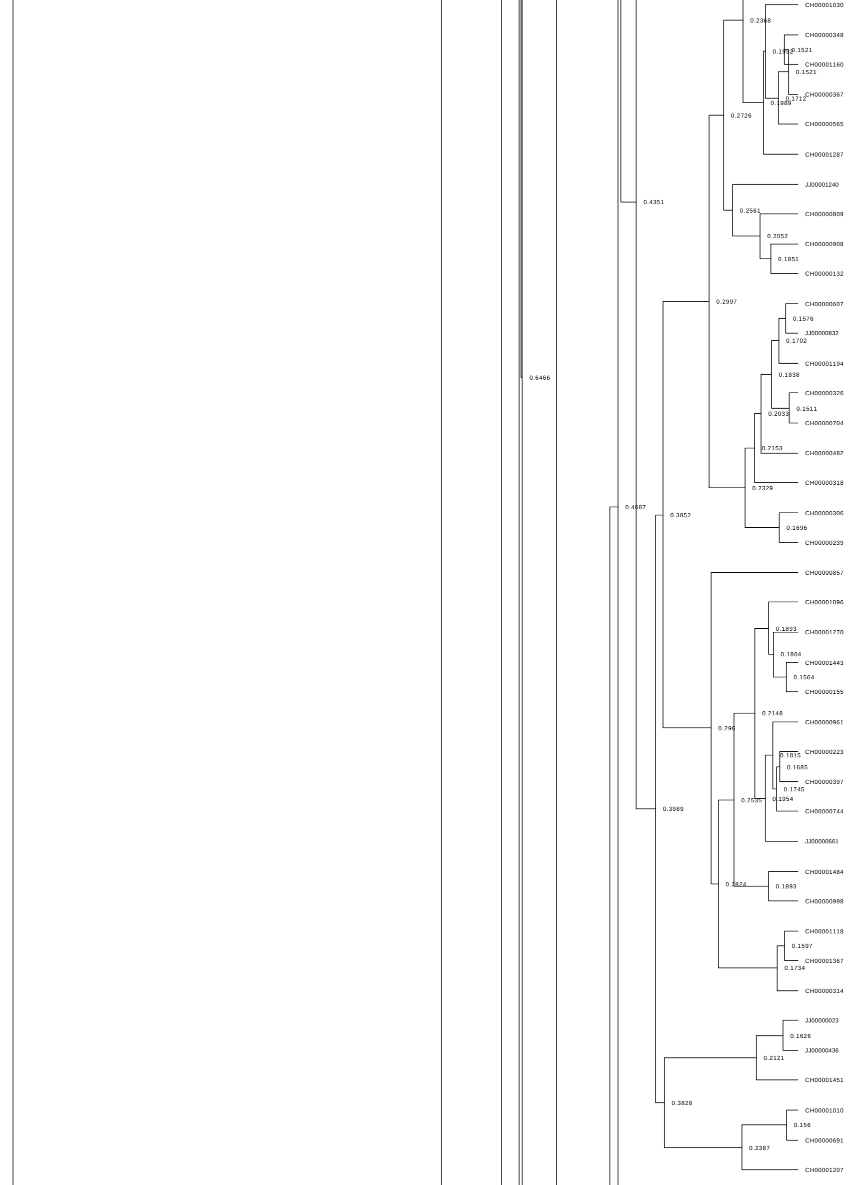

CH00001030
0.2368
CH00000348
0.1952
0.1521
CH00001160
0.1521
CH00000367
0.1712
0.1989
0.2726
CH00000565
CH00001287
JJ00001240
0.4351
0.2561
CH00000809
0.2052
CH00000908
0.1851
CH00000132
0.2997
CH00000607
0.1576
JJ00000832
0.1702
CH00001194
0.1838
0.6466
CH00000326
0.1511
0.2033
CH00000704
0.2153
CH00000482
CH00000318
0.2329
0.4687
CH00000306
0.3852
0.1696
CH00000239
CH00000857
CH00001096
0.1893
CH00001270
0.1804
CH00001443
0.1564
CH00000155
0.2148
CH00000961
0.296
CH00000223
0.1815
0.1685
CH00000397
0.1745
0.1954
0.2535
0.3989
CH00000744
JJ00000661
CH00001484
0.2824
0.1893
CH00000998
CH00001118
0.1597
CH00001367
0.1734
CH00000314
JJ00000023
0.1626
JJ00000436
0.2121
CH00001451
0.3828
CH00001010
0.156
CH00000691
0.2387
CH00001207

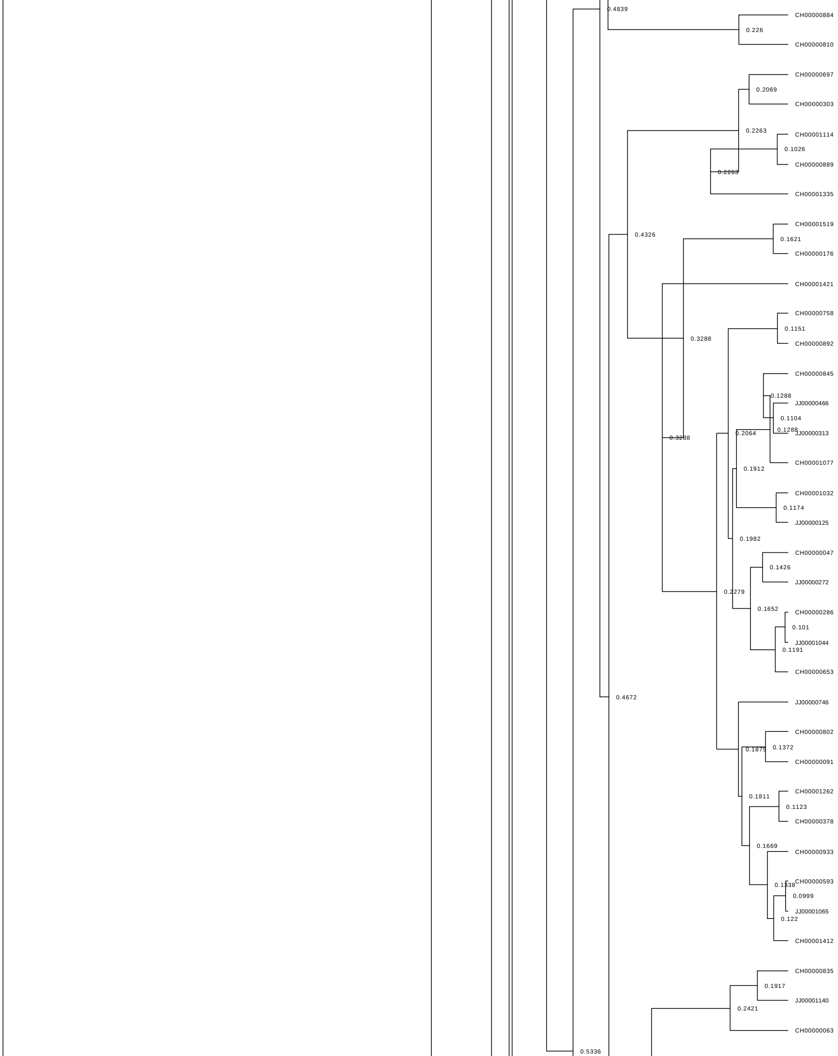

0.4839
CH00000884
0.226
CH00000810
CH00000697
0.2069
CH00000303
0.2263
CH00001114
0.1026
CH00000889
0.2263
CH00001335
CH00001519
0.4326
0.1621
CH00000176
CH00001421
CH00000758
0.1151
0.3288
CH00000892
CH00000845
0.1288
JJ00000466
0.1104
0.1288
0.2064
JJ00000313
0.3288
CH00001077
0.1912
CH00001032
0.1174
JJ00000125
0.1982
CH00000047
0.1426
JJ00000272
0.2279
0.1652
CH00000286
0.101
JJ00001044
0.1191
CH00000653
0.4672
JJ00000746
CH00000802
0.1875
0.1372
CH00000091
CH00001262
0.1811
0.1123
CH00000378
0.1669
CH00000933
CH00000593
0.1338
0.0999
JJ00001065
0.122
CH00001412
CH00000835
0.1917
JJ00001140
0.2421
CH00000063
0.5336

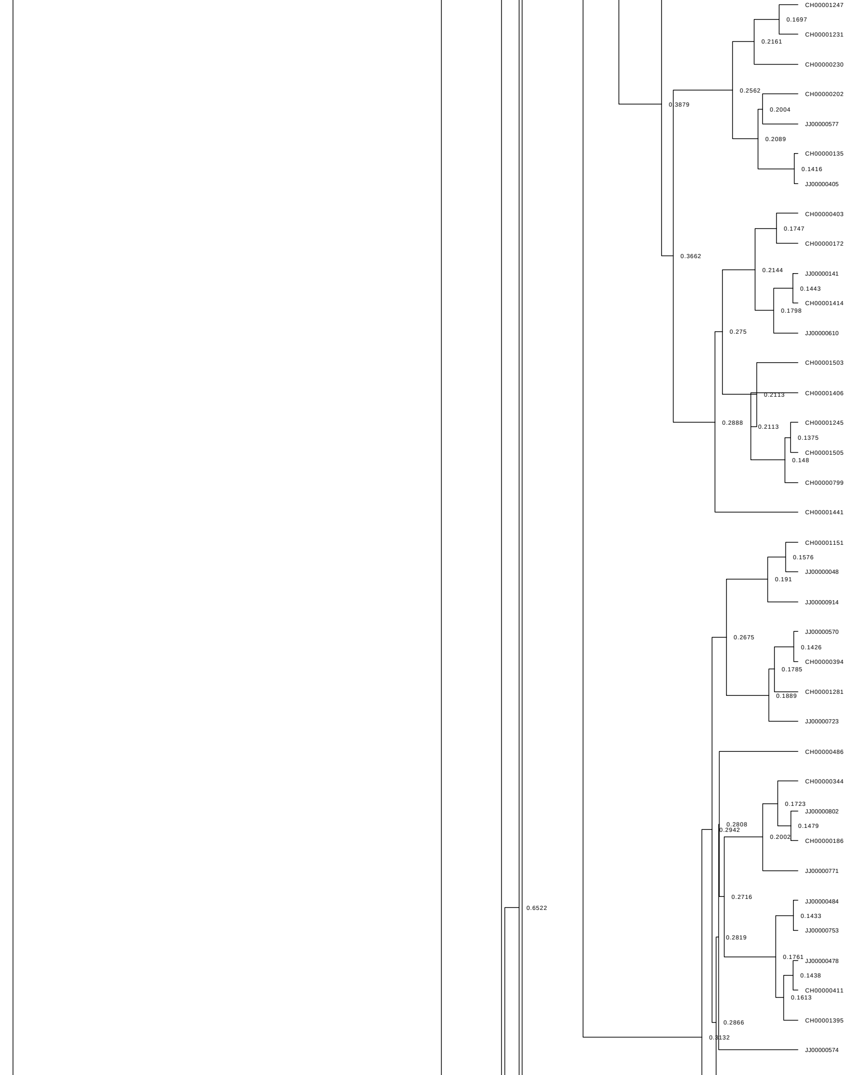
CH00001247
0.1697
CH00001231
0.2161
CH00000230
0.2562
CH00000202
0.3879
0.2004
JJ00000577
0.2089
CH00000135
0.1416
JJ00000405
CH00000403
0.1747
CH00000172
0.3662
0.2144
JJ00000141
0.1443
CH00001414
0.1798
0.275
JJ00000610
CH00001503
CH00001406
0.2113
0.2888
CH00001245
0.2113
0.1375
CH00001505
0.148
CH00000799
CH00001441
CH00001151
0.1576
JJ00000048
0.191
JJ00000914
0.2675
JJ00000570
0.1426
CH00000394
0.1785
CH00001281
0.1889
JJ00000723
CH00000486
CH00000344
0.1723
JJ00000802
0.2808
0.2942
0.1479
0.2002
CH00000186
JJ00000771
0.2716
0.6522
JJ00000484
0.1433
JJ00000753
0.2819
0.1761
JJ00000478
0.1438
CH00000411
0.1613
CH00001395
0.2866
0.3132
JJ00000574

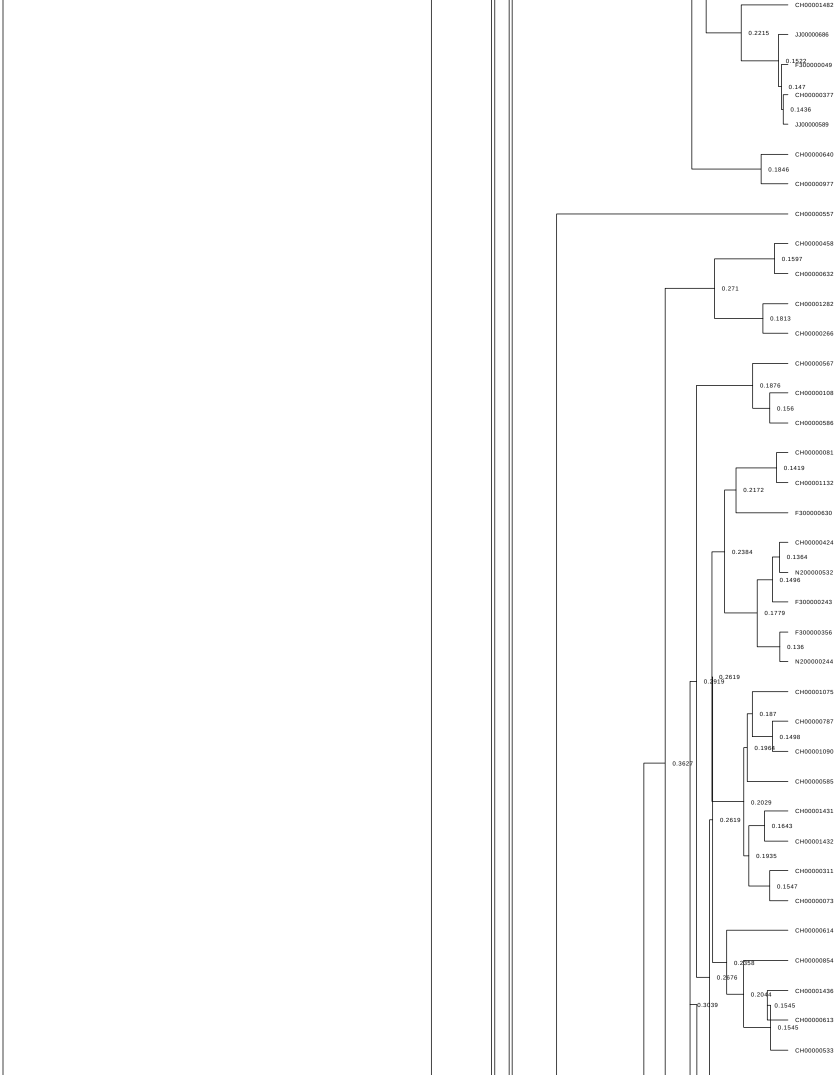
CH00001482
0.2215
JJ00000686
0.1522
F300000049
0.147
CH00000377
0.1436
JJ00000589
CH00000640
0.1846
CH00000977
CH00000557
CH00000458
0.1597
CH00000632
0.271
CH00001282
0.1813
CH00000266
CH00000567
0.1876
CH00000108
0.156
CH00000586
CH00000081
0.1419
CH00001132
0.2172
F300000630
CH00000424
0.2384
0.1364
N200000532
0.1496
F300000243
0.1779
F300000356
0.136
N200000244
0.2619
0.2919
CH00001075
0.187
CH00000787
0.1498
0.1964
CH00001090
0.3627
CH00000585
0.2029
CH00001431
0.2619
0.1643
CH00001432
0.1935
CH00000311
0.1547
CH00000073
CH00000614
CH00000854
0.2358
0.2676
CH00001436
0.2044
0.1545
0.3039
CH00000613
0.1545
CH00000533

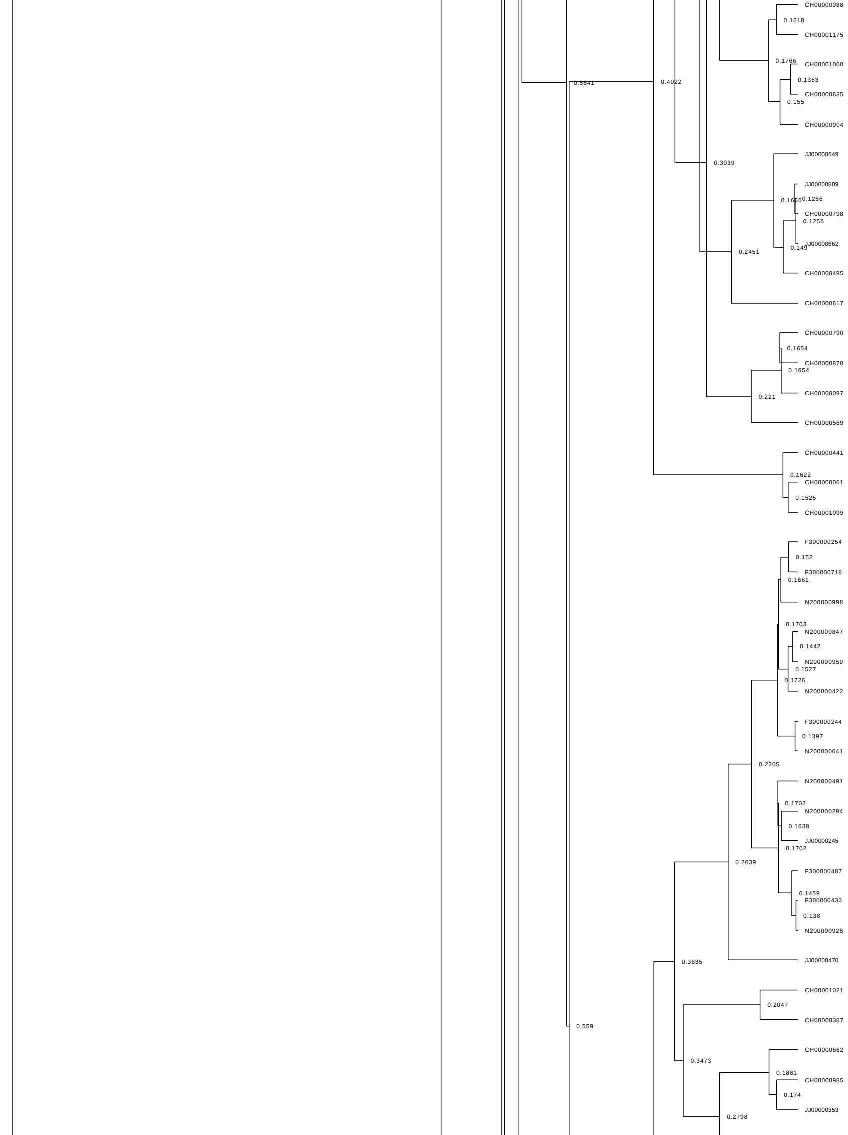

CH00000088
0.1618
CH00001175
0.1766
CH00001060
0.1353
0.5641
0.4022
CH00000635
0.155
CH00000904
JJ00000649
0.3039
JJ00000809
0.1256
0.1656
CH00000798
0.1256
JJ00000662
0.149
0.2451
CH00000495
CH00000617
CH00000790
0.1654
CH00000870
0.1654
CH00000097
0.221
CH00000569
CH00000441
0.1622
CH00000061
0.1525
CH00001099
F300000254
0.152
F300000718
0.1661
N200000998
0.1703
N200000847
0.1442
N200000959
0.1527
0.1726
N200000422
F300000244
0.1397
N200000641
0.2205
N200000491
0.1702
N200000294
0.1638
JJ00000245
0.1702
0.2639
F300000487
0.1459
F300000433
0.138
N200000928
0.3635
JJ00000470
CH00001021
0.2047
0.559
CH00000387
CH00000662
0.3473
0.1881
CH00000985
0.174
JJ00000353
0.2798

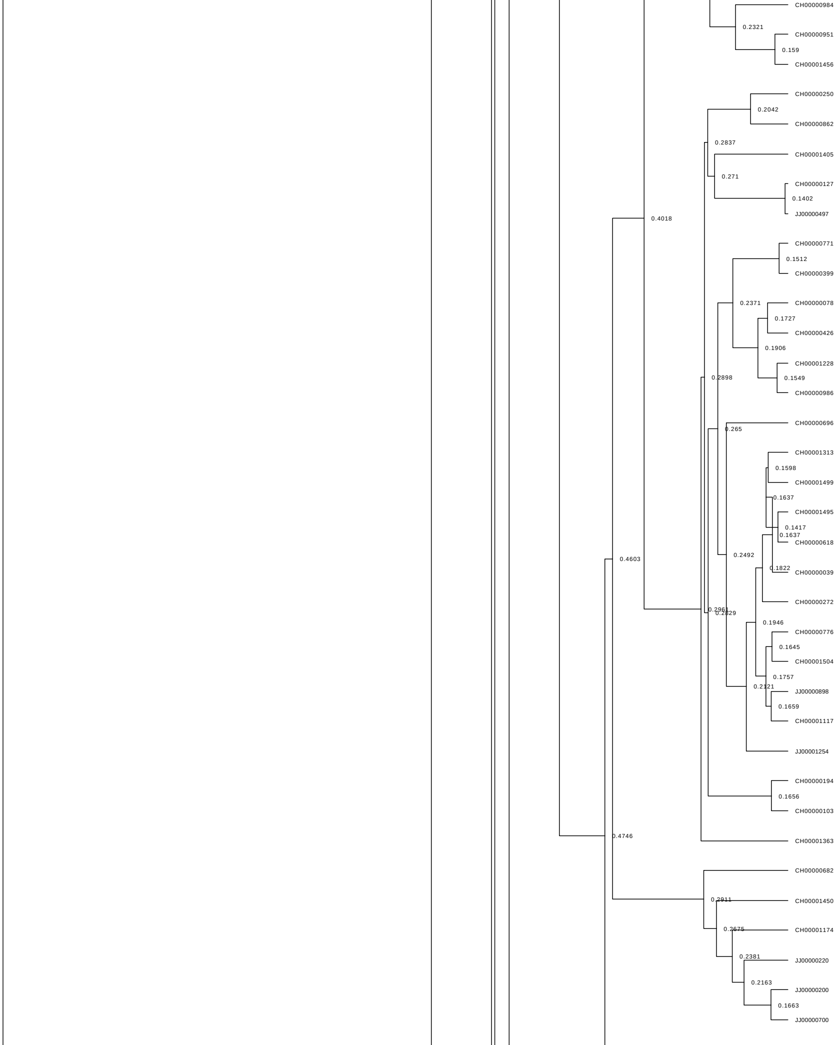
CH00000984
0.2321
CH00000951
0.159
CH00001456
CH00000250
0.2042
CH00000862
0.2837
CH00001405
0.271
CH00000127
0.1402
JJ00000497
0.4018
CH00000771
0.1512
CH00000399
0.2371
CH00000078
0.1727
CH00000426
0.1906
CH00001228
0.2898
0.1549
CH00000986
CH00000696
0.265
CH00001313
0.1598
CH00001499
0.1637
CH00001495
0.1417
0.1637
CH00000618
0.4603
0.2492
0.1822
CH00000039
CH00000272
0.2961
0.2829
0.1946
CH00000776
0.1645
CH00001504
0.1757
0.2121
JJ00000898
0.1659
CH00001117
JJ00001254
CH00000194
0.1656
CH00000103
0.4746
CH00001363
CH00000682
0.2911
CH00001450
0.2675
CH00001174
0.2381
JJ00000220
0.2163
JJ00000200
0.1663
JJ00000700

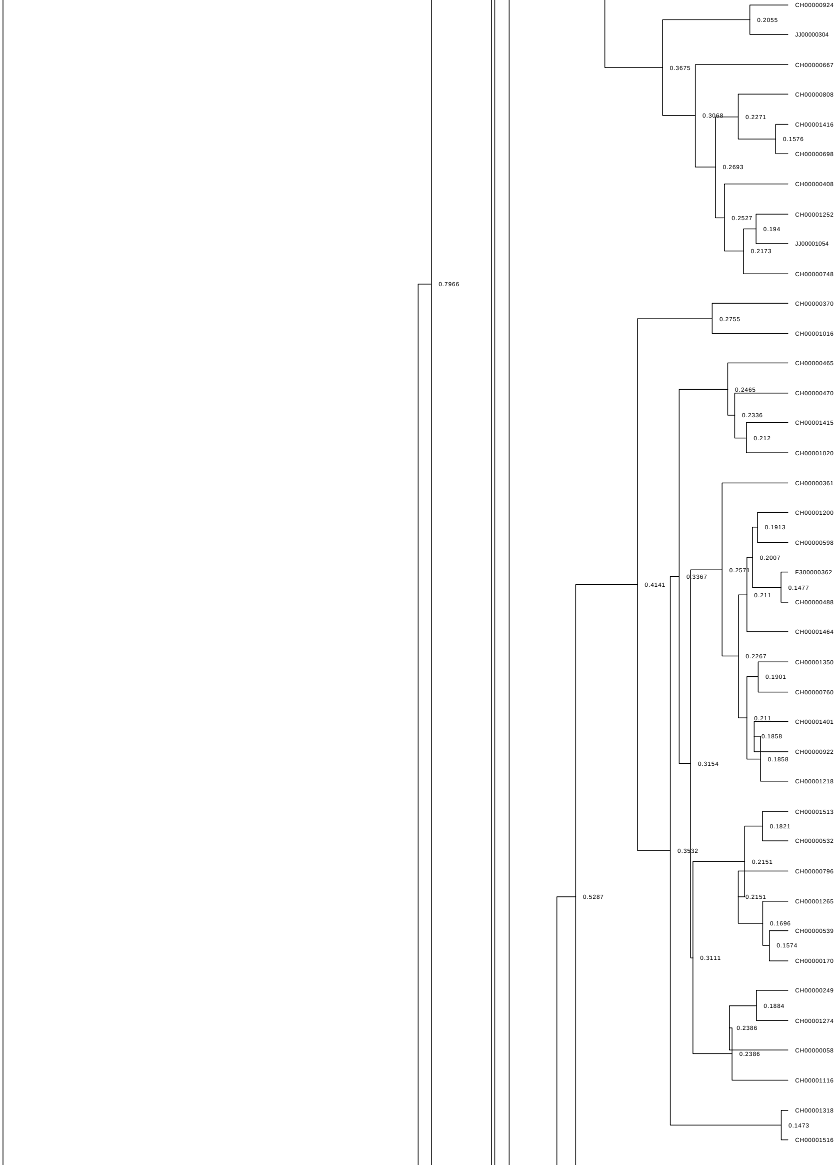

CH00000924
0.2055
JJ00000304
0.3675
CH00000667
CH00000808
0.3068
0.2271
CH00001416
0.1576
CH00000698
0.2693
CH00000408
0.2527
CH00001252
0.194
JJ00001054
0.2173
CH00000748
0.7966
CH00000370
0.2755
CH00001016
CH00000465
0.2465
CH00000470
0.2336
CH00001415
0.212
CH00001020
CH00000361
CH00001200
0.1913
CH00000598
0.2007
F300000362
0.2571
0.3367
0.1477
0.4141
0.211
CH00000488
CH00001464
0.2267
CH00001350
0.1901
CH00000760
0.211
CH00001401
0.1858
CH00000922
0.1858
0.3154
CH00001218
CH00001513
0.1821
CH00000532
0.3532
0.2151
CH00000796
0.5287
0.2151
CH00001265
0.1696
CH00000539
0.1574
0.3111
CH00000170
CH00000249
0.1884
CH00001274
0.2386
CH00000058
0.2386
CH00001116
CH00001318
0.1473
CH00001516

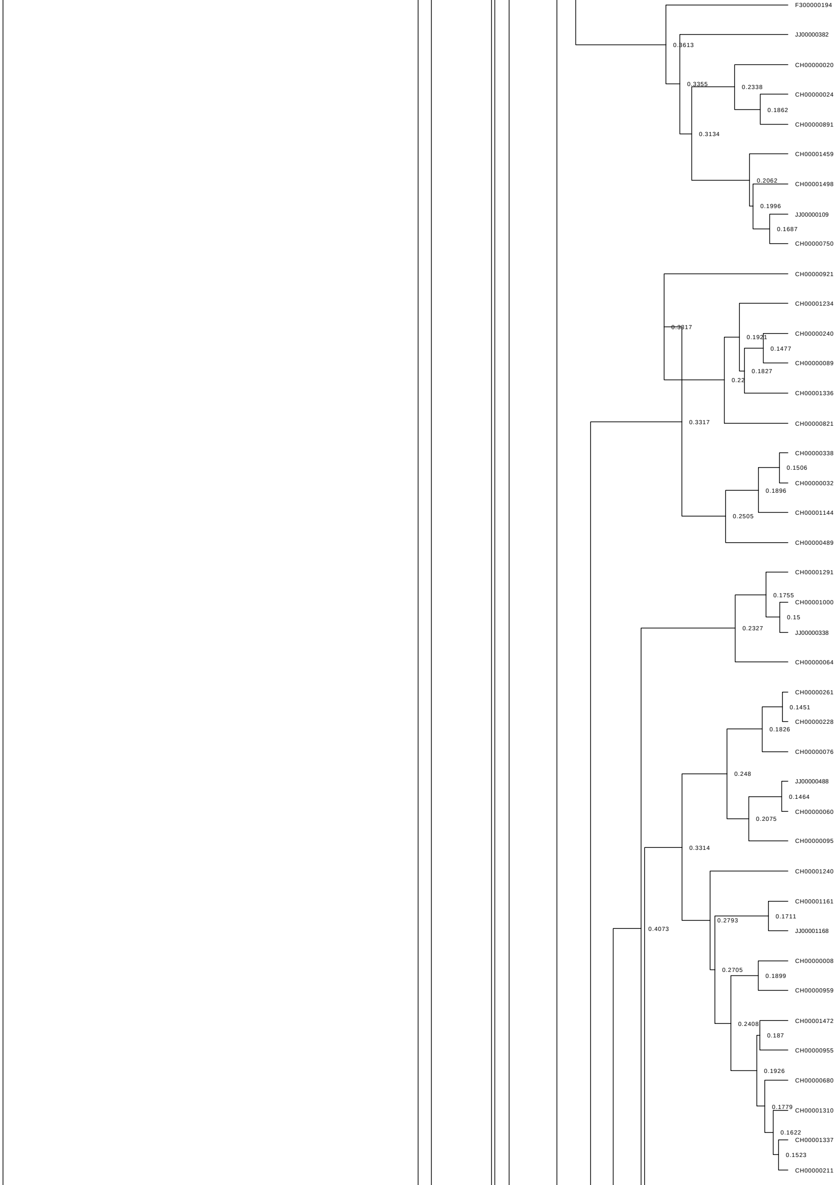
F300000194
JJ00000382
0.3613
CH00000020
0.3355
0.2338
CH00000024
0.1862
CH00000891
0.3134
CH00001459
0.2062
CH00001498
0.1996
JJ00000109
0.1687
CH00000750
CH00000921
CH00001234
0.3317
CH00000240
0.1921
0.1477
CH00000089
0.1827
0.22
CH00001336
0.3317
CH00000821
CH00000338
0.1506
CH00000032
0.1896
CH00001144
0.2505
CH00000489
CH00001291
0.1755
CH00001000
0.15
JJ00000338
0.2327
CH00000064
CH00000261
0.1451
CH00000228
0.1826
CH00000076
0.248
JJ00000488
0.1464
CH00000060
0.2075
CH00000095
0.3314
CH00001240
CH00001161
0.1711
0.2793
JJ00001168
0.4073
CH00000008
0.2705
0.1899
CH00000959
CH00001472
0.2408
0.187
CH00000955
0.1926
CH00000680
0.1779
CH00001310
0.1622
CH00001337
0.1523
CH00000211

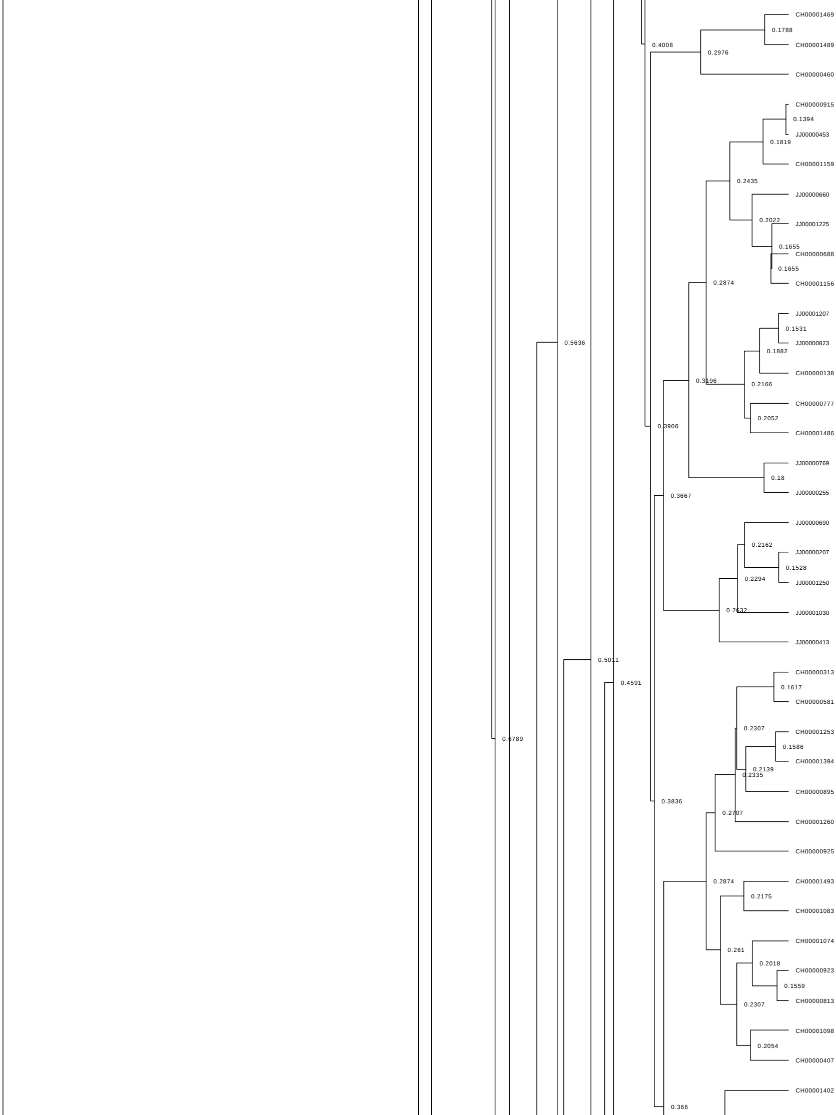
CH00001469
0.1788
0.4008
CH00001489
0.2976
CH00000460
CH00000915
0.1394
JJ00000453
0.1819
CH00001159
0.2435
JJ00000660
0.2022
JJ00001225
0.1655
CH00000688
0.1655
CH00001156
0.2874
JJ00001207
0.1531
JJ00000823
0.5636
0.1882
CH00000138
0.3196
0.2166
CH00000777
0.2052
0.3906
CH00001486
JJ00000769
0.18
JJ00000255
0.3667
JJ00000690
0.2162
JJ00000207
0.1528
0.2294
JJ00001250
0.2632
JJ00001030
JJ00000413
0.5011
CH00000313
0.4591
0.1617
CH00000581
0.2307
CH00001253
0.6789
0.1586
CH00001394
0.2139
0.2335
CH00000895
0.3836
0.2707
CH00001260
CH00000925
0.2874
CH00001493
0.2175
CH00001083
CH00001074
0.261
0.2018
CH00000923
0.1559
CH00000813
0.2307
CH00001098
0.2054
CH00000407
CH00001402
0.366

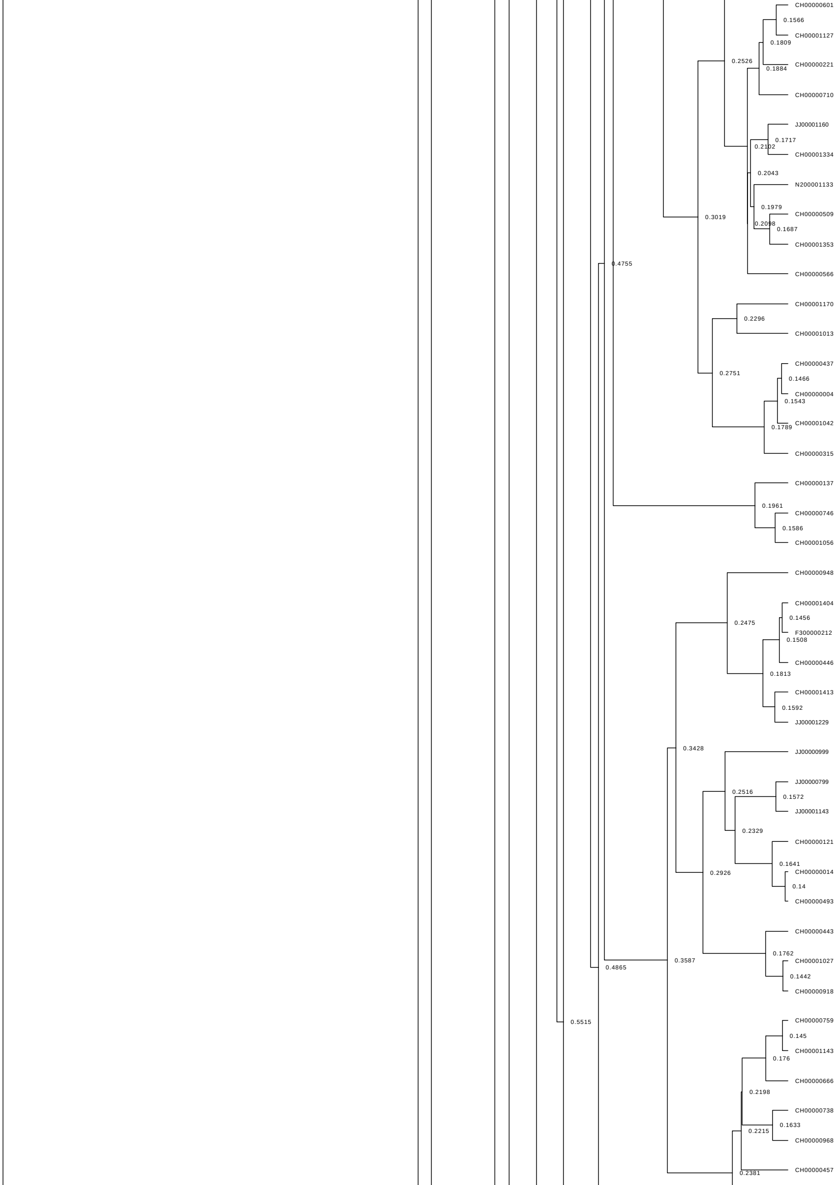
CH00000601
0.1566
CH00001127
0.1809
0.2526
CH00000221
0.1884
CH00000710
JJ00001160
0.1717
0.2102
CH00001334
0.2043
N200001133
0.1979
CH00000509
0.2098
0.1687
CH00001353
0.3019
0.4755
CH00000566
CH00001170
0.2296
CH00001013
CH00000437
0.1466
0.2751
CH00000004
0.1543
CH00001042
0.1789
CH00000315
CH00000137
0.1961
CH00000746
0.1586
CH00001056
CH00000948
CH00001404
0.1456
0.2475
F300000212
0.1508
CH00000446
0.1813
CH00001413
0.1592
JJ00001229
JJ00000999
0.3428
JJ00000799
0.2516
0.1572
JJ00001143
0.2329
CH00000121
0.1641
CH00000014
0.2926
0.14
CH00000493
CH00000443
0.1762
CH00001027
0.3587
0.4865
0.1442
CH00000918
0.5515
CH00000759
0.145
CH00001143
0.176
CH00000666
0.2198
CH00000738
0.1633
0.2215
CH00000968
CH00000457
0.2381

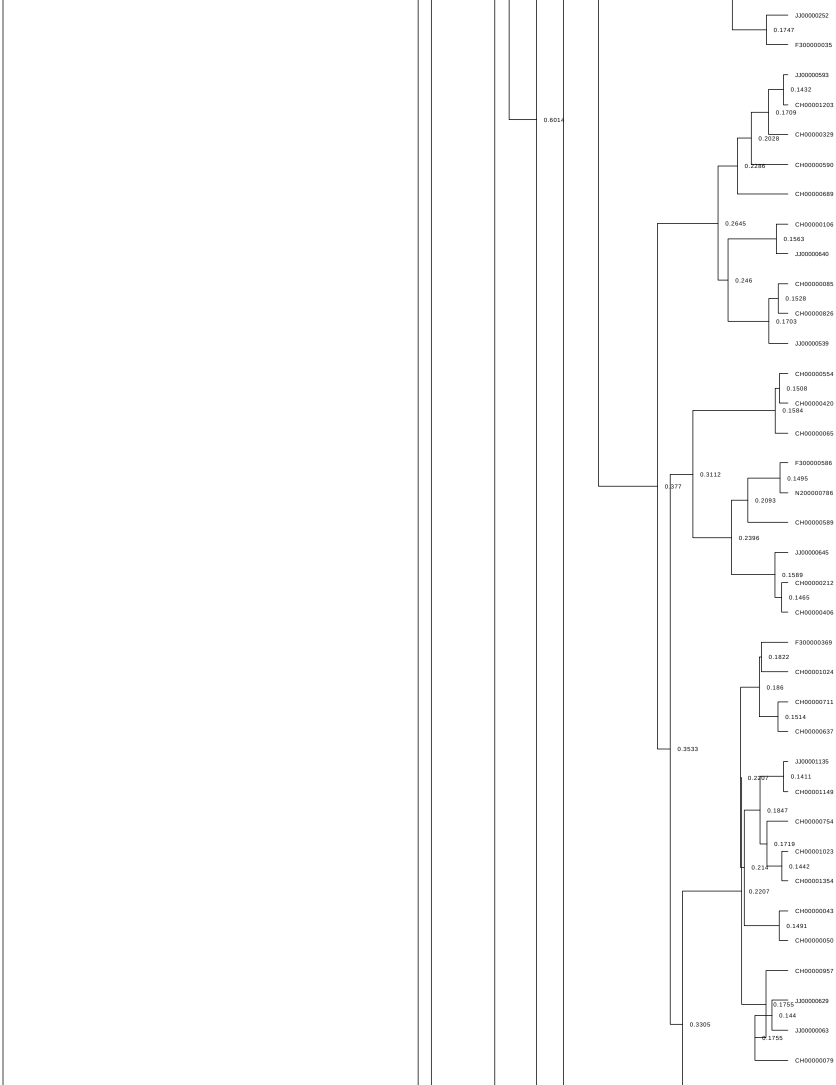
JJ00000252
0.1747
F300000035
JJ00000593
0.1432
CH00001203
0.1709
0.6014
CH00000329
0.2028
CH00000590
0.2286
CH00000689
0.2645
CH00000106
0.1563
JJ00000640
0.246
CH00000085
0.1528
CH00000826
0.1703
JJ00000539
CH00000554
0.1508
CH00000420
0.1584
CH00000065
F300000586
0.3112
0.1495
0.377
N200000786
0.2093
CH00000589
0.2396
JJ00000645
0.1589
CH00000212
0.1465
CH00000406
F300000369
0.1822
CH00001024
0.186
CH00000711
0.1514
CH00000637
0.3533
JJ00001135
0.2207
0.1411
CH00001149
0.1847
CH00000754
0.1719
CH00001023
0.1442
0.214
CH00001354
0.2207
CH00000043
0.1491
CH00000050
CH00000957
0.1755
JJ00000629
0.144
0.3305
JJ00000063
0.1755
CH00000079

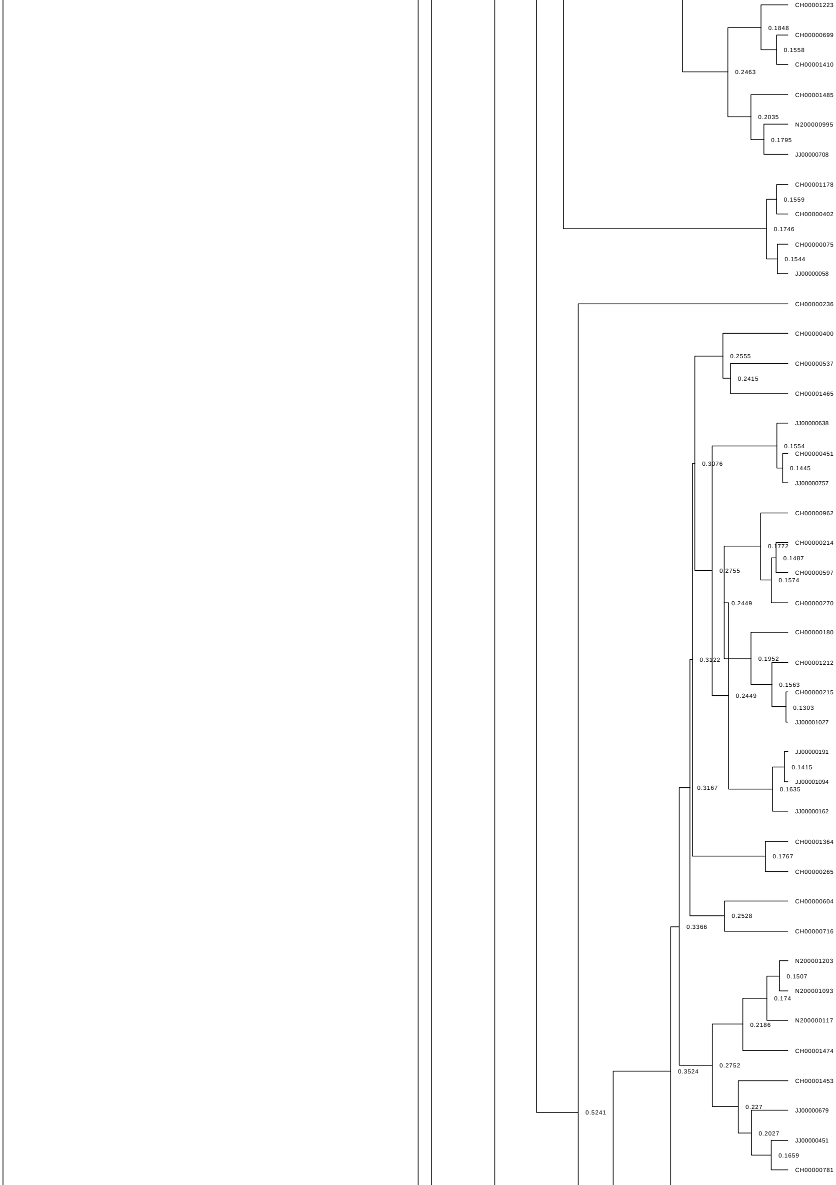

CH00001223
0.1848
CH00000699
0.1558
CH00001410
0.2463
CH00001485
0.2035
N200000995
0.1795
JJ00000708
CH00001178
0.1559
CH00000402
0.1746
CH00000075
0.1544
JJ00000058
CH00000236
CH00000400
0.2555
CH00000537
0.2415
CH00001465
JJ00000638
0.1554
CH00000451
0.3076
0.1445
JJ00000757
CH00000962
CH00000214
0.1772
0.1487
0.2755
CH00000597
0.1574
0.2449
CH00000270
CH00000180
0.1952
0.3122
CH00001212
0.1563
CH00000215
0.2449
0.1303
JJ00001027
JJ00000191
0.1415
JJ00001094
0.3167
0.1635
JJ00000162
CH00001364
0.1767
CH00000265
CH00000604
0.2528
0.3366
CH00000716
N200001203
0.1507
N200001093
0.174
N200000117
0.2186
CH00001474
0.2752
0.3524
CH00001453
0.227
JJ00000679
0.5241
0.2027
JJ00000451
0.1659
CH00000781

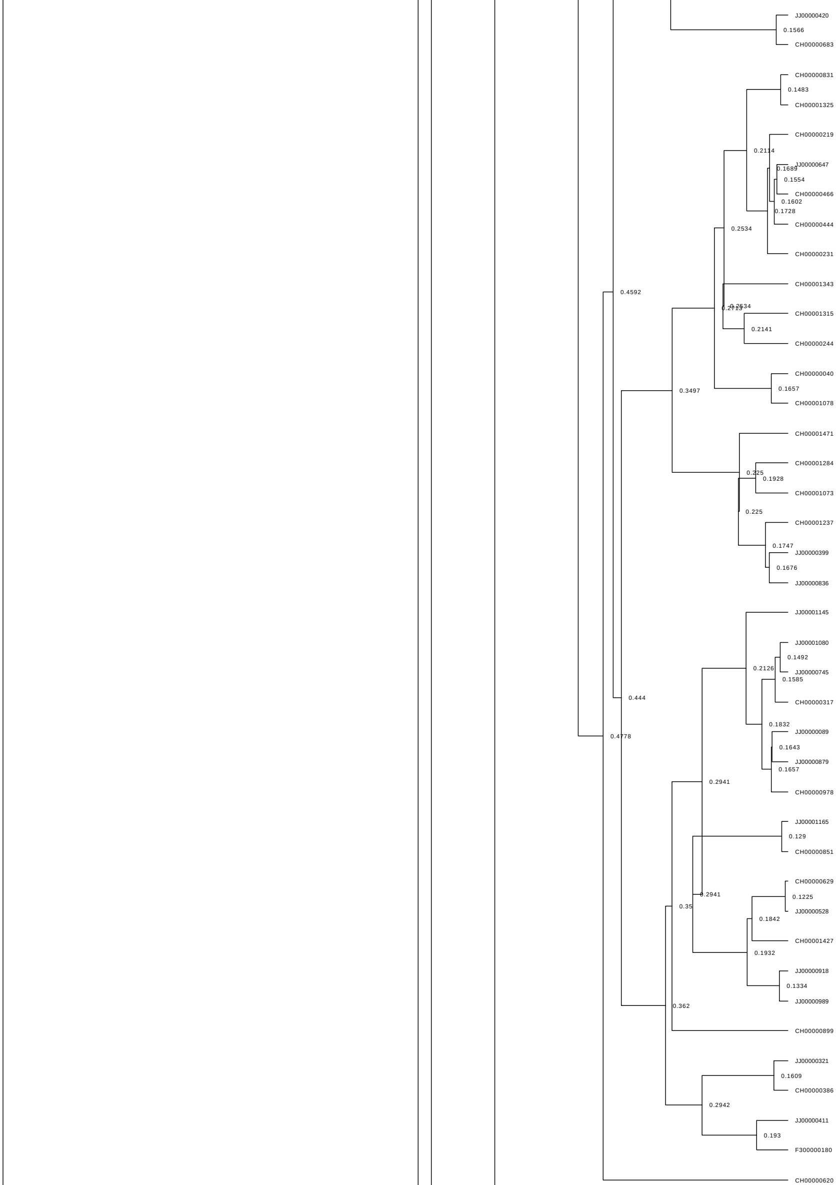
JJ00000420
0.1566
CH00000683
CH00000831
0.1483
CH00001325
CH00000219
0.2114
JJ00000647
0.1689
0.1554
CH00000466
0.1602
0.1728
CH00000444
0.2534
CH00000231
CH00001343
0.4592
0.2534
0.2713
CH00001315
0.2141
CH00000244
CH00000040
0.1657
0.3497
CH00001078
CH00001471
CH00001284
0.225
0.1928
CH00001073
0.225
CH00001237
0.1747
JJ00000399
0.1676
JJ00000836
JJ00001145
JJ00001080
0.1492
0.2126
JJ00000745
0.1585
0.444
CH00000317
0.1832
JJ00000089
0.4778
0.1643
JJ00000879
0.1657
0.2941
CH00000978
JJ00001165
0.129
CH00000851
CH00000629
0.1225
0.2941
JJ00000528
0.35
0.1842
CH00001427
0.1932
JJ00000918
0.1334
JJ00000989
0.362
CH00000899
JJ00000321
0.1609
CH00000386
0.2942
JJ00000411
0.193
F300000180
CH00000620

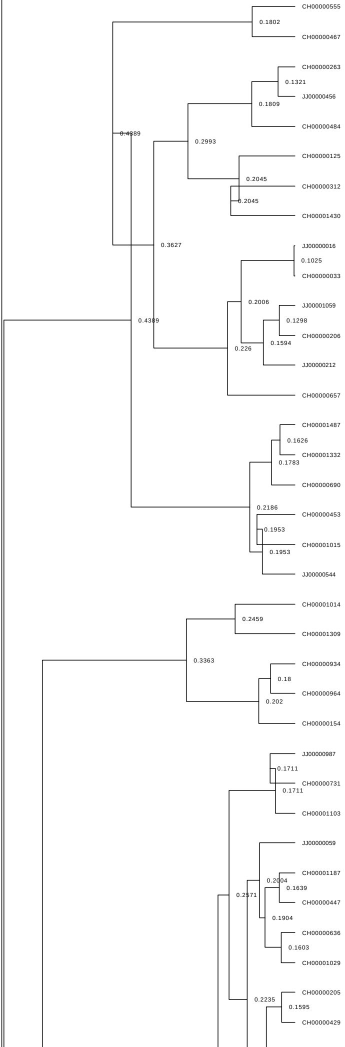
CH00000555
0.1802
CH00000467
CH00000263
0.1321
JJ00000456
0.1809
CH00000484
0.4389
0.2993
CH00000125
0.2045
CH00000312
0.2045
CH00001430
0.3627
JJ00000016
0.1025
CH00000033
0.2006
JJ00001059
0.4389
0.1298
CH00000206
0.1594
0.226
JJ00000212
CH00000657
CH00001487
0.1626
CH00001332
0.1783
CH00000690
0.2186
CH00000453
0.1953
CH00001015
0.1953
JJ00000544
CH00001014
0.2459
CH00001309
0.3363
CH00000934
0.18
CH00000964
0.202
CH00000154
JJ00000987
0.1711
CH00000731
0.1711
CH00001103
JJ00000059
CH00001187
0.2004
0.1639
0.2571
CH00000447
0.1904
CH00000636
0.1603
CH00001029
CH00000205
0.2235
0.1595
CH00000429

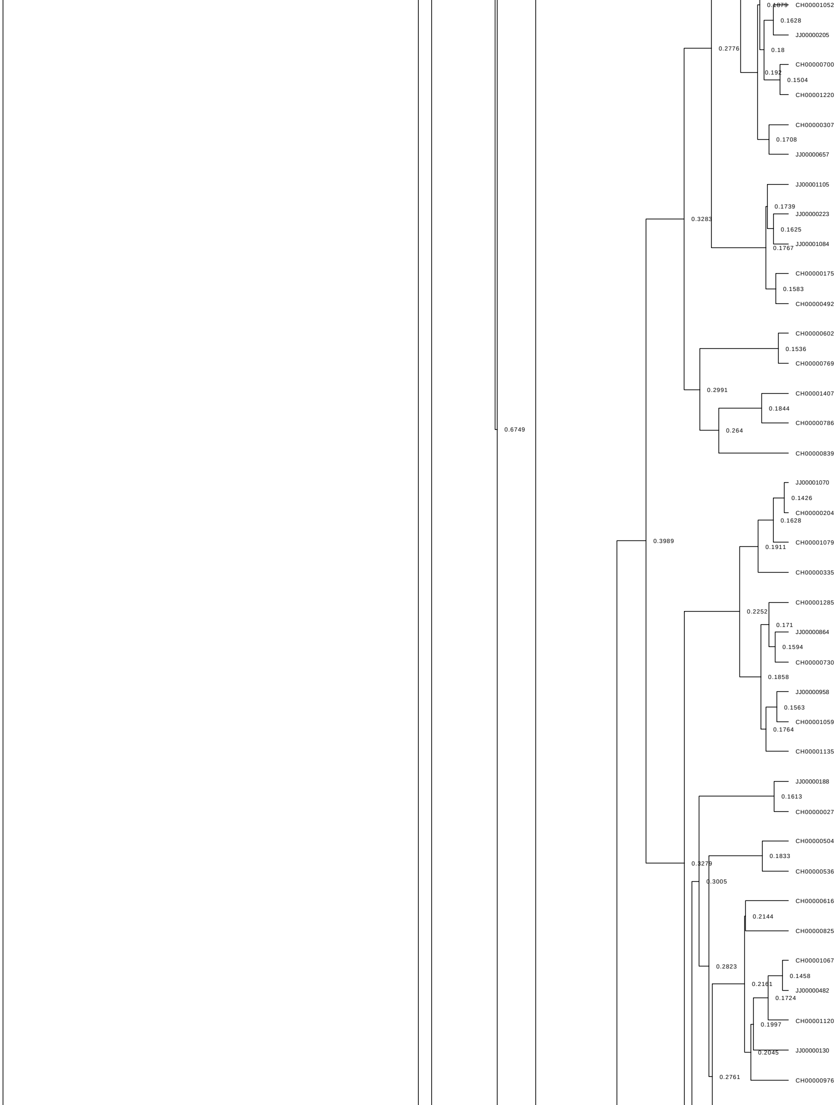

0.1879
CH00001052
0.1628
JJ00000205
0.2776
0.18
CH00000700
0.192
0.1504
CH00001220
CH00000307
0.1708
JJ00000657
JJ00001105
0.1739
JJ00000223
0.3283
0.1625
JJ00001084
0.1767
CH00000175
0.1583
CH00000492
CH00000602
0.1536
CH00000769
0.2991
CH00001407
0.1844
0.6749
0.264
CH00000786
CH00000839
JJ00001070
0.1426
CH00000204
0.1628
0.3989
CH00001079
0.1911
CH00000335
CH00001285
0.2252
0.171
JJ00000864
0.1594
CH00000730
0.1858
JJ00000958
0.1563
CH00001059
0.1764
CH00001135
JJ00000188
0.1613
CH00000027
CH00000504
0.1833
0.3279
CH00000536
0.3005
CH00000616
0.2144
CH00000825
CH00001067
0.2823
0.1458
0.2161
JJ00000482
0.1724
CH00001120
0.1997
JJ00000130
0.2045
0.2761
CH00000976

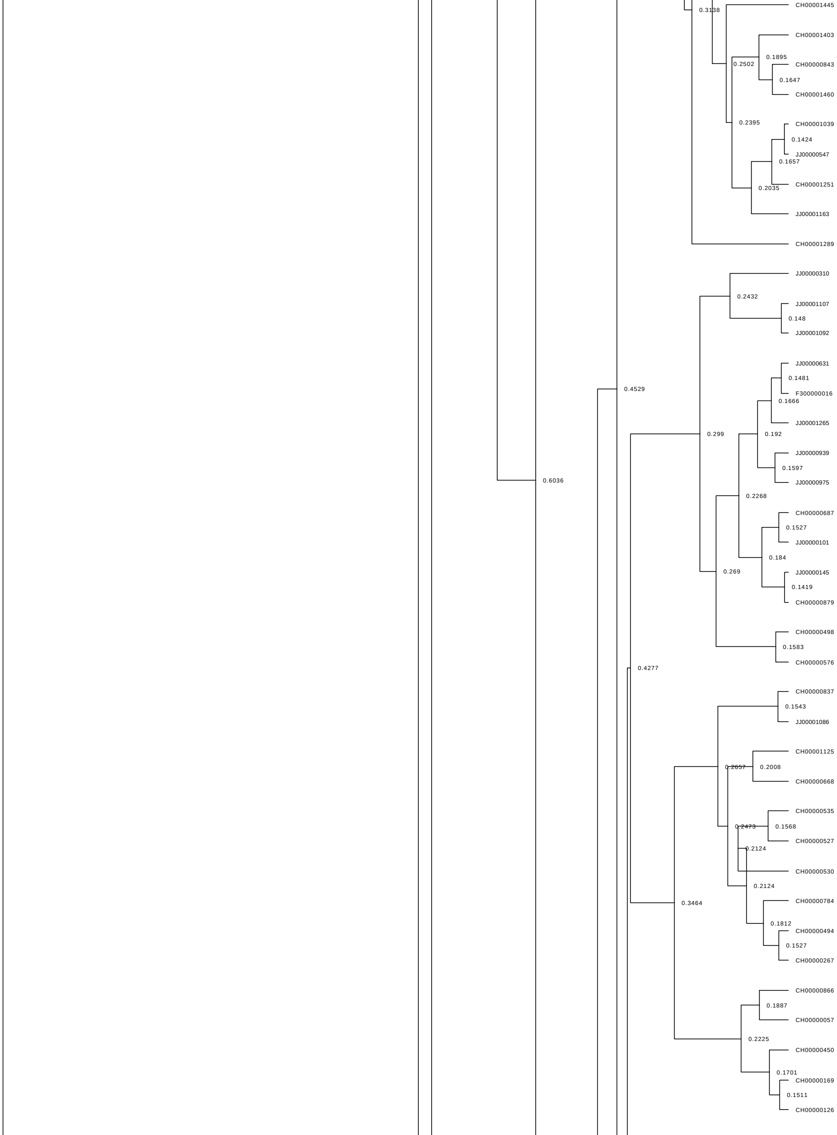

CH00001445
0.3138
CH00001403
0.1895
0.2502
CH00000843
0.1647
CH00001460
0.2395
CH00001039
0.1424
JJ00000547
0.1657
CH00001251
0.2035
JJ00001163
CH00001289
JJ00000310
0.2432
JJ00001107
0.148
JJ00001092
JJ00000631
0.1481
0.4529
F300000016
0.1666
JJ00001265
0.299
0.192
JJ00000939
0.1597
0.6036
JJ00000975
0.2268
CH00000687
0.1527
JJ00000101
0.184
JJ00000145
0.269
0.1419
CH00000879
CH00000498
0.1583
CH00000576
0.4277
CH00000837
0.1543
JJ00001086
CH00001125
0.2657
0.2008
CH00000668
CH00000535
0.2473
0.1568
CH00000527
0.2124
CH00000530
0.2124
0.3464
CH00000784
0.1812
CH00000494
0.1527
CH00000267
CH00000866
0.1887
CH00000057
0.2225
CH00000450
0.1701
CH00000169
0.1511
CH00000126

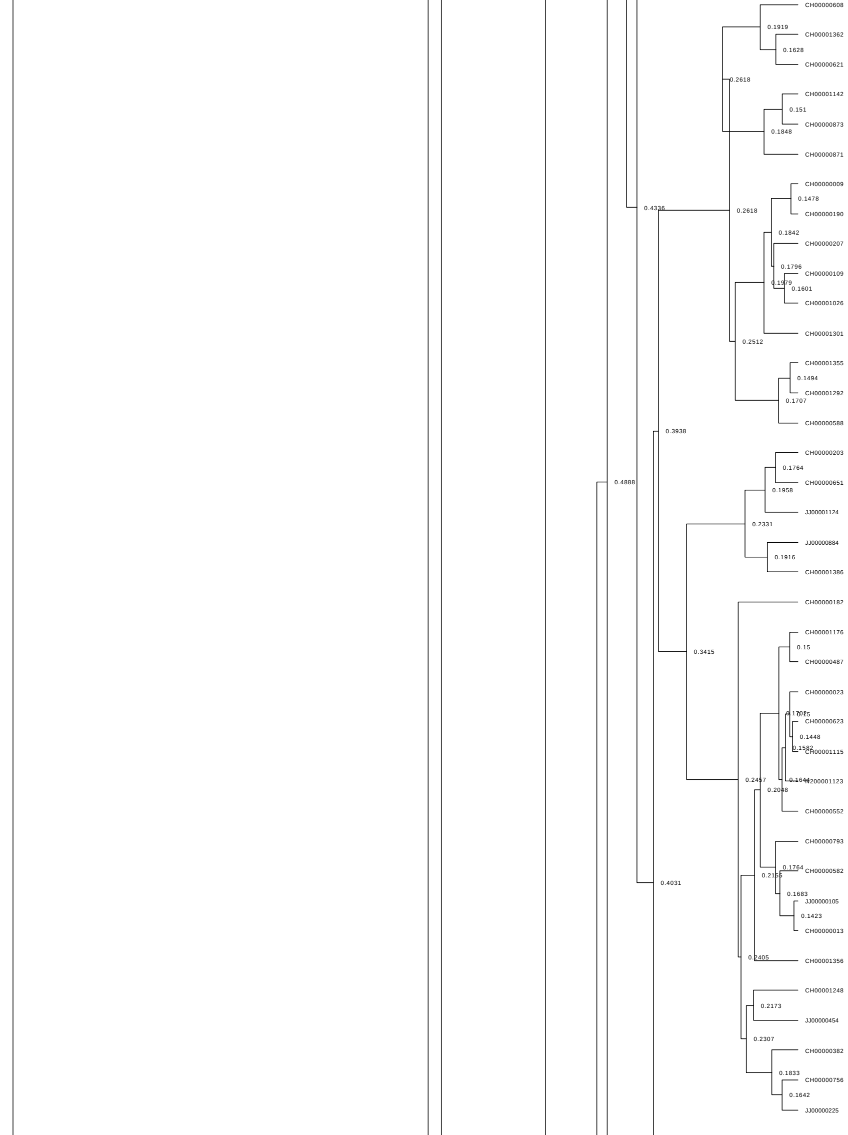
CH00000608
0.1919
CH00001362
0.1628
CH00000621
0.2618
CH00001142
0.151
CH00000873
0.1848
CH00000871
CH00000009
0.1478
0.4336
0.2618
CH00000190
0.1842
CH00000207
0.1796
CH00000109
0.1979
0.1601
CH00001026
CH00001301
0.2512
CH00001355
0.1494
CH00001292
0.1707
CH00000588
0.3938
CH00000203
0.1764
0.4888
CH00000651
0.1958
JJ00001124
0.2331
JJ00000884
0.1916
CH00001386
CH00000182
CH00001176
0.15
0.3415
CH00000487
CH00000023
0.1702
0.15
CH00000623
0.1448
0.1582
CH00001115
0.2457
0.1644
N200001123
0.2048
CH00000552
CH00000793
0.1764
0.2156
CH00000582
0.4031
0.1683
JJ00000105
0.1423
CH00000013
0.2405
CH00001356
CH00001248
0.2173
JJ00000454
0.2307
CH00000382
0.1833
CH00000756
0.1642
JJ00000225

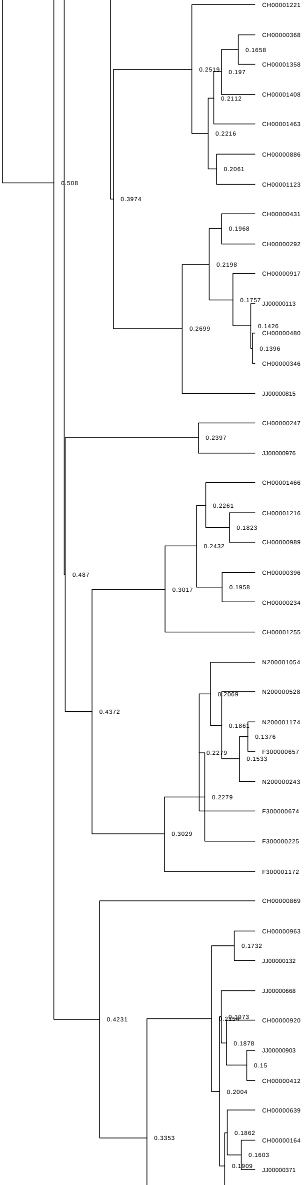

CH00001221
CH00000368
0.1658
CH00001358
0.2519
0.197
CH00001408
0.2112
CH00001463
0.2216
CH00000886
0.2061
CH00001123
0.508
0.3974
CH00000431
0.1968
CH00000292
0.2198
CH00000917
0.1757
JJ00000113
0.1426
CH00000480
0.1396
CH00000346
0.2699
JJ00000815
CH00000247
0.2397
JJ00000976
CH00001466
0.2261
CH00001216
0.1823
CH00000989
0.2432
0.487
CH00000396
0.1958
CH00000234
0.3017
CH00001255
N200001054
N200000528
0.2069
N200001174
0.1861
0.1376
F300000657
0.2279
0.1533
N200000243
0.2279
F300000674
F300000225
0.3029
F300001172
0.4372
CH00000869
CH00000963
0.1732
JJ00000132
JJ00000668
0.1073
CH00000920
0.1878
JJ00000903
0.15
CH00000412
0.2004
0.4231
CH00000639
0.1862
CH00000164
0.1603
0.1909
JJ00000371
0.3353

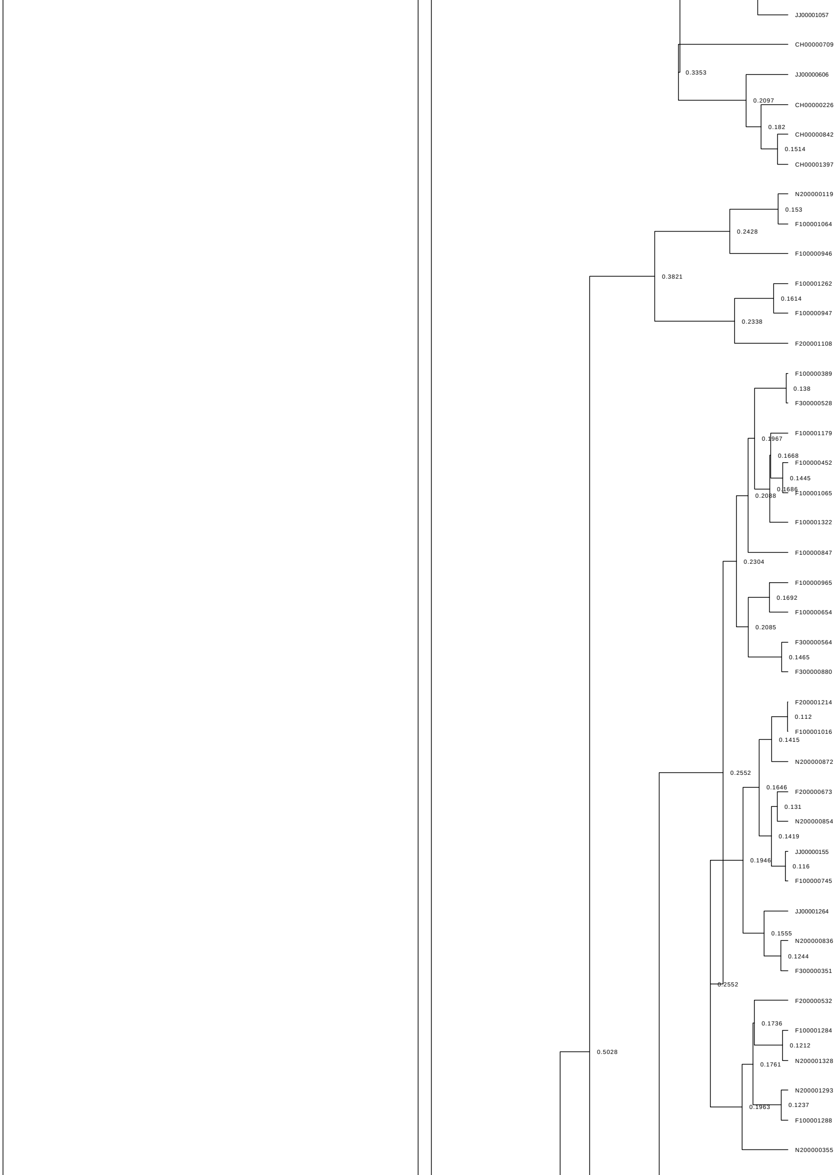

JJ00001057
CH00000709
0.3353
JJ00000606
0.2097
CH00000226
0.182
CH00000842
0.1514
CH00001397
N200000119
0.153
F100001064
0.2428
F100000946
0.3821
F100001262
0.1614
F100000947
0.2338
F200001108
F100000389
0.138
F300000528
F100001179
0.1967
0.1668
F100000452
0.1445
0.1686
F100001065
0.2088
F100001322
F100000847
0.2304
F100000965
0.1692
F100000654
0.2085
F300000564
0.1465
F300000880
F200001214
0.112
F100001016
0.1415
N200000872
0.2552
0.1646
F200000673
0.131
N200000854
0.1419
JJ00000155
0.1946
0.116
F100000745
JJ00001264
0.1555
N200000836
0.1244
F300000351
0.2552
F200000532
0.1736
F100001284
0.1212
0.5028
N200001328
0.1761
N200001293
0.1237
0.1963
F100001288
N200000355

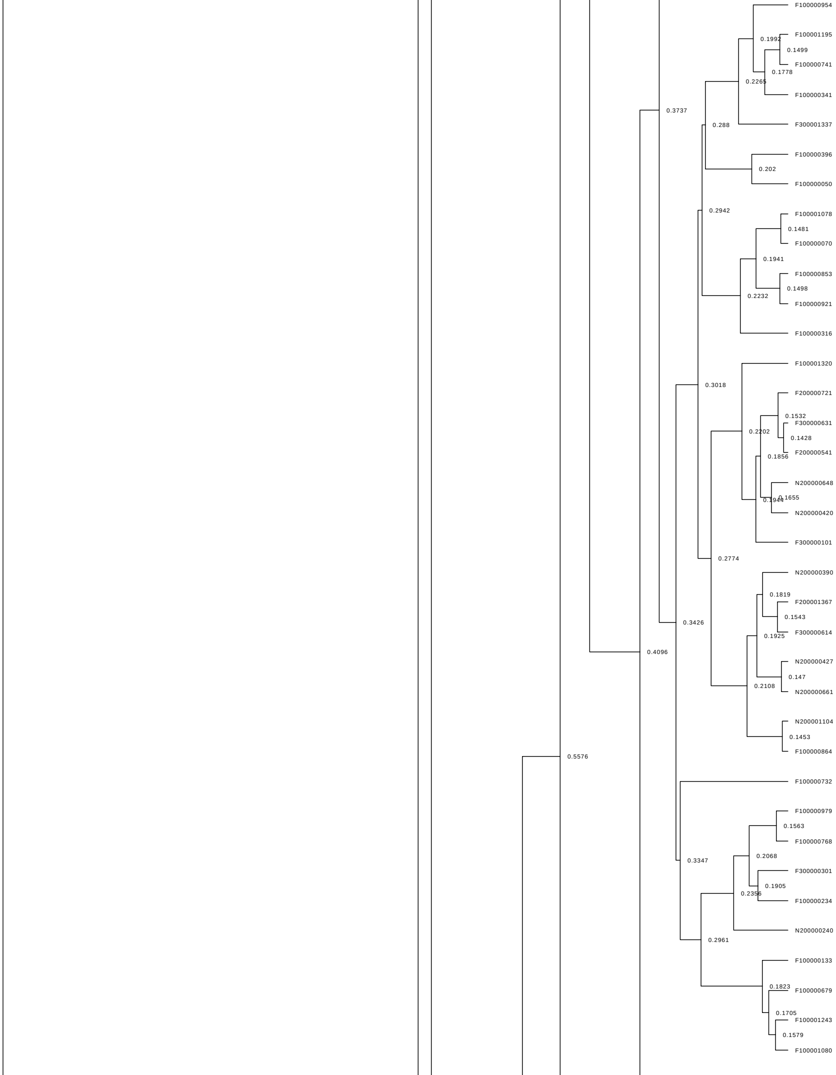
F100000954
F100001195
0.1992
0.1499
F100000741
0.1778
0.2265
F100000341
0.3737
F300001337
0.288
F100000396
0.202
F100000050
0.2942
F100001078
0.1481
F100000070
0.1941
F100000853
0.1498
0.2232
F100000921
F100000316
F100001320
0.3018
F200000721
0.1532
F300000631
0.2202
0.1428
F200000541
0.1856
N200000648
0.1944
0.1655
N200000420
F300000101
0.2774
N200000390
0.1819
F200001367
0.1543
0.3426
F300000614
0.1925
0.4096
N200000427
0.147
0.2108
N200000661
N200001104
0.1453
F100000864
0.5576
F100000732
F100000979
0.1563
F100000768
0.2068
0.3347
F300000301
0.1905
0.2356
F100000234
N200000240
0.2961
F100000133
0.1823
F100000679
0.1705
F100001243
0.1579
F100001080

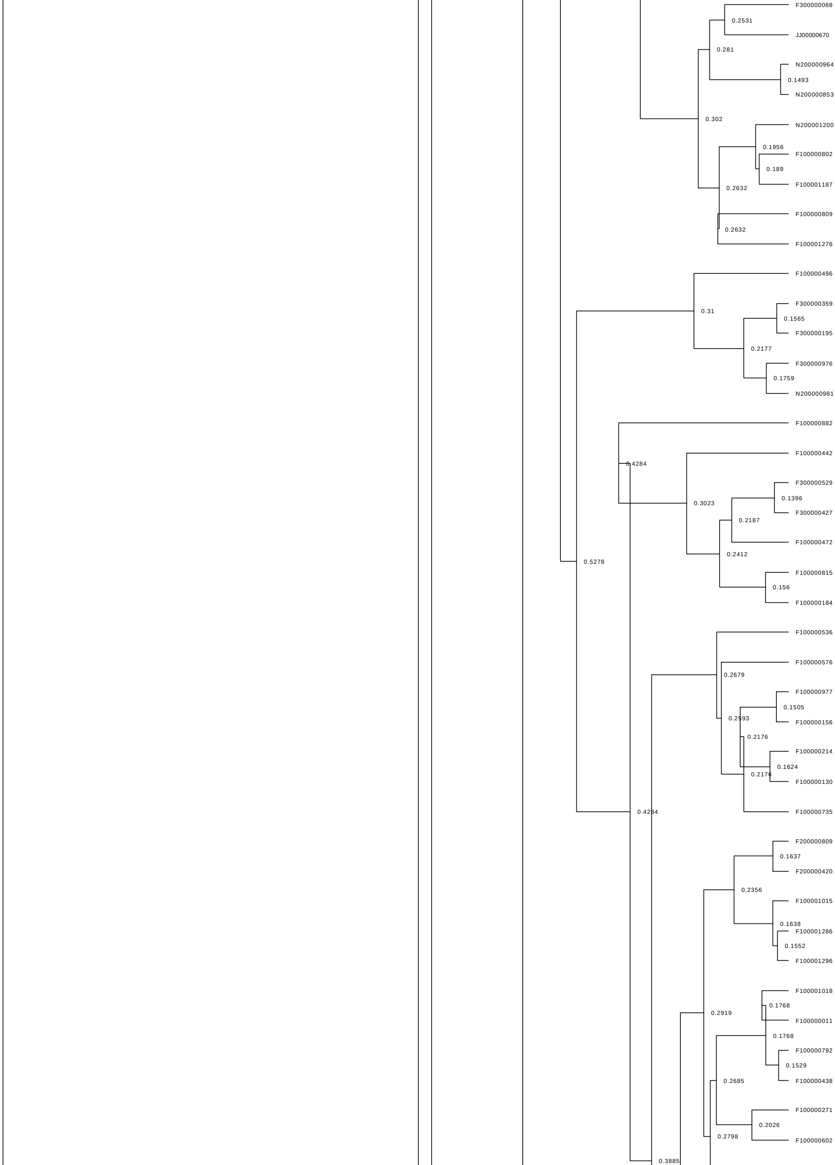
F300000068
0.2531
JJ00000670
0.281
N200000964
0.1493
N200000853
0.302
N200001200
0.1956
F100000802
0.189
F100001187
0.2632
F100000809
0.2632
F100001276
F100000496
F300000359
0.31
0.1565
F300000195
0.2177
F300000976
0.1759
N200000981
F100000882
F100000442
0.4284
F300000529
0.1396
0.3023
F300000427
0.2187
F100000472
0.2412
0.5278
F100000815
0.156
F100000184
F100000536
F100000576
0.2679
F100000977
0.1505
0.2593
F100000156
0.2176
F100000214
0.1624
0.2176
F100000130
0.4284
F100000735
F200000809
0.1637
F200000420
0.2356
F100001015
0.1638
F100001286
0.1552
F100001296
F100001018
0.1768
0.2919
F100000011
0.1768
F100000792
0.1529
F100000438
0.2685
F100000271
0.2026
0.2798
F100000602
0.3885

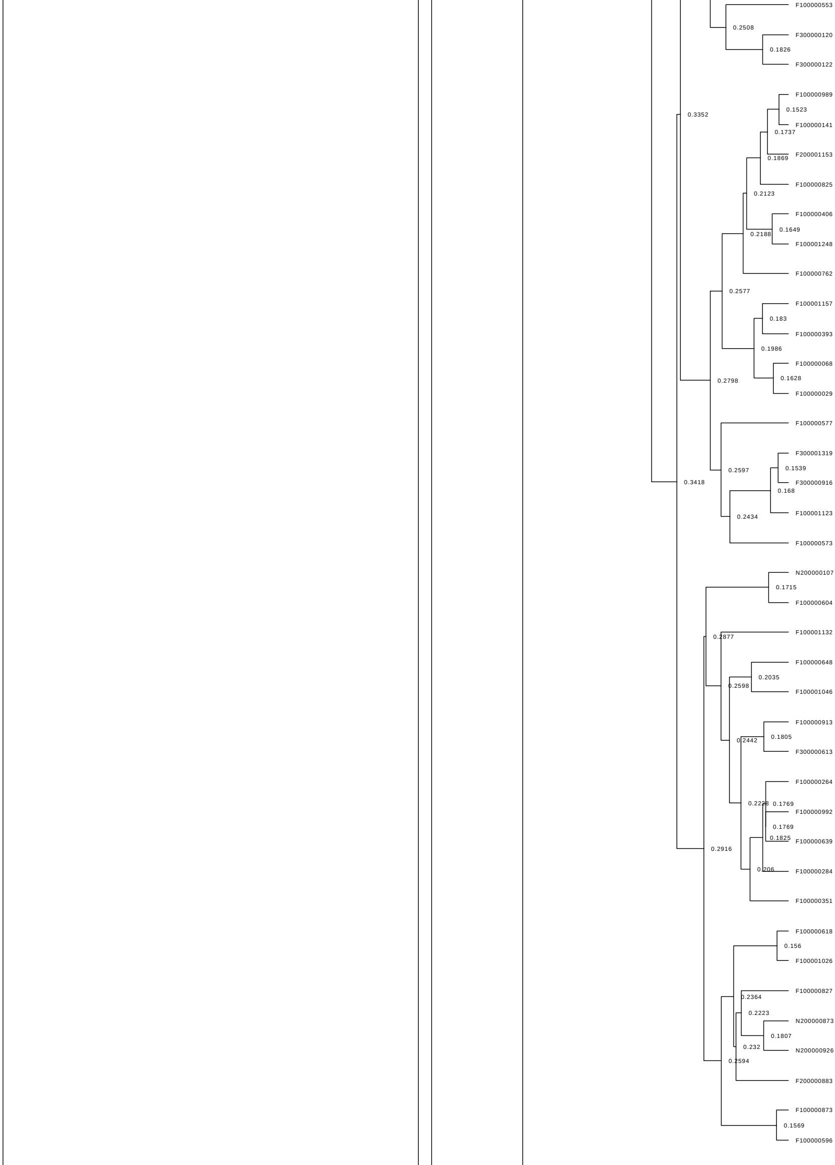
F100000553
0.2508
F300000120
0.1826
F300000122
F100000989
0.1523
0.3352
F100000141
0.1737
F200001153
0.1869
F100000825
0.2123
F100000406
0.1649
0.2188
F100001248
F100000762
0.2577
F100001157
0.183
F100000393
0.1986
F100000068
0.1628
0.2798
F100000029
F100000577
F300001319
0.1539
0.2597
F300000916
0.168
0.3418
F100001123
0.2434
F100000573
N200000107
0.1715
F100000604
F100001132
0.2877
F100000648
0.2035
0.2598
F100001046
F100000913
0.1805
0.2442
F300000613
F100000264
0.2228
0.1769
F100000992
0.1769
0.1825
F100000639
0.2916
0.206
F100000284
F100000351
F100000618
0.156
F100001026
F100000827
0.2364
0.2223
N200000873
0.1807
0.232
N200000926
0.2594
F200000883
F100000873
0.1569
F100000596

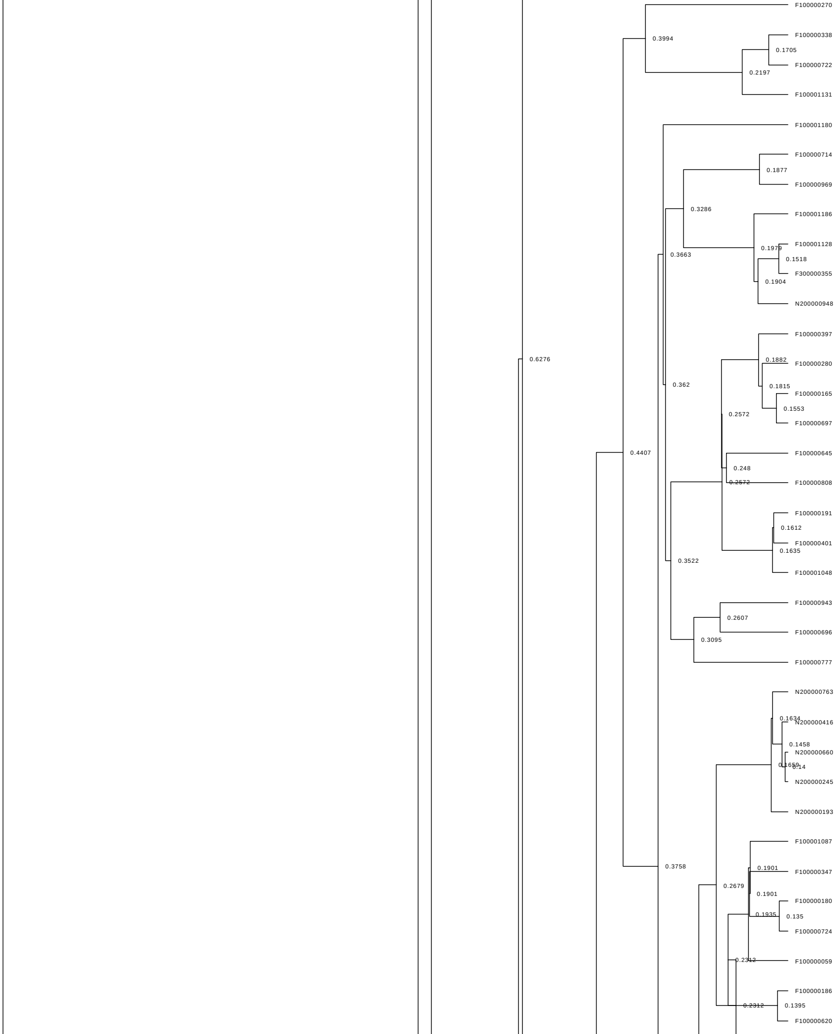
F100000270
0.3994
F100000338
0.1705
F100000722
0.2197
F100001131
F100001180
F100000714
0.1877
F100000969
0.3286
F100001186
F100001128
0.1979
0.1518
0.3663
F300000355
0.1904
N200000948
F100000397
0.6276
0.1882
F100000280
0.1815
0.362
F100000165
0.1553
0.2572
F100000697
F100000645
0.4407
0.248
0.2572
F100000808
F100000191
0.1612
F100000401
0.1635
0.3522
F100001048
F100000943
0.2607
F100000696
0.3095
F100000777
N200000763
0.1634
N200000416
0.1458
N200000660
0.1659
0.14
N200000245
N200000193
F100001087
0.3758
0.1901
F100000347
0.2679
0.1901
F100000180
0.1935
0.135
F100000724
0.2312
F100000059
F100000186
0.2312
0.1395
F100000620

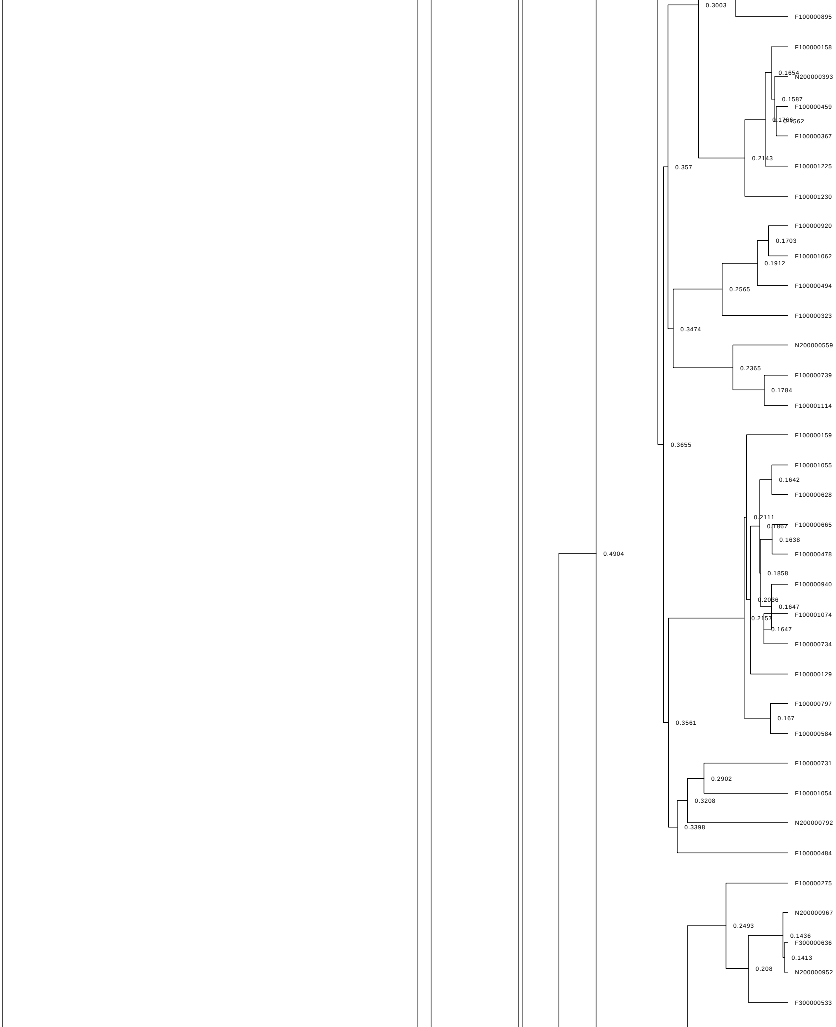

0.3003
F100000895
F100000158
0.1654
N200000393
0.1587
F100000459
0.1766
0.1562
F100000367
0.2143
F100001225
0.357
F100001230
F100000920
0.1703
F100001062
0.1912
F100000494
0.2565
F100000323
0.3474
N200000559
0.2365
F100000739
0.1784
F100001114
F100000159
0.3655
F100001055
0.1642
F100000628
0.2111
F100000665
0.1867
0.1638
F100000478
0.4904
0.1858
F100000940
0.2036
0.1647
F100001074
0.2157
0.1647
F100000734
F100000129
F100000797
0.167
0.3561
F100000584
F100000731
0.2902
F100001054
0.3208
N200000792
0.3398
F100000484
F100000275
N200000967
0.2493
0.1436
F300000636
0.1413
N200000952
0.208
F300000533

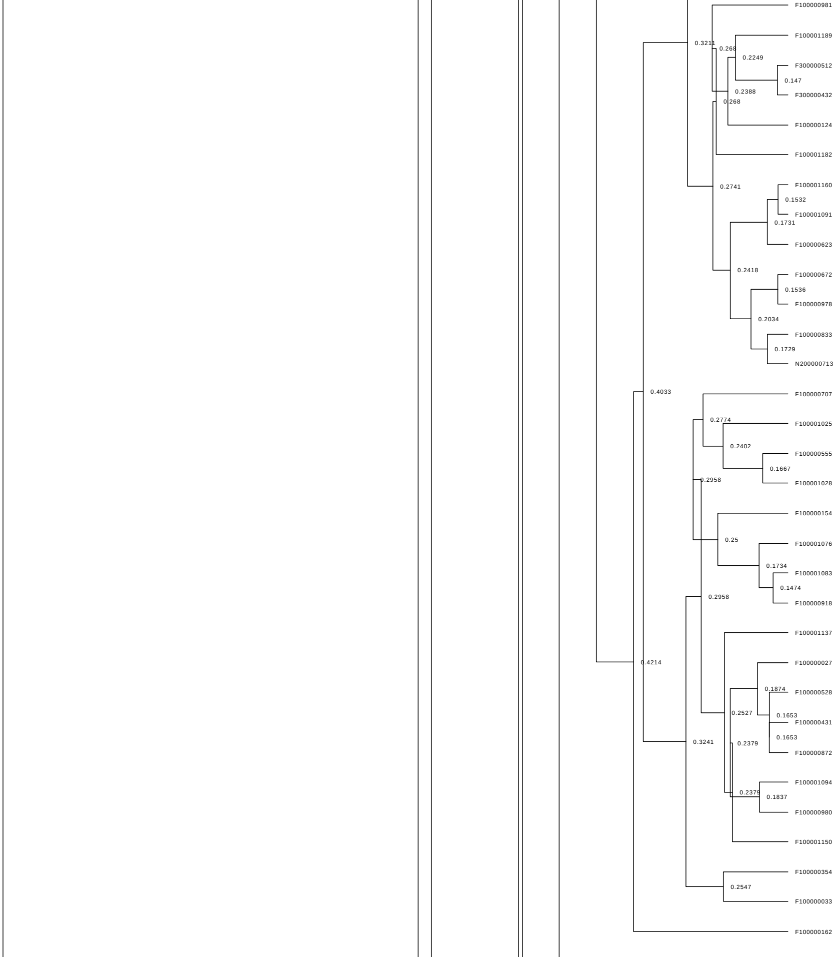
F100000981
0.3211
F100001189
0.268
0.2249
F300000512
0.147
F300000432
0.2388
0.268
F100000124
F100001182
0.2741
F100001160
0.1532
F100001091
0.1731
F100000623
0.2418
F100000672
0.1536
F100000978
0.2034
F100000833
0.1729
N200000713
0.4033
F100000707
0.2774
F100001025
0.2402
F100000555
0.1667
F100001028
0.2958
F100000154
0.25
F100001076
0.1734
F100001083
0.1474
F100000918
0.2958
F100001137
F100000027
0.1874
F100000528
0.4214
0.2527
0.1653
F100000431
0.1653
0.3241
0.2379
F100000872
F100001094
0.2379
0.1837
F100000980
F100001150
F100000354
0.2547
F100000033
F100000162

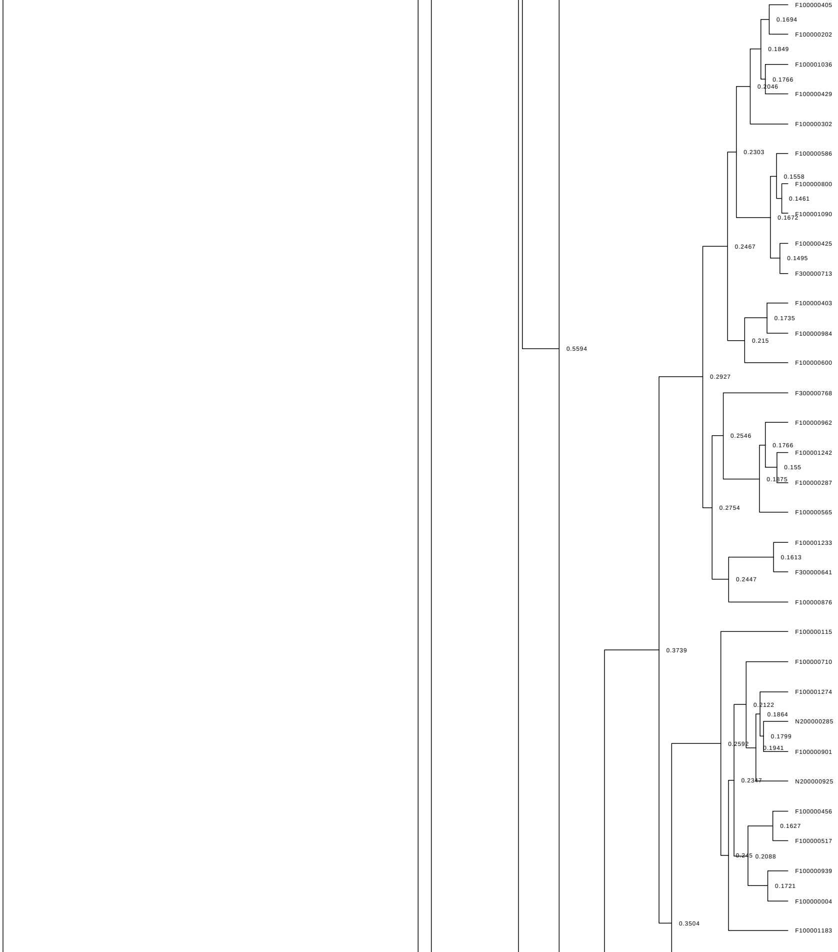
F100000405
0.1694
F100000202
0.1849
F100001036
0.1766
0.2046
F100000429
F100000302
0.2303
F100000586
0.1558
F100000800
0.1461
F100001090
0.1672
F100000425
0.2467
0.1495
F300000713
F100000403
0.1735
F100000984
0.215
0.5594
F100000600
0.2927
F300000768
F100000962
0.2546
0.1766
F100001242
0.155
0.1875
F100000287
F100000565
0.2754
F100001233
0.1613
F300000641
0.2447
F100000876
F100000115
0.3739
F100000710
F100001274
0.2122
0.1864
N200000285
0.1799
0.2592
0.1941
F100000901
0.2347
N200000925
F100000456
0.1627
F100000517
0.245
0.2088
F100000939
0.1721
F100000004
0.3504
F100001183

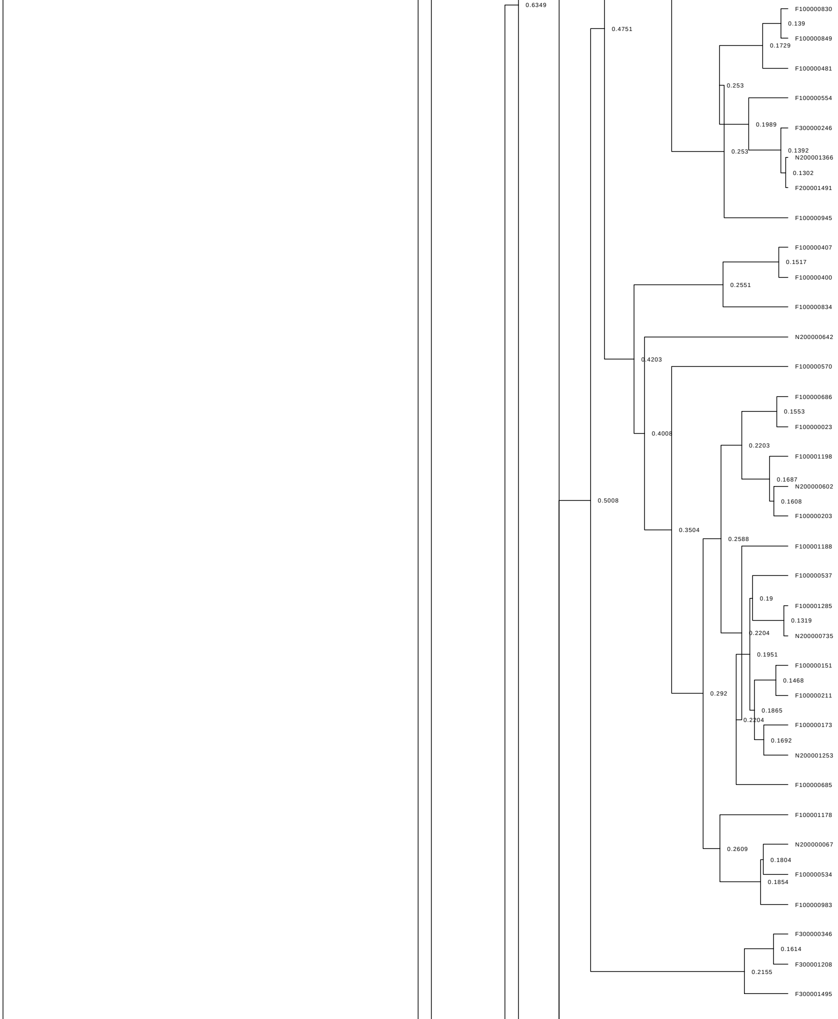
0.6349
0.4751
F100000830
0.139
F100000849
0.1729
F100000481
0.253
F100000554
0.1989
F300000246
0.1392
N200001366
0.1302
F200001491
0.253
F100000945
F100000407
0.1517
F100000400
0.2551
F100000834
N200000642
0.4203
F100000570
F100000686
0.1553
F100000023
0.4008
0.2203
F100001198
0.1687
N200000602
0.1608
F100000203
0.5008
0.3504
0.2588
F100001188
F100000537
0.19
F100001285
0.1319
0.2204
N200000735
0.1951
F100000151
0.1468
F100000211
0.292
0.1865
0.2204
F100000173
0.1692
N200001253
F100000685
F100001178
N200000067
0.2609
0.1804
F100000534
0.1854
F100000983
F300000346
0.1614
F300001208
0.2155
F300001495

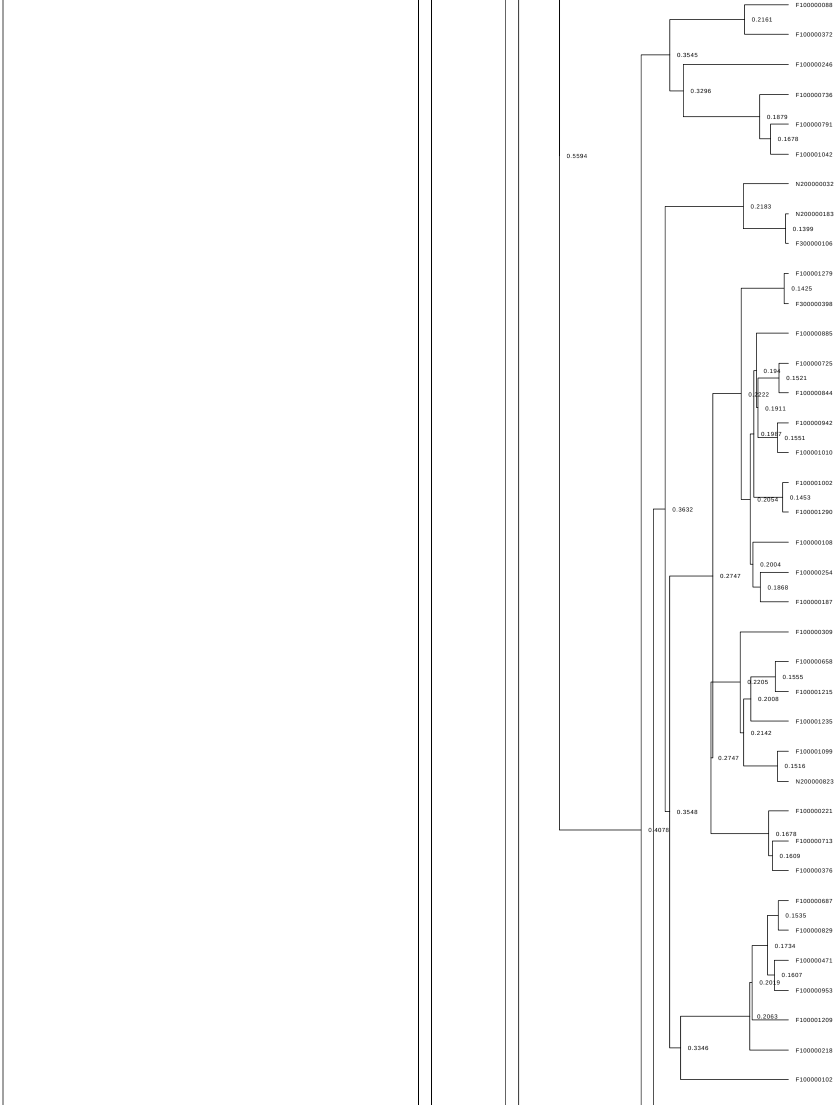
F100000088
0.2161
F100000372
0.3545
F100000246
0.3296
F100000736
0.1879
F100000791
0.1678
F100001042
0.5594
N200000032
0.2183
N200000183
0.1399
F300000106
F100001279
0.1425
F300000398
F100000885
F100000725
0.194
0.1521
F100000844
0.2222
0.1911
F100000942
0.1987
0.1551
F100001010
F100001002
0.1453
0.2054
F100001290
0.3632
F100000108
0.2004
F100000254
0.2747
0.1868
F100000187
F100000309
F100000658
0.1555
0.2205
F100001215
0.2008
F100001235
0.2142
F100001099
0.1516
0.2747
N200000823
F100000221
0.3548
0.4078
0.1678
F100000713
0.1609
F100000376
F100000687
0.1535
F100000829
0.1734
F100000471
0.1607
0.2019
F100000953
0.2063
F100001209
F100000218
0.3346
F100000102

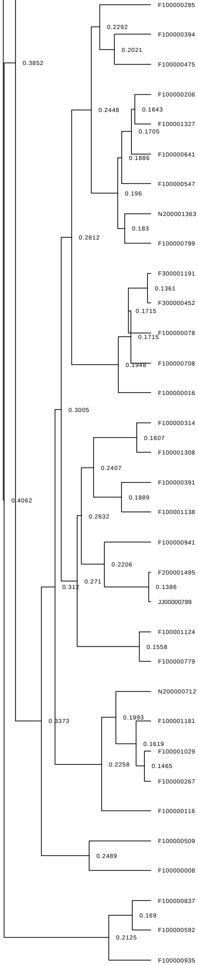
F100000285
0.2292
F100000394
0.2021
F100000475
0.3852
F100000206
0.1643
0.2448
F100001327
0.1705
F100000641
0.1886
F100000547
0.196
N200001363
0.183
0.2812
F100000799
F300001191
0.1361
F300000452
0.1715
F100000078
0.1715
F100000708
0.1948
F100000016
0.3005
F100000314
0.1607
F100001308
0.2407
F100000391
0.1889
0.4062
F100001138
0.2632
F100000941
0.2206
F200001495
0.271
0.312
0.1386
JJ00000789
F100001124
0.1558
F100000779
N200000712
0.1993
F100001181
0.3373
0.1619
F100001029
0.1465
0.2258
F100000267
F100000116
F100000509
0.2489
F100000008
F100000837
0.169
F100000592
0.2125
F100000935

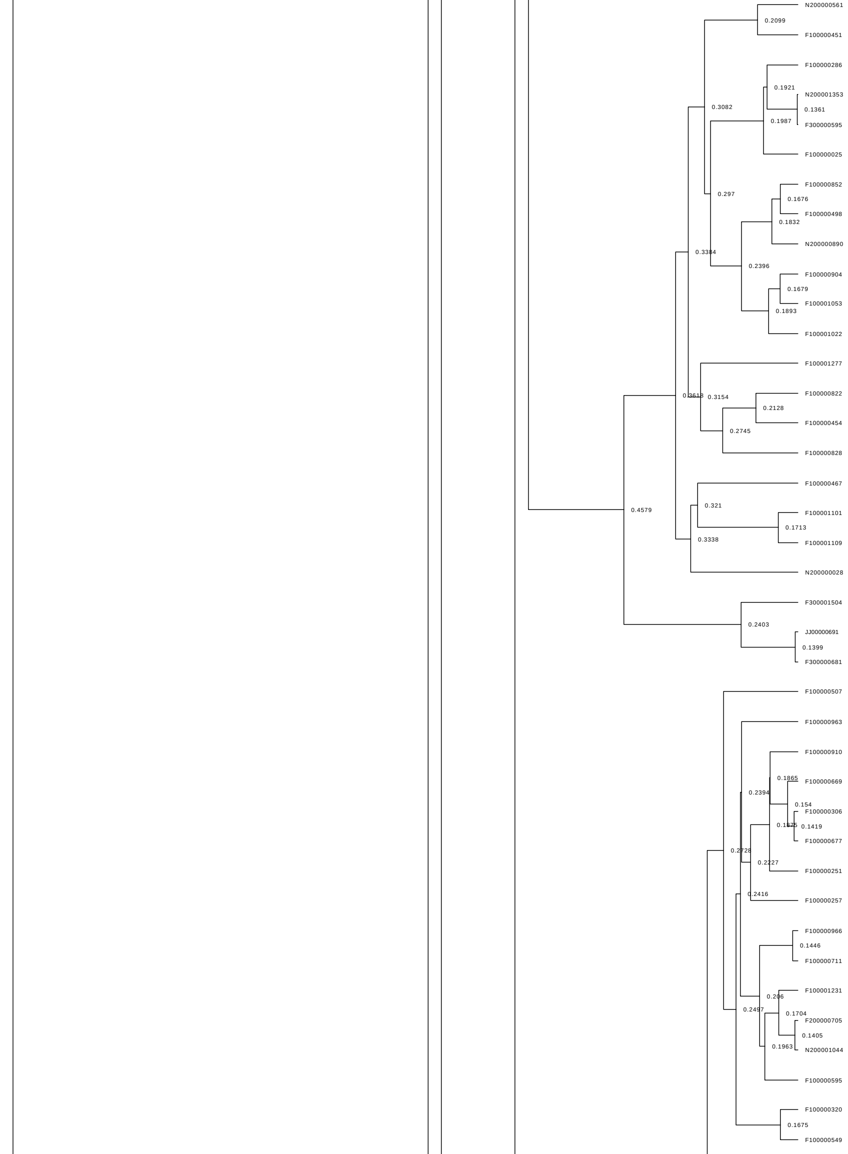
N200000561
0.2099
F100000451
F100000286
0.1921
N200001353
0.1361
F300000595
0.3082
0.1987
F100000025
F100000852
0.297
0.1676
F100000498
0.1832
N200000890
0.3384
0.2396
F100000904
0.1679
F100001053
0.1893
F100001022
F100001277
F100000822
0.3618
0.3154
0.2128
F100000454
0.2745
F100000828
F100000467
0.321
F100001101
0.4579
0.1713
F100001109
0.3338
N200000028
F300001504
0.2403
JJ00000691
0.1399
F300000681
F100000507
F100000963
F100000910
0.1865
F100000669
0.2394
0.154
F100000306
0.1875
0.1419
F100000677
0.2728
0.2227
F100000251
0.2416
F100000257
F100000966
0.1446
F100000711
F100001231
0.206
0.2497
0.1704
F200000705
0.1405
0.1963
N200001044
F100000595
F100000320
0.1675
F100000549

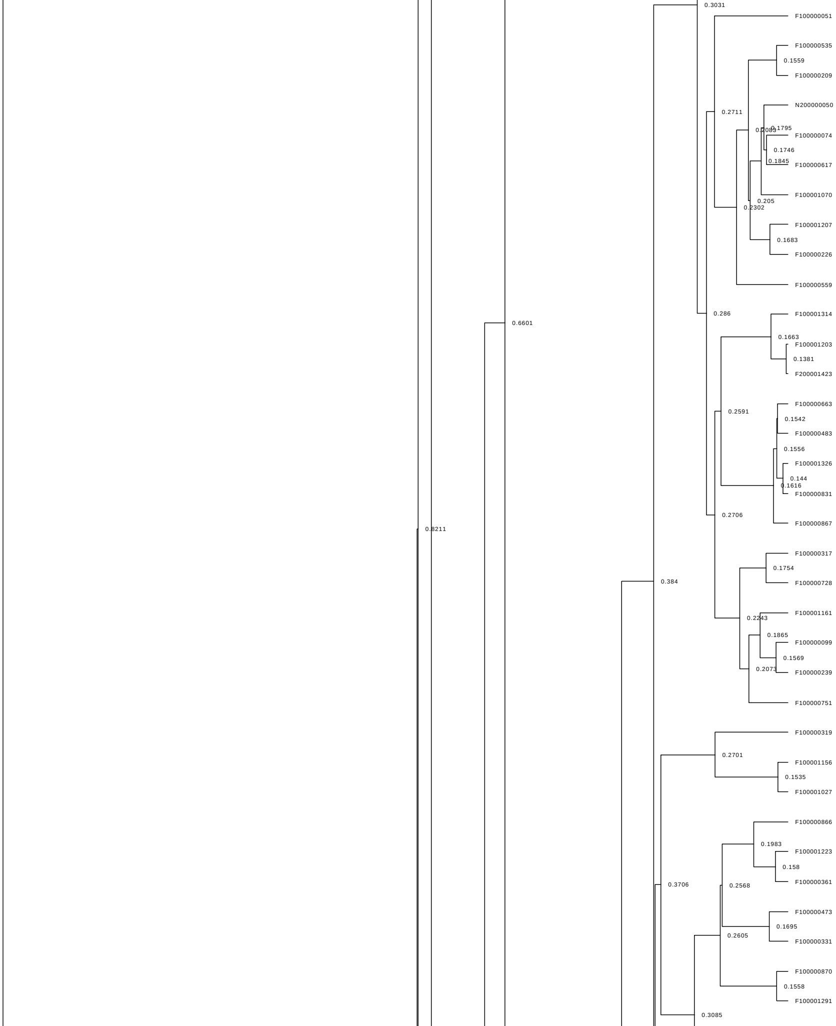
0.3031
F100000051
F100000535
0.1559
F100000209
0.2711
N200000050
0.2089
0.1795
F100000074
0.1746
0.1845
F100000617
F100001070
0.205
0.2302
F100001207
0.1683
F100000226
F100000559
0.286
F100001314
0.6601
0.1663
F100001203
0.1381
F200001423
F100000663
0.2591
0.1542
F100000483
0.1556
F100001326
0.144
0.1616
F100000831
0.2706
F100000867
0.8211
F100000317
0.1754
0.384
F100000728
F100001161
0.2243
0.1865
F100000099
0.1569
0.2073
F100000239
F100000751
F100000319
0.2701
F100001156
0.1535
F100001027
F100000866
0.1983
F100001223
0.158
F100000361
0.3706
0.2568
F100000473
0.1695
0.2605
F100000331
F100000870
0.1558
F100001291
0.3085

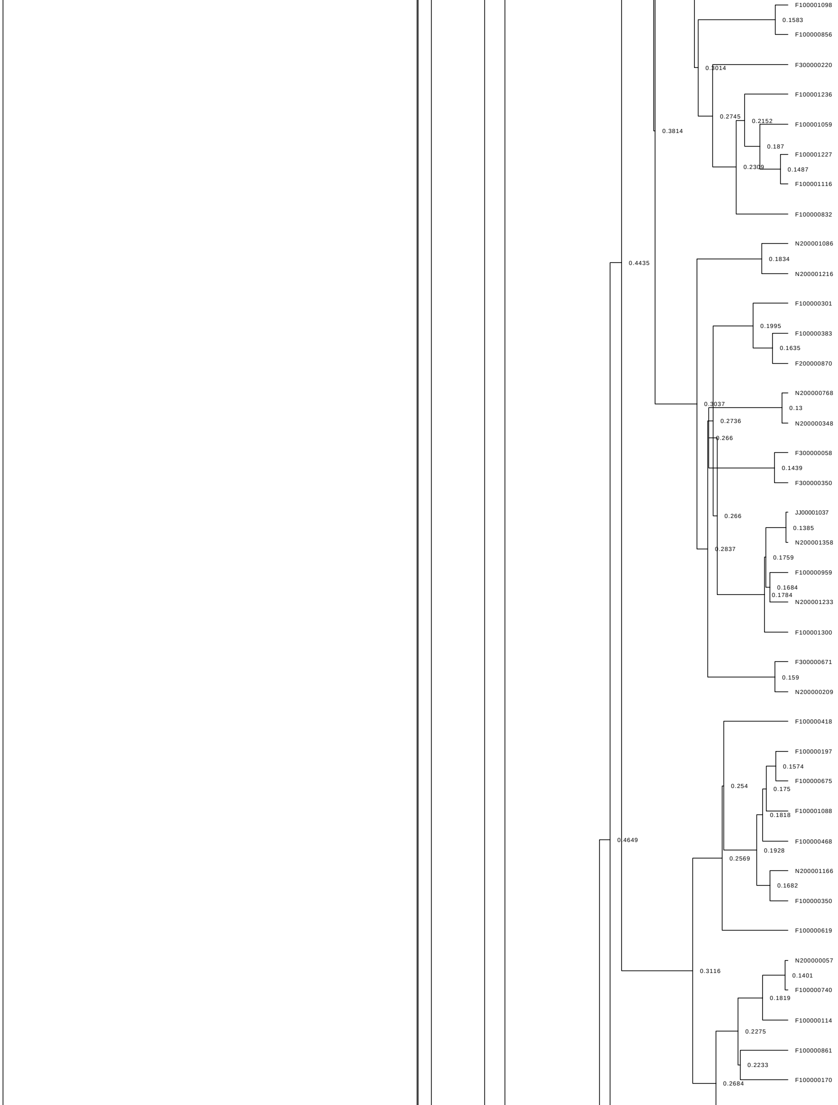

F100001098
0.1583
F100000856
0.3014
F300000220
F100001236
0.2745
0.2152
F100001059
0.3814
0.187
F100001227
0.2309
0.1487
F100001116
F100000832
N200001086
0.4435
0.1834
N200001216
F100000301
0.1995
F100000383
0.1635
F200000870
N200000768
0.3037
0.13
0.2736
N200000348
0.266
F300000058
0.1439
F300000350
0.266
JJ00001037
0.1385
0.2837
N200001358
0.1759
F100000959
0.1684
0.1784
N200001233
F100001300
F300000671
0.159
N200000209
F100000418
F100000197
0.1574
0.254
F100000675
0.175
F100001088
0.1818
0.4649
F100000468
0.1928
0.2569
N200001166
0.1682
F100000350
F100000619
N200000057
0.3116
0.1401
F100000740
0.1819
F100000114
0.2275
F100000861
0.2233
0.2684
F100000170

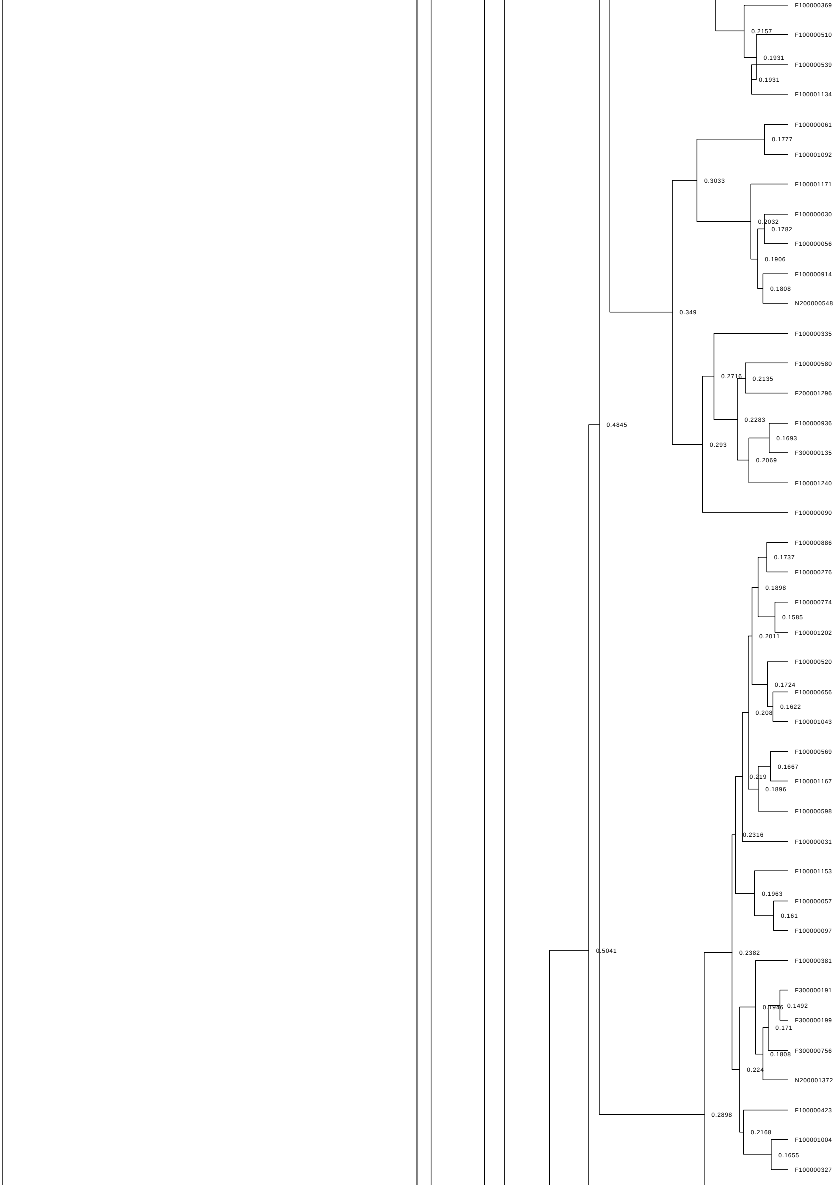
F100000369
0.2157
F100000510
0.1931
F100000539
0.1931
F100001134
F100000061
0.1777
F100001092
0.3033
F100001171
F100000030
0.2032
0.1782
F100000056
0.1906
F100000914
0.1808
N200000548
0.349
F100000335
F100000580
0.2716
0.2135
F200001296
0.2283
F100000936
0.4845
0.1693
0.293
F300000135
0.2069
F100001240
F100000090
F100000886
0.1737
F100000276
0.1898
F100000774
0.1585
F100001202
0.2011
F100000520
0.1724
F100000656
0.1622
0.208
F100001043
F100000569
0.1667
0.219
F100001167
0.1896
F100000598
0.2316
F100000031
F100001153
0.1963
F100000057
0.161
F100000097
0.5041
0.2382
F100000381
F300000191
0.1946
0.1492
F300000199
0.171
F300000756
0.1808
0.224
N200001372
F100000423
0.2898
0.2168
F100001004
0.1655
F100000327

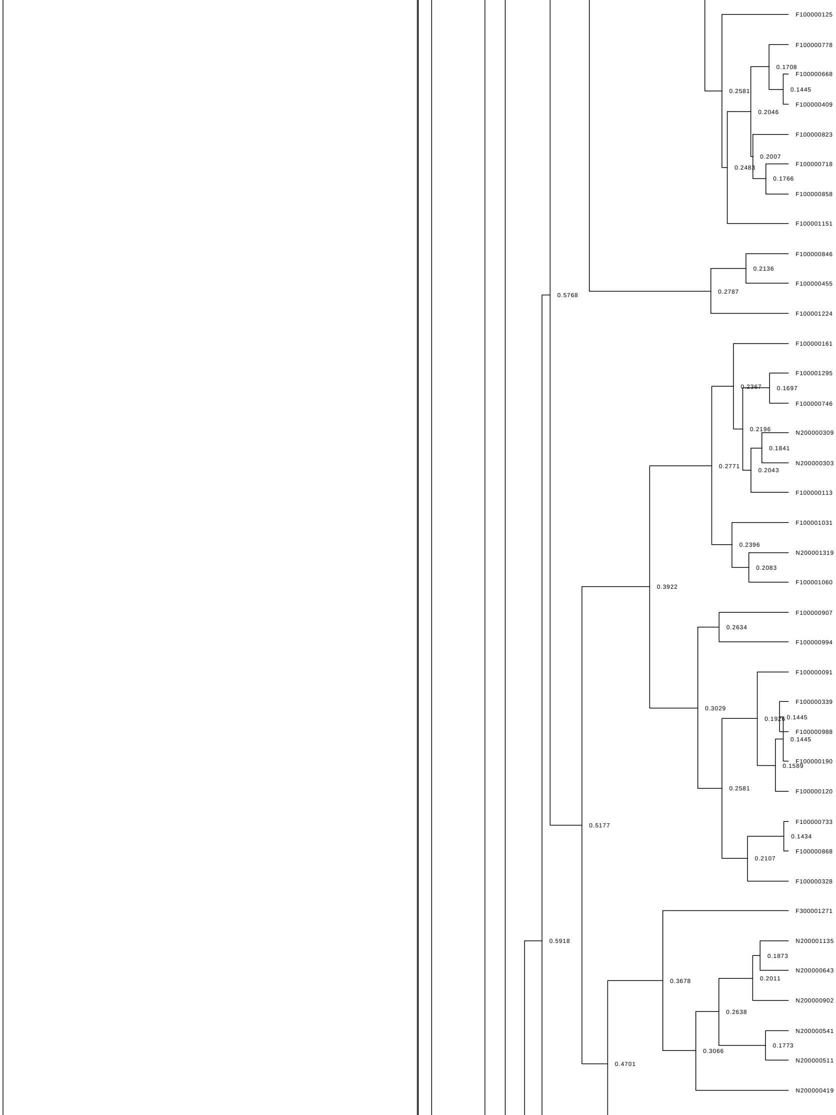
F100000125
F100000778
0.1708
F100000668
0.1445
F100000409
0.2581
0.2046
F100000823
0.2007
F100000718
0.2483
0.1766
F100000858
F100001151
F100000846
0.2136
F100000455
0.2787
0.5768
F100001224
F100000161
F100001295
0.2367
0.1697
F100000746
0.2196
N200000309
0.1841
N200000303
0.2771
0.2043
F100000113
F100001031
0.2396
N200001319
0.2083
F100001060
0.3922
F100000907
0.2634
F100000994
F100000091
F100000339
0.1445
0.3029
F100000988
0.1445
F100000190
0.1589
0.2581
F100000120
F100000733
0.1434
F100000868
0.2107
0.5177
F100000328
F300001271
N200001135
0.5918
0.1873
N200000643
0.2011
0.3678
N200000902
0.2638
N200000541
0.1773
0.3066
N200000511
0.4701
N200000419

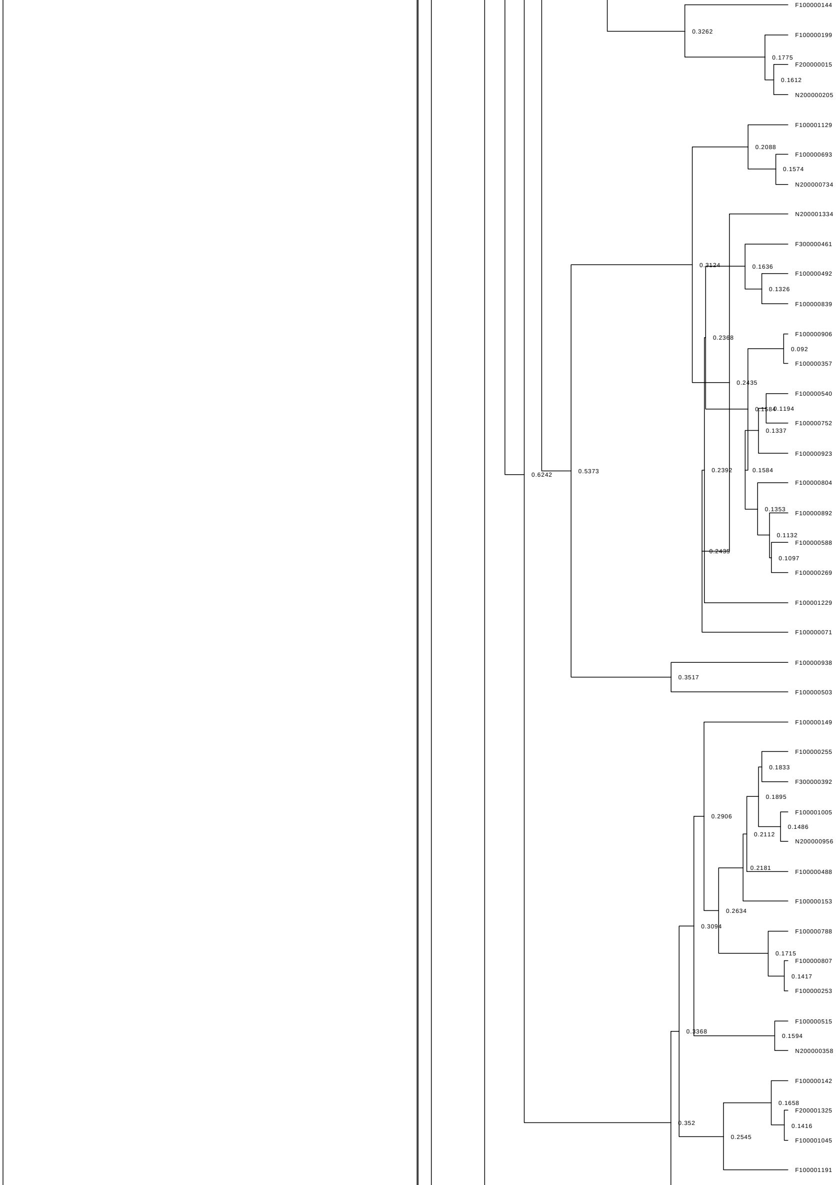

F100000144
0.3262
F100000199
0.1775
F200000015
0.1612
N200000205
F100001129
0.2088
F100000693
0.1574
N200000734
N200001334
F300000461
0.3124
0.1636
F100000492
0.1326
F100000839
F100000906
0.2368
0.092
F100000357
0.2435
F100000540
0.1584
0.1194
F100000752
0.1337
F100000923
0.6242
0.5373
0.2392
0.1584
F100000804
0.1353
F100000892
0.1132
F100000588
0.2435
0.1097
F100000269
F100001229
F100000071
F100000938
0.3517
F100000503
F100000149
F100000255
0.1833
F300000392
0.1895
F100001005
0.2906
0.1486
0.2112
N200000956
0.2181
F100000488
F100000153
0.2634
0.3094
F100000788
0.1715
F100000807
0.1417
F100000253
F100000515
0.3368
0.1594
N200000358
F100000142
0.1658
F200001325
0.352
0.1416
0.2545
F100001045
F100001191

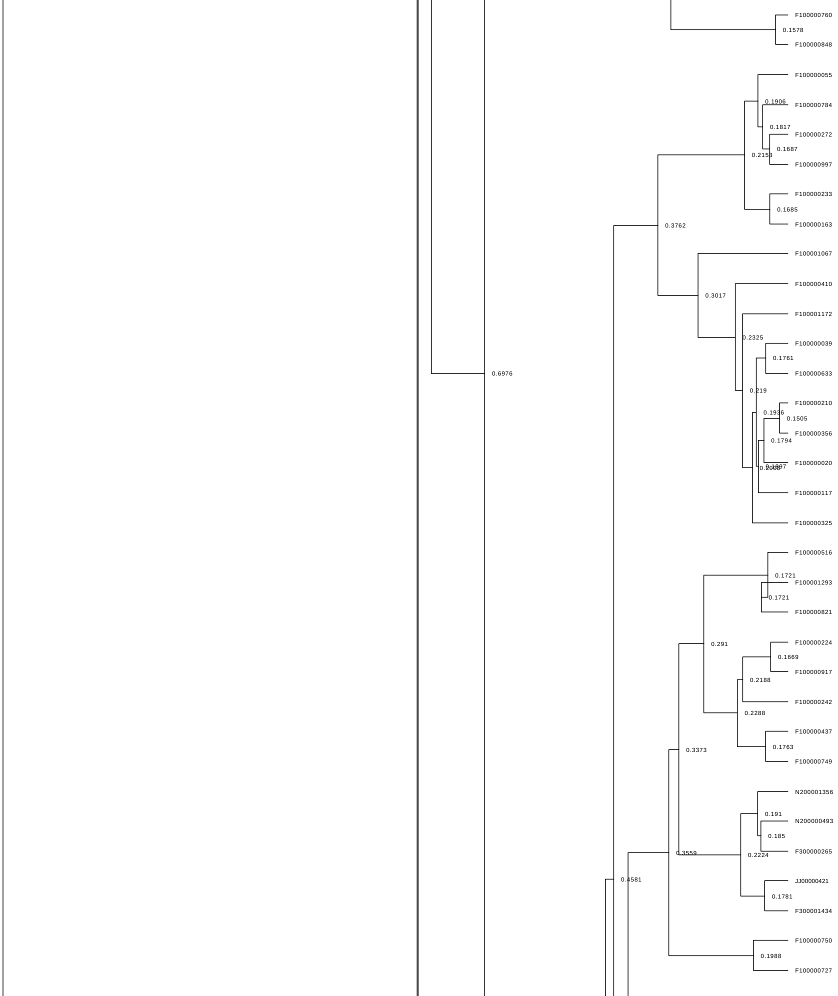
F100000760
0.1578
F100000848
F100000055
0.1906
F100000784
0.1817
F100000272
0.1687
0.2153
F100000997
F100000233
0.1685
F100000163
0.3762
F100001067
F100000410
0.3017
F100001172
0.2325
F100000039
0.1761
F100000633
0.219
F100000210
0.1936
0.1505
F100000356
0.1794
F100000020
F100000117
F100000325
F100000516
0.1721
F100001293
0.1721
F100000821
F100000224
0.291
0.1669
F100000917
0.2188
F100000242
0.2288
F100000437
0.1763
F100000749
0.3373
N200001356
0.191
N200000493
0.185
F300000265
0.3559
0.2224
JJ00000421
0.1781
F300001434
0.4581
F100000750
0.1988
F100000727
0.6976

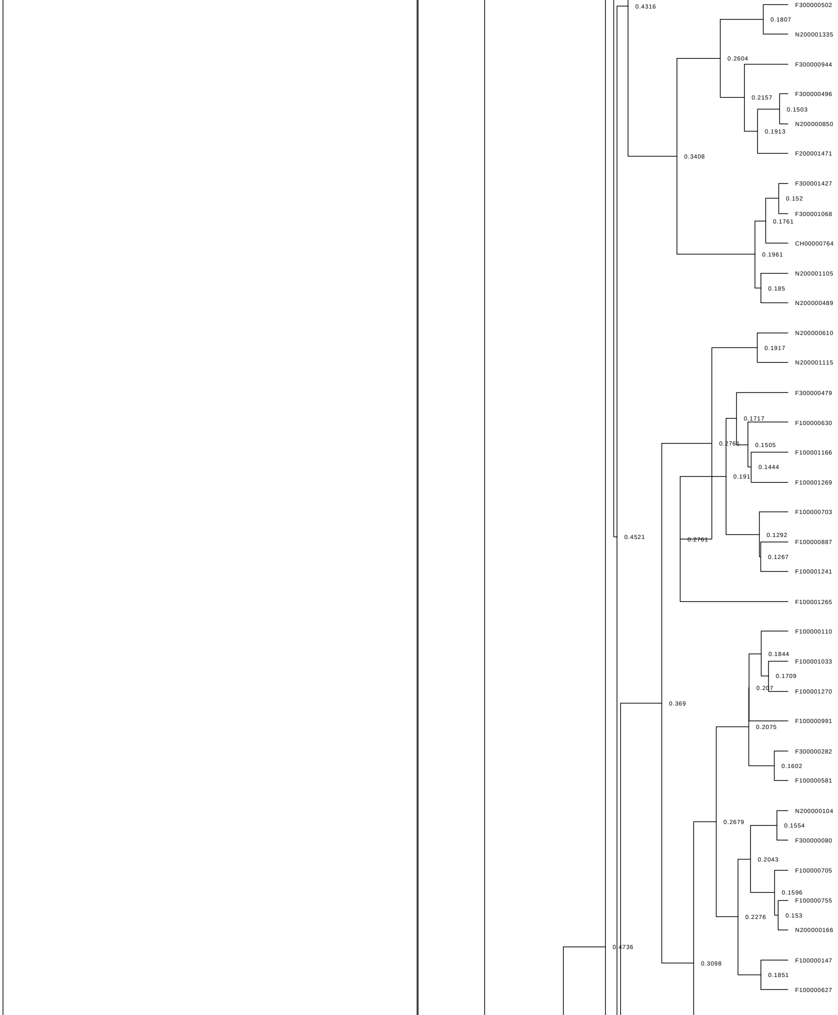

F300000502
0.4316
0.1807
N200001335
0.2604
F300000944
F300000496
0.2157
0.1503
N200000850
0.1913
F200001471
0.3408
F300001427
0.152
F300001068
0.1761
CH00000764
0.1961
N200001105
0.185
N200000489
N200000610
0.1917
N200001115
F300000479
0.1717
F100000630
0.276
0.1505
F100001166
0.1444
0.191
F100001269
F100000703
0.4521
0.2761
0.1292
F100000887
0.1267
F100001241
F100001265
F100000110
0.1844
F100001033
0.1709
0.207
F100001270
0.369
F100000991
0.2075
F300000282
0.1602
F100000581
N200000104
0.2679
0.1554
F300000080
0.2043
F100000705
0.1596
F100000755
0.153
N200000166
0.2276
0.4736
F100000147
0.3098
0.1851
F100000627

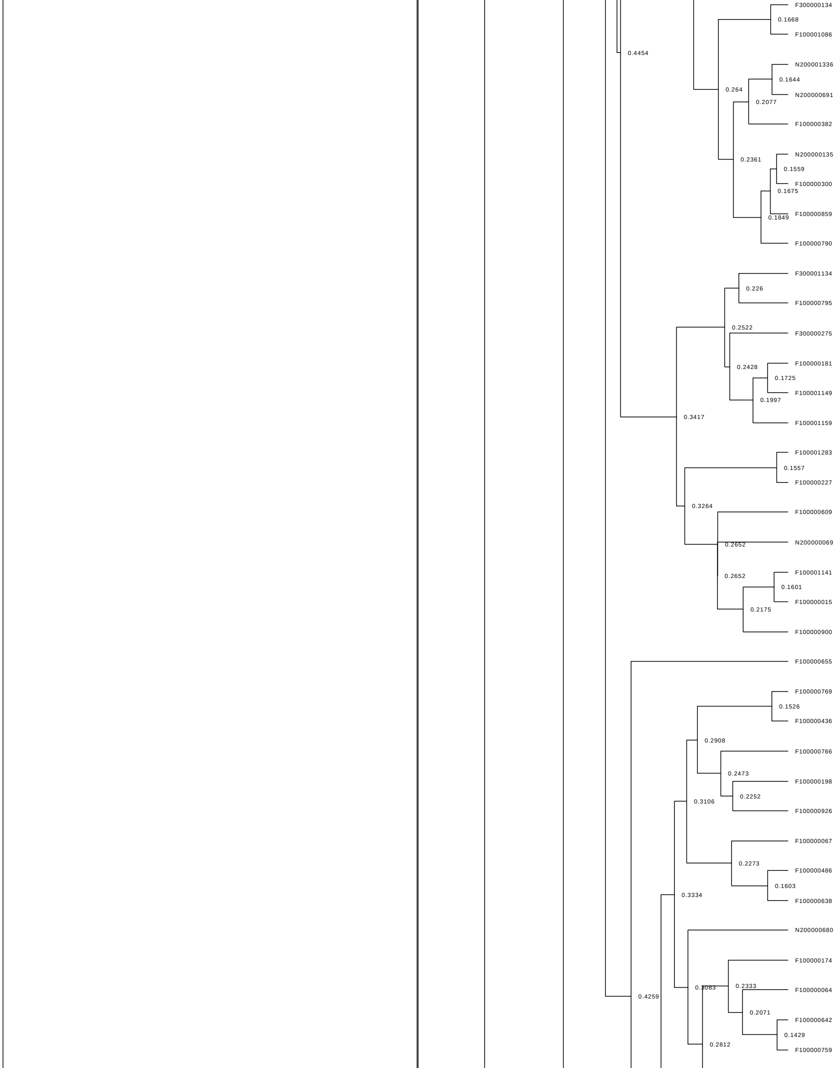

F300000134
0.1668
F100001086
0.4454
N200001336
0.1644
N200000691
0.264
0.2077
F100000382
N200000135
0.2361
0.1559
F100000300
0.1675
F100000859
0.1849
F100000790
F300001134
0.226
F100000795
0.2522
F300000275
F100000181
0.2428
0.1725
F100001149
0.1997
0.3417
F100001159
F100001283
0.1557
F100000227
0.3264
F100000609
N200000069
0.2652
F100001141
0.2652
0.1601
F100000015
0.2175
F100000900
F100000655
F100000769
0.1526
F100000436
0.2908
F100000766
0.2473
F100000198
0.2252
0.3106
F100000926
F100000067
0.2273
F100000486
0.1603
0.3334
F100000638
N200000680
F100000174
0.3083
0.2333
F100000064
0.4259
0.2071
F100000642
0.1429
0.2812
F100000759

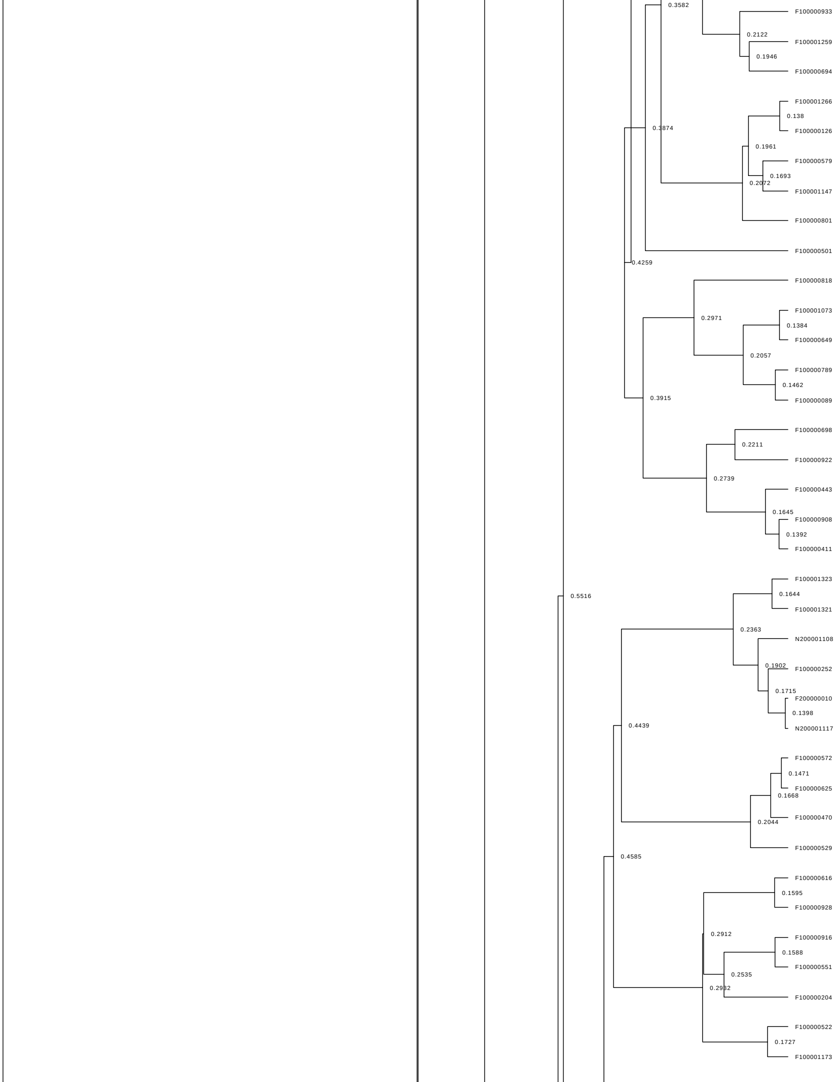
0.3582
F100000933
0.2122
F100001259
0.1946
F100000694
F100001266
0.138
F100000126
0.3874
0.1961
F100000579
0.1693
0.2072
F100001147
F100000801
F100000501
0.4259
F100000818
F100001073
0.2971
0.1384
F100000649
0.2057
F100000789
0.1462
0.3915
F100000089
F100000698
0.2211
F100000922
0.2739
F100000443
0.1645
F100000908
0.1392
F100000411
F100001323
0.5516
0.1644
F100001321
0.2363
N200001108
0.1902
F100000252
0.1715
F200000010
0.1398
0.4439
N200001117
F100000572
0.1471
F100000625
0.1668
F100000470
0.2044
F100000529
0.4585
F100000616
0.1595
F100000928
0.2912
F100000916
0.1588
F100000551
0.2535
0.2932
F100000204
F100000522
0.1727
F100001173

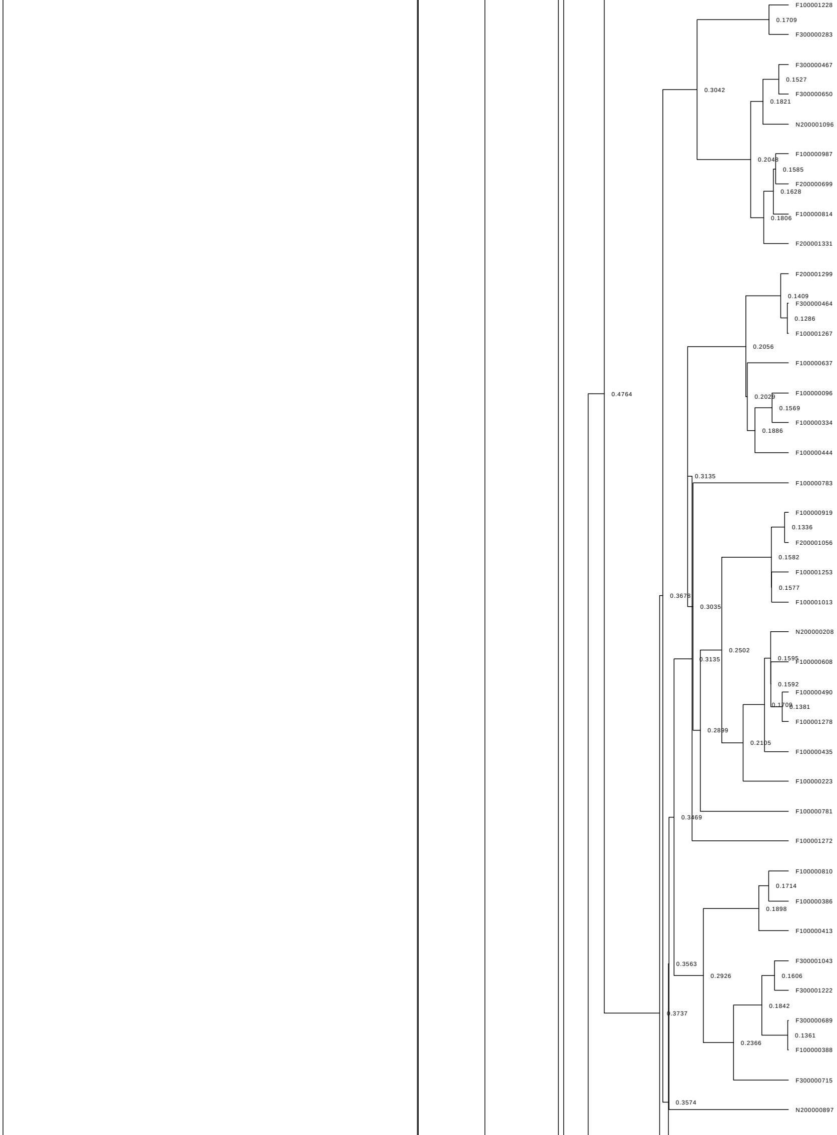
F100001228
0.1709
F300000283
F300000467
0.1527
F300000650
0.1821
N200001096
F100000987
0.2048
0.1585
F200000699
0.1628
F100000814
0.1806
F200001331
0.3042
F200001299
0.1409
F300000464
0.1286
F100001267
0.2056
F100000637
0.2029
F100000096
0.1569
F100000334
0.1886
F100000444
0.4764
0.3135
F100000783
F100000919
0.1336
F200001056
0.1582
F100001253
0.1577
F100001013
0.3678
0.3035
N200000208
0.2502
0.3135
0.1595
F100000608
0.1592
F100000490
0.1709
0.1381
F100001278
0.2899
0.2105
F100000435
F100000223
F100000781
0.3469
F100001272
F100000810
0.1714
F100000386
0.1898
F100000413
0.3563
F300001043
0.2926
0.1606
F300001222
0.1842
0.3737
F300000689
0.1361
0.2366
F100000388
F300000715
0.3574
N200000897

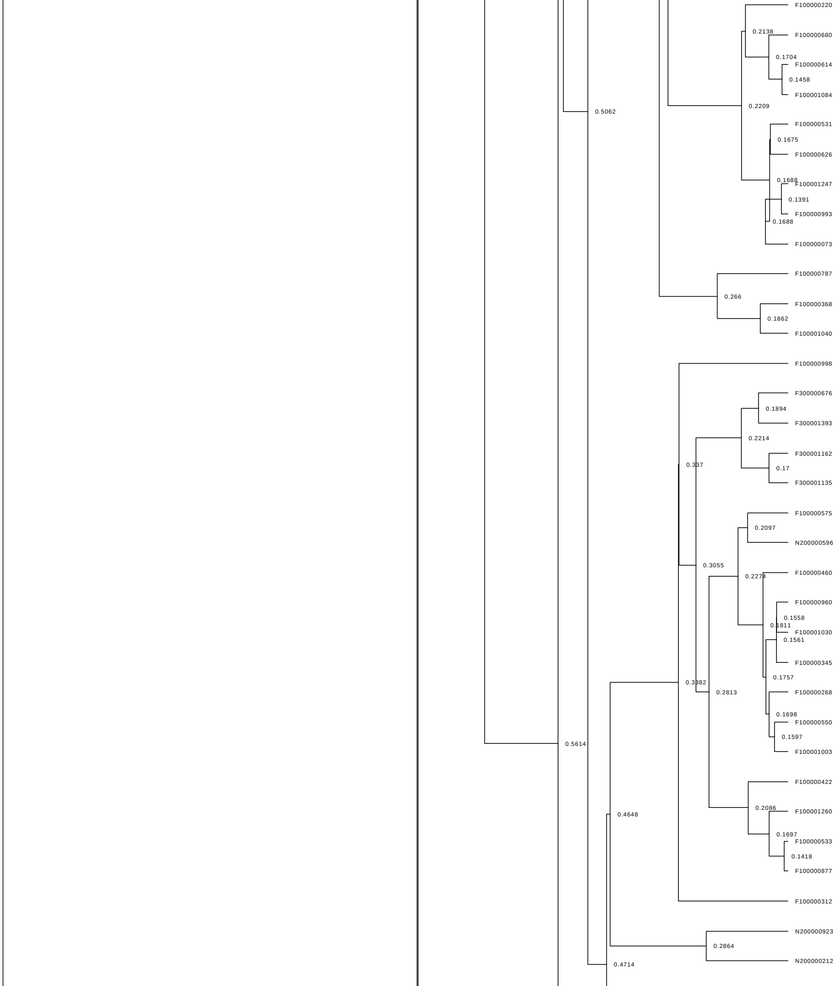
F100000220
0.2138
F100000680
0.1704
F100000614
0.1458
F100001084
0.2209
0.5062
F100000531
0.1675
F100000626
0.1688
F100001247
0.1391
F100000993
0.1688
F100000073
F100000787
0.266
F100000368
0.1862
F100001040
F100000998
F300000676
0.1894
F300001393
0.2214
F300001162
0.337
0.17
F300001135
F100000575
0.2097
N200000596
0.3055
F100000460
0.2274
F100000960
0.1558
0.1811
F100001030
0.1561
F100000345
0.1757
0.3382
0.2813
F100000268
0.1698
F100000550
0.1597
0.5614
F100001003
F100000422
0.2086
F100001260
0.4648
0.1697
F100000533
0.1418
F100000877
F100000312
N200000923
0.2864
N200000212
0.4714

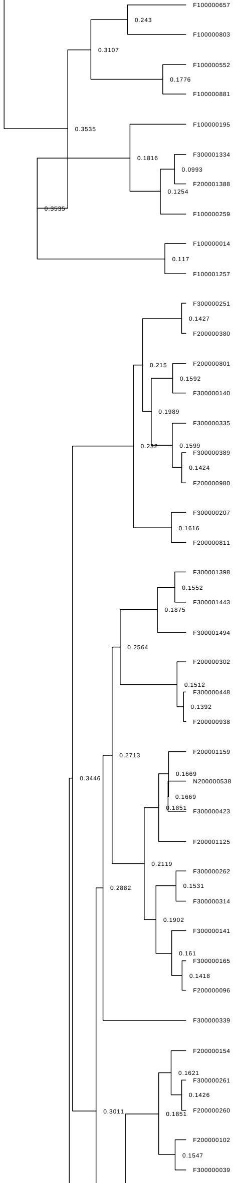
F100000657
0.243
F100000803
0.3107
F100000552
0.1776
F100000881
F100000195
0.3535
0.1816
F300001334
0.0993
F200001388
0.1254
0.3535
F100000259
F100000014
0.117
F100001257
F300000251
0.1427
F200000380
F200000801
0.215
0.1592
F300000140
0.1989
F300000335
0.232
0.1599
F300000389
0.1424
F200000980
F300000207
0.1616
F200000811
F300001398
0.1552
F300001443
0.1875
F300001494
0.2564
F200000302
0.1512
F300000448
0.1392
F200000938
F200001159
0.2713
0.1669
0.3446
N200000538
0.1669
0.1851
F300000423
F200001125
0.2119
F300000262
0.1531
0.2882
F300000314
0.1902
F300000141
0.161
F300000165
0.1418
F200000096
F300000339
F200000154
0.1621
F300000261
0.1426
0.3011
0.1851
F200000260
F200000102
0.1547
F300000039

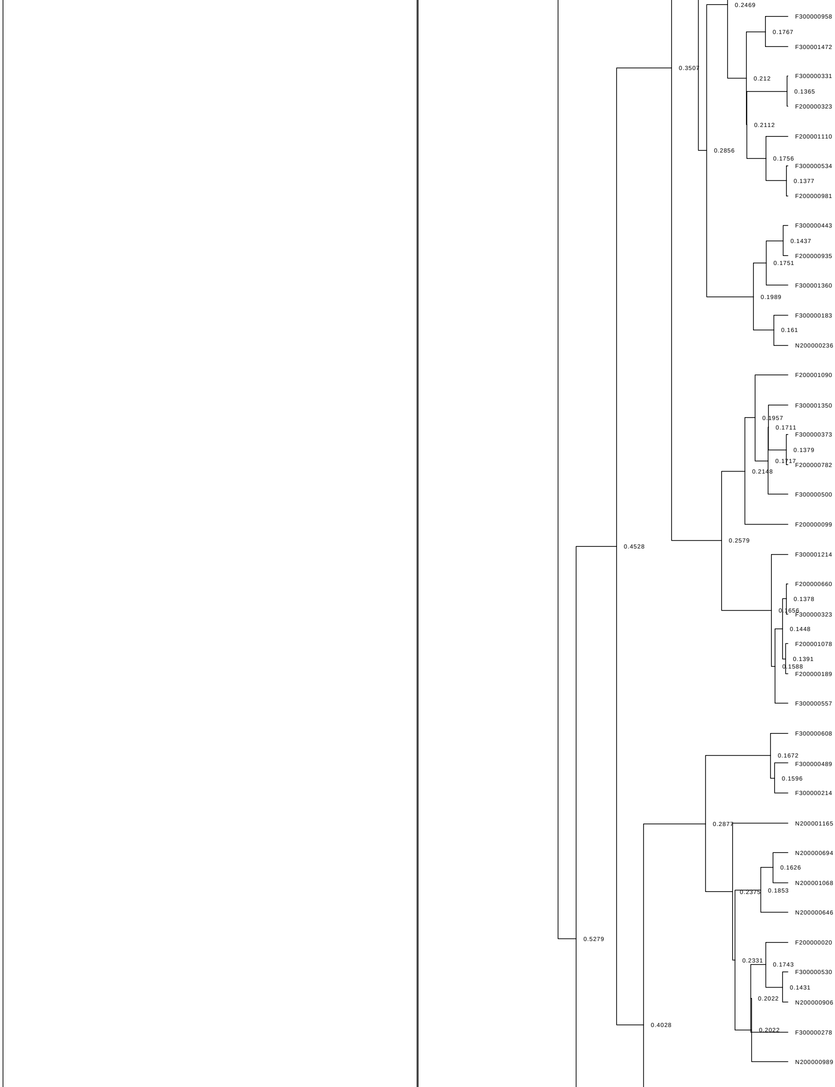
0.2469
F300000958
0.1767
F300001472
0.3507
F300000331
0.212
0.1365
F200000323
0.2112
F200001110
0.2856
0.1756
F300000534
0.1377
F200000981
F300000443
0.1437
F200000935
0.1751
F300001360
0.1989
F300000183
0.161
N200000236
F200001090
F300001350
0.1957
0.1711
F300000373
0.1379
0.1717
F200000782
0.2148
F300000500
F200000099
0.4528
0.2579
F300001214
F200000660
0.1378
0.1656
F300000323
0.1448
F200001078
0.1391
0.1588
F200000189
F300000557
F300000608
0.1672
F300000489
0.1596
F300000214
0.2877
N200001165
N200000694
0.1626
N200001068
0.2375
0.1853
N200000646
0.5279
F200000020
0.2331
0.1743
F300000530
0.1431
0.2022
N200000906
0.4028
0.2022
F300000278
N200000989

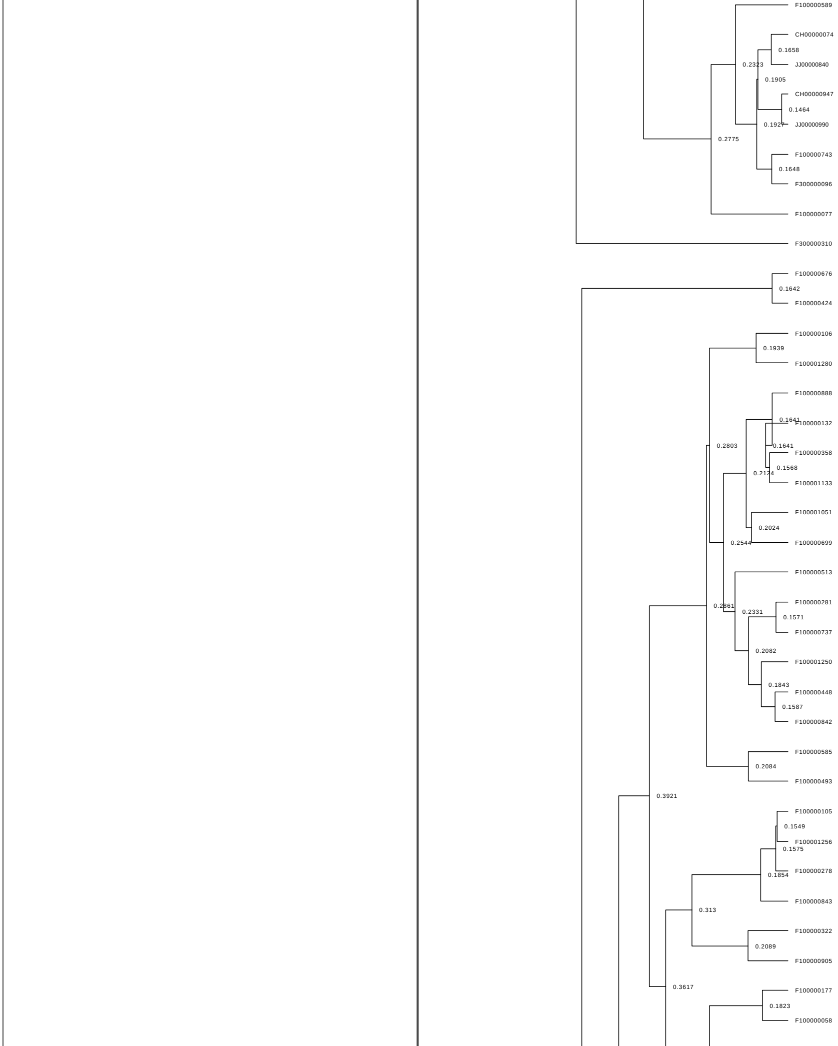
F100000589
CH00000074
0.1658
0.2323
JJ00000840
0.1905
CH00000947
0.1464
0.1927
JJ00000990
0.2775
F100000743
0.1648
F300000096
F100000077
F300000310
F100000676
0.1642
F100000424
F100000106
0.1939
F100001280
F100000888
0.1641
F100000132
0.2803
0.1641
F100000358
0.1568
0.2124
F100001133
F100001051
0.2024
0.2544
F100000699
F100000513
F100000281
0.2861
0.2331
0.1571
F100000737
0.2082
F100001250
0.1843
F100000448
0.1587
F100000842
F100000585
0.2084
F100000493
0.3921
F100000105
0.1549
F100001256
0.1575
F100000278
0.1854
F100000843
0.313
F100000322
0.2089
F100000905
0.3617
F100000177
0.1823
F100000058

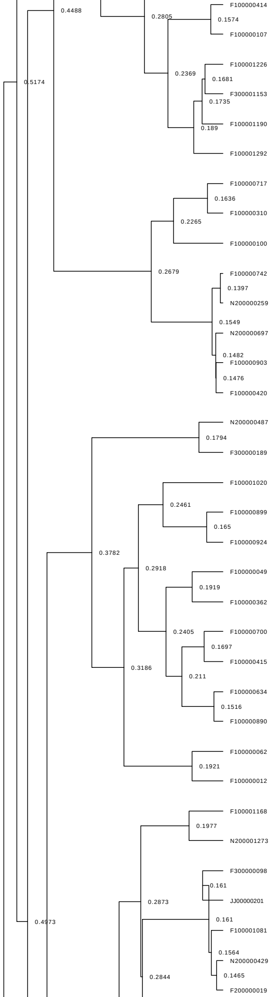

F100000414
0.4488
0.2805
0.1574
F100000107
F100001226
0.2369
0.1681
0.5174
F300001153
0.1735
F100001190
0.189
F100001292
F100000717
0.1636
F100000310
0.2265
F100000100
0.2679
F100000742
0.1397
N200000259
0.1549
N200000697
0.1482
F100000903
0.1476
F100000420
N200000487
0.1794
F300000189
F100001020
0.2461
F100000899
0.165
F100000924
0.3782
0.2918
F100000049
0.1919
F100000362
0.2405
F100000700
0.1697
F100000415
0.3186
0.211
F100000634
0.1516
F100000890
F100000062
0.1921
F100000012
F100001168
0.1977
N200001273
F300000098
0.161
JJ00000201
0.2873
0.4973
0.161
F100001081
0.1564
N200000429
0.1465
0.2844
F200000019

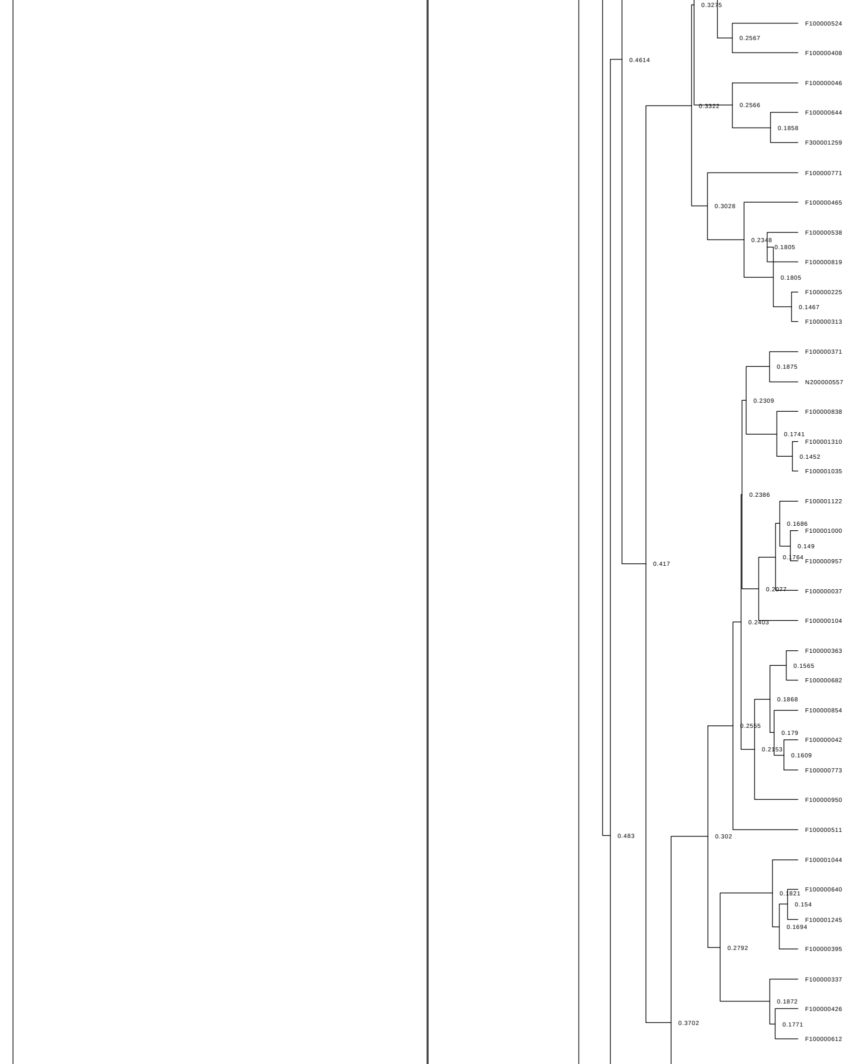
0.3275
F100000524
0.2567
F100000408
0.4614
F100000046
0.3322
0.2566
F100000644
0.1858
F300001259
F100000771
F100000465
0.3028
F100000538
0.2348
0.1805
F100000819
0.1805
F100000225
0.1467
F100000313
F100000371
0.1875
N200000557
0.2309
F100000838
0.1741
F100001310
0.1452
F100001035
0.2386
F100001122
0.1686
F100001000
0.149
0.1764
F100000957
0.417
0.2077
F100000037
0.2403
F100000104
F100000363
0.1565
F100000682
0.1868
F100000854
0.2555
0.179
F100000042
0.2153
0.1609
F100000773
F100000950
F100000511
0.483
0.302
F100001044
F100000640
0.1821
0.154
F100001245
0.1694
F100000395
0.2792
F100000337
0.1872
F100000426
0.1771
F100000612
0.3702

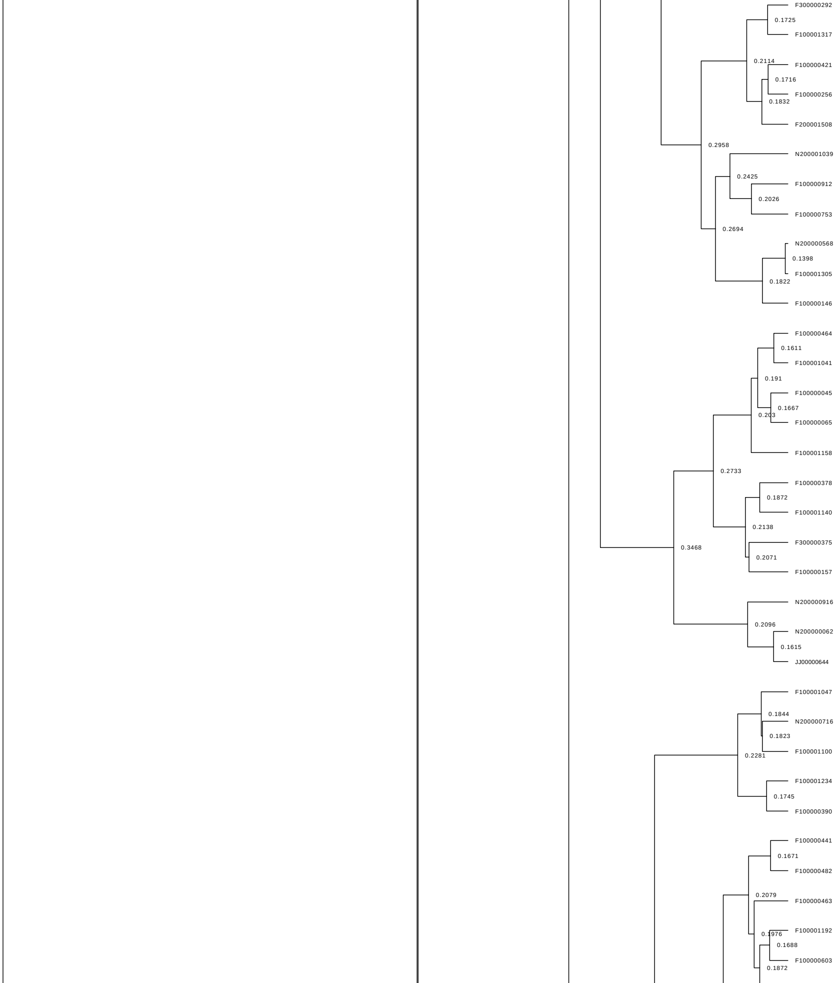

F300000292
0.1725
F100001317
0.2114
F100000421
0.1716
F100000256
0.1832
F200001508
0.2958
N200001039
0.2425
F100000912
0.2026
F100000753
0.2694
N200000568
0.1398
F100001305
0.1822
F100000146
F100000464
0.1611
F100001041
0.191
F100000045
0.1667
0.203
F100000065
F100001158
0.2733
F100000378
0.1872
F100001140
0.2138
F300000375
0.2071
F100000157
0.3468
N200000916
0.2096
N200000062
0.1615
JJ00000644
F100001047
0.1844
N200000716
0.1823
F100001100
0.2281
F100001234
0.1745
F100000390
F100000441
0.1671
F100000482
0.2079
F100000463
0.1976
F100001192
0.1688
F100000603
0.1872

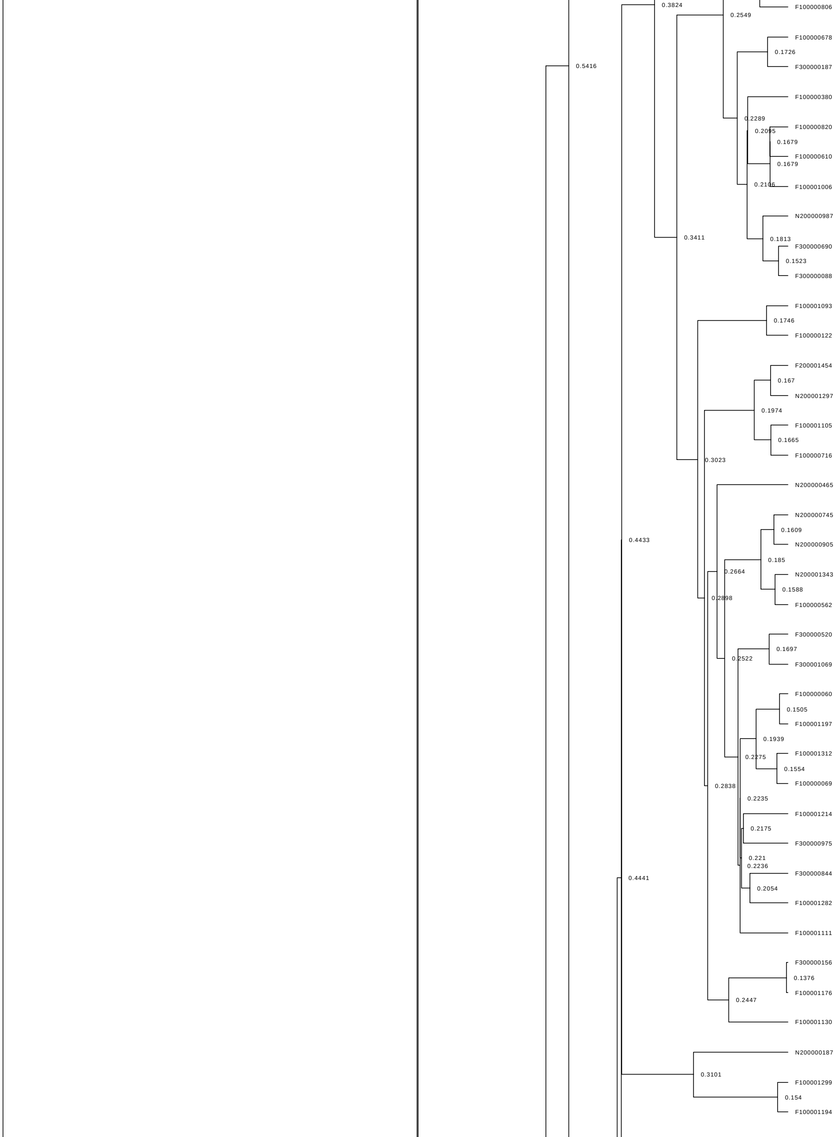
0.3824
F100000806
0.2549
F100000678
0.1726
F300000187
0.5416
F100000380
0.2289
0.2095
F100000820
0.1679
F100000610
0.1679
0.2106
F100001006
N200000987
0.3411
0.1813
F300000690
0.1523
F300000088
F100001093
0.1746
F100000122
F200001454
0.167
N200001297
0.1974
F100001105
0.1665
F100000716
0.3023
N200000465
N200000745
0.1609
0.4433
N200000905
0.185
0.2664
N200001343
0.1588
0.2898
F100000562
F300000520
0.1697
0.2522
F300001069
F100000060
0.1505
F100001197
0.1939
F100001312
0.2275
0.1554
F100000069
0.2838
0.2235
F100001214
0.2175
F300000975
0.221
0.2236
F300000844
0.4441
0.2054
F100001282
F100001111
F300000156
0.1376
F100001176
0.2447
F100001130
N200000187
0.3101
F100001299
0.154
F100001194

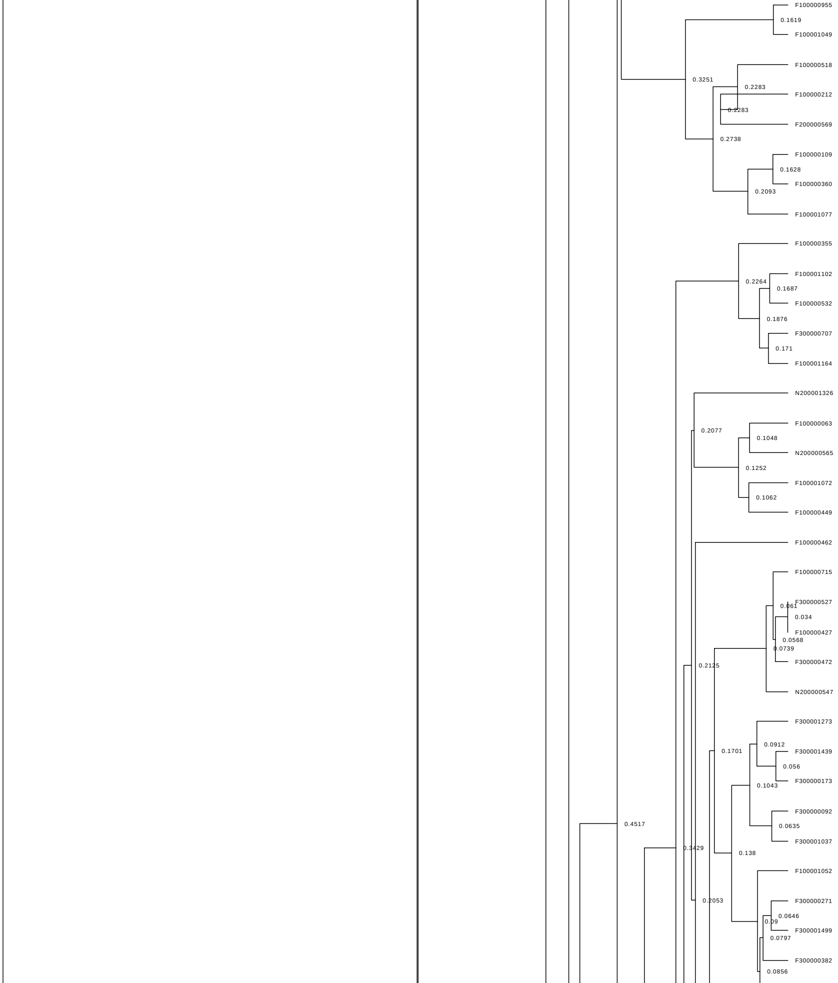

F100000955
0.1619
F100001049
F100000518
0.3251
0.2283
F100000212
0.2283
F200000569
0.2738
F100000109
0.1628
F100000360
0.2093
F100001077
F100000355
F100001102
0.2264
0.1687
F100000532
0.1876
F300000707
0.171
F100001164
N200001326
F100000063
0.2077
0.1048
N200000565
0.1252
F100001072
0.1062
F100000449
F100000462
F100000715
F300000527
0.061
0.034
F100000427
0.0568
0.0739
F300000472
0.2125
N200000547
F300001273
0.0912
0.1701
F300001439
0.056
F300000173
0.1043
F300000092
0.0635
0.4517
F300001037
0.3429
0.138
F100001052
0.2053
F300000271
0.0646
0.09
F300001499
0.0797
F300000382
0.0856

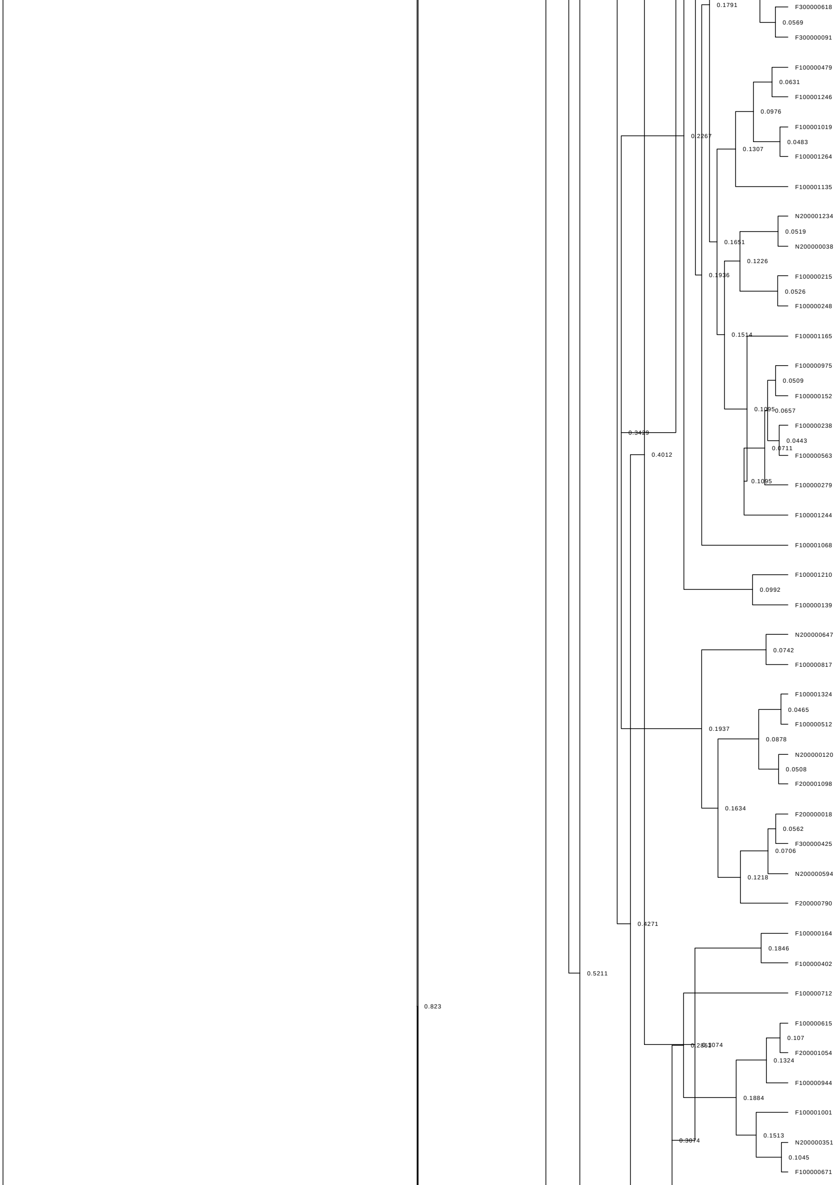

F300000618
0.1791
0.0569
F300000091
F100000479
0.0631
F100001246
0.0976
F100001019
0.2267
0.0483
0.1307
F100001264
F100001135
N200001234
0.0519
0.1651
N200000038
0.1226
0.1936
F100000215
0.0526
F100000248
0.1514
F100001165
F100000975
0.0509
F100000152
0.1095
0.0657
F100000238
0.3429
0.0443
0.4012
0.0711
F100000563
0.1095
F100000279
F100001244
F100001068
F100001210
0.0992
F100000139
N200000647
0.0742
F100000817
F100001324
0.0465
F100000512
0.1937
0.0878
N200000120
0.0508
F200001098
0.1634
F200000018
0.0562
F300000425
0.0706
N200000594
0.1218
F200000790
0.4271
F100000164
0.1846
0.5211
F100000402
F100000712
0.823
F100000615
0.107
0.2861
0.1074
F200001054
0.1324
F100000944
0.1884
F100001001
0.1513
0.3074
N200000351
0.1045
F100000671

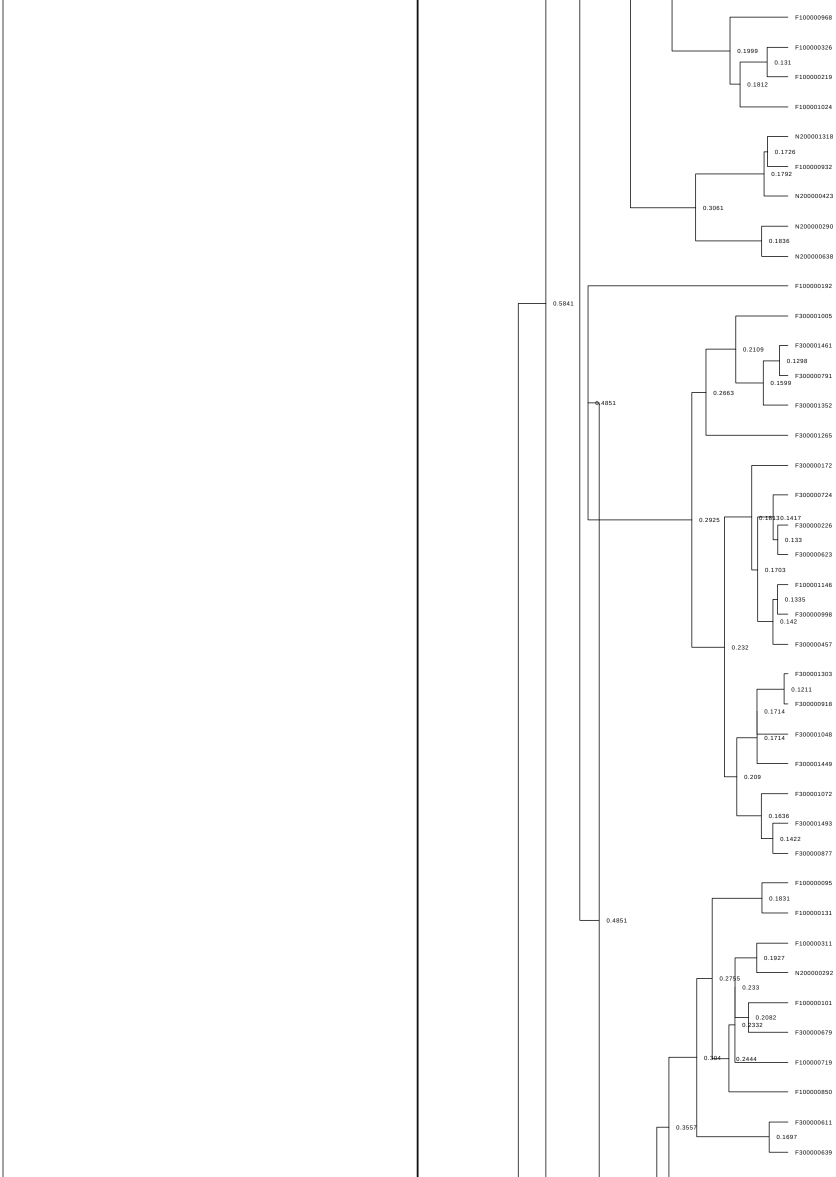
F100000968
0.1999
F100000326
0.131
F100000219
0.1812
F100001024
N200001318
0.1726
F100000932
0.1792
N200000423
0.3061
N200000290
0.1836
N200000638
F100000192
0.5841
F300001005
0.2109
F300001461
0.1298
F300000791
0.1599
0.2663
0.4851
F300001352
F300001265
F300000172
F300000724
0.1813
0.1417
0.2925
F300000226
0.133
F300000623
0.1703
F100001146
0.1335
F300000998
0.142
F300000457
0.232
F300001303
0.1211
F300000918
0.1714
F300001048
0.1714
F300001449
0.209
F300001072
0.1636
F300001493
0.1422
F300000877
F100000095
0.1831
F100000131
0.4851
F100000311
0.1927
N200000292
0.2755
0.233
F100000101
0.2082
0.2332
F300000679
0.304
0.2444
F100000719
F100000850
0.3557
F300000611
0.1697
F300000639

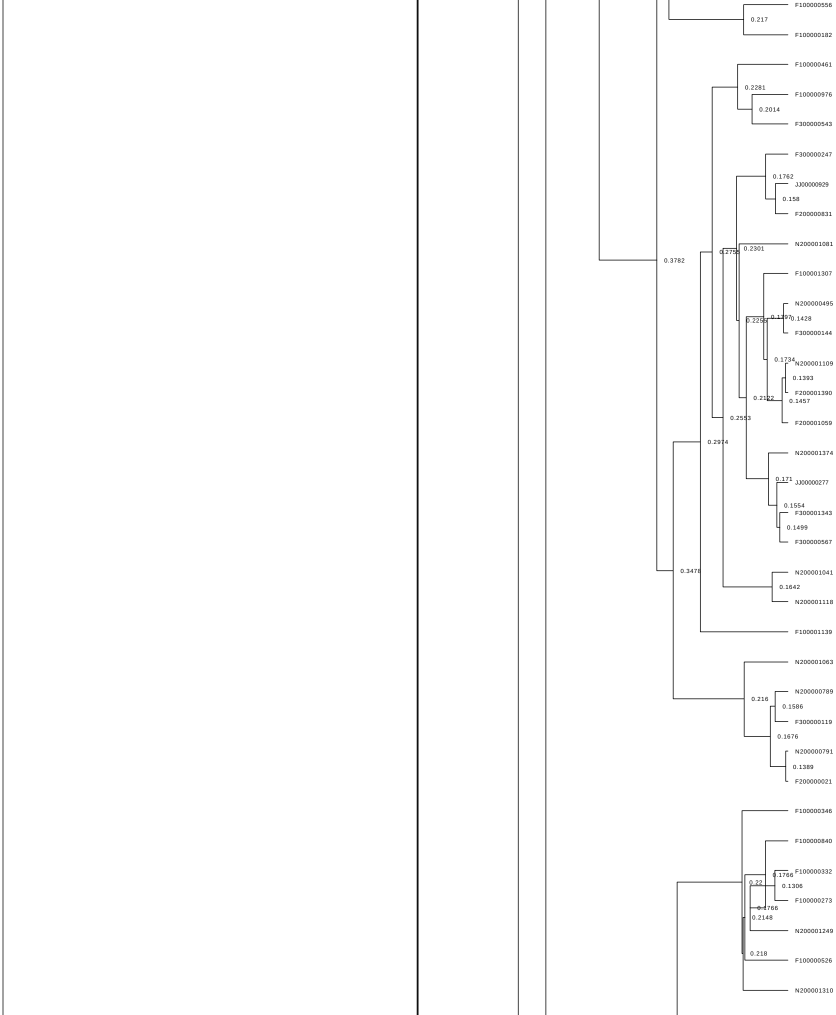

F100000556
0.217
F100000182
F100000461
0.2281
F100000976
0.2014
F300000543
F300000247
0.1762
JJ00000929
0.158
F200000831
N200001081
0.2301
0.2755
0.3782
F100001307
N200000495
0.1797
0.1428
0.2255
F300000144
0.1734
N200001109
0.1393
F200001390
0.2122
0.1457
0.2553
F200001059
0.2974
N200001374
0.171
JJ00000277
0.1554
F300001343
0.1499
F300000567
N200001041
0.3478
0.1642
N200001118
F100001139
N200001063
N200000789
0.216
0.1586
F300000119
0.1676
N200000791
0.1389
F200000021
F100000346
F100000840
F100000332
0.1766
0.22
0.1306
F100000273
0.1766
0.2148
N200001249
0.218
F100000526
N200001310

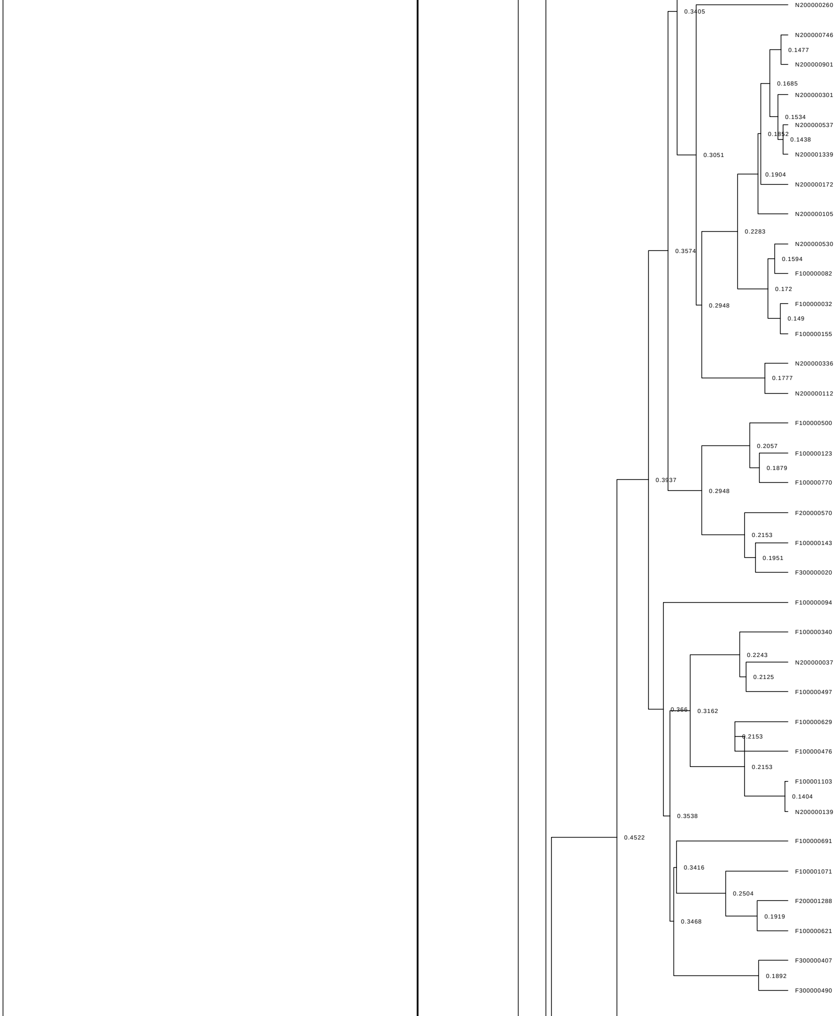

N200000260
0.3405
N200000746
0.1477
N200000901
0.1685
N200000301
0.1534
N200000537
0.1852
0.1438
N200001339
0.3051
0.1904
N200000172
N200000105
0.2283
N200000530
0.1594
F100000082
0.3574
0.172
F100000032
0.2948
0.149
F100000155
N200000336
0.1777
N200000112
F100000500
0.2057
F100000123
0.1879
0.3937
F100000770
0.2948
F200000570
0.2153
F100000143
0.1951
F300000020
F100000094
F100000340
0.2243
N200000037
0.2125
F100000497
0.366
0.3162
F100000629
0.2153
F100000476
0.2153
F100001103
0.1404
N200000139
0.3538
0.4522
F100000691
0.3416
F100001071
0.2504
F200001288
0.1919
0.3468
F100000621
F300000407
0.1892
F300000490

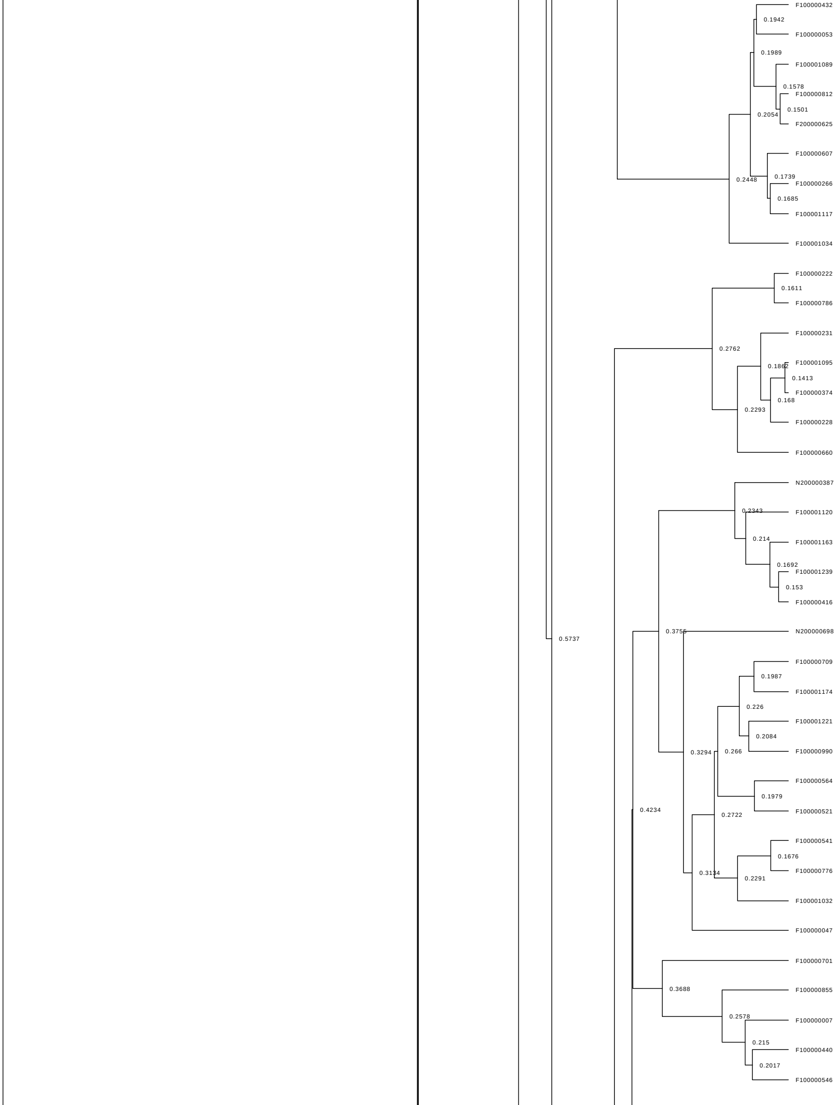
F100000432
0.1942
F100000053
0.1989
F100001089
0.1578
F100000812
0.1501
0.2054
F200000625
F100000607
0.1739
0.2448
F100000266
0.1685
F100001117
F100001034
F100000222
0.1611
F100000786
F100000231
0.2762
F100001095
0.1862
0.1413
F100000374
0.168
0.2293
F100000228
F100000660
N200000387
0.2343
F100001120
0.214
F100001163
0.1692
F100001239
0.153
F100000416
0.3755
N200000698
0.5737
F100000709
0.1987
F100001174
0.226
F100001221
0.2084
0.3294
0.266
F100000990
F100000564
0.1979
F100000521
0.4234
0.2722
F100000541
0.1676
F100000776
0.3134
0.2291
F100001032
F100000047
F100000701
0.3688
F100000855
0.2578
F100000007
0.215
F100000440
0.2017
F100000546

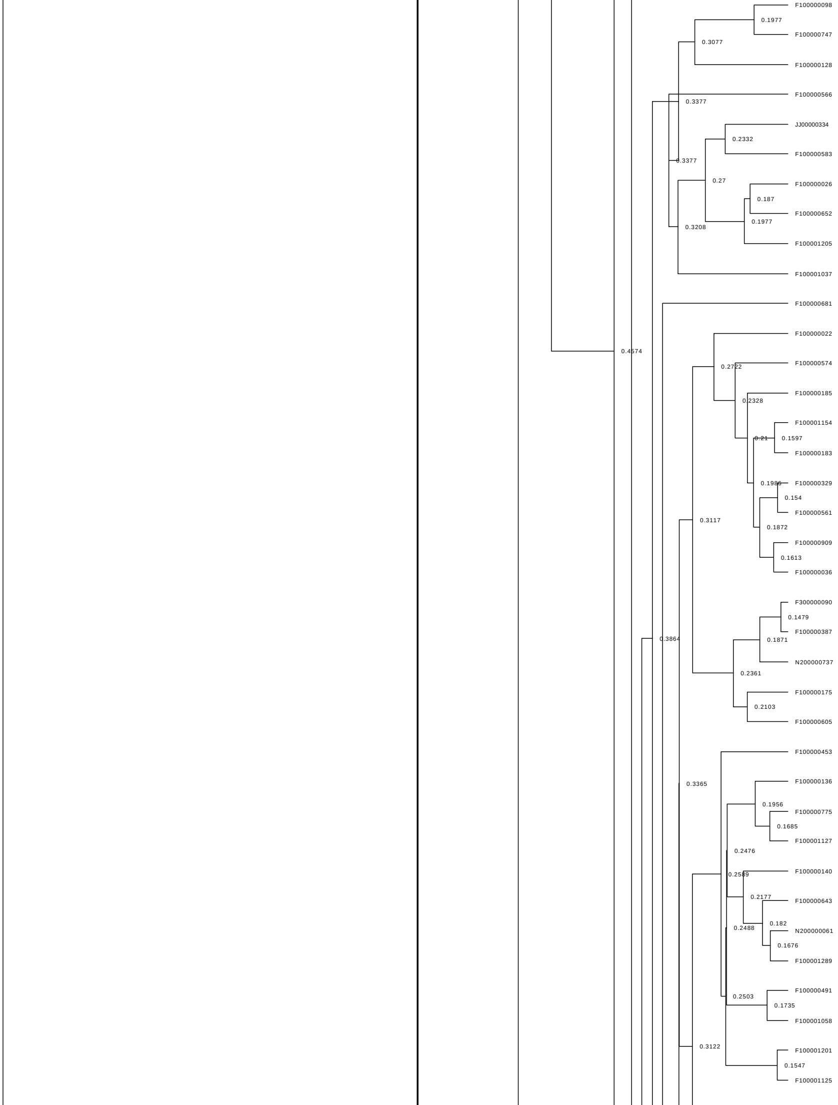
F100000098
0.1977
F100000747
0.3077
F100000128
F100000566
0.3377
JJ00000334
0.2332
F100000583
0.3377
0.27
F100000026
0.187
F100000652
0.1977
0.3208
F100001205
F100001037
F100000681
F100000022
0.4574
0.2722
F100000574
F100000185
0.2328
F100001154
0.21
0.1597
F100000183
0.1986
F100000329
0.154
F100000561
0.3117
0.1872
F100000909
0.1613
F100000036
F300000090
0.1479
F100000387
0.3864
0.1871
N200000737
0.2361
F100000175
0.2103
F100000605
F100000453
0.3365
F100000136
0.1956
F100000775
0.1685
F100001127
0.2476
F100000140
0.2589
0.2177
F100000643
0.182
0.2488
N200000061
0.1676
F100001289
F100000491
0.2503
0.1735
F100001058
0.3122
F100001201
0.1547
F100001125

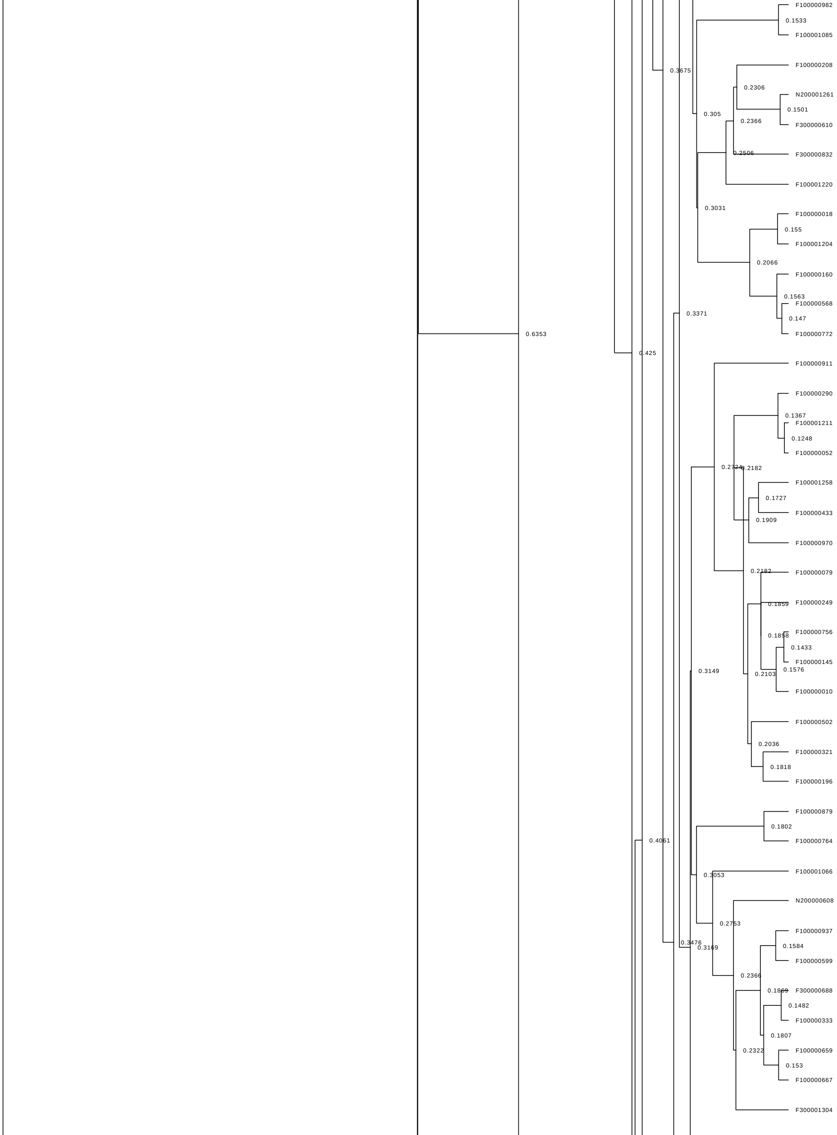
F100000982
0.1533
F100001085
0.3675
F100000208
0.2306
N200001261
0.1501
0.305
0.2366
F300000610
0.2506
F300000832
F100001220
0.3031
F100000018
0.155
F100001204
0.2066
F100000160
0.1563
F100000568
0.147
0.3371
0.6353
F100000772
0.425
F100000911
F100000290
0.1367
F100001211
0.1248
F100000052
0.2724
0.2182
F100001258
0.1727
F100000433
0.1909
F100000970
0.2182
F100000079
0.1859
F100000249
0.1858
F100000756
0.1433
F100000145
0.1576
0.3149
0.2103
F100000010
F100000502
0.2036
F100000321
0.1818
F100000196
F100000879
0.1802
0.4061
F100000764
0.3053
F100001066
N200000608
0.2753
F100000937
0.3476
0.3169
0.1584
F100000599
0.2366
0.1869
F300000688
0.1482
F100000333
0.1807
0.2322
F100000659
0.153
F100000667
F300001304

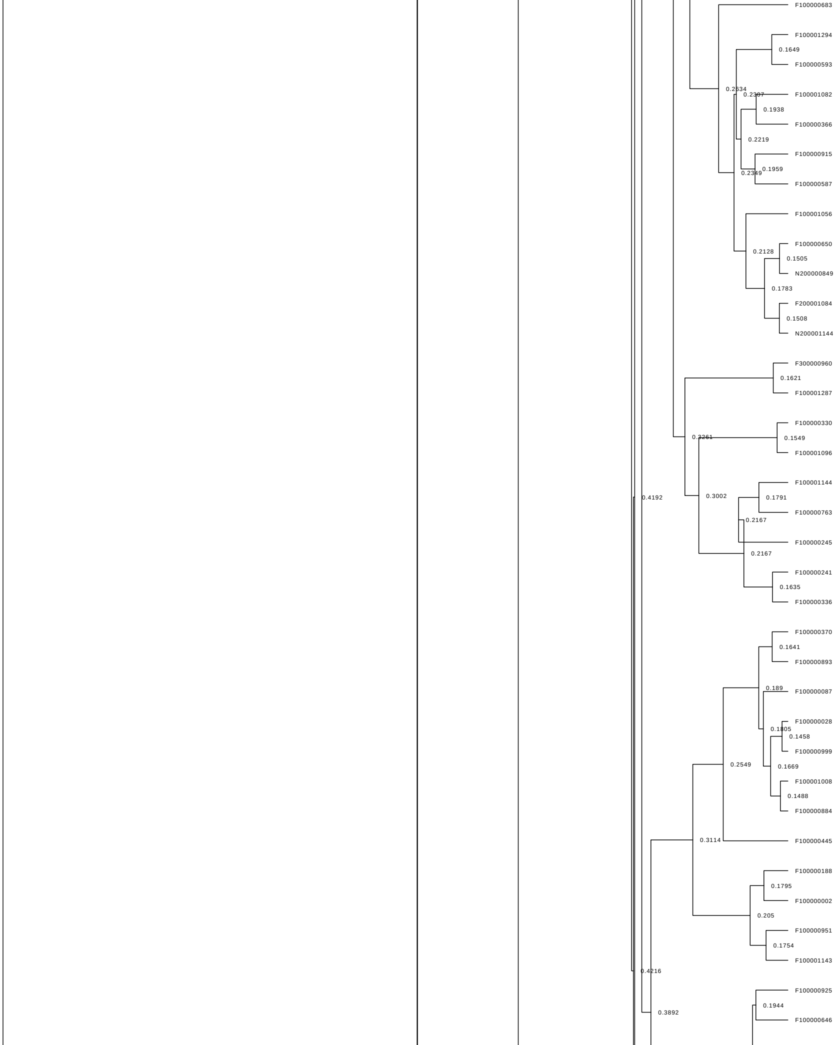
F100000683
F100001294
0.1649
F100000593
0.2634
0.2307
F100001082
0.1938
F100000366
0.2219
F100000915
0.2349
0.1959
F100000587
F100001056
F100000650
0.2128
0.1505
N200000849
0.1783
F200001084
0.1508
N200001144
F300000960
0.1621
F100001287
F100000330
0.3261
0.1549
F100001096
F100001144
0.4192
0.3002
0.1791
F100000763
0.2167
F100000245
0.2167
F100000241
0.1635
F100000336
F100000370
0.1641
F100000893
0.189
F100000087
F100000028
0.1805
0.1458
F100000999
0.2549
0.1669
F100001008
0.1488
F100000884
0.3114
F100000445
F100000188
0.1795
F100000002
0.205
F100000951
0.1754
F100001143
0.4216
F100000925
0.1944
0.3892
F100000646

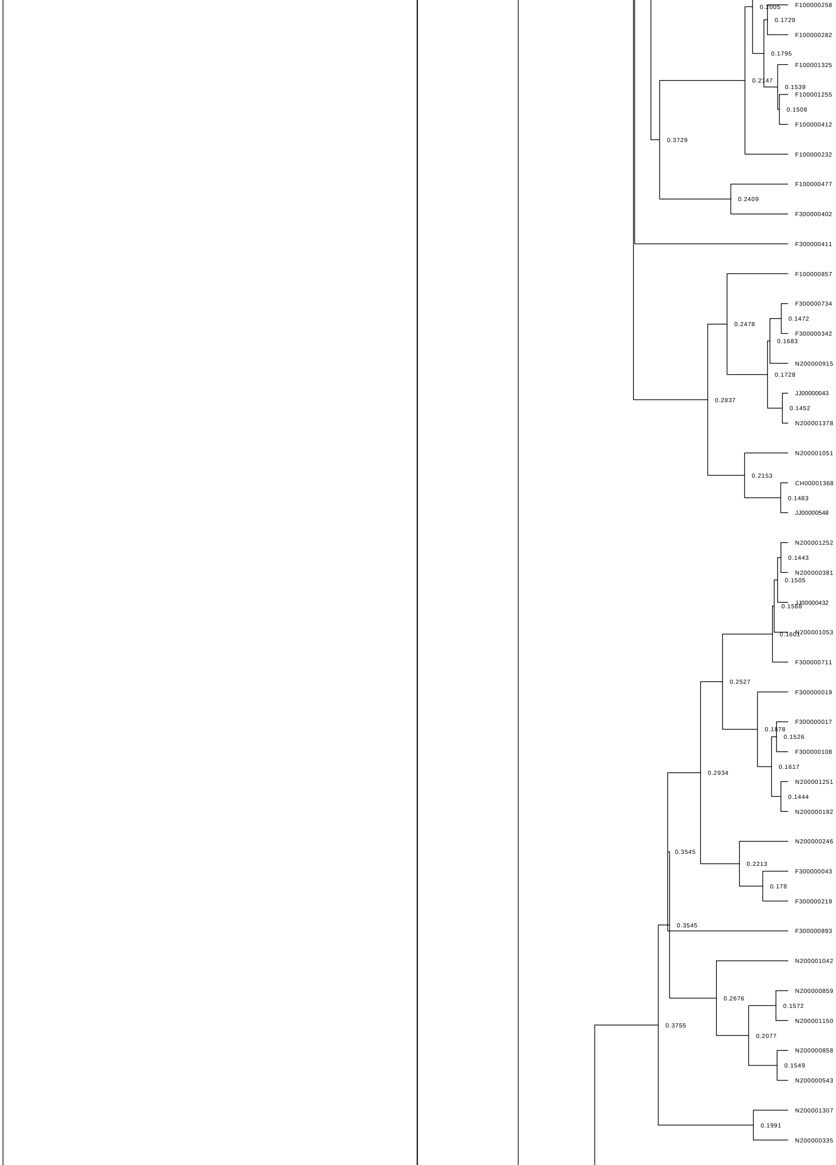
F100000258
0.2005
0.1729
F100000282
0.1795
F100001325
0.2147
0.1539
F100001255
0.1508
F100000412
0.3729
F100000232
F100000477
0.2409
F300000402
F300000411
F100000857
F300000734
0.1472
0.2478
F300000342
0.1683
N200000915
0.1728
JJ00000043
0.2837
0.1452
N200001378
N200001051
0.2153
CH00001368
0.1483
JJ00000548
N200001252
0.1443
N200000381
0.1505
JJ00000432
0.1568
N200001053
0.1601
F300000711
0.2527
F300000019
F300000017
0.1878
0.1526
F300000108
0.1617
0.2934
N200001251
0.1444
N200000192
N200000246
0.3545
0.2213
F300000043
0.178
F300000219
0.3545
F300000893
N200001042
N200000859
0.2676
0.1572
N200001150
0.3755
0.2077
N200000858
0.1549
N200000543
N200001307
0.1991
N200000335

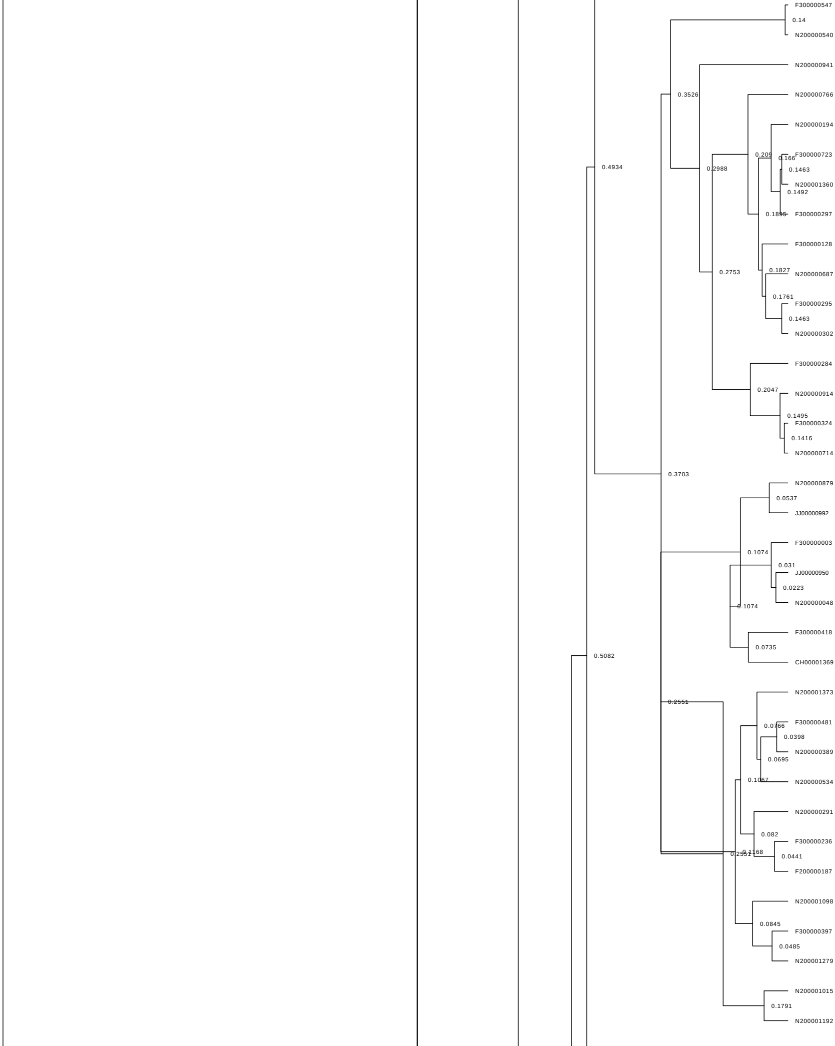
F300000547
0.14
N200000540
N200000941
0.3526
N200000766
N200000194
0.209
0.166
F300000723
0.1463
0.4934
0.2988
N200001360
0.1492
0.1895
F300000297
F300000128
0.1827
0.2753
N200000687
0.1761
F300000295
0.1463
N200000302
F300000284
0.2047
N200000914
0.1495
F300000324
0.1416
N200000714
0.3703
N200000879
0.0537
JJ00000992
F300000003
0.1074
0.031
JJ00000950
0.0223
N200000048
0.1074
F300000418
0.0735
0.5082
CH00001369
N200001373
0.2551
F300000481
0.0766
0.0398
N200000389
0.0695
0.1067
N200000534
N200000291
0.082
F300000236
0.2551
0.1168
0.0441
F200000187
N200001098
0.0845
F300000397
0.0485
N200001279
N200001015
0.1791
N200001192

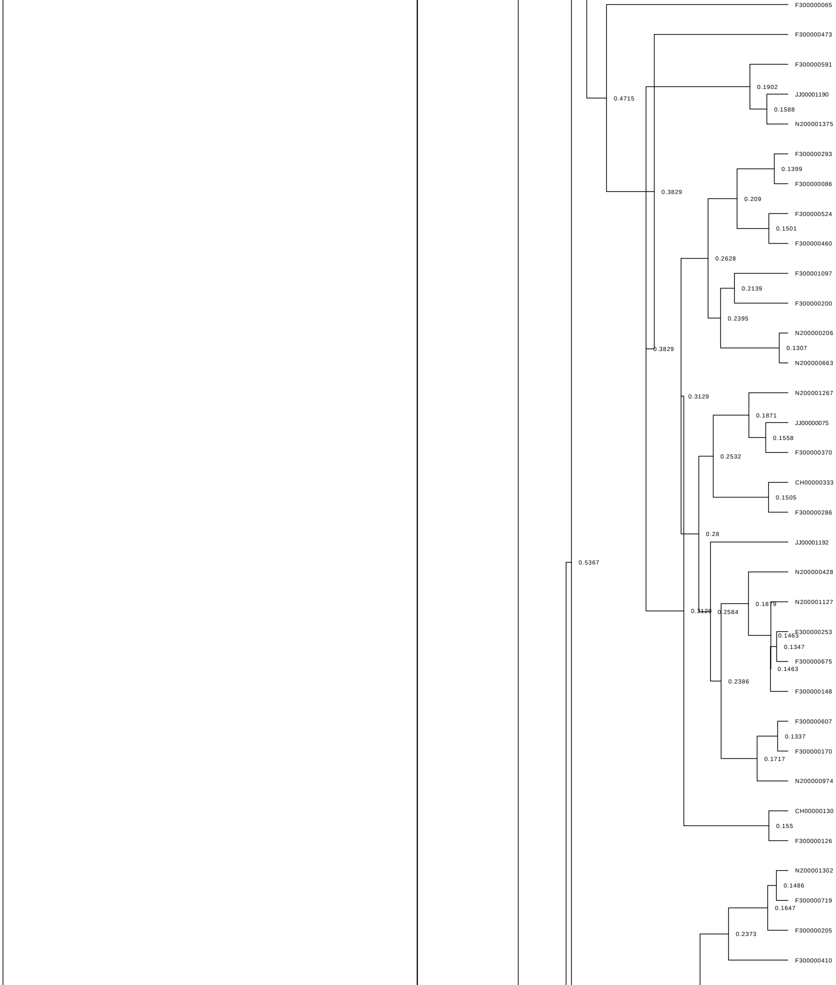
F300000065
F300000473
F300000591
0.1902
JJ00001190
0.1588
N200001375
0.4715
F300000293
0.1399
F300000086
0.3829
0.209
F300000524
0.1501
F300000460
0.2628
F300001097
0.2139
F300000200
0.2395
N200000206
0.1307
0.3829
N200000663
N200001267
0.3129
0.1871
JJ00000075
0.1558
F300000370
0.2532
CH00000333
0.1505
F300000286
0.28
JJ00001192
0.5367
N200000428
N200001127
0.1879
0.3129
0.2584
F300000253
0.1463
0.1347
F300000675
0.1463
0.2386
F300000148
F300000607
0.1337
F300000170
0.1717
N200000974
CH00000130
0.155
F300000126
N200001302
0.1486
F300000719
0.1647
0.2373
F300000205
F300000410

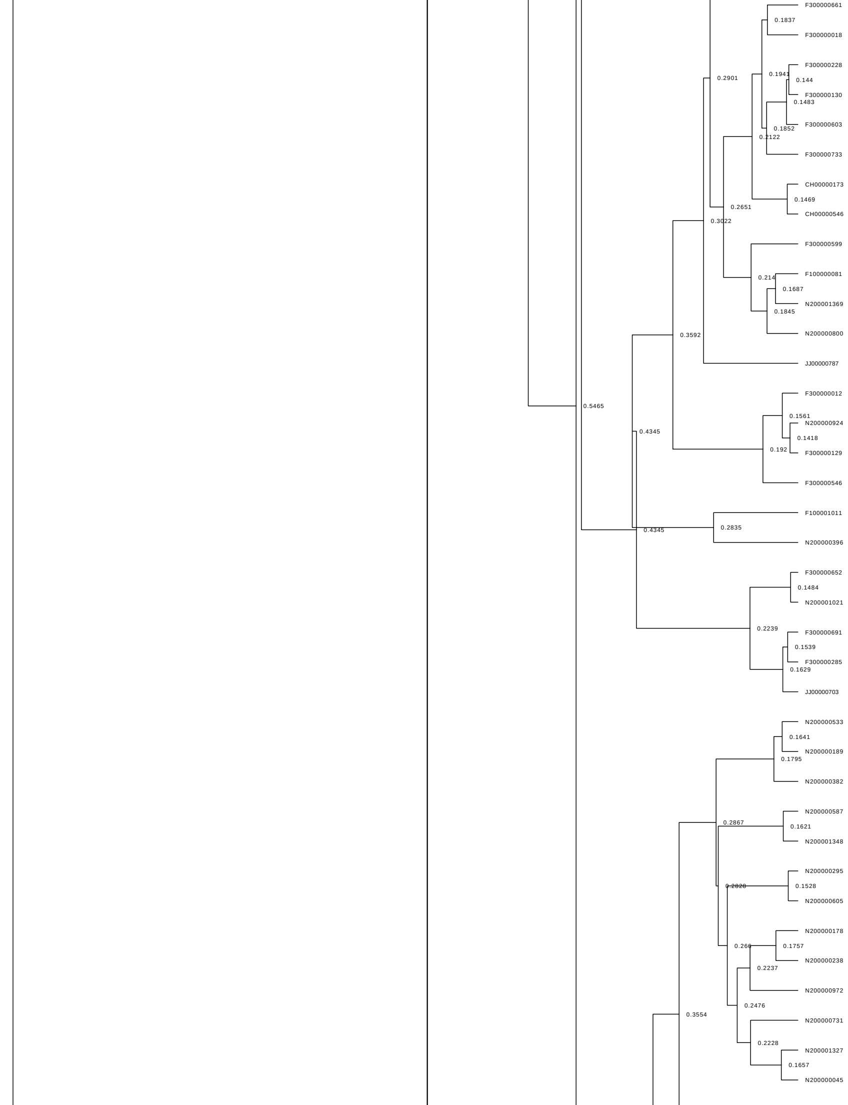
F300000661
0.1837
F300000018
F300000228
0.1941
0.144
0.2901
F300000130
0.1483
F300000603
0.1852
0.2122
F300000733
CH00000173
0.1469
0.2651
CH00000546
0.3022
F300000599
0.214
F100000081
0.1687
N200001369
0.1845
N200000800
0.3592
JJ00000787
F300000012
0.5465
0.1561
N200000924
0.4345
0.1418
0.192
F300000129
F300000546
F100001011
0.2835
0.4345
N200000396
F300000652
0.1484
N200001021
0.2239
F300000691
0.1539
F300000285
0.1629
JJ00000703
N200000533
0.1641
N200000189
0.1795
N200000382
N200000587
0.2867
0.1621
N200001348
N200000295
0.2828
0.1528
N200000605
N200000178
0.266
0.1757
N200000238
0.2237
N200000972
0.2476
0.3554
N200000731
0.2228
N200001327
0.1657
N200000045

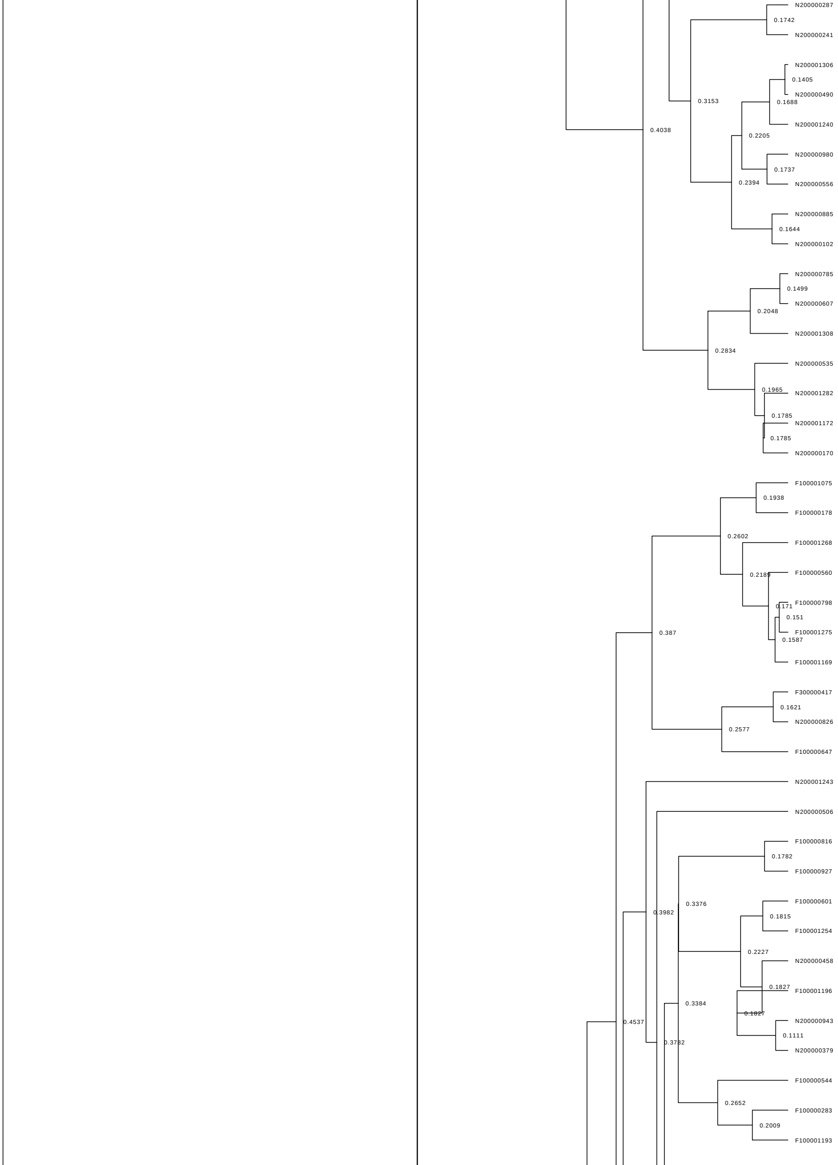

N200000287
0.1742
N200000241
N200001306
0.1405
N200000490
0.1688
N200001240
0.2205
N200000980
0.1737
N200000556
0.2394
0.3153
0.4038
N200000885
0.1644
N200000102
N200000785
0.1499
N200000607
0.2048
N200001308
0.2834
N200000535
0.1965
N200001282
0.1785
N200001172
0.1785
N200000170
F100001075
0.1938
F100000178
0.2602
F100001268
F100000560
0.2189
F100000798
0.171
0.151
F100001275
0.1587
F100001169
0.387
F300000417
0.1621
N200000826
0.2577
F100000647
N200001243
N200000506
F100000816
0.1782
F100000927
0.3376
F100000601
0.1815
F100001254
0.3982
0.2227
N200000458
0.1827
F100001196
0.3384
0.1827
N200000943
0.4537
0.1111
0.3782
N200000379
F100000544
0.2652
F100000283
0.2009
F100001193

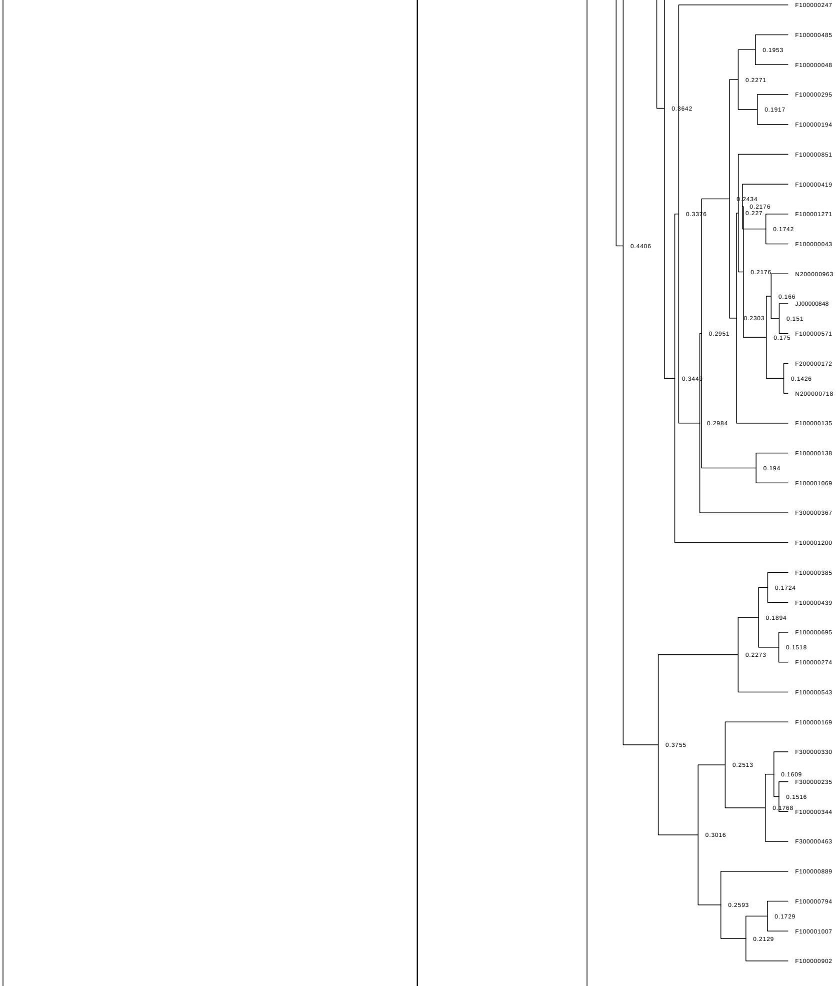

F100000247
F100000485
0.1953
F100000048
0.2271
F100000295
0.3642
0.1917
F100000194
F100000851
F100000419
0.2434
0.2176
0.3376
0.227
F100001271
0.1742
0.4406
F100000043
0.2176
N200000963
0.166
JJ00000848
0.2303
0.151
0.2951
F100000571
0.175
F200000172
0.1426
0.3449
N200000718
0.2984
F100000135
F100000138
0.194
F100001069
F300000367
F100001200
F100000385
0.1724
F100000439
0.1894
F100000695
0.1518
0.2273
F100000274
F100000543
F100000169
0.3755
F300000330
0.2513
0.1609
F300000235
0.1516
0.1768
F100000344
0.3016
F300000463
F100000889
F100000794
0.2593
0.1729
F100001007
0.2129
F100000902

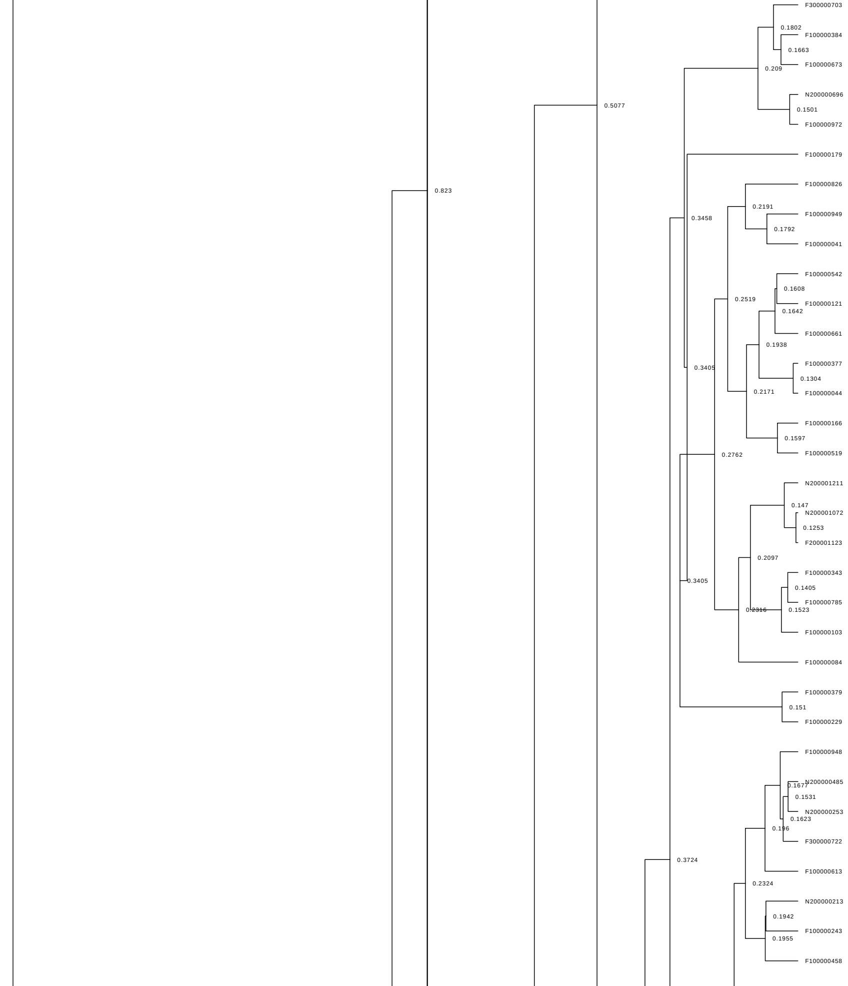
F300000703
0.1802
F100000384
0.1663
F100000673
0.209
N200000696
0.5077
0.1501
F100000972
F100000179
0.823
F100000826
0.2191
F100000949
0.3458
0.1792
F100000041
F100000542
0.1608
F100000121
0.2519
0.1642
F100000661
0.1938
F100000377
0.3405
0.1304
0.2171
F100000044
F100000166
0.1597
F100000519
0.2762
N200001211
0.147
N200001072
0.1253
F200001123
0.2097
F100000343
0.1405
0.3405
F100000785
0.2316
0.1523
F100000103
F100000084
F100000379
0.151
F100000229
F100000948
N200000485
0.1677
0.1531
N200000253
0.1623
0.196
F300000722
0.3724
F100000613
0.2324
N200000213
0.1942
F100000243
0.1955
F100000458

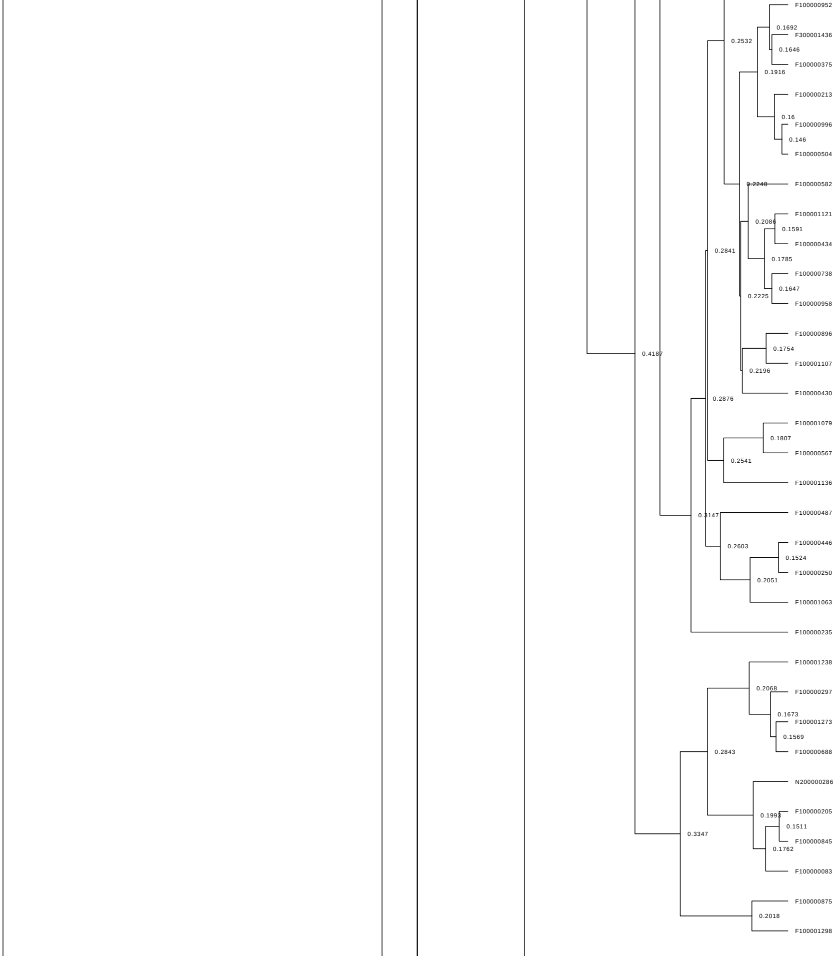
F100000952
0.1692
F300001436
0.2532
0.1646
F100000375
0.1916
F100000213
0.16
F100000996
0.146
F100000504
0.2248
F100000582
F100001121
0.2086
0.1591
F100000434
0.2841
0.1785
F100000738
0.1647
0.2225
F100000958
F100000896
0.1754
0.4187
F100001107
0.2196
F100000430
0.2876
F100001079
0.1807
F100000567
0.2541
F100001136
0.3147
F100000487
0.2603
F100000446
0.1524
F100000250
0.2051
F100001063
F100000235
F100001238
0.2068
F100000297
0.1673
F100001273
0.1569
0.2843
F100000688
N200000286
0.1993
F100000205
0.1511
0.3347
F100000845
0.1762
F100000083
F100000875
0.2018
F100001298

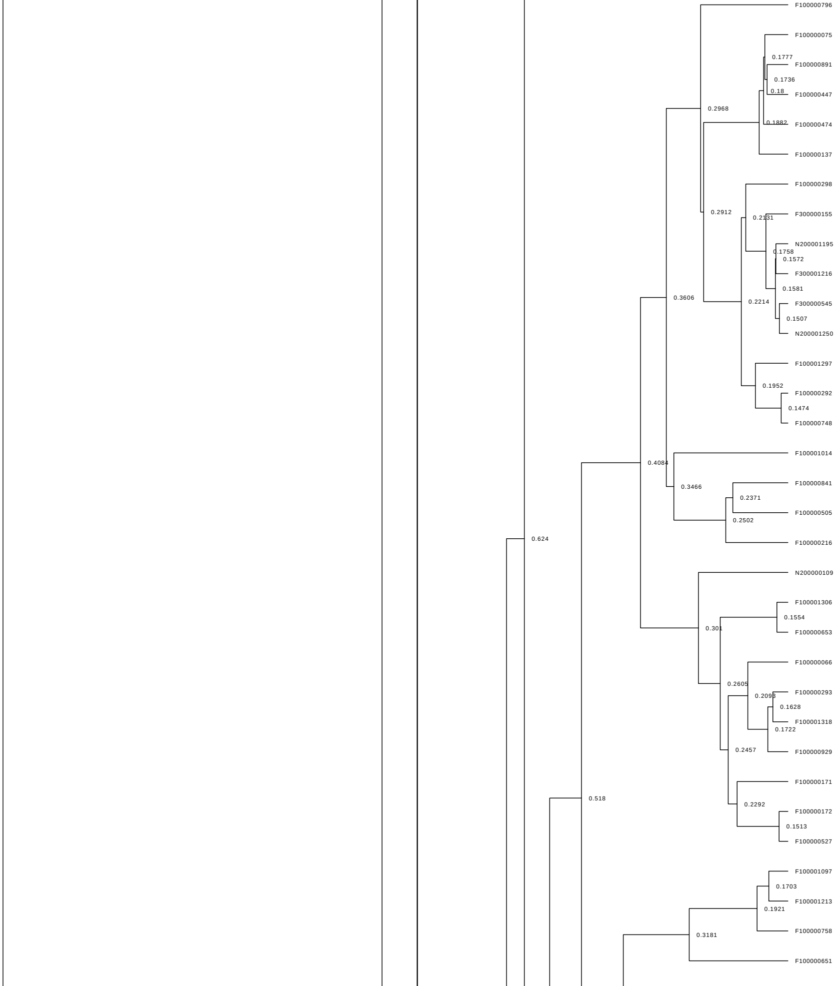

F100000796
F100000075
0.1777
F100000891
0.1736
0.18
F100000447
0.2968
0.1882
F100000474
F100000137
F100000298
0.2912
0.2131
F300000155
N200001195
0.1758
0.1572
F300001216
0.1581
0.3606
0.2214
F300000545
0.1507
N200001250
F100001297
0.1952
F100000292
0.1474
F100000748
F100001014
0.4084
0.3466
F100000841
0.2371
F100000505
0.2502
0.624
F100000216
N200000109
F100001306
0.1554
0.301
F100000653
F100000066
0.2605
0.2093
F100000293
0.1628
F100001318
0.1722
0.2457
F100000929
F100000171
0.518
0.2292
F100000172
0.1513
F100000527
F100001097
0.1703
F100001213
0.1921
0.3181
F100000758
F100000651

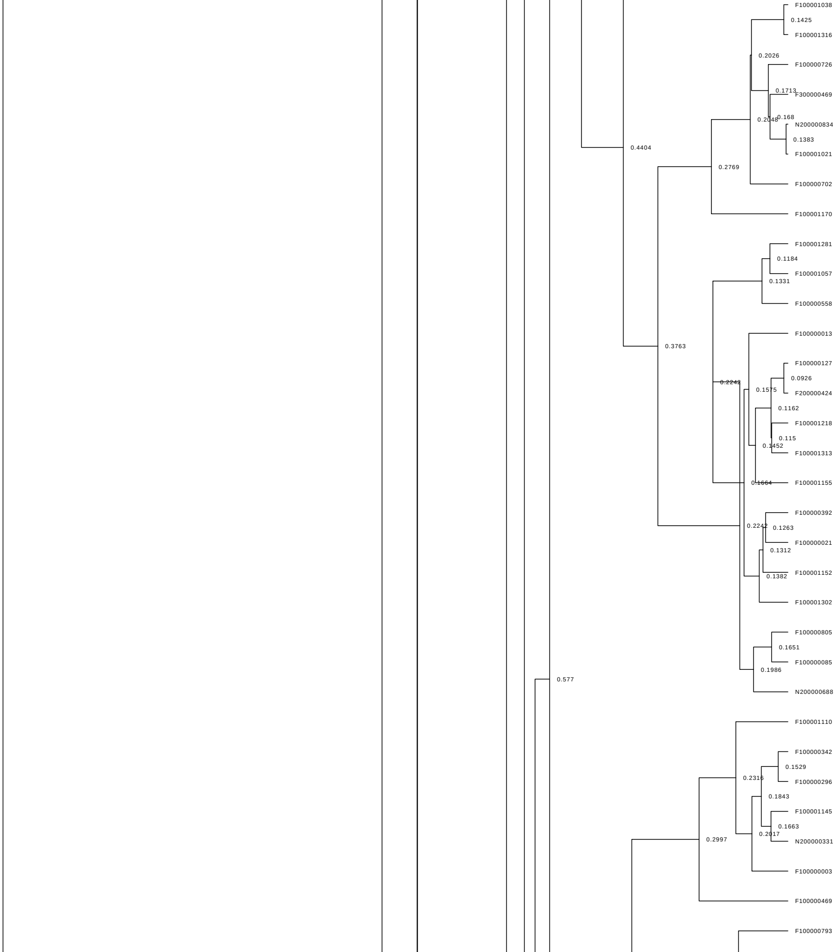
F100001038
0.1425
F100001316
0.2026
F100000726
0.1713
F300000469
0.2048
0.168
N200000834
0.1383
F100001021
0.4404
0.2769
F100000702
F100001170
F100001281
0.1184
F100001057
0.1331
F100000558
F100000013
0.3763
F100000127
0.0926
0.2242
0.1575
F200000424
0.1162
F100001218
0.115
0.1452
F100001313
0.1664
F100001155
F100000392
0.2242
0.1263
F100000021
0.1312
F100001152
0.1382
F100001302
F100000805
0.1651
F100000085
0.1986
0.577
N200000688
F100001110
F100000342
0.1529
0.2316
F100000296
0.1843
F100001145
0.1663
0.2017
N200000331
0.2997
F100000003
F100000469
F100000793

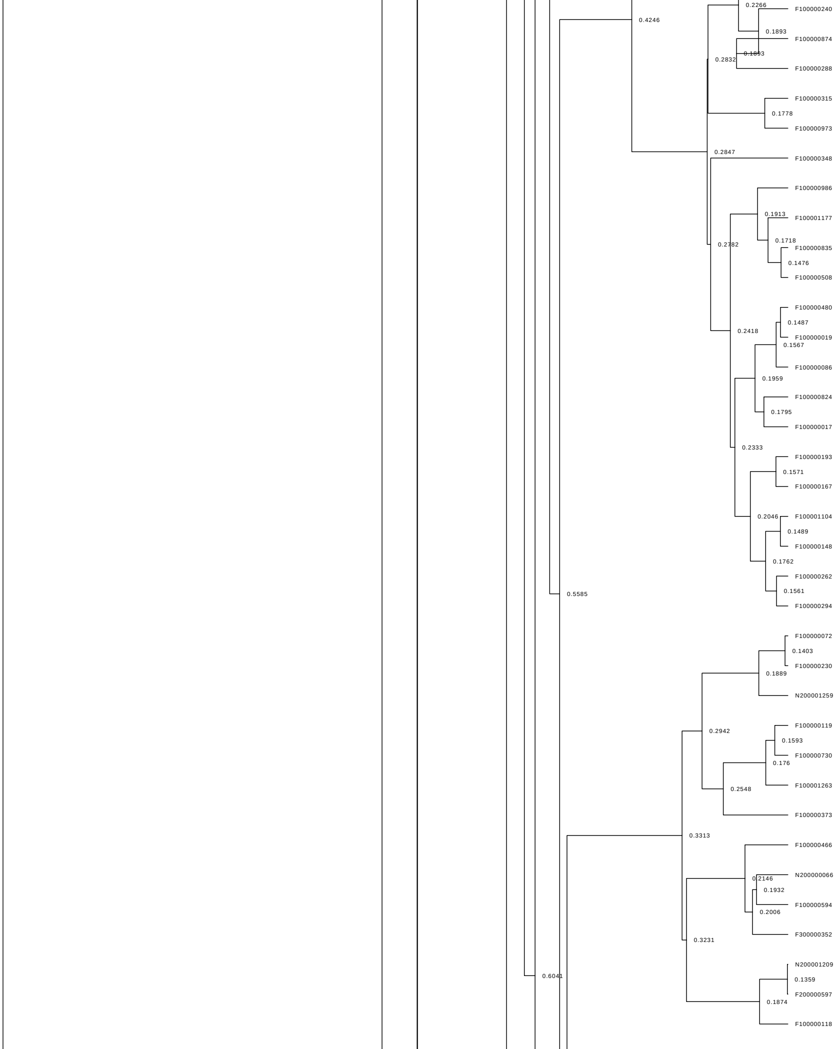
0.2266
F100000240
0.4246
0.1893
F100000874
0.1893
0.2832
F100000288
F100000315
0.1778
F100000973
0.2847
F100000348
F100000986
0.1913
F100001177
0.1718
0.2782
F100000835
0.1476
F100000508
F100000480
0.1487
0.2418
F100000019
0.1567
F100000086
0.1959
F100000824
0.1795
F100000017
0.2333
F100000193
0.1571
F100000167
0.2046
F100001104
0.1489
F100000148
0.1762
F100000262
0.5585
0.1561
F100000294
F100000072
0.1403
F100000230
0.1889
N200001259
F100000119
0.2942
0.1593
F100000730
0.176
F100001263
0.2548
F100000373
0.3313
F100000466
N200000066
0.2146
0.1932
F100000594
0.2006
F300000352
0.3231
N200001209
0.6041
0.1359
F200000597
0.1874
F100000118

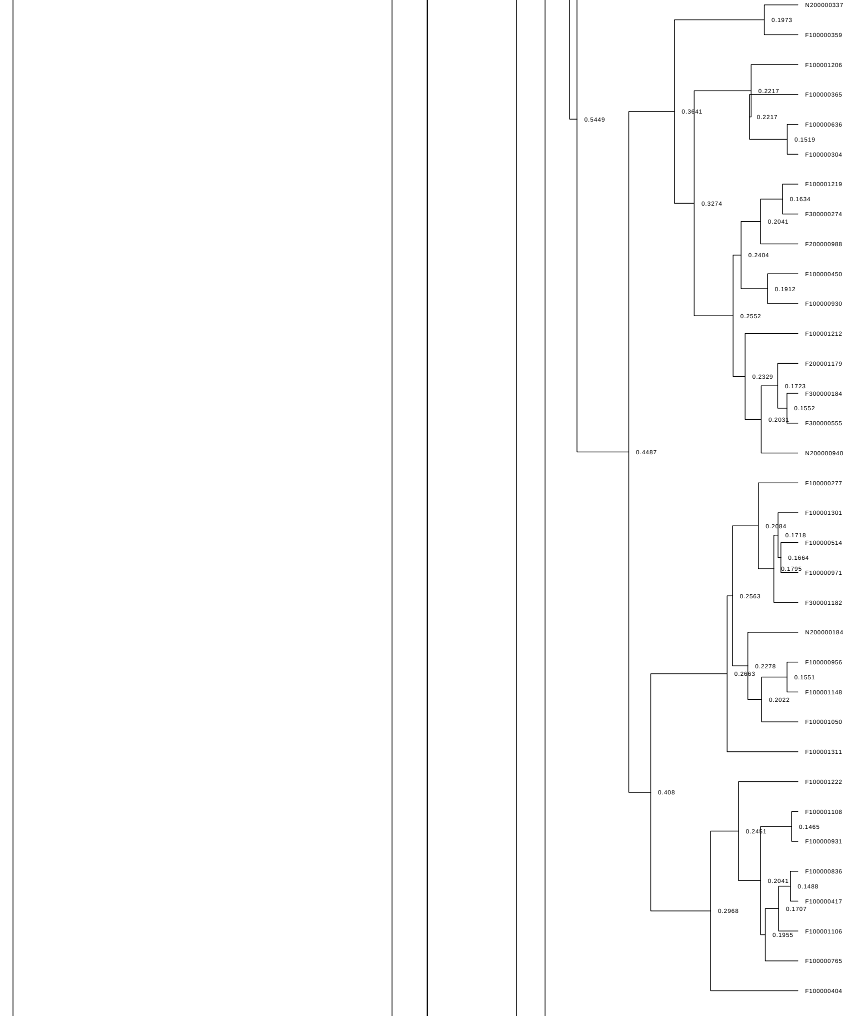
N200000337
0.1973
F100000359
F100001206
0.2217
F100000365
0.5449
0.3641
0.2217
F100000636
0.1519
F100000304
F100001219
0.1634
0.3274
F300000274
0.2041
F200000988
0.2404
F100000450
0.1912
F100000930
0.2552
F100001212
F200001179
0.2329
0.1723
F300000184
0.1552
0.2031
F300000555
0.4487
N200000940
F100000277
F100001301
0.2084
0.1718
F100000514
0.1664
0.1795
F100000971
0.2563
F300001182
N200000184
F100000956
0.2278
0.2663
0.1551
F100001148
0.2022
F100001050
F100001311
F100001222
0.408
F100001108
0.1465
0.2451
F100000931
F100000836
0.2041
0.1488
F100000417
0.1707
0.2968
F100001106
0.1955
F100000765
F100000404

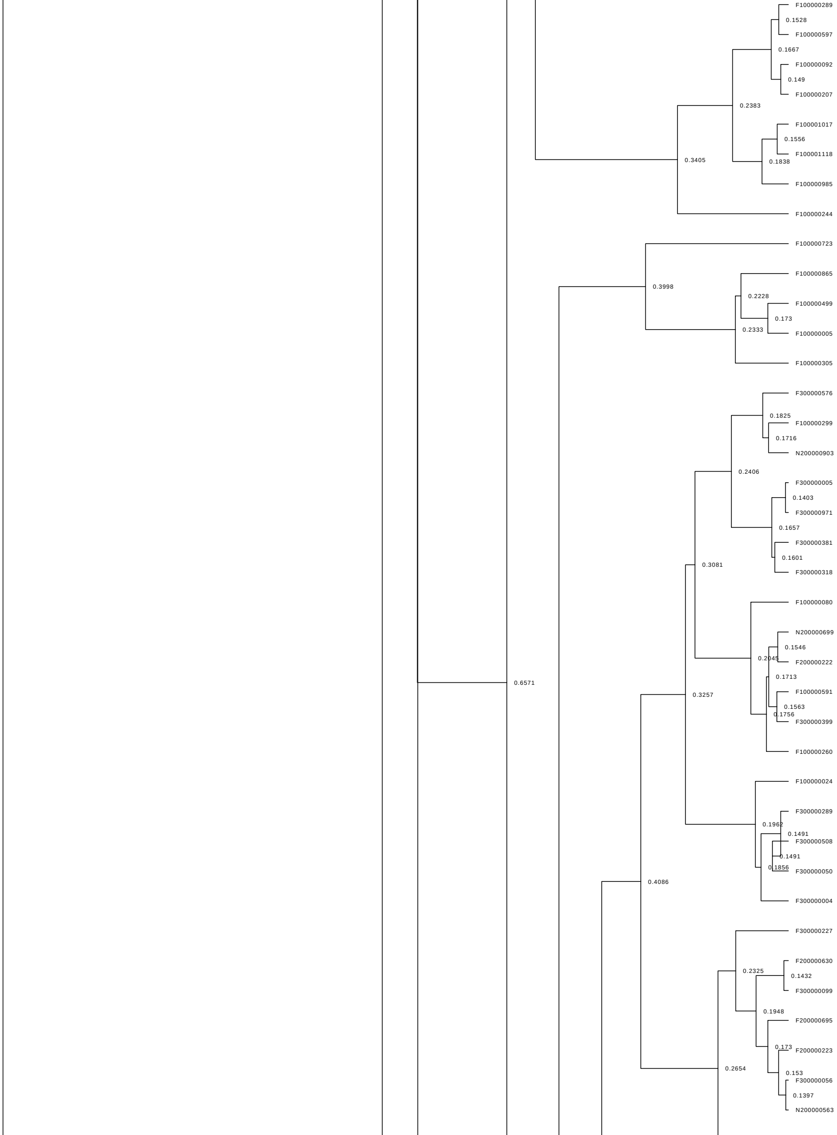
F100000289
0.1528
F100000597
0.1667
F100000092
0.149
F100000207
0.2383
F100001017
0.1556
F100001118
0.3405
0.1838
F100000985
F100000244
F100000723
F100000865
0.3998
0.2228
F100000499
0.173
0.2333
F100000005
F100000305
F300000576
0.1825
F100000299
0.1716
N200000903
0.2406
F300000005
0.1403
F300000971
0.1657
F300000381
0.1601
F300000318
0.3081
F100000080
N200000699
0.1546
0.2045
F200000222
0.1713
0.6571
F100000591
0.3257
0.1563
0.1756
F300000399
F100000260
F100000024
F300000289
0.1962
0.1491
F300000508
0.1491
0.1856
F300000050
0.4086
F300000004
F300000227
F200000630
0.2325
0.1432
F300000099
0.1948
F200000695
0.173
F200000223
0.2654
0.153
F300000056
0.1397
N200000563

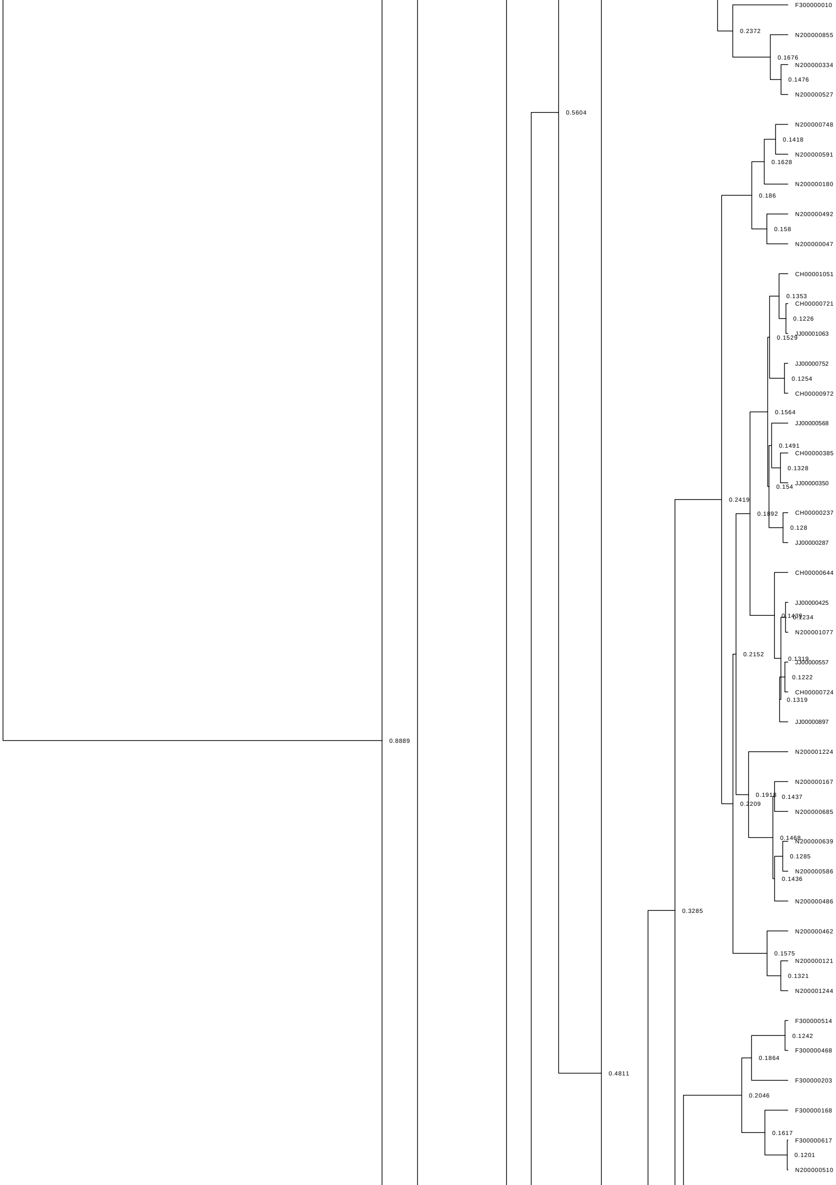
F300000010
0.2372
N200000855
0.1676
N200000334
0.1476
N200000527
0.5604
N200000748
0.1418
N200000591
0.1628
N200000180
0.186
N200000492
0.158
N200000047
CH00001051
0.1353
CH00000721
0.1226
JJ00001063
0.1529
JJ00000752
0.1254
CH00000972
0.1564
JJ00000568
0.1491
CH00000385
0.1328
JJ00000350
0.154
0.2419
CH00000237
0.1892
0.128
JJ00000287
CH00000644
JJ00000425
0.1439
0.1234
N200001077
0.2152
0.1319
JJ00000557
0.1222
CH00000724
0.1319
JJ00000897
0.8889
N200001224
N200000167
0.1918
0.1437
0.2209
N200000685
0.1468
N200000639
0.1285
N200000586
0.1436
N200000486
0.3285
N200000462
0.1575
N200000121
0.1321
N200001244
F300000514
0.1242
F300000468
0.1864
0.4811
F300000203
0.2046
F300000168
0.1617
F300000617
0.1201
N200000510

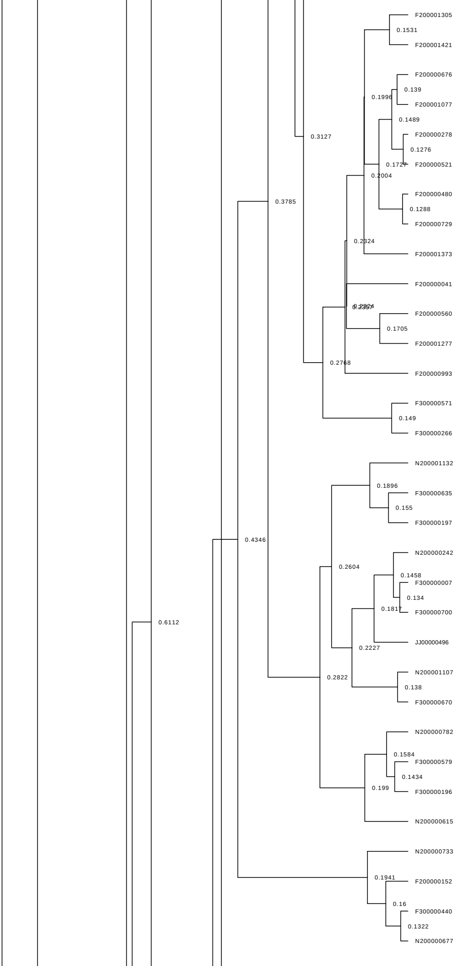
F200001305
0.1531
F200001421
F200000676
0.139
0.1996
F200001077
0.1489
F200000278
0.3127
0.1276
0.1727
F200000521
0.2004
0.3785
F200000480
0.1288
F200000729
0.2324
F200001373
F200000041
0.2324
0.2357
F200000560
0.1705
F200001277
0.2768
F200000993
F300000571
0.149
F300000266
N200001132
0.1896
F300000635
0.155
F300000197
0.4346
N200000242
0.2604
0.1458
F300000007
0.134
0.1817
F300000700
0.6112
JJ00000496
0.2227
N200001107
0.2822
0.138
F300000670
N200000782
0.1584
F300000579
0.1434
0.199
F300000196
N200000615
N200000733
0.1941
F200000152
0.16
F300000440
0.1322
N200000677

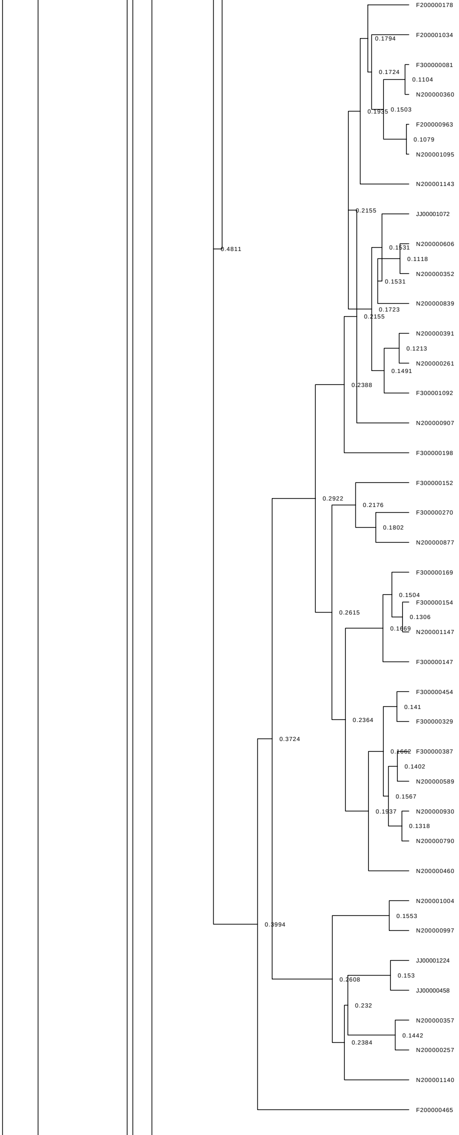
F200000178
0.1794
F200001034
0.1724
F300000081
0.1104
N200000360
0.1935
0.1503
F200000963
0.1079
N200001095
N200001143
0.2155
JJ00001072
0.4811
0.1531
N200000606
0.1118
N200000352
0.1531
N200000839
0.1723
0.2155
N200000391
0.1213
N200000261
0.1491
0.2388
F300001092
N200000907
F300000198
F300000152
0.2922
0.2176
F300000270
0.1802
N200000877
F300000169
0.1504
F300000154
0.1306
0.2615
0.1669
N200001147
F300000147
F300000454
0.141
F300000329
0.2364
0.3724
0.1662
F300000387
0.1402
N200000589
0.1567
0.1937
N200000930
0.1318
N200000790
N200000460
N200001004
0.1553
N200000997
0.3994
JJ00001224
0.153
0.2608
JJ00000458
0.232
N200000357
0.1442
0.2384
N200000257
N200001140
F200000465

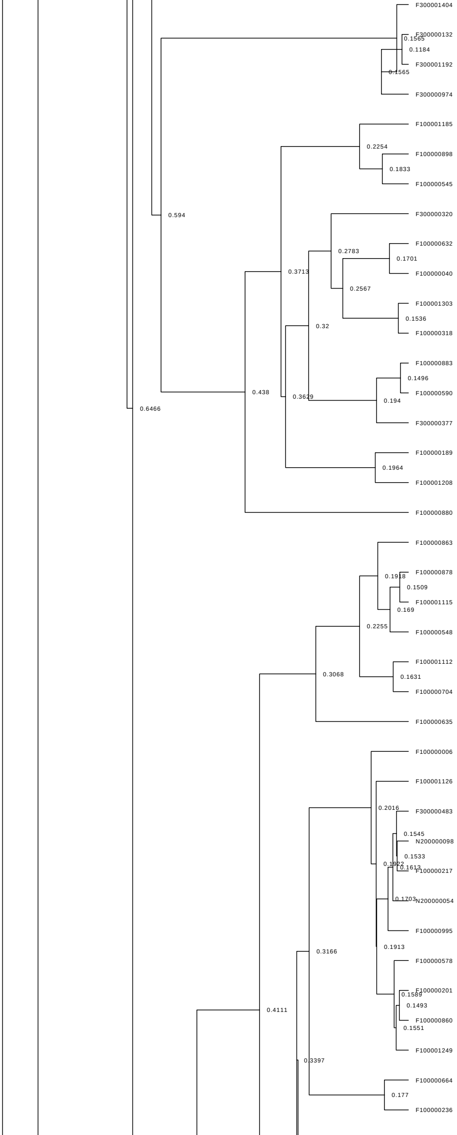
F300001404
F300000132
0.1565
0.1184
F300001192
0.1565
F300000974
F100001185
0.2254
F100000898
0.1833
F100000545
0.594
F300000320
0.2783
F100000632
0.1701
F100000040
0.3713
0.2567
F100001303
0.1536
F100000318
0.32
F100000883
0.1496
0.438
F100000590
0.3629
0.194
0.6466
F300000377
F100000189
0.1964
F100001208
F100000880
F100000863
F100000878
0.1918
0.1509
F100001115
0.169
0.2255
F100000548
F100001112
0.3068
0.1631
F100000704
F100000635
F100000006
F100001126
0.2016
F300000483
0.1545
N200000098
0.1533
0.1922
0.1613
F100000217
0.1703
N200000054
F100000995
0.1913
0.3166
F100000578
F100000201
0.1589
0.1493
0.4111
F100000860
0.1551
F100001249
0.3397
F100000664
0.177
F100000236

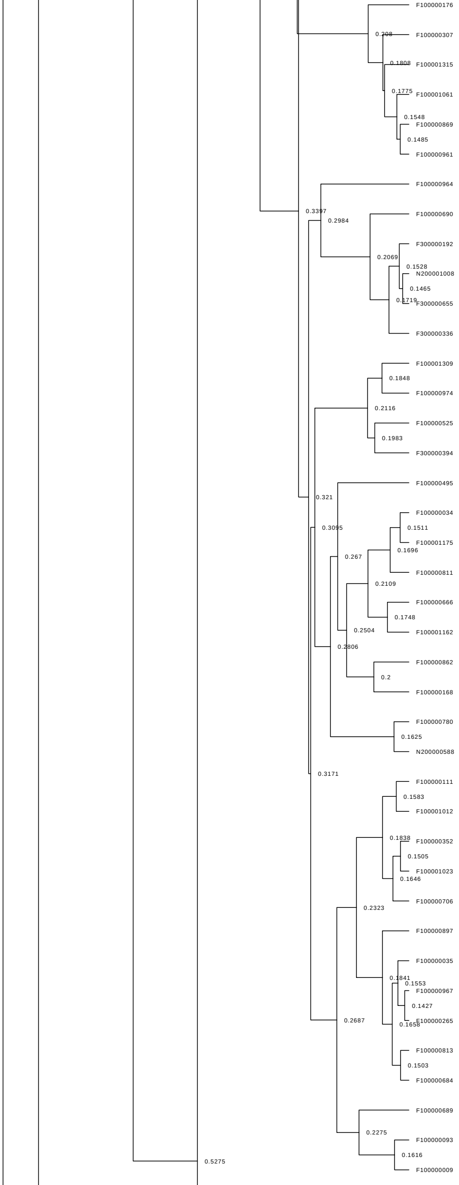
F100000176
0.208
F100000307
0.1808
F100001315
0.1775
F100001061
0.1548
F100000869
0.1485
F100000961
F100000964
0.3397
0.2984
F100000690
F300000192
0.2069
0.1528
N200001008
0.1465
0.1719
F300000655
F300000336
F100001309
0.1848
F100000974
0.2116
F100000525
0.1983
F300000394
F100000495
0.321
F100000034
0.3095
0.1511
F100001175
0.1696
0.267
F100000811
0.2109
F100000666
0.1748
0.2504
F100001162
0.2806
F100000862
0.2
F100000168
F100000780
0.1625
N200000588
0.3171
F100000111
0.1583
F100001012
0.1838
F100000352
0.1505
F100001023
0.1646
F100000706
0.2323
F100000897
F100000035
0.1841
0.1553
F100000967
0.1427
0.2687
F100000265
0.1658
F100000813
0.1503
F100000684
F100000689
0.2275
F100000093
0.1616
0.5275
F100000009

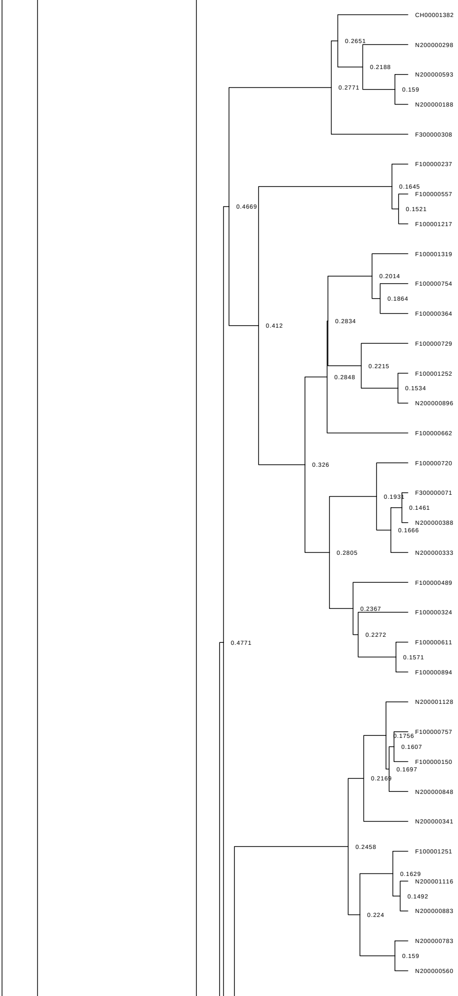

CH00001382
N200000298
N200000593
N200000188
F300000308
F100000237
F100000557
F100001217
F100001319
F100000754
F100000364
F100000729
F100001252
N200000896
F100000662
F100000720
F300000071
N200000388
N200000333
F100000489
F100000324
F100000611
F100000894
N200001128
F100000757
F100000150
N200000848
N200000341
F100001251
N200001116
N200000883
N200000783
N200000560
0.2651
0.2188
0.159
0.2771
0.1645
0.1521
0.4669
0.2014
0.1864
0.2834
0.412
0.2215
0.2848
0.1534
0.326
0.1931
0.1461
0.1666
0.2805
0.2367
0.2272
0.1571
0.4771
0.1756
0.1607
0.1697
0.2169
0.2458
0.1629
0.1492
0.224
0.159

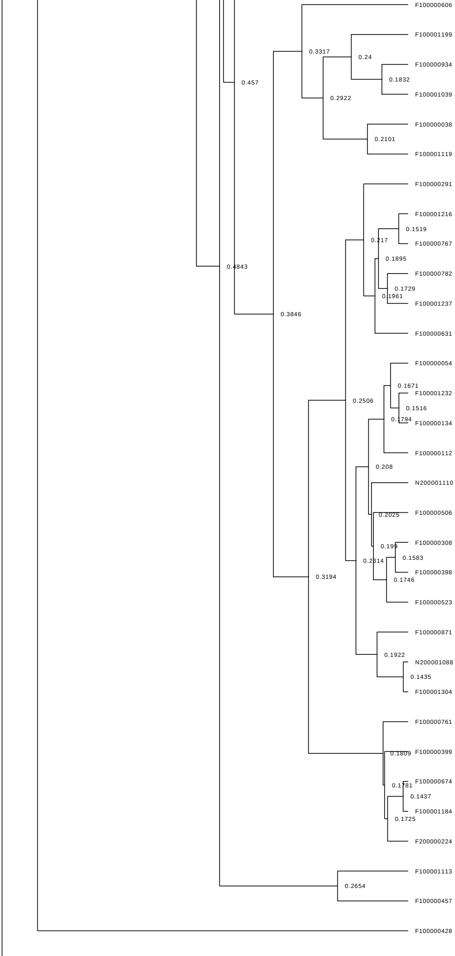

F100000606
F100001199
0.3317
0.24
F100000934
0.457
0.1832
F100001039
0.2922
F100000038
0.2101
F100001119
F100000291
F100001216
0.1519
0.217
F100000767
0.1895
F100000782
0.1729
0.1961
F100001237
0.4843
0.3846
F100000631
F100000054
0.1671
F100001232
0.2506
0.1516
0.1794
F100000134
F100000112
0.208
N200001110
F100000506
0.2025
F100000308
0.199
0.1583
0.2314
F100000398
0.1746
0.3194
F100000523
F100000871
0.1922
N200001088
0.1435
F100001304
F100000761
0.1809
F100000399
F100000674
0.1781
0.1437
F100001184
0.1725
F200000224
F100001113
0.2654
F100000457
F100000428

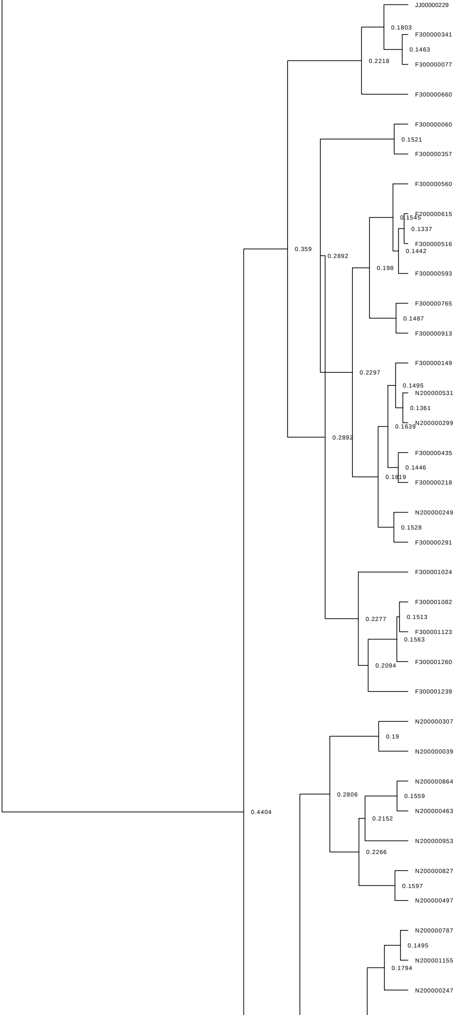
JJ00000229
0.1803
F300000341
0.1463
0.2218
F300000077
F300000660
F300000060
0.1521
F300000357
F300000560
0.1545
F200000615
0.1337
F300000516
0.1442
0.359
0.2892
0.198
F300000593
F300000765
0.1487
F300000913
F300000149
0.2297
0.1495
N200000531
0.1361
N200000299
0.1639
0.2892
F300000435
0.1446
0.1819
F300000218
N200000249
0.1528
F300000291
F300001024
F300001082
0.1513
0.2277
F300001123
0.1563
F300001260
0.2094
F300001239
N200000307
0.19
N200000039
N200000864
0.1559
0.2806
N200000463
0.4404
0.2152
N200000953
0.2266
N200000827
0.1597
N200000497
N200000787
0.1495
N200001155
0.1794
N200000247

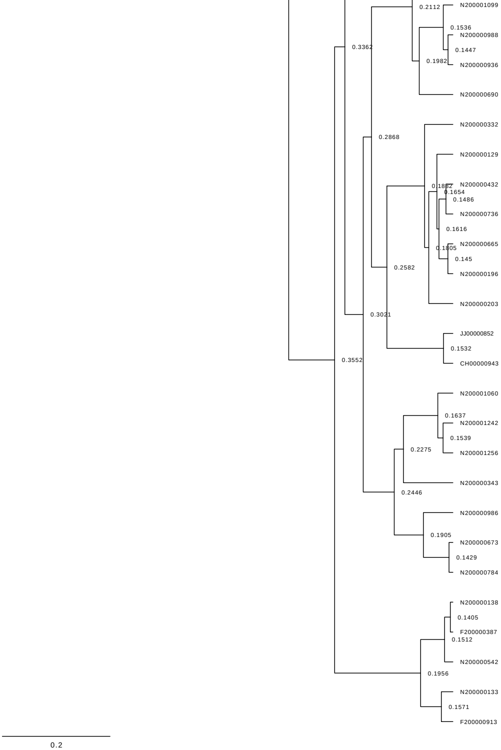
N200001099
N200000988
N200000936
N200000690
N200000332
N200000129
N200000432
N200000736
N200000665
N200000196
N200000203
JJ00000852
CH00000943
N200001060
N200001242
N200001256
N200000343
N200000986
N200000673
N200000784
N200000138
F200000387
N200000542
N200000133
F200000913
0.2112
0.1536
0.1447
0.1982
0.3362
0.2868
0.1882
0.1654
0.1486
0.1616
0.1805
0.145
0.2582
0.3021
0.1532
0.3552
0.1637
0.1539
0.2275
0.2446
0.1905
0.1429
0.1405
0.1512
0.1956
0.1571
0.2
